# Evolution of Hemoglobin Genes in Codfishes Influenced by Ocean Depth

**DOI:** 10.1038/s41598-017-08286-2

**Published:** 2017-08-11

**Authors:** Helle Tessand Baalsrud, Kjetil Lysne Voje, Ole Kristian Tørresen, Monica Hongrø Solbakken, Michael Matschiner, Martin Malmstrøm, Reinhold Hanel, Walter Salzburger, Kjetill S. Jakobsen, Sissel Jentoft

**Affiliations:** 1 Department of Biosciences, Centre for Ecological and Evolutionary Synthesis (CEES), University of Oslo, Oslo, Norway; 2 Institute of Fisheries Ecology, Johann Heinrich von Thünen-Institute, Federal Research Institute for Rural Areas, Forestry and Fisheries Hamburg, Germany; 3 Zoological Institute, University of Basel, Basel, Switzerland; 4 Department of Natural Sciences, Centre for Coastal Research, University of Agder, Kristiansand, Norway

## Abstract

Understanding the genetic basis of adaptation is one of the main enigmas of evolutionary biology. Among vertebrates, hemoglobin has been well documented as a key trait for adaptation to different environments. Here, we investigate the role of hemoglobins in adaptation to ocean depth in the diverse teleost order Gadiformes, with species distributed at a wide range of depths varying in temperature, hydrostatic pressure and oxygen levels. Using genomic data we characterized the full hemoglobin (*Hb*) gene repertoire for subset of species within this lineage. We discovered a correlation between expanded numbers of Hb genes and ocean depth, with the highest numbers in species occupying shallower, epipelagic regions. Moreover, we demonstrate that the *Hb* genes have functionally diverged through diversifying selection. Our results suggest that the more variable environment in shallower water has led to selection for a larger *Hb* gene repertoire and that *Hb*s have a key role in adaptive processes in marine environments.

## Introduction

Understanding the genetic basis for how organisms adapt to specific environments is a fundamental challenge within evolutionary biology. The use of model systems allowing genetic manipulation has in many cases shown to be a powerful approach^1–3^. However, for non-model species - including deep-sea and cold-adapted teleost fish - with limited options for experimental manipulations and where little pre-existing knowledge is available, comparative genomics is a powerful route to apply^4, 5^. The teleost fish lineage Gadiformes – the codfishes – is a large group of 610 species with substantial ecological and economic impact distributed across the world in a wide variety of marine and freshwater environments. Most Gadiformes species are characterized as benthopelagic, i.e. neutrally buoyant in close association with the sea floor, and they inhabit almost every section of the continental slope, from shallow waters to the deep sea^6^. Fossil and phylogenetic evidence suggest that the ancestor of codfishes was a deep-sea fish, which later diversified into species inhabiting shallower waters^7–9^. Elucidation of biological traits crucial for such evolutionary transitions is not trivial, particularly because deep-sea adapted species are not well studied. The large latitudinal and vertical ranges in which Gadiformes reside represent a wide span of environmental conditions, including temperature, dissolved oxygen and hydrostatic pressure^10^. The hemoglobin (*Hb*) gene-family is a prime candidate for investigating molecular adaptation to depth because the relationship between the structure and function and how this links to environmental factors is well characterized.

The Hb protein is a key component of respiration, and consists of two *α*- and *β*-globins subunits assembled as a tetramer. Teleost fish have evolved a more diverse *Hb* repertoire compared to other vertebrates due to being ectotherms and water-breathers, and thus exposed to a wider span in temperatures and oxygen availability^11^. The foundation for this diverse repertoire is partially associated with the teleost whole-genome duplication (TGD) 320–400 Ma^12^, which resulted in two *Hb* clusters located on different chromosomes; the LA cluster and the MN cluster, respectively^11, 13^. Such duplication events provide raw evolutionary material allowing species to acquire new biological functions when additional copies are relieved from the functional constraints associated with the original gene^14, 15^. The genome sequencing of Atlantic cod (*Gadus morhua*)^16^ confirmed that it contains four *α* genes and five *β* genes^17^, and this high multiplicity of *Hb* genes might increase its ability to respond to different environmental conditions^17, 18^. Furthermore, the *β1* gene displays a clear latitudinal gradient between two haplotypes, which may be linked to temperature adaptation^19, 20^. Hb could consequently be important for adaptation to the variety of environments occupied by the other gadiform species.

Here, we examine the evolutionary history of *Hb* in a wider phylogenetic perspective using low coverage (approx. 30x) genome sequencing to characterize the full *Hb* repertoire across the Gadiformes lineage. We selected 27 gadiform species, as well as 3 closely related outgroup species^9^: *Stylephorus chordatus* (closest living relative of Gadiformes^9^), *Zeus faber* and *Percopsis transmontanta*. Combining the extracted *Hb* gene repertoire with data on depth and latitude of occurrence we show that the expanded number of *Hb* genes found within this lineage is negatively correlated to depth, with the highest numbers in species living in shallower or epipelagic regions. Furthermore, *in silico* modeling of the Hb tetramer combined with comprehensive tests of natural selection revealed strong signs of diversifying selection on the surface of the protein structure indicating that the different variants have functionally diverged. Taken together, our findings suggest that the evolution of the hemoglobin gene repertoire has played a fundamental role in the Gadiformes’ adaptation to the wide range of depths it occupies today.

## Results/Discussion

### Expansion of Hb Genes in Codfishes

**Figure 1:**
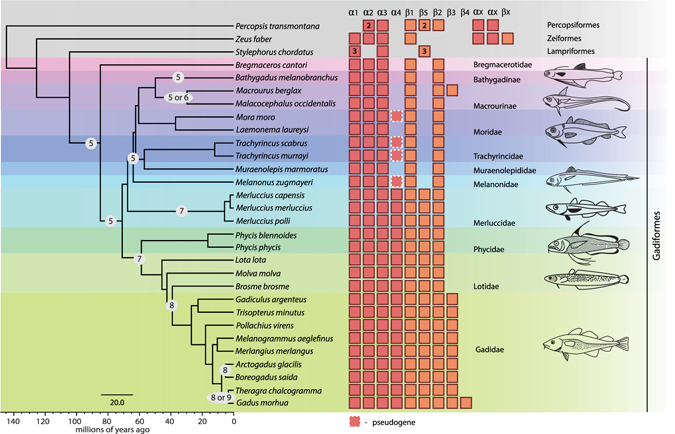
The repertoire of hemoglobin genes in the Gadiformes. The number of *Hb*s in 27 species of Gadiformes, as well as three outgroup species from Stylephoriformes, Zeiformes and Percopsiformes, are mapped onto a time-calibrated molecular phylogeny. This phylogeny is part of a larger teleost phylogeny presented in ref. 9. *α*- and *β*-globin genes are indicated by boxes. Some species have more than one copy of a gene, which is indicated by a number. *α*x and *β*x refer to *α*- and *β* genes that are not 1:1 orthologs to the gadiform *Hb* genes. The ancestral *Hb* copy number with the highest likelihood is indicated at nodes where there has been an evolutionary change, as well as any ambiguity (Supplementary Fig. 1). Time is given in million years. Fish illustrations drawn by Geir Holm are reprinted with permission from ref. 9.

**Figure 2:**
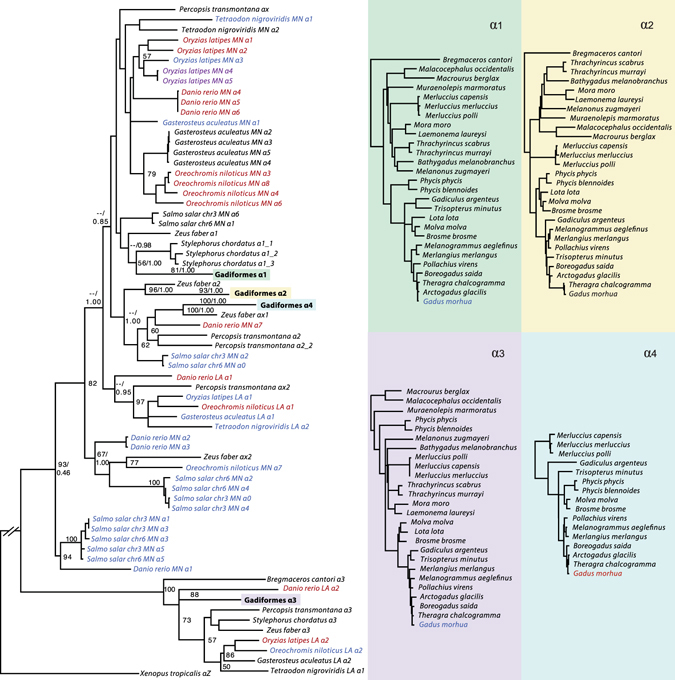
Phylogenetic relationships of *α*-globin genes. ML phylogeny of *α*-globin genes from 36 species of teleosts, and western clawed frog (*Xenopus tropicalis*) as the outgroup species. Numbers on nodes show bootstrap values and Bayesian posterior probabilities where topology is concordant, -- denotes support lower than 50/0.50. Sequences are colored according to timing of expression^11, 45^; embryonic (red), adult (blue), embryonic and adult (purple) and unknown (black). For each gadiform *α*-globin gene the phylogenetic tree is shown separately. Some branches are shortened for convenience, which is indicated by gaps.

**Figure 3:**
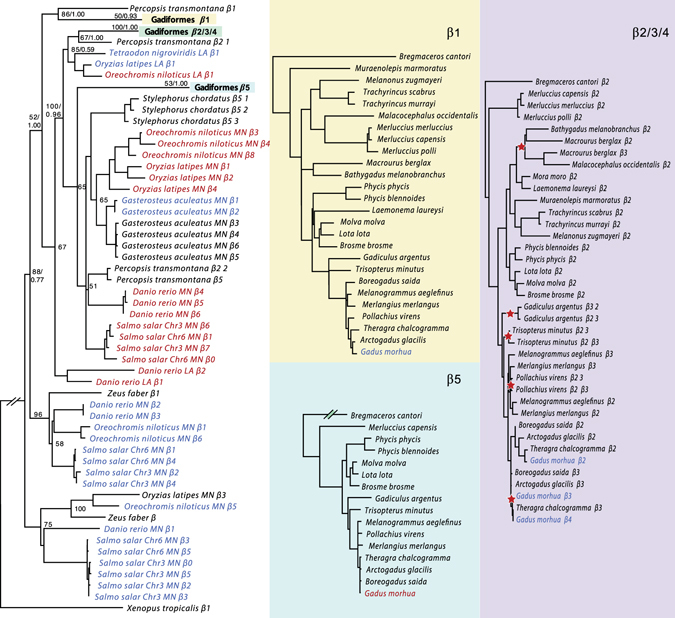
Phylogenetic relationships among *β*-globin genes. ML phylogeny of *β*-globin genes from 36 species of teleosts, and western clawed frog (*Xenopus tropicalis*) as the outgroup species. Numbers on nodes show bootstrap values and Bayesian posterior probabilities where topology is concordant, -- denotes support lower than 50/0.50. Sequences are colored according to timing of expression^11, 45^; embryonic (red), adult (blue) and unknown (black). For each gadiform *β*-globin gene the phylogenetic tree is shown separately. Lineage specific duplications of *β*2 are indicated by red stars. Some branches are shortened for convenience, indicated by gaps.

A full characterization of the *Hb* genes was obtained from the genome sequence data of the 30 selected species^9^ (Supplementary Table 1). The number of *Hb* genes varied from five to nine in extant Gadiformes (Fig. 1). The ancestral state at the root of the Gadiformes lineage was estimated to five *Hb* genes (Supplementary Fig. 1), which indicates an overall expansion of the *Hb* repertoire within Merlucciidae, Phycidae, Lotidae and Gadidae (Fig. 1 and Supplementary Fig. 1). The observed variation in *Hb* gene number is indicative of gene duplications and/or gene deletions in different lineages. Construction of phylogenetic trees for all *α*- (Fig. 2) and *β*-globin (Fig. 3) sequences, including sequences from *Danio rerio, Oreochromis niloticus, Gasterosteus aculeatus, Oryzias latipes, Salmo salar* and *Xenopus tropicalis*, the latter as an outgroup species, enabled proper gene annotation of the *α*- and *β*-sequences. In concordance with previous phylogenies of teleost *Hb*s^11, 17^ our gene trees reveal the dynamic nature of *Hb* family gene evolution. For the gadiform *α-*globins, *α*1, *α*2, *α*3 and *α*4 form monophyletic groups (Fig. 2) with *α*1, *α*2, and *α*4 showing high sequence similarity, which indicates recent gene duplications. However, due to high degree of sequence similarity in *α-*globins across all teleosts, either as a result of homoplasy, gene conversion or sequence conservation, the phylogenetic relationship between the clades containing *α*1, *α*2, and *α*4 could not be resolved with sufficient statistical support (i.e. bootstrap or posterior probabilities). Furthermore, it seems like *α*4 has been lost, or pseudogenized, as indicated by premature stop codons or frame-shifting indels in the clade flanked by *Bregmaceros cantori* and *Melanonus zugmayeri* (Fig. 1). The phylogeny of the *β-*sequences suggests that *β1*, the ancestor of *β2*/3/4 and *β5*, represents the ancestral *β* genes in Gadiformes (Fig. 3). Additionally, the gene tree indicates that *β*2 has been duplicated and giving rise to *β3* in Gadidae – and again independently duplicated in *Macrourus berglax* – implying that *β3* in *Macrourus berglax* and Gadidae are not strict 1:1 orthologs (Fig. 3). Within Gadidae, *β2* seems to have been duplicated independently to form *β3* in *Gadiculus argentus* and *Trisopterus minutus*, respectively. This may call for renaming of *β3* in these species. In *Gadus morhua β3* has been duplicated giving rise to *β4*. Overall, the observed expansion of *Hb* genes in Gadiformes relative to the ancestral state is indicative of an increased repertoire of combinatory Hb tetramers, likely contributing to a high respiratory plasticity.

**Figure 4:**
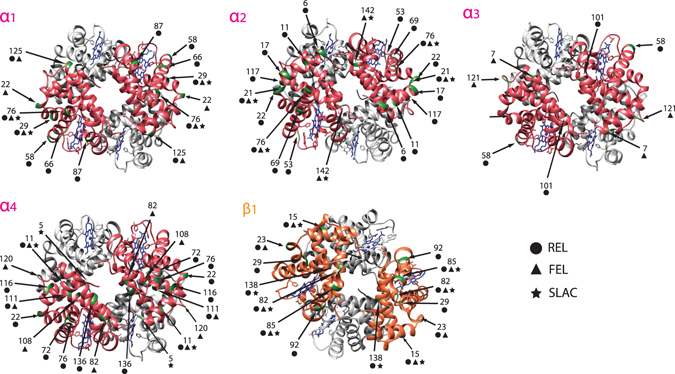
Sites under diversifying selection at the surface of hemoglobin tetramers. *In silico* models of the hemoglobin tetramers, based on sequences from Atlantic cod (*Gadus morhua*). *α* sequences are highlighted in pink, and *β* sequences in orange, with respective gene names shown. Three different tests (indicated by symbols according to the key) were used to test for diversifying selection; REL, FEL and SLAC, respectively. Arrows point to sites under diversifying selection, which are also highlighted in green.

According to the birth-death model of gene family evolution, new genes are often redundant or deleterious, and are consequently pseudogenized or lost^14, 21^. If the new gene confers some advantage to the organism either by increasing gene dosage or acquiring a new function/sub-function, it will be maintained by natural selection. Thus, duplicated *Hb* genes could have slightly different oxygen-carrying properties due to diversifying or positive selection, i.e. evolutionarily optimized for functionality at different environmental conditions. To address this, we tested whether the identified *Hb* genes within the Gadiformes have diversified faster than expected by chance with multiple site-specific tests (SLAC, FEL and REL) for natural selection based on the nonsynonymous (dN)/synonymous (dS) substitution rate ratio within the different *Hb* genes. For *α1*, *α2*, *α3, α4*, *β1* and *β5* many sites were under diversifying selection (dN/dS > 1) (Fig. 4, Supplementary Table 3). For the three tests of natural selection (REL, FEL and SLAC, respectively) that were carried out, not all tests reported the same sites (Fig. 4, Table S3) due to different underlying models and assumptions affecting evolutionary inference. The SLAC test is the most conservative with a low false positive rate, however, SLAC sometimes misses sites that are under selection. The less stringent tests (FEL, followed by REL) usually identify more sites, with the cost of a higher type I error-rate^22^. These tests can either be used on species level phylogenies or on phylogenetic gene trees. Because species and gene phylogenies might not have the exact same topology, we performed the tests at both levels. Notably, many sites were consistently reported using either a species tree or a gene tree by two or all of the three tests. These sites are therefore likely to be the most important for the evolution of divergent functions (Fig. 4, Table S3). Moreover, most sites reported under natural selection were found to be under purifying selection (dN/dS < 1) for all genes, as many domains are conserved to uphold the function of *O*_*2*_ transport (Table S3). Since *β2*, *β3* and *β4* are more similar due to a recent duplication (Fig. 3), uncertainties regarding true 1:1 orthology did not allow for dN/dS tests.

Sites showing diversifying selection are indicative of sub-functionalization of the different *Hb* genes. To further evaluate whether the sites identified to be under diversifying selection have any potential impact on the actual function of the *Hb* tetramer, we constructed a structural protein model based on *G. morhua* hemoglobin sequences and plotted these sites onto the structure (Fig. 4). Most of the sites in gadiform *Hb* genes appear to be on the surface of the tetramer (Fig. 4), which again supports sub-functionalization, as substitutions on the surface of the protein affect Hb subunit interactions and affinities, and are thus likely to influence its O_2_ binding capacity under different environmental conditions (reviewed in ref. 23).

### The Hb Copy Number is Inversely Correlated with Depth

To investigate the relationship between the number of *Hb* genes observed and environmental factors – e.g. the geographical and vertical distribution of the Gadiformes (Table 1) – we used a phylogenetic comparative approach implemented in the R program SLOUCH^24^. For vertical distribution, we chose to use maximum depth (Table 1) as an indicator of depth of usual occurrence since benthopelagic species usually swim near the bottom^25^. In the best model (according to its AIC_c_-score) *Hb* copy numbers have evolved towards optima that are functions of maximum depth. This model explained about 28% of the variance in *Hb* copy number (Supplementary Table 2) and returned an optimal regression slope of −0.018, which implies a reduction of two copies with every 100 meters added to maximum depth. However, species are far from realizing this optimal relationship between depth and copy number since evolution of copy number towards the maximum depth optimum is extremely slow (Supplementary Table 2). Additionally, the southernmost latitudinal distribution of the species (Table 1) explained about 25% of the variance in *Hb* copy number, but that model has a substantially higher (i.e. worse) AIC_c_ score compared to the model using only maximum depth as predictor (Supplementary Materials and Methods).

**Table 1:** The latitudinal distribution and depth of occurrence for the species of Gadiformes included in this study. Latitude is given in degrees north (°N) for the most southern and most northern observation for each species, respectively, as well as the range. Depth of occurrence is given in meters (m), with minimum and maximum depth recorded for each species, including range.

Species	Latitude (°N)			Depth (m)		
	South	North	Range	Min	Max	Range
*Gadus morhua*	35	83	48	0	600	600
*Arctogadus glacilis*	69	87	18	0	1000	1000
*Boreogadus saida*	52	87	35	0	400	400
*Trisopterus minutus*	28	68	40	1	440	439
*Pollachius virens*	33	77	44	37	364	327
*Melanogrammus aeglefinus*	35	79	44	10	450	440
*Merlangius merlangus*	35	72	37	10	200	190
*Theragra chalcogramma*	34	68	34	0	1280	1280
*Gadiculus argentus*	24	74	50	100	1000	900
*Phycis phycis*	13	45	32	13	614	601
*Molva molva*	35	75	40	100	1000	900
*Lota lota*	40	78	38	1	700	699
*Brosme brosme*	37	83	46	18	1000	982
*Merluccius merluccius*	18	76	58	70	1000	930
*Merluccius capensis*	11	37	26	50	1000	950
*Merluccius polli*	−19	29	48	50	910	860
*Melanonus zugmayeri*	−49	60	109	0	3000	3000
*Macrourus berglax*	37	82	45	100	1000	900
*Malacocephalus occidentalis*	−37	43	80	140	1945	1805
*Bathygadus melanobranchus*	−34	53	87	400	2600	2200
*Muraenolepis marmoratus*	−56	−44	12	30	1600	1570
*Bregmaceros cantori*	NA	NA	NA	450	475	25
*Mora moro*	−51	64	115	450	2000	1550
*Laemonema laureysi*	−8	8	16	200	618	418
*Trachyrincus murrayi*	NA	NA	NA	0	1630	1630
*Phycis blennoides*	20	71	51	10	1047	1037
*Trachyrincus scabrus*	−27	55	82	395	1700	1305

The significant decline in number of gadiform *Hb* genes with depth of occurrence (Supplementary Table 2) suggests that the diversification from ancestral deep-sea habitats into more shallow zones has been facilitated by an expansion of *Hb* genes within the Gadiformes. Our analyses therefore suggest that the number of *Hb* genes is a result of adaptive evolution, albeit slow, and that the optimal number of *Hb* genes in different branches of the phylogeny is related to maximum depth, with a broader *Hb* repertoire in species living in more shallow waters. However, given that 72% of the variation remains unexplained in our best model, other factors than maximum depth may be important for the evolution of *Hb* copy number. Furthermore, an Ornstein–Uhlenbeck process is the simplest stochastic model that allows evolution toward a specific state, and we acknowledge that the evolution of *Hb* copy number in the Gadiformes may not be perfectly described by this model. Nevertheless, 28% is a high number given the complexity of the interaction between ecological factors and evolution and diversification of codfishes over millions of years.

It should be emphasized that it might not be depth *per se* that is the environmental factor affecting the evolution of *Hb* copy number evolution. Suggestively, other abiotic factors that correlate with depth might be the causal drivers, such as hydrostatic pressure and temperature, which are both known to influence the function of the Hb tetramer^26^. Hydrostatic pressure increases with roughly 1 atm every 10 meters and temperature generally drops with depth^10^. Deep-sea fishes are thus exposed to enormously high pressure and low temperatures (0–6 °C) which require special adaptations physiologically and at the molecular level^27^, including the Hb repertoire^26^. However, deep-sea adaptation may not require a broad repertoire of *Hb* genes since it represents an extreme, yet stable environment. Furthermore, decreasing temperature increases Hbs O_2_ affinity, while metabolic rate is negatively correlated with depth; this implies that oxygen demands are readily met^28^ with a relatively narrow Hb repertoire. In contrast, species exposed to a wider range of depths are dependent on a repertoire of Hbs with O_2_ affinities optimized for different temperatures and pressures, especially in shallow zones with highly fluctuating temperatures.

The adaptive significance of *Hb* multiplicity in teleost fishes in general is not clear-cut, and the link between multiplicity and environmental variation has been questioned by some reports^29, 30^. There are, however, few experimental studies comparing closely related species in a phylogenetic context. One such study conducted on triplefin fishes (family Tripterygiidae) demonstrated that species in shallow waters have a greater diversity of Hbs and a higher O_2_ affinity and reduced pH sensitivity than species living in deeper and more temperature stable habitats^31^. In concordance with these findings, goldfish (*Carassius auratus*) express a larger Hb repertoire when acclimated to a more variable temperature regime^32^. Antarctic Notothenioid fishes are cold-water specialists – adapted to a thermally stable climate – displaying an extremely narrow Hb repertoire, even a complete loss in the Channichthyidae family (reviewed in ref. 33). On the other hand, in mullets no connection was found between Hb multiplicity and temperature variability^34^. Thus, the adaptive role of the size of the Hb repertoire in different codfishes may not be obvious. Demonstration of diversifying selection acting on several *Hb* genes, however, suggests that they are evolutionarily and functionally fine-tuned for different environmental conditions, also supported by the finding that most of these sites are on the surface of the Hb tetramer (Fig. 4). Numerous studies have demonstrated that *Hb* evolution is driven by temperature adaptation, which is not surprising given the fact that O_2_ affinity is negatively correlated with temperature^35^. Notably, Campbell *et al*.^36^ found several surface substitutions on the chimeric β/δ-globin subunit of mammoth hemoglobin that have a large phenotypic effect and contribute to O_2_ offloading at cold temperatures. In Atlantic cod two linked substitutions on the β1 gene with potentially the same effect has been identified in populations that are more cold-tolerant ^20^, although a recent report questions whether these substitutions are linked to temperature adaptation^37^. Further, equivalent substitutions have been linked to adaptation to altitudinal ranges in North American pikas^38^ and deer mice^39^.

Temperature and hydrostatic pressure are obvious candidates underlying depth adaptation, however, there could be other selective drivers correlated with depth shaping *Hb* copy number. Expressing different Hb tetramers could increase Hb solubility and thus cellular Hb concentration resulting in a higher O_2_ carrying capacity of the blood^30^. This could be of importance to species living in more shallow waters, which generally have higher metabolic rates^28^. Moreover, Hb heterogeneity is shown to boost cell longevity as well as affect cellular metabolism^30^. Biotic factors such as pathogen load may also play a role in depth adaptation, as the microbial community varies at different depths ^8, 40^. Hbs are known to have immune-related functions, such as Hb-derived antimicrobial peptides found in the channel catfish^41^ and humans^42^, or killing microbes directly by creating reactive oxygen species^43^. Further, many fish species are also exposed to diverse environments at different life stages. This likely promotes subfunctionalization due to various paralogs being differentially expressed during embryonic and larval development. Hemoglobin genes in teleosts do not cluster together phylogenetically according to the developmental timing of their expression (Figs 2 and 3)^11^, thus the evolution of *Hb* regulation seems to be less constrained in teleosts compared to birds and mammals^44^. In Atlantic cod all nine variants are expressed throughout their lifespan, however, *α1*, *β1*, *α2* and *β2* are the most common adult globins, whereas *β5* and *α4* are more highly expressed in juvenile fish^45^ ^17, 45^. In addition to timing of expression, neo-/subfunctionalization could involve expression in novel tissues, or different allosteric regulation^35^. Perhaps the most famous example of Hb subfunctionalization is the pH-dependent reduction in O_2_ carrying capacity known as the Root effect – involved in retinal oxygenation and swim bladder inflation (reviewed in ref. 46), but also generally enhances O_2_ delivery during stress^47^.

### The Evolutionary Transition from Obligate Deep-Sea Habitats to Various Depths and Shallow Zones Promoted a Large Hb Repertoire and Ecological Speciation

To our knowledge, no experiments have so far been carried out on deep-sea organisms to address the function of Hb in relation to hydrostatic pressure. However, Noble *et al*.^48^ investigated the functional properties of Hbs in five gadiform species (belonging to the Moridae and Macrouridae families) – occupying various depths – using carbon monoxide (CO) affinity as a proxy for O_2_ affinity under high hydrostatic pressure. They found that deep-sea species have heme groups with a much lower CO affinity than more shallow-water species, most likely an adaptation to allow pumping of O_2_ into the swim bladder under high-pressure conditions. On a broader scale, studies of other proteins show that functional properties in deep-sea species are basically unaffected by high pressure compared to species not occupying high-pressure environments^27, 49^. This suggests that the functionality of proteins in deep-sea organisms has evolved to withstand hydrostatic pressure. Accordingly, we postulate that during the evolutionary transition from obligate deep-sea habitats to more shallow zones, gadiform species have undergone an adaptive selection towards a broader Hb repertoire functionally optimized to low pressure as well as coping with fluctuating temperatures. Moreover, the specialized adaptation to an array of different ocean depths has most likely promoted ecological speciation within the Gadiformes. Ecological speciation along environmental gradients is seen as the major mode of speciation where strict geographic isolation is generally non-existent, such as in marine environments^50, 51^. In deep-sea populations, differentiation and speciation can occur along environmental gradients, attenuating with depth. This has been shown in bivalves^52^, corals^53^ and rockfish^54^. The weak relationship between number of *Hb* genes and geographical distribution observed in this dataset is in line with other studies that support isolation by depth as a stronger driver of population differentiation and ultimately speciation than isolation by distance in deep-sea taxa^52, 54^.

### Hb Gene Duplications and the High Diversification Rates in Codfishes

Investigating the evolutionary and ecological changes accompanying the transition from deep sea to more shallow waters is challenging as little is known about the ecology of deep-sea teleosts^10^. Here, we have shown that comparative genomic studies can provide vital insight into the evolutionary history of lineages where data is otherwise scarce. To conclude, we demonstrate that the evolution of the *Hb* gene repertoire in Gadiformes is characterized by gene duplications as well as losses, accompanied with high degree of gene diversification indicative of subfunctionalization. This is influenced by ocean depth and putatively linked to adaptation to temperature and hydrostatic pressure. Ultimately, our data suggest that hemoglobin plays an important role in the evolutionary puzzle explaining the diversification of Gadiformes, which has one of the highest diversification rates of teleosts^9^ and is one of the most species-rich teleost clades, with species displaying a wide distribution with respect to geographic and vertical range.

## Materials and Methods

### Specimen collection

Working with animals we always aim to limit the effect our research afflict populations and individuals. Whenever possible we collaborate with other sources, such as commercial fisheries or museums. This way, no animals need to be euthanized to serve our scientific purpose alone. The tissue samples used in this study are either from museum specimen or commercially fished individuals intended for human consumption. The commercially caught fish were immediately stunned by bleeding, following standard procedures by a local fisherman. Sampling in this manner does not fall under any specific legislation in Norway, but it is in accordance with the guidelines set by the ‘Norwegian consensus platform for replacement, reduction and refinement of animal experiments’ (www.norecopa.no). For more information regarding the samples see^9^.

### Whole-genome sequencing

We selected 27 species, which represent most of the lineages in the Gadiformes order, in addition to its closest living relatives, *Stylephorus chordatus*, *Zeus faber* and *Percopsis transmontanta* ^9, 55^. We sequenced paired end libraries with an average insert size of 350 bp (2 × 150 bp reads on Illumina HiSeq. 2000) with coverage ranging from 18 to 40x (average coverage 28x). This sequence strategy gives contigs spanning the average median gene-length^56^, making it ideal for finding and identifying genes, but without substantial gene-order information (synteny). The Celera assembler^57^ was used to assemble the genomes, with contig N50 ranging from 3.1 to 8.1 kb with an average of 4.1 kb. CEGMA^58^ and BUSCO^59^ were used to evaluate gene completeness; CEGMA gave, on average, complete or partial hits for 69% of the conserved eukaryotic genes included in the CEGMA analysis and BUSCO gave, on average, 68% of the conserved genes belonging to the Actinopterygii clade in the BUSCO analysis. A list of species with relevant genome statistics is given in Supplementary Table 1. For further information regarding the sequencing see^9, 55^.

### Gene mining and annotation

*Hb* genes were annotated by tBLASTn^60^ searches with known *Hb* sequences from *Gadus morhua*, *Oryzias latipes*, *Tetraodon nigroviridis*, *Oreochromis niloticus*, *Gasterosteus aculeatus, Salmo salar* and *Danio rerio* (annotation and nomenclature following^11^). For paralogous genes that have recently been duplicated or are similar due to gene conversion, gene copies can collapse in the assembly process. In contrast, with polymorphic genes alleles could be misjudged as copies. However, by manually inspecting alignments of intronic sequences it was possible to distinguish paralogous gene copies from alleles.

### Phylogenetic tree construction

To identify orthologous *Hb* sequences phylogenetic gene trees were constructed, α and β sequences were analyzed separately. Amino acid sequences were aligned using ClustalW^61^ as implemented in MEGA7^62^ with default settings for all species (alignments of α and β sequences are in Supplementary Data 1). Using the model selection tool in MEGA7 we determined that the best model (i.e. having the lowest AIC score) for molecular evolution was TN93 + G + I for α-sequences and GTR + G + I for β-sequences. Phylogenetic trees were constructed based on codon triplets using maximum likelihood (ML) implemented in MEGA7 and a Bayesian method in MrBayes 3.2.2^63^. A ML tree was constructed based on the models of molecular evolution stated above, with 1000 bootstrap replicates. Bayesian trees were run using standard priors, with four chains of simulations for 1 × 10^7^ generations sampling every 1 × 10^3^ generation. The GTR + G + I model was used for both α and β as the TN93 + G + I is not available in this program. A given run was considered to have reached convergence when the likelihood scores leveled off asymptotically. All trees sampled prior to convergence were discarded and support (posterior probability) was calculated based on a consensus of the last 2250 trees. Previous work on teleost *Hb*s shows that *Hb* from the frog *Xenopus tropicalis* is clearly outside the clade constituting teleost *Hb*s^11^, therefore it was chosen as an outgroup species.

The identified α and β genes were then mapped on a phylogenomic species tree based on 567 exons of 111 genes, selected after stringent filtering for single-copy orthologous markers. Branching times were estimated in BEAST v.2.2^64^ using a relaxed clock model and 17 fossil constraints. This phylogeny is a modified version from^9^, which describes the procedures in more detail.

### Ancestral reconstruction of the number of Hbs

The ancestral reconstruction of number of *Hbs* in gadiformes was estimated using the function ace implemented in the R package APE^65^. *Percopsis transmontana*, *Zeus faber* and *Stylephorus chordatus* were not included as many of the *Hbs* found in these species are not 1:1 orthologs with gadiform *Hbs*. We used maximum likelihood estimation of the ancestral state for discrete characters with three different models, an equal rates model (ER), an all rates different model (ARD) and a symmetrical model (SYM), goodness of fit was estimated using a Chi-square test. All statistics was carried out in R v3.1.3^66^.

### Phylogenetic comparative analyses

We used a phylogenetic comparative method called SLOUCH (Stochastic Linear Ornstein-Uhlenbeck models for Comparative Hypotheses)^24, 67–70^ implemented in R v3.1.3^66^, to investigate whether the number of *Hb* genes has evolved as a response to changes in maximum depth and latitude, respectively (data was obtained for the different species in the global information system FishBase^6^). The assumed model of trait evolution (trait is here the number of *Hb* genes) is an Ornstein-Uhlenbeck (OU) process, where the trait evolves towards an optimum that is assumed to be a linear function of a predictor *x*, as $\theta =a+{b}_{a}x$, the regression parameters are informative of the relationship between the optimum and the trait. The deterministic pull of the trait towards the optimum is can be quantified with the phylogenetic half-life, ${t}_{1/2}=\frac{\mathrm{ln}\,2}{\alpha },\,\,$the average time it takes for a species to move half the way from an ancestral state to a new optimum i.e. a half-life above zero indicates adaptation is not immediate. SLOUCH returns an “optimal regression”, which represents the best fit of the estimated primary optimum^67^ on *Hb* copy number. In other words, this optimal regression describes the expected relationship between the number of *Hb* genes and the predictor in the model if adaptation was instantaneous (i.e. there are no constraints on the evolution of number of *Hb* genes towards the optimal state). A model that includes a predictor variable can be contrasted with an intercept-only model where no predictor variables are included. Phylogenetic effect is a measure of how well the phylogeny alone explains the distribution of the trait (number of *Hb* genes). Model comparisons are done using the small sample-size corrected version of Akaike information criterion (AICc).

### Analyses of natural selection

For each Hb gene translated amino acid sequences from all species available for that gene in the dataset were aligned following same procedure as described above (alignments presented in Supplementary Data 1). To test for diversifying and purifying selection we used the SLAC, FEL and REL analyses^22^ as implemented in the Hyphy software package on the Datamonkey server (www.datamonkey.org) and using the phylogenies in Fig. 1 (referred to as the species tree), and Figs 2 and 3 (referred to as the gene trees).

### Homology model building

A 3D protein model was created using the SWISS-MODEL Workspace and the DeepView software^71^ for *Gadus morhua* Hb-I (α1 and β1) based on homology. A template search was carried out in the SWISS-MODEL Workspace, identifying hemoglobin from *Trematomus bernacchii* (Protein Data Bank (PDB) code 1HBH) as the best template. *Gadus morhua* α1, α2, α3, α4 and β1, respectively were aligned to the template in DeepView, the alignment was then submitted to the SWISS-MODEL Workspace under project mode. The automated modeler procedure gave one model with high quality (QMEAN4 = 1.34) of a Hb tetramer with two β1 units, and two alpha units of either α1, α2, α3 or α4. This gave four different Hb tetramers in total, which are all shown in Supplementary Data 2.

## Electronic supplementary material

Supplementary Info
