# Supplementary material for: Evolution of Hemoglobin Genes in Codfishes Influenced by Ocean Depth
Source: Sci Rep. 2017 Aug 11;7:7956. doi: 10.1038/s41598-017-08286-2 (PMC5554263; doi:10.1038/s41598-017-08286-2)
Supplement: Supplementary file 1 — Supplementary Info [file 41598_2017_8286_MOESM1_ESM.pdf]

Supplementary Information

Evolution of Hemoglobin Genes in Codfishes Influenced by Ocean Depth

Helle Tessand Baalsrud, Kjetil Lysne Voje, Ole Kristian Tørresen, Monica Hongrø Solbakken, Michael Matschiner, Martin Malmstrøm, Reinhold Hanel, Walter Salzburger, Kjetill S. Jakobsen and Sissel Jentoft

correspondence to: sissel.jentoft@ibv.uio.no

This PDF file includes:

Supplementary Materials and Methods
Supplementary Notes
Supplementary Figs. 1 to 3
Supplementary Tables 1 to 4
Supplementary Data 1 to 2

Supplementary Materials and Methods

Phylogenetic comparative analyses

We used a phylogenetic comparative method called SLOUCH (Stochastic Linear Ornstein-Uhlenbeck models for Comparative Hypotheses) [1-5], to investigate whether the number of *Hb*-genes has evolved as a response to changes in maximum depth and latitude, respectively (data was obtained for the different species in the global information system FishBase [6]). We assume that average copy number in a lineage can take any non-negative real number (i.e., intraspecific variation in copy numbers exist). The evolution of a trait in this comparative framework (here the number of *Hb*-genes) is modeled as an Ornstein-Uhlenbeck (OU) process around an optimal state modeled as a function of a predictor variable that is assumed to have an effect on the optimal trait state. The OU process contains a deterministic pull of the trait towards the optimal state, plus stochastic changes in the trait that can be interpreted as being due to unmeasured selective forces acting on the trait and genetic drift and can be expressed by the stochastic differential equation:

$$dy = -\alpha(y - \theta)dt + \sigma dB,$$

where dy represents changes in number of *Hb*-genes (any real number, not only integers), y, in a small time interval, dt, and dB is a white-noise process (i.e., independent, normally-distributed random changes with mean zero and unit variance). The optimum, θ is assumed to be a linear function of the predictor x, as $\theta = a + b_a x$, where a and b_a are regression parameters that are informative of the relationship between the optimum and the trait. The α parameter represents

the deterministic pull of the trait towards the optimum and how fast the new optimal state is approached can be quantified with the phylogenetic half-life, $t_{1/2} = \frac{\ln 2}{\alpha}$, the average time it takes for a species to move half the way from an ancestral state to a new optimum i.e. a half-life above zero indicates adaptation is not immediate.

SLOUCH returns an "optimal regression", which represents the best fit of the estimated primary optimum [2] on hemoglobin copy number. In other words, this optimal regression describes the expected relationship between the number of *Hb*-genes and the predictor in the model if adaptation was instantaneous (i.e. there are no constraints on the evolution of number of *Hb*-genes towards the optimal state). The stochastic component σ can be interpreted as evolutionary changes in hemoglobin copy number due to unmeasured selective forces and genetic drift. Generalized least squares are used for estimation of the regression parameters and maximum likelihood for estimation of α and σ^2 in an iterative procedure. For a full description of the model implemented in SLOUCH, see [1].

A model that includes a predictor variable can be contrasted with an intercept-only model where no predictor variables are included, which estimate the phylogenetic effect in the number of *Hb*-genes. Phylogenetic effect is a measure of how well the phylogeny alone explains the distribution of the trait. A half-life of zero in such an intercept-only model indicates that the trait is not phylogenetically structured, while a half-life > 0 indicates that there exists an influence of phylogeny on the trait distribution due to slowness of adaptation and/or adaptation towards phylogenetically structured optima. Comparing models with and without predictors is one way to investigate how much of the phylogenetic effect in a particular trait that can be accounted for by

adaptation of the response variable towards optima influenced by the included predictor variables. Model comparisons are done using the small sample-size corrected version of Akaike information criterion (AICc). All statistical analyses in relation to the comparative approach were done in R v3.1.3 [7].

Supplementary Notes.

Ancestral state reconstruction

The ancestral reconstruction of number of *Hbs* was estimated using the function ace implemented in the R package APE [8]. We used maximum likelihood estimation of the ancestral state for discrete characters with three different models: an equal rates model (ER), an all rates different model (ARD) and a symmetrical model (SYM). These gave log likelihood scores of -23.74, -20.69 and -19.8, respectively, implying that the ARD and SYM models gives the highest likelihoods. However, ARD has 20 parameters, compared to SYM with 10 parameters and ER with only one parameter. As the difference in the cumulative likelihood between any two models is distributed as chi square, we used a chi square test to find the model with the best fit, where the degrees of freedom is the difference in the number of parameters in the models. The p-values were 0.99 when comparing ER to ARD, 0.99 when comparing SYM to ARD and 0.55 when comparing ER to SYM, meaning that at a 5% significance level we did not accept the more parameterized models SYM or ARD. Subsequently, we used the ER model to calculate the ancestral states, as shown in figure S1. All statistics was carried out in R v3.1.3 [7].

Supplementary Figures

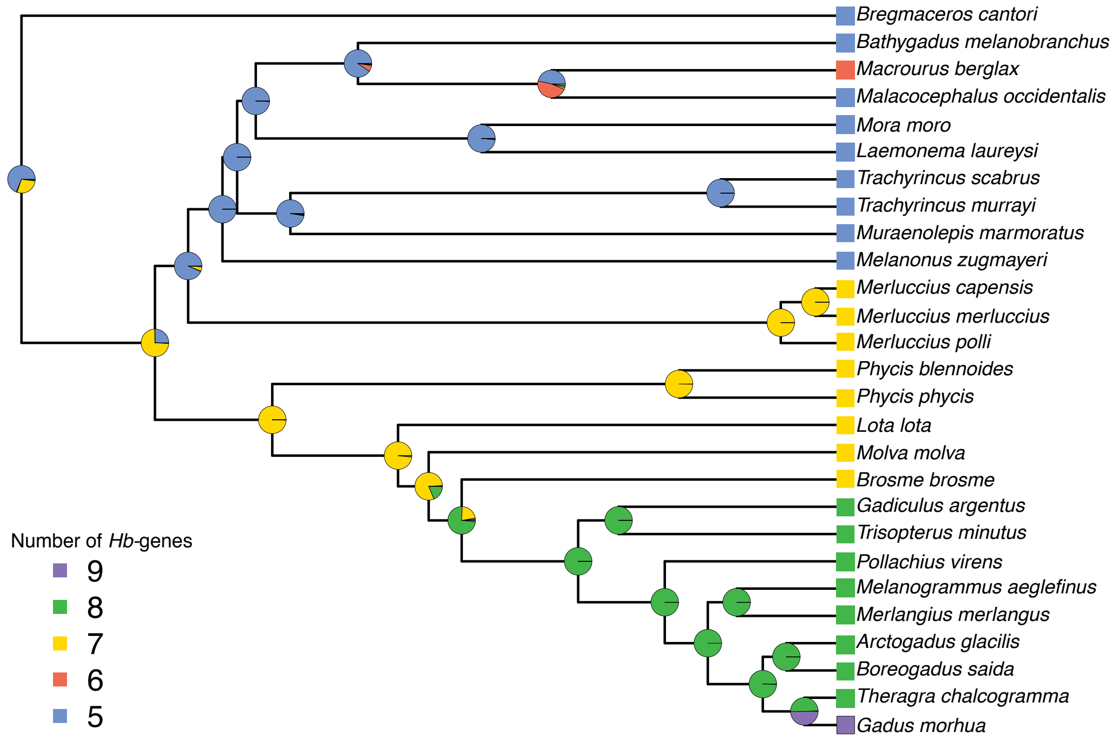

Supplementary Figure 1. Ancestral reconstruction of number of hemoglobin genes. The number of *Hb*-genes in each species is indicated by color as given in the legend. The pie charts illustrate the likelihood for each state (the number of *Hb*-genes) at each node

Supplementary Tables

Supplementary Table 1.

Sequencing statistics for the Celera assemblies of the genomes from species included in this study, including the library insert size, the estimated genome size, average coverage and N50 contig size given in number of base pairs (bp).

Order	Species	Insert size	Genome size	Coverage	N50 contig
Gadiformes	*Gadus morhua*	531	673980209	25.86	5765
Gadiformes	*Arctogadus glacilis*	322	646046698	27.54	3282
Gadiformes	*Boreogadus saida*	316	640664347	30.48	3221
Gadiformes	*Trisopterus minutus*	325	517406287	33.50	3248
Gadiformes	*Pollachius virens*	328	512964257	25.84	3457
Gadiformes	*Melanogrammus aeglefinus*	324	543479405	25.17	3215
Gadiformes	*Merlangius merlangus*	340	566197774	37.17	3538
Gadiformes	*Theragra chalcogramma*	322	660821200	29.43	3603
Gadiformes	*Gadiculus argenteus*	320	567051019	29.03	3379
Gadiformes	*Phycis blennoides*	505	673558859	31.24	4532
Gadiformes	*Phycis phycis*	328	467761927	32.04	3458
Gadiformes	*Molva molva*	329	539410497	33.68	4136
Gadiformes	*Lota lota*	341	512013778	30.51	3803
Gadiformes	*Brosme brosme*	342	550784471	26.78	3682
Gadiformes	*Merluccius merluccius*	359	610727262	29.65	3670
Gadiformes	*Merluccius capensis*	353	652894032	19.89	3792
Gadiformes	*Merluccius polli*	353	608812989	22.35	3471
Gadiformes	*Melanonus zugmayeri*	351	589223343	37.09	4562
Gadiformes	*Macrourus berglax*	356	692612974	21.18	3353
Gadiformes	*Malacocephalus occidentalis*	352	504123321	32.68	3697
Gadiformes	*Bathygadus melanobranchus*	330	576895271	26.32	4956
Gadiformes	*Muraenolepis marmoratus*	345	840377252	18.19	3126
Gadiformes	*Bregmaceros cantori*	343	1649969348	23.60	4452
Gadiformes	*Mora moro*	345	498760318	39.10	3267
Gadiformes	*Laemonema laureysi*	348	523675229	24.28	3431
Gadiformes	*Trachyrincus murrayi*	519	677614186	26.47	6231
Gadiformes	*Trachyrincus scabrus*	358x	579129872	37.96	6346
Stylephori -formes	*Stylephorus chordatus*	428	971441468	23.04	3373
Percopsi	*Percopsis transmontana*	350	509228518	35.57	8161

-formes					
Zeiformes	*Zeus faber*	335	732353037	22.92	4642

Supplementary Table 2.

Results of the comparative analyses. For all the reported models, the number of species the analyses (N), the maximum-likelihood estimates of the phylogenetic half-life in units of tree length (= 1), the stationary variance, phylogenetically corrected R^2, the estimated slope of the optimal regression with standard errors (SE), model fit described by the log likelihood (logL), and AIC_c score. Since we did not have latitudinal data for two of the species in the full phylogeny, we ran separate analyses of the latitudinal variables on a phylogeny where these two species had been removed (reduced phylogeny).

Full phylogeny	Predictor	N	half-life	stationary variance	R^2 (%)	Optimal regression slope (SE)	AIC_c	logL
	-	27	10.00	8.47	-	-	63.15	-28.05
	maximum depth	27	10.00	4.90	27.73	-0.018 (0.006)	56.80	-23.49
	minimum depth	27	4.35	3.53	5.43	-0.017 (0.014)	64.45	-27.32
	depth range	27	10.00	5.46	23.26	-0.017 (0.006)	58.45	-24.31
	max depth & depth range	27	10.00	2.45	34.09	max depth -0.061 (0.049) depth range 0.042 (0.050)	73.59	-30.36
Reduced phylogeny	-	25	10.00	7.45	-	-	57.80	-25.90
	latitudinal range	25	10.00	7.45	0.01	0.0087 (0.1440)	61.80	-25.90
	southern latitude	25	0.67	0.57	25.99	0.0394 (0.0131)	57.49	-23.74
	northern latitude	25	10.00	6.53	6.99	0.1733 (0.1258)	59.72	-24.86

Supplementary Table 3. Selection tests.

For each Hb-gene tested for natural selection using REL, FEL and SLAC, respectively, is shown the positions on the gene under positive selection as well as the number of positions under negative selection. Sites reported by analyses using gene trees and species trees are highlighted in bold. Sites only reported using gene trees are shown in parentheses.

	Gene	REL (Bayes)	FEL (ML)	SLAC
Sites under positive selection (position)	α1	**29**, 58, 66, **76**, **87**, (107),125	**22**, **29**, **76**, **125**	**29**, **76**
	α2	6, 11, 17, **21**, 22, 53, 69, **76**, 117	21, **76**, **142**	**21**, **76**, **142**
	α3	58, (69), **101**	7, **121**	(48, 121)
	α4	11	5, **11**, 82, 108, 111, **120**	**11**, 22, 72, 76, 111, 116, 136
	β1	**15**, **23**, 29, (31), **82**, 85, 92, 138	**15**, 23, **82**, **85**	**15**, 82, 85, 138
	β5	-	-	-
Number of sites under negative selection	α1	99	63	46
	α2	96	67	36
	α3	75	67	53
	α4	22	46	40
	β1	84	69	41
	β5	-	66	35

Additional Data 1: Alignment of alpha genes and beta genes

```
#NEXUS
BEGIN DATA;
  DIMENSIONS NTAX=159 NCHAR=435;
  FORMAT DATATYPE=DNA
  GAP=-
  ;
MATRIX
[1] Gadus_morhua_A1
ATG---AGTCTCACACCAAAGGACAAGGCGACCGTCAAGCTCTTCTGGGGCAGGATG---
TCCGGCAAGGCCGAGCTTATCGGCGCCGATGCTCTTTCAAGGATGCTCGCTGTGTACCCG
CAGACCAAGACTTACTTCAGCCACTGGAAGAGCCTCAGCCCTGGTTCCCCTGACGTGAAG
AAGCACGGCAAGACCATCATGATGGGCATCGGAGATGCTGTGACCAAGATGGACGACCTG
GAAAGGGGTCTTCTCACTCTGAGCGAGCTGCACGCCTTCAAGCTGAGAGTTGACCCCACC
AACTTCAAGCTTCTCTCACTCAACATCTTGGTGGTTATGGCTATTATGTTCCCTGATGAT
TTCACCCCAATGGCTCATTTGGCCGTCGACAAGTTCCTGTGTGCTTTGGCCCTGGCTCTG
TCCGAGAAGTACCGA
[2] Percopsis_transmontana_Ax2
ATG---AGTCTAACTCCGAAAGACAAGAATGCTGTCAAGGCGCTGTGGACAAAAATC---
TCCAGTAGGGCAGATGAGATCGGCGCCGTGGCTCTGTACAGGATGCTGACCGTCTATCCA
CAGACCAAGACTTACTTCTCCCATTGGACTGACATGGGCCCAAACTCCGCCCAAGTAAAG
AACCACGGGGAGAAGGTGATTGGGGAAATCGCTCTGGCAGTGGAGAAAATTGATAACTTG
ACCAGCGGGCTCCTCGAGCTCAGCGAGCTGCACGCCTTTAAATTAAGGATTGACCCCACC
AACTTCAAGATTCTGTCCCACAACATTCTTGTGTCGATAGCCAATGTTTTTCCGGACGAC
TTCACCCCGCAGGCGCATGTGGCTTTTGACAAGTTCCTGGTCAATGTGGCTCTGGCTCTC
GCTGAGCGGTACCGC
[3] Arctogadus_glacilis_A1
ATG---AGTCTCACACCAAAGGACAAGGCGACCGTCAAGCTCTTCTGGGGCAGGATG---
TCCGGCAAGGCCGAGCTTATCGGCGCCGATGCTCTTTCAAGGATGCTCGCTGTGTACCCG
CAGACCAAGATTTACTTCAGCCACTGGAAGAGCCTCAGCCCTGGTTCCTCTGAAGTGAAG
AAGCACGGCAAGACCATCATGATGGGCATCGGAGATGCTGTGACCAAGATGGAAGACCTG
GAAAGGGGTCTTCTCACTCTGAGCGAGCTGCACGCCTTCAAGCTGAGAGTTGACCCCACC
AACTTCAAGCTTCTCTCACTCAACATCTTGGTGGTTATGGCTATTATGTTCCCTGAGGAT
TTCACCCCAATGGCTCATTTGGCCGTCGACAAGTTCCTGTGTGCTTTGGCCCTGGCTCTG
TCCGAGAAGTACCGA
[4] Boreogadus_saida_A1
ATG---AGTCTCACACCAAAGGACAAGGCGACCGTCAAGCTCTTCTGGGGCAAGATG---
TCCGGCAAGTCCGAGCTTATCGGCGCCGATGCTCTTTCAAGGATGCTCGCTGTGTACCCG
CAGACCAAGATTTACTTCAGCCACTGGAAGAGCTGCAGCCCTGGTTCCCCTGAAGTGAAG
AAGCACGGCAAGACCATCATGATGGGCATCGGAGAAGCTGTGACCAAGATGGACGACCTG
GAAAGGGGTCTTCTCACTCTGAGCGAGCTGCACGCCTTCAAGCTGAGAGTTGACCCCACC
AACTTCAAGCTTCTCTCACTCAACATCCTGGTGGTTATGGCTATTATGTTCCCTCTGGAT
TTCACCCCAATGGCTCATTTGGCCGTCGACAAGTTCCTGTGTGCTCTGGCCCTGGCTCTG
TCCGAGAAGTACCGA
[5] Trisopterus_minutus_A1
ATG---AGTCTCACACCCAAGGACAAGGCGACCGTCAAGCTCTTCTGGAGCAAGATG---
CACGGAAAAGCCGAGAACATCGGCGCCGATGCTCTTTCTAGGATGCTCGCGGTGTACCCG
CAGACCAAGACCTACTTCAGCCACTGGAAGAACCTTGGCCAGGGCTCCGCAGAGGTGAAG
AAGCACGGCAAGGTCATCATGCAGGGCATCGGAGAGGCTGTGGGCAAGATGGACAACCTG
GAACAGGGCCTCCTCGCTCTGAGCGAGCTGCACGCCTTCAAGCTGAGGGTTGACCCCACC
AACTTCAGGCTTCTCTCCCTCAACATTGTGGTGGTTATGGCTATAATGTTCCCTGATGAG
TTCGACCCAGTGTCTCACTTGGCCGTGGACAAGTTCCTGTGCGCCCTGGCCCTGGCCCTG
TCCGAGAAGTACCGA
[6] Pollachius_virens_A1
```

ATG---AGCCTCACACCAAAGGACAAGGCGACCGTCAAGCTCTTCTGGAGCAAGATG---
TCTTCCAAGGCCGAGCTTATCGGCGCCGATGCTCTTTCAAGGATGCTGGCTGTGTACCCG
CAGACCAAGACTTACTTCAGCCACTGGAAGAGCGTTAGCCCTGGTTCCTCTGAAGTGAAG
AAGCACGGCAAGGTCATCATGATGGGCATCGGAGATGCTGTGGGCAAGATGGACGACCTG
GAAAGGGGCCTCCTCAGTCTGAGCGAGCTGCACGCCTTCAAGCTGAGAGTTGACCCCACC
AACTTCAAGCTTCTCTCACTCAACATCTTGGTGGTTATGGCTATAATGTTCCCTGATGAT
TTCACCCCAATGGCTCATTTGGCAGTGGACAAGTTCCTGTGTGCTCTGGCCCTGGCTCTG
TCCGAGAAGTACCGA
[7] Melanogrammus_aeglefinus_A1
ATG---AGCCTCACACCAAGGGACAAAGCGACCGTCAAGCTCTTCTGGAGCAAGATG---
GCCAACAAGGCCGAGCTTATCGGAGCCGATGCTCTTTCAAGGATGCTCGCTGTGTACCCG
CAGACCAAGACTTACTTCAGCCACTGGAAAAGCCTTAGCCCTGGATCCGCTGAAGTGAGG
AAGCACGGCAAGACCATCATGGGTGGCATCGGAGACGCTGTGAGCAAGATGGACGACCTG
GACAGGGGTCTCCTCTCTCTGAGCGAGCTGCACGCCTTCAAGCTGAGAGTTGACCCCACC
AACTTCAAGCTTCTCTCACTCAACATCGTGGTGGTTATGGCTATTATGTTCCCTGAGGAT
TTCACCCCAATGGCTCATTTGGCCGTCGATAAGTTCCTGTGTGCTCTGGCCCTGGCTCTG
TCCGAGAAGTACCGA
[8] Merlangius_merlangus_A1
ATG---AGCCTCACACCAAAGGACAAGGCGACCGTCAAGCTCTTCTGGAGCAAGATG---
GCCAACAAGGCCGAGCTCATCGGAGCCGATGCTCTTTCAAGGATGCTCGCAGTGTATCCG
CAGACCAAGACTTACTTCAGCCACTGGAAGAGCCTTAGCCCTGGTTCCGCTGAAGTGAAG
AAGCACGGCAAGACCATCATGAATGGCATCGGAGACGCTGTGGGCAAGATGGACGACCTG
GACAGGGGTCTCCTCACTCTGAGCGAGCTGCACGCCTTCAAGCTGAGAGTTGACCCCACC
AACTTCAAGCTTCTCTCAATCAACATCGTGGTGGTTATGGCTATTATGTTCCCTGACGAT
TTCACCCCAATGGCTCATTTGGCCGTCGATAAGTTCCTGTGTGCTTTGGCCCTGGCTCTG
TCCGAGAAGTACCGA
[9] Theragra_chalcogramma_A1
ATG---AGTCTCACACCAAAGGACAAGGCGACCGTCAGGCTCTTCTGGGGCAGGATG---
TCCGGCAAGGCCGAGCTTATCGGCGCCGATGCTCTTTCAAGGATGCTCGCTGTGTACCCG
CAGACCAAGACTTACTTCAGCCACTGGAAGAGCATCAGCCCTGGTTCCTCTGAAGTGAAG
AAGCACGGCAAGACCATCATGATGGGCATCGGAGATGCTGTGAACAAGATGGACGACCTG
GAAAGGGGTCTTCTTACTCTGAGCGAGCTGCACGCCTTCAAGCTGAGAGTTGACCCCACC
AACTTCAAGCTTCTCTCACTCAACATCTTGGTGGTTATGGCTATTATGTTCCCTGATGAT
TTCACCCCAATGGCTCATTTGGCCGTCGACAAGTTCCTGTGTGCTTTGGCCCTGGCTCTG
TCCGAGAAGTACCGA
[10] Gadiculus_argentus_A1
ATG---AGTCTTACACCCAAGGACAAGGCCACCGTCAAGCTCTTCTGGAGCAGGATG---
TCCGCCAAGGCCGAGAACATCGGCTCCGATGCTCTAACAAGGATGCTCGCGGTGTACCCG
CAGACCAAGACTTACTTCAGCCACTGGAAGAGCCTGGCTCCTGGCTCCACTGACGTGAAG
AGGCACGGCAAGGTCATCATGCAGGGCATCGGAGAAGCTGTGGGCAAGATGGACAACCTG
GAGCAGGGTCTGCTCACTCTGAGCGAGCTGCACGCCTTCAAGCTGAGAGTTGACCCCACC
AACTTCAGGCTTCTCTCACTCAACATCGTGATTGTTATGGCTATTATGTTCCCTGAGGAT
TTCACCCCCCAGGCTCATTTGGCCGTGGACAAGTTCCTGTCTGCTCTGGCCCTGGCCCTG
TCCGAGAAGTACCGA
[11] Phycis_phycis_A1
ATGACTAGTCTCAATGATAAAGACAAGGCCACCGTCAAGCTCTTCTGGAAGAAGATG---
TCCACAAAGGCCGAAGTTGTCGGGTCCGATGCTCTGTCAAGGATGCTGGCTGTGTACCCA
CAGACCAAGACCTACTTCAGTCACTGGAAGGACCTGAGCCCTGGCTCTGCTGCGGTCAGG
AAGCACGGCAAGACCGTCATGATGGGCATTGGCGATGCTGTGAGCAAAATGGATGACCTG
AACAACGGTCTGCTCAGTCTGAGCGAGCTGCACGCCTTCAACCTGAGAGTCGACCCCACC
AACTTCAGGCTTCTTTCCGTGAACATCATGGTGGTGATGGCCATCATGTTCCCTGATGAC
TTCACCGAGGTGGCTCATCTGGCCGTCGACAAGTTCCTGGCGGCTGTGGCCCAGGCCCTC
TCCGAGAAGTACCGA
[12] Molva_molva_A1
ATG---AGTCTCACACCAAGGGACAAGGCGACCGTCAAGCTCTTCTGGAGCAAGATG---
GCTTTAAAAGCCGAACTTATCGGCGGCGATGCTCTGTCAAGGATGCTCGCTGTGTTCCCT
CAGACCAAGATTTACTTCAGTCACTGGAAGAACCTGAGCCCTGGCTCCTCTGAAGTGAAG

AAGCACGGCAAGACCATCATGATGGGCATTGGAGACGCTGTGGGCAAAATGGACGACCTT
AACGCAGGTCTTCTCAGTCTTAGCGAGCTGCACGCCTTCAAACTGAGAGTTGACCCCGCC
AACTTCAGGCTTCTCTCACTCAACATCGTGGTGGTTATGGCTATTATGTTCCCTGACGAT
TTCACCCCCATGGCTCATTTGGCCGTGGATAAGTTCCTGTGTGCCTTGGCCCTGGCCCTG
TCCGAGAAGTACCGA
[13] Lota_lota_A1
ATG---AGTCTCTCACCAAAGGACAAGGCGGCCGTCAAGCTTCTCTGGGGCAAGATG---
TCTTCAAAGGCCGAAATTGTCGGCGCCGATGCTCTGTCAAGGNNNNNNNNNNNNNNNNNN
NNNNNNNNNNNNNNNNNNNNNNNNNNNNNNNNNNCTGAGCCCTGGCTCCGCCGAAGTGAAG
AAGCACGGCAAGACCATCATGATGGGCGTTGGAGATGCTGTGGGCAAAATGGACGACCTT
AACGCAGGTNNNNNNNNNNNNNNNNNNNNNNNNNNNNNNNNNNNNNNNNNNNNNNNNNNN
NNNNNNNNNCTGCTCTCAGTCAACATCATGGTGGTTATAGCTATGATGTACCCTGACGAT
TTCACCCCAATGGCTCATTTGGCCATGGATAAGTTCCTGTCTGCTGTGGCCCTGGCCCTG
TCCGAGAAGTACCGA
[14] Brosme_brosme_A1
ATG---AGCCTCACACCAAGAGACAAGGCGACCTGCAAGCTCTTCTGGAGCAAGATG---
GCTTTAAAAGCCGAACATATCGGCGCCGATGCTCTGTCAAGGNNNNNNNNNNNNNNNNNN
NNNNNNNNNNNNNNNNNNNNNNNNNNNNNNNNNNNNNNNNNNNGCTCCGCTGAAGTGAGG
AAGCACGGCAAGACCATTATGATGGGCATTGGAGATGCTGTGGGCAAAATGGATGACCTT
AACGCAGGTCTTCTCAGTCTTAGCGAGCTGCACGCCTTCCAGCTGAGAGTTGACCCCGTC
AACTTCAGGCTTCTCTCACTCAACATCATGGTGGTTATGGCTATCATGTTCCCTGATGAT
TTCACCCCAATGGCTCATTTGGCTGTGGATAAGTTCCTGTGTGCTCTGGCCCTGGCCCTG
TCCGAGAGGTACCGA
[15] Merluccius_merluccius_A1
ATG---AGTCTCACCGCAAAAGACAAGGCGACCGTCCGCCTCTTCTGGAACAAGGTG---
GCCGAGAAGCCCGAGGCCGTCGGCATCGATGCGCTTAACAGGATGCTGGCTGTGTACCCG
CAGACCAAGACGTACTTCAGTCACTGGAAGGACTTGAGCCCTCGCTCCCCTTCCGTGAAG
AAGCACGGAAAGACCCTCATGGTGGGCATCGGAGCCGCCGTGGGCAAAATCGACGACCTT
AACGCGGGTCTCCTCGCCCTCAGCGAGCTGCATGCCTTCCAGCTGAGAGTTGACCCCAAC
AACTTCAGGCTTCTCTTCATCAACCTGTTGGTGGTTCTGGCTATCATGTTCCCAGATGAA
TTTACCCCACAGGCTCATGTGGCCCTGGATAAGTTCCTGTGTGCCTTGGCTTTGGCCCTC
GCCGAGAAGTACCGA
[16] Merluccius_capensis_A1
ATG---AGTCTCACCGCAAAAGACAAGGCGACCGTCCGCCTCTTCTGGAACAAGGTG---
GCCGAGAAGCCCGAGGCCGTCGGCATCGATGCGCTGAACAGGATGCTGGCTGTGTACCCC
CAGACCAAGACGTACTTCAGTCACTGGAAGGACTTGGGCCCTCGCTCCCCTGCCGTGAAG
AAGCACGGAAAGACCCTCATGGTGGGCATCGGAGCCGCCGTGGGCAAAATCGACGACCTT
AACACGGGTCTCCTCGCCCTCAGCGAGCTGCACGCCTTCCAGCTGAGAGTTGACCCCAAC
AACTTCAGGCTTCTCTCCATCAACCTGTTGGTGGTTCTGGCTATCATGTTCCCAGATGAA
TTTACCCCACAGGCTCATGTGGCCCTGGATAAGTTCCTGTGTGCCTTGGCTTTGGCCCTC
GCCGAGAAGTACCGA
[17] Merluccius_polli_A1
ATG---AGTCTCACCGCAAAAGACAAGGCGACCGTCCGCCTCTTCTGGAACAAGGTG---
GCCGAGAAGCCCGAGGCCGTCGGCATCGATGCGCTGAACAGGATGCTGGCTGTGTACCCG
CAGACCAAGACGTACTTCAGTCACTGGAAGGACTTGAGCCCTCGCTCCCCTGCCGTGAAG
AAGCACGGAAAGACCCTCATGTTGGGCATCGGAGCCGCTGTGGGCAAAATGGACGACCTT
AACGCGGGTCTCCTCGCCCTCAGCGAGCTGCACGCCTTCCAGCTGAGAGTTGACCCCAAC
AACTTCAGGCTTCTCTTCATCAACCTGTTGGTGGTTCTGGCTATCATGTTCCCAGATGAA
TTTACCCCACAGGCTCATGTGGCCCTGGATAAGTTCCTGTGTGCCTTGGCTTTGGCCCTC
GCCGAGAAGTACCGA
[18] Melanonus_zugmayeri_A1
ATG---AGTCTCACCGACAAGGACAAGGCGACCGTCAAGCTCTTCTGGGACAAAGTG---
TCTGGAAAGGCCGAGGCTGTCGGCTCCAATGCTCTGTCAAGGATGCTGGCTGTGTACCCA
CAGACCAAGACATACTTCAGTCACTGGAAGGACCTGAGCCCTGGCTCCGTTGCTGTTAGG
AAGCACGGCAAGATCATCCTGATGGGTATTGGAGATGCTGTGTCCAAAATTGACGACCTT
AACGCAAATCTGCTCAGTCTCAGCGAGCTGCACGCCTTCCAGCTGAGAGTTGATCCCACC
AACTTCAGGCTGTTGTCCATCAGCCTCATCGTGGTTCTGGCCATTATGTTCCCAGATGAG

TTCACCCCCCAGGCTCATGTGGCCGTGGATAAGTTCCTCTGCGCCTTGGCTCTGGCGCTC
TCCGAGAAGTACCGA
[19] Macrourus_berglax_A1
ATG---AGTCTCACCGCTAAAGACAGGGCCACCGTCAGGCTCTTCTGGAACAAGGTT---
GCTTCCAATCCCGAGGCTGTCGGAGCCGAAGCTCTGTCCAGGATGCTGGCTGTGTACCCC
CAGACCAAGACCTACTTCAGCCACTGGAAAGACCTTAGCCCTAACTCCGCCCCTGTGAAG
AAGCATGGCAAGACCATCATGACCGGCATTGGAGAGGCTGTGGCCAATATGGACGACCTG
AACGCCAATCTCCTCACTCTCAGTGAGCTGCACGCCTTCCAGCTGCGAGTGGACCCCACC
AACTTCAGGCTTCTCTCCCTGAACCTCATCGTGGTTCTGGCCATCATGTTCCCAGATGAG
TTCACCCCCGTGGCCCACGTGGCTGTGGACAAGTTCCTGTGCGCAGTGGCCCTTGCCCTG
TCCGAGAAGTACCGG
[20] Malacocephalus_occidentalis_A1
ATG---AGTCTCACCGAGACAGACAAGGCGACCGTCCGGCTCTTCTGGAACAAGGTG---
GCTGCCAAATCTGAGGCCGTTGGCAGCGAGGCTCTCTCAAGGATGATGGGCGTGTTCCCC
CAGACCAAGACTTACTTCAGTCACTGGAAGGACCTCAGCCCTAACTCTGCAGTTTTGAAG
AAGCATGGCGGGGTCATCATGGCTGGCATTGGAGCAGCTGTGTCCAAGATGGACGACCTG
AACACAAATCTCCTCACTCTTAGTGAGCTGCATGCCTTCCAGCTGAGAGTTGACCCCACC
AACTTCAGGCTTCTCTCCCTGAACCTCATCGTGGTCCTGGCCATCATGTTCCCAGAAGAG
TTCACACCAGTGGCCCATGTTGCTGTGGATAAGTTCCTGTGTGCTGTGGCCCGTGCTCTT
TCTGAGAAGTACCGA
[21] Bathygadus_melanobranchus_A1
ATG---AGTCTCACCGATAAGGACAAGAGGACCGTCATTCTCTTCTGGAACAAAGTG---
GCTGCAAAGGCCGAAGCTGTCGGCACCGATGCTCTGTCAAGGATGCTGACTGTGTTCCCG
CAGACCAAGACGTACTTTAGTCACTGGAAGGACCTGAGCCCTAGCTCCCCTGCCCTGAGG
AAGCACGGCAGGATCATCATGAAGGGCATTGGAGATGCTGTGGCCAACATGGACGACCTT
AACTCAAATCTTCTCAGTCTCAGCGAGCTGCACGCCTTCCAGCTCAGAGTTGACCCCACC
AACTTCAGGCTTCTCTCCCTCAACCTCATCGTGGTTCTGGCCATTATGTTCCCCAACGAA
TTCACCCCAATGGCTCACGTGGCCGTGGATAAGTTCCTGTGCGCCTTGGCGATGGCCCTC
TCCGAGAAGTACCGA
[22] Muraenolepis_marmoratus_A1
ATG---AGTCTCACCGACAAGGACAAGGCCACCGTCAAGCTCTTCTGGAACAAGGTC---
GCCGGAAAGGCCGAACTCGTCGGCGCCGATGCTCTCTCCAGGATGCTGTCGGTCTACCCC
CAGACCAAGACGTACTTCAGCCACTGGAAGGACCTGAGCCCCGGCTCTGCCCCCGTGAGG
AAGCACGGCAAGACCATCATGATGGGCATCGGAGACGCTGTCACCAAGATGGACGATCTG
AACGCGAACCTCCTCAGCCTCAGCGAGCTGCACGCCTTCCAGCTGAGAGTGGACCCCACC
AACTTCAGGCTGCTCTCCCTCCAGCTGGTGGTGGTTCTGGCCATCATGTTCCCCGTGGAG
TTCACCCCCCAGGCTCACGTGGCCGTGGATAAGTTCCTGTGCGCCCTGGCTCTGGCTCTC
TCCGAGAAGTACCGA
[23] Bregmaceros_cantori_A1
ATG---AGTCTTACCGCCAAAGACCAGGCCGCCGTCAAGAAGATCTGGGACAAGATC---
GCCACCAAGACCGAGCAGGTCGGAGCCGATACCCTGTTCAGGATGCTGCAGGTGTACCCC
CAGACCAAGACCTACTTCGGCCAGTGGAAGGACATCACCAGTGATAACGCCATGCTGAGG
AAGCACGGCAAGAAGATCCTGATGGGCATCGGCGAGGCCACCACCAAGATGAACGATCTG
GAGAACCAGCTTCTTACCCTCAGCGAGCTCCACGCCTTCCAGCTGCGAGTGGACCCCGCC
AACTTCAAGCTGCTCAGCTCCTGCCTGATGGTTGTGCTGGCCATCATGTTCCCCGATGAC
TTCACCCCCCAGGCCCATGTCGCCCTCGACAAGTTCATGGCCGCCCTGGCCATGGCTCTG
GCCGAGAAGTACCGA
[24] Mora_moro_A1
ATG---AGTCTCACCGCAAAAGACAAGGTCACCGTCCGGATGTTCTGGAACAGGGTG---
GCCAACAAGGCTGAAGCTGTCGGCGCCGATGCTCTGTCCAGGATGCTGTCTGTGTTCCCA
CAGACCAAGACGTACTTCAGTCACTGGAAGGACCTGAGCCCTGGCTCCGCTCCCGTGAGG
AAGCACGGCAAGATCATCATGATGGGTATTGGAGATGCTGTGAGCAAAATGGACGACCTT
AACGCAAACCTTCTGGGTCTCAGTGAGCTGCATGCCTTCCAGCTGAGAGTTGACCCCACC
AACTTCAGGCTTCTCTCCCTGAACATCATAGTGGTTCTGGCTATTATGTTCCCAGATGAG
TTCACTCCAATGGCTCATGTGGCCGTGGATAAGTTCCTGTGTGCCTTGGCTCGGGCCCTC
TCAGAGAAGTACCGA
[25] Laemonema_laureysi_A1

ATG---AGTCTCTCTGCAAAGGACAAGGCGACCGTCCGGATGTTCTGGAACAAGGTG---
GCCAACAAGGCCGAAGCTGTCGGCGCCGATGCACTGTCCAGGNNNNNNNNNNNNNNNCCCA
CAGACCAAGACGTACTTCAGTCACTGGAAGGATCTGAGCCCTGGCTCTTCTCCCGTGAGG
AAGCACGGCAAGATCATCATGATGGGAATTGGAGATGCTGTGAAGAAAATGGATGACCTT
GACTCAAATCTTCTGAGTCTTAGTGAGCTGCATGCCTTCCAGCTGAGAGTTGACCCCGCC
AACTTCAGGCTTCTCTCCCTGAACCTCATAGTGGTTCTGGCTATTATGTTCCCAGATGAG
TTCACCCCAGTGGCTCATGTGGCCGTGGATAAGTTCCTGTGTGCCGTGGCTCGGGCCCTC
TCAGAAAAGTACCGA
[26] Percopsis_transmontana_A2
ATG---AGTCTCTCAGCCAAAGACAAAGCCGCCGTCAAAGCCTTCTTTGACAAAGTG---
GGCGCTAAAGGGGACGAAATTGGACACGGTGCACTTGCACGGACCATATTCGTGTATCCG
CAGACCAAGACTTACTTCGCTCACTGGAAGGATCTGAGCCCCACTTCTCCCAACATAAAG
AAGCATGGAGCCACCATCATGCGTGGGGTGCTGGACGCAGTAGCTGCGATAGATGACATG
ACCGCAGGACTTCTCACCCTCAGTGAACTGCATGCCTTCCGCCTGCGTATTGATCCGGCC
AACTTCAAGCTCATCAGTCACAACCTGCTGGTGGTCATGGCCCTCCTGTTCCCGGATGAC
TTTACCCCCCAGCTGCACGTTGCCATGGACAAGTTCCTGGCCAAACTGGCTCTGGCACTG
GCTGAGAAGTACCGT
[27] Thrachyrincus_scabrus_A1
ATG---AGTCTCACCGAGAAAGACAAGGCGACCGTCAAGCTCTTCTGGAACAAGGTG---
GCCGGAAAGGCCGAGCTTGTCGGCTCCGACGCGCTGTCAAGGATGCTGCACGTGTTCCCG
CAGACCAAGACGTACTTCAGCCACTGGAAGGACTTGAGCCCCGGCTCCTCTCCCGTGAGG
AAGCACGGCAAGACAATCATGATGGGCATCGGAGAGGCTGTGAAGAGAATTGACGACCTT
GACGCAGACCTCCTCACTCTCAGCGAGCTGCACGCCTTCCAGCTGAGAGTGGACCCCACC
AACTTCAGGCTTCTCTCCCTCAACCTCATCGTGGTTCTGGCTATTATGTTCCCTGATGAA
TTCACCCCGATGGCTCATGTGGCCGTGGATAAGTTCCTGTGCGCCGTGGCTCTGGCCCTT
TCCGAGAAGTACCGA
[28] Arctogadus_glacilis_A2
ATG---AGTCTCTCAGCTAAGCAAAAGACCACAGTCAAGGAATTCTTCGGCAAGATG---
TCCACCCGGTCCGATGATATCGGAGCTGAGGCTCTGTCCAGGTTGGTGGCTGTGTACCCC
CAGACCAAGTCCTATTTCTCTCACTGGAAGGACGCGAGCCCCGGCTCTGCCCCCGTCAGG
AAGCATGGCATCACCATCATGGGTGGAGTGTACGATGCCGTCACCAAGATCGACGACCTG
AAGGGAGGACTCCTCAGCCTTAGCGAGCTGCACGCGTTCATGCTGAGAGTGGACCCCGTC
AACTTCAAGCTCCTGGCTCACTGCATGCTGGTGTGCATGTCCATGGTCTTCCCCGAGGAG
TTCACCCCTCAGGTCCATGTGGCCGTCGACAAATTCCTGGCCCAGCTGGCCCTGGCTCTT
GCCGAGAAGTACCGT
[29] Boreogadus_saida_A2
ATG---AGTCTGTCAGCTAAGCAAAAGGCCACAGTCAAGGATTTCTTCGGCAAGATG---
TCCACCCGGTCCGATGATATCGGAGCTGAGGCTCTGTCCAGGTTGGTGGCTGTGTACCCC
CAGACCAAGTCCTATTTCGCTCACTGGAAGAGCGCGAGCCCCGGCTCTGCCCCCGTCAGG
AAGCATGGCATCACCATCATGGGTGGAGTGTACGATGCCGTCGGCAAGATCGACGACCTG
AAGGCAGGACTCCTCAGCCTTAGCGAGCTGCACGCGTTCATGCTGAGAGTGGACCCCGTC
AACTTCAAGCTCCTGGCTCACTGCATGCTGGTGTGCATGTCCATGGTCTTCCCCGAGGAG
TTCACCCCTCAGGTCCATGTGGCCGTCGACAAATTCCTGGCCCAGCTGGCCCTGGCTCTT
TGCGAGAAGTACCGC
[30] Trisopterus_minutus_A2
ATG---AGTCTTACAGGCAAGCAGAAGGCCACAGTCAAGGATTTCTTCGCCAAGATT---
TCCACCCGGTCCGAGGATATCGGAGCTGAGGCTCTGTCCAGGTTGGTGGCTGTGTACCCC
CAGACCAAGTCCTATTTCTCTCACTGGAAGGACACGAGCCCCGGCTCTGCCCCCGTCAGG
AAGCATGGCATCACCATCATGGGTGGAGTGTACGATGCCGTCGGCAAGATCGACGACATG
AAGACAGGACTCCTCAGCCTCAGCGAGCTGCACGCGTTCATGCTGCGAGTGGACCCCGTC
AACTTCAAGCTCCTGGCTCACTGCATGCTGGTGTGCATGGCCATGATCTTCCCCGATGAG
TTCACACCTCAGGTCCATGTGGCCGTCGACAAATTCCTGGCCCAGCTGGCCCTGGCTCTT
GCCGAGAAGTACCGT
[31] Pollachius_virens_A2
ATG---AGTCTGACATCTAAGCAAAAGGCCACAGTCAGGGACTTCTTCGGCAAGGTG---
TCCAGCCGGTCCGAGGATGTCGGAGCTGAGGCTCTGTCCAGGTTGGTGGCTGTGTACCCC
CAGACCAAGTCCTATTTCTCTCACTGGAAGGACGTGAGCCCCGGCTCTGCCCCCGTCAGG

AAGCATGGCATCACCATCATGGGTGGAGTGTACGATGCCGTCGGCAAGATCGACGACCTG
AAGGGAGGACTCCTCAGCCTTAGCGAGCTGCACGCGTTCATGCTGAGAGTGGACCCCGTC
AACTTCAAGCTCCTGGCTCACTGCATGCTGGTGTGCATGGCCATGATCTTCCCCGATGAG
TTCACCCCTCAGGTCCACGTGGCCGTCGACAAATTCCTGGCCCAGCTGGCCCTGGCTCTG
GCCGAGAAGTACCGT
[32] Melanogrammus_aeglefinus_A2
ATG---AGTCTGACACCTAAGCAAAAGGCCACAGTCAAGGACTTCTTCGCCAAGGTG---
GCCAGCCGGTCCGAGGATATCGGAGCTGAGGCTCTGTCCAGGTTGGTGGCTGTGTACCCC
CAGACCAAGTCCTATTTCTCTCACTGGAAGGACCAGAGCCCCGGCTCTGCCCCCGTCAGG
AAGCATGGCATCACTATCATGGGTGGAGTGTACGATGCCGTCGGCAAGATCGACGACCTG
AAGGGAGGACTCCTCAGCCTTAGCGAGCTGCACGCGTTCATGCTGAGAGTGGACCCCGTC
AACTTCAAGCTTCTGGCTCACTGCATGCTGGTGTGCATGGCCATGGTCTTCCCCGATGAG
TTCACCCCTCAGGTCCATGTGGCCGTCGACAAATTCCTGGCCCAGCTGGCCCTGGCTCTT
GCCGAGAAGTACCGA
[33] Merlangius_merlangus_A2
ATG---AGTCTGACACCTAAGCAAAAGGCCACAGTCAAGGACTTCTTCGCCAAGGTG---
TCCAGCCGGTCCGAGGATATCGGAGCTGAGGCTCTGTCCAGGTTGGTGGCTGTGTACCCC
CAGACCAAGTCCTATTTCTCTCACTGGAAGGACGTGAGCCCCGGCTCTGCCCCCGTCAGG
AAGCATGGCATCACCATCATGGGTGGAGTGTACGATGCCGTCGGCAAGATCGACGACCTG
AAGGGAGGACTCCTCAGCCTTAGCGAGCTGCACGCGTTCATGCTGAGAGTGGACCCCGTC
AACTTCAAGCTTCTGGCTCACTGCATGCTGGTGTGCATGGCCATGATCTTCCCCGATGAG
TTCACCCCTCAGGTCCATGTGGCCGTCGACAAATTCTTGGCCCAGCTGGCCCTGGCTCTT
GCCGAGAAGTACCGT
[34] Theragra_chalcogramma_A2
ATG---AGTCTCTCAGCTAAGCAAAAGGCCACAGTCAAGGATTTCTTCAGCAAGATG---
TCCACCCGGTCCGATGATATCGGAGCTGAGGCTCTGTCCAGGTTGGTGGCTGTGTACCCC
CAGACCAAGTCCTATTTCTCTCACTGGAAGGACGCGAGCCCCGGCTCTGCCCCCGTCAGG
AAGCATGGCATCACCATCATGGGTGGAGTGTACGATGCCGTCGGCAAGATCGACGACCTG
AAGGGAGGACTCCTCAGCCTTAGCGAGCTGCACGCGTTCATGCTGAGAGTGGACCCCGTC
AACTTCAAGCTCCTGGCTCACTGCATGCTGGTGTGCATGTCCATGATCTTCCCCGAGGAG
TTCACCCCTCAGGTCCATGTGGCCGTCGACAAATTCCTGGCCCAGCTGGCCCTGGCTCTT
GCCGAGAAGTACCGT
[35] Gadiculus_argentus_A2
ATG---AGTCTCACCGCTAAGCAGAAGGCCACAGTCAGGGAGTTCTTCGGCAAGATG---
TCCGCACGGTCCGAGGACATCGGAGCTGAGGCTCTGTCCAGGTTGGTGGCTGTGTACCCC
CAGACCAAGTCCTATTTCTCTCACTGGAAGGACACGAGCCCCGGCTCTGCCCCCGTCAGG
AAGCACGGCATCACCATCATGGGTGGAGTGTACGACGCCGTCGGCAAGGTCGACGACCTG
AAGGGAGGACTCCTCAGCCTCAGCGAGCTGCACGCGTTCATGTTGAGAGTGGACCCCGTC
AACTTCAAGCTTCTGGCTCACTGCATGCTGGTGTGCATGGCCATGGTGTTCCCAGATGAG
TTCACCCCTCAGGTCCATGTGGCCGTCGACAAATTCCTGGCCCAGCTGGCCCTGGCTCTT
GCCGAGAAGTACCGT
[36] Phycis_phycis_A2
ATG---AGTCTGACCTCCAAACAAAAGGCCACAGTGAAGGACTTCTTCGCCAAGGTG---
TCCAGCAGATCCGAGGACATCGGAGCCGAAGCTCTGTCCAGGCTGGTCGCTGTGTACCCA
CAGACCAAGTCCTACTTCGCCCACTGGAAGGACGTGAGCCCCGGGTCTGGCCCCGTCAGG
AAACATGGCATCACCATCATGGGTGGAGTGTACGATGCCGTCGGCAAGATCGACGACCTG
AAGGGAGGACTCCTCAGCCTGAGCGAGCTGCACGCTTTCATGCTGAGGGTTGACCCCGTC
AACTTCAAGCTCCTGGCCCACTGCATGCTGGTCTGCATGTCCATGCTCTTCCCCGAGGAG
TTCACCCCCCAGGTCCACGTGGCCGTCGACAAATTCCTGGCCCAGCTTGCCCTGGCTCTG
GCCGAGAAGTACCGT
[37] Molva_molva_A2
ATG---AGTCTGACCTCTAAGCAAAAGGCCACAGTCAAGGACTTCTTCAGCAAGGTG---
TCCAGCAGATCCGACGATATCGGAGCTGAAGCCCTTTCCAGGTTGGTCGCTGTGTACCCA
CAGACCAAGTCCTACTTCTCCCACTGGAAGGATGTGAGCCCCGGCTCTGGCCCCGTCAGG
AAACATGGCATCACCATCATGAATGGAGTGTACGATGCCGTCACCAAGATCGACGACTTG
AAGGGAGGACTCCTCAGCCTCAGCGAGCTGCACGCATTCATGCTGAGGGTTGACCCCGTC
AACTTCAAGCTCCTGGCCCACTGCATGCTGGTGTGCATGTCCATGCTCTTCCCCGAAGAA

TTCACCCCTCAGGTCCACGTGGCCGTCGACAAATTCCTGGCCCAGCTGGCCCTGGCTCTT
GCCGAGAAGTACCGT
[38] Lota_lota_A2
ATG---AGTCTGTCCCCTAAGCAAAAGGCCACAGTCAAGGAGTTCTTCGGCAAGGTC---
TCCAGCAGATCCGAGGATATCGGAGCCGAATCTCTGTCCAGGTTGGTCGCTGTGTACCCA
CAGACCAAGTCCTACTTCTCCCACTGGAAGGATGTGAGCCCCGGCTCTGCCCCCGTCAGG
AAACATGGCATCACCATCATGGGTGGAGTGTACGATGCCGTCTCCAAGATCGACGACATG
AAGGGAGGACTCCTCAGCCTCAGCGAGCTGCACGCATTCATGCTGAGGGTTGACCCCGTC
AACTTCAAGCTCCTCGCCCACTGCATGCTGGTGTCCATGTCCATGCTCTTCCCCGATGAT
TTCACCCCTCAAGTCCACGTGGCCGTCGACAAATTCCTGGCCCAGCTGGCCCTGGCTCTT
TCCGAGAAGTACCGT
[39] Brosme_brosme_A2
ATG---AGTCTCACCTTTAAGCAAAAGGCCACAGTCAAGGACTTCTTCAGCAAGGTG---
TCCAGCAGATCCGAGGATATCGGAGCTGATTCTCTTTCCAGGTTGGTCGCTGTGTACCCA
CAGACCAAGTCCTACTTCTCTCACTGGAAGGAGACGAGCCCCGGCTCTGCCCCCGTCAGG
AAACATGGCATCACCATCATGGGTGGAGTGTACGATGCCGTCACCAAGATGGACGACTTG
AAGGGAGGACTCCTCAGCCTCAGCGAGCTGCACGCATTCATGTTGAGGGTTGACCCCGTC
AACTTCAAGCTCCTGGCCCACTGCATGCTGGTGTCCATGTCCATGATGTTCCCCGACGAA
TTCACCCCTCAGGTCCACGTGGCCGTCGACAAATTCCTGGCCCAGCTGGCCCTGGCTCTT
GCCGAGAAGTACCGT
[40] Merluccius_merluccius_A2
ATG---AGTCTCACCGTTAAGCAAAAGGCCACCGTCAAGGAGTTCCTCAACAAGGTC---
ACCAGCAGACACGAGGATATCGGAGCTGAATCCCTCTCCAGGCTGGTCGCTGTGTACCCT
CAGACCAAGTCCTACTTCGCCCACTGGACCTCCACGGACCCCGGCTCCGCACCCGTCAGG
AAACATGGTATCACCATCATGAACGGCGTGTACGACGCCATCTCCAAGATGGACGACCTG
AAGGGAGGACTCCTCAGCCTCAGCGAGCTGCACGCATTCATGCTGAGGGTTGACCCCGTC
AACTTCAAGCTCCTGGCCCACTGCATGCTGGTGAGCTTCGCAATGTTCTACCCCGATGAC
TTCACCCCTCAGGTCCACGTGGCCATTGACAAATTCCTGGCCCAGCTGGCCCTGGCCCTG
TGCGAGAAGTACCGT
[41] Merluccius_capensis_A2
ATG---AGTCTCACCGTTAAGCAAAAGGCCACCGTCAAGGAGTTCCTCAGCAAGGTC---
ACCAGCAGACACGAGGATATCGGAGCTGAAGCCCTCTCCAGGATGGTCGCTGTGTACCCT
CAGACCAAGTCCTACTTCGCCCACTGGGCCTCCACGGACCCCGGCTCCGCACCCGTCAGG
AAACATGGTGTCACCATCATGAACGGCGTGTACGACGCCATCACCAAGATGGACGACCTG
AAGGGAGGACTCCTCAGCCTCAGCGAGCTGCACGCATTCATGCTGAGGGTTGACCCCGTC
AACTTCAAGCTCCTGGCCCACTGCATGCTGGTGAGCTTCGCAATGTTCTACCCCGATGAC
TTCACCCCTCAGGTCCACGTGGCCATTGACAAATTCCTGGCCCAGGTGGCCCTGGCCCTG
TGCGAGAAGTACCGT
[42] Merluccius_polli_A2
ATG---AGTCTCACCGTTAAGCAAAAGGCTACCATCAAGGATTTCCTCAGCAAGGTC---
ACCAGCAGACACGAGGATATCGGAGCTGAAGCCCTCTCCAGGATGGTCGCTGTGTACCCC
CAGACCAAGTCCTACTTCGCCCACTGGACCTCCACGGACCCCGGCTCCGCACCCGTCAGG
AAACATGGTGTCACCATCATGAACGGCGTGTACGACGCCATCTCCAAGATAGACGACCTG
AAGGGAGGACTCCTCAGCCTCAGCGAGCTGCACGCATTCATGCTGAGGGTTGACCCCGTC
AACTTCAAGCTCCTGTCCCACTGCATGCTGGTGAGCTTCGCCATGTTCTACCCCGATGAC
TTCACCCCTGTGGTCCACGTGGCCATTGACAAATTCCTGGCCCAGGTGGCCCTGGCCCTG
TGCGAGAAGTACCGT
[43] Melanonus_zugmayeri_A2
ATG---AGTCTGAGCGCTAAGCAAAAGGTCGTGGTCAAGGACTTCTTCAAGTTGATG---
GGCGCAAGATCCGAAGATGTCGGTGCTGACACTCTTTCCAGGCTGGTCGCTGTCTACCCA
CAGACCAAGTCCTACTTCGCCCACTGGAAGTCCGCCGAGCCCGGCTCTGCCCCCGTCAGG
AAACACGGCATTACCATCATGACTGGAGTGATCGATGCCGTCGGCAAGCTGGACGACCTG
AAGGGAGGACTCCTCAGTCTCAGCGAGCTGCACGCATTCATGCTGAGGGTTGACCCCGTC
AACTTCAAGCTCCTGGCCCACTGCATGTTGGTGTGCATGTCCATTTACTTCCCCGATGAT
TTCACCCCTAAGGTCCATATTGCCATGGACAAATTCCTGAACCAGCTGGCCCTGGCCATT
TCCGATAAGTACAGA
[44] Macrourus_berglax_A2

ATG---AGTCTGACATTCAAGCAGAAATCCGCTGTCAAGGACTTCTTTGGACTCATG---
TCTTCCAGGTCCGAGGATATCGGTGCCGATGCTCTTTCCAGGTTGGTGGCTGTGTACCCC
CAGACCAAGTCCTACTTCTCCCACTGGGAGTCCACCGACCCTGGCTCCGCCCCTGTGAGG
AAACATGGCATGACCATCATGACTGGCGTGTACGAAGCCGTCGGCAAGCTGGACAACTTG
ATCGATGGTCTGCTGAGGCTCAGCGAGCTCCACGCCTTCATGCTGAGGGTTGACCCTATC
AACTTCAAGCTGCTGGCCCACTGCATGCTGGTGTGCATGGCCATGTACTACCCTGAGGAG
TTCACCCCCAAGGTCCATGTGGCCGTCGACAAGTTCCTCACCCAGCTGGCCCTGGCTCTG
TCCGACAAGTACCGA
[45] Malacocephalus_occidentalis_A2
ATG---AGTCTGTCAGTCAAGCAGAGAAACCTGGTAAGGGAATTCTTCAAAGTCGTT---
GCCACCAGGTCCGACGATATCGGCACAGAAGCTCTGTCCAGGTTGTTGATTGTGTACCCC
CAGACCAAGTCTTACTTCTCCCACTGGAAGTCCACTGAACCCGGCTCCGCCCCTGTCAGG
AAACACGCCATTATCATCTTTAATGGTGTGTGTGACGCCGTCAGCAAGCTGGACGACCTG
AAGGGTGGTCTTCTGCGCCTCAGTGAGCTCCACGCTTTCGTGCTGAGGGTTGACCCTGTC
AACTTCAAGCTCCTGGCCCACTGCTTGCTGGTGACTATGTCCATGTACTTCCCTGAAGAG
TTCACCCCCAAGGTCCATTTGGCCGTCGACAAGTTCCTCAATCAGTTGGCCGAGGCTCTG
GCCGAGAAGTACCGA
[46] Bathygadus_melanobranchus_A2
ATG---AGTCTGAGCTCTAAGCAAAAGATCTTGGTCAAGGAATTCTTCGGCCTCATG---
GCCACCAGGTCCGAGGATATCGGAGCCGAAGCTCTTTCCAGGTTGGTTTGTGTGTACCCA
CAAACCAAGTCGTACTTCTCCCACTGGGAGTCCACCGACCCCGGCTCTGGCCCGGTCAAG
AAGCACGGCATCACCATCATGGGCGGCGTGTACGACGCGATCGACAAGATCGACAACCTG
AAGGGTGGACTCCTCAGCCTCAGCGAGCTGCACGCATTCATGCTGAGGGTTGACCCCGTC
AACTTCAAGCTGATGGCTCACTGCTTGCTGGTGTGCATCTCCATGTACTACCCCGATGAG
TTTACACCCAAGGTCCATGTGGCCGTCGACAAATTCCTGAACCAGCTCGCCCTGGCTCTG
TCCGACAAGTACCGA
[47] Muraenolepis_marmoratus_A2
ATG---AGTCTCAATTCTAAGCAGAAGGTCATCGTCAGGGACTTCTTCAAAGTGATC---
TCCAGCAGATCCGAAGACATCGGAGCTGATTCTCTCTCCAGGTTGGTGGCTGTGTACCCA
CAGACCAAGTCGTACTTCGCCCACTGGAAGTCCTCCGAACCTGGCTCCGCCCCTGTCAGG
AAACATGGCATCACCATCATGGGAGGAGTGTATGATGCCGTTGGCAAGCTGGACGACCTG
AAGGGTGGGCTCCTCAGCCTCAGCGAGCTGCATGCCTTCATGCTGAGGGTTGACCCTGTC
AACTTCAAGCTGCTGGCCCACTGTATGCTGGTCTGCATGTCCATCTACTTCCCAGATGAG
TTCACCCCTCAGGTCCATGTGGCTGTTGACAAATTCCTGGCCCAGCTGGCCCTGGCTCTG
GCCGACAAGTACCGC
[48] Bregmaceros_cantori_A2
ATGTCGACCCTCAGCCCCAAGCAAAAGGCACTTGTCAAGGAGTTTTTCGGCAAGATC---
GCCGACAGCGCTAACGACGTTGGAGCCGAGGCACTTTGCAGGCTGGTGGCCGTGTACCCA
CAGACCAAGTCCTACTTCTCCCACTGGAAGTCCACCGACCCCGGCTCTGCCCCAGTCATC
AAGCATGGCATCACCATCATGGGAGGCGTGTACGATGCCGTCAACAAGATTGATGACCTG
AAGGGAGGACTGCTGACCCTGAGCGAGCTCCACGCCTTCATGCTGCGCGTTGACCCAGTG
AACTTCAAGCTCCTGGCCCATTGCATGATCTTGTCCCTGGCCATGGCTTTCCCCGCCGAG
TTCACCCCCGAAGTTCACGTGGCCGTGGACAAGTTCCTGGCCCGTCTGGCTCTGGCTCTG
TGCGAGAAGTACCGA
[49] Mora_moro_A2
ATG---AGTCTGACCGCTAAGCAAGCGACCACTGTGAGGGACTTCTTCAAGATCATT---
GCCACCAGATCCGAGGATATTGGAGCCGAGGCACTTTCCAGGATGGTCGCTGTGTACCCA
CAGACCAAGTCCTACTTCTCCCACTGGGAGTCCACCGAGCCCGGCTCCGCCGCCGTGAAG
AAGCACGGCAGCGTCATCGTCGGCGGAGTGTTCGACGCCGTCGGCAAGCTGGACGACCTG
AAGGGAGGCCTGCTCAGCCTCAGCGAGCTGCACGCTTTCATGCTGAGGGTTGACCCCGTC
AACTTCAAGCTCCTGAGTCACTGCTTCCTGGTGTGCATATCCGCCTACTTCCCCGAGGAG
TTCACCCCCAAGGTCCATGTGGCCGTCGACAAATTCCTGGTCCAGGTGGCCCTGGCTCTT
TCCGACAAGTACCGA
[50] Laemonema_laureysi_A2
ATG---AGTCTGAGCGCTAAGCAAGTCACCACTGTGAAGGACTTCTTCAAGATCATT---
TCCACCAGAGCCGAGGATATTGGAGCCGAAGCTCTTTCCAGGATGGTGGCTGTGTACCCA
CAGACCAAGTCCTACTTCTCCCACTGGGAGTCTACCGATCCCGGCTCTCCCGCCGTCAAG

AAGCACGGCAGCGTCATCGTTGGCGGAGTGTTCGATGCCGTCGGCAAGCTGGACGACCTG
AAGGGAGGACTCCTCAGCCTCAGCGAGCTGCACGCATTCATGCTGAGGGTTGACCCCGTC
AATTTCAAGCTCCTGAGTCACTGCCTTCTAGTGTGCATATCCTCATACTACCCCGAAGAG
TTCACCCCCAAGGTCCACGTGGCCGTCGACAAATTCCTGGTCCAGGTGGCCCTGGCTCTT
TCCGACAAGTACCGA
[51] Gadus_morhua_A2
ATG---AGTCTCTCATCTAAGCAGAAGGCCACAGTCAAGGATTTCTTCAGCAAGATG---
TCCACCCGGTCCGATGATATCGGAGCTGAGGCTCTGTCCAGGTTGGTGGCTGTGTACCCC
CAGACCAAGTCCTATTTCTCTCACTGGAAGGACGCGAGCCCCGGCTCTGCCCCCGTCAGG
AAGCATGGCATCACCATCATGGGTGGAGTGTACGATGCCGTCGGCAAGATCGACGACCTG
AAGGGAGGACTCCTCAGCCTTAGCGAGCTGCACGCGTTCATGCTGAGAGTTGACCCCGTC
AACTTCAAGCTCCTGGCTCACTGCATGCTGGTGTGCATGTCCATGATCTTCCCCGAGGAG
TTCACCCCTCAGGTCCATGTGGCCGTCGACAAATTCCTGGCCCAGCTGGCCCTGGCTCTT
GCCGAGAAGTACCGT
[52] Thrachyrincus_scabrus_A2
ATG---AGTCTGAGCTCCAAGCAAAAGATTATAGTCAGGGATTTCTTCAAATTGATC---
GCCGCCAGGTCCGAGGATATCGGAGCCGAGTCTCTTTCCAGGNNNNNNNNNGTGTACCCA
CAGACCAAGTCCTACTTCGCCCACTGGAAGTCTACCGACCCCGGCTCTGCCCCCGTCAGG
AAACATGGCATCACCATCATGGGTGGAGTGTACAAAGCTATCGAAAACCTCGACGACATG
AAGGGAGGACTCCTCGGCCTCAGCGAGCTGCACGCATTCATGCTGAGGGTTGACCCAGTC
AACTTCAAGCTCCTGGGACACTGTTTGCTGGTGTGCATGTCCGCGTACTTCCCCGATGAC
TTCACCCCCCAGGTCCACGTGGCCGTCGACAAATTCCTGGCCCGGCTGGCCCTGGCTCTC
TCCGACAAGTACCGT
[53] Percopsis_transmontana_A3
ATG------CTCTCAAAAAAGGAGAAAGACTTGATTAGAGAAATATGGGAGAGCTGT---
ACACCTGAGGCCGAGGCCATTGGATCAGACGCCCTTCTGAGGATGTTTACAACTGTTCCT
GGCAGCAAGACGTATTTCTCCCACTTG---GACATCAGTCCGCGCTCGCCTCACCTGTTG
AGGCATGGCAAGAAGATTATCATGGCCATAGCCGAAGGAGCCAAGGATATCACTCAGCTC
ACTGAAACCTTAGCACCTCTCCAAACTCTGCACGCCTACACGCTCCGGATAGACCCAACG
AACTTCAAGGTCTTCTCACACTGTCTGCTTGTCAGTCTGGGCTGTTTCATGGGTGACAGC
TTCACGGCGGTGGCACATGCGGCCATGGACAAGTTCCTCTCGGCGTTCGCCGCGGTGCTG
TCGGAGAAATACAGA
[54] Arctogadus_glacilis_A3
ATG------CTCTCAAAACGGGAGAAAGAGCTTATCATAGAAATATGGACCAGACTG---
ACCCCTGTGGCTGACCGCATAGGAGCAGAGGCCCTGCGCAGGATGTTCACGTCATACCCT
GGCACCAAGACGTACTTCTCCCACATG---GACATCACCCCCCGCTCGGCCCACCTGCTG
TCCCTTGGCCAGAAGATCGTCCTGGCGTTGGCGGAGGGCTCCAAGGACATTGCTAACCTG
ATGACTAGCCTCGCTCCTCTTCAAACCTACCACGCCTACCAGCTCCGCATCCAGCCCAAC
AACTTCAAGCTGTTTTCGCACTGTATGATAGTCACCCTTGCCTGTTTCATGGGTGACCGC
TTCACGCCCAGTTCGCATGCTGCCATGGACAAGTATCTCTCTGCGTTCTCTGCCGTACTT
GGGGAGAAATTCAGA
[55] Gadus_morhua_A3
ATG------CTCTCAAAACAGGAGAAAGAGCTTATCATAGAAATATGGACCAGACTG---
ACCCCTTTGGCTGACCGCATAGGAGCAGAGGCTCTTCTGAGGATGTTCACATCATACCCT
GGCACCAAGACGTACTTCTCCCACCTG---GACATCACCCCCCGCTCGGCCCACCTGCTG
TCCCATGGCCAGAAGATCTTCCTGGCGTTGGCAGAGGGCTCCAAGGACATTGCTAACCTG
ATGACTAACCTCGCTCCTCTTCAAACCTACCACGCCTACCAGCTCCGCATCCAGCCCAAC
AACTTCAAGCTGTTTTCGCACTGTATGATAGTCACCCTTGCCTGTTTCATGGGTGACCGC
TTCACGCCCAGTTCGCATGCCGCCATGGACAAGTATCTCTCTGCGTTCTCTGCCGTACCT
GGGGAGAAATTCAGA
[56] Boreogadus_saida_A3
ATG------CTCTCAAAACAGGAGAAAGAGCTTATCATAGAAATATGGACCAGACTG---
ACCCCTGTGGCTGACCGCATAGGAGCAGAGGCTCTTCTGAGGATGTTCACGTCATACCCT
GGCACCAAGACGTACTTCTCCCACCTG---GACATCACCCCCCGCTCGGCCCACCTGCTG
TCCCATGGCCAGAAGATCTTCCTGGCGTTGGCGGAGGGCTCCAAGGACATTGCTAACCTG
ATGACTACCCTCGCTCCTCTTCAAACCTACCACGCCTACCAGCTCCGCATCCAGCCCAGC
AACTTCAAGCTGTTTTCGCACTGTATGATAGTCACCCTTGCCTGTTTCATGGGTGACCGC

TTCACGCCCAGTTTGCATGCCGCCATGGACAAGTATCTCTCTGCGTTCTCTGCCGTACTC
GGGGAGAAATTCAGA
[57] Trisopterus_minutus_A3
ATG------CTCTCAGAACAGGAGAAAGAGCTAATCGTAGAAATATGGACCAGACTG---
ACCCCTGTCGCTGACCGCATAGGAGCAGAGGCTCTTCTGAGGATGTTTAAGTCATACCCT
GGCACCAAGACGTACTTCTCCCACCTG---GACATCACCCCCCGCTCTGCCCACATGCTG
TCCCATGGCCAGAAGATCTTCCTGGCTTTGGCGGAGGGCTCCAAGGACATTGCTAACCTG
ATGACTAACCTCGCTCCTCTTCAAACCTATCATGCCTACCAGCTTCGCATCCAGCCCAAC
AACTTCAAGCTGTTTTCGCACTGTATGATAGTCACCCTGGCCTGTTTCATGGGCGACCGC
TTCACGCCCAGTTCGCATGCCGCCATGGACAAGTATCTCTCGGCGTTCTCTGCCGTACTT
GGGGAGAAATTCAGA
[58] Pollachius_virens_A3
ATG------CTCTCAAAACAGGAGAAAGAGCTTATCATAGAGATATGGACCAGACTG---
ACCCCTGTGGCTGACCGTATAGGAGCAGAGGCTCTTCTGAGGATGTTCACGTCATACCCT
GGCACCAAGACGTACTTCTCCCACCTG---GACATCACCCCCCGCTCGGCCCACCTGCTG
TCCCATGGCCAGAAGATCGTCCTGGCGTTGGCGGAGGGCTCCAAGGACATTGCTAACCTG
ATGACTAACCTCGCTCCTCTTCAAATCTACCACGCCTACCAGCTCCGCATCCAGCCCAAC
AACTTCAAGTTGTTTTCGCACTGTATGATAGTCACCCTCGCCTGTTTCATGGGCGACCGC
TTCACGCCCAGTTCGCATGCCGCCATGGACAAGTATCTCTCTGCGTTCTCTGCCGTACTT
GGTGAGAAATTCAGA
[59] Melanogrammus_aeglefinus_A3
ATG------CTCTCAAAACAGGAGAAAGAGCTTATCATAGAAATATGGACCAGACTG---
ACCCCTGTGGCAGACCGTATAGGAGCAGAGGCTCTTCTGAGGATGTTCACGTCGTACCCC
GGCACCAAGACGTACTTCTCCCACCTG---GACATCACCCCCCGCTCGGCCCACCTGCTG
TCCCATGGCCAGAAGATCGTCCTGGCGTTGGCGGAGGGCTCCAAGGACATTGCTAACCTG
ATGACTAACCTCGCTCCTCTTCAAACCTACCACGCCTACCAGCTCCGCATTCAGCCCAAC
AACTTCAAGCTGTTTTCACACTGTATGATAGTCACCCTCGCCTGTTTCATGGGCGACCGC
TTCACGCCCAGTGCGCATGCCGCCATGGACAAGTATCTCTCTGCCTTCTCTGCCGTACTT
GGGGAGAAATTCAGA
[60] Merlangius_merlangus_A3
ATG------CTCTCAAAACAGGAGAAAGAGCTCATCATAGAAATATGGACCAGACTG---
ACCCCTGTGGCTGACCGCATAGGAGCAGAGGCTCTTCTGAGGATGTTCACGTCGTACCCT
GGCACCAAGACGTACTTCTCCCACCTG---GACATCACCCCCCGCTCTGCCCACCTGCTG
TCCCATGGCCAGAAGATCGTCCTGGCGTTGGCAGAGGGCTCCAAGGACATTGCTAACCTG
ATGACTAACCTCGCTCCTCTTCAAACCTACCACGCCTACCAGCTCCGCATCCAGCCCAAC
AACTTCAAGCTGTTTTCGCACTGTATGTTAGTCACGCTCGCCTGTTTCATGGGCGACCGC
TTCACGCCCAGCTCTCACGCCGCCATGGACAAGTACCTTTCTGCCTTCTCTGCCGTACTT
GGGGAGAAATTCAGA
[61] Theragra_chalcogramma_A3
ATG------CTCTCAAAACAGGAGAAAGAGCTTATCATAGAAATATGGACCAGACTG---
ACCCCTGTGGCTGACCGCATAGGAGCAGAGGCTCTTCTGAGGATGTTCACGTCATACCCT
GGCACCAAGACGTACTTCTCCCACCTG---GACATCACCCCCCGCTCGGCCCACCTGCTG
TCCCTTGGCCAGAAGATCTTCCTGGCGTTGGCGGAGGGCTCCAAGGACATTGCTAACCTG
ATGACTAACCTCGCTCCTCTTCAAACCTACCACGCCTACCAGCTCCGCATCCAGCCAGAC
AACTTCAAGCTGTTTTCGCACTGTATGATAGTCACCCTTGCCTGTTTCATGGGTGACCGC
TTCACGCCCAGTTCGCATGCCGCCATGGACAAGTATCTCTCTGCGTTCTCTGCCGTACTT
GGGGAGAAATTCAGA
[62] Gadiculus_argentus_A3
ATG------CTCTCAGAAAAGGAGAAAGAGCTAATCGTAGAAATATGGACCAGACTG---
ACCCCTGTCGCTGACCGCATCGGAGCAGAGGCTCTTCTGAGGATGTTCAGGTCATACCCT
GGCACCAAGACGTACTTCTCCCACCTG---GACATCACCCCCCGCTCGCCCCACATGCTG
TCCCATGGCCAGAAGATCTTCCAGGCGTTGGCGGAGGGCTCCAAGGACATTGCTAACCTG
ATGACTAACCTCGCTCCTCTTCAAACCTATCACGCCTACCAGCTCCGCATCCAGCCTGAC
AACTTCAAGCTGTTGTCTTACTGTATGATAGTCACCATCGCCTGTTTCATGGGCGACCGC
TTTACGCCCAGTTCGCACGCCGCCATGGACAAGTATCTCTCTGCGTTCTCTGCCGTACTT
GGGGAGAAATTCAGA
[63] Phycis_phycis_A3

ATG------CTCTCGAAACAGGAGAAAGAGCTAATTGAGGAGATATGGACGGGCCTG---
AGCCCTGTCGCCGACACCATTGGAGCAGAGGCTCTGCTGAGGATGTTCACGTCCTACCCC
GGCACCAAGACGTACTTCGCCCACCTG---GACATCAGACCGCGCTCCCGCCACCTGCTG
TCCCACGGCGAGAAGATCGTCATGGCGTTGGCAGAAGGTTCCAAGGACATCAGCAACCTG
ATGACCAACCTGGCTGCTCTTCAGACCTACCATGCCTACCAGCTCCGCATCCATCCCACC
AACTTCAAGCTGTTTTCGCACTGTATGCTGGTCACCCTAGCCTGCTTCATGAGAGACGAC
TTCACGCCCATTGTGCACGCAGCCATGGACAAGTACCTCTCCGCGTTCTCCGCCGTGCTG
TCTGAGAAGTTCCGA
[64] Molva_molva_A3
ATG------CTCTCCAAACAGGAGAAAGAGCTAATCGTAGAAATATGGACCAGACTG---
ACCCCTGTCGCTGACACCATAGGAGCAGAGGCTCTTCTGAGGATGTTCACGTCATACCCT
GGCACCAAGACGTACTTCGCCCACCTA---GACATCACCCCCCGTTCTCCCCACCTGCTG
TCCCATGGCCAGAAGATCTTCATGGCGTTGGCGGAGGGTTCCAAGGACATTAGTAACCTG
ATGACTACCTTAGCTCCTCTTCAAACCTACCACGCCTATCAGCTCCGCATTCACCCCACC
AACTTCAAGCTGTTTTCGCACTGTATGATAGTAACCCTCGCTTGTTTCATGGGCCAACAC
TTCACGCCCAGTTCGCACGCAGCCATGGACAAGTACCTCTCAGCCTTCTCTGCCGTACTT
GCGGAGAAATTCAGA
[65] Lota_lota_A3
ATG------CTCTCAAAACAGGAGAAAGAGCTCATCGTAGAAATATGGACCAGACTG---
ACCCCTGTCGCTGACACCATAGGAGCAGAGGCTCTTCTGAGGATGTTCACGTCATACCCT
GGAACCAAGACGTACTTCGCCCACCTA---GATATCACCCCCCGTTCTGCCCACCTGCTG
TCCCATGGCCGTAAGATCGTCATGGCTTTGGCAGAGGGTTCCAAGGACATTAGTAACCTG
ATGACTAATTTAGCTCCTCTTCAAACCTACCACGCCTATCAGCTCCGCATTCACCCCACC
AACTTCAAGCTTTTTTCGCACTGTATGGTAGTCACCCTCGCCTGTTTCATGGGCGAACGC
TTCACGCCCAGTTCGCATGCAGCCATGGACAAGTATCTCTCTGCGTTCTCTGCCGTACTT
GCAGAGAAATTCAGA
[66] Brosme_brosme_A3
ATG------CTCTCAAAACAGGAGAAAGAGCTAATCGTAGAACTATGGACCAGACTG---
GCCCCTGTCGCTGACACCATAGGAGCGGAGGCTCTTCTGAGGATGTTCACTGCATACCCT
GGCACCAAGACGTACTTCTCCCACCTA---GACATCACCGCCCGTTCTCCCCACCTGCTG
TCCCATGGCCGGAAGATCGTCATGGCGTTGGCGGAGGGTTCCAAGGACATCGGTAACCTG
ATGACTAACCTAGCTCCTCTTCAGACCTACCATGCCTATCAGCTCCGCATTCACCCCACC
AACTTCAAGCTGTTTTCGCACTGTATGCTAGTCACCCTCGCCTGTTTCATGGGCGACCGC
TTCACGCCCAGTTCGCACGCAGCCATGGACAAGTACCTCTCGGCGTTCTCTGCCGTACTT
GCGGAGAAATTCAGA
[67] Merluccius_merluccius_A3
ATG------CTCTCGAAACAGGAGAAAGAGCTCATCGTCGAAATATGGACCGGGCTG---
ACCCCTGTCGCTGACACCATAGGAGCAGAAGCTCTTCTCAGGATGCTCACGTCGTTCCCG
GGCACCAAGACGTACTTCGCCCACCTG---GACATCACGCCGCGCTCCGCCTACCTGCTG
GCCCACGGCCGGAAGATCGTCCTGGCGCTGGCGGACGCCTCCAAGGACATGGGAAACCTG
ATGACCAATTTAGGTCCTCTTCAAACCTACCATGCCTATCAGCTCCGCATTCACCCCACC
AACTTCAAGCTGTTGTCGTACTGTATGCTGGTGACCCTCGCCTGTTACATGGGCCAGAAG
TTCACACCAGTCTCCCACGCAGCCATGGACAAGTACCTGTCAGCATTCTCCGCTGTGCTT
GGGGAGAAATTCAGA
[68] Merluccius_capensis_A3
ATG------CTCTCGAAACAGGAGAAAGAGCTCATCGTCGAAATATGGACCGGGCTG---
ACCCCTGTCGCTGACACCATAGGAGCAGAAGCTCTTCTCAGGATGCTCACGTCGTTCCCG
GGCACCAAGACGTACTTCGCCCACCTG---GACATCACGCCGCGCTCCGCCTACCTGCTG
GCCCACGGCCGCAAGATCGTCCTGGCGCTGGCGGACGCCTCCAAGGACATGGGAAACCTG
ATGACCAATTTAGGTCCTCTTCAAACCTACCATGCCTATCAGCTCCGCATTCACCCCACC
AACTTCAAGCTGTTGTCGTACTGTATGCTGGTGACCCTCGCCTGTTACATGGGCCAGAAG
TTCACACCAGTCTCCCACGCAGCCATGGACAAGTACCTGTCAGCATTCTCCGCTGTGCTT
GGGGAGAAATTCAGA
[69] Merluccius_polli_A3
ATG------CTCTCGAAAAAGGAGAAAGAGCTCATTGTCGAAATATGGACCGGGCTG---
ACCCCTGTCGCTGACACCATAGGAGCAGAAGCTCTTCTCAGGATGCTCACGTCGTTCCCG
GGCACCAAGACGTACTTCGCCCACCTG---GACATCACGCCGCGCTCCGCCTACCTGCTG

GCCCACGGCCGGAAGATCGTCCTGGCGCTGGCGGACGCCTCCAAGGACATGGGAAACCTG
ATGACCAATTTAGGTCCTCTTCAAACCTACCATGCCTATCAGCTCCGCATTCACCCCACC
AACTTCAAGCTGTTGTCGTACTGTATGCTGGTGACCCTCGCCTGTTACATGGGCCAGAAG
TTCACACCAGTCTCCCACGCAGCCATGGACAAGTACCTGTCAGCAATCTCCGCTGTGCTT
GGGGAGAAATTCAGA
[70] Melanonus_zugmayeri_A3
ATG------CTCTCAGAACATGAAAAAGAGCTTATCGTGGGAATATGGACCGAACTG---
ACCCCTTATGCTGATACCATAGGAGCAGGAGCTCTTCTCAGGATGTTCGCGTCATACCCG
GGCACCAAGACGTACTTCACCCACATG---GACATCAACCCACGCTCCGTCCGACTGCTG
ACCCAAGGCGAGAAGATCGTCATGGCGTTGGCGGACGGTTCCAAGGACATAGGCAACCTG
ATGACAAGTTTATCTGCTCTTCAAGCCCACCATGCCTATCAGCTCCGCATTCAACCCACC
AACTTCAAGCTGTTTTCGCACTGTATGCTGGTGACCCTGGCCTGTTTCCTGGGCGATGAC
TTCACGCCAAGTTCACACGCAGCCATGGACAAATACCTGTCAGCCTTCTCCGCTGTACTT
TCGGAGAAATACAGA
[71] Macrourus_berglax_A3
ATG------CTCTCCAAGAAGGAGAAGGAGCTGATAATGGGCATCTGGACCAGACTG---
ACTCCTGCTGCTGAATCCATCGGAGCTGAAGCCCTCCTCAGGATGATCTCGTGCTACCCG
GGCACCAAGACCTACTTCGCCCACCTG---GACACCAGCCTGCGCTCCGCCCACCTGCTG
TCCCATGGCAGGAAGGTGTTCCTGGCCTTGGCCGACGGCTCCAAGGACATCGACAACCTG
CTGACCAGTCTAGCTGCCCTGCAGACCTACCACGCCTATCAGCTCCGCATTCACCCCTTC
AACTTCAAGCTGTTATCCCACTGCATGCTGGTCACCCTCGCCTGCTACATGGGCGACAGC
TTCACGCCCATCGCCCACGCTGCCATGGACAAGTTCCTGTCTGCGTTCTCCGCCGTGCTC
GCTGAGAAGTTCAGA
[72] Malacocephalus_occidentalis_A3
ATG------CTCTCGAAGAAGGAGAAAGACCTGATCGTAGACATCTGGACCAGACTG---
ACTCCGCTTGCTGAAACCATCGGAGCAGAAGCCCTCCTCAGGATGATGACGTGCTACCCG
GGCACCAAGACCTACTTCTCCCACCTG---GACACCAGCCTGCGCTCAGCCCCCCTGCTG
TCCCACGGCCGGAAGGTCTTCCTGTCCTTGGCGGATGGTTCCAAGGACATCGACAACCTG
CTGACCAGTTTAGCTGCTCTTCAGACGTACCACGCCTATCAGCTCCGCATTCACCCCACC
AACTTCAAGCTGCTGTCCCATTGCATGCTGATTACCCTCGGCTGCCACTTGGGGGAGCTC
TTCACTCCCAGCGCTCACGCTGCAATGGACAAGTTTCTGTCGGCCTTCTCAGCTGTGCTT
GCAGAGAAGTTCAGA
[73] Bathygadus_melanobranchus_A3
ATG------CTCTCGAAACGGGAGAAAGATCTTATCATAGAGCTGTGGACCACACTG---
ACTCCTGTTGCTGACACCATAGGAGCAGATGCTCTTCTCAGGATGATCACGTCATACCCG
GGCACCAAGACCTACTTCTCCCACCTG---GACATCAGCCCGCGGTCTGCCCACCTTCTG
TCCCACGGCCGGAAGATCTTCATGGCGTTGGCAGACGGTTCCAAGGACGTCAGCAACTTG
ATGACAGATTTAGCTCCTCTTCAAACCTACCATGCATATCAGCTCCGCATTCACCCAACC
AACTTCAAGTTGTTCTCCCTCTGCATGCTGGTCACCGTAGCCTGCTACATGGGCGAGAGG
TTCACATCGAGCTGGCACGCTGCCATGGACAAGTACCTCTCAGCGTTCTGTGCCGTACTC
GCTGAGAAGTTCAGA
[74] Muraenolepis_marmoratus_A3
ATG------CTCTCGGACCAGGAGAAAGCTCTTATCAGAAACATATGGACCAGACTG---
ACGCCTATGGCTGATACCATCGGAGCAGAAGCTCTCCTCAGGATGCTCACCATGTACCCG
GGGACCAAGACCTACTTCGCCCACCTG---GACATCAGCCCGCGCTCTGCCCACCTGCTG
TCCCACGGCCGGAAGATCGTCGAGGCGCTAGCGGACGGTTCCGAGGACATTGATAACCTG
ATGACCCACTTAGCTCCTCTCCAGACCTATCACGCCTATCAGCTCCGCATCCACCCCACC
AACTTCAAGCTGTTGTCCCACTGCATGGTGGTGACTCTGGCCTGCTACATGGGGGACCGC
TTCACAGCCGTGTCCCACGCCGCCATGGACAAGTACCTCTCGGCCTTCTCTGCTGTACTT
GGGGAGAAGTTCAGA
[75] Bregmaceros_cantori_A3
ATG------CTCTCGCCCAAGGACAAAATCCTTCTCTTAGAAATCTGGGCCACGGTC---
ACACCGTCCGCTTCCAAACTAGGAGCAGAGGCCCTAAACAGGATGTTCCTGTCGTACCCG
GGCTCAAAGACCTATTTCAAGCACTTG---GACATCACCCCGGGGTCAGACTTTGTCAAC
ACTCACGGCAGGAGGATTGTGTTGGCGCTGGCTGAGGGATGCAGGGACATGGACAACCTC
ATGACCAACATCTACCCTCTACAGACATACCACGCCTACAAGCTCCGTATCCAGCCAGTC
AACTTCAAGTTGTTCTGCCACTGCCTGCTGGTGAGCCTCGGCTGTCAGCTGGGTGAGACG

TTCACCCCCACCTTACACGCTGCCATGGACAAATACCTGTCAGCCTACTGTGCTGTGCTG
TCCTCCCGGTTCAGG
[76] Mora_moro_A3
ATG------CTCTCAAAACAGGAGAAACAGCTTATCGCAGAAATATGGACCAGACTG---
ACCCCCGTTGCTGATACCATCGGATCAGAAGCTCTTCTCAGGATGTTCACGGTGTACCCG
GGCACCAAGACGTACTTTTCCCACCTG---GACATCAGCCCGCACTCGGCCTACCTGCTG
TCCCACGGCCGGAAGATCTTCCTGGCTTTGGCGGACGCTTCCAAGGACATAGGCAACCTG
ATGACCCATTTAGCTCCTCTTCAAACCTACCATGCCTATCAGCTCCGCATTGACCCCACC
AACTTCAAGCTGCTGTCACACTGCATGCTGGTCTCCCTCACCTGTTACATGGGCGAGCAC
TTCACGGCAAGTTCACACGCAGCCATGGACAAGTACCTCTCGGCGTTCTCTGCAGTACTC
GCGGAGAAGTTCAGA
[77] Laemonema_laureysi_A3
ATG------CTCTCAAAACAGGAGAAACAGCTTATCGTAGAAATATGGACCAGACTG---
ACCCCTGTTGCTGATACCATCGGAGCAGATACTCTTCTCAGGATGATCACCGTGTACCCG
GGCACCAAGACGTACTTTGCCCACCTG---AACATCAGCCCGCGCTCTGCCCACATGCTG
TCCCACGGCCGGAAGATCTTCCTGGCGTTGGCGGACGGTTCCAAGGACATAGGCAACCTG
ATGACCCATTTAGCTCCTCTTCAGACCTACCACGCCTATCAGCTCCGCATTCATCCCACC
AACTTCAAGCTGTTTTCGCACTGCATGCTGGTCTCCCTCGCCTGTTACATGGGCGAGCGC
TTCACACCTACATCACACGCAGCCATGGACAAGTACCTTTCAGCGTTCTCTGCTGTACTT
GCAGAGAAATTCAGA
[78] Thrachyrincus_scabrus_A3
ATG------CTCTCGAAAAAGGAGAAAGAGCTTATCGTAGAAATATGGACCAAACTG---
ACCCCTATTGCTGATACCATAGGAGCTGAAGGTCTTCTCAGGATGATGACGTCATACCCG
GGCACCAAGACGTACTTTGCCCACCTG---GACATCAGCCCGCACTCTGCCCACCTGCTG
TCCCACGGCCGGAAGATCGTCCTGGCGTTGGCGGACGGTTCCAAGGACATAGGCAACCTG
ATGACCAATTTAGCTCCTCTTCAAACCTACCACGCCTATCAGCTCCGCATTCACCCCAGC
AACTTCAAGCTGTTTTCGCAAAGTCTGCTGGTCGCCCTCGCCTGTTACATGGGTGAGCGC
TTCACACCAGGTTCACACGCAGCCATGAAGAAGTACCTGTCCGCGTTCTGCGCTGTGCTC
GCGGAGAAATACAGA
[79] Gadus_morhua_A4
ATG---AGTCTCACAGACAAAGACAAGGCCCTCATCAAGGGATTCTTTGCCAAGGTC---
TCCAGCAAAGCAGTGGAGATCGGACACCAGACGCTGGCACGCACAATCGTCGTCTACCCC
CAAACCAAAGTCTACTTCTCCCACTGGAAAGACCTCGGGCCCGACTCCCCCAACATTAGG
AAGCATGGCTACACCGTGGTGAAAGGGGTCCTGGATTCCGTCGACCTGATTGACGACCTT
GTCGGTGGTCTTCTCGAGCTCAGTGAGCTCCATGCTTTCCGTCTGCGCATTGACCCTGCA
AACTTCAAGATCTTGAACCTCAACCTGGTGGTCGTGCTGGGACTGATGTTCCCAGATGAT
TTCACTCCTCAGGTGCATGTTTCTGTCGACAAATATTTAGCTCTAATTTGTCTGGCATTG
TGCGAAAAGTATAGG
[80] Arctogadus_glacilis_A4
ATG---AGTCTCACAGACAAAGACAAGGCCCTCATCAAGGGATTCTTTGCCAAGATC---
TCCAGCAAAGCGGTGGAGATCGGACACCAGACGCTGGCACGGACAATCGTTGTCTACCCC
CAAACCAAAGTCTACTTCTCCCACTGGAAAGACCTCGGGCCCGACTCCCCCAACATTAGG
AAGCATGGCTACACCGTGGTGAAAGGGGTCCTGGATTCCGTCGACCTGATTGACGACCTT
GTCGGTGGTCTTCTCGAGCTCAGTGAGCTCCATGCTTTCCGTCTGCGCATTGACCCCGCA
AACTTCAAGGTCTTGAACCTCAACCTGGTGGTCGTGCTGGGACTGATGTTCCCAGATGAT
TTCACTCCTCAGGTGCATGTTTCTGTCGACAAATATTTAGCTCTAATTTGTCTGGCATTG
TGCGAAAAGTATAGG
[81] Boreogadus_saida_A4
ATG---AGTCTCACAGACAAAGACAAGGCCATCATCAAGGGATTCTTTGCCAAGATC---
TCCAGCAAAGCGGTGGAGATCGGACACCAGACGCTGGCACGCACAATCGTCGTCTACCCC
CAAACCAAAGTCTACTTCGCCCACTGGAAAGACCTCGGGCCCGACTCCCCCAACATTAGG
AAGCATGGCTACACCGTGGTGAAAGGGGTCCTGGATTCCGTTGACCTGATTGACGACCTT
GTCGGTGGTCTTCTCGAGCTCAGTGAGCTCCATGCTTTCCGTCTGCGCATTGACCCTGCA
AACTTCAAGATCTTGAACCTCAACCTGGAGGTCGTGCTGGGAATGATGTTCCCAGATGAT
TTCACTCCTCAGGTGCATGTTTGTGTCGACAAATATTTAGCTCTAATTTGTCTGGCATTG
TGCGAAAAGTATAGG
[82] Trisopterus_minutus_A4

ATG---AGTCTCACAGACAAAGACAAGTCCACCATCAAGGAATTCTTTGCCAAGATCACC
ACCAGCAAAGCGGTGGAGATCGGACACCAGACGCTGGCACGCACGGTCGTCGTCTACCCC
CAAACCAAGGTTTACTTCTCCCACTGGAAAGACCTCGGGCCCGACTCTCCCAACATCAGG
AAGCACGGCTACACCGTGGTGAAGGGGGTCCTGGATTCCGTCGCGCTGATTGACGATCTC
GTCGGTGGTCTTCTCACCCTCAGTGAGCTCCATGCTTTCCGTCTGCGCATTGACCCTGCC
AACTTCAAGATCTTGAACCTCAACCTGGAGGTCGTGCTGGCATTGATGTTCCCTGATGAG
TTCACTCCTCAGGTGCATGTGGCTGTGGACAAATACCTAGCTCTTATTTGCCTGGCATTG
TGCGAGAAGTATCGG
[83] Pollachius_virens_A4
ATGACGAGTCTCACAGACAAAGACAAGGCCCTCATCAAGGGATTCTTTGCCAAGGTT---
TCCAGCAAAGGGGTGGAGATCGGACACCAGACGCTGGCACGGACAATCGTCGTCTACCCC
CAAACCAAAGTCTATTTCTCCCACTGGAAAGACCTCGGGCCCGACTCCCCCAACATTAGG
AAGCATGGCTACACCGTGGTGAAAGGGGTCCTGGATTCCGTCGAGCTGATTGACGACCTC
GTCGGTGGTCTTCTCGAACTCAGTGAGCTCCATGCTTTCCGTCTGCGCATTGACCCTGCA
AACTTCAAGATCTTGAACCTCAACCTGGAGGTCGTGCTGGCAATGATGTTCCCAGATGAG
TTCACTCCTCAGGTGCATGTGGCTGTCGACAAATATTTAGCTCAAATTTGTCTGGCATTG
TGCGAAAAGTATAGG
[84] Melanogrammus_aeglefinus_A4
ATG---AGTCTCTCAGACAAAGACAAGGCCCTCATCAAGGGATTCTTTGCCAAGATT---
TCCAGCCAAGCGGTGGAGATCGGACACCAGACGCTGGCACGGACAATCGTCGTCTACCCC
CAAACCAAAGTCTACTTCTCCCACTGGAAAGACCTCGGGCCCGACTCCCCCAACATTAGG
AAGCATGGCTACACCGTGGTGAAAGGGGTCCTGGATTCCGTCGACCTGATTGACGACCTC
GTCGGTGGTCTTCTCGAGCTCAGTGAGCTCCATGCTTTCCGTCTGCGCATTGACCCTGCA
AACTTCAAGATCTTGAACCTCAACCTGGAGGTCGTGTTGGGACTGATGTTCCCAGATGAC
TTCACTCCTCAGGTGCATGTGGCTGTCGACAAATATTTAGCTCAAATTTGTCTGGCATTG
TGCGAAAAGTATAGG
[85] Merlangius_merlangus_A4
ATG---AGTCTCTCAGACAAAGACAAGGCCCTCATCAAGGGATTCTTTGCCAAGATT---
TCCAGCCAAGCGGTGGAGATCGGACACCAGACGCTGGCACGGACAATCGTCGTCTACCCC
CAAACCAAAGTCTACTTCTCCCACTGGAAAGACCTCGGGCCCGACTCCCCCAACATTAGG
AAGCATGGCTACACCGTGGTGAAAGGGGTCCTGGATTCCGTCAGCCTGATTGACGACCTC
GTCGGTGGTCTTCTCGAGCTCAGTGAGCTCCACGCTTTCCGTCTGCGCATTGACCCTGCA
AACTTCAAGATCTTGAACCTCAACCTGGAGGTCGTGCTGGGAATGATGTACCCAGATGAC
TTCACTCCTCAGGTGCATGTGGCTGTCGACAAATATTTAGCTCAAATTTGTCTGGCACTG
TGCGAAAAGTATAGG
[86] Theragra_chalcogramma_A4
ATG---AGTCTCACAGACAAAGACAAGGCCCTCATCAAGGGATTCTTTGCCAAGATC---
TCCAGCAAAGCGGTGGAGATCGGACACCAGACGCTGGCACGGACAATCGTCGTCTACCCC
CAAACCAAAGTCTACTTCTCCCACTGGAAAGACCTCGGGCCCGACTCCCCCAACATTAGG
AAGCATGGCTACACCGTGGTGAAAGGGGTCCTGGATTCCGTCGACCTGATTGACGACCTT
GTCGGTGGTCTTCTCGAGCTCAGTGAGCTCCATGCTTTCCGTCTGCGCATTGACCCCGCA
AACTTCAAGATCTTGAACCTCAACCTGGTGGTCGTGCTGGGATTGATGTTCCCAGATGAT
TTCACTCCTCAGGTGCATGTTTCTGTCGACAAATATTTAGCTCTAATTTGTCTGGCATTG
TGCGAAAAGTATAGG
[87] Gadiculus_argentus_A4
ATGACTAGCCTCTCAGCCAAAGACAAGTCCGTCATCAAGGATTTCTTTGCCAAGGTG---
GCCCCTCAAATGATTGAGATTGGACACCAGGCACTGGCACGGACAATCGTCGTCTACCCC
CAGACCAAGGTGTACTTCTCCCACTGGGCAGACCTCGGGCCCGACTCCCCCAACATCAGG
AAGCATGGCTACACCGTGGTGAAGGGCATCATCGATTCCGTCGACCTGATCGACGACCTG
ATCGGTGGTCTTCTGGAGCTCAGTGAGCTGCACGCCTTCCGTCTGCGTATTGACCCTGCA
AACTTCAAGATCTTCAACAGCAACCTGGAGATCTCCTTGGGAATGATGTTCCCAGATGAC
TTCACTCCTCAGGTGCATGTCGCTGTGGACAAGTATCTGGCTCTCATTTGCATGGCTCTG
TGCGAGAAGTACCGG
[88] Phycis_phycis_A4
ATG---AGTCTCACAGACAAAGACAAGGCCCTCGTCAAGGGATTCTTTGCCAAGGTT---
TCTAGCAAAGCGGTCGAGATCGGACACCAGACGCTCGCACGGACAATTGTCGTCTTCCCT
CAAACCAAGACTTACTTCTCCCACTGGAAAGACCTGGGCCCCGACTCCCCCAACGTGAGG

AAACATGGCTACACCGTGGTCAAGGGGGTCATTGATTCCGTGGATCTGATTGACGACCTC
GTCGGAGGTCTTCTCACCCTCAGTGAGCTCCATGCCTTCCGTCTGCGCATCGACCCTGCA
AACTTCAAGATCATCAACCTCAACCTGGAGGTAGTGCTGGCATTGATGTTCCCCGATGAC
TTCACTCCTCAGGTGCACGTGGCTGTGGACAAATATTTAGCTCTACTTTGTCTGGCATTG
TGCGAAAAGTATCGG
[89] Molva_molva_A4
ATG---AGTCTCACAGACAAAGACAAAGCCCTCATCAGGGGATTCTTTTCCAAGGTT---
TCTGGCAAAGCGGTGGAGATCGGACACCAGACGCTGGCACGGACAATTGTCGTCTACCCC
CAAACCAAGACTTACTTCTCCCACTGGAAAGACCTAGGACCCGACTCCCCCAATGTGAGG
AAGCATGGCTACACCGTGGTGAAGGGGGTCCTGGATTCCGTCGAACTGATTGACGACCTC
GTCGGTGGTCTTCTCACCCTCAGTGAGCTCCATGCTTTCCGTCTGCGCATCGACCCTGCA
AACTTCAAGATCTTGAACCTCAACCTGGTGGTAGTGCTGGGAATGATGTTCCCAACTGAG
TTCACTCCTCAGGTGCACGTGGCTGTGGACAAATATTTAGCTCTAATTTGTCTGGCATTG
TGCGAAAAGTATCGG
[90] Brosme_brosme_A4
ATG---AGTCTCACAGACAAAGACAAGGCCCTCATCAAGGGATTCTTTTCCAAGGTT---
TCTGGCAAAGTGGTGGAGATCGGACACCAGACGCTGGCACGCACAATTGTCGTCTACCCC
CAAACCAAGACTTACTTCTCCCACTGGAAAGACCTAGGGCCCGACTCCCCCAACGTGAGG
AAGCATGGCTACACCGTGGTGAAGGGGGTCTTTGATTCCGTCGAGCTGATTGACGACCTC
GTCGGTGGTCTTCTCACCCTCAGTGAGCTCCATGCTTTCCGTCTGCGCATCGACCCTGCA
AACTTCAAGATCTTGAACCTCAACCTGGTGGTAGTGATGGGAATGATGTTCCCAGATGAG
TTCACTCCTCAGGTGCACGTGGCTGTGGACAAATATTTAGCTCAAATTTGTCTGGCATTG
TGCGAAAAGTATCGG
[91] Merluccius_merluccius_A4
ATG---AGTCTCTCAGAAAAAGACAAGGCCCGCATCAAGGGCTTCTTCTCCCTGGTG---
GCCGACCAAGCGAAGGACATCGGACACCAGACGCTCGCACGGNNNNNNNNNNNNNNNNNN
NNNNNNNNNNNNNNNNNNNCCCACTGGAAGGACCTGAGCCCCAACTCCCCCAACGTGAAG
AAGCACGGCTACACCGTGGTGAAGGGGGTCATTGACTCCGTGGACCTGATTGACAACCTG
AACGACGGTCTCCTCAACCTCAGTGAGCTCCACGCTTTCCGTCTGCGCATCGACCCTGCA
AACTTCAAGATCCTGAACAGCAACCTGCAGATCGTGCTGGCGATGATGTACCCAGCTGAG
TTCACCCCTCAGGTGCATGTCTCTGTGGACAAGTATTTAGCTCTCTTGTGCATGGGATTG
TGCGAAAAGTATCGG
[92] Merluccius_capensis_A4
ATG---AGTCTCTCAGAAAAAGACAAGGCCCGCATCAAGGGCTTCTTCTCCCTGGTG---
GCCGACCAAGCGAAGGACATCGGACACCAGACGCTCTCACGGACACTCATCGTCTACCCC
CAGACCAAGGTGTACTTCGCCCACTGGCAGGACCTGGGCCCCAACTCCCCCAACGTGAAG
AAGCACGGCTTCACCGTGGCGAAGGCGATCATTGACTCCGTGGACCTGATTGACAACCTG
AACGACGGTCTCCTCAACCTCAGTGAGCTCCACGCTTTCCGTCTGCGCATCGACCCTGCA
AACTTCAAGATCCTGAACAGCACCCTGCAGATCGTGCTGGCAATGATGTACCCAGCTGAG
TTCACCCCTCAGGTGCATGTCTCTGTGGACAAGTATTTAGCTATGTTGTGCATGGCATTG
TGCGAAAAGTATCGG
[93] Merluccius_polli_A4
ATG---AGTCTCTCAGAAAAAGACAAGGCCCGCATCAAGGGTTTCTTCTCCCTGGTG---
GCCGACCAAGCGAAGGACATCGGACAGCAGACGCTCGCACGGACACTCGTCGTCTACCCC
CAGACCAAGGTGTACTTCGCCCACTGGAAGGACCTGGGCCCCAACTCCCCCAACGTGAAG
AAGCACGGCTACACCGTGGTGAAGGGGGTCATTGACTCCGTGGACCTGATTGACAACCTG
AACGACGGTCTCCTCAACCTCAGTGAGCTCCACGCGTTCCGTCTGCGCATCGACCCTGCA
AACTTCAAGGTCCTGAACAGCAACCTGCAGGTCGTGCTGGCAATGATGTACCCAGCTGAG
TTCACCCCTCAGGTGCATGTCTCTGTGGACAAGTATTTAGCTCTGTTGTGCATGGGATTG
TGCGAAAAGTATCGG
[94] Thrachyrincus_murrayi_A1
ATG---AGTCTCACCGAGAAAGACAAGGCGACCGTCAAGCTCTTCTGGAACAAGGTG---
GCCGGAAAGGCCGAGCTTGTCGGCTCCGACGCGCTGTCAAGGATGCTGCACGTGTACCCG
CAGACCAAGACGTACTTCAGCCACTGGAAGGACATGAGCCCCGGCTCCGCTCCCGTGAGG
AAGCACGGCAAGATCATCTTGATGGGCATCGGAGAGGCTGTGAAGAAAATGGACGACCTT
GACGCAAACCTCCTCACTCTCAGCGAGCTGCACGCCTTCCAGCTGAGAGTGGACCCCCACC
AACTTCAGGCTTCTCAACCTCAACCTCATCGTGGTTCTGGCTATTATGTTCCCTGAGGAA

TTCACCCCGATGGCTCATGTGGCCGTGGATAAGTTCCTGTGCGCCCTGGCTCTGGCCCTT
TCCGAGAAGTACCGA
[95] Thrachyrincus_murrayi_A2
ATG---AGTCTGAGCTCCAAGCAAAAGATTATAGTCAGGGATTTCTTCAAATTGATC---
TCCAGCAGGTCCGAGGATATCGGAGCCGAGTCTCTTTCCAGGTTGGTCGCTGTGTACCCA
CAGACCAAGTCCTACTTCTCCCACTGGAAGTCTACCGACCCCGGCTCTGCCCCCGTCAGG
AAACATGGCATCACCATCATGGGTGGAGTGTACAAAGCTATCGACAACATCGACGACTTG
AAGGGAGGACTCCTCGGCCTCAGCGAGCTGCACGCATTCATGCTGAGGGTTGACCCAGTC
AACTTCAAGCTCCTGGGACACTGTTTGCTGGTGTGCATGTCCGTGTACTTCCCCGATGAC
TTCACCCCCCAGGTCCACGTGGCCGTCGACAAATTCCTCGGCCGCCTGGCCCTGGCTCTC
TCCGACAAGTACCGT
[96] Thrachyrincus_murrayi_A3
ATG------CTCTCGAAAAAGGAGAAAGAGCTTATCGTAGAAATATGGACCAAACTG---
ACCCCTATTGCTGATATCATAGGAGCTGACGGTCTTCTCAGGATGATGACGTCATACCCG
GGCACCAAGACGTACTTCGCCCACCTG---GACATCAGCCCGCGCTCTGCCTACCTGCTG
TCCCACGGCCGGAAGATCGTCCTGGCGTTGGCGGACGGTTCCAACGACATAGGCAACCTG
ATGACCAATTTAGCTGATCTTCAAACCTACCACGCCTATCAGCTCCGCATTCACCCCATC
AACTTCAAGCTGTTTTCGCAAAGTCTGCTGGTCGCCATCGCCTGTTACATGGGTGAGCGC
TTCACACCAAGTTCACACGCAGCCATGGACAAGTACCTGTCAGCGTTCTGCGCTGTGCTC
GCGGAGAAATACAGA
[97] Phycis_blennoides_A1
ATGACTAGTCTCAATGATAAAGACAAGGCCACCGTCAAGCTCTTCTGGAGGAAGATG---
TCCACAAAGGCCGAGGTTGTCGGGTCCGATGCTCTGACAAGGATGCTGGCTGTGTACCCG
CAGACCAAGACCTACTTCAGTCACTGGAAGGACCTGAGCCCTGGCTCCAGTGCGGTCAGG
AAGCACGGCAAGACCGTCATGATGGGCATCGGCGAGGCTGTGAGCAAAATGGAAGACCTG
AACGCTGGTCTTCTCAGTCTCAGCGAGCTGCACGCCTTCAACCTGAGAGTTGATCCCACC
AATTTCAGGCTTCTTTCCTCCAACATCATGATTGTGATGGCCATCATGTTCCCTAATGAC
TTCACCGAGGTGGCTCATCTTGCCGTCGATAAGTTCCTGTGCGCTGTGGCCCAGGCCCTC
TCCGAGAAGTACCGA
[98] Phycis_blennoides_A2
ATG---AGTCTGACCTCCAAACAAAAGGCCACAGTGAAGGACTTCTTCAACAAGGTG---
GGCAGCAGATCCGAGGAGATCGGAGCCGAAGCTCTGTCCAGGCTGGTCGCTGTGTACCCA
CAGACTAAGTCCTACTTTGCCCACTGGAAGGATGTGAGCCCCGGCTCTGCCCCCGTCAGG
AAGCATGGCATCACCATCATGGGTGGAGTGTACGATGCCGTCGGCAAGATTGACGACCTG
AAGGGAGGACTCCTCAGCCTCAGCGAGCTGCACGCTTTCATGCTGAGGGTTGACCCCGTC
AACTTCAAGCTCCTGGCCCACTGCATGCTGGTCTGCATGTCCATGCTCTTCCCCGAGGAA
TTCACCCCCCAGGTCCACGTGGCCGTCGACAAATTCCTGGCCCAGCTTGCCCTGGCTCTG
GCCGAGAAGTACCGT
[99] Phycis_blennoides_A3
ATG------CTCTCGAAAAAGGAGAAAGAGCTAATTGAGGAGGTATTGAGCGGCCTG---
AGCCCTGTCGCCGACACCATCGGAGCAGAGGCTCTGCTGAGGATGTTCACGTCCTACCCC
GGCACCAAGACGTACTTCCGCCACCTG---GACATCGGCCCACGCTCCCCCCACCTGCTG
TCCCACGGCGAGAAGATCGTCATGGCGTTGGCAGAAGGTTGCAAGGACATCGGCAACCTG
ATGACCAATCTGGCTGCTCTTCAGACCTACCATGCCTACCAGCTCCGCATCCACCCCACC
AACTTCAAGCTGTTTTCGCACTGTATGCTGGTCACCCTCGCCTGCTACATGAGAGAAGAC
TTCACGCCCGTTGTGCACGCAGCCATGGACAAGTATCTCTCCGCGTTCTCCGCCGTGCTG
GCTGAGAAGTTCCGA
[100] Phycis_blennoides_A4
ATG---AGTCTCACAGACAAAGACAAGGCCCTCGTCAAGGGATTCTTTGCCAAGGTT---
TCTAGCAAAGCGGTCGAGATCGGACACCAGACGCTCGCACGGACAATTGTTGTCTTCCCT
CAAACCAAGACTTACTTCTCCCACTGGAAGGACCTCGGCCCCGACTCCCCCAATGTGAGG
AAACATGGCTACACCGTGGTCAAGGGGGTCATGGATTCTGTGGATCTGATTGACGACCTC
GTCGGGGGTCTTCTCACCCTCAGTGAGCTCCATGCTTTCCGTCTGCGCATCGACCCTGCA
AACTTCAAGATCATCAACCTCAACCTGGAGGTAGTGCTGGCATTGATGTTCCCCAATGAG
TTCACTCCTCAGGTGCACGTGGCTGTGGACAAATATTTATCTCTACTTTGTCTGGCATTG
TGCGAAAAGTATCGG
[101] Stylephorus_chordatus_A1_1

ATG---AGTCTAAGTGTTAAGGACAGGGCTATCGTCAAGGATTTCTGGACCAGGGTG---
TCTGGAAAGGTGGAGGAGGTCGGAGCCGATGCTCTGTCAAGGATGCTGACTGTGTACCCA
CAGACCAAAACCTACTTCTCTCACTGGAAGGACCTGCGTCCTGGCTCCGCTCCTGTGAGG
AAGCATGGAAAGACTGTCTTGATGGCAATAGGAGACGCCGTGTCAAAAATGGACAATCTT
GTTGGAGGGCTTCTCACTCTCAGTGAGCTGCATGCATTCCAGCTGAGAGTTGATCCTGCT
AACTTCAAGATCTTAAACCAGAACCTCCTGGTGGTTCTGGCCATTATGTTCCCGGATGAC
TTCACTCCTGAGGCTCATGTGGCCGTAGATAAGTTCCTCGCTGCCTTGGCTCTGGCTCTC
TCTGAGAAATACCGA
[102] Stylephorus_chordatus_A1_2
ATG---AGTCTAAGTGCTAAGGACAGGGCTATCGTCAAGACATTCTGGACCAAGGTG---
TCTGGAAATGTGGAGGAAGTCGGAGCCGATGCTCTGTCAAGGATGCTGACTGTGTACCCA
CAGACCAAAACCTACTTCTCTCACTGGAAGGACCTGAGACCTGGCTCCACTCCTGTGAGG
AAGCATGGAAAGGTTATCTTGACGGCAATAGGAGACGCCGTGACAAAAATTGACGATCTT
GTTGGAGGGCTTCTCGCTCTCAGCGAGCTGCATGCATTCAAGCACAGAGTTGATCCTGCT
AACTTCAAGATCTTAAACCACAACCTCCTGCTGGTTCTGGCCATTATGTTCCCAGATGAA
TTCACTCCTGAGGCTCATGTGGCCGTAGATAAGTTCCTTGCTGCCATGGTTGTGGCTCTC
TCTGAGAGATACAGA
[103] Stylephorus_chordatus_A1_3
ATG---AGTCTAAGTGCTAAGGACAGGGCTATCGTCAAGACATTCTGGACCAAGGTG---
TCTGGAAATGTGGAGGAAGTCGGAGCCGATGCTCTGTCAAGGATGCTGACTGTGTACCCA
CAGACCAAAACCTACTTCTCTCACTGGAAGGACCTGAGACCTGGCTCCGCTCCTGTGAGG
AAGCATGGAAAGATTGTCTTGACGGCAATAGGAGAGGCCGTGAAAAAAATTGACGATCTT
GTTGGAGGGCTTCTCACTCTCAGCGAGCTGCATGCCTTCCAGCTGAGAGTTGATCCTGCT
AACTTCAAGATCTTAAACCACAACCTCCTGCTGGTCCTGGCCATTATGTTCCCAGATGAG
TTCACTCCAGAAGCTCATGTGGCCGTAGATAAGTTCCTCGCTGCCTTGGCTCTGGCTCTC
TCTGAGAAATACAGA
[104] Stylephorus_chordatus_A3
ATG------CTCTCGCAACGGGAGAAAGACCTTATTATGGAAATATGGGAGAGATTG---
ACTCCTGTTGCTGATGATATTGGGGCAGAAGCGCTTCTCAGGATGTTCACATCATATCCT
GGCACCAAGACTTACTTCTCTCACCTG---GACCTCTCTCCACGCTCTCCTAACCTGCTG
TCCCATGGGAAGAAGATTGTTTTAGCCCTAGCTGAGGGATCCAAGGACATCAGCCAGCTG
ATGACAACTTTAGCACCACTTCAAACCTATCACGCCTATCAGCTTCGAATTGACCCAACT
AACTTCAAGCTTCTTTCACACTGCATGCTCGTCACCCTGGCCTGTTACATGCGTGACCAC
TTCTCGCCACGTGCGCACGCAGCCATGGACAAGTACCTCTCAGCGTTTGCAGCCGTACTC
GCAGAGAAATTCAGA
[105] Percopsis_transmontana_A2_2
ATG---AGTCTCAGCGCTAAAGACAAAGCTACCGTGAGGGATTTCTTCGCCAAGATT---
GGCAGCAAGTCTGACGACGTTGGCACCGAGGCCCTTGCCAGGACTCTGTCCGTATACCCC
CAGACTAAAACCTACTTCTCTCACTGGAAAGACCTGAGCGCCACCTCCCCCAACGTCAGG
AAGCATGGCAAGACCGTCATGACAGGCGTCCTGGACGCTGTGGAAAAGATGGACAACTTG
ACAGCAGGTCTGCTGACTCTAAGCGAGCTGCACGCCTTCATGCTGCGGATTGATCCTGCC
AACTTCAAGATCATTTCTCATAACCTGCTGGTCTCCATGGCTATGTTGTTTCCCGATGAA
TTCACCCCGCAAGTTCACGTGTCTGTGGACAAGTTCCTTGCCAACCTCGCTCTGGCTCTG
GCCGAGAAGTATCGC
[106] Percopsis_transmontana_Ax
ATG---AGTCTCACTAAGAAGGACAAGGCAGCGGTCAGGGAGTTTTGGGAAAAGATC---
AAAGGTAAAAGAGAAGACATTGGTGGCGACGCTCTGGCGAGGACACTGCAAGTGTACCCG
CAGACAAAGACTTATTTCTCCCACTGGAAGGACCTGAGCCCCAGCTCTGCAGCAGTCAAG
AAGCATGGACTGACCATCATGGACGGAGTTGGAGAGGCTGTGAGCAAAATTGACGACCTT
ACCACAGGTCTCCTCACTCTCAGTGAGCTGCACGCCTTCAAGCTGCGAGTGGATCCTGCT
AACTTCAAGATCCTCACCCTCAATCTCCTCGTGGTTTTGGGCATCATGTTCCCCAAGGAC
TTCACCCCCCAGGTCCACGTGTCTCTTGACAAGTTCATGGCCGCCTTGTCCCTGGCTCTG
GCTGAGAAGTACAGA
[107] Zeus_faber_Ax2
ATG---AGTCTCTCGGGAAAAGACAAGGCTGTGGTGAATGCGTTCTGGAAGCTGGCC---
TCTCCAAGGTCTGCCGACATTGGTGGAGAAGCTCTGGGCAGGATGCTCACTGTTTATCCG
CAGACCAAGGCTTATTTCTCCCACTGGGACAGTCAGAGAGCCGGAACAAAACGGGTCAAG

ACACATGGTGCAATTATCATGGAAGCGGTCGGTAACGCGATCATAAACATTGGTGACCTT
GACAATTTTTTCTCCAACCTGAGTGAACTGCACGCTTTCAAACTGAGAATTGACCCTTCA
AACTTCAAGATTCTGGCCCACAACCTTATCGTGGTCATGGCCATGCTCTATCCCAATGAC
TTCAGTCCCGAGGTCCATTTGTCGGTTGACAAGTTCTTTATGAGGCTAGCCCTGGCCCTG
TCTGAGAAGTACCGC
[108] Zeus_faber_A2
ATG---AGTCTGAGCACCAAGGACAAGGCCACCGTCAGGGCCTTCTTCGGCAAAGTG---
AGCAGCAGGAAGGAGGAGATCGGCAGCAATGCTCTTTCCAGGATGGTGGCGGTGTACCCA
CAGACCAAATCCTACTTCGCCCACTGGAAGGACCTGGGACCCAACTCCGCCCCGGTGAAG
AAGCACGGGATCACCATCATGAACGGCGTGTACGACGCCGTGAGCAAGTTGGACGATCTG
AAGGGAGGCCTGCTCAGCCTGAGCGAGCTGCACGCCTTCATGCTGAGGGTCGACCCCGTC
AACTTCAAGCTCCTCACCCACTGCATCCTGGTGGTGATGGCCATCCTGTTCCCCGAGGAC
TTCATCCCTCAGGTGCACGTTTCCGTCGACAAGTTCCTGGCTCAGCTGGCCCTGGCCCTC
GCCGAGAAGTACCGT
[109] Zeus_faber_A1
ATGACT---CTCTCCAATAAGGACAAGGAATACGTGAGGACCTTCTGGGCCAAGGTG---
TCCGTACAGGTTGAAGATGTCGGAAACGATGTTCTCTCCAGGATGTTGGCGGTTTACCCC
CAGACCAAGACCTACTTCTCCCACTGGAAGGACCTGAGTCCCGGCTCAGCCCTGGTCAGG
AAGCACGGCAAGGTCGTGATGATGGGAGTGGGTGAGGCCGTGGGCAAGATCGACGACCTG
GAAAAAGGCCTCCTCACCCTCAGTGAGCTGCACGCCTTCCAGCTGAGAGTGGACCCCGCC
AACTTCAAGATCCTCTGCCACAACATCCTCGTGGTCCTGGCCATCAAGTACCCCAATGAG
TTCACTGCACAGGTCCACATGTCCTTGGACAAGTTCCTCTGTGCCTTGTCTCTGGCTCTT
GCTGAGAAATACCGA
[110] Zeus_faber_A3
ATG------TTAACGCAGCGAGAGAAGGAGCTTCTTGCGCAGATATGGGAGAGAAAT---
ATTCCCGTTGCCGATGACATTGGGGCAGAAGCTCTTCTTAGGATGTTCACGACTTTCCCC
GGCTCCAAGACTTACTTCTCTCACCTG---GACATCAGCCCCCGCTCACCGCACCTCCTG
TCCCACGGCCGGAAGATTGTCCTGGCCATCGCCAAGGCGTCCAAGGACATCGACCATCTG
ACAGAGACTCTGGCCCCGCTCCAAACGCTGCACGCCTACCGGCTCCGAATTGACCCCGCT
AACTTCAAGCTTTTCTCGCACTGTATGCTGGTCACCCTGGCCAGTAACATGGGAGACAGC
TTCACGCCGGTTGCACATGCGGCCATGGACAAGTATCTTTCTGCGTTCTCAGCCGTGCTC
TCTGAGAAATACAGA
[111] Zeus_faber_Ax1
ATG---GGTTTCACAGCCAAAGAAAAAACCCTCATCAAGACCTTCTTTGCCACAATT---TCT
GACAAAACTCAAGAAATTGGGCACCAATCACTGGCACGGACCATCGTGGTCTACCCCCAG
ACCAAGACTTACTTCGCCCACTGGAAGGACCTCAGTCCCAACTCCCCCAATGTGAAGAAG
CACGGCAACACCGTGGTGAAGGGACTGATAGATGCCGTGGACATGATCGATGACCTGGTG
GGAGGACTGCTCCCCCTCAGCGAGCTGCACGCCTTCCGTCTGCGCATCGACCCTGCCAAC
TTCAAGATCCTGATCCACAACATGCTGGTGGTGCTGGCCATCATGTACCCCAATGAATTC
ACCCCTATTGTCCATGTGACTCTGGACAAGTACTGCGCCAAGGTGTCCCAGGCCCTGTCC
GACAAGTATCGG
[112] Danio_rerio_MN_Hba1
ATG---AGTCTGACAGCTAAGGATAAAGCTGTGATCAAGGGCTTCTGGGGGAAAATC---
GCCAGTCAGGCAGATTCCATAGGACAAGAAGCTATGGGGCGAATGCTTACTGTCTACCCA
CAGACTAAAATATATTTCGCTCATTGGCCTGACCACTCTCTTGGATCTGCTCAAGTGAAG
AAACATGGCAAAATTGTGATGGGTGCTATCACCGATGCTGTGGGAAAAATAGACGATCTT
CTTGGTGCATTGAGCTCCTTGAGTGATCTCCATGCCACCAAACTCCGCGTAGACCCTGGA
AACTTCAAGATTTTGTCCCATAACATCCTCGTGACACTTGCAGTTCATTTCCCCGCTGAC
TTCACCGCAGAAGTACACGTGGCTGTGGACAAGTTCCTCGCTGCTTTGTCTGCTGCTCTT
GCTGATAAATACAGA
[113] Danio_rerio_MN_Hba2
ATG---AGTCTCTCTGATACGGACAAGGCTGTTGTTAAGGCCATCTGGGCTAAGATC---
AGCCCCAAGGCCGATGAAATTGGTGCTGAAGCCCTCGCCAGAATGCTGACCGTCTACCCT
CAAACCAAGACCTACTTTTCTCACTGGGCTGACTTGAGCCCTGGGTCTGGTCCCGTGAAG
AAGCACGGAAAGACTATCATGGGTGCCGTCGGCGAAGCTATTTCAAAAATAGACGACCTT
GTGGGAGGACTGGCCGCCCTGAGCGAACTCCATGCCTTCAAGCTGCGTGTTGACCCGGCC
AACTTCAAGATCCTGTCACACAATGTCATTGTGGTCATCGCCATGCTCTTCCCTGCAGAC

TTCACCCCTGAGGTTCACGTGTCAGTCGACAAGTTCTTTAATAACTTGGCCCTGGCTCTC
TCTGAGAAGTACCGC
[114] Danio_rerio_MN_Hba3
ATG---AGTCTCTCTGATAAGGACAAGGCTGTTGTTAAGGCCATCTGGGCTAAGATC---
AGCCCCAAGGCCGATGAAATTGGTGCTGAAGCCCTCGCCAGAATGCTGACCGTCTATCCT
CAGACCAAGACCTACTTCTCTCACTGGTCTGACTTGAGCCCTGGGTCTGGTCCCGTGAAG
AAGCACGGAAAGACTATCATGGGTGCCGTCGGCGAAGCTATTTCAAAAATAGACGACCTT
GTGGGAGGACTGGCCGCCCTGAGCGAACTTCATGCCTTCAAGCTGCGTGTTGACCCGGCC
AACTTCAAGATCCTGTCACACAATGTCATTGTGGTCATCGCCATGCTCTTCCCTGCAGAC
TTCACCCCTGAAGTTCACGTGTCAGTCGACAAGTTCTTTAATAACTTGGCCCTGGCTCTC
TCTGAGAAGTACCGC
[115] Danio_rerio_MN_Hba4
ATG---AGTCTCTCTGCCAAAGACAAAGCTGCCGTCAAAACCCTGTGGGCCAAGATC---
GCTGGAAAGGCTGACGACATCGGACACGATGCTCTCTCCAGGATGTTGATTGTCTACCCC
CAGACCAAGACCTACTTCTCTCACTGGAAAGACCTGAGCCCAGGCTCTGCCCCAGTGAGG
AAACACGGCAAGACTGTGATGGGAGGCGTTGCTGAGGCTGTCAGCAAAATCGATGACCTT
AATGCCGGACTCCTGAACCTCAGTGAGCTCCATGCTTTCCAGCTGCGTGTGGACCCCGCC
AATTTCAAGATTCTGTCCCACAACATCCTCGTGGTTCTGGCCACTTTGTTCCCCGCCGAT
TTCACTCCTGAGGCTCATGTTGCAATGGACAAGTTCCTCTCAGCTCTGGCTCTGGCCATG
TCTGAGAAGTACAGA
[116] Danio_rerio_MN_Hba5
ATG---AGTCTCTCTGCCAAAGACAAAGCTGCCGTCAAAACCCTGTGGGCCAAGATC---
GCTGGAAAGGCTGACGACATCGGACACGATGCTCTCTCCAGGATGTTGATTGTCTACCCC
CAGACCAAGACCTACTTCTCTCACTGGAAAGACCTGAGCCCAGGCTCTGCCCCAGTGAGG
AAACACGGCAAGACTGTGATGGGAGGCGTTGCTGAGGCTGTCAGCAAAATCGATGACCTT
AATGCCGGACTCCTGAACCTCAGTGAGCTCCATGCTTTCCAGCTGCGTGTGGACCCCGCC
AATTTCAAGATTCTGTCCCACAACATCCTCGTGGTTCTGGCCACTTTGTTCCCCGCCGAC
TTCACTCCTGAGGCTCATGTTGCAATGGACAAGTTCCTTTCAGCTCTGGCTCTGGCCATG
TCTGAGAAGTACAGA
[117] Danio_rerio_MN_Hba6
ATG---AGTCTCTCTGCCAAAGACAAAGCTGCCGTCAAAACCCTGTGGGCCAAGATC---
GCTGGAAAGGCTGACGACATCGGACACGATGCTCTCTCCAGGATGTTGATTGTCTACCCC
CAGACCAAGACCTACTTCTCTCACTGGAAAGACCTGAGCCCAGGCTCTGCCCCAGTGAGG
AAACACGGCAAGACTGTGATGGGAGGCGTTGCTGAGGCTGTCAGCAAAATCGATGACCTT
AATGCCGGACTCCTGAACCTCAGTGAGCTCCATGCTTTCCAGCTGCGTGTGGACCCCGCC
AATTTCAAGATTCTGTCCCACAACATCCTCGTGGTTCTGGCCACTTTGTTCCCCGCCGAC
TTCACTCCTGAGGCTCATGTTGCTATGGACAAGTTCCTTTCAGCTCTGGCTCTGGCCATG
TCTGAGAAGTACAGA
[118] Danio_rerio_MN_Hba7
ATG---AGTCTTTCCGCAAAGGACAAAGCGAACGTCAAGGCCTTCTTTGACAAGGTT---
GCACCCAAAGCTGAGGAGATCGGCCGTGAGACTCTTTCCAGGACTTTGTTCGTTTACCCT
CAGACGAAGACATACTTCTCCCACTGGGCAGACCTAAGCCCCAACTCTCCTCAGGTGAAG
AAGCACGGAACCACGGTGATAAACGGAGTTTTGACTGCCGTCGGGCTGATGGATGACCTG
AAGGGAGGTTTGCTGACCCTCAGCGAGCTACACGCCTTCATGCTGCGCGTGGACCCCGCA
AACTTCAAGATCATCAACCACAATCTGCTGGTGTCTCTGGCCATGATGTTCCCAGACGAC
TTCACTCCTGAGGTGCATGTTTCCGTGGACAAGTTCCTGGCCCAGGTCAGCCTGGCCCTG
TCCGAGAAGTACCGC
[119] Danio_rerio_LA_Hba2
ATG------CTCTCGAGTGCCGAAAAAGAGCTGATTGCAGAAATATGGGACAAAATG---
ACTCCAGTGGCGGAAGAAATTGGATCTGAAGCTCTTTTAAGGATGTTCACAACGTTCCCC
AAAACAAAGACATACTTCTCTCATCTA---AATATCAGCGCTAATTCAGAGCATTTGCGC
TCCCACGGAAAGAAAATCGTCGAGGCTCTGGCCGAGGGTGCGAAGAACATAAGCACACTT
ACTACAACGTTGGCACCACTTAGCAGGTTCCATGCCTACCAACTGCGAATACATCCTACA
AACTTCAAGCTTTTCAATCATTGCATCCTTGTGACGTTAGCCTGCAGAATGGGTGACGAC
TTCACTCCAGTGGTGCACGCGGCGATAGACAAGTTTCTGTCGGCATTCTCAGCTGTTCTA
GCCGAGAAGTTCCGA
[120] Danio_rerio_LA_Hba1

ATG---AGTCTTTCTGCTAAAGACAAGGCCGCCGTGAGGGGCTTCTGGGCCAAGATT---
GCCCCAAAGGGAGAGCAAATTGGTAACGAGGCGTTTTCCAGATTGCTTTTGGTGTACCCT
CAGACCAAGACCTACTTCTCCCACTGGAACGATCTGGCCCCCGGCTCTCCCTCTGTGAAG
AAGCAGGGAAAGAAGATCGTCGGTGGACTCGGTCTGGCTGTTGATAAAATCGACGACCTT
TTCAACGGCCTGCTGAACCTCAGTGAATTGCACGCCTTTCAGCTGAGAGTCGACCCTGCT
AACTTCAAGCTCCTGTCTCACTGTCTGCTGGTGGTGTTCGCCATGCTCTTCCCTGATGAC
TTCACCGCTGAGGTCCATCTGGCCATCGACAAGTTCCTGGCAAGAGTGGCTTTGGCTCTG
TCTGACAAATATCGT
[121] Oryzias_latipes_MN_Hba1
ATG---AGTCTCACAGCAAAGGACAAGGACGCCGTCAGGACCTTCTGGGCTAAAATC---
TCAGGCAAGGCGGACGATGTGGGCACAGATGCTCTCTCCAGGATGCTGAAAGTTTACCCA
CAGACCAAGACCTACTTTTCCCACTGGACTGACCTGAGCCCAGGCTCTGCTTCAGTAAAG
AAACACGGGAAGACCGTCATTGGGGGAGTTGCTGATGCTGTGGCCAAAATCGATGCTCTA
TCATCAGGTCTTCTGAGCCTCAGTGAACTGCACGCCTTCACCCTGAGAGTGGACCCTGCC
AACTTTAAGATCCTCGGTCATTGCATCCTGGTAGTGATGGCCATGATGTTTCCCAATGAC
TTCACTCCTGAGGTGCATGTGGCCATGGACAAGTTCCTGGCTGCTTTGGCTCTGGCTCTG
TCTGAGAAGTACAGA
[122] Oryzias_latipes_MN_Hba2
ATG---AGTCTCAGCGCGAAGGACAAGGCAGCAGTTAAGGCCTTCTGGGCCAAAGTG---
TCTGGACAGGCAGATGCCATTGGCTCAGATGCTCTGTCTAGGATGCTGGTGGTCTACCCC
CAGACCAAGACCTACTTTGCCCACTGGAAGGACCTGAGCCCCGGCTCTGCCCCAGTAAAG
AAGCACGGGAAGACTGTGATGGGAGGAATTGCTGATGCTGTGGGCAAAATCGATGACATT
TCCTCAGGTCTGCTAAACCTCAGTGAACTTCATGCCTTCACCCTGAGAGTGGACCCCACC
AACTTTAAGATTCTTTCTCACAACATCCTTGTGGTTATGGCCATAATGTTCCCCCAGGAC
TTCACTCCTGAGGTCCATGTGGCTTTGGATAAGTTCCTGGCTGCAGTGTCCCTGGCTCTG
TCTGAGAAATATCGA
[123] Oryzias_latipes_MN_Hba3
ATG---AGTCTCACAGCTAAGGATAAGGAGGTCGTCAAGGCCTTCTGGACCAAATTG---
GCTCCCAAGGCAGATGATGTTGGAGCTGACGCTTTGTCCAGGATGTTGGTTGTGTACCCT
CAGACCAAGACCTACTTCGCCCACTGGAAGGACCTGAGCCCCGGCTCTGCCCCGGTGAAG
AAGCATGGGAAGACTATAATGGGAGGAGTTGCTGATGCTGTGGGCAAAATCGACGACCTG
ACAGCAGGTCTGCTGGACCTCAGTGAGCTGCACGCTTTCACCCTGAGAGTGGATCCAGCC
AACTTCAAGATTCTCTCTCACAACATCCTGGTTGTGTTGGCCATCGTCTTCCCCAACGAA
TTCACCCCTGAGGTCCATGTGGCCCTGGACAAGTTCCTGGGGGCTTTGTCCCGCGCCCTG
GCTGAGAAATACCGG
[124] Oryzias_latipes_MN_Hba4
ATGACCAGTCTCTCTGCTAAAGACAAGGATGTCGTCAAGGCATTCTGGGCCAAGATC---
TCTTCCAAGGCAACAGATATTGGAGCAGATGCTCTTGGCAGGATGCTGGTGGTCTACCCT
CAGACCAAGACCTACTTCGCCCACTGGAAGGACCTGAGCCCCGGCTCTGCCCCGGTGAAG
AAGCACGGACAGACTGTGATGGGAGGAGTTGCTGAAGCTGTGGGCAAAATCGACAATCTG
ACTGCTGGTCTCCTGAACCTCAGTGAGCTGCATGCTTTCACTCTGAGAGTGGATCCTGCC
AACTTCAAGATTCTCTCCCACAACATCCTGGTAGTGCTGGCCATCATGTTCCCCAATGAC
TTCACCCCTGAGGTGCATGTGGCTATGGACAAGTTCCTGGCTGCTTTGGCTCTGGCTCTG
GCTGAGAAGTACAGA
[125] Oryzias_latipes_MN_Hba5
ATGACCAGTCTCTCTGCTAAAGACAAGGATGTCGTCAAGGCATTCTGGGCCAAGATC---
TCTTCCAAGGCAACAGATATTGGAGCAGATGCTCTTGGCAGGATGCTGGTGGTCTACCCT
CAGACCAAGACCTACTTCGCCCACTGGAAGGACCTGAGCCCCGGCTCTGCCCCGGTGAAG
AAGCACGGACAGACTGTGATGGGAGGAGTTGCTGAAGCTGTGGGCAAAATCGACAATCTG
ACTGCTGGTCTCCTGAACCTCAGTGAGCTGCATGCTTTCACTCTGAGAGTGGATCCTGCC
AACTTCAAGATTCTCTCCCACAACATCCTGGTGGTGCTGGCCATCATGTTCCCCAATGAC
TTCACCCCTGAGGTGCATGTGGCCATGGACAAGTTCCTGGCTGCTTTGGCTCTGGCTCTG
GCTGAGAAGTACAGA
[126] Oryzias_latipes_LA_Hba2
ATG------TTGTCAAAGAAGGAGAAACAACTCATACGTGAAATATGGGAAAGACTG---
ACTCCAGTGGCTGAAGACATTGGAGCAGATGCTCTCCTTAGGATGTTTGCTTCATATCCA
GGCACTAAGACATATTTCTCTCATCTG---GACATCGGTCCTGGCTCCGCCCACCTATCC

TCCCACGGGAAGAAGATCGTCCTGGCCATCGCAGAGGGAGCCAAAGACATCAGCCAGCTG
ACCGTCACCCTGGCTCCCCTGCAGACTCTGCACGCCTACCAGCTCCGAATAGATCCCACC
AACTTCAAGCTGCTCTCATACTGTCTGCTTGTCACCCTGGCTTGCTACATGGGCGAAGGA
TTCACGCCGGAGGCCCACGCCGCCATAGACAAATTCCTGTCGGCTTTTTCGGCTGTCCTC
GCCGAAAAATACAGA
[127] Oryzias_latipes_LA_Hba1
ATG---AGTTTGACAGAGAAGGACAAAGCTGCCGTCAAGGCCCTTTGGGCCAAAATC---
TCCAAGTCCGCTGATGCGATTGGTGCTGACGCTCTGAGCAGGATGCTTCTTGTGTATCCC
CAAACCAAGACCTACTTCTCCCACTGGCCAGACACGAAGGCTGGTTCCGAGCCCGTGAAG
AAGCACGGCAAGAAGATCATGGGTGGGGTCGGTCTGGCCGTGTCCAAGATCGACGACCTG
GCCGCCGGCCTGCTGGAGCTCAGCGAGCTGCACGCCTTCAAGCTGCGGGTTGACCCGGCC
AACTTCAAGCTCCTGGCGCACTGCCTCCAGGTGGTCATCGCCAACATGTTCCCCAAGGAT
TTCACCCCGGAGGCCCACGTGGCTTGTGATAAGTTCCTGGCCAACGTGGCTTTGGCTCTT
TCTGAGAAATACCGC
[128] Gasterosteus_aculeatus_MN_Hba2
ATGACCACTCTCTCTACCAAGGACAAGGAGGTAGTCAAAGCCTTCTGGGCTAAAGTG---
TCTGGACAAGGGGAGGAAATCGGCACTGATGCCGTGGCCAGGATGCTGAGAGTTTACCCC
CAGACCAAGACTTACTTCTCCCACTGGAAGGACCGGAGCGCCTCCTCTCCCGATGTGCAG
AAGCACGGCCGGACTGTGATGGCTGGAGTTGGAGACGCTGTGGCCAAGATCGACGACCTG
AAAGGAGGTCTCTTGAACCTCAGTGAGCTGCACGCCTTCACTCTGCGTGTGGACCCTGCC
AACTTCAAGATCCTCTCCCACAACCTCCTGGTGGTCATGGCCACCATGTTGCCCAACGAC
TTCACCCCTGAGGTCCATGTGTCTATGGACAAGTTCCTGGCTGCTGTGGCTCTGGCTCTG
TCTGAGAAGTACCGA
[129] Gasterosteus_aculeatus_MN_Hba1
ATG---AGTCTCAACGCTAAGGACAAGGACGCGGTCAAGGCCTTCTGGGCGAAGGTG---
TCCGGCAAGGCTGGCGACATCGGATCCGATGCTCTGTCCAGGATGCTGACGGTGTACCCT
CAGACCAAGACCTACTTCGCCCACTGGAAGGACATGAGCGCCGGGTCCGATCCGGTGGTG
AAGCACGGAAAGGCCGTGATGGGTGGAGTTGCAGAGGCCATAAACAAAATCGACGACCTG
AATGCGGGTCTCCTGAACCTCAGTGAGCTGCACGCCTTCACTCTGCGTGTGGACCCTGCC
AACTTCAAGATTCTCTCCCACAACATCCTTGTGGTCATGGCCATCCTGTTCCCCAAAGAC
TTCACCCCCGAGGTCCACGTGGCCATGGATAAGTTCCTGGGCGCTTTGTCTCGTGCCCTG
GCCGAGAAATACAGA
[130] Gasterosteus_aculeatus_MN_Hba3
ATGACCACTCTCTCTACCAAGGACAAGCAGGTAGTCAAAGCCTTCTGGGCTAAAGTC---
TCTGGACAAGGGGAGGAAATCGGCACTGATGCCGTGGCCAGGATGCTGAGAGTTTACCCG
CAGACCAAGACTTACTTCTCCCACTGGAAGGACCGGAGCGCCTCCTCTCCCGATGTGCAG
AAGCACGGCCGGACTGTGATGGCTGGAGTTGGAGACGCTGTGGCCAAGATCGACGACCTG
AAAGGAGGTCTCTTGAACCTCAGTGAGCTGCACGCCTTCACTCTGCGTGTGGACCCTGCC
AACTTCAAGATCCTCTCCCACAACCTCCTGGTGGTCATGGCCACCATGTTGCCCAACGAC
TTCACCCCTGAGGTCCATGTGTCTATGGACAAGTTCCTGGCTGCTGTGGCTCTGGCTCTG
TCTGAGAAGTACCGA
[131] Gasterosteus_aculeatus_MN_Hba4
ATGACCACTCTCTCTACCAAGGACAAGAAGGTAGTCAAAGCCTTCTGGGCTAAAGTG---
TCTGGACAAGGGGAGGAAATCGGCACTGATGCCGTGGCCAGGATGCTGAGAGTTTACCCG
CAGACCAAGACTTACTTCTCCCACTGGAAGGACCAGAGCGCCTCCTCTCCCGATGTGAAG
AGGCACGGCCGGACTGTGATGGCTGGAGTTGGAGACGCTGTGGCCAAGATCGACGACCTG
AAAGGAGGTCTCTTGAACCTCAGTGAGCTGCACGCCTTCACTCTGCGTGTGGACCCTGCC
AACTTCAAGATCCTCTCCCACAACCTCCTGGTGGTCATGGCCACCATGTTCCCCAACGAC
TTCACCCCTGAGGTCCATGTGTCTATGGACAAGTTCCTGGCTGCTGTGGCTCTGGCTCTG
TCTGAGAAGTACCGA
[132] Gasterosteus_aculeatus_MN_Hba5
ATGACCACTCTCTCTACCAAGGACAAGGAGGTAGTCAAAGCCTTCTGGGCTAAAGTG---
TCTGGACAAGGGGAGGAAATCGGCACTGATGCCGTGGCCAGGATGCTGAGAGTTTACCCG
CAGACCAAGACTTACTTCTCCCACTGGAAGGACCGGAGCGCCTCCTCTCCCGATGTGCAG
AAGCACGGCCGGACTGTGATGGCTGGAGTTGGAGACGCTGTGGCCAAGATCGACGACCTG
AAAGGAGGTCTCTTGAACCTCAGTGAGCTGCACGCCTTCACTCTGCGTGTGGACCCTGCC
AACTTCAAGATCCTCTCCCACAACCTCCTGGTGGTCATGGCCACCATGTTCCCCAACGAC

TTCACCCCTGAGGTCCATGTGTCTATGGACAAGTTCCTGGCTGCTGTGGCTCTGGCTCTG
TCTGAGAAGTACCGA
[133] Gasterosteus_aculeatus_LA_Hba1
ATG---AGTTTGACTGACAAGGACAAGGCTACCGTCAAGGCCGTGTGGGCCAAGATC---
TCCAAGAGCGCGGACGCCATTGGGGCCGATGCGCTGAGCAGGATGCTCGTCGTCTACCCG
CAGACCAAGACCTACTTCTCCCACTGGCCCGACCTGGGCCCCAACTCCGCTTCCGTGAAG
AACCACGGAAAGAACGTGATGGGTGGAATCGCTTTGGCCGTGAGCAAGATCGACGACCTG
ACCGCCGGCCTGCTGGAGCTCAGCGAACAGCACGCCTTCCAGCTGAGGGTGGACCCGGCC
AACTTCAAGATCCTGTCTCACTGCATCCTCGTGGTGATCGCCGGCCTGTTCCCGAAGGAC
TTCACCCCCGAGGCCCACGTCGCCGTGGACAAGTTCTTCTGCGGCGTGTCTCTGGCCCTG
GCAGAGAGGTACCGC
[134] Gasterosteus_aculeatus_LA_Hba2
ATG------CTCTCCAAGAAGCAGAAAGACCTGATAGCGGAAATATGGGAAGGGTTG---
ATCTCTGTGGCAGACGATATTGGAGCAGACGCGCTGCTCAGGATGTTCGCCTCTTATCCC
GGTACCAAGACCTACTTCTCCCACCTG---GACATCAGCCACCGCTCCGCTCACCTGCTC
TCTCACGGGAAGAAGATCGTGCTGGCGATCGCGGAGGGGGCCAAAGACATCAGCCAGCTG
ACCGTCACGCTGGCTCCTCTGCAGACGCTGCACGCCTACCAGCTGCGGATAGACCCGACC
AACTTCAAGCTCTTCTCACACTGTATGCTCGTGAGCCTGGCGTGTCACATGGGAGAAGAC
TTCACCCCGGAAGCGCACGCGGCGATGGATAAATACCTGTCGGCCTTCGCTGCCGTGCTC
GCCGAGAAGTACAGA
[135] Oreochromis_niloticus_MN_Hba3
ATGACCAGTCTTTCTGCAAAGGACAAGAACACAGTCAAAGCCTTCTGGGCTAAAGTG---
GCTGGCAAGGAGGAACAAATCGGCTGTGATGCTGTCTCCAGGATGCTGACAGTGTACCCT
CAGACCAAGACTTACTTCTCCCACTGGAAGGACCTGAGCCCCCGCTCTGCCCCGGTGAAG
AAGCACGGAGCAACCGTGATGGCTGCAGTTACTGATGCTGTCAGCAAAATCGACGATCTG
ACCGGAGCTCTTCTGAGCCTCAGTGAGCTGCACGCCTTCACTCTGAGAGTGGACCCTGCT
AACTTCCAGGTTCTGTCTCACAACCTCCTCGTGGTCCTGTCCATTATGTTCCCCCAGGAC
TTCACCCCTGAGGTCCATGTGGCTATGGACAAGTTCCTGGCTGCTGTGGCTCTCGCCCTG
TCTGAGAAATACAGA
[136] Oreochromis_niloticus_MN_Hba4
ATGACCAGTCTTTCTGCTAAGGACAAGAACACAGTCAAAGCCTTTTGGGCTAAAGTG---
GCTGGCAAGGAGGAACAAATCGGCTGTGATGCCGTCTCCAGGATGCTGACAGTGTACCCT
CAGACCAAGACTTACTTCTCCCACTGGAAGGACCTGAGCCCCGGCTCTGCCCCGGTGAGG
AAACACGGAGCAACCGTGATGGCTGCAGTTACTGATGCTGTCAGCAAAATTGACGATCTC
ACCGGTGGTCTTCTGAGCCTCAGTGAGCTGCACGCCTTCACTCTGAGAGTGGACCCTGCT
AACTTCAAGGTTCTTGCTCACAACCTCCTCGTGGTCCTGGCCACCGTGTTTCCCAACGAT
TTCACTCCCGAGGTCCACGTGGCGATGGACAAGTTCCTGGCTGCCGTGGCTCATGCCTTG
TCCGAGAAATATCGA
[137] Oreochromis_niloticus_MN_Hba6
ATG---AGTCTTTCTGCAAAGGACAAGGAGACAGTCAAGGCCTTTTGGGCTAAAGTG---
TCGGGAAGGGCGGGAGACATTGGCAGTGACGCTGTTGCCAGGATGCTGACAGTGTACCCT
CAGACCAAGACTTACTTCTCCCACTGGAAGGACCTGAGCCCCGGCTCTGCCCCAGTGAGG
AAGCACGGAGGAATCGTGATAGCTTCAATAACCGATGCTGTGAACAAAATCGACGACTTG
GCAGAGAGTCTTCTTACCCTCAGTGAGCTGCATGCTTTCACTCTGCGAGTGGACCCTGCA
AACTTCCAGATTCTTGCTCAGAACATCCTGGTGGTTCTGGCCACCATGTTCCCCGTCGAC
TTCACCCCTGAGGTCCACATGGCTTTAGACAAGTTCCTGGGTGCCTTGGCTCGTGCCCTG
TCTGAGAAATACCGA
[138] Oreochromis_niloticus_MN_Hba7
ATG---AGTCTCTCTTCGAAAGACAAGACAGTGGTGAAAACCTTCTGGGGCAAAGTC---
GACTCAAAGAGCGCCGAGATTGGTGGAGAAGCTCTGGGCAGGATGCTCGTTGCGTATCCG
CAGACCAAGACCTACTTTTCTCACTGGGGCGACTTGACCCCTAGCTGCCCGCAGGTGAAG
AAGCATGGTGCAGTCATCATGGCAGCTGTGGGAAAAGCTGTCAAGAACATTGATGATCTT
ACCGGCCACCTTAGCAAGCTCAGTGAGCTGCATGCCACCCAGCTCCGCGTGGATCCTGCC
AACTTCCAGATCCTCGCTCACAGCATCATCTTGGTCCTGGGCATGTACTTCCCTGGGGAC
TTCACCCCGGAGGTTCACGTCTCCGTGGACAAGTTCTTCAATAACCTCGCTTGGGCTCTG
TCCGAGAGATACCGC
[139] Oreochromis_niloticus_MN_Hba8

ATGACCAGTCTTTCTGCAAAGGACAAGAACACAGTCAAAGCCTTCTGGGCTAAAGTG---
GCTGGCAAGGAGGAACAAATCGGCTGTGATGCTGTCTCCAGGATGCTGACAGTGTACCCT
CAGACCAAGACTTACTTCTCCCACTGGAAGGACCTGAGCCCCGGCTCTGCCCCGGTGAAG
AAGCACGGAGCAGCCGTGATGGCTGCAGTTACTGATGCTGTCAGCAAAATCGACGATCTG
ACCGGAGCTCTTCTGAGCCTCAGTGAGCTGCACGCCTTCACTCTGAGAGTGGACCCTGCT
AACTTCCAGGTTCTGTCTCACAACCTCCTCGTGGTCCTGTCTACCATGTTCCCCGACGAC
TTCACCCCTGAGGTCCATGTGGCTATGGACAAGTTCCTGGCTGCTGTGGCTCTCGCCCTG
TCTGAGAAATACAGA
[140] Oreochromis_niloticus_LA_Hba2
ATG------TTGTCCAAGAGAGGGAGAAAGACTTAATTAAGGAAATATGGGAAAGACTG---
ACTCCTGTGGCAGCAGAGATTGGCGCAGATGCGCTCCTTAGGATGTTTGCTTCTTATCCG
GGCACCAAGACATATTTTTCCCATCTA---GACATCAGTCCTAACTCATCCCACCTGCTT
GCTCATGGGAAGAAGATAGTCCTGGCTATAGCTGAGGGGGCCCAAGACATCAGTCAGCTA
ACTGTCACCCTGGCTCCCCTGCAGACTCTGCATGCCTACCAGCTCCGGATAGACCCCAGA
AACTTTAAGCTGCTGTCGCACAGTATGCTCGTCAGCCTTGCCTGTTACTTGGGGGATGAA
TTCACACCGGTGGCTCATGCCGCAATGGACAAGTACCTGTCAGCGTTTGCTGCTGTTCTT
GCTGAGAAATACAGA
[141] Oreochromis_niloticus_LA_Hba1
ATG---AGTCTGACTGAGAAAGACAAAGCTGCCGTCAAGGCACTCTGGGCCAAGATC---
TCCAAGTCTGTGGATGCTATTGGAGCCGAAGCTTTGGGCAGGATGCTCCTCGTGTATCCG
CAAACCAAGACTTACTTCTCCCACTGGCCGGACCTGACTCCCGGTTCTGCCCCCGTGGTG
AGTCACGGAAAGCAGATCATGGGTGGAGTCACTGAGGCCATGTCCAAGATTGACAACCTG
CGCGGCGGCCTGCTGGAACTGAGCGAGCTGCACGCCTTCAAGCTGAGGGTGGACCCATCT
AACTTCCAGATCTTGGCTCAGACCATTATGGTGGTGGTCGCCGCTATGTTCCCTAACGAT
TTCACCCCGGAAGCTCATGTCGCCTTCGACAAATTCTTGGCAGCCGTGGCCCTGGGTCTC
TCTGAGAGATACCGT
[142] Tetraodon_nigroviridis_LA_Hba1
ATG------ATCACCGAGAAGGAGAAAGAGCTGCTCAGAAAAGTGTGGAACAGTCTC---
ATCCCTGTGGCCGAGGACATCGGCTCGGATTCGCTCCTGAGGTTGTTCACCACAGTCCCA
GGCAGCAAGACGTACTTCTCCCACCTG---GACATCAGTCCTCGCTCCCCCCACATGCTC
TCCCATGGCAGGAAGATTGTTCTGGCAATCGCAGAAGGAGCCCAAGACATCAGCCAGCTG
GCCGTCAGCCTGGCTCCGCTGCAAACCCTGCACGCCTACCAGCTCCGGATCGACCCCACC
AACTTCAAGCTCCTGACACACTGTCTCCTGGTGTCGCTGGCCTGTCACATGGGCGACGAC
TTCACGCCGCAGGCTCATGCAGCCACAGACAAGTACCTCTCAGCTTTTGCAGCTGTGCTC
TCTGAGAAGTACAGA
[143] Tetraodon_nigroviridis_LA_Hba2
ATG---AGTTTGACTAAGACCGACAAGGCGGCCGTGAAGGCCCTGTGGAACAAACTG---
TCCAAGAGCGTCGATGTCATCGGGGCCGAAGCTTTTGGCAGGATGCTCCTCGTCTATCCT
CAAACCAAGATCTACTTCAGCAAATGGGGCGACATTAGTTTCGGCTCCAGCCAGGTGAAG
AACCACGGGAAGATAGTGATGGGTGGAATCGCCACTGCTGTGGCCAACATCGACGACCTG
ACATCTGGCCTGCAGAAGCTCAGCGAGGTTCACGCCTTTGATTTGAAAGTGGACCCGGCC
AACTTCAAGATCCTGGGTCAGTGCGTTGTTGTGGTCACTGCCATGTTGTTCCCCAAAGAC
TTCACTCCGGAGGTCCACGTCTCTTTCGATAAATTCCTGGCCGCGGTGGCGCTGGCTCTC
TCCGAGAAGTACCGC
[144] Tetraodon_nigroviridis_MN_Hba1
ATG---TCTCTTAACGCTAATGACAAGAAAGTGATCACCACCTTCTGGGGCAAAGCC---
TCGGCACAGGCTGACGCCATCGGATCGGATGCTCTGGGAAGGTTGCTGCTGGCCTACCCG
CAGACCAAGACTTACTTCTCCCACTGGAAAGACCTATCTCCTGGCTCTTCTGATGTCAAG
AGGCACGGAGCCCTCATCATGGGTGGCGTGACGGAGGCCGTCACTAAAATTGACAACCTG
TCCACCGGACTTCTTGAGCTCAGTGAGCTGCACGCCTTTACCCTGCGTGTGGACCCTGCC
AACTTCAAGCTCTTTGCCCACGCCATCATTGTGGCCATTGCCATAAGGTTCCCAGGGGAC
TTCACCCCTGAGGTCCACATGGCAATGGACAAGTTCTTTGCAGCTGTGGCCCGTGCCATG
TCCGAGAAATACCGA
[145] Tetraodon_nigroviridis_MN_Hba2
ATGACGAGTCTCAGCACTAAGGACAAGGAAACAGTCAGAGCCTTCTGGGCTAAAGTG---
GCTTCCAACAGGGAGGAGATTGGAGCCAGTGCTCTCTGCAGGTTGCTCTCCGTGTACCCG
CAGACCAAGACCTACTTCTCCCACTGGAAGGACCAGAGCCCCAACTCTGCCTCCGCCAAG

AAGCACGGAATCACCATCATGAACGCCGTTGGAGACGCTGTGTCCAAAATCGACGATCTG
AAAACGGGTCTCTTCAACCTGAGCGAGCTGCACGCCTTCACCCTGCGTGTGGACCCCGCC
AACTTCAAGCTCCTGGCTCAGTGCATGATGGTGGTCATCGCCATCATGTACCCTGCTGAC
TTCACCCCCGAGGTCCATGTGGCTATGGACAAGTTCCTGGCTTCTCTGGCTCTTGCCCTG
TCTGAGAAATACAGA
[146] Salmo_salar_chr3_MN_Hba0
ATG---AGTCTCTCAGCCAAGGACAAAGCCAACGTGAAGGCCATCTGGGGCAAAATC---
CTCCCTAAATCCGATGAGATTGGAGAACAGGCTCTTTCCAGGATGCTCGTCGTCTACCCC
CAGACCAAGGCCTACTTCTCCCACTGGGCTTCGGTGGCCCCCGGTTCCGCTCCCGTGAAG
AAGCACGGCATCACCATCATGAATCAGATCGATGAATGTGTTGGCAACTTGGACGACCTT
TTTGGTTTCTTGACCAAGCTCAGTGAACTGCACGCCACCAAGCTGAGGGTGGACCCCACC
AACTTCAAGATCCTGGCTCACAACCTGATTGTGGTCGTTGCCGCCTACTTCCCCGCAGAA
TTCACCCCCGAGATCCATTTGTCCGTGGACAAGTTCCTGCAGCAACTGGCTCTGGCCCTG
GCAGAGAAGTACCGC
[147] Salmo_salar_chr3_MN_Hba1
ATG---AGTCTGACAGCAAAGGACAAATCTGTGGTCAAGGCCTTCTGGGGCAAGATT---
AGTGGAAAGGCAGATGTTCTCGGCGCTGAGGCTTTGGGAAGGATGCTGACTGCTTACCCC
CAGACTAAGACCTACTTCTCCCACTGGGCTGACCTGAGCCCCGGCTCTGCCCCAGTCAAG
AAGCATGGAGGCGTCATCATGGGTGCAATTGGTAATGCTGTCGGACTGATGGACGACCTC
GTGGGGGGAATGAGTGGTCTCAGCGATCTGCACGCCTTCAAGCTGCGCGTTGACCCTGGA
AACTTCAAGATTCTGTCCCACAACATCCTTGTCACCCTGGCTATTCACTTCCCTGCGGAT
TTCACTCCCGAAGTGCACATTGCTGTGGATAAATTCCTTGCAGCTTTGTCCGCTGCCCTG
GCTGACAAATACAGA
[148] Salmo_salar_chr3_MN_Hba2
ATG---AGTCTCACAGCCAAGGACAAACAGATCGTGACAGCCTTTTTTGGAAAGGTG---
GCTGGCAAAGCAGAGGACATCGGAAATGAGGCTCTCTCTAGGACCCTGGTGGTGTACCCC
CAGACCAAGACCTACTTCTCCCACTGGACGGACCTGAGCCCCGGCTCTGCACCAGTCAAG
AAGCACGGTCTGACCGTCATGGGAGGCGTCCTGGATGCCGTGACCAAGATCGACGACCTG
GCCAGTGGTCTTCTGACCCTCAGCGAGCTTCACGCCTTCACGCTGCGTGTGGATCCCGCC
AACTTCAAGATCATCAACCACAACATCCTGGTGGTGCTGGCCATGATGTTCCCTGACGAC
TTTACCCCTGAGGTGCACTTGTCTGTGGACAAGTTCCTCGCCAAGTTGGCCCTGGCGCTT
TCTGAGAAGTATCGT
[149] Salmo_salar_chr3_MN_Hba3
ATG---AGTCTGACAGCAAAGGACAAATCTGTGGTCAAGGCCTTCTGGGGCAAGATT---
AGTGGAAAGGCAGATGTTCTCGGCGCTGAGGCTTTGGGAAGGATGCTGACTGCTTACCCC
CAGACTAAGACCTACTTCTCCCACTGGGCTGACCTGAGCCCCGGCTCTGCCCCAGTCAAG
AAGCATGGAGGCGTCATCATGGGTGCAATTGGTAATGCTGTCGGACTGATGGACGACCTC
GTGGGGGGAATGAGTGGTCTCAGCGATCTGCACGCCTTCAAGCTGCGCGTTGACCCTGGA
AACTTCAAGATTCTGTCCCACAACATCCTTGTCACCCTGGCTATTCACTTCCCTGCGGAT
TTCACTCCCGAAGTGCACATTGCTGTGGATAAATTCCTTGCAGCTTTGTCCGCTGCCCTG
GCTGACAAATACAGA
[150] Salmo_salar_chr3_MN_Hba4
ATG---AGTCTCTCAGCCAAGGACAAAGCCAACGTGAAGGCCATCTGGGGCAAAATC---
CTCCCTAAATCCGATGAGATTGGAGAACAGGCTCTTTCCAGGATGCTCGTCGTCTACCCC
CAGACCAAGGCCTACTTCTCCCACTGGGCTTCCGTGGCCCCCGGTTCCGCTCCAGTGAAG
AAGCACGGCATCACCATCATGAATCAGATCGATGAATGTGTTGGCAACTTGGACGACCTT
TTTGGTTTCTTGACCAAGCTCAGTGAACTGCACGCCACCAAGCTGAGGGTGGACCCCACC
AACTTCAAGATCCTGGCTCACAACCTGATTGTGGTCGTTGCCGCCTACTTCCCCGCAGAA
TTCACCCCCGAGATCCATTTGTCCGTGGACAAGTTCCTGCAGCAACTGGCTCTGGCCCTG
GCAGAGAAGTACCGC
[151] Salmo_salar_chr3_MN_Hba5
ATG---AGTCTGACAGCAAAGGACAAATCTGTGGTCAAGGCCTTCTGGGGCAAGATT---
AGTGGAAAGGCAGATGTTCTCGGCGCTGAGGCTTTGGGAAGGATGCTGACCGCCTACCCC
CAGACTAAGACCTACTTCTCCCACTGGGCTGACCTGAGCCCCGGCTCTGCCCCAGTCAAG
AAGCATGGAAGCACCATCATGGGTGCAATTGGTAATGCTGTCGGAGTGATCGACGACCTC
GTCGGAGGACTGAGTGCTCTGAGCGATCTGCACGCCTTTAAACTGCGCGTTGACCCTGGA
AACTTCAAGATTCTGTCCCACAACATCCTTGTCACCCTGGCTATTCACTTCCCTGCGGAT

TTCACTCCCGAAGTGCACATTGCTGTGGATAAATTCCTTGCAGCCTTGTCCGCTGCCCTG
GCTGACAAATACAGA
[152] Salmo_salar_chr3_MN_Hba6
ATG---AGTCTCACCGCTAAGGACAAGAAAATGGTCAGGGCCTTCTGGGCCAAGGTA---
TCCGGCAAGGCTGAGGACATCGGCTGCGATGCTCTGTCTCGGACGCTGGTTGTGTACCCC
CAGACCAAGACCTACTTCTCCCACTGGAAGGACTTGAGCCCCGGCTCTGCCCCAGTCAGG
AAGCATGGTGGGACCATCATGGGAGGCATCAGTTTAGCCGTGGCCAGCATCGACGACATC
AGCGCAGGTCTCCTCGCCCTCAGCGAGCTGCATGCCTTCAAGCTGCGTGTCGATCCCGCC
AACTTCAAGATCCTGTCCCACAACATCTTGGTGGTGCTGGCTATCTTGTTCCCCAATGAT
TTCAACCCCGAAGCACATGTGGCCATGGACAAGTTCCTGGCAGCGGTGGGCCGGGCTTTG
TCTGAGAAGTACCGA
[153] Salmo_salar_chr6_MN_Hba0
ATG---AGTCTCACAGCCAAGGACAAAAAGATCGTGAAAGCCTTTTTTGGAAAGGTG---
GCTGGCAAAGCAGAGGACATCGGAAATGAGGCTCTCTCTAGGACCCTGGTGGTGTACCCC
CAGACCAAGACCTACTTCTCCCACTGGACGGACCTGAGACCCGGCTCTGCTCCCGTCAAG
AAGCACGGTCTGACCGTCATGGGAGGCGTCCTTGATGCCGTGACCAAGATCGACGACCTG
ACCGGTGGTCTTCTGGCCCTCAGCGAGCTGCACGCCTTCACGCTGCGTGTGGATCCCGCC
AACTTCAAGATCATCAACCACAACATCCTGGTGGTGCTGGCCATGATGTTCCCTGACGAC
TTTACCCCTGAGGTGCACGTGTCTGTGGACAAGTTCCTCGCCAAGTTGGCCCTGGCGCTT
TCCGAGAAGTATCGT
[154] Salmo_salar_chr6_MN_Hba1
ATG---AGTCTCACCACTAAGGACAAGAAAATGGTCAAGGCCTTCTGGGCCAAGGTG---
TCCGGCAAGGCTGAGGACATCGGCTGCGATGCTCTGTCTAGGATGCTGGTTGTGTACCCC
CAGACCAAGACCTACTTCTCCCACTGGAAGGACCTGAGCCCCGGCTCTGCCCCAGTCAGG
AAGCACGGTGGGACCATCATGGGAGGCATCAGTTTAGCCGTGGCAAGCATCGACGACATC
AGCGCAGGTCTCCTCGCCCTCAGCGAGCTGCACGCCTTCCAGCTGCGTGTCGATCCCGCC
AACTTCAAGATCCTGTCCCACAACATCTTGGTGGTGCTGGCTGTCTTGTTCCCCAATGAA
TTCAACCCCGAAGCTCATGTGGCCATGGACAAGTTCCTGGCCGCGGTGGGCCGGGCTCTG
TCTGAGAAGTACCGA
[155] Salmo_salar_chr6_MN_Hba2
ATG---AGTCTCTCAGCCAAGGACAAAGCCAACGTGAAGGCCATCTGGGGCAAGATC---
CTCCCTAAAACCGATGAGATTGGAGAACAGGCTCTTTCCAGGATGCTTGTCGTCTACCCC
CAGACCAAGACCTACTTCTCCCACTGGACTTCCGTGGCCCCCGGTTCCGATCCAGTGAAG
AAGCACGGCATCACCATCATGAATCAGATCGATGACTGTGTTGGACACTTGGACGACCTT
TTTGGTTTCTTGACCAAGCTCAGTGAACTGCACGCCACCACGCTGAGGGTGGACCCCAAC
AACTTCAAGATCCTGGCTCACAACCTGATTGTGGTCTTTGCCGCCTACTTCCCTGCGGAA
TTTACCCCCGAGATCCACCTGTCCGTGGACAAGTTCCTGCAGCAAGTGGCTCTGGCCCTG
GCGGAGAAGTACCGC
[156] Salmo_salar_chr6_MN_Hba3
ATG---AGTCTGACAGCAAGGGACAAATCTGTGGTCAATGCCTTCTGGGGCAAGATT---
AAAGGAAAGGCAGATGTCGTCGGCGCTGAGGCTTTGGGAAGGATGCTGACTGCTTACCCC
CAGACTAAGACCTACTTCTCCCACTGGGCTGACCTGAGCCCCGGCTCTGCCCCAGTCAAG
AAGCATGGAGCCGTCATCATGGGTGCAATTGGTAATGCTGTCGGACTGATGGACAACCTC
GTGGGTGGACTGAGTGCTCTCAGCGATCTGCACGCCTTCAAGCTGCGCGTTGACCCTGGA
AACTTCAAGATTCTGTCCCACAACATCCTTGTGACCCTGGCTATTCACTTCCCTGCGGAT
TTCACTCCCGAAGTGCACATTGCTGTGGATAAATTCCTTGCAGCCTTGTCCGCTGCCCTG
GCTGACAAATACAGA
[157] Salmo_salar_chr6_MN_Hba4
ATG---AGTCTCTCAGCCAAGGACAAAGCCAACGTGAAGGCCATCTGGGGCAAGATC---
CTCCCTAAATCCGATGAGATTGGAGAACAGGCTCTTTCCAGGATGCTCGTCGTCTACCCC
CAGACCAAGGCCTACTTCTCCCACTGGGCTTCCGTGGCCCCCGGTTCCGCTCCAGTGAAG
AAGCACGGCATCACCATCATGAATCAGATCGATGAATGTGTTGGCAACTTGGACGACCTT
TTTGGTTTCTTGACCAAGCTCAGTGAACTGCACGCCACCAAGCTGAGGGTGGACCCCACC
AACTTCAAGATCCTGGCTCACAACCTGATTGTGGTCGTTGCCGCCTACTTCCCTGCCGAA
TTCACCCCCGAGATCCACCTGTCCGTGGACAAGTTCCTGCAGCAACTGGCTCTGGCCCTG
GCGGAGAAGTACCGC
[158] Salmo_salar_chr6_MN_Hba5

```
ATG---AGTCTGACAGCAAAGGACAAATCTGTGGTCAAGGCCTTCTGGGGCAAGATT---
AGTGGAAAGGCAGATGTCGTCGGCGCTGAGGCTTTGGGAAGGATGCTGACCGCCTACCCC
CAGACTAAGACCTACTTCTCCCACTGGGCTGACCTGAGCCCCGGCTCTGCCCCAGTCAAG
AAACATGGAAGCACCATCATGGGTGCAATTGGTAATGCTGTCGGACTGATCGACGACCTC
GTCGGAGGACTGAGTGCTCTCAGCGATTTGCACGCCTTCAAGCTCCGCGTTGACCCTGGA
AACTTCAAGATTCTGTCCCACAACATCCTTGTGACCCTGGCTATTCACTTCCCTGCGGAT
TTCACTCCCGAAGTGCACATTGCTGTGGATAAATTCCTTGCAGCCTTGTCCGCTGCCCTG
GCTGACAAATACAGA
[159] Xenopus_tropicalis_HbaZ
ATG---CTTTTCTCTGATGCCGAGAAGGCTGCAGTTGTGTCCCTCTGGGCAAAAGCA---
TCTGGCAATGTGAATGCCCTTGGAGCTGAAGCTTTGGAAAGGCTATTTCTGAGCTACCCT
CAGACCAAGACATACTTCAGCCACTTT---GACTTGGGTTCTGGATCTCATGATCTTCAA
GTTCATGGAGGAAAGGTCCTTGGTGCCATTGGAGAGGCCACCAAACATTTGGACAACCTG
GATGAAGCTCTGTCCAAGCTGAGTGACCTGCATGCTTATAACCTGAGAGTAGATCCAGGA
AATTTTAGGTTGCTGTCTCACACTATCCAAGTTACTCTGGCTGCCCACTTCCAGGCTGAC
TTTGATGCAACAGCCCAGGCTGCTTGGGACAAGTTCCTTGCCGCCATCTCTACTGTTCTT
ACCTCCAAGTACAGA
;
END
```

```
#NEXUS
BEGIN DATA;
  DIMENSIONS NTAX=131 NCHAR=447;
  FORMAT DATATYPE=DNA
  GAP=-
  ;
MATRIX
[1] Arctogadus_glacilis_B1
ATG---GTTGAATGGACAGCTGCCGAGCGGAGGCACGTCGAGGCGGTCTGGAGCAAGATC
GACATTGATGTCTGCGGACCACTCGCGTTGCAGAGGTGCCTGATTGTGTATCCGTGGACG
CAGCGCTACTTCGGTAGCTTTGGCGACCTGAGCACCGACGCCGCTATTATGGGAAACCCC
AAGGTGGCCCAGCACGGCGTCGTGGCCCTGACCGGCCTGAGGACGGCTCTGGACCACATG
GACGAAATCAAGTCCACCTACGCTGCCCTGAGCGTGCTGCACTCCGAGAAACTGCACGTC
GACCCCGACAACTTCCGACTGCTGTGTGAGTGCCTGACCATTGTCATCGCCGGGAAGATG
GGGAAGAAA---TTGAGCCCCGACATGCAGGCTGCGTGGCAGAAGTACCTGTGCGCGGTG
GTTTCCGCCCTGGGGAGACAGTACCAC
[2] Boreogadus_saida_B1
ATG---GTTGAATGGACAGCTACCGAACGGACCCACATCGAGGCGATCTGGAGCAAGATC
GACATTGATGTCTGCGGACCACTCGCGTTGCAGAGGTGCCTGATTGTGTATCCGTGGACG
CAGCGCTACTTCGGTAGCTTTGGCGACCTGAGCACCGACGCCGCTATTGTTGGAAACCCC
AAGGTGGCCAACCACGGCGTCGTGGCCCTGACCGGCCTGAGGACGGCTCTGGACCACATG
GACGACATCAAGGCCACCTACGCTACGCTGAGCGTGCTGCACTCCGAGAAACTGCACGTC
GACCCCGACAACTTCCGACTGCTGTGTGACTGCCTGACCATTGTCGTCGCCGGGAAGATG
GGGAAGACA---TTGAGGCCCGAGATGCAGGCTGCGTGGCAGAAGTACCTGTCCGCGGTG
GTTTCCGCCCTGGGGAGACAGTACCAC
[3] Trisopterus_minutus_B1
ATG---GTTGAATGGACAGATGACGAGCGGAAAGCGATCGAGAAGGTTTGGAAAAACATC
GACATTGATGTTTTCGGACCGCTCGCACTGCAGAGGTGCTTGATTGTGTATCCCTGGACT
CAGCGCTACTTCGGTAGCTTTGGCGACCTGACCACCGACGCCGCTATCATGAAGAACCCC
AAGGTGGCCAAGCACGGCGTGGTGGCCCTGAGCGGCCTGAAGACGGCTCTGGATAACATG
GACAACATCAAGAACACCTACGCTGCCCTGAGCGTGCTACACTCCGAGACACTGCACGTC
GACCCCGACAACTTCCGANNNNNNNNNNNNNNNNNNNNNNNNNNNNNNNNNCCGCGAAGATG
GGGAAGAAA---CTGTCCCCGGCGATGCAGGCCGCCTGGCAGAAGTACCTGTCCGCGGTG
GTCTCCGCTCTGGGGAGGCAGTACCAC
[4] Pollachius_virens_B1
ATG---GTTGAATGGACAGCTGCCGAGCGGAAGCACATCGAGACGGTCTGGAGCAAGATC
GACATTGATGTCTGCGGACCACTCGCGTTGCAGAGGTGCCTGATTGTGTATCCGTGGACG
CAGCGCTACTTCGGTAGCTTTGGCGACCTGAGCACCGATGCCGCTATCGTGGGAAACCCC
AAGGTGGCCAAGCACGGCGTCGTGGCCCTGACTGGCCTGAAGACGGCTCTGGACCACATG
GACGACATCAAGTCCACCTACGCTGCCCTGAGCGTGCTGCACTCCGAGAAGCTGCACGTC
GACCCCGACAACTTCCGANNNNNNNNNNNNNNNNNNNNNNNNNNNNNNNNNNCGCGAAGATG
GGGAAGAAA---ATGAGCCCCGAGATGCAGGCTGCGTGGCAGAAGTACCTGTGCGCGGTG
GTTTCCGCCCTGGGGAGACAGTACCAC
[5] Melanogrammus_aeglefinus_B1
ATG---GTTGAATGGACAGATGCCGAGCGGAGGCACATCGAGACGATCTGGGGCAAGATC
GACATTGATGTCTGCGGACCACTCGCGTTGCAGAGGTGCCTGATTGTGTATCCGTGGACG
CAGCGCTACTTCGGTAGCTTTGGCGACCTGAGCACCGACGCCGCTATCGTGGGAAACCCC
AAGGTGGCCAAGCACGGCGTCGTGGCCCTGACCGGCCTGAAGACGGCTCTGGACCACATG
GACGACATCAAGGCCACCTACGCTGCCCTGAGCGTGCTGCACTCCGAGAAACTGCACGTC
GACCCCGACAACTTCCGACTGCTGTGTGACTGCCTGACGATTGTCGTCGCCGGGAAGATG
GGGAAGAGA---CTGAGCCCCGAGATGCAGGCTGCGTGGCAGAAGTACCTCTCCGCGGTG
GTTTCCGCCCTGGGGAGACAGTACCAC
[6] Merlangius_merlangus_B1
ATG---GTTGAATGGACAGCTGCCGAGCGGAGCCACATCGAGACGATCTGGAGCAAGATC
GACATTGATGTCTTCGGACCGCTCGCGTTGCAGAGGTGCCTGATTGTGTATCCGTGGACG
CAGCGCTACTTCGGTAGCTTTGGGGACCTGAGCACCGACGCCGCTATTGTCGGAAACCCC
AAGGTGGCCAGGCACGGCGTCGTGGCCCTGACCGGCCTGAGGACGGCTCTGGACCACATG
```

```
GACGAGATCAAGGCCACCTACGCTGCCCTGAGCGTGCTGCACTCCGAGAAACTGCACGTC
GACCCCGACAACTTCCGANNNNTGTGTGACTGCCTGACCATTGTCGTCGCCGGGAAGATG
GGGAAGAAA---CTGAGCCCCGAAATGCAGTCTGCGTGGCAGAAGTACCTCTCCGCGGTG
GTTGCCGCCCTGGGGAGACAGTACCAC
[7] Theragra_chalcogramma_B1
ATG---GTTGAATGGACAGCTGCCGAGCGGAGACACATCGAGGCGGTCTGGAGCAAGATC
GACATTGATGTCTGCGGACCACTCGCGTTGCAGAGATGCCTGATTGTGTATCCGTGGACG
CAGCGCTACTTCGGTGGCTTTGGCGACTTGAGCACCGACACCGCTATTATGGGAAACCCC
AAGGTGGCCAAGCACGGCGTCGTGGCCCTGACCGGCCTGAGGACGGCTCTGGACCACATG
GACGAAATCAAGACCACCTACGCTGCCCTGAGCGTGCTGCACTCCGAGAAACTGCACGTC
GACCCCGACAACTTCCGACTGCTGGGTNNNNNNNNNNNNNNNNNNNNNNNNNGGAAGATG
GGGAAGAAA---ATGAGCCCAGAAATGCAGGCTGCGTGGCAGAAGTACCTGTCCGCGGTG
GTTTCCGCCCTGGGGAGACAGTACCAC
[8] Gadiculus_argentus_B1
ATG---GTTGAATGGACAGATGCCGAGAAGAGCGCCATCACGAAGGTGTGGACTTACATC
GACATCGATGTAATCGGACCTGCTGCGCTGCGGAGGTGCATGATTGTGTATCCCTGGACG
CAGCGCTACTTTGGCAGCTTCGGCGACCTGAGCACCGACGCCGCTATCATGGGTAACGCC
AGGGTGGCCCAGCACGGCGTCACGGCCCTGACCGGCCTGAAGACTGCTCTGGACAACATG
GACAACATCAAGGGCACCTACGCTGCCCTGAGCGTGCTGCACTCCGAGAAACTGCACGTC
GACCCTGACAACTTCCGACTGCTGTGTGACTGCCTGACCATTGTCGTCGCCGGGAAGATG
GGGAAGAAA---ATGACCCCTGAGATGCAGGCTGCTTGGCAGAAGTACCTGACCGCAGTG
GTCTACGCCCTGGGGAGGCAGTACCAC
[9] Phycis_phycis_B1
ATG---GTTGAGTGGACAGATAGCGAGCGCAAGGCCATCGACAAGGTCTGGAGCTACATC
GACATTAATGTCATCGGGCCGCTCATGTTGNNNNNNTGCCTTATTGTGTACCCCTGGACG
CAGCGGTACTTCGGTGCCTTTGGCGACCTGAGCACCGATCAGGCCATCGCGAACAACCCC
AAGGTGGCACAGCACGGCATCGTCGCCCTGACCGGCTTGAAGGCAGCTCTGGATAGCATG
GACGACATCAAAAACGCGTACGCTCCTCTGAGCGTTCTGCATTCCGAGAAACTGCACGTT
GATCCCGACAACTTCAGACTGCTGTGTGACTGCCTGACCGTGATCGTCGCTGGGAAGATG
GGTCTCAAA---TGCACCCCGGCCATGCAAGCTGCGTGGCAGAAGTACCTGACCGCCATC
GTCGCGGCCCTAGGGAGGCAGTACCAC
[10] Molva_molva_B1
ATG---GTTGCATGGACAGATTTTGAGCGGAAGGCCATCGACACGATCTGGAGCAACATC
GACATTGAGGTTATTGGACCACTCGCGTTGCAAAGGTGCTTAATTGTGTATCCCTGGACG
CAGCGCTACTTCGGTAGCTTTGGCGACCTGAGCACCGACAATGCTATTATGAACAACCCC
AGGGTTGCCAAGCACGGCGTCACCGCCCTGACCGGCCTAAGGACGGCTCTGGACAACATG
GACAACATCAAGAGCACCTACGCTGCTCTTAGCGTGCTGCACTCCGAGAAACTGCACGTT
GATCCCGACAACTTCAGACTGCTGTGTGACTGCCTGACCATTGTCGTCGCCGGGAAGATG
GGACGCAAA---ATCACCCCGGAGATGCAGGCGGCGTGGCAGAAGTATCTGTCCGTGGTT
GTCTCTGCCCTCGGGAGGCAGTACCAC
[11] Lota_lota_B1
ATG---GTTGCATGGACAGATTACGAGAGGAAGGCCATCGACCAGATCTGGAGCACCATC
GACATTGATGTCATCGGACCACTAGCGTTGCAAAGGTGCTTAATTGTGTATCCCTGGACG
CAGCGCTACTTCGGTAGCTTTGGCGACCTGAGCACCGACAATGCTATTTTGAGCAACCCC
AAGGTGGCCAAGCACGGCGTCGTCGCCCTGACCGGCCTAAAGATGGCTCTGGACAACATG
GACAACATCAAGAACGCTTACTCTGCTCTGAGCGTGCTGCACTCCGAGAAACTGCACGTC
GACCCCGACAACTTCAGACTGCTGTGTGACTGCCTGACCATAGTCGTCGCCGGGAAGATG
GGACGCAAA---TGCACCCCGGAGATGCAAGCCGCGTGGCAGAAGTACCTGTGCGTGGTT
GTCTCTGCCCTCGGTAGGCAGTACCAC
[12] Brosme_brosme_B1
ATG---GTTGCATGGACAGAAACCGAGCGTAAGACAATCGAGCTGATCTGGAACAACATC
GACATTAATGTCATCGGACCACTTGCGTTGCAAAGGTGCTTAATTGTGTATCCCTGGACG
CAGCGCTACTTCGGTAGCTTTGGCGACCTGAGCACCGACAACGCTATTATGAACAACCCC
AAGGTGGCCAAGCACGGCGTAACCGCCCTGACCGGCCTAAAGATGGCTCTGGACAACATG
GACAACATCAAGAACACCTACTCTGCTCTGAGCGTGCTGCACTCCGAGACACTGCACGTT
GACCCGGACAACTTCAGANTGCTGTGTGACTGCCTGACCATTGTCGTCGTCGGGAAGATG
GGACGCAGA---GTCACCCCGGAAATGCAGGCGACGTGGCAGAAGTACCTGTCCGTGGTT
```

GTCAACGCCCTCGGGAGGCAGTACCAC
[13] Merluccius_merluccius_B1
ATG---GTTGAGTGGACAGACAGCGAGAGGAGCTTGATCCAGAGGGTCTGGGACGCAATC
GACGTTAATGTCGTCGGGCCACTCATCCTGAGAAGGTGCTTAATTGTGTATCCCTGGACG
CAGCGCTACTTCGGTAAATTCGGAGACCTGACCACCGAGGTCTCCATCATGAACAACGAG
TCCGTGGCCAAACACGGCGTGACAGTCCTGAACGGCCTGAAGATGGGGCTGGACAACATG
GACACCATGAAGAAGACCTACTCTGACCTGAGCGTGCTGCACTCCGAGAAGCTGCACGTG
GACCCCGACAACTTCAAGNNGCTGTGTGACTGCCTGACCATAGTCGTCGCCGGGAAGATG
GGCCTGAAG---TTCAGCCCGGCAATGCAAGCCGCGTGGCAGAAGTACTTGAACGTGGTG
CTCTCGGCCCTCACGAAACAGTACCAC
[14] Merluccius_capensis_B1
ATG---GTTGTGTGGACAGACAGCGAGAAGAGCTTGATCCAGAGGGTCTGGGACGCAATC
GACATTAATGTCGTCGGGCCACTCATCCTGAGAAGGNNNTTAGTTGTGTATCCCTGGACG
CAGCGCTACTTCGGTAAATTCGGAGACCTGACCACCGAGGTCTCCATCATGAACAACGAG
GCCGTGGCCAAACACGGCGTGATAGTCATGAACGGCCTGAAGATGGGGCTGGACAACATG
GACACCATGAAGAAGACCTACTCTGACCTGAGCGTGCTGCACTCCGAGAAGCTGCACGTG
GACCCCGACAACTTCAAGCTGCTGTGTGACTGCCTGACCATAGTCGTCGCCGGGAAGATG
GGCCTGAAG---TTCAGCCCGGAAATGCAAGCCGCGTGGCAGAAGTACTTGAACGTGGTG
CTCTCGGCCCTCACGAAACAGTACCAC
[15] Merluccius_polli_B1
ATG---GTTGAGTGGACAGAGAGCGAGAAGACCTTGATCCAGAGGGTCTGGGATTCAATC
GACGTTAATGTCGTCGGGCCACTCGTCCTGAGAAGGTGCTTAATTGTGAATCCCTGGACG
CAGCGCTACTTCGGTAAATTCGGAGACCTGACCACCGAACTCTCCATCATGACCAACGAG
GACGTGGCCAAACACGGCGTGGTGGTCATGAAGGGCATGAAGATGGGGCTGGACACCATG
GACACCATGAAGAAGACCTACTCTGACCTGAGCGTGCTGCACTCCGAGAAGCTGCACGTG
GACCCCGACAACTTCAAGCTGCTGTGTGACTGCCTGACCATAGTCGTCGCCGGGAAGATG
GGCCTGAAG---TTCAGCCCGGCAATGCAAGCCGCGTGGCAGAAGTACTTGAACGTGGTG
ATCTCGGCCCTCACGAAACAGTACCAC
[16] Melanonus_zugmayeri_B1
ATG---GTTGACTGGACAGATGAAGAGGTGAGGCTCGTTACCAAGGTCTGGGACAACATC
GACATTGGTGTCATCGGACCACTCATCTTGCAAAGGTGCTTAATTGTGTATCCCTGGACG
CAGCGCTACTTCGGTAAGTTCGGCGACCTGACCACTACCGAGTCTATCATGACCAACCCC
AACGTGGCCAAGCACGGCATCACCGCCCTGAACGGCTTGAAGATGGCGTTGGACAACATG
AACACCATCAAGAAGACTTACGCTGCTCTGAGCGTGTTGCACTCCGAGACACTGCACGTG
GACCCCGACAACTTCAGGCTGCTGAGTGACTGCCTGACCATCACCATCGCCGGGAAGATG
GGACAGAGA---TTCACACCAGACATGCAGGCGGCGTGGCAGAAGTACTTGGCTGTGGTG
GTCGCAGCCCTCTCAAAGCAGTACCAC
[17] Macrourus_berglax_B1
ATG---GTTGAGTGGACAGACTTCGAGCGCAAGGCCATCGAGAAGGTCTGGAGCAGCGTC
AACATTGATGTCATCGGACCCCTCAGCTTGACAAGGTGCCTAATTGTGTATCCCTGGACG
CAGCGCTACTTCGGTAACTTCGGCGACCTGAGCACCCATGCAGCGATTCTGGGCAACCCC
AAGGTTGCCAAGCATGGTGTGGTTGTCCTGGCTGGGCTGAAGAAGGGGATGGAGGACATG
GACCACATCAAGGACACATACACGGCCCTGAGCATCTTGCACTCCGAGACCCTGCACGTG
GACCCCGACAACTTTAGGCTGCTGTGCGACTGTCTGACCGTCGTGGTGGCCGGGAAGATG
GGCCAGAAG---TTCAACCCTGAGATGCAGGCCGCCTGGCAGAAGTACCTGAACACGGTG
GTGGCGGCCCTGGGGAAGCAGTACCAC
[18] Malacocephalus_occidentalis_B1
ATG---GTTGAGTGGACAGAAAGCGAGAGGACGTCTATTGAGAAGGTCTGGCAGAACATC
GACATACAGGACATTGGACCACTCATCTTGACAAGGTGCTTACTTGTGTATCCCTGGACG
AAGCGTTACTTCGGTAACTTTGGCGACCTGACCACCACTGACGCCATCAAGAACAACCCC
AAGGTGTCCCAACACGGCGTGATGGTCTTGACCGCCCTGAAGTTGGCGCTGGACAACATG
GACAGCATCAAGCAAACATACTCAGATCTGAGCAAGTTGCACTCTGAACAGCTTCACGTC
GACCCGGACAACNNNNNNCTGCTGTGTGACTGCGCGACCATCATTGTAGCTGGGAAGATG
GGACGCAAG---TTCACCCCGGCCATGCAGGGTGCGTGGCAGAAGTACCTGAGCGCGGTG
GTTTCAGCCATGTCGAAGCAGTACCAC
[19] Bathygadus_melanobranchus_B1
ATG---GTTGAGTGGACAGACGCCGAGCGGAAGGCCATCCAGAAGGTCTGGGACAACATC

AACGTTGATGTCATCGGACCAGTCACCTTGTCAAGGTGCCTAATTGTGTATCCGTGGACG
CAGCGTTACTTCGGGAGCTTCGGCGACCTGAGCACCTATGACGCTATTCAGAGCAACCCC
ATGGTGGCGAAACACGGCGTGGTCGCCCTGACCGGTCTGAAGAGGGCCTTGGACAACATG
GACGACATCAAGAACACTTACGCTTCTCTGAGCGTGTTGCACTCCGAGACTCTGCACGTG
GACCCCGACAACTTCAGGCTGCTGTGTGACTGCCTGACCATCACGATCGCTGGGAAGATG
GGACGCAAA---TTCACCTATGAGATGCAAGCGGCGTGGCAGAAATACTTGGCTGTGGTT
GTCTCTGCCCTGGGGAAGCAGTACCAC
[20] Muraenolepis_marmoratus_B1
ATG---GTTGAATGGACTGAAAGCGAGGTGAAGGCTCTCGAAAGGATCTGGAGGAACGTC
GATGTTGACGTCATCGGACCACTCGCCTTGACCAGGTGCTTGATCGTGTATCCCTGGACG
CAGCGCTACTTCGGTAGCTTCGGCGACCTGAGCACGGAGAAGTCCATCCAGACCAACCCC
AAGGTGGCCAATCACGGCGTGGTGGTGCTGGCCAGCATGAAGAGCGCCATGGAGAGCCTG
ACCGACATGAAGAACAAGTTCTCTGCGCTCAGTACGCTGCACTCTGAGAAGCTGCACGTG
GACCCCGACAACTTCAGGCTGCTGTCCGACTGCTTGACCATCACCGTCGCCGGGAAGATG
GGAAGCAAG---TTCACCGCTGAGATGCACGCCACTTGGCAGAAGTTCCTGTCCGTGCTG
GTGTCGGCCCTGGGCCGGCAGTACCAC
[21] Bregmaceros_cantori_B1
ATG---GTTCAGTGGACGGATGACGAGACTAAGGCTATCCTGATGATCTGGGGCAGCGTT
GACGTCAATGAGATTGGACCCGCTGCCCTCAGGAGGTGCCTGCTCGTCTACCCATGGACG
CAGCGTTACTTCGGCAAGTTCGGAGACATCGCCACCCCCACGGCCATCATGAACAACGCC
GGCGTGGCGCAGCACGGCATCACTGTGATGAATGGCCTGAAGCTCGCCCTGGACAACATG
ACCAACATCAAGGCCGTGTACAAAGATCTGAGCAAGCTGCACTCCGAGAAGCTCCAAGTC
GACCCCGACAACTTCCGGTTGCTGGGTGAGTGCCTGACCATCCTGATCGGACAGAAGCTC
GGCAAGCAGGTGTTCAACCCCCAGGTGCACGCGGCGTGGACCAAGTACCTGGCTGTGTGC
GTGTCCGCCCTGTCCCGCCAGTACCAG
[22] Laemonema_laureysi_B1
ATG---GTTGAATGGAAAGATAGCGAGTTGAAGGCCATCGAGACGGTCTGGAACAGCATC
GATGTTAGTGTCGTCGGACCACTCGCCTTGTCAAGGTGCTTAATTGTGTATCCCTGGACG
CAGCGCTACTTCGGGAGCTTCGGCGACCTGAGCACCAGAGAGGCCATTATGAACAACCCC
AAGGTGGCCCGGCACGGAGTCGTCGCCTTGAACGGACTCAAGATGGCTATGGATAACATG
AGCAGCATCAAGGCCGCCTACGCGGCTCTGAGCGTGCTGCACTCTGAGACTCTGCATGTT
GACCCCGACAACTTCAGGCTGCTGTGTGACTGCCTGACCATAGTCGTTGCCGGAAAGATG
CAACAGAAA---TTCACTGCAATGATGCAAGCGGCGTGGCAGAAGTACTTGAGCGTGGTT
GTCTCAGCCCTCACTAAGCAGTACCAC
[23] Percopsis_transmontana_B2
ATG---GTCGTGTGGACAGATGAAGAGAGAAACTACATCACCGGCATCTTCTCCAACCTG
GACTATGAAGACATAGGTCCTAAGGCTCTTTGCAGGTGTCTGATCGTGTACCCTTGGACC
CAGCGCTACTTTGGCAGCTTTGGCAACCTCTACAACGCCGAGGCCATCCTGTCAAATCCG
CTTATCGCAGCTCACGGCGTCAAGGTGTTGCACGGTCTGGACAGAGCTGTGAAGAACATG
GATGACATCAAGAATACCTACGCCTCCCTGAGCGTGCTGCACTCTGAGAAGCTCCACGTG
GACCCCGATAACTTCAAGTTGCTGGGTGACTGCCTCACCGTCGTCGTCGCTGCCAAATTA
GGGAAAGAT---TTCACCATCCAGCTGCAGGCGGCCTGGCAGAAGTTCTTGGCCGTCGTG
GTGTCTGCTTTGTGCAAGCAATACCAC
[24] Gadus_morhua_B1_B1
ATG---GTTGAATGGACAGCTGCTGAGCGGAGGCACGTCGAGGCGGTCTGGAGCAAGATC
GACATTGATGTCTGCGGACCACTCGCGTTGCAGAGATGCCTGATTGTGTATCCGTGGACG
CAGCGCTACTTCGGTAGCTTTGGCGACCTGAGCACCGACGCCGCTATTGTGGGAAACCCC
AAGGTGGCTGCGCACGGCGTCGTGGCCCTGACCGGCCTGAGGACGGCTCTGGACCACATG
GACGAAATCAAGTCCACCTACGCTGCCCTGAGCGTGCTGCACTCCGAGAAACTGCACGTC
GACCCCGACAACTTCCGACTGCTGTGTGAGTGCCTGACCATTGTCGTCGCCGGGAAGATG
GGAAGAAA---TTGAGCCCGGAGATGCAGGCTGCGTGGCAGAAGTACCTGTGCGCGGTG
GTTTCCGCCCTGGGGAGACAGTACCAC
[25] Thrachyrincus_scabrus_B1
ATG---GTTGAGTGGACAGAAAGCGAGATTACGGCCATCACAAAGGTCTGGAGAAACATC
GACATTGACGTCATCGGACCACTCGTCTTGACTAGGTGCCTAATTGTGTATCCCTGGACG
CAGCGCTACTTCGGTAGCTTCGGCGACCTGAGCACAGAGGAGTCTATTAAGTCCAACTCC
AAGGTGGCCAAGCACGGCATAACCGTCATGAACAGCCTGAAGACGGCGATGGACAACTTG

GACAAGATCAAGAGCACCTACGCTAGCCTGAGCACGCTGCACTCCGAGACACTGCACGTC
GACCCGGACAACTTCAGGCTGTTGTGTGACTGCCTGACCATAATCATCGCCGGGAAAATG
GGACGCAGA---TTCACCCCGGACATGCAAGCGGCGTGGCAGAAGTACCTGGCCGTGATC
GTCTCCGCCCTCAGCAGACAGTACCAC
[26] Gadus_morhua_B2_B2
ATG---GTTGAGTGGACTGATGAAGAGCGCACCATCATTAATGACATCTTCTCCACCTTG
GACTACGAAGAGATCGGTCGCAAGTCTCTGTGCAGGTGTCTGATCGTGTACCCCTGGACC
CAGAGGTACTTCGGCGCCTTCGGCAACCTGTACAATGCAGAGACCATCATGGCCAACCCC
CTGATCGCAGCCCACGGAACCAAGATCCTGCACGGTCTGGACCGGGCCCTGAAGAACATG
GACGACATCAAGAACACCTACGCCGAGCTGTCTCTGCTCCACTCTGACAAGCTGCACGTG
GATCCCGACAACTTCAGGCTGCTGGCTGACTGCTTGACCGTCGTCATCGCCGCCAAGATG
GGCACCAAA---TTCACCGTGGAGACCCAGGTGGCGTGGCAGAAGTTCCTGTCTGTCGTC
GTCTCCGCTCTGGGCAGACAGTACCAC
[27] Arctogadus_glacilis_B2
ATG---GTTGAGTGGACAGATAGTGAGCGCGCCATCATTAATGACATCTTCGCCACCTTG
GACTACGAAGAGATCGGTCGCAAGTCTCTGACCAGGTGTCTGATCGTGTACCCCTGGACC
CAGAGGTACTTCGGCGCCTTCGGCAACCTGTACAATGCAGCGACCATCATGGCCAACCCC
CTGATCGCAGCCCACGGCACCAAGATCCTGCACGGTCTGGACCGGGCCCTGAAGAACATG
GACGACATCAAGAACACCTACGCCGAGCTGTCTCTGCTCCACTCTGACAAGCTGCACGTG
GATCCCGACAACTTCAGGCTGTTGGCCGACTGCCTGACCGTCGTCATCGCCGCCAAGATG
GGCGCCGCC---TTCACCGTGGATACCCAGGTGGCGTGGCAGAAGTTCCTGTCTGTCGTC
GTCTCCGCTCTGGGCAGACAGTACCAC
[28] Boreogadus_saida_B2
ATG---GTTGAGTGGACAGATAGTGAGCGCGCCATCATTAATAGCATCTTCTCCAACTTG
GACTACGAAGAGATCGGTCGCAAGTCTCTGTGCAGGTGTCTGATCGTGTACCCCTGGACC
CAGAGGTACTTCGGCGCCTTCGGCAACCTGTACAATGCAGAGACCATCATGGCCAACCCC
CTGATCGCCGCCCATGGCACCAAGATCCTGCACGGTCTGGACCGGGCCCTGAAGAACATG
GACGACATCAAGAACACCTACGCCGAGCTGTCTCTGCTCCACTCTGACAAGCTGCACGTG
GATCCCGACAACTTCAGGCTGCTGGCCGACTGCCTGACCGTCGTCATCGCCGCCAAGATG
GGCACCGCC---TTCACCGTGGAGACCCAGGTGGCGTGGCAGAAGTTCCTGTCTGTCGTC
GTCTCCGCTCTGGGCAGACAGTACCAC
[29] Trisopterus_minutus_B2
ATG---GTTCAGTGGACTGATGAAGAGCGCGGCATCATCAATGGCATCTTCGCCAACTTG
GACTACGAAGAGATTGGCCGCAAGTCTCTTTGCAGGTGTCTGATCGTGTACCCCTGGACC
CAGAGGTACTTCGGCGGCTTCGGCAACCTGTACAACGCAGAGACCATCCTGTGCAACCCC
CTGATCGCCGCCCACGGCACCAAGATCCTGCACGGTCTGGACCGGGCCCTGAAGAACATG
GACGACATCAAGAACACCTACGCCGAGCTGTCTCTGCTCCACTCCGACAAGCTTCACGTG
GACCCCGACAACTTCAGGCTGCTGGCCGACTGCTTGACCGTCGTCATCGCCGCCAAAATG
GGCAACGCC---TTCACCGTGGAGACCCAGGTGGCGTGGCAGAAGTTCCTGTCTGTCGTC
GTCTCCGCTCTGGGCAGACAGTACCAC
[30] Pollachius_virens_B2
ATG---GTTGAGTGGACTGATGATGAGCGCTCCATCATTACTAGCATCTTCTCCAACTTG
GACTACGAAGAGATCGGTCGCAAGTCTCTGTGCAGGTGTCTGATCGTGTACCCCTGGACC
CAGAGGTACTTCGGCGGCTTCGGCAACCTGTACAACGCAGAGACCATCCTGTGCAACCCC
CTGATCGCCGCCCACGGCACCAAGATCCTGCACGGTCTGGACCGGGCCCTGAAGAACATG
GACGACATCAAGAACACCTACGCCGAGCTCTCTCAGCTCCACTCTGACAAGCTGCACGTG
GATCCCGACAACTTCAGGCTGCTGGCCGACTGCCTGACCGTCGTCATCGCCGCCAAGATG
GGCACCGCC---TTCACCGTGGAGACCCAGGTGGCGTGGCAGAAGTTCCTGGCTGTCGTC
GTCTCCGCTCTGGGCAGACAGTACCAC
[31] Melanogrammus_aeglefinus_B2
ATG---GTTGAGTGGACTGATGATGAGCGCACCATCATTAATGGCATCTTCTCCAACTTG
GACTACGAAGAGATCGGTCGCAAGTCTCTGTGCAGGTGTCTGATCGTGTACCCCTGGACC
CAGAGGTACTTTGGTGCCTTCGGCAACCTGTACAACGCAGAGACCATCCTGGGCAACCCC
CTGATCGCCGCCCACGGCACCAAGATCCTGCACGGTCTGGACCGGGCCTTGAAGAACATG
GACGACATCAAGAACACCTACGCCGAGCTGTCTCTGCTCCACTCTGACAAGCTGCACGTC
GATCCCGACAACTTCAGGCTGCTGGCCGACTGCCTGACCGTCGTCATCGCCGCCAAGATG
GGCACCGCC---TTCACCGTGGAGACCCAGGTGGCGTGGCAGAAGTTCCTGGCTGTCGTC

GTCTCCGCTCTGGGCAGACAGTACCAC
[32] Merlangius_merlangus_B2
ATG---GTTGAGTGGACTGACGATGAGCGCGCTATCATTAATAGCATCTTCGCCAACTTG
GACTACGAAGAGATCGGTCGCAAGTCTCTGTGCAGGTGTCTGATCGTGTACCCCTGGACC
CAGAGGTACTTCGGTGCCTTCGGCAACCTGTACAACGCCGAGACCATTATGGGCAACCCC
CTGATCGCCGCCCACGGCACCAAGATCTTGCACGGTCTGGACCGGGCCCTGAAGAACATG
GACGACATCAAGAACACCTACGCCGAGCTGTCTCAGCTCCACTCTGACAAGCTGCACGTG
GATCCCGACAACTTCAGGCTGCTGGCCGACTGCCTGACCGTCGTCATCGCCGCCAAGATG
GGCACCGCC---TTCACCGTGGAGACCCAGGTGGCGTGGCAGAAGTTCCTGGCTGTCGTC
GTCTCCGCTCTGGGCAGACAGTACCAC
[33] Theragra_chalcogramma_B2
ATG---GTTGAGTGGACTGATGAAGAGCGCACCATCATTAATGACATCTTCTCCACCTTG
GACTACGAAGAGATCGGTCGCAAGTCTCTGTGCAGGTGTCTGATCGTGTACCCCTGGACC
CAGAGGTACTTCGGCGCCTTCGGCAACCTGTACAATGCCGAGACCATCATGGCCAACCCC
CTGATTGCAGCCCACGGCACCAAGATCCTGCACGGTCTGGACCGGGCCCTGAAGAACATG
GACGACATCAAGAACACCTACGCCGAGCTGTCTCTGCTCCACTCTGACAAGCTGCACGTG
GATCCCGACAACTTCAGGCTGCTGGCCGACTGCCTGACCGTCGTCATCGCCGCCAAGATG
GGCACCAAA---TTCACCGTGGACATCCAGGTGGCGTGGCAGAAGTTCCTGTCTGTCGTC
GTCTCCGCTCTGGGCAGACAGTACCAC
[34] Gadiculus_argentus_B2
ATG---GTTGAGTGGACTGATGACGAGCGCACCATCATTAACAACATCTTCTCCACCTTG
GACTATGAAGAGATTGGCCGCAAGTCTCTCACCAGGTGTCTGATCGTGTACCCCTGGACC
CAGAGGTACTTCGGTGCCTTCGGCAACCTGTACAACGCCGAGACCATCCTGGCCAACCCC
CTGATCGCCGCCCATGGCACCAGGATCCTGCACGGTCTGGACCGGGCCCTGAAGAACATG
GACGACATCAAGAACACCTACTCTGAGCTGTCCCAGCTCCACTCCGAGAAGCTTCACGTG
GACCCCGACAACTTCAGGCTGCTGGCCGACTGCATGACCGTCGTCATCGCCGCCAAGATG
GGCGTCGCC---TTCACCGTGGACACCCAGGTGGCGTGGCAGAAGTTCCTGTCTGTCGTC
GTCTCCGCTCTGGGCAGACAGTACCAC
[35] Phycis_phycis_B2
ATG---GTTCAGTGGACTGACGATGAGCGCAGCATCATTAATGGCATCTTCGCCAACCTG
GACTACGAAGATATCGGCCGCAAGTCTCTTTGCAGGTGTCTGATCGTGTACCCATGGACC
CAGAGGTATTTCGGAGGATTTGGTAACCTGTACAACGCAGAGACCATCCTGTGCAACCCC
CTGATCGCCGCCCACGGCACCAAGATCCTGCACGGTCTTGACCGCGCCCTGAAGAACATG
GACGACATCAAGAACACCTACGCCGAGCTGAGTCAGCTCCACTCCGACAGGCTGCACGTG
GACCCCGACAACTTCAGGCTGCTGGCTGACTGCCTGACCGTCGTCATCGCCGCCAAGATG
GGAACCGCC---TTCACCGTGGACATCCAGGTCGCATGGCAGAAGTTCCTGGCCGTCGTT
GTCTCTGCTCTGGGCAGACAGTACCAT
[36] Molva_molva_B2
ATG---GTTCAGTGGACTGAGGATGAGCGCAGCATCATTTCTAGCATCTTTGCCAACTTG
GACTATGAAGAGGTCGGCCGCAAGTCTCTTTGCAGGTGTCTGATCGTGTACCCATGGACC
CAGAGGTATTTCGGTGGATTTGGCAACCTGTACAACGCAGAGACCATCTTGTGCAACCCC
CTGATCGCCGCCCATGGCACCAAGATCCTGCACGGTCTGGACCGGGCCCTGAAGAACATG
GACGACATCAAGAACACCTACACTGAGCTGAGTCAGCTCCACTCCGACAGGTTGCACGTG
GACCCCGACAACTTCAGGCTTCTGGCTGACTGCCTGACCGTCGTCATTGCCGCCAAGATG
GGAAGCGCC---TTCACCGTGGATACCCAGGTCGCATGGCAGAAGTTCCTGGCTGTCGTT
GTCTCTGCTCTGGGAAGACAGTACCAT
[37] Lota_lota_B2
ATG---GTTCAGTGGACTGATGAAGAGCGCAGCATCATTACTGGCATCTTTTCCAACTTG
GACTATGAAGAGATCGGCCGCAAGACTCTTTGCAGGTGTCTGATCGTGTACCCATGGACC
CAGAGGTATTTCGGTGGATTTGGCAACCTGTACAACGCAGAGACCATCATGAGCAACCCC
CTGATCGCCGCCCATGGCACCAAGATCCTGCACGGTCTGGACCGCGCCCTGAAGAACATG
GACGACATCAAGAACACCTACGCTGAGCTGAGTCAGCTCCACTCCGAGAAGCTGCACGTG
GACCCCGACAACTTCAGGCTGCTGGCTGACTGCCTCACCGTCGTCATTGCCGCCAAGATG
GGAAACGCC---TTCACCGTGGATGCCCAGGTCGCATGGCAGAAGTTCCTGGCTGTCGTT
GTCTCTGCTCTGGGCAGACAGTACCAT
[38] Brosme_brosme_B2
ATG---GTTCAGTGGACTGATGAAGAGCGCAGCATCATTTCTGGCATCTTTGCTAACTTG

GACTATGAAGAGATCGGCCGCAAGTCCCTTTGCAGGTGTCTGATCGTGTACCCATGGACC
CAGAGATACTTCGGTGGATTTGGCAACCTGTACAACGCAGAGACCATCTTGTGCAACCCC
CTGATCGCCGCCCATGGCACCAAGATCCTGCACGGTCTGGACCGGGCCCTGAAGAACATG
GACGACATCAAGAACACCTACACTGAGCTGAGTCAACTCCACTCCGATAAACTGCACGTG
GACCCCGACAACTTCAGGCTGCTGGCCGATTGCCTGACCGTCGTCGTTGCCGCCAAGATG
GGAAACGCC---TTCACCGTGGAAACCCAGGTCGCATGGCAGAAGTTCCTGGCTGTCGTT
GTCTCTGCTCTGGGCAGACAGTACCAT
[39] Merluccius_merluccius_B2
ATG---GTGAAGTGGACAGACGAAGAGCGCAGCATCATCAATGGCATCTTCAGCACATTG
GACTACGAGGATATCGGCAAGAAGGCCCTCTGCAGGTGTCTGATCGTGTACCCATGGACC
CAGCGTTACTTCGGCGCCTTCGGCAACCTGTACAACGCCGAGACCATCATGTGCAACCCC
CTGATCGCCGCCCACGGCACCAAGATCCTGCACGGTCTGGACCGCGCCCTGAAGAACATG
GACGACATCAAGAACACATACGCAGAGCTGAGCGTGCTGCACTCTGACACCCTCCACGTG
GACCCAGACAACTTCAGGCTGTTCTCCGACTGCCTGACCGTCGTCATCGCCGCCAAGCTC
GGCAACTCC---TTCACCGTGGACACCCAGGTGGCCTGGCAGAAGTTCCTGGCCGTGGTG
GTCTCTGCTCTGGGCAGACAGTACCAT
[40] Merluccius_capensis_B2
ATG---GTGAAGTGGACAGATGAAGAGCGCAGCATCATCAATAGCATCTTCAGCACCTTG
GACTACGAAGATATCGGCAAGAGGGCCCTCTGCAGGTGTCTGATCGTGTACCCATGGACC
CAGCGTTACTTCGGCGCCTTCGGCAACCTGTACAACGCCGAGACCATCATGTCCAACCCC
CTGATCGCCGCCCACGGCACCAAGATCGTGCACGGTCTGGACCGCGCCCTGAAGAACATG
GACGACATCAAGACCACATACGCAGAGCTGAGCGTGCTGCACTCTGACACCCTCCACGTG
GACCCAGACAACTTCAGGCTGTTCTCCGACTGCCTGACCGTCGTCATCGCCGCCAAGCTC
GGCAACTCC---TTCACCGTGGAAACCCAGGTGGCCTGGCAGAAGTTCCTGGCCGTGGTG
GTCTCTGCTCTGGGCAGACAGTACCAT
[41] Merluccius_polli_B2
ATG---GTGAAGTGGACAGACGAAGAGCGCACCATCATTAATGGCATCTTCAGCACCTTG
GACTACGAAGATATCGGCAAGAGGGCCCTCTGCAGGTGTCTGATCGTGTACCCATGGACC
CAGCGTTACTTCGGCGCCTTCGGCAACCTGTACAACGCCGAGACCATCATGTGCAACCCC
CTGATCGCCGCCCACGGCACCAAGATCCTGCACGGTCTGGACCGCGCCCTGAAGAACATG
GACGACATCAAGAACACATACGCAGAGCTGAGCGTGCTGCACTCCGACACCCTCCACGTG
GACCCAGACAACTTCAGGCTGTTTTCCGACTGCCTGACCGTTGTGATCGCCGCCAAGCTC
GGCAACGCC---TTCACCGTGGAAACCCAGGTGGCCTGGCAGAAGTTCCTGGCCGTGGTG
GTCTCTGCTCTGGGCAGACAGTACCAT
[42] Melanonus_zugmayeri_B2
ATG---GTTGAGTGGACAGACGATGAGCGCGCCATCATTACTGGCATCTTCAACACCATG
GATTACGATGAGATCGGCAAAAAGTCTCTTTGCAGGTGTCTGATCGTGTACCCATGGACC
CAGAGGTATTTCGGCGCATACGGCAACCTGTACAACGCCGAGACCATCATGAACAACCCG
CTCATCGCCGCCCACGGCACCAAGATCCTGCACGGTCTGGACCGGGCCCTGAAGAACATG
GACAACATCAAGGAAACCTACTCAGAGCTGAGCCAGCTGCACTCTGAGAAGCTCCACGTG
GATCCCGACAACTTCAGGCTGCTGGCTGACTGCATGACCGTGGTCATCGCCGCCAAGATG
GGAACCGGC---TTCACCATTGACACGCAGGCCGCATGGCAGAAGTTCGTGCACGTCGTC
GTCTCTGCTCTGGGCAAACAGTACCAT
[43] Macrourus_berglax_B2
ATG---GTTGAGTGGACCGATGACGAGCGCAAGATCATCAATGGCATTTTCGCCAACTTG
GACTACGATGATATTGGCCACAATTCTCTGTGCAGGTGCCTGATTGTGTACCCATGGACC
CAGAGGTACTTCGGTGCCTATGGAAACCTGTACAACGCAGAAACCATTATGGGCAACCCC
CTGATCGCCAAACTCGGCAGCAAGATTGTGCATGGTCTGGATCGTGCCCTGAAGAACATG
GACGACATCAAGAACACCTACACAGAGCTGAGTGTCCTTCACTCCGAAAAGCTGCAAGTG
GACCCTGACAACTTCAGGCTGCTGGCCGACTGCATGACGGTGGTCATCGCCGCCAAGCTG
GGAACTGGC---TCCACCATCGACACCCAGGTGGCCTGGCAGAAGTTCCTGTCTGTGGTG
GTCTCTGCTCTGGAAAGACAGTACAAC
[44] Malacocephalus_occidentalis_B2
ATG---GTTGAGTGGACCGACAGCGAGCGCACCATCATTAACAGCATCTTCGCCAACTTG
GACTATGAAGAAGTTGGCCGCAAGTCTCTTTGCAGGTGTCTGATTGTGTACCCATGGACC
CAGAGATATTTTGGTGCATTTGGCAACCTGTACAACGCAGAAACCATCATGGCCAACCCA
TTGATCGCTAAACACGGCACCACCATCCTGCACGGTCTAGACCGGGCACTGAAGAACATG

GACAACATNNNNNNNNNNNNNNNNNNNNNNNNNNNNNNNNNNNNNNNNNNNNNNNNNNNN
NNNNNNNNNNNNNNNNNNCTGCTGGCTGACTGCATGACTGTTGTCATTGCCACCAAGTTA
GGAAGCGCC---TTCGACATTGACACCCAGGCTGCATGGCAGAAGTTCGTGATGGTCGTT
GTCTCTGCTCTGGGCAGACAGTACCAT
[45] Bathygadus_melanobranchus_B2
ATG---GTTGAGTGGACTGATGAAGAGCGCAAAATCATCACTGACATCTTCTCCACCTTG
GATTACGACGAGGTCGGCCGCAGGTCGCTTTGCAGGTGTCTGATCGTGTACCCATGGACC
CAGAGGTATTTCGGTGGGTATGGAAACCTGTACAACGCAGAGACCATCATGGCCAACCCC
ATGATCGCCAAACATGGCACCAAGATCCTGCACGGTCTGGACCGGGCCCTGAAGAACATG
GACGACATCAAGAACACCTACTCAGAGCTGAGTGTCCTGCACTCCGAAACGCTTCATGTG
GACCCCGACAACTTCAGGCTGCTGACTGACTGCATGACCGTCGTCATTGCCGCCAAGTTG
GGAAACAGC---TTCACCGCGGATATCCAGGCGGCGTGGCAGAAGTTCATGACTGTCGTC
GTCTCTGCTCTGGGCAGACAGTACCAT
[46] Muraenolepis_marmoratus_B2
ATG---GTTCAGTGGACTGATGAAGAGCGCGCCATCATCACCGACATCTTCGCCAACTTG
GACTACGTAGAGGTCGGTCGCAAGTCTCTGTGCAGGTGTCTGATCGTGTTCCCATGGACC
CAGAGGTACTTCGGCGCATACGGTAACCTGTACAACGCAGAGACCATCCTGTGCAACCCT
CTGATCGCTGCCCATGGCACCAAGATCCTGCACGGTCTGGACCGGGCCTTGAAGAACATG
GACAACATCAAGGAGATCTACACCGAGCTGAGCCAGCTTCACTCCGAGAAGCTGCACGTG
GACCCCGACAACTTCAGGCTGCTGGCCGACTGCATGACGGTGGTCATCGCCGCCCAGATG
GGAGCTGCC---TTCACCGTGGAGACCCAGGTCGCATGGCAGAAGTTCCTGTGTGTGGTC
GTCTCTGCTCTGGGCAGACAGTACCAC
[47] Bregmaceros_cantori_B2
ATG---GTTGTCTGGTCTGATCAGGAGCGTTCCATCATCACCAGCATTTTCTCTGGCCTC
GACTACGAGGAGATTGGCAGGAAGTCGCTGTGCAGGTGTCTGATCGTGTTCCCCTGGACG
CAGCGTTACTTCGGCGGCTTCGGCAACTTGTACAACGCTGACACCATCTTGAGCAACCCA
CTGATCGCCGCCCATGGCACCAAGATCCTGCACGGCCTGGACAGGGCGCTGAAGAACATG
GACGACATCAAGAACACGTACACCGAGCTGAGCAAGCTGCACTCCGAGAAACTGCACGTG
GATCCCGACAACTTCAGGTTGATCTCTGAGTGCATGACCGTGGTGATCGCCGCCAAGATG
GGCAACGCC---TTCACCGTGGAGGTCCAGATGGCCTGGCAGAAGTTCCTCGCTGTGGTG
GTCTCTGCTCTGAGCAAGCAGTACCAC
[48] Mora_moro_B2
ATG---GTTAAGTGGAGTGAAGACGAGCGCACCATCATCAATGGCATCTTCGCCAACTTG
GACTACGAAGATATCGGCCGCAAGTCTCTTTGCAGGTGTCTGATCGTGTACCCATGGACC
CAGAGGTATTTCGGCGCCTACGGAAACCTGTACAACGCCGAGACCATCATGTCCAACCCC
CTGATCGCCGCCCACGGCACCAAGATCCTGCACGGTCTGGACCGGGCCCTGAAGAACATG
GACGACATCAAGAACACCTACACAGAGCTGAGCCAGCTCCACTCCGAGAAGCTGCACGTG
GACCCCGACAACTTCAGGCTGCTGGCTGACTGCATGACCGTCGTCATTGCCGCCAAGCTG
GGACCCGGC---TTCACCGTGGAGACCCAGGTGGCGTGGCAGAAGTTCCTGTCCGTGGTC
GTCTCCGCTCTGGGCAGGCAGTACCAT
[49] Laemonema_laureysi_B2
ATG---GTTCAGTGGACTGACGACGAGCGCAGCATCATCAATGGCATCTTTGCCAGCTTG
GACTACGAAGATATTGGCCGCAAGTCTCTTTGCAGGTGTCTGATCGTGTACCCATGGACC
CAGAGGTATTTCGGTGCCTATGGAAACCTGTACAACGCCGAAACCATCATGTCCAACCCT
CTGATCGCCGCCCATGGCACCAAGATCCTGCACGGTCTGGACCGGGCCCTGAAAAACATG
GACGACATCAAGAACACCTACACAGAGCTGAGTCAGCTCCACTCCGAAAAGCTGCACGTG
GACCCCGACAACTTCAGGCTGCTGGCTGACTGCATGACCGTCGTCATTGCCGCCAAGTTA
GGAACCGGC---TTCACCGTGGAGACCCAGGTCGCATGGCAGAAGTTCCTGTCCGTCGTC
GTCTCCGCTCTGGGCAGACAGTACCAT
[50] Percopsis_transmontana_B1
ATG---GTTGAATGGACACCTGCTGAGCTTAAGGCCATCCAACTGATCTGGGGCACTCTC
AAGGTCGATGTAGACGGACCCCTGGCCTTGCAAAGGTGTCTCATAGTGTACCCGTGGACT
CAACGATACTTTAAGTCTTTTGGAGACTTGAGTGACCCCAAGTCCATAATAAGTAACCCC
AAAGTGGCCAAGCACGGCATCACCGTGATCAAAGGCATGGAGAAAGCTTTACAGAACATG
AACAACATCAAGAAGACGTTCGCCCCTTTGAGTACTCTGCATTCCGAAAAGCTCCAAGTA
GATCCCGATAACTTCAAGGTGCTTGGTGATTGCTTGACCATTGTTGTAGCAGCGAGAATG
GGAATTGGC---TTTACCCCAGACGTCCATTGTGCATGGCAGAAGTTCCTGTCCGTGGTG

ATCTCCGCACTGGGAAGAGAATATTAT
[51] Percopsis_transmontana_B5_2
ATG---GTTGAATGGACAGACTTCGAGCGCGCTACCATCACAGACATCATGTCTAAGATG
GACTATGAGGTTATCGGACCGGCAGCTCTTTCCAGGTGCCTGATCGTCTACCCCTGGACT
CAGAGGTATTTCGGGAACTTTGGAAACCTCTACAACGCCGAAGCCATCATTGCGAACCCA
ATGGTGGCTGCCCACGGGATCACAGTGCTGCACGGACTGGACCGGGCTGTGAAGAACCTA
GACAACATCAAGGCCACTTATGCTGAGCTTAGCGTGCTGCACTCCGAGAAGCTGCACGTG
GACCCTGACAACTTCCGGCTGCTGGCTGACTGTCTGACCATCGTCATTGCTTCCAAAATG
GGATCTGGA---TTCACAGCTGACGTCCAGGCCGCCTTGCAGAAGTTCCTGGCCGTCGTG
GTGTCCTCCCTGGGAAGACAGTACCAC
[52] Thrachyrincus_scabrus_B2
ATG---GTTGAGTGGACTGATGAAGAGCGTTCCATCATTGACGGCATCTTCGCCAACTTG
GACTACGAAGAAGTCGGCAAGAAGTCTCTTTGCAGGTGTCTCATCGTCTACCCATGGACC
CAGAGGTACTTCGGCGCATATGGCAACCTGTACAACGCAGAGACCATCCTGTGCAACCCC
CTGATCGCTGCCCACGGCACCAAGATCCTGCACGGTCTGGACCGCGCCCTGAAGAACATG
GACAACATCAAGGAGACCTACTCTGAACTGAGTCATCTGCACTCCGAGAAGCTGCACGTG
GACCCCGACAACTTCAGGCTGCTGGGTGACTGCATGACCGTCGTCATTGCCGCCAAGATG
GGAGCCGCC---TTCACAATTGATACTCAGTTCGCGTTCCAGAAGTTCCTCAGCGTGGTC
ATCTCCGCTCTGGGCAGACAGTACCAC
[53] Gadus_morhua_B3_B3
ATG---GTTGAGTGGACAGATAGTGAGCGCGCCATCATTAATGGCATCTTCTCCAACTTG
GACTACGAAGAGATCGGCCGCAAGTCTCTGTGCAGGTGTCTGATCGTGTACCCCTGGACC
CAGAGGTACTTCGGCGGCTTCGGCAACCTGTACAACGCAGAGACCATCCTGTGCAACCCC
CTGATCGCCGCCCATGGCACCAAGATCCTGCACGGTCTGGACCGGGCCCTGAAGAACATG
GACGACATCAAGAACACCTACGCCGAGCTGTCTCTGCTCCACTCTGACAAGCTGCACGTG
GATCCCGACAACTTCAGGCTGCTGGCCGACTGCCTGACCGTCGTCATCGCCGCCAAGATG
GGCCCCGCC---TTCACCGTGGATACCCAGGTGGCGTGGCAGAAGTTCCTGTCTGTCGTC
GTCTCCGCTCTGGGCAGACAGTACCAC
[54] Arctogadus_glacilis_B3
ATG---GTTGAGTGGACAGATAGTGAGCGCGCCATCATTAATAGCATCTTCTCCAACTTG
GACTACGAAGAGATCGGCCGCAAGTCTCTGTGCAGGTGTCTGATCGTGTACCCCTGGACC
CAGAGGTACTTCGGCGGCTTCGGCAACCTGTACAACGCAGAGACCATCCTGTGCAACCCC
CTGATCGCCGCCCATGGCACCAAGATCCTGCACGGTCTGGACCGGGCCCTGAAGAACATG
GACGACATCAAGAACACCTACGCCGAGCTGTCTCTGCTCCACTCTGACAAGCTGCACGTG
GATCCCGACAACTTCAGGCTGCTGGCCGACTGCCTGACCGTCGTCATCGCCGCCAAGATG
GGCGCCGCC---TTCACCGTGGAGACCCAGGTGGCGTGGCAGAAGTTCCTGTCTGTCGTC
GTCTCCGCTCTGGGCAGACAGTACCAC
[55] Boreogadus_saida_B3
ATG---GTTGAGTGGACAGATAGTGAGCGCGCCATCATTACTAGCATCTTCTCCAACTTG
GACTACGAAGAGATCGGCCGCAAGTCTCTGTGCAGGTGTCTGATCGTGTACCCCTGGACC
CAGAGGTACTTCGGCGGCTTCGGCAACCTGTACAACGCAGAGACCATCCTGTGCAACCCC
CTGATCGCCGCCCATGGCACCAAGATCCTGCACGGTCTGGACCGGGCCCTGAAGAACATG
GACGATATCAAGAACACCTACGCCGAGCTGTCTCTGCTCCACTCTGACAAGCTGCACGTG
GATCCCGACAACTTCAGGCTGCTGGCCGACTGCCTGACCGTCGTCATCGCCGCCAAGATG
GGCACCGCC---TTCACCGTGGAGACCCAGGTGGCGTGGCAGAAGTTCCTGTCTGTCGTC
GTCTCCGCTCTGGGCAGACAGTACCAC
[56] Trisopterus_minutus_B3
ATG---GTTCAGTGGACAGATGAAGAGCGCGGCATCATCAATGGCATCTTCGCCAACTTG
GACTACGAAGAGATCGGCCGCAAGTCTCTTTGCAGGTGTCTGATCGTGTACCCCTGGACC
CAGAGGTACTTCGGTGCCTTCGGCAACCTGTACAACGCAGAGACCATCCTGGCCAACCCC
CTGATCGCCGCCCACGGCACCAAGATCCTGCACGGTCTGGACCGGGCCCTGAAGAACATG
GACGACATCAAGAACACCTACGCCGAGCTGTCTCTGCTCCACTCCGACGTGCTTCACGTG
GACCCCGACAACTTCAGGCTGCTGGCCGACTGCTTGACCGTCGTCATCGCCGCCAAGATG
GGCAACGCC---TTCACCGTGGAGACCCAGGTGGCGTGGCAGAAGTTCCTGTCTGTCGTC
GTGTCCGCTCTGGGCAGACAGTACCAC
[57] Pollachius_virens_B3
ATG---GTTGAGTGGACTGATGATGAGCGCTCCATCATTAATGGCATCTTCTCCAACTTG

GACTACGAAGAGATCGGTCGCAAGTCTCTGTGCAGGTGTCTGATCGTGTACCCCTGGACC
CAGAGGTACTTCGGCGGCTTCGGCAACCTGTACAACGCAGAGACCATCCTGTGCAACCCC
CTGATCGCCGCCCACGGCACCAAGATCCTGCACGGTCTGGACCGGGCCCTGAAGAACATG
GACGACATCAAGAACACCTACGCCGAGCTGTCTCAGCTCCACTCTGACAAGCTGCACGTG
GATCCCGACAACTTCAGGCTGCTGGCCGACTGCCTGACCGTCGTCATCGCCGCCAAGATG
GGCACCGCC---TTCACCGTGGAGACCCAGGTGGCGTGGCAGAAGTTCCTGGCTGTCGTC
GTCTCTGCTCTGGGCAGACAGTACCAC
[58] Melanogrammus_aeglefinus_B3
ATG---GTTGAGTGGACAGATGATGAGCGCGCCATCATTAATGGCATCTTCTCCAACTTG
GACTACGAAGAGATCGGCCGGAAGTCTCTGTGCAGGTGTCTGATCGTGTACCCCTGGACC
CAGAGGTACTTCGGCGGCTTCGGCAACCTGTACAACGCAGAGACCATCCTGTGCAACCCC
CTGATCGCCGCCCACGGCACCAAGATCCTGCACGGTCTGGACCGGGCCTTGAAGAACATG
GACGACATCAAGAACACCTACGCCGAGCTGTCTCTGCTCCACTCTGACAAGCTGCACGTC
GATCCCGACAACTTCAGGCTGCTGGCCGACTGCTTGACCGTCGTCATCGCCGCCAAGATG
GGCGCCGCC---TTCACCGTGGACACCCAGGTGGCGTGGCAGAAGTTCCTGGCTGTCGTC
GTCTCCGCTCTGGGAAGACAGTACCAC
[59] Merlangius_merlangus_B3
ATG---GTTGAGTGGACTGATGATGAGCGCGCCATCATTAATAGCATCTTCTCCACCTTG
GACTACGAAGAGATCGGCCGGAAGTCTCTGTGCAGGTGTCTGATCGTGTACCCCTGGACC
CAGAGGTACTTCGGCGGCTTCGGCAACCTGTACAACGCAGAGACCATCCTGTGCAACCCC
CTGATCGCCGCCCACGGCACCAAGATCCTGCACGGTCTGGACCGGGCCCTGAAGAACATG
GACGACATCAAGAACACCTACGCCGAGCTGTCTCAGCTCCACTCTGACAAGCTGCACGTG
GATCCCGACAACTTCAGGCTGCTGGCCGACTGCCTGACCGTCGTCATCGCCGCCAAGATG
GGCACCGCC---TTCACCGTGGAGACCCAGGTGGCGTGGCAGAAGTTCCTGGCCGTCGTC
GTCTCTGCTCTTGGCAGACAGTACCAC
[60] Gadiculus_argentus_B3
ATG---GTTGAGTGGACTGCTGAAGAGCGCACCATCATTAACAACATCTTCTCCACCTTG
GACTATGAAGAGATTGGCCGCAAGTCTCTCACCAGGTGTCTGATCGTGTACCCCTGGACC
CAGAGGTACTTCGGTGCCTTCGGCAACCTGTACAACGCCGAGACCATCCTGGCCAACCCC
CTGATCGCCGCCCATGGCACCAGGATCCTGCACGGTCTGGACCGGGCCCTGAAGAACATG
GACGACATCAAGAACACCTACGCTGAGCTGTCCCAGCTCCACTCCGAGAAGCTTCACGTG
GACCCCGACAACTTCAGGCTGCTGGCCGACTGCCTGACCGTCGTCATCGCCGCCAAGATG
GGCACCGCC---TTCACCGTGGACATCCAGGTGGCGTGGCAGAAGTTCCTGTCTGTCGTC
GTCGCCGCTCTGGGCAGACAGTACCAC
[61] Macrourus_berglax_B3
ATG---GTTGAGTGGACTGAGTCAGAGCGCAGCATCATTAGCAGCATCTTCGCCAACTTG
GACTATGAGGAAATCGGCCGCAAGTCTCTTTGCAGGTGTCTGATTGTGTACCCATGGACC
CAGAGGTACTTTGGTGCATTTGGTAACCTGTACAACGCAGAGACCATCATGGCCAACCCC
CTGATCGCTAAGCATGGCACCACCATCGTGCACGGTCTGGACCGGGCTCTGAAGAACATG
GACAACATCAAGGAAACCTATGCAGAGCTGAGCGTGCTGCACTCCGAGAAACTGCATGTG
GACCCCGACAACTTCAGGCTGCTGGCTGACTGCATGACAGTTGTCATCGCTGCCAAGATG
GGAAACGCC---TTCACTATTGATACCCAGGTGGCATGGCAGAAGTTCCTGTCTGTCGTC
GTCTCTGCTCTGGGCAGACAGTACCAT
[62] Gadus_morhua_B4_B4
ATG---GTTGAGTGGACAGATAGTGAGCGCGCCATCATTACTAGCATCTTCTCCAACTTG
GACTACGAAGAGATCGGCCGCAAGTCTCTGTGCAGGTGTCTGATCGTGTACCCCTGGACC
CAGAGGTACTTCGGCGGCTTCGGCAACCTGTACAACGCAGAGACCATCCTGTGCAACCCC
CTGATCGCCGCCCATGGCACCAAGATCCTGCACGGTCTGGACCGGGCCCTGAAGAACATG
GACGACATCAAGAACACCTACGCCGAGCTGTCTCTGCTCCACTCTGACAAGCTGCACGTG
GATCCCGACAACTTCAGGCTGCTGGCCGACTGCCTGACCGTCGTCATCGCCGCCAAGATG
GGCCCCGCC---TTCACCGTGGATACCCAGGTGGCGTGGCAGAAGTTCCTGTCTGTCGTC
GTCTCCGCTCTGGGCAGACAGTACCAC
[63] Theragra_chalcogramma_B4
ATG---GTTGAGTGGACAGATAGTGAGCGCGCCATCATTACTAGCATCTTCTCCAACTTG
GACTACGAAGAGATCGGCCGCAAGTCTCTGTGCAGGTGTCTGATCGTGTACCCCTGGACC
CAGAGGTACTTCGGCGGCTTCGGCAACCTGTACAACGCAGAGACCATCCTGTGCAACCCC
CTGATCGCCGCCCATGGCACCAAGATCCTGCACGGTCTGGACCGGGCCCTGAAGAACATG

GACGACATCAAGAACACCTACGCCGAGCTGTCTCTGCTCCACTCTGACAAGCTGCACGTG
GATCCCGACAACTTCAGGCTGCTGGCCGACTGCCTGACCGTCGTCATCGCCGCCAAGATG
GGCCCCGCC---TTCACCGTGGATACCCAGGTGGCGTGGCAGAAGTTCCTGTCTGTCGTC
GTCTCCGCTCTGGGCAGACAGTACCAC
[64] Gadus_morhua_B5_B5
ATG---GTAGAGTGGACAGAATTTGAGCGCGATACAATCAAGGACATCTTCTCTAAAATC
GACTATGACGTGGTGGGTCCCGCCGCCCTCACAAGGTGCCTGGTTGTGTACCCGTGGACT
CGGAGGTACTTTGGAAACTTCGGGGCCCTCTACAACGCCGAGGCAATCATGGGCAACGAG
ATGGTGGCAAACCACGGGAAAAAAGTGCTCCACGGACTGGACCGAGCCGTGAAGAACATG
GATCACATCAAGGAGAGCTATTGCGAGCTCAGTCAGCTGCACTCCGATCAGTTTCACGTG
GACCCTGACAACTTCAGACTGCTTGCTGACTGCCTTGCAATCGCCATTGCCACCCAGTGG
GGCAGTGCA---TTCACCCCGGACATCCAGGCCGCGTTCCAGAAGTTCTTGTCCGTTGTC
GTCTTCTCTTTGGGAAGCCAGTACCAC
[65] Arctogadus_glacilis_B5
ATG---GTAGAGTGGACAGAATTTGAGCGCGATACAATCAAGGACATCTTCTCTAAGATC
GACTATGACGTGGTGGGTCCCGCCGCCCTCACAAGGTGCCTGGTTGTGTACCCGTGGACT
CGGAGGTACTTTGGAATCTTCGGGGCCCTCTACAACGCCGAGGCAATCATGGGCAACGAG
ATGGTGGCAAACCACGGGAAAAAAGTGCTCCACGGACTGGACCGAGCCGTGAAGAACATG
GATCACATCAAGGAGAGCTATTGCGAGCTCAGTCAGCTGCACTCCGATCAGTTTCACGTG
GACCCTGACAACTTCAGACTGCTTGCTGACTGCCTTGCAATCGCCATTGCCACCCAGTGG
GGCAGTGCA---TTCACCCCGGACATCCAGGCCGCGTTCCAGAAATTCTTGTCCGTTGTC
GTCTTCTCTTTGGGAAGCCAGTACCAC
[66] Boreogadus_saida_B5
ATG---GTAGAGTGGACAGAATTTGAGCGCGATACAATCAAGGACATCTTCTCTAAGATC
GACTATGACGTGGTGGGTCCCGCCGCCCTCACAAGGTGCCTGGTTGTGTACCCGTGGACT
CGGAGGTACTTTGGAAACTTCGGGGCCCTCTACAACGCCGAGGGAATCATGGGCAACGAG
ATGGTGGCAAACCACGGGAAAAAAGTGCTCCACGGACTGGACCGAGCCGTGAAGAACATG
GATCACATCAAGGAGAGCTATTGCGAGCTCAGTCAGCTGCACTCCGATCAGTTTCACGTG
GACCCTGACAACTTCAGACTGCTTGCTGACTGCCTTGCAATCGCCATTGCCACACAGTGG
GGCAGTGCA---TTCACCCCGGACATCCAGGCCGCGTTCCAGAAGTTCTTGTCCGTTGTC
GTCTTCTCTTTGGGAAGCCAGTACCAC
[67] Trisopterus_minutus_B5
ATG---GTGGTGTGGACAGACTTCGAGCGCGAAACAATCAAGGCCATCTTCTCTAAAATC
GACTATGACGTGGTTGGTCCCGCCGCCCTTACCAGGTGCCTGGTTGTGTACCCGTGGACT
CGGAGGTACTTTGGAAACTTTGGGGCCCTGCACAACGCCGCGGCAATCATGGGCAACGAG
ATGGTGGCAAACCACGGGAAGAAAGTGCTTCACGGACTGGACCGGGCTGTGCAGGACATG
GACCACATCAAGGAGAGCTATTGCGAGCTCAGTCAGCTGCACTCCGATCAGTTTCACGTG
GACCCCGACAACTTCAGACTGCTGGCTGACTGCCTGGCAATCGCCATCGCCACCCAGTGG
GGGAGTGCA---TTCACCCCGGACATCCAGGCCGCTTTCCAGAAGTTCTTGTCCGTTGTT
GTCTTCTCTTTGGGAAGCCAGTACCAC
[68] Pollachius_virens_B5
ATG---GTAGAGTGGACAGAATTTGAGCGCGATACAATCAAGGACATCTTCTCTAAGATC
GACTATGACGTGGTGGGTCCCGCCGCCCTCACAAGGTGCCTGGTTGTGTACCCGTGGACT
CGGAGGTACTTTGGAAACTTCGGGGCCCTCCACAACGCCGAGGCAATCATGGGCAACGAG
ATGGTGGCAAACCACGGGAAAAAAGTGCTCCACGGAATGGACCGAGGCGTGCAGAACATG
GACCACATCAAGGAGAGCTATTGCGAGCTCAGTCAGCTGCACTCCGATCAGTTTCACGTG
GACCCTGAAAACTTCAGACTACTTGCTGACTGCCTTGCAATCGCCATTGCCACCCAGTGG
GGGAGTGCA---TTCACCCCGGACATCCAGGCCGCGTTCCAGAAGTTCTTGTCCGTTGTC
GTCTTCTCTTTGGGAAGTCAGTACCAC
[69] Melanogrammus_aeglefinus_B5
ATG---GTAGAGTGGACAGAATTTGAGCGCGATACAATCAAGGATATCTTCTCTAAGATC
GACTATGACGTGGTGGGTCCCGCCGCCCTCACAAGGTGCCTGGTTGTGTACCCGTGGACT
CGGAGGTACTTTGGAAACTTCGGGGCCCTCTACAACGCCGAGGCAATCATGGGCAACGAT
ATGGTGGCAAACCACGGGAAAAAAGTGCTCCACGGACTGGACCGAGCCGTGCAGAACATG
GACCACATCAAGGAGAACTATTGCGAGCTCAGTCAGCTGCACTCCGATCAGTTCCACGTG
GACCCTGACAACTTCAGACTGCTTGCTGACTGCCTTGCAATCGCCATTGCCACCCAGTGG
GGGAGTGCA---TTCACCCCGGACATCCAGGCCGCGTTCCAGAAGTTCTTGTCCGTTGTC

GTATTCTCTTTGGGAAGCCAGTACCAC
[70] Merlangius_merlangus_B5
ATG---GTAGAGTGGACAGAATTTGAGCGCGATACAATCAAGGACATCTTCTCTAAGATC
GACTATGACGTGGTGGGTCCCGCCGCCCTCACAAGGTGCCTGGTTGTGTACCCGTGGACT
CGGAGGTACTTTGGAAACTTCGGGGCCCTCTACAACGCCGAGGCAATCACGGGCAACGAG
ATGGTGGCAAACCACGGGGAAAAAGTGCTCCACGGACTGGACCGAGCCGTGCAGGACATG
GACAACATCAAAGAGAGCTATTGCGAGCTCAGTCAGCTGCACTCCGATCAGTTTCACGTG
GACCCTGATAACTTCAGACTGCTTGCTGACTGCCTTACAATCGCCATTGCCACCCAGTGG
GGGAGTGCA---TTCACCCCGGACATCCAGGCCGCGTTCCAGAAGTTCTTGTCCGTTGTC
GTCTTCTCTTTGGGAAGCCAGTACCAC
[71] Theragra_chalcogramma_B5
ATG---GTAGAGTGGACAGAATTTGAGCGCGATACAATCAAGGACATCTTCTCTAAGATC
GACTATGACGTGGTGGGTCCCGCCGCCCTCACAAGGTGCCTGGTTGTGTACCCGTGGACT
CGGAGGTACTTTGGAAACTTCGGGGTCCTCTACAACGCCGAGGCAATCATGGGCAACGAG
ATGGTGGCAAACCACGGGAAAAAAGTGCTCCACGGACTGGACCGAGCCGTGAAGAACATG
GATCACATCAAGGAGAGCTATTACGAGCTCAGTCAGCTGCACTCCGATCAGTTTCACGTG
GACCCTGACAACTTCAGACTGCTTGCTGACTGCCTTGCAATCGCCATTGCCACCCAGTGG
GGCAGTGCA---TTCACCCCGGACATCCAGGCCGCGTTCCAGAAGTTCTTGTCCGTTGTC
GTCTTCTCTTTGGGAAGCCAGTACCAC
[72] Gadiculus_argentus_B5
ATGGTCGTCGAGTGGACAGACTTTGAACGCGAAACAATCAAGGACATCTTCTCCAAGATC
GACTATGACGTGGTGGGTCCCGCCGCCCTTACCAGGTGCCTGGTTGTGTACCCGTGGACT
CGGAGGTACTTCGGAAACTTTGGCACTCTCCACAACACCGCGGCAATCGTGGGCAACGAG
ATGGTGGCAAACCACGGAAAAATGGTGCTCCACGGCCTGGACCGAGCCGTGCGGAACATG
GACCACATCAAGGAGAGCTACACCGAGCTCAGCAAGCTGCACTCCGATCAGTTCCACGTG
GACCCCGACAACTTCAGACTCCTGGCTGACTGCCTGGCAATCGCCATTGCCACCCAGTGG
AGGGAGGAA---TTCACCCCGGACATCCAGGCCGCCTTCCACAAGTTCTTGTCCGTTGTT
GTCTACTCTTTGGGGAGCGAGTACCAC
[73] Phycis_phycis_B5
ATG---GTCGAGTGGACGGACTTCGAGCGCGCAACAATCCGGGACATCTTCTCAAAGATG
GACTATGACGTCGTGGGTCCCGCCGCTCTTACCAGGTGCCTGATTGTATACCCATGGACC
CAGAGGTACTTCGGCAACTTTGGGACCCTCTACAACGCCGCGGCAATAATGGGAAACGAG
ATGGTTGCAAACCACGGGAAAAAAGTGCTCCACGGACTGGACCGGGCCGTGCAGGACATG
GACAACATCAAGGAAAATTATTCCGAGCTCAGTCAGCTGCACTCTGACACATTTCACGTG
GACCCCGACAACTTCAGACTGCTGGCTGACTGCCTGGCAATCGCCATTGCCACCCAGTGG
GGGAGCGCA---TTCACCCCGGACGTCCAGGCCGCGTTCCATAAGTTTTTGTCCATTGTC
GTGTTCTCGATGGGGACGCAGTATCAC
[74] Molva_molva_B5
ATG---GTCGAGTGGACAAACTTTGAGCGCGAAACAATCCAGGACATCTTCTCTAAGATT
GACTATGACGTCGTGGGTCCCGCCGCCCTTTCCAGGTGCCTGGTTGTATACCCATGGACT
CGGAGGTACTTTGGAAACTTTGGGAACCTCTACAACGCCGCAGCCATCATGGGCAACGAG
ATGGTTGCAAACCACGGGAAAAAGGTGCTTCACGGATTGGACCGGGCCGTACAGAACATG
GACAACATCAAGGAAAGCTATTACGAGCTCAGTCAGCTGCACTCCGATAAATTTCACGTG
GACCCCGACAACTTCAGACTGCTGGCTGACTGCCTGGCAATCGTCATTGCCACCCAGTGG
GGAAGTGCA---TTCACCCCGGACATCCAGGCCGCGTTCCAGAAGTTCTTGTCCATTGTC
GTCTTCGCTCTGGGAAGCCAGTACCAC
[75] Lota_lota_B5
ATG---GTCGAGTGGACAGACTTTGAGCGCGCAACAATCCAGGACATCTTTTCTAAGATC
GACTATGACGTCGTGGGTCCCGCCGCCCTTTCCAGGTGCCTGGTTGTGTACCCATGGACT
CGGAGGTACTTTGGAAACTTTGGGAACCTCTACAACGCCGCAGCCATCATGGGCAACGAG
ATGGTTGCAAACCACGGGAAAAAAGTGCTTCACGGATTGGACCGGGCCGTGCAGAACATG
GACAACATCAAGGAAAGTTATTACGAGCTCAGTCAGCTGCACTCCGATAAATTTCACGTG
GACCCCGACAACTTCAGACTGCTGGCTGACTGCCTGGCAATCGTCATTGCCATCCAGTGG
GGAAGTGCA---TTCACCCCGGACATCCAGGCTGCGTTTCAGAAGTTCTTGTCCGTTGTC
GTCTTCTCCCTGGGAAGCCAGTACCAC
[76] Brosme_brosme_B5
ATG---GTCGTGTGGACAGACTTTGAGCGCGAAACAATCAAGGACATCTTCTCTAAGATC

GACTATGACGTCGTTGGTCCCGTCGCCCTTTGCAGGTGCCTGGTTGTATACCCATGGACT
CGGAGGTACTTTGGGAACTTTGGAACCATCTACAACGCCGCAGCCATCATGGGCAACGAG
ATGGTTGCAAACCACGGGAAAAAAGTGCTCCACGGATTGGACCGGGCAGTGCAGAACATG
GACAACATCAAGGAAAGTTATTACGAGCTCAGTCAGCTGCACTCCGATACATTTCACGTG
GACCCCGACAACTTCAGACTGCTGGCTGACTGCCTGGTAATCGCCATTGCCACCCAGTGG
GGAAGTGCA---TTCACCCCGGACATCCAGGCCGCGTTCCAGAAATTCATGTCCATTGTC
GTCTTCTCTCTGGGAAGCCAGTACCAC
[77] Merluccius_capensis_B5
ATG---GTCGAGTGGACGGACTTTGAGCGCGAGACCATCAAGCACATCTTCTCCAAGATC
GACTATGACGTCGTGGGTCCCGCCGCGCTCTGCAGGNNNNNNNNNNNNNNNNNNNNNNNN
NNNNGGTACTTCGGGAACTTCGGGATCCTGTACAACGCGGACACCATCACGGGGAACGAG
CAGGTGGCCCGTCACGGGGTGAAGGTGCTGCGCGGGCTGGACCGGGCCGTGCGGGACATG
GACCACATCAAGGAGGGCTACGCCGAGCTGGGACTGCTGCACTCGGACAAGTACCACGTG
GACCCAGACAACTTCAAACTGCTGGCGGACTGCCTGACCATCGTCATCGCCACGGAGTGG
GGCAGTTTG---TTCACCCCCGAGATCCAGGCCGCCTTCCAGAAGTTCATGTCCGTCGTC
GTCTTCTCTCTGGAGAGGCAGTACCAC
[78] Bregmaceros_cantori_B5
ATG---GTGGAATGGACGGAGCTGGAGCGTCAAACTTTAAAAGACATATTTTCAAAAATT
GACTATGATGTCGTGGGCAAAGCTGCGCTCATCAGGTGTCTCGTGGTCTACCCCTGGACG
CAGCGCTATTTTGGCGATGTCCAGAGCCTCCACAACGAGGAGGCCATCGCAGCAAGTCCA
AAAATCGCTGCCCACGGTCCCAAGATCCTCCGCGGCCTGGACCGAGCCGTGGAAGACCTG
GAAGGGATCAGAGAGAACTACGTGGGTCTCAGCAAGATGCACTCC---TCCATCCATGTG
GACCCTGAAAACTTCAGACTGATGGCCGACTGTTTGACAGTGGAGCTGGCGACCCAGTGG
AAGAGCGTC---TTCACCCCCGACATGCAGGCCACATTCCAGAAGTTTCTGTCCGTGCTG
GTCTACGCCTTGAGCCGGGAATACTAT
[79] Percopsis_transmontana_B5_1
ATG---GTTGAATGGACAGAATTCGAGCGCGCTACCATCACAGACATCATGTCTAAGATG
GACTACCAAATCATCGGACCGGCAGCTCTTTCCAGGTGCCTGATCGTCTACCCCTGGACT
CAGAGGTATTTCGGGAACTTTGGAAACCTCTACAACGCCGATGCCATCACTGCGAACCCA
ATGGTGGCTGCCCACGGGATCACAGTGCTGCGTGGACTGGACCGGGCTGTGAAGAACATG
GACAACATCAAGGCCACTTATGCTGAGCTTAGCGTGCTGCACTCCGAGAAGCTGCACGTG
GACCCTGACAACTTCCGGCTGCTGGCTGACTGCCTGACCATCGTCCTTGCTTCCAAAATG
GGAACTGGA---TTCACAGCTGACGTCCAGGCCGCCTGGCAGAAGTTCCTGGCCGTCGTG
GTGTCCTCCCTGGGAAGACAGTACCAC
[80] Phycis_blennoides_B1
ATG---GTTGAGTGGACGGACAGCGAGCGCAGGGCCATCGAGAAGGTCTGGAGCTACATC
AACATTGATGTCATTGGACCGCTCATGTTGCAAAGGTGCCTTATTGTGTATCCCTGGACG
CAGCGGTACTTCGGTGCCTTTGGCGACCTGAGCACCGACCAGGCCATCGCTGACAACCCC
AAGGTGGCAAAGCACGGCATCGTCGCTCTGACCGGCCTGAAGACGGCTCTGGATAACATG
GACTGCATCAAAGCCACGTACGCTCCTCTGAGCGTGCTGCATTCCGAGACACTGCACGTT
GATCCCGACAACTTCAGACTGCTGTGTGACTGCCTGACCGTGATCGTCGCTGGGAAGATG
GGTCTCAAA---TGCACCCCGTACATTCATGCTGCGTGGCAGAAGTACCTGTGCGTCGTA
GTCGGTGCCCTCGGGAAGCAGTACCAC
[81] Phycis_blennoides_B2
ATG---GTTCAGTGGACTGACGATGAGCGCGCCATCATCAATGGCATCTTCGCCAACCTG
GACTACGAAGATATCGGCCGCAAGTCTCTTTGCAGGTGTCTGATCGTGTACCCATGGACC
CAGAGGTATTTCGGAGGATTCGGTAACCTGTACAGCGCAGAGACCATCCTGGGCAACCCC
CTGATCGCCGCCCACGGTACCAAGATCCTGCACGGTCTGGACCGGGCCCTGAAGAACATG
GACGACATCAAGAACACCTACTCCGAGCTGAGTCAGCTCCACTCCGAGAAGCTGCACGTG
GACCCCGACAACTTCAGGCTGCTGGCTGACTGCCTGACGGTCGTCATCGCCGCCCAGATG
GGAAACGCC---TTCACCGTGGATATCCAGGTCGCATGGCAGAAGTTCCTGGCTGTCGTT
GTCTCTGCTCTGGGCAGACAGTACCAT
[82] Phycis_blennoides_B5
ATG---GTCGAGTGGACAGCCTTCGAGCGCGCAACAATCCGGGACATCTTCTCAAAGATG
GACTATGACGTCGTGGGTCCCGCCGCTCTTGCCAGGTGCCTGATTGTATACCCGTGGACC
CAGAGGTACTTTGGCAACTTTGGGTCCCTCCACAACGCCGCGGCAATAATGGGAAACGAG
AAGGTTGCAAACCACGGGAAGAAAGTGCTCCGCGGACTGGACCGGGCCGTGCAGAACATG

GATAACATCAAGGAAAGTTATTCCGAGCTCAGTCAGCTGCACTCCGATACATTTCACGTG
GACCCCGACAACTTCAGACTGCTGGCTGACTGCCTGGCAATCGCCATTTCCACCCAGATG
GGGAGCGCG---TTCACCCCGGACGTCCAGGCCGCGTTCCATAAGTTTTTGTCCATCGTC
GTGTTCTCTATGGGAACGCAGTACCAC
[83] Thrachyrincus_murrayi_B1
ATG---GTTCACTGGACAGAAAGCGAGATTGCGGCCGTCACAAAGGTCTGGAGAAACATC
GACGTTGAAGTCATCGGACCACTCGTCTTGACTAGGTGCTTAATTGTGTATCCCTGGACG
CAGCGCTACTTCGGTAGCTTCGGCGACCTGAGCACAGAGCAGTCTATTAAGTCCAACCCC
AAGGTGGCCAAGCACGGCATAACCGTCTTGAGCAGCCTGAAGACGGCGATGGACAACATG
GACAACATCAAGAACACCTACGCTAGCCTGAGCACGCTGCACTCCGACACACTGCACGTC
GACCCGGACAACTTCAGGCTGCTGTGTGACTGCCTGACCATAATCATCGCCGGGAAAATG
GGACGCAAA---TTCACCCCGGACATGCAAGCGGCGTGGCAGAAGTACCTGGCCGTGATC
GTCTCCGCCCTCAGCAGACAGTACCAC
[84] Thrachyrincus_murrayi_B2
ATG---GTTGAGTGGACTGATGAGGAGCGCAGCATCATTGACGGCATCTTCGCCAACTTG
GACTACGAAGAAGTCGGCAAGAAGTCTCTTTGCAGGTGTCTCATCGTCTACCCATGGACC
CAGAGGTACTTCGGTGCATATGGCAACCTGTACAACGCAGAGACCATCCTGTGCAACCCC
CTGATCGCCGCCCACGGCACCAAGATCCTGCACGGTCTGGACCGGGCCCTGAAGAACATG
GACAACATCAAGGAGACCTACTCTGAGCTGAGTCATCTGCACTCCGAGAAGCTGCACGTG
GACCCCGACAACTTCAGGCTGCTGGGTGACTGCATGACCGTCGTCATTGCCGCCAAGATG
GGAGCCGCC---TTCACAATTGAAACCCAGTTCGCGTTCCAGAAGTTCCTCAGCGTGGTC
GTCTCCGCTCTGGGCAGACAGTACCAC
[85] Stylephorus_chordatus_B5_3
ATG---GTCGAGTGGACAGACTTTGAGCGCGCTACAATTCAGGACATCTTCTCTAAGATG
GACTACGACAGCATGGGTGCCGCCGCCCTTTCCAGGTGCCTGATCGTCTATCCATGGACT
CAGAGGTATTTCGGAAACTTTGGAAACCTCTACAACGCCGCTGCCATTAAAACAAACCCA
ATGGTTGCAAAGCACGGAACGACTATTGTCCACGGTCTGGACAGAGCCCTAAAGAACATG
GACAACATCAAGGAAGCTTATGCTGACCTCAGTGTGCTGCACTCCGAGAAATTGCACGTG
GACCCTGACAATTTCAGACTGTTTTCCGACTGTCTGACCATTACCGTCGCTGCCCAGTGG
GGACATGCG---TTCACTCCTGACATCCAGGCAGCTTTCCAAAAGTTCTTGTCTGTCGTT
GTGGCCTCTCTCTCACAACAGTACCAC
[86] Stylephorus_chordatus_B5_1
ATG---GTCGAGTGGACAGACTTTGAGCGCGCTACAATTCAGGCCATCTTCGCTAAGATG
GACTACGACAACATGGGTGGCGCCGCCCTTTCCAGGTGCCTGATCGTCTACCCATGGACT
CAGAGGTATTTCTTAAAGTTTGGAAACCTCTACAACGCCGCTGCCATTAAAACAAACCCA
ATGGTTGCAAAGCATGGAACAATTATTCTCCACGGTCTGGACAAAGCCCTACAGAACTTG
GACAACATGGCGGAAGCTTATGCTGACCTCAGTGTGCTGCACTCCGAGAAACTGCACGTG
GACCCTGACAATTTCAGACTGTTTTCCGACTGTCTGACCATTACCGTCGCTGCCCAGTGG
GGGCATGCG---TTCACTGTTGACATCCAAGCCGCTTTCCAAAAGTTCATGTCTGTCGTT
GTGGCCTCTCTCTCAAAACAGTACCAC
[87] Stylephorus_chordatus_B5_2
ATG---GTCGAGTGGACAGACTTTGAGCGCGCTACAATTGAGGCCATCTTCGCTAAGATG
GACTACGACACTATGGGTGCCGCCGCCCTTTCCAGGTGCCTGGTCGTCTATCCATGGACT
CAGAGGTATTTCGGAAACTTTGGAAACCTCTACAACGCCGCTGCCATTAAAACAAACCCA
CTGATTGCAAAGCACGGAACGACTATTGTCCACGGTCTGGACAGGGCCCTAAAGAACTTG
GACAACATAAAGGAGGCTTATGCTGAGCTCAGCGTGCTGCACTCCGAGAAACTGCACGTG
GACCCTGACAATTTCAGACTGTTTTCTGACTGCCTAACCATTACCGTCGCTGCCCAGTGG
GGGAATGCG---TTCACTGCTGACGTCCAGGCCGCTTTCCAAAAGTTCTTGTCTGTCGTT
GTGTCCTCTCTTGGAAGACAGTACCAC
[88] Zeus_faber_Bx_2
ATG---GTTGAGTGGACAGAGAGCGAGCGCAGAACCGTCACTGCAGTTTGGGACAAAATA
AATGTGGATGAGATTGGGCCACAATCTCTGGCAAGGGTCCTGATCGTGTATCCCTGGACA
CAGCGGTATTTCCGCTCTTTCGGAGATGTCTCTTACGCATCCGCTATTTTGAACAACCCC
AAAGTGTCCTCGCACGGCAAAGTGGTGCTGAAGGCGCTGGACTGGGCGGTGAGGAACATG
GACAACATCAGGGGCGTCTACTCTGAGCTGAGCCGGATCCACTGTGAGAAACTCAAGGTG
GATCCTGATAACTTCAGGCTGCTGGCAGATTGCATCACCATTGCTGTTGCCTGCAAACTG
CGCAGCGAA---TTCCCTCCCAAGGATCAAGCAATCTGGCAGAAGTTTCTAAATGCCGTG

GTGGACGCCATGGGGAGTCAGTACACA
[89] Zeus_faber_Bx_1
ATG---GTCGAGTGGACAGATGTTGAGCGCGCCGCCATCATTGACCTTTGGAAGAGGATC
AACGTGGATGAGATTGGACCCCAGGCTCTGGGCAGGCTTCTGATCGTGTCCCCATGGACC
CAGAGGCACTTCTCCACATTGGGCAACTTGTCGAACGCCGCGGCTATTATGACGAATCCC
GCCGTGGCCGAGCACGGGAGACTCATCATGGGTGGCCTCGACAGGGCTGTGAAGAACCTG
GATGACATCAAGAACACCTACAAAACCCTTAGTTCGAAGCACTCTGAAATCCTGCACGTT
GATCCTGATAACTTCAGGCTTATGGCCGAATCCATCACCGTGTGTGTGGCCATGAAGTTT
GGCGGCACCATCTTCACCCCTGATGTCCACGAGGCCTGGCAGAAGTTCCTGTCGGTTGTC
GTCTCGGCCCTTGGCAGGCAGTACCAC
[90] Danio_rerio_MN_Hbb1
ATG---GTGCAGTGGTCAGATTCCGAGCGCAAAACAATTGCGAGTGTCTGGAGCAAAATC
AACGTCGATGAAATCGGACCACAGACCTTGGCAAGGGTGCTGGTTGTTTATCCCTGGACT
CAGAGGTATTTTGGCGCATTTGGAGATCTATCCTGTGCATCTGCAATCATGGGCAACCCC
AAAGTTTCAGAACACGGCAAAACTGTGCTGAAAGCGTTGGAGAAAGCTGTCAAGAACGTG
GATGACATCAAAACCACTTACGCCAAACTCAGCCAGCTGCACTGCGAGAAACTCAACGTC
GATCCAGACAACTTCAAGCTGTTGGCGGACTGCCTGAGCATTGTCATCGCGACTAATTTT
GGACCCGCT---TTCAACCCCTCAGTCCAGTCAACCTGGCAGAAACTTTTGTCAGTAGTT
GTGGCTGCTCTCACAAGCCGCTACTTC
[91] Danio_rerio_MN_Hbb2
ATG---GTTGAGTGGACAGATGCCGAGCGCACAGCCATCCTTGGCCTGTGGGGAAAGCTC
AATATCGATGAAATCGGACCTCAGGCCCTATCCAGATGTCTGATCGTGTATCCCTGGACT
CAGAGATATTTCGCCACATTCGGCAACCTGTCAAGCCCCGCTGCGATCATGGGTAACCCC
AAAGTGGCAGCTCATGGGAGGACTGTGATGGGAGGTCTTGAGAGAGCCATCAAGAACATG
GACAACGTCAAGAACACCTATGCCGCCCTCAGTGTGATGCACTCTGAGAAACTGCATGTG
GATCCCGACAACTTCAGGCTTCTCGCTGATTGCATCACCGTTTGCGCTGCCATGAAGTTC
GGCCAAGCTGGTTTCAATGCTGATGTCCAGGAGGCCTGGCAGAAGTTTCTGGCTGTGGTC
GTTTCTGCTCTGTGCAGACAGTACCAC
[92] Danio_rerio_MN_Hbb3
ATG---GTTGAGTGGACAGATGCCGAGCGCACAGCCATCCTTGGCCTGTGGGGAAAGCTC
AATATCGATGAAATCGGACCTCAGGCCCTATCCAGATGTCTGATCGTGTATCCCTGGACT
CAGAGATATTTCGCCACATTCGGCAACCTGTCAAGCCCCGCTGCGATCATGGGTAACCCC
AAAGTGGCAGCTCATGGGAGGACTGTGATGGGAGGTCTTGAGAGAGCCATCAAGAACATG
GACAACGTCAAGAACACCTATGCCGCCCTCAGTGTGATGCACTCTGAGAAACTGCATGTG
GATCCCGACAACTTCAGGCTTCTCGCTGATTGCATCACCGTTTGCGCTGCCATGAAGTTC
GGCCAAGCTGGTTTCAATGCTGATGTCCAGGAGGCCTGGCAGAAGTTTCTGGCTGTGGTC
GTTTCTGCTCTGTGCAGACAGTACCAC
[93] Danio_rerio_MN_Hbb4
ATG---GTTGTGTGGACAGACTTCGAGAAGGCCACCATTCAAGATATCTTCGCCAAGGCT
GACTACGACGTCATCGGTCCTCAGGCTCTGGCAAGGTGTCTCATCGTGTACCCCTGGACT
CAGCGGTACTTCGCCAAGTTTGGAAACCTCTACAATGCCGCTGCCATCCTGGGAAACCCA
ATGGTTGCTGCCCACGGTAAAACTGTGCTCAAGGGTCTGGAGCTGGCAGTGAAGAACATG
GACAACATCAAGGCCACCTATGCTGATTTAAGTGTGCTGCACTCCGAGAAGCTCCACGTA
GATCCCGACAACTTCAGGCTTTTGGCTGATTGCTTGACCATCGTTGTTGCTGCTCAGATG
GGTGCTGGA---TTCACACCTGAAGTTCAGGCCGCTTTCCAGAAATTCATCGCTGTCGCC
GTGTCCGCTCTGGGAAGACAGTACCAC
[94] Danio_rerio_MN_Hbb5
ATG---GTTGTGTGGACAGACTTCGAGAAGGCCACCATTCAAGATATCTTCGCCAAGGCT
GACTACGACGTCATCGGTCCTCAGGCTCTGGCAAGGTGTCTTATCGTGTACCCCTGGACC
CAGCGGTACTTCGCCAAGTTTGGAAACCTCTACAATGCCGCTGCCATCCTGGGAAACCCA
ATGGTTGCTGCCCACGGTAAAACTGTGCTCAAGGGTCTGGAGCTGGCTGTAAAGAACATG
GACAACATCAAGGCCACCTATGCTGATTTAAGTGTGCTGCACTCCGAGAAGCTCCACGTA
GATCCCGACAACTTCAGGCTTTTGGCTGATTGCTTGACCATCGTTGTTGCTGCTCAGATG
GGTGCTGGA---TTCACACCTGAAGTTCAGGCCGCTTTCCAGAAATTCATCGCTGTTGCG
GTGTCCGCTCTGGGAAGACAGTACCAC
[95] Danio_rerio_MN_Hbb6
ATG---GTTGTGTGGACAGACTTCGAGAAGGCCACCATTCAAGATATCTTCGCCAAGGCT

GACTACGATGTCATTGGTCCTCAGGCTCTGGCAAGGTGTCTCATCGTGTACCCCTGGACC
CAGCGGTACTTCGCCAAGTTTGGAAACCTCTACAATGCCGCTGCCATCCTGGGAAACCCA
ATGGTTGCTGCCCACGGTAAAACTGTGCTCAAAGGTCTGGAGCTGGCAGTGAAGAACATG
GACAACATCAAGGCCACCTATGCTGATTTGAGTGTGCTGCACTCCGAGAAGCTCCACGTA
GATCCCGACAACTTCAGGCTTTTGGCTGATTGCTTGACCATCGTTGTTGCTGCTCAGATG
GGTGCTGGA---TTCACACCTGAAGTTCAGGCCGCTTTCCAGAAATTCATCGCTGTCGCT
GTGTCCGCTCTGGGAAGACAGTACCAC
[96] Danio_rerio_LA_Hbb2
ATG---GTTGTGTGGACAGCTGAGGAGCGCGCAGCGATTCAGAACATCTTTGCCAAACTC
GACTTTGAGTCGGTGGGCCTCGAGACCTTGACAAGATGCTTGGTCGTCTATCCGTGGACT
CAGCGGTACTTTGGTGGGTTTGGAAACCTATACAACACAGAGGCAATCATGGCTAACCCA
AAAGTCAAAGCGCACGGTGTTGTGGTCCTCAAGGGACTTGAAAAGGCTCTCAACAACATG
GATAATATCAAGAGCACCTATGCTTCTCTCAGCGAGCTTCACTCAGAGAAACTACAAGTC
GACCCAGGCAATTTCCGGCTGTTAGCGGACTGCCTGACCGTGGTGATCGCCACACGCATG
AGGAGTGAG---TTCACCCCGGACATCCAAGCTGCTTGGCAGAAGTTCCTGTCTGTTGTT
GTGTCAGCCCTCAGAAGGCAATATCTT
[97] Danio_rerio_LA_Hbb1
ATG---GTTGCGTGGACCGCCGAGGAACGTGCCTTCATCCAGGACATCTTCAGCAAACTC
AACTATGAGGAGGCTGGACCCAAAGCACTGCAGAGGGCTTTGATTGTGTATCCCTGGACT
CAGAGATACTTTGGAAGTTTTGGAAACCTGTACAATGCTGAGGCCATCATTAACAACCCA
AAGGTCGCAGCCCACGGAACCGTCGTGCTCCACGGACTGGACAGAGCCATGAAGAACATG
GATGACATCAAGAACACCTACGCCGAGCTGAGCGTGCTGCACTCCGAGAAACTGCACGTG
GATCCTGACAACTTCAGGCTGCTGGCCGACTGCCTGACAATCGTGATTGCCTCCACCATG
GGCGCCGCT---TTCACAGCCGACATGCAGGCTGCCTGGCAGAAGTTCCTCGCTGTTGTT
GTCTCTGCTCTGCAAAGACAGTATCAT
[98] Oryzias_latipes_MN_Hbb1
ATG---GTCAAATGGTCAGACTTCGAGCGTGCCACAATCCAGGACATCTTCTCCAAGATA
GATAAGGATGTCGTGGGTCAGGCGGCTCTTTCCAGGTGTCTGATCGTCTACCCCTGGACT
CAGAGGTACTTCGGCAGCTTTGGGAACCTCTACAACGCCGCCGCCATCACCTCCAACCCA
AAGGTAGCAGCCCATGGAAAGGTTGTCATTGGGGGTTTGGAAAAAGCTTTGCAGAACATG
GACAACATCAAGCAGGCTTATACAGATCTGAGTGTGCTCCACTCAGAGAAACTGCAGGTG
GATCCTGACAATTTCAAGCTCCTGGCTGACTGCCTGACCATCGTGGTTGCTTCCAATTTG
GGTATCAGC---TTCACACCTGAAGTCCAAGCAGCTTTCCAGAAGTTTCTGGATGTTGTG
GTGGCCGCCCTGAGGAAGCAGTACTAC
[99] Oryzias_latipes_MN_Hbb4
ATG---GTTGAATGGACAGACTTTGAGCGCGCCACCATCCAGGACATCTTCTCCAGGATA
GACAAGGATGTTGTTGGACCTGCTGCTCTCTCCAGGTGTCTGATCGTCTACCCCTGGACT
CAGAGGTACTTTGGCAGCTTTGGAAACCTCTACAACGCCGCCGCCATCACCTCCAACCCA
AAGGTCGCAGCACACGGAAAGGTTGTCCTGTCGGGTCTGGAGCTGGCCGTGAAGAACATG
GATGACATCAAGCAGACTTACGCAGATCTGAGCGTGCTGCACTCCGAGAAACTGCATGTG
GACCCCGACAATTTTAAGCTCCTGGCAGACTGCCTGACGATTGTGGTCGCCGCTCAGATG
GGACCAGAT---TTCACCTGCGACGTCCAGGCAGCTTTCCAGAAGTTCCTGGCCGTGGTG
GTGTCTTCCCTCCGGAGGCAGTACCAC
[100] Oryzias_latipes_MN_Hbb2
ATG---GTCACCTGGACAGACTTTGAGCGTGCAACCATCCAGGACATCTTCTCCAAGATA
GATTATGATATTGTGGGGCCTGATGCTCTTTCCAGGTGTCTGATCGTCTACCCCTGGACT
CAGAGGTACTTCGGCAGCTTTGGGAACCTCTACAATGCTGCTGCCATCGCGTCCAACCCA
AAGGTAGCAGCACATGGAAAGGTTGTCCTTGGGGGTTTGGAAAAAGCTCTAAAGAACATG
GACGACATCAAGCAGGCTTATGCAGATCTGAGTGTCCTGCACTCTGAAAAACTACAAGTA
GATCCTGACAATTTCAAGCTCCTGGCTGACTGCCTGACCATCGTCGTTGCCTCTCAGCTC
GGTAAAGAC---TTCACACCGGAGGTGCATGCAGCGTTTGCAAAGTTCTTGGCTGTGGTG
GTGGCAGCTCTCAGGAAGCAGTACCAC
[101] Oryzias_latipes_MN_Hbb3
ATG---GTCAAGTGGACAGAAGAGGAGCGCTGCACCATCAGGGAAGTTTGGGAAAAAGTT
GATGTTGATGAGATTGGACCACAAATTCTAACAAGGGTTCATATTGTTTACCCCTGGAGA
GAGACATACTTTGGCACCTTTGGAGACATTTTCACTAACACTTCCATTCTGAATAACCCC
AAAGTAGCTCACCATGGAAAGGTTGTGCTGAGGTCCATTGACAAAGCAGTGAGAATCATG

GACAGGATACAAGAGACACATGCTGCTCTGAGCAGGCTGCACTATGAA------TGTGTG
GATCCGGATAACTTCAAACTGCTGGGAGACTGCATCACCATCTCCATTGCCTGCAAACTC
AAGGAAGCC---TTGAACCCTCAGGTCCAAGCAGTCTGGCAGAAGTTCCTCTGTGCTGTG
GTGGAGGCCATGAACAGCCAGAACAAA
[102] Oryzias_latipes_LA_Hbb1
ATG---GTTGAGTGGACCGAGCAGGAGCGCAGCATCATCACCAACATCTTCGGCAACCTG
GACTATGAAGACGTGGGCTCCAAGGCTCTCAGCAGGTGTCTGATCGTCTACCCCTGGACT
CAGAGGTACTTTGCCAGCTTCGGTAACCTCTACAACGCCGAAGCCATCAAGACCAACCCG
AACATCGCCGCCCACGGCACCAAGGTCCTGCACGGTCTGGACCGCGCCGTGAAGAACATG
GACAACATCAAGGCCACCTACGCCGAGCTGAGCGTGCTGCACTCCGAGAAGCTGCACGTG
GACCCCGACAACTTCAAGCTGCTGGCTGACTGCTTGACCATCGTCATTGCCGCCAAACTG
GGCTCCGCC---TTCAGCCCAGAGATTCAGGCAACCTTCCAGAAGTTCTTGGCCGTGGTG
GTGTCCGCTCTGGGAAGGCAGTACCAC
[103] Gasterosteus_aculeatus_MN_Hbb1
ATG---GTCGTATGGACAGACTTCGAGCGCTCCACCATCCAGGACATCTTCTCCAAGATG
GACTATGAGGTGGTGGGCCCTGCAGCTCTCACCAGGTGTCTCATCGTCTACCCCTGGACT
CAGAGGTATTTCGGCAACTTCGGAAACCTCTACAACGCCGCCGCGATCATGGGAAATCCG
ATGGTTGCAAAACACGGCACCACAATCCTCCACGGCCTGGACCGCGGTGTGAAGAACATG
GACGACCTCAAGGCAACCTACGCCGAGCTGAGCGTGCTGCACTCCGAGAAACTGCACGTG
GACCCCGACAACTTCAAGCTTCTCTCCGACTGCCTGACCATCGTGGTGGCTGCAAAGTTG
GGCAAAGCC---TTCACTGGAGAAGTCCAGGCAGCTTTCCAGAAGTTCCTGGCCGTGGTG
GTGTCCTCCCTGGGAAGACAGTACCAC
[104] Gasterosteus_aculeatus_MN_Hbb2
ATG---GTCGTATGGACAGACTTCGAGCGCTCCACCATCCAGGACATCTTCTCCAAGATG
GACTATGAGGTGGTGGGCCCTGCAGCTCTCACCAGGTGTCTCATCGTCTACCCCTGGACT
CAGAGGTATTTCGGCAACTTCGGAAACCTCTACAACGCCGCCGCGATCATGGGAAATCCG
ATGGTTGCAAAACACGGCACCACAATCCTCCACGGCCTGGACCGCGGTGTGAAGAACATG
GACGACCTCAAGACAACCTACGCCGAGCTGAGCGTGCTGCACTCCGAGAAACTGCACGTG
GACCCCGACAACTTCAAGCTCCTCTCCGACTGCCTGACCATCGTGGTGGCTGCTCAGTTG
GGCAAAGCC---TTCACTGCAGAGGTCCAGGCAGCTTTCCAGAAGTTCCTGGCCGTGGTG
GTGTCCTCCCTGGGAAGACAGTACCAC
[105] Gasterosteus_aculeatus_MN_Hbb3
ATG---GTTGCATGGACAGAATTCGAGCGCGCCACCATCCAGGACATCTTCTCCAAAATC
GACTATGATGTCGTAGGTCCTGCAGCTCTGTCCAGGTGTCTGGTCGTCTACCCCTGGACT
CAGAGGTATTTCGGTAACTTCGGAAACCTCTACAACGCCGCAGCAATTATGGGAAATCCG
CTGGTTGCAAAACACGGAACGACTATCCTCAACGGACTGGAGCGGGCTGTGAAGGACATG
GACAACATCAAGGCAACCTACGCCGAGCTGAGCGTGCTGCACTCCGAGAAACTGCACGTG
GACCCCGACAACTTCAAGCTCCTCTCCGACTGCCTGACCATCGTGGTGGCTGCTCGGTTC
GGTGAAGCC---TTCACTGCAGAAGTCCAGGCAGCTTTCCAGAAGTTCCTGGCCGTGGTG
GTGTCCTCCCTGGGAAGACAGTACCAC
[106] Gasterosteus_aculeatus_MN_Hbb4
ATG---GTTGCATGGACAGAATTCGAGCGCGCCACCATCAAGGACATCTTCTCCAAGATC
GACTACGATGTCGTAGGTCCTGCAGCTCTGTGCAGGTGTCTGGTCGTCTACCCCTGGACT
CAGAGGTATTTCGGTAACTTTGGAAACCTCTACAACGCCGCAGCAATTATGGGAAATCCG
CTGGTTGCAAAACACGGAACAACTATTCTCAACGGACTGGAGCGGGCTGTGAAGGACATG
GACAACATCAAGACAACTTACGCCGAGCTGAGCGTGCTGCACTCCGAGAAACTGCACGTG
GACCCTGACAACTTCAAGCTCCTCTCCGACTGCCTGACCATCGTGGTGGCTGCTCGGTTC
GGCGAAGCC---TTCACTGCAGAAGTCCAGGCAGCTTTCCAGAAGTTCCTGGCCGTGGTG
GTGTCCTCCCTGGGAAGACAGTACCAC
[107] Gasterosteus_aculeatus_MN_Hbb5
ATG---GTTGCATGGACAGAATTCGAGCGCGCCACCATCAAGGACATCTTCTCCAAGATC
GACTACGATGTCGTAGGTCCTGCAGCTCTGTGCAGGTGTCTGGTCGTCTACCCCTGGACT
CAGAGGTATTTCGGTAACTTTGGAAACCTCTACAACGCCGCAGCAATTATGGGAAATCCG
CTGGTTGCAAAACACGGAACAACTATCCTCAACGGACTGGAGCGGGCTGTGAAGGACATG
GACAACATCAAGACAACCTACGCCGAGCTGAGCGTGCTGCACTCCGAGAAACTGCACGTG
GACCCCGACAACTTCAAGCTCCTCTCCGACTGCCTGACCATCGTGGTTGCTGCTCAGTTC
GGTAAAGCC---TTCACTGCAGAAGTCCAGGCAGCTTTCCAGAAGTTCCTGGCCGTGGTG

GTGTCCTCCCTGGGAAGACAGTACCAC
[108] Gasterosteus_aculeatus_MN_Hbb6
ATG---GTTGCATGGACAGAATTCGAGCGCGCCACCATCAAGGACATCTTCTCCAAGATC
GACTACGATGTCGTAGGTCCTGCAGCTCTGTGCAGGTGTCTGGTCGTCTACCCCTGGACT
CAGAGGTATTTCGGTAACTTCGGAAACCTCTACAACGCCGCAGCAATTATGGGAAATCCG
CTGGTGGCAAAACACGGAACAACAATCCTGTACGGACTGGAGCGGGCTGTGAAGGACATG
GACAACATCAAGACAACCTACGCCGAGCTGAGCGTGCTGCACTCCGAGAAACTGCACGTG
GACCCCGACAACTTCAAGCTCCTCTCCGACTGCCTGACCATCGTGGTGGCTGCTCGGTTC
GGCGAAGCC---TTCACTGCAGAAGTCCAGGCAGCTTTCCAGAAGTTCCTGGCCGTGGTG
GTGTCCTCCCTGGGAAGACAGTACCAT
[109] Tetraodon_nigroviridis_LA_Hbb1
ATG---GTTGTGTGGACTGACCAGGAGCGCGCCATCATCGACAACATCTTCTCCAACCTG
GACTACGAGGATGTCGGCTCCAAGGCTCTGATCAGGTGTCTGATCGTTTACCCCTGGACC
CAGAGATACTTCAGCAGCTTCGGCAACCTCTACAATGCAGAGGCCATCAGAAATAACCCC
AACGTCGCAAAACACGGTGTCACGGTGCTGCACGGACTGGACAGGGCTCTGAAGAACATG
GACAACATCAAGGAGGAATACAAGAAG------------CACTCCGAGAAGCTGCACGTC
GACCCCGACAACTTCAAACTGCTCTCTGACTGCCTGACCGTCGTCATCGCCGGCAAACTG
GGAAGCAAG---TTCACACCTGAGTACCAGGCTGCCCTCCAGAAGTTCCTGGCCGTGGTC
GTCTCAGCTCTGGGAAGACAGTACCAC
[110] Oreochromis_niloticus_MN_Hbb1
ATG---GTCGAGTGGACAGATGCTGAGCGCAAGGCCATCGCAAGCCTGTGGGGAAAGATC
GATGTGGGGGAAATCGGTCCCCAGGCCCTCGCCAGGCTTCTGATTGTGTATCCCTGGGCT
CAGAGATACTTCAAAGCCTTTGGAGACCTGTCCACCAATGCTGCCATTATGGGAAACCCC
AAAGTGGCACAGCACGGAAAGACCGTGATGGGTGGTCTGGAAAATGCTGTGAAGAACTTG
GACAACATCAAGCAGACCTACGCCAAACTGAGCGTTATGCACTCTGAGAAGCTCCACGTG
GATCCCGATAACTTCAGGGTGCTCGCTGAATGCATCAGCCTGTGTGTGGCCGCTAAGTTT
GGTCCCAGC---GTCTTCACCGCTGNCCAGGAAGCCTGGCAGAAGTTTTTGGCCGTGGTC
GTTTCTGCCCTGGGTAGACAGTACCAC
[111] Oreochromis_niloticus_MN_Hbb3
ATG---GTTGTGTGGACAGACTTTGAGCGCACCACGATCCAGGACATCTTCTCCAAGATC
GACTATGCAGTCGTTGGGCAAGCAGCTTTTTCCAGGTGTTTGACTGTCTACCCCTGGACT
CAGAGGTATTTTGGTCAATTTGGAAACCTCTACAATGCTGCTGCTATCGCATCAAATCCA
AAGGTTGCTGCTCATGGAAAAGTTATCATGGAAGCTCTGGAAAAAGCAGTGAAGGACATG
GACAACATCAAGGCCACATATGCAGAGCTGAGCGCGCTGCACTCTGAGAAACTGCAGGTG
GACCCTGACAATTTCATGCTCCTGGGTGACTGCTTGACCATTGTGGTTGCTTCCCAGTTG
GGTAAAAAC---TTCACTGCTGAGGTTCATGCAGCTTTCCAGAAGTTCCTGGCAGTGGTG
GTGTCCTCCCTCAGAAGACAGTACTAC
[112] Oreochromis_niloticus_MN_Hbb6
ATG---GTCGAGTGGACAGATGCTGAGCGCAGTGCCATCACAAGCCTGTGGGGAAAGATT
GATGTGGGGGAAATCGGTCCCCAGGCCCTCACCAGGCTTCTGATTGTGTATCCCTGGGCT
CAGAGATACTTCCAGTCCTTTGGAGACCTGTCCACCAATGCCGCCATTATGGGAAACCCC
AAAGTGGCACAGCACGGAAGGACCGTGATGGGTGGTCTGGAAAATGCTGTGAAGAACTTG
GACAACATCAAGAACACCTATGCCAACCTGAGCCATATGCACTCCGAGAAACTCCATGTG
GATCCCGATAACTTCAGGGTGCTCGCTGAATGCATCAGCCTGTGCGTTGCCGCTAAGTTT
GGCCCCAGCGTCTTCACCGCTGACGCCCAGGAAGCCTGGCAGAAGTTTTTGGCCGTGGTC
GTTTCTGCCCTGGGTAGACAGTACCAC
[113] Oreochromis_niloticus_MN_Hbb8
ATG---GTTGCATGGACAGACTTCGAGCGAGCCACAATCAAGGACATCTTCTCCAAGATC
GACTATGAAGTCGTTGGCCCAGCAGCTATTTCCAGGTGTCTGATTGTCTACCCCTGGACT
CAGAGGTATTTCGCTGGCTTTGGAAACCTCTACAATGCTGCTGCCATCACATCAAATCCA
AAAGTTGCTGCTCACGGAAAAGTCGTCATGCAAGGTCTGGAAAAAGCTGTGAAGAACATG
GACAACATCAAGGCCACATTTACAGAGCTGAGCACGCTGCACTCTGAGAAACTGCAGGTG
GACCCTGACAATTTCATGCTCCTGGGCGACTGCCTGGCCATTGTGGTTGCTTCTCAGTTG
GGTAAAGAC---TTCTCTCCCGAGGTCCATGCAGCTTTCCAGAAGTTCCTGGCAGTGGTG
GTGTCCTCCCTGAGGAGGCAGTACTAT
[114] Oreochromis_niloticus_LA_Hbb1
ATG---GTTGAGTGGAGCGAAAAGGAACGCAGCATCATCACCAACATCTTTTCCAACCTG

GACTATGAAGATGTTGGCCCCAAGGCTCTGGTCAGGTGTCTGGTCGTTTACCCCTGGACT
CAGAGGTATTTCGCCTCCTTCGGTAACCTCTACAACGCCGAGGCCATCAGCACCAACCCG
AAAGTCGCTGCCCACGGAATCAAAGTGCTCCACGGTCTGGACCGCGCTGTGAAGAACATG
GACAACATTAAGGCCACCTACGCCGAGCTGAGCGTCCTGCACTCAGAGAAGCTGCACGTC
GACCCCGACAACTTCAAGCTGCTTTCTGACTGCCTGACCATTGTTGTTGCTGGAAAACTG
GGTTCCGCT---TTCACCCCAGAGGTTCAGGCTACCTTCCAGAAGTTCTTGGCCGTAGTG
GTGTCCGCTCTCGGAAAGCAGTACCAC
[115] Oreochromis_niloticus_MN_Hbb5
ATG---GTTAAGTGGACGGAACTGGAGCGCAGCACCGTGAAAGCTATCTGGGAAAAAGTT
GATATCGATGAGATTGGGCCACAAATTTGGGCAAGAGTCTTGATTGTCTACCCCTGGACT
GAGCGATATTTTGGTTCTTTTGGAGATCTCTTCACCATCACTGCTATTTTGAAAAACGAC
AAAGTGGCGGCTCACGGAAAGGTGGTGCTGAAGGCTCTGGACAAAGCAGTGAACAACATG
GACAACATGAAACGGACGTATGCTGATCTGAGCCAGTTGCATTTTCAGAAACTCGAAGTG
GATCCTGATAGCTTCAGGCTGTTGGCAGACTGTATCACTATCACAATCGCATGCAAACTC
AAGTCAGCC---CTGAGCCCCCAGAGTCAAGCTATCTGGCAGAAGTTTCTATATGCTGTG
GTCGACGCTATGAGCAGTCAGTACCGC
[116] Salmon_chr3_MN_Hbb0
ATG---GTTGACTGGACAGACGCCGAGAAGAGCACCATCAGTGCTGTCTGGGGAAAAGTA
GATATCAATGAGGTCGGACCACTGGCTCTGGCAAGAGTCCTGATCGTCTACCCCTGGACT
CAGCGTTATTTCGGCTCTTTCGGAGATGTGTCTACTCCCGCAGCAATCATGGGCAACCCC
AAAGTTGCTGCTCACGGCAAGGTCGTGTGTGGAGCTCTGGATAAAGCTGTGAAGAACATG
GGCAACATCTTGGCCACATACAAGTCACTGAGCGAGACACACGCCAACAAACTCTTCGTC
GACCCTGACAATTTCAGGGTGTTGGCTGACGTCCTCACAATTGTCGTTGCCGCCAAGTTC
GGAGCCTCT---TTCACTCCTGAAATCCAGGCAACCTGGCAGAAGTTCATGAAAGTGGTT
GTCGCAGCCATGGGCAGCCGGTACTTC
[117] Salmon_chr3_MN_Hbb1
ATG---GTCGACTGGACAGATGCTGAGCGCAGCGCCATCGTAGGCCTGTGGGGAAAGATC
AGCGTGGATGAGATCGGACCCCAGGCCCTGGCCAGACTTCTGATCGTGTCTCCATGGACT
CAGAGGCACTTTAGCACCTTCGGCAACCTGTCCACACCCGCTGCCATCATGGGTAACCCC
GCCGTGGCCAAGCACGGAAAGACCGTGATGCACGGACTGGACAGAGCTGTGCAGAACCTG
GATGACATCAAAAACGCCTATACTGCCCTGAGTGTGATGCACTCCGAGAAACTGCACGTG
GATCCCGACAACTTCAGGCTCCTCGCCGACTGCATCACCGTGTGCGTGGCAGCCAAGCTC
GGTCCCACCGTTTTCAGTGCTGATATTCAGGAAGCCTTCCAGAAGTTCCTGGCTGTCGTT
GTGTCCGCTCTTGGCAGACAGTACCAC
[118] Salmon_chr3_MN_Hbb2
ATG---GTTGACTGGACAGACGCCGAGAAGAGCACCATCAGTGCTGTCTGGGGAAAAGTA
GATATCAATGAGGTCGGACCACTGGCTCTGGCAAGAGTCCTGATCGTCTATCCCTGGACT
CAGCGTTATTTCGGCTCTTTTGGAGACGTGTCCACTCCCGCAGCAATCATGGGCAACCCC
AAAGTTGCTGCTCACGGCAAGGTCGTGTGTGGAGCTCTGGATAAAGCTGTGAAGAACATG
GGCAACATCTTGGCCACATACAAGTCACTGAGCGAGACCCACGCCAACACACTCTTCGTC
GACCCTGACAATTTCAGGGTGTTGGCTGACGTCCTCACAATTGTCATTGCCGCCAAGTTC
GGAGCCTCT---TTCACTCCTGAAATCCAGGCAACCTGGCAGAAGTTCATGAAAGTGGTT
GTCGCAGCCATGGGCAGCCGGTACTTC
[119] Salmon_chr3_MN_Hbb3
ATG---GTTGACTGGACAGACGCCGAGAAGAGCACCATCAGTGCTGTCTGGGGCAAAGTA
GATATCAATGAGGTCGGACCACTGGCTCTGGCAAGAGTCCTGATCGTCTACCCCTGGACT
CAGCGTTATTTCGGCTCTTTCGGAGACGTGTCCACTCCCGCAGCAATCATGGGCAACCCC
AAAGTTGCTGCTCACGGCAAGGTCGTGTGTGGAGCTCTGGATAAAGCTGTGAAGAACATG
GGCAACATCTTGGCCACATACAAGTCACTGAGCGAGACCCACGCCAACAAACTCTTCGTC
GACCCTGACAATTTCAGGGTGTTGGCTGACGTCCTCACAATTGTCATTGCCGCCAAGTTC
GGAGCCTCT---TTCACTCCTGAAATCCAGGCAACCTGGCAGAAGTTCATGAAAGTGGTT
GTCGCAGCCATGGGCAGCCGGTACTTC
[120] Salmon_chr3_MN_Hbb4
ATG---GTCGACTGGACAGATGCTGAGCGCAGCGCCATCGTAGGCCTGTGGGGAAAGATC
AGCGTGGATGAGATCGGACCCCAGGCCCTGGCCAGACTTCTGATCGTGTCTCCGTGGACT
CAGAGGCACTTTAGCACCTTCGGCAACCTGTCCACACCCGCTGCCATCATGGGTAACCCC
GCCGTGGCCAAGCACGGAAAGACCGTGATGCACGGACTGGACAGAGCTGTGCAGAACCTG

GATGACATCAAAAACGCCTATACTGCCCTGAGTGTGATGCACTCCGAGAAACTGCACGTG
GATCCCGACAACTTCAGGCTCCTCGCCGACTGCATCACCGTGTGCGTGGCCGCCAAGCTC
GGTCCCGCCGTTTTCAGTGCTGATATTCAGGAAGCCTTCCAGAAGTTCCTGTCTGTCGTT
GTGTCCGCTCTTGGCAGACAGTACCAC
[121] Salmon_chr3_MN_Hbb5
ATG---GTTGACTGGACAGACGCCGAGAAGAGCACCATCAGTGCTGTCTGGGGCAAAGTA
GATATCAATGAGGTCGGACCACTGGCTCTGGCAAGAGTCCTGATCGTCTACCCCTGGACT
CAGCGTTATTTCGGCTCTTTCGGAGACGTGTCCACTCCCGCAGCAATCATGGGCAACCCC
AAAGTTGCTGCTCACGGCAAGGTCGTGTGTGGAGCTCTGGATAAAGCTGTGAAGAACATG
GGCAACATCTTGGCCACATACAAGTCACTTAGCGAGACACACGCCAACAAACTCTTCGTC
GACCCTGAAAATTTCAGGGTGTTGGCTGACGTCCTCACAATTGTCGTTGCCGCCAAGTTC
GGAGCCTCT---TTCACTCCTGAAATCCAGGCAACCTGGCAGAAGTTCATGAAAGTTGTT
GTCGCAGCCATGGGCAGTCGGTACTTC
[122] Salmon_chr3_MN_Hbb6
ATG---GTCCAGTGGACAGACTTTGAGCGCGCCACCATTCAGAGCGTCTTCGAGAAGATG
GACTACGATGACGTAGGCCCCGCGGCTCTTTCCAGGTGTCTGGTCGTGTACCCCTGGACC
CAGAGGTATTTCGGTAACTTTGGAAACCTGTACAACGCCGCTGCCATCCAGGGAAACCCA
ATGGTCGCCGCTCACGGAAAGACCGTCCTGCGCGGACTGGACCGGGCTGTCAAGAACATG
GATGACATCAAGGCCACCTACGCAGAGCTGAGCGTGCTGCACTCCGAGAAACTGCACGTG
GATCCAGACAACTTCCGGCTGCTGGCTGACTGCCTTACTATTGTCGTTGCTGCGAGAATG
GGTGCTGAC---TTCACCGCTGATGTCCAGGGCGCTTTCCAGAAGTTCCTGGCCGTCGTG
GTGAGCTCCCTGGGCAGACAGTACCAC
[123] Salmon_chr3_MN_Hbb7
ATG---GTTGAGTGGACAGACTTTGAGCGCGCCACAATCCAAAGCATCTTCTCAAAGATG
GACTACGATGACGTGGGCCCCGCGGCTCTTTCCAGGTGTCTGGTCGTGTACCCCTGGACC
CAGAGGTATTTCGGTAACTTCGGAAACCTGTACAACGCCGCTGCCATCCAGGGAAACCCA
ATGGTCGCCGCTCACGGAAAGACCGTCCTGCGCGGACTGGACCGGGCTGTCAAGAACATG
GATGACATCAAGGCCACCTACGCAGAGCTGAGCGTGCTGCACTCCGAGAAACTGCACGTG
GATCCCGACAATTTCAGGCTGCTGGCTGACTGCCTTACTATTGTCGTTGCTGCGAGAATG
GGTGCTGAC---TTCACCGCTGATGTTCAGGGCGCTTTCCAGAAGTTCCTGGCCGTCGTG
GTGAGCTCCCTGGGCAGACAGTACCAC
[124] Salmon_chr6_MN_Hbb0
ATG---GTTCAGTGGACAGACTTCGAGCGCCAAACAATCCAGAGCATCTTCGGGAAGATG
GACTACGATGACGTGGGCCCCGCGGCTCTTTCCAGGTGTCTGATCGTGTACCCCTGGACC
CAGAGGTATTTTGGTAACTTCGGAAACCTGTCCAACGCCGCTGCCATCCAGGGAAACCCC
AAGGTCGCCGCTCACGGAAAGACTGTCCTGCAAGGACTGGTCCGGGCTGTCAAGAACATG
GATGACATCAAGGCCACCTTCACAGAGCTTAGCGTGCTGCACTCCGAGAAATTGCGCGTG
GATCCCGACAATTTCCGGCTGCTGGCTGACTGCTTGACTATTGTCGTTGCTGCAAGAATG
GGTGCTGAC---TTCACCGCCGACGTCCAGGGGGCTTTCCAGAAGTTCCTTGCCATCGTG
GTGTGCTCCCTGTGCAGACAGTACCAC
[125] Salmon_chr6_MN_Hbb1
ATG---GTTCAGTGGACAGACTTTGAGCGCGCCACCATTCAGAGCGTCTTCGAGAAGATG
GACTACGATGTCGTAGGCCCCGCGGCTCTTTCCAGGTGTCTGGTCGTGTACCCCTGGACC
CAGAGGTATTTCGGTAACTTCGGAAACCTGTACAACGCCGCTGCCATCCAGGGAAACCCA
ATGGTCGCCGCTCACGGAAAGACTGTCCTGCACGGACTGGACCGGGCTGTCAAGAACATG
GATGACATCAAGGCCACCTACGCAGAGCTGAGCGTGCTGCACTCCGAGAAACTACACGTG
GATCCCGACAACTTCCGGCTGCTGGCTGACTGCCTTACTATTGTTGTTGCTGCGAGAATG
GGTGCTGAC---TTCACCGCTGATGTCCAGGGTGCTTTCCAGAAGTTCCTGGCTGTGGTG
GTGAGCTCCCTGGGCAAACAGTACCAC
[126] Salmon_chr6_MN_Hbb2
ATG---GTCGACTGGACAGATGCTGAGCGCAGTGCCATCTTAGGGCTGTGGGGAAATATC
AGCGTGGATGAGATCGGACCCCAGGCCCTGGCCAGACTTCTGATCGTGTTTCCATGGACT
CAGAGACACTTCAGCACCTTCGGCAACCTGTCCACACCCGCTGCCATCATGGGTAACCCC
GCCGTGGCCAAGCACGGAAAGGTCGTGATGCACGGACTGGACAGAGCTGTGCAGAACCTG
GATGACATCAAGAACGCCTATTCAGCACTGAGCGTGAAGCACTCCGAGAAACTGCACGTG
GATCCCGATAACTTCAGGCTCCTCGCTGAATGCATCACCGTGTGCGTGGCCGCCAAGCTC
GGTCCCGCCGTTTTCAGTGCTGATATTCAGGAAGCCTTCCAGAAGTTCCTGGCTGTCGTT

GTGTCCGCTCTTGGCAGACAGTACCAC
[127] Salmon_chr6_MN_Hbb3
ATG---GTTGACTGGACAGACGCTGAGAAGAGCACCATCAGTGCTGTCTGGGGCAAAGTA
GATATCAATGAGGTCGGACCACTGGCTCTGGCAAGAGTCCTGATCGTCTACCCCTGGACT
CAGCGTTATTTCGGCTCTTTCGGAGATGTGTCCACTCCCGCAGCAATCATGGGCAACCCC
AAAGTTGCTGCTCACGGCAAGGTCGTGTGTGGAGCTCTGGACAAAGCTGTGAAGAACATG
GGCAACATCTTGGCCACATACAAGTCACTGAGCGAGACCCACGCCAACAAACTCTTCGTC
GACCCTGACAATTTCAGGGTGTTGGCTGACGTCCTCACAATTGTCATTGCCGCCAAGTTC
GGAGCCTCT---TTCACTCCTGAAATCCAAGCAACCTGGCAGAAGTTCATGAAAGTGGTT
GTCGCAGCTATGGGCAGTCGGTACTTC
[128] Salmon_chr6_MN_Hbb4
ATG---GTCGACTGGACAGATGCTGAGCGCAGTGCCATCTTAGGCCTGTGGGGAAAGATC
AGCGTGGATGAGATCGGACCCCAGGCCCTGGCCAGACTTCTGATCGTGTCTCCATGGACT
CAGAGGCACTTTAGCACCTTCGGCAACCTGTCCACACCCGCTGCCATCATGGGTAACCCC
GCCGTGGCCAAGCACGGAAAGACCGTGATGCACGGACTGGACAGAGCTGTGCAGAACCTG
GATGACATCAAGAACGCCTATTCAGCACTGAGCGTGATGCACTCCGAGAAACTGCACGTG
GATCCCGATAACTTCAGGCTCCTCGCCGACTGCATCACCGTGTGCGTGGCCGCCAAGCTC
GGTCCCGCCGTTTTCAGTGCTGATATTCAGGAAGCCTTCCAGAAGTTCCTGGCTGTCGTT
GTGTCCGCTCTTGGCAGACAGTACCAC
[129] Salmon_chr6_MN_Hbb5
ATG---GCTGACTGGACAGACGCTGAGAAGAGCACCATCAGTGCTGTCTGGGGCAAAGTA
GATATCAATGAGGTCGGACCACTGGCTCTGGCAAGAGTCCTGATCGTCTACCCCTGGACT
CAGCGTTATTTCGGCTCTTTCGGAGATGTGTCCACTCCCGCAGCAATCATGGGCAACCCC
AAAGTTGCTGCTCACGGCAAGGTCGTGTGCGGAGCTCTGGATAAAGCTGTGAAGAACATG
GGCAACATCTTGGCCACATACAAGTCACTGAGCGAGACCCACGCCAACAAACTCTTCGTC
GACCCTGAAAATTTCAGGGTGTTGGCTGACGTCCTCACAATTGTCATTGCCGCCAAGTTC
GGAGCCTCT---TTCACTCCTGAAATCCAAGCAACCTGGCAGAAGTTCATGAAAGTGGTT
GTCGCAGCCATGGGCAGTCGGTACTTC
[130] Oreochromis_niloticus_MN_Hbb4
ATG---GTTGTGTGGACAGACTTTGAGCGCACCACGATCCAGGACATCTTCTCCAAGATC
GACTATGCGGTCGTTGGGCAAGCAGCTTTTTCCAGGTGTTTGATTGTGTACCCCTGGACT
CAGAGGTATTTTGGTGGATTTGGAAACCTCTACAATGCTGCTGCTATCACAACAAATCCA
AAGGTTGCTGCTCATGGAAAAGTTATCATGGAAGCTCTGGAAAAAGCAGTGAAGGACATG
GACAACATCAAGGCCACATATGCAGAGNNNNNNNNNNNNNNNNNNNNCAGTGAAGGACAT
GGACAACATCAAGGCCACCTCCTGGGTGACTGCTTGACCATTGTGGTTGCTTCTCAATTG
GGTAAAAAC---TTCACTGCTGAGGTTCACGCAGCTTTCCAGAAGTTCCTGGCAGTGGTG
GTGTCCTCCCTCAGAAGACAGTACTAC
[131] Xenopus_tropicalis_hbb1
ATG---GTCCATTGGACAGCTGACGAAAAGGCCGCAATTACTTCTGTATGGCAGAAGGTT
GATGTACAACAGGATGGTCATGAGGCCCTGAGCAGGCTTTTGGTTGTGTATCCCTGGACC
CAGAGATACTTCAGTAGTTTTGGAAACCTCTCCAATGCAGCTGCTATTGCTGGAAATGCC
AAGGTTAATGCCCATGGCAAGAAGGTTCTGGGAGCTGTTGGCAGCACAATCCAACATCTT
GATAATGTGAAGGCCTCTCTCCATGACCTCAGTGTGACCCATGCTTTAAAGCTGCATGTG
GACCCTGAGAACTTCAAGCGTTTTGGTGAAGTGCTGGTGATTGTCTTGGCTAAAAAACTG
GGAACTGCC---TTTACTCCTAAAATCCATGCTTCTTGGGAGAAATTCATTGCAGTGTTG
GTTGATGGCCTTAGCCAAGGCTATCAC
;
END

Additional Data 2 (separate file)

Molecular structures of the *Hb* α1/β1 tetramer for *Gadus morhua*

```
REMARK    File generated by Swiss-PdbViewer  4.00b0
REMARK    http://www.expasy.org/spdbv/
REMARK    File generated by Swiss-PdbViewer  4.00b0
REMARK    http://www.expasy.org/spdbv/
REMARK    File generated by Swiss-PdbViewer  4.00b0
REMARK    http://www.expasy.org/spdbv/
REMARK    File generated by Swiss-PdbViewer  4.00b0
REMARK    http://www.expasy.org/spdbv/
REMARK    File generated by Swiss-PdbViewer  4.00b0
REMARK    http://www.expasy.org/spdbv/
REMARK    File generated by Swiss-PdbViewer  4.00b0
REMARK    http://www.expasy.org/spdbv/
REMARK    File generated by Swiss-PdbViewer  4.00b0
REMARK    http://www.expasy.org/spdbv/
HETATM
HETATM
HETATM
HETATM
HETATM
HETATM
HETATM
HETATM
HETATM    9  CMA   d _   1      11.149 -17.787  10.479  1.00 31.13
HETATM
HETATM
HETATM
HETATM
HETATM
HETATM
HETATM
HETATM
HETATM
HETATM
HETATM
HETATM
HETATM
```

```
HETATM
HETATM
HETATM
HETATM
HETATM
HETATM
HETATM
HETATM
HETATM
HETATM
HETATM
HETATM
HETATM
HETATM
HETATM
HETATM
HETATM
HETATM
10CAAd_1 11CBAd_1 12CGAd_1 13O1Ad_1 14O2Ad_1 15C1Bd_1 16C2Bd_1
17C3Bd_1 18C4Bd_1 19CMBd_1 20CABd_1 21CBBd_1 22C1Cd_1 23C2Cd_1
24C3Cd_1 25C4Cd_1 26CMCd_1 27CACd_1 28CBCd_1 29C1Dd_1 30C2Dd_1
31C3Dd_1 32C4Dd_1 33CMDd_1 34CADd_1 35CBDd_1 36CGDd_1 37O1Dd_1
38O2Dd_1 39NAd_1 40NBd_1
 9.505 -20.646  10.738  1.00 34.12
10.664 -21.503  11.044  1.00 40.25
10.515 -22.937  10.467  1.00 42.05
11.045 -23.075   9.328  1.00 41.86
 9.862 -23.729  11.238  1.00 43.52
 8.227 -14.635  11.600  1.00 29.52
 8.571 -13.214  11.619  1.00 28.70
 7.452 -12.541  11.915  1.00 28.34
 6.404 -13.526  12.170  1.00 29.66
 9.943 -12.660  11.232  1.00 27.08
 7.222 -11.037  12.025  1.00 26.94
 8.104 -10.324  12.792  1.00 27.51
 4.138 -14.140  12.930  1.00 28.21
 2.892 -13.801  13.580  1.00 28.15
 2.291 -14.978  13.868  1.00 28.90
 3.113 -16.049  13.373  1.00 28.77
 2.438 -12.358  13.900  1.00 22.71
```

0.893 -15.150 14.530 1.00 29.95
0.621 -15.010 15.799 1.00 28.86
3.720 -18.424 13.079 1.00 30.70
3.454 -19.853 13.205 1.00 30.78
4.549 -20.499 12.753 1.00 29.67
5.538 -19.535 12.377 1.00 29.67
2.108 -20.429 13.695 1.00 28.47
4.830 -22.024 12.762 1.00 30.30
5.441 -22.343 14.173 1.00 32.69
5.385 -23.734 14.682 1.00 34.02
6.055 -24.094 15.720 1.00 36.19
4.592 -24.514 14.133 1.00 35.07
7.589 -17.573 11.775 1.00 31.40
6.904 -14.808 11.985 1.00 29.50
1CHAd_1 2CHBd_1 3CHCd_1 4CHDd_1 5C1Ad_1 6C2Ad_1 7C3Ad_1 8C4Ad_1
6.769 -19.890 11.886 1.00 29.14
9.085 -15.673 11.334 1.00 30.46
5.090 -13.234 12.515 1.00 29.37
2.827 -17.407 13.429 1.00 29.82
7.719 -18.946 11.604 1.00 31.31
9.073 -19.261 11.127 1.00 32.70
9.718 -18.094 10.987 1.00 32.66
8.795 -17.020 11.358 1.00 32.27

HETATM41NCd_1 HETATM42NDd_1 HETATM 43FE d_ 1 HETATM 44 CHA HEM _ 2 HETATM 45 CHB HEM _ 2 HETATM 46 CHC HEM _ 2 HETATM 47 CHD HEM _ 2 HETATM 48 C1A HEM _ 2 HETATM 49 C2A HEM _ 2 HETATM 50 C3A HEM _ 2 HETATM 51 C4A HEM _ 2 HETATM 52 CMA HEM _ 2 HETATM 53 CAA HEM _ 2 HETATM 54 CBA HEM _ 2 HETATM 55 CGA HEM _ 2 HETATM 56 O1A HEM _ 2 HETATM 57 O2A HEM _ 2 HETATM 58 C1B HEM _ 2 HETATM 59 C2B HEM _ 2 HETATM 60 C3B HEM _ 2 HETATM 61 C4B HEM _ 2 HETATM 62 CMB HEM _ 2 HETATM 63 CAB HEM _ 2 HETATM 64 CBB HEM _ 2 HETATM 65 C1C HEM _ 2 HETATM 66 C2C HEM _ 2 HETATM 67 C3C HEM _ 2 HETATM 68 C4C HEM _ 2 HETATM 69 CMC HEM _ 2 HETATM 70 CAC HEM _ 2 HETATM 71 CBC HEM _ 2 HETATM 72 C1D HEM _ 2 HETATM 73 C2D HEM _ 2 HETATM 74 C3D HEM _ 2 HETATM 75 C4D HEM _ 2 HETATM 76 CMD HEM _ 2 HETATM 77 CAD HEM _ 2 HETATM 78 CBD HEM _ 2 HETATM 79 CGD HEM _ 2 HETATM 80 O1D HEM _ 2 HETATM 81 O2D HEM _ 2 HETATM82NAHEM_2 HETATM83NBHEM_2 HETATM84NCHEM_2 HETATM85NDHEM_2 HETATM 86 FE HEM _ 2 HETATM 87 CHA HEM _ 3 HETATM 88 CHB HEM _ 3 HETATM 89 CHC HEM _ 3 HETATM

90 CHD HEM _ 3 HETATM 91 C1A HEM _ 3 HETATM 92 C2A HEM _ 3 HETATM
93 C3A HEM _ 3 HETATM 94 C4A HEM _ 3
4.252 -15.518 12.781 1.00 30.38
5.013 -18.260 12.589 1.00 30.68
5.847 -16.522 11.924 1.00 30.75
-5.954 19.739 25.579 1.00 27.67
-5.998 15.270 27.464 1.00 22.39
-4.487 13.411 23.233 1.00 20.42
-4.231 17.927 21.399 1.00 26.51
-6.138 18.704 26.475 1.00 26.42
-6.717 18.736 27.804 1.00 27.91
-6.676 17.491 28.312 1.00 26.38
-6.094 16.627 27.290 1.00 25.35
-7.198 16.993 29.680 1.00 27.02
-7.305 19.905 28.594 1.00 28.89
-7.087 21.301 28.195 1.00 33.81
-8.134 22.271 28.767 1.00 36.15
-8.966 21.769 29.567 1.00 36.08
-7.922 23.428 28.329 1.00 38.73
-5.696 14.410 26.429 1.00 20.24
-5.600 12.982 26.603 1.00 18.74
-5.142 12.492 25.461 1.00 19.62
-4.969 13.611 24.527 1.00 21.60
-5.954 12.255 27.925 1.00 18.35
-4.877 11.031 25.046 1.00 22.05
-4.617 10.088 25.992 1.00 22.82
-4.261 14.507 22.439 1.00 23.61
-3.685 14.400 21.087 1.00 22.22
-3.650 15.631 20.575 1.00 22.28
-4.166 16.555 21.544 1.00 23.19
-3.280 13.040 20.501 1.00 21.28
-3.142 16.043 19.189 1.00 21.53
-2.824 15.014 18.375 1.00 25.07
-4.638 18.841 22.381 1.00 29.80
-4.711 20.274 22.236 1.00 29.12
-5.222 20.765 23.364 1.00 30.73
-5.488 19.688 24.268 1.00 29.59
-4.229 21.028 20.985 1.00 29.13
-5.412 22.270 23.751 1.00 33.85
-4.224 22.789 24.590 1.00 38.61

2907	-4.160	24.301	24.729	1.00	41.04
2908	-3.567	24.964	23.836	1.00	41.52
2909	-4.707	24.731	25.794	1.00	43.25
2910	-5.776	17.388	26.177	1.00	24.68
2911	-5.307	14.792	25.153	1.00	21.02
2912	-4.559	15.855	22.695	1.00	25.10
2913	-5.146	18.489	23.639	1.00	28.08
2914	-5.500	16.670	24.323	1.00	25.71
2915	-6.357	17.548	-1.048	1.00	22.36
2916	-5.651	13.306	-3.356	1.00	21.09
2917	-6.965	10.865	0.668	1.00	17.24
2918	-7.554	15.087	2.993	1.00	18.84
2919	-6.054	16.578	-1.997	1.00	25.30
2920	-5.657	16.864	-3.382	1.00	26.74
2921	-5.483	15.698	-4.008	1.00	25.72
2922	-5.730	14.642	-3.041	1.00	23.54

2923	HETATM	95	CMA	HEM	_	3
2924	HETATM	96	CAA	HEM	_	3
2925	HETATM	97	CBA	HEM	_	3
2926	HETATM	98	CGA	HEM	_	3
2927	HETATM	99	O1A	HEM	_	3
2928	HETATM	100	O2A	HEM	_	3
2929	HETATM	101	C1B	HEM	_	3
2930	HETATM	102	C2B	HEM	_	3
2931	HETATM	103	C3B	HEM	_	3
2932	HETATM	104	C4B	HEM	_	3
2933	HETATM	105	CMB	HEM	_	3
2934	HETATM	106	CAB	HEM	_	3
2935	HETATM	107	CBB	HEM	_	3
2936	HETATM	108	C1C	HEM	_	3
2937	HETATM	109	C2C	HEM	_	3
2938	HETATM	110	C3C	HEM	_	3
2939	HETATM	111	C4C	HEM	_	3
2940	HETATM	112	CMC	HEM	_	3
2941	HETATM	113	CAC	HEM	_	3
2942	HETATM	114	CBC	HEM	_	3
2943	HETATM	115	C1D	HEM	_	3
2944	HETATM	116	C2D	HEM	_	3
2945	HETATM	117	C3D	HEM	_	3
2946	HETATM	118	C4D	HEM	_	3

2947 HETATM 119 CMD HEM _ 3
2948 HETATM 120 CAD HEM _ 3
2949 HETATM 121 CBD HEM _ 3
2950 HETATM 122 CGD HEM _ 3
2951 HETATM 123 O1D HEM _ 3
2952 HETATM 124 O2D HEM _ 3
2953 HETATM 125 NA HEM _ 3
2954 HETATM 126 NB HEM _ 3
2955 HETATM 127 NC HEM _ 3
2956 HETATM 128 ND HEM _ 3
2957 HETATM 129 FE HEM _ 3
2958 HETATM 130 CHA HEM _ 4
2959 HETATM 131 CHB HEM _ 4
2960 HETATM 132 CHC HEM _ 4
2961 HETATM 133 CHD HEM _ 4
2962 HETATM 134 C1A HEM _ 4
2963 HETATM 135 C2A HEM _ 4
2964 HETATM 136 C3A HEM _ 4
2965 HETATM 137 C4A HEM _ 4
2966 HETATM 138 CMA HEM _ 4
2967 HETATM 139 CAA HEM _ 4
2968 HETATM 140 CBA HEM _ 4
2969 HETATM 141 CGA HEM _ 4
2970 HETATM 142 O1A HEM _ 4
2971 HETATM 143 O2A HEM _ 4
2972 HETATM 144 C1B HEM _ 4
2973 HETATM 145 C2B HEM _ 4
2974 HETATM 146 C3B HEM _ 4
2975 HETATM 147 C4B HEM _ 4
2976 HETATM 148 CMB HEM _ 4
2977 -5.049 15.410 -5.469 1.00 24.91
2978 -5.462 18.273 -3.895 1.00 30.70
2979 -6.399 19.104 -4.680 1.00 36.33
2980 -5.650 20.318 -5.269 1.00 39.50
2981 -4.796 20.002 -6.132 1.00 40.89
2982 -5.974 21.427 -4.778 1.00 42.06
2983 -5.941 12.324 -2.439 1.00 19.20
2984 -5.954 10.916 -2.783 1.00 19.02
2985 -6.302 10.235 -1.679 1.00 19.88
2986 -6.560 11.223 -0.619 1.00 20.25

2987	-5.544	10.416	-4.180	1.00	15.69
2988	-6.415	8.724	-1.403	1.00	19.68
2989	-7.064	7.897	-2.252	1.00	22.53
2990	-7.316	11.815	1.614	1.00	16.36
2991	-7.845	11.494	2.941	1.00	15.23
2992	-8.037	12.651	3.595	1.00	16.21
2993	-7.582	13.728	2.695	1.00	17.49
2994	-8.181	10.064	3.420	1.00	12.49
2995	-8.533	12.940	5.011	1.00	14.25
2996	-9.433	12.311	5.727	1.00	12.75
2997	-7.356	16.110	2.073	1.00	22.49
2998	-7.511	17.522	2.338	1.00	23.17
2999	-7.145	18.187	1.227	1.00	23.05
3000	-6.793	17.244	0.225	1.00	21.55
3001	-7.951	18.115	3.708	1.00	24.77
3002	-7.233	19.741	1.046	1.00	24.47
3003	-8.614	20.082	0.449	1.00	28.02
3004	-9.091	21.474	0.319	1.00	29.87
3005	-9.561	21.796	-0.802	1.00	33.32
3006	-9.122	22.247	1.303	1.00	31.78
3007	-6.079	15.204	-1.824	1.00	22.93
3008	-6.324	12.495	-1.112	1.00	20.16
3009	-7.124	13.180	1.500	1.00	18.46
3010	-6.887	15.960	0.763	1.00	21.32
3011	-6.263	14.243	-0.055	1.00	23.44
3012	-19.680	-22.249	11.676	1.00	34.91
3013	-21.544	-17.744	11.868	1.00	34.06
3014	-17.418	-15.844	10.245	1.00	30.72
3015	-15.602	-20.305	9.837	1.00	33.32
3016	-20.538	-21.176	11.914	1.00	38.16
3017	-21.881	-21.290	12.453	1.00	40.13
3018	-22.389	-20.031	12.496	1.00	39.22
3019	-21.389	-19.119	12.002	1.00	37.73
3020	-23.813	-19.651	12.996	1.00	39.97
3021	-22.664	-22.511	12.928	1.00	41.97
3022	-22.418	-23.946	12.723	1.00	47.28
3023	-22.711	-24.872	13.909	1.00	49.17
3024	-23.555	-24.516	14.779	1.00	49.14
3025	-21.993	-25.924	13.893	1.00	51.05
3026	-20.587	-16.837	11.476	1.00	29.93

-20.709 -15.396 11.514 1.00 29.80
-19.562 -14.895 11.039 1.00 29.69
-18.682 -16.025 10.736 1.00 30.00
-21.998 -14.691 11.994 1.00 26.04

HETATM 149 CAB HEM _ 4 HETATM 150 CBB HEM _ 4 HETATM 151 C1C HEM _ 4 HETATM 152 C2C HEM _ 4 HETATM 153 C3C HEM _ 4 HETATM 154 C4C HEM _ 4 HETATM 155 CMC HEM _ 4 HETATM 156 CAC HEM _ 4 HETATM 157 CBC HEM _ 4 HETATM 158 C1D HEM _ 4 HETATM 159 C2D HEM _ 4 HETATM 160 C3D HEM _ 4 HETATM 161 C4D HEM _ 4 HETATM 162 CMD HEM _ 4 HETATM 163 CAD HEM _ 4 HETATM 164 CBD HEM _ 4 HETATM 165 CGD HEM _ 4 HETATM 166 O1D HEM _ 4 HETATM 167 O2D HEM _ 4 HETATM 168 NA HEM _ 4 HETATM 169 NB HEM _ 4 HETATM 170 NC HEM _ 4 HETATM 171 ND HEM _ 4 HETATM 172 FE HEM _ 4 ATOM173NSERA1 ATOM 174 CA SER A 1 10.699 2.949 2.174 1.00 0.89 ATOM 175 C SER A 1 11.753 3.501 3.148 1.00 0.89 ATOM 176 O SER A 1 11.643 4.620 3.658 1.00 0.89 ATOM 177 CB SER A 1 11.174 3.227 0.737 1.00 0.89 ATOM 178 OG SER A 1 11.401 4.627 0.556 1.00 0.89 ATOM 179 N LEU A 2 12.714 2.651 3.473 1.00 0.93 ATOM 180 CA LEU A 2 13.820 3.012 4.371 1.00 0.93 ATOM 181 C LEU A 2 14.840 3.874 3.639 1.00 0.93 ATOM 182 O LEU A 2 15.284 3.546 2.533 1.00 0.93 ATOM 183 CB LEU A 2 14.493 1.742 4.894 1.00 0.93 ATOM 184 CG LEU A 2 13.521 0.867 5.691 1.00 0.93 ATOM 185 CD1 LEU A 2 14.192 -0.462 6.022 1.00 0.93 ATOM 186 CD2 LEU A 2 13.075 1.564 6.978 1.00 0.93 ATOM 187 N THR A 3 15.231 4.944 4.302 1.00 1.00 ATOM 188 CA THR A 3 16.275 5.839 3.793 1.00 1.00 ATOM 189 C THR A 3 17.650 5.332 4.264 1.00 1.00 ATOM 190 O THR A 3 17.694 4.438 5.150 1.00 1.00 ATOM 191 CB THR A 3 16.013 7.283 4.261 1.00 1.00 ATOM 192 OG1 THR A 3 16.206 7.391 5.677 1.00 1.00 ATOM 193 CG2 THR A 3 14.680 7.835 3.743 1.00 1.00 ATOM 194 N PRO A 4 18.750 5.903 3.803 1.00 0.99 ATOM 195 CA PRO A 4 20.109 5.534 4.254 1.00 0.99 ATOM 196 C PRO A 4 20.279 5.730 5.772 1.00 0.99 ATOM 197 O PRO A 4 20.814 4.863 6.450 1.00 0.99 ATOM 198 CB PRO A 4 21.051 6.436 3.459 1.00 0.99 ATOM 199 CG PRO A 4 20.211 7.684 3.188 1.00 0.99 ATOM 200 CD PRO A 4 18.844 7.081 2.891 1.00 0.99 ATOM 201 N LYS A 5 19.636 6.782 6.293 1.00 0.84 ATOM 202 CA LYS A 5 19.636 7.088 7.733 1.00 0.84

-19.034 -13.460 10.814 1.00 28.70
-19.926 -12.503 10.528 1.00 30.09
-16.629 -16.911 9.933 1.00 31.42
-15.387 -16.782 9.188 1.00 31.27
-14.862 -18.008 9.096 1.00 32.16
-15.749 -18.930 9.783 1.00 32.13
-14.856 -15.440 8.665 1.00 28.99
-13.566 -18.453 8.375 1.00 32.77

-12.613 -17.618 8.013 1.00 32.82
-16.560 -21.205 10.261 1.00 34.12
-16.425 -22.647 10.244 1.00 34.60
-17.558 -23.146 10.771 1.00 35.57
-18.445 -22.071 11.108 1.00 33.49
-15.187 -23.393 9.721 1.00 32.21
-17.951 -24.625 10.964 1.00 38.04
-18.788 -25.201 9.804 1.00 42.96
-18.805 -26.720 9.889 1.00 46.71
-18.063 -27.372 9.091 1.00 48.48
-19.535 -27.159 10.842 1.00 48.85
-20.246 -19.832 11.666 1.00 36.63
-19.332 -17.199 11.013 1.00 31.66
-16.828 -18.237 10.301 1.00 32.54
-17.805 -20.872 10.784 1.00 34.88
-18.417 -18.995 11.303 1.00 31.79
9.362 3.537 2.418 1.00 0.89

ATOM 203 C LYS A 5 18.924 5.980 8.526 1.00 0.84 ATOM 204 O LYS A 5 19.495 5.432 9.453 1.00 0.84 ATOM 205 CB LYS A 5 18.957 8.439 7.974 1.00 0.84 ATOM 206 CG LYS A 5 19.054 8.859 9.443 1.00 0.84 ATOM 207 CD LYS A 5 18.359 10.200 9.663 1.00 0.84 ATOM 208 CE LYS A 5 18.467 10.634 11.124 1.00 0.84 ATOM 209 NZ LYS A 5 17.811 11.934 11.318 1.00 0.84 ATOM 210 N ASP A 6 17.780 5.530 7.995 1.00 0.89 ATOM 211 CA ASP A 6 17.006 4.433 8.609 1.00 0.89 ATOM 212 C ASP A 6 17.788 3.113 8.635 1.00 0.89 ATOM 213 O ASP A 6 17.975 2.494 9.678 1.00 0.89 ATOM 214 CB ASP A 6 15.698 4.201 7.849 1.00 0.89 ATOM 215 CG ASP A 6 14.733 5.386 7.906 1.00 0.89 ATOM 216 OD1 ASP A 6 14.712 6.076 8.946 1.00 0.89 ATOM 217 OD2 ASP A 6 14.092 5.599 6.855 1.00 0.89 ATOM 218 N LYS A 7 18.387 2.773 7.497 1.00 0.85 ATOM 219 CA LYS A 7 19.187 1.538 7.382 1.00 0.85 ATOM 220 C LYS A 7 20.398 1.557 8.325 1.00 0.85 ATOM 221 O LYS A 7 20.619 0.603 9.062 1.00 0.85 ATOM 222 CB LYS A 7 19.694 1.337 5.956 1.00 0.85 ATOM 223 CG LYS A 7 18.564 1.151 4.947 1.00 0.85 ATOM 224 CD LYS A 7 19.176 0.925 3.566 1.00 0.85 ATOM 225 CE LYS A 7 18.100 0.853 2.485 1.00 0.85 ATOM 226 NZ LYS A 7 18.726 0.689 1.164 1.00 0.85 ATOM 227 N ALA A 8 21.010 2.737 8.453 1.00 0.89 ATOM 228 CA ALA A 8 22.177 2.935 9.331 1.00 0.89 ATOM 229 C ALA A 8 21.787 2.826 10.809 1.00 0.89 ATOM 230 O ALA A 8 22.457 2.144 11.586 1.00 0.89 ATOM 231 CB ALA A 8 22.806 4.305 9.065 1.00 0.89 ATOM 232 N THR A 9 20.608 3.342 11.130 1.00 0.86 ATOM 233 CA THR A 9 20.081 3.323 12.509 1.00 0.86 ATOM 234 C THR A 9 19.661 1.914 12.948 1.00 0.86 ATOM 235 O THR A 9 19.928 1.513 14.078 1.00 0.86 ATOM 236 CB THR A 9 18.898 4.275 12.681 1.00 0.86 ATOM 237 OG1 THR A 9 17.858 3.886 11.798 1.00 0.86 ATOM 238 CG2 THR A 9 19.259 5.744 12.443 1.00 0.86 ATOM

239 N VAL A 10 19.144 1.132 11.998 1.00 0.88 ATOM 240 CA VAL A 10 18.701 - 0.252 12.260 1.00 0.88 ATOM 241 C VAL A 10 19.920 -1.169 12.438 1.00 0.88 ATOM 242 O VAL A 10 19.962 -1.960 13.380 1.00 0.88 ATOM 243 CB VAL A 10 17.779 - 0.771 11.142 1.00 0.88 ATOM 244CG1VALA10 17.356 -2.224 11.393 1.00 0.88 ATOM 245 CG2 VAL A 10 16.503 0.069 11.051 1.00 0.88 ATOM 246 N LYS A 11 20.913 - 0.988 11.574 1.00 0.80 ATOM 247 CA LYS A 11 22.160 -1.784 11.606 1.00 0.80 ATOM 248 C LYS A 11 22.926 -1.522 12.903 1.00 0.80 ATOM 249 O LYS A 11 23.358 -2.445 13.587 1.00 0.80 ATOM 250 CB LYS A 11 23.106 -1.407 10.464 1.00 0.80 ATOM 251 CG LYS A 11 22.509 -1.742 9.109 1.00 0.80 ATOM 252 CD LYS A 11 23.392 -1.423 7.909 1.00 0.80 ATOM 253 CE LYS A 11 22.716 -1.834 6.598 1.00 0.80 ATOM 254 NZ LYS A 11 23.602 -1.530 5.471 1.00 0.80 ATOM 255 N LEU A 12 22.939 -0.244 13.274 1.00 0.80 ATOM 256 CA LEU A 12 23.634 0.237 14.470 1.00 0.80

ATOM 257 C LEU A 12 22.982 -0.305 15.753 1.00 0.80
ATOM 258 O LEU A 12 23.653 -0.886 16.602 1.00 0.80
ATOM 259 CB LEU A 12 23.624 1.768 14.400 1.00 0.80
ATOM 260 CG LEU A 12 24.553 2.415 15.424 1.00 0.80
ATOM 261 CD1 LEU A 12 26.021 1.998 15.293 1.00 0.80
ATOM 262 CD2 LEU A 12 24.330 3.923 15.535 1.00 0.80
ATOM 263 N PHE A 13 21.650 -0.294 15.759 1.00 0.80
ATOM 264 CA PHE A 13 20.859 -0.781 16.903 1.00 0.80
ATOM 265 C PHE A 13 20.940 -2.305 17.023 1.00 0.80
ATOM 266 O PHE A 13 21.193 -2.847 18.101 1.00 0.80
ATOM 267 CB PHE A 13 19.398 -0.354 16.741 1.00 0.80
ATOM 268 CG PHE A 13 18.565 -0.808 17.940 1.00 0.80

ATOM 269CD1PHEA13 ATOM 270CD2PHEA13 ATOM 271CE1PHEA13 ATOM 272CE2PHEA13 ATOM 273 CZ PHE A 13 17.046 -1.601 20.128 1.00 0.80 ATOM 274 N TRP A 14 20.791 -2.970 15.881 1.00 0.76 ATOM 275 CA TRP A 14 20.852 -4.432 15.800 1.00 0.76 ATOM 276 C TRP A 14 22.239 -4.945 16.199 1.00 0.76 ATOM 277 O TRP A 14 22.340 -5.927 16.897 1.00 0.76 ATOM 278 CB TRP A 14 20.522 -4.913 14.389 1.00 0.76 ATOM 279 CG TRP A 14 20.342 -6.427 14.391 1.00 0.76

18.571 -0.051 19.104 1.00 0.80
17.804 -1.969 17.866 1.00 0.80
17.813 -0.447 20.199 1.00 0.80
17.045 -2.352 18.961 1.00 0.80

ATOM 280CD1TRPA14 ATOM 281CD2TRPA14 ATOM 282NE1TRPA14 ATOM 283CE2TRPA14 ATOM 284CE3TRPA14 ATOM 285CZ2TRPA14 ATOM 286CZ3TRPA14 ATOM 287CH2TRPA14 ATOM 288 N GLY A 15 23.272 -4.132 15.899 1.00 0.78 ATOM 289 CA GLY A 15 24.656 -4.437 16.302 1.00 0.78 ATOM 290 C GLY A 15 24.797 -4.481 17.832 1.00 0.78 ATOM 291 O GLY A 15 25.467 -5.352

18.378 1.00 0.78 ATOM 292 N ARG A 16 24.046 -3.597 18.490 1.00 0.68 ATOM 293
CA ARG A 16 24.041 -3.462 19.959 1.00 0.68 ATOM 294 C ARG A 16 23.213 -4.523
20.696 1.00 0.68 ATOM 295 O ARG A 16 23.602 -4.989 21.763 1.00 0.68 ATOM 296
CB ARG A 16 23.512 -2.085 20.362 1.00 0.68 ATOM 297 CG ARG A 16 24.434 -0.958
19.911 1.00 0.68 ATOM 298 CD ARG A 16 23.920 0.366 20.476 1.00 0.68 ATOM 299
NE ARG A 16 24.814 1.466 20.096 1.00 0.68 ATOM 300 CZ ARG A 16 24.834 2.078
18.903 1.00 0.68 ATOM 301 NH1 ARG A 16 23.972 1.705 17.989 1.00 0.68 ATOM 302
NH2 ARG A 16 25.690 3.059 18.659 1.00 0.68 ATOM 303 N MET A 17 22.101 -4.923
20.091 1.00 0.74 ATOM 304 CA MET A 17 21.119 -5.776 20.776 1.00 0.74 ATOM 305
C MET A 17 21.098 -7.219 20.277 1.00 0.74 ATOM 306 O MET A 17 20.704 -8.100
21.050 1.00 0.74 ATOM 307 CB MET A 17 19.755 -5.129 20.594 1.00 0.74 ATOM 308
CG MET A 17 18.673 -5.902 21.327 1.00 0.74 ATOM 309 SD MET A 17 17.047 -5.107
21.125 1.00 0.74 ATOM 310 CE MET A 17 16.708 -5.719 19.487 1.00 0.74
21.142 -7.330 13.847 1.00 0.76
19.311 -7.110 15.060 1.00 0.76
20.670 -8.558 14.132 1.00 0.76
19.590 -8.449 14.849 1.00 0.76
18.238 -6.685 15.786 1.00 0.76
18.708 -9.403 15.421 1.00 0.76
17.359 -7.631 16.336 1.00 0.76
17.616 -8.984 16.137 1.00 0.76
ATOM 311 N SER A 18 21.487 -7.439 19.027 1.00 0.71 ATOM 312 CA SER A 18
21.484 -8.752 18.338 1.00 0.71 ATOM 313 C SER A 18 21.851 -9.946 19.234 1.00 0.71
ATOM 314 O SER A 18 21.229 -10.990 19.159 1.00 0.71 ATOM 315 CB SER A 18
22.479 -8.757 17.178 1.00 0.71 ATOM 316 OG SER A 18 22.419 -9.973 16.435 1.00
0.71 ATOM 317 N GLY A 19 22.781 -9.680 20.183 1.00 0.69 ATOM 318CAGLYA19
23.219 -10.675 21.179 1.00 0.69 ATOM 319 C GLY A 19 22.081 -11.274 22.029 1.00
0.69 ATOM 320 O GLY A 19 22.127 -12.450 22.391 1.00 0.69 ATOM 321 N LYS A 20
21.038 -10.491 22.275 1.00 0.71 ATOM 322CALYSA20 19.886 -10.894 23.110 1.00
0.71 ATOM 323 C LYS A 20 18.616 -11.161 22.293 1.00 0.71 ATOM 324 O LYS A 20
17.519 -11.256 22.866 1.00 0.71 ATOM 325 CB LYS A 20 19.607 -9.776 24.120 1.00
0.71 ATOM 326 CG LYS A 20 20.770 -9.545 25.095 1.00 0.71
ATOM 327CDLYSA20 ATOM 328CELYSA20 ATOM 329NZLYSA20 ATOM 330 N
ALA A 21 18.778 -11.483 21.014 1.00 0.81 ATOM 331CAALAA21 17.638 -11.750
20.111 1.00 0.81 ATOM 332 C ALA A 21 16.717 -12.864 20.639 1.00 0.81 ATOM 333
O ALA A 21 15.502 -12.705 20.672 1.00 0.81 ATOM 334CBALAA21 18.140 -12.123
18.716 1.00 0.81 ATOM 335 N GLU A 22 17.330 -13.888 21.240 1.00 0.76 ATOM
336CAGLUA22 16.590 -15.025 21.824 1.00 0.76 ATOM 337 C GLU A 22 15.743 -
14.632 23.044 1.00 0.76 ATOM 338 O GLU A 22 14.615 -15.096 23.198 1.00 0.76
21.050 -10.747 26.003 1.00 0.71

19.879 -10.973 26.953 1.00 0.71
20.084 -12.111 27.855 1.00 0.71
ATOM 339CBGLUA22 ATOM 340CGGLUA22 ATOM 341CDGLUA22 ATOM
342OE1GLUA22 ATOM 343OE2GLUA22 ATOM 344 N LEU A 23 16.276 -13.715
23.852 1.00 0.81 ATOM 345CALEUA23 15.562 -13.201 25.033 1.00 0.81 ATOM 346 C
LEU A 23 14.406 -12.275 24.633 1.00 0.81 ATOM 347 O LEU A 23 13.286 -12.393
25.144 1.00 0.81
17.544 -16.150 22.228 1.00 0.76
18.290 -16.724 21.023 1.00 0.76
19.194 -17.882 21.446 1.00 0.76
20.163 -17.601 22.184 1.00 0.76
18.888 -19.020 21.039 1.00 0.76
ATOM 348CBLEUA23 ATOM 349CGLEUA23 ATOM 350CD1LEUA23 ATOM
351CD2LEUA23 ATOM 352 N ILE A 24 14.665 -11.464 23.618 1.00 0.85 ATOM
353CAILEA24 13.660 -10.541 23.054 1.00 0.85 ATOM 354 C ILE A 24 12.498 -11.317
22.435 1.00 0.85 ATOM 355 O ILE A 24 11.342 -10.966 22.648 1.00 0.85 ATOM 356
CB ILE A 24 14.312 -9.639 22.008 1.00 0.85
16.536 -12.467 25.962 1.00 0.81
15.861 -12.081 27.287 1.00 0.81
15.418 -13.309 28.083 1.00 0.81
16.775 -11.226 28.157 1.00 0.81
ATOM 357CG1ILEA24 ATOM 358CG2ILEA24 ATOM 359CD1ILEA24 ATOM 360 N
GLY A 25 12.865 -12.361 21.683 1.00 0.93 ATOM 361CAGLYA25 11.884 -13.216
20.996 1.00 0.93 ATOM 362 C GLY A 25 10.944 -13.924 21.966 1.00 0.93 ATOM
363OGLYA25 9.732 -13.946 21.751 1.00 0.93 ATOM 364 N ALA A 26 11.520 -14.361
23.084 1.00 0.89
15.299 -8.755 22.758 1.00 0.85
13.301 -8.809 21.183 1.00 0.85
16.253 -8.182 21.741 1.00 0.85
ATOM 365CAALAA26 ATOM 366CALAA26 ATOM 367OALAA26 ATOM
368CBALAA26 ATOM 369 N ASP A 27 10.353 -12.882 25.144 1.00 0.91
10.756 -15.040 24.142 1.00 0.89
9.800 -14.053 24.825 1.00 0.89
8.592 -14.263 24.862 1.00 0.89
11.714 -15.639 25.174 1.00 0.89
ATOM 370CAASPA27 ATOM 371CASPA27 ATOM 372OASPA27 ATOM
373CBASPA27 ATOM 374 CG ASP A 27 10.112 -9.893 27.426 1.00 0.91
9.619 -11.822 25.845 1.00 0.91
8.478 -11.233 24.999 1.00 0.91
7.358 -11.117 25.487 1.00 0.91

10.625 -10.753 26.269 1.00 0.91
ATOM 375OD1ASPA27 ATOM 376OD2ASPA27 ATOM 377NALAA28 ATOM
378CAALAA28 ATOM 379CALAA28 ATOM 380OALAA28 ATOM
381CBALAA28 ATOM 382NLEUA29 ATOM 383CALEUA29 ATOM
384CLEUA29 ATOM 385OLEUA29 ATOM 386CBLEUA29 ATOM
387CGLEUA29 ATOM 388CD1LEUA29 ATOM 389CD2LEUA29 ATOM
390NSERA30 ATOM 391CASERA30 ATOM 392CSERA30 ATOM
393OSERA30 ATOM 394CBSERA30 ATOM 395OGSERA30 ATOM
396NARGA31 ATOM 397CAARGA31 ATOM 398CARGA31 ATOM
399OARGA31 ATOM 400CBARGA31 ATOM 401CGARGA31 ATOM
402CDARGA31 ATOM 403NEARGA31 ATOM 404CZARGA31 ATOM
405NH1ARGA31 ATOM 406NH2ARGA31 ATOM 407NMETA32 ATOM
408CAMETA32 ATOM 409CMETA32 ATOM 410 O MET A 32 -0.740 -11.668 22.749
1.00 0.88
9.241 -10.383 28.179 1.00 0.91
10.640 -8.774 27.560 1.00 0.91
8.747 -11.025 23.710 1.00 0.93
7.764 -10.459 22.764 1.00 0.93
6.616 -11.417 22.439 1.00 0.93
5.449 -11.014 22.468 1.00 0.93
8.459 -10.025 21.472 1.00 0.93
6.939 -12.696 22.226 1.00 0.91
5.894 -13.707 22.024 1.00 0.91
4.984 -13.889 23.236 1.00 0.91
3.788 -13.804 23.130 1.00 0.91
6.420 -15.088 21.648 1.00 0.91
6.852 -15.220 20.190 1.00 0.91
6.872 -16.713 19.905 1.00 0.91
5.944 -14.497 19.187 1.00 0.91
5.633 -13.869 24.414 1.00 0.94
4.892 -14.045 25.674 1.00 0.94
3.886 -12.919 25.921 1.00 0.94
2.749 -13.172 26.327 1.00 0.94
5.873 -14.127 26.845 1.00 0.94
6.632 -15.333 26.736 1.00 0.94
4.267 -11.708 25.519 1.00 0.87
3.416 -10.514 25.648 1.00 0.87
2.264 -10.519 24.640 1.00 0.87
1.117 -10.301 25.018 1.00 0.87
4.247 -9.248 25.454 1.00 0.87

5.283 -9.132 26.567 1.00 0.87
6.158 -7.907 26.346 1.00 0.87
7.052 -7.773 27.506 1.00 0.87
6.827 -6.991 28.560 1.00 0.87
5.700 -6.300 28.677 1.00 0.87
7.771 -6.831 29.473 1.00 0.87
2.565 -10.961 23.419 1.00 0.88
1.554 -11.081 22.354 1.00 0.88
0.435 -12.043 22.779 1.00 0.88

ATOM 411CBMETA32 ATOM 412CGMETA32 ATOM 413SDMETA32 ATOM 414CEMETA32 ATOM 415NLEUA33 ATOM 416CALEUA33 ATOM 417 C LEU A 33 -1.021 -13.748 24.928 1.00 0.87 ATOM 418 O LEU A 33 -2.219 -14.016 24.911 1.00 0.87

2.219 -11.578 21.069 1.00 0.88
1.259 -11.466 19.882 1.00 0.88
1.925 -12.173 18.332 1.00 0.88
1.802 -13.912 18.689 1.00 0.88
0.827 -13.188 23.318 1.00 0.87
-0.106 -14.229 23.793 1.00 0.87

ATOM 419CBLEUA33 ATOM 420CGLEUA33 ATOM 421CD1LEUA33 ATOM 422CD2LEUA33 ATOM 423 N ALA A 34 -0.442 -12.976 25.847 1.00 0.90 ATOM 424CAALAA34 -1.167 -12.487 27.035 1.00 0.90 ATOM 425 C ALA A 34 -2.001 -11.228 26.764 1.00 0.90 ATOM 426 O ALA A 34 -3.169 -11.156 27.160 1.00 0.90 ATOM 427CBALAA34 -0.172 -12.234 28.170 1.00 0.90 ATOM 428 N VAL A 35 -1.406 -10.260 26.089 1.00 0.88 ATOM 429 CA VAL A 35 -2.047 -8.956 25.806 1.00 0.88 ATOM 430 C VAL A 35 -3.050 -9.063 24.642 1.00 0.88 ATOM 431 O VAL A 35 -4.050 -8.338 24.609 1.00 0.88 ATOM 432 CB VAL A 35 -0.980 -7.873 25.558 1.00 0.88 ATOM 433CG1VALA35 -1.619 -6.498 25.351 1.00 0.88 ATOM 434CG2VALA35 -0.000 -7.754 26.731 1.00 0.88 ATOM 435 N TYR A 36 -2.739 -9.911 23.673 1.00 0.87 ATOM 436CATYRA36 -3.594 -10.123 22.490 1.00 0.87 ATOM 437 C TYR A 36 -3.910 -11.625 22.387 1.00 0.87 ATOM 438 O TYR A 36 -3.439 -12.304 21.471 1.00 0.87 ATOM 439 CB TYR A 36 -2.908 -9.596 21.225 1.00 0.87 ATOM 440 CG TYR A 36 -2.459 -8.147 21.385 1.00 0.87

ATOM 441CD1TYRA36 ATOM 442CD2TYRA36 ATOM 443CE1TYRA36 ATOM 444CE2TYRA36 ATOM 445 CZ TYR A 36 -1.583 -5.541 21.628 1.00 0.87 ATOM 446 OH TYR A 36 -1.134 -4.267 21.722 1.00 0.87 ATOM 447 N PRO A 37 -4.832 -12.106 23.222 1.00 0.88 ATOM 448CAPROA37 -5.198 -13.540 23.305 1.00 0.88 ATOM 449 C PRO A 37 -5.795 -14.161 22.037 1.00 0.88 ATOM 450 O PRO A 37 -5.800 -15.398 21.891 1.00 0.88

0.692 -15.451 24.257 1.00 0.87

1.146 -16.406 23.140 1.00 0.87
1.972 -15.776 22.021 1.00 0.87
2.025 -17.490 23.756 1.00 0.87
-1.202 -7.890 21.916 1.00 0.87
-3.286 -7.108 20.984 1.00 0.87
-0.760 -6.582 22.037 1.00 0.87
-2.848 -5.797 21.109 1.00 0.87
ATOM 451CBPROA37 ATOM 452CGPROA37 ATOM 453CDPROA37 ATOM 454 N GLN A 38 -6.293 -13.376 21.117 1.00 0.81 ATOM 455CAGLNA38 -6.866 -13.872 19.842 1.00 0.81 ATOM 456 C GLN A 38 -5.802 -14.478 18.918 1.00 0.81 ATOM 457 O GLN A 38 -6.130 -15.267 18.038 1.00 0.81
-6.153 -13.626 24.493 1.00 0.88
-6.794 -12.242 24.559 1.00 0.88
-5.650 -11.314 24.165 1.00 0.88
ATOM 458CBGLNA38 ATOM 459CGGLNA38 ATOM 460CDGLNA38 ATOM 461OE1GLNA38 ATOM 462NE2GLNA38 ATOM 463 N THR A 39 -4.561 -14.030 19.082 1.00 0.87 ATOM 464CATHRA39 -3.416 -14.525 18.285 1.00 0.87 ATOM 465 C THR A 39 -3.089 -15.994 18.586 1.00 0.87 ATOM 466 O THR A 39 -2.513 -16.699 17.758 1.00 0.87
-7.652 -12.818 19.060 1.00 0.81
-6.841 -11.594 18.598 1.00 0.81
-6.563 -10.572 19.706 1.00 0.81
-6.028 -9.501 19.463 1.00 0.81
-6.904 -10.892 20.940 1.00 0.81
ATOM 467CBTHRA39 ATOM 468OG1THRA39 ATOM 469CG2THRA39 ATOM 470 N LYS A 40 -3.528 -16.450 19.756 1.00 0.81 ATOM 471CALYSA40 -3.384 -17.842 20.216 1.00 0.81 ATOM 472 C LYS A 40 -4.036 -18.872 19.293 1.00 0.81
-2.162 -13.678 18.520 1.00 0.87
-1.797 -13.752 19.898 1.00 0.87
-2.375 -12.229 18.075 1.00 0.87
ATOM 473 O LYS A 40 -3.600 -20.025 19.247 1.00 0.81
ATOM 474CBLYSA40 ATOM 475CGLYSA40 ATOM 476CDLYSA40 ATOM 477CELYSA40 ATOM 478NZLYSA40 ATOM 479 N THR A 41 -4.983 -18.409 18.483 1.00 0.82 ATOM 480CATHRA41 -5.653 -19.232 17.457 1.00 0.82 ATOM 481 C THR A 41 -4.673 -19.936 16.507 1.00 0.82 ATOM 482 O THR A 41 -4.949 -21.055 16.108 1.00 0.82
-3.984 -17.939 21.612 1.00 0.81
-3.079 -17.165 22.571 1.00 0.81
-3.664 -16.915 23.962 1.00 0.81
-4.220 -18.138 24.697 1.00 0.81

-4.793 -17.675 25.963 1.00 0.81
ATOM 483CBTHRA41 ATOM 484OG1THRA41 ATOM 485CG2THRA41 ATOM 486
N TYR A 42 -3.482 -19.352 16.326 1.00 0.80 ATOM 487CATYRA42 -2.444 -19.910
15.434 1.00 0.80 ATOM 488 C TYR A 42 -1.545 -20.959 16.099 1.00 0.80 ATOM 489
O TYR A 42 -0.807 -21.680 15.425 1.00 0.80
-6.628 -18.410 16.611 1.00 0.82
-5.934 -17.315 16.004 1.00 0.82
-7.823 -17.936 17.444 1.00 0.82
ATOM 490CBTYRA42 ATOM 491CGTYRA42 ATOM 492CD1TYRA42 ATOM
493CD2TYRA42 ATOM 494CE1TYRA42 ATOM 495CE2TYRA42 ATOM
496CZTYRA42 ATOM 497OHTYRA42 ATOM 498 N PHE A 43 -1.619 -21.048
17.420 1.00 0.78 ATOM 499CAPHEA43 -0.768 -21.982 18.177 1.00 0.78 ATOM 500 C
PHE A 43 -1.577 -23.053 18.911 1.00 0.78 ATOM 501 O PHE A 43 -1.048 -23.754
19.780 1.00 0.78
-1.570 -18.799 14.847 1.00 0.80
-2.381 -17.889 13.924 1.00 0.80
-2.719 -18.314 12.645 1.00 0.80
-2.756 -16.628 14.365 1.00 0.80
-3.447 -17.481 11.809 1.00 0.80
-3.473 -15.785 13.527 1.00 0.80
-3.819 -16.218 12.252 1.00 0.80
-4.508 -15.395 11.425 1.00 0.80
ATOM 502CBPHEA43 ATOM 503CGPHEA43 ATOM 504CD1PHEA43 ATOM
505CD2PHEA43 ATOM 506CE1PHEA43 ATOM 507CE2PHEA43 ATOM
508CZPHEA43 ATOM 509 N SER A 44 -2.763 -23.333 18.383 1.00 0.75 ATOM
510CASERA44 -3.651 -24.386 18.918 1.00 0.75 ATOM 511 C SER A 44 -3.109 -25.817
18.758 1.00 0.75 ATOM 512 O SER A 44 -3.593 -26.747 19.389 1.00 0.75 ATOM
513CBSERA44 -5.055 -24.275 18.324 1.00 0.75 ATOM 514OGSERA44 -5.001 -24.251
16.896 1.00 0.75 ATOM 515 N HIS A 45 -2.089 -25.972 17.915 1.00 0.65 ATOM
516CAHISA45 -1.361 -27.246 17.741 1.00 0.65 ATOM 517 C HIS A 45 -0.397 -27.572
18.898 1.00 0.65
ATOM 518OHISA45 ATOM 519CBHISA45 ATOM 520CGHISA45 ATOM
521ND1HISA45 ATOM 522CD2HISA45 ATOM 523CE1HISA45 ATOM
524NE2HISA45 ATOM 525 N TRP A 46 -0.073 -26.565 19.706 1.00 0.68 ATOM
526CATRPA46 0.718 -26.759 20.933 1.00 0.68
0.105 -21.200 19.158 1.00 0.78
0.879 -20.073 18.490 1.00 0.78
1.985 -20.417 17.732 1.00 0.78
0.494 -18.745 18.637 1.00 0.78
2.724 -19.426 17.111 1.00 0.78

1.243 -17.752 18.018 1.00 0.78
2.357 -18.093 17.257 1.00 0.78
0.103 -28.694 18.995 1.00 0.65
-0.655 -27.298 16.379 1.00 0.65
0.323 -26.142 16.151 1.00 0.65
0.019 -24.847 16.132 1.00 0.65
1.621 -26.262 15.894 1.00 0.65
1.129 -24.166 15.872 1.00 0.65
2.121 -25.041 15.727 1.00 0.65
ATOM 527 C TRP A 46 -0.233 -26.924 22.121 1.00 0.68
ATOM 528 O TRP A 46 -1.316 -26.338 22.163 1.00 0.68
ATOM 529CBTRPA46 ATOM 530CGTRPA46 ATOM 531CD1TRPA46 ATOM 532CD2TRPA46 ATOM 533NE1TRPA46 ATOM 534CE2TRPA46 ATOM 535CE3TRPA46 ATOM 536CZ2TRPA46 ATOM 537CZ3TRPA46 ATOM 538CH2TRPA46 ATOM 539NLYSA47 ATOM 540CALYSA47 ATOM 541 C LYS A 47 -0.125 -26.772 25.422 1.00 0.71 ATOM 542 O LYS A 47 -0.771 -26.621 26.458 1.00 0.71
1.631 -25.557 21.212 1.00 0.68
2.673 -25.256 20.132 1.00 0.68
3.116 -26.050 19.163 1.00 0.68
3.318 -24.022 19.989 1.00 0.68
4.003 -25.381 18.411 1.00 0.68
4.143 -24.167 18.884 1.00 0.68
3.269 -22.858 20.713 1.00 0.68
4.941 -23.071 18.498 1.00 0.68
4.055 -21.768 20.321 1.00 0.68
4.879 -21.893 19.207 1.00 0.68
0.235 -27.664 23.114 1.00 0.71
-0.501 -27.846 24.383 1.00 0.71
-0.252 -29.251 24.943 1.00 0.71
1.214 -29.480 25.317 1.00 0.71
1.429 -30.868 25.915 1.00 0.71
2.901 -31.088 26.272 1.00 0.71
3.353 -30.183 27.341 1.00 0.71
1.005 -26.120 25.168 1.00 0.82
1.609 -25.132 26.076 1.00 0.82
1.704 -23.774 25.376 1.00 0.82
2.782 -23.286 24.979 1.00 0.82
2.982 -25.635 26.536 1.00 0.82
3.856 -25.753 25.406 1.00 0.82

0.581 -23.095 25.352 1.00 0.82
0.543 -21.719 24.843 1.00 0.82
0.413 -20.818 26.073 1.00 0.82

ATOM 543CBLYSA47 ATOM 544CGLYSA47 ATOM 545CDLYSA47 ATOM 546CELYSA47 ATOM 547NZLYSA47 ATOM 548NSERA48 ATOM 549CASERA48 ATOM 550CSERA48 ATOM 551OSERA48 ATOM 552CBSERA48 ATOM 553OGSERA48 ATOM 554NLEUA49 ATOM 555CALEUA49 ATOM 556CLEUA49 ATOM 557 O LEU A 49 -0.658 -20.332 26.467 1.00 0.82

ATOM 558CBLEUA49 ATOM 559CGLEUA49 ATOM 560CD1LEUA49 ATOM 561CD2LEUA49 ATOM 562NSERA50 ATOM 563CASERA50 ATOM 564CSERA50 ATOM 565OSERA50 ATOM 566CBSERA50 ATOM 567OGSERA50 ATOM 568NPROA51 ATOM 569CAPROA51 ATOM 570CPROA51 ATOM 571OPROA51 ATOM 572CBPROA51 ATOM 573CGPROA51 ATOM 574CDPROA51 ATOM 575NGLYA52 ATOM 576CAGLYA52 ATOM 577CGLYA52 ATOM 578OGLYA52 ATOM 579NSERA53 ATOM 580CASERA53

-0.587 -21.599 23.824 1.00 0.82
-0.482 -20.312 23.013 1.00 0.82
-1.507 -20.372 21.900 1.00 0.82
-0.859 -19.114 23.863 1.00 0.82
1.489 -20.848 26.816 1.00 0.89
1.642 -20.100 28.066 1.00 0.89
2.946 -19.301 27.999 1.00 0.89
3.890 -19.761 27.308 1.00 0.89
1.651 -21.094 29.233 1.00 0.89
1.750 -20.388 30.471 1.00 0.89
3.025 -18.157 28.653 1.00 0.96
4.214 -17.281 28.651 1.00 0.96
5.467 -18.072 29.049 1.00 0.96
5.451 -18.847 29.999 1.00 0.96
3.932 -16.233 29.721 1.00 0.96
2.412 -16.097 29.695 1.00 0.96
1.934 -17.519 29.439 1.00 0.96
6.495 -17.930 28.209 1.00 0.96
7.814 -18.558 28.496 1.00 0.96
7.780 -20.097 28.555 1.00 0.96
8.750 -20.703 29.058 1.00 0.96
6.766 -20.743 28.022 1.00 0.91
6.725 -22.205 27.803 1.00 0.91

ATOM 581CSERA53 ATOM 582OSERA53 ATOM 583CBSERA53 ATOM 584OGSERA53 ATOM 585NPROA54 ATOM 586CAPROA54 ATOM 587CPROA54 ATOM 588OPROA54 ATOM 589CBPROA54 ATOM 590CGPROA54 ATOM 591CDPROA54 ATOM 592NASPA55 ATOM 593CAASPA55 ATOM 594CASPA55 ATOM 595OASPA55 ATOM 596CBASPA55 ATOM 597CGASPA55 ATOM 598OD1ASPA55 ATOM 599OD2ASPA55 ATOM 600NVALA56 ATOM 601CAVALA56 ATOM 602CVALA56 ATOM 603OVALA56 ATOM 604CBVALA56 ATOM 605CG1VALA56 ATOM 606CG2VALA56 ATOM 607NLYSA57 ATOM 608CALYSA57 ATOM 609 C LYS A 57 11.642 -20.295 22.549 1.00 0.81 ATOM 610 O LYS A 57 12.347 -19.499 21.928 1.00 0.81

ATOM 629CBHISA59 ATOM 630CGHISA59 ATOM 631ND1HISA59 ATOM 632CD2HISA59 ATOM 633CE1HISA59 ATOM 634NE2HISA59

7.949 -20.078 18.444 1.00 0.81
7.367 -19.449 17.175 1.00 0.81
7.500 -19.924 15.943 1.00 0.81
6.626 -18.351 17.094 1.00 0.81
6.851 -19.117 15.109 1.00 0.81
6.308 -18.139 15.825 1.00 0.81
7.881 -22.607 26.820 1.00 0.91
8.383 -21.856 26.063 1.00 0.91
5.359 -22.648 27.272 1.00 0.91
5.214 -22.369 25.874 1.00 0.91
8.258 -23.929 26.869 1.00 0.88
9.306 -24.436 25.960 1.00 0.88
9.049 -24.114 24.485 1.00 0.88
9.918 -23.564 23.815 1.00 0.88
9.257 -25.948 26.201 1.00 0.88
8.844 -26.051 27.665 1.00 0.88
7.823 -24.924 27.823 1.00 0.88
7.781 -24.233 24.066 1.00 0.82
7.363 -23.944 22.679 1.00 0.82
7.476 -22.467 22.306 1.00 0.82
7.946 -22.117 21.218 1.00 0.82
5.935 -24.429 22.406 1.00 0.82
5.780 -25.951 22.492 1.00 0.82
6.811 -26.656 22.471 1.00 0.82
4.631 -26.394 22.674 1.00 0.82
7.129 -21.625 23.265 1.00 0.85
7.202 -20.168 23.056 1.00 0.85

8.657 -19.665 23.023 1.00 0.85
8.976 -18.806 22.202 1.00 0.85
6.350 -19.365 24.048 1.00 0.85
4.894 -19.820 24.137 1.00 0.85
6.954 -19.387 25.424 1.00 0.85
9.530 -20.251 23.844 1.00 0.81
10.959 -19.879 23.856 1.00 0.81
ATOM 611CBLYSA57 ATOM 612CGLYSA57 ATOM 613CDLYSA57 ATOM
614CELYSA57 ATOM 615NZLYSA57 ATOM 616 N LYS A 58 11.246 -21.468 22.059
1.00 0.77 ATOM 617CALYSA58 11.758 -22.009 20.785 1.00 0.77 ATOM 618 C LYS
A 58 11.328 -21.162 19.586 1.00 0.77 ATOM 619 O LYS A 58 12.170 -20.661 18.842
1.00 0.77
11.709 -20.479 25.046 1.00 0.81
11.383 -19.739 26.346 1.00 0.81
12.308 -20.197 27.473 1.00 0.81
12.032 -19.406 28.753 1.00 0.81
12.905 -19.843 29.851 1.00 0.81
ATOM 620CBLYSA58 ATOM 621CGLYSA58 ATOM 622CDLYSA58 ATOM
623CELYSA58 ATOM 624NZLYSA58 ATOM 625 N HIS A 59 10.025 -20.891 19.497
1.00 0.81 ATOM 626CAHISA59 9.476 -20.066 18.405 1.00 0.81 ATOM 627CHISA59
9.981 -18.616 18.488 1.00 0.81 ATOM 628 O HIS A 59 10.269 -17.970 17.496 1.00 0.81
11.283 -23.443 20.574 1.00 0.77
11.876 -24.399 21.612 1.00 0.77
11.359 -25.828 21.430 1.00 0.77
9.849 -25.922 21.637 1.00 0.77
9.346 -27.280 21.437 1.00 0.77
ATOM 635 N GLY A 60 10.184 -18.154 19.733 1.00 0.90 ATOM 636CAGLYA60
10.711 -16.806 19.995 1.00 0.90 ATOM 637 C GLY A 60 12.060 -16.569 19.297 1.00
0.90 ATOM 638 O GLY A 60 12.286 -15.559 18.645 1.00 0.90 ATOM 639 N LYS A 61
12.923 -17.581 19.335 1.00 0.78 ATOM 640CALYSA61 14.223 -17.444 18.651 1.00
0.78 ATOM 641 C LYS A 61 14.150 -17.690 17.142 1.00 0.78 ATOM 642 O LYS A 61
15.012 -17.201 16.403 1.00 0.78
ATOM 643CBLYSA61 ATOM 644CGLYSA61 ATOM 645CDLYSA61 ATOM
646CELYSA61 ATOM 647NZLYSA61 ATOM 648 N THR A 62 13.130 -18.413 16.695
1.00 0.80 ATOM 649CATHRA62 12.864 -18.613 15.258 1.00 0.80 ATOM 650 C THR
A 62 12.295 -17.354 14.578 1.00 0.80 ATOM 651 O THR A 62 12.491 -17.090 13.410
1.00 0.80
15.322 -18.268 19.316 1.00 0.78
15.224 -19.778 19.086 1.00 0.78
16.294 -20.517 19.888 1.00 0.78

16.068 -20.318 21.390 1.00 0.78
15.918 -21.605 22.066 1.00 0.78
ATOM 652CBTHRA62 ATOM 653OG1THRA62 ATOM 654CG2THRA62 ATOM 655
N ILE A 63 11.559 -16.567 15.362 1.00 0.82 ATOM 656CAILEA63 10.981 -15.314
14.835 1.00 0.82 ATOM 657 C ILE A 63 12.058 -14.227 14.750 1.00 0.82 ATOM 658 O
ILE A 63 12.279 -13.626 13.700 1.00 0.82
11.961 -19.811 14.941 1.00 0.80
10.649 -19.547 15.422 1.00 0.80
12.490 -21.123 15.531 1.00 0.80
ATOM 659CBILEA63 ATOM 660CG1ILEA63 ATOM 661CG2ILEA63 ATOM
662CD1ILEA63 ATOM 663 N MET A 64 12.814 -14.101 15.840 1.00 0.82 ATOM
664CAMETA64 13.935 -13.156 15.930 1.00 0.82 ATOM 665 C MET A 64 15.071 -
13.489 14.965 1.00 0.82 ATOM 666 O MET A 64 15.709 -12.575 14.448 1.00 0.82
9.768 -14.841 15.655 1.00 0.82
10.159 -14.527 17.099 1.00 0.82
8.667 -15.901 15.592 1.00 0.82
9.138 -13.842 17.992 1.00 0.82
ATOM 667CBMETA64 ATOM 668CGMETA64 ATOM 669SDMETA64 ATOM
670CEMETA64 ATOM 671 N MET A 65 15.225 -14.773 14.636 1.00 0.79 ATOM
672CAMETA65 16.185 -15.169 13.586 1.00 0.79 ATOM 673 C MET A 65 15.763 -
14.622 12.205 1.00 0.79 ATOM 674 O MET A 65 16.603 -14.205 11.413 1.00 0.79
14.468 -13.062 17.355 1.00 0.82
13.499 -12.285 18.243 1.00 0.82
13.213 -10.544 17.764 1.00 0.82
11.555 -10.722 17.152 1.00 0.82
ATOM 675CBMETA65 ATOM 676CGMETA65 ATOM 677SDMETA65 ATOM
678CEMETA65 ATOM 679 N GLY A 66 14.433 -14.533 12.006 1.00 0.88 ATOM
680CAGLYA66 13.827 -13.957 10.790 1.00 0.88 ATOM 681 C GLY A 66 14.065 -
12.442 10.711 1.00 0.88 ATOM 682 O GLY A 66 14.336 -11.900 9.638 1.00 0.88
ATOM 683 N ILE A 67 14.100 -11.803 11.878 1.00 0.84 ATOM 684CAILEA67 14.395
-10.358 12.002 1.00 0.84 ATOM 685 C ILE A 67 15.878 -10.103 11.695 1.00 0.84
ATOM 686 O ILE A 67 16.217 -9.146 11.006 1.00 0.84 ATOM 687 CB ILE A 67
14.042 -9.839 13.409 1.00 0.84 ATOM 688CG1ILEA67 12.573 -10.120 13.764 1.00
0.84
16.419 -16.686 13.531 1.00 0.79
15.238 -17.451 12.932 1.00 0.79
15.314 -19.272 12.798 1.00 0.79
15.836 -19.747 14.433 1.00 0.79
ATOM 689CG2ILEA67 14.399 -8.353 13.602 1.00 0.84 ATOM 690CD1ILEA67 11.518
-9.408 12.912 1.00 0.84 ATOM 691 N GLY A 68 16.713 -11.052 12.149 1.00 0.87

ATOM 692CAGLYA68 18.167 -11.020 11.906 1.00 0.87 ATOM 693 C GLY A 68 18.457 -11.081 10.400 1.00 0.87 ATOM 694 O GLY A 68 19.161 -10.230 9.859 1.00 0.87 ATOM 695 N ASP A 69 17.713 -11.962 9.726 1.00 0.81 ATOM 696 CA ASP A 69 17.773 -12.105 8.260 1.00 0.81 ATOM 697 C ASP A 69 17.317 -10.818 7.555 1.00 0.81 ATOM 698 O ASP A 69 17.950 -10.356 6.608 1.00 0.81 ATOM 699 CB ASP A 69 16.902 -13.283 7.811 1.00 0.81 ATOM 700 CG ASP A 69 16.952 -13.475 6.291 1.00 0.81 ATOM 701 OD1 ASP A 69 18.067 -13.720 5.783 1.00 0.81 ATOM 702 OD2 ASP A 69 15.883 -13.295 5.672 1.00 0.81 ATOM 703 N ALA A 70 16.250 -10.224 8.075 1.00 0.88 ATOM 704 CA ALA A 70 15.703 -8.973 7.526 1.00 0.88 ATOM 705 C ALA A 70 16.706 -7.825 7.619 1.00 0.88 ATOM 706 O ALA A 70 16.937 -7.162 6.612 1.00 0.88 ATOM 707 CB ALA A 70 14.435 -8.594 8.275 1.00 0.88 ATOM 708 N VAL A 71 17.426 -7.741 8.740 1.00 0.87 ATOM 709 CA VAL A 71 18.499 -6.755 8.953 1.00 0.87 ATOM 710 C VAL A 71 19.620 -6.931 7.891 1.00 0.87 ATOM 711 O VAL A 71 20.013 -6.059 7.174 1.00 0.87 ATOM 712 CB VAL A 71 19.066 -6.832 10.384 1.00 0.87 ATOM 713CG1VALA71 20.187 -5.821 10.640 1.00 0.87 ATOM 714CG2VALA71 17.975 -6.581 11.429 1.00 0.87 ATOM 715 N THR A 72 19.990 -8.197 7.679 1.00 0.83 ATOM 716 CA THR A 72 21.051 -8.469 6.684 1.00 0.83 ATOM 717 C THR A 72 20.597 -8.216 5.238 1.00 0.83 ATOM 718 O THR A 72 21.382 -7.795 4.391 1.00 0.83 ATOM 719 CB THR A 72 21.583 -9.894 6.848 1.00 0.83 ATOM 720 OG1 THR A 72 20.530 -10.829 6.621 1.00 0.83 ATOM 721 CG2 THR A 72 22.199 -10.093 8.236 1.00 0.83 ATOM 722 N LYS A 73 19.291 -8.357 5.012 1.00 0.85 ATOM 723 CA LYS A 73 18.660 -8.179 3.688 1.00 0.85 ATOM 724 C LYS A 73 17.857 -6.877 3.599 1.00 0.85 ATOM 725 O LYS A 73 17.031 -6.705 2.685 1.00 0.85 ATOM 726 CB LYS A 73 17.715 -9.352 3.406 1.00 0.85 ATOM 727 CG LYS A 73 18.398 -10.716 3.327 1.00 0.85 ATOM 728 CD LYS A 73 19.489 -10.766 2.250 1.00 0.85 ATOM 729 CE LYS A 73 20.047 -12.180 2.090 1.00 0.85 ATOM 730 NZ LYS A 73 20.437 -12.753 3.387 1.00 0.85 ATOM 731 N MET A 74 18.245 -5.897 4.401 1.00 0.92 ATOM 732 CA MET A 74 17.542 -4.600 4.494 1.00 0.92 ATOM 733 C MET A 74 17.490 -3.780 3.203 1.00 0.92 ATOM 734 O MET A 74 16.681 -2.867 3.092 1.00 0.92 ATOM 735 CB MET A 74 18.123 -3.720 5.593 1.00 0.92 ATOM 736 CG MET A 74 17.666 -4.231 6.950 1.00 0.92 ATOM 737 SD MET A 74 18.267 -3.297 8.395 1.00 0.92 ATOM 738 CE MET A 74 20.010 -3.426 8.150 1.00 0.92 ATOM 739 N ASP A 75 18.377 -4.094 2.256 1.00 0.91 ATOM 740 CA ASP A 75 18.395 -3.384 0.963 1.00 0.91 ATOM 741 C ASP A 75 17.284 -3.829 0.002 1.00 0.91 ATOM 742 O ASP A 75 16.937 -3.112 -0.932 1.00 0.91 ATOM 743 CB ASP A 75 19.767 -3.482 0.293 1.00 0.91 ATOM 744 CG ASP A 75 20.835 -2.792 1.144 1.00 0.91 ATOM 745 OD1 ASP A 75 20.819 -1.542 1.183 1.00 0.91 ATOM 746 OD2 ASP A 75 21.639 -3.540 1.738 1.00 0.91 ATOM 747 N ASP A 76 16.771 -5.036 0.234 1.00 0.86 ATOM 748 CA ASP A 76 15.699 -5.617 -0.581 1.00 0.86 ATOM 749 C ASP A 76 15.001 -6.707 0.238 1.00 0.86 ATOM 750 O ASP A 76 15.101 -7.905 -0.045 1.00 0.86 ATOM 751 CB ASP A 76 16.295 -6.180 -1.877 1.00 0.86

ATOM 752 CG ASP A 76 15.216 -6.664 -2.851 1.00 0.86 ATOM 753OD1ASPA76 14.026 -6.741 -2.449 1.00 0.86 ATOM 754OD2ASPA76 15.631 -7.025 -3.966 1.00 0.86 ATOM 755 N LEU A 77 14.130 -6.231 1.111 1.00 0.83 ATOM 756 CA LEU A 77 13.341 -7.096 2.008 1.00 0.83 ATOM 757 C LEU A 77 12.372 -8.020 1.268 1.00 0.83 ATOM 758 O LEU A 77 12.174 -9.160 1.669 1.00 0.83 ATOM 759 CB LEU A 77 12.556 -6.242 3.001 1.00 0.83 ATOM 760 CG LEU A 77 13.478 -5.543 4.002 1.00 0.83 ATOM 761 CD1 LEU A 77 12.670 -4.544 4.821 1.00 0.83 ATOM 762 CD2 LEU A 77 14.130 -6.556 4.946 1.00 0.83 ATOM 763 N GLU A 78 11.884 -7.544 0.122 1.00 0.77 ATOM 764 CA GLU A 78 10.925 -8.310 -0.696 1.00 0.77 ATOM 765 C GLU A 78 11.560 -9.618 -1.184 1.00 0.77 ATOM 766 O GLU A 78 11.099 -10.701 -0.843 1.00 0.77 ATOM 767 CB GLU A 78 10.455 -7.485 -1.895 1.00 0.77 ATOM 768CGGLUA78 ATOM 769CDGLUA78 ATOM 770OE1GLUA78 ATOM 771OE2GLUA78 ATOM 772 N ARG A 79 12.741 -9.470 -1.787 1.00 0.69 ATOM 773CAARGA79 13.536 -10.616 -2.263 1.00 0.69 ATOM 774 C ARG A 79 14.135 - 11.428 -1.109 1.00 0.69 ATOM 775 O ARG A 79 14.080 -12.651 -1.114 1.00 0.69 ATOM 776CBARGA79 14.664 -10.135 -3.177 1.00 0.69 ATOM 777 CG ARG A 79 14.129 -9.503 -4.462 1.00 0.69 ATOM 778CDARGA79 13.427 -10.510 -5.368 1.00 0.69 ATOM 779 NE ARG A 79 12.936 -9.806 -6.564 1.00 0.69 ATOM 780 CZ ARG A 79 11.812 -9.090 -6.660 1.00 0.69 ATOM 781NH1ARGA79 10.997 -8.938 -5.622 1.00 0.69 ATOM 782NH2ARGA79 11.485 -8.530 -7.817 1.00 0.69 ATOM 783 N GLY A 80 14.541 -10.687 -0.062 1.00 0.85 ATOM 784 CA GLY A 80 15.202 -11.260 1.122 1.00 0.85 ATOM 785 C GLY A 80 14.298 -12.210 1.918 1.00 0.85 ATOM 786 O GLY A 80 14.712 -13.309 2.280 1.00 0.85 ATOM 787 N LEU A 81 13.029 -11.833 2.034 1.00 0.82 ATOM 788 CA LEU A 81 12.042 -12.586 2.834 1.00 0.82 ATOM 789 C LEU A 81 11.016 -13.319 1.965 1.00 0.82 ATOM 790OLEUA81 9.960 -13.738 2.462 1.00 0.82 ATOM 791 CB LEU A 81 11.303 -11.621 3.768 1.00 0.82 ATOM 792 CG LEU A 81 12.213 -10.866 4.742 1.00 0.82 ATOM 793 CD1 LEU A 81 11.369 -9.881 5.547 1.00 0.82 ATOM 794 CD2 LEU A 81 12.945 -11.820 5.691 1.00 0.82 ATOM 795 N LEU A 82 11.396 -13.641 0.736 1.00 0.73 ATOM 796CALEUA82 10.459 -14.282 -0.205 1.00 0.73

9.688 -6.236 -1.457 1.00 0.77
9.150 -5.469 -2.666 1.00 0.77
8.427 -6.096 -3.473 1.00 0.77
9.465 -4.263 -2.744 1.00 0.77

ATOM 797CLEUA82 ATOM 798OLEUA82 ATOM 799CBLEUA82 ATOM 800CGLEUA82 ATOM 801CD1LEUA82 ATOM 802CD2LEUA82 ATOM 803 N THR A 83 10.929 -16.480 0.728 1.00 0.72 ATOM 804 CA THR A 83 10.633 -17.849 1.214 1.00 0.72 ATOM 805CTHRA83 9.800 -17.836 2.501 1.00 0.72 ATOM 806OTHRA83 8.860 -18.615 2.656 1.00 0.72 ATOM 807 CB THR A 83 11.915 -18.646 1.475 1.00 0.72

ATOM 808 OG1 THR A 83 12.708 -17.961 2.449 1.00 0.72 ATOM 809 CG2 THR A 83 12.711 -18.868 0.184 1.00 0.72 ATOM 810 N LEU A 84 10.069 -16.833 3.336 1.00 0.75 ATOM 811CALEUA84 ATOM 812CLEUA84 ATOM 813OLEUA84 ATOM 814 CB LEU A 84 10.130 -15.665 5.494 1.00 0.75 ATOM 815CGLEUA84 9.711 -15.756 6.967 1.00 0.75 ATOM 816CD1LEUA84 9.997 -17.144 7.553 1.00 0.75 ATOM 817 CD2 LEU A 84 10.447 -14.693 7.783 1.00 0.75

ATOM 818NSERA85 ATOM 819CASERA85 ATOM 820CSERA85 ATOM 821OSERA85 ATOM 822CBSERA85 ATOM 823OGSERA85 ATOM 824NGLUA86 ATOM 825CAGLUA86 ATOM 826CGLUA86 ATOM 827OGLUA86 ATOM 828CBGLUA86 ATOM 829CGGLUA86 ATOM 830CDGLUA86 ATOM 831OE1GLUA86 ATOM 832OE2GLUA86 ATOM 833NLEUA87 ATOM 834CALEUA87 ATOM 835CLEUA87 ATOM 836OLEUA87 ATOM 837CBLEUA87 ATOM 838CGLEUA87 ATOM 839CD1LEUA87 ATOM 840CD2LEUA87 ATOM 841NHISA88 ATOM 842CAHISA88 ATOM 843CHISA88 ATOM 844OHISA88 ATOM 845CBHISA88 ATOM 846CGHISA88 ATOM 847ND1HISA88 ATOM 848CD2HISA88 ATOM 849CE1HISA88 ATOM 850NE2HISA88

7.691 -15.504 3.313 1.00 0.79
6.357 -15.013 2.928 1.00 0.79
5.447 -16.137 2.423 1.00 0.79
4.299 -16.237 2.839 1.00 0.79
6.493 -13.945 1.847 1.00 0.79
5.203 -13.530 1.398 1.00 0.79
6.012 -17.050 1.632 1.00 0.72
5.246 -18.197 1.107 1.00 0.72
4.877 -19.200 2.204 1.00 0.72
3.796 -19.782 2.183 1.00 0.72
6.016 -18.932 0.014 1.00 0.72
6.216 -18.053 -1.221 1.00 0.72
6.800 -18.832 -2.401 1.00 0.72
7.594 -19.766 -2.154 1.00 0.72
6.451 -18.476 -3.544 1.00 0.72
5.755 -19.301 3.200 1.00 0.70
5.526 -20.178 4.359 1.00 0.70
4.356 -19.659 5.216 1.00 0.70
3.524 -20.439 5.687 1.00 0.70
6.845 -20.298 5.136 1.00 0.70
6.790 -21.363 6.236 1.00 0.70
8.185 -21.878 6.570 1.00 0.70
6.297 -20.734 7.525 1.00 0.70
4.256 -18.338 5.321 1.00 0.74

3.159 -17.677 6.051 1.00 0.74
1.872 -17.588 5.225 1.00 0.74
0.787 -17.773 5.772 1.00 0.74
3.591 -16.283 6.508 1.00 0.74
4.571 -16.373 7.677 1.00 0.74
5.881 -16.570 7.588 1.00 0.74
4.266 -16.251 8.965 1.00 0.74
6.388 -16.576 8.813 1.00 0.74
5.389 -16.381 9.667 1.00 0.74
9.983 -15.646 0.327 1.00 0.73
8.776 -15.911 0.384 1.00 0.73
11.083 -14.394 -1.603 1.00 0.73
10.124 -15.007 -2.638 1.00 0.73
10.531 -14.561 -4.039 1.00 0.73
10.149 -16.541 -2.621 1.00 0.73
9.369 -16.664 4.617 1.00 0.75
7.914 -16.226 4.408 1.00 0.75
7.023 -16.654 5.134 1.00 0.75
ATOM 851NALAA89 ATOM 852CAALAA89 ATOM 853CALAA89 ATOM 854 O ALA A 89 -0.952 -18.806 3.188 1.00 0.79
ATOM 855CBALAA89 ATOM 856NPHEA90 ATOM 857CAPHEA90 ATOM 858CPHEA90 ATOM 859 O PHE A 90 -0.667 -22.849 2.943 1.00 0.64
ATOM 860CBPHEA90 ATOM 861CGPHEA90 ATOM 862CD1PHEA90 ATOM 863CD2PHEA90 ATOM 864CE1PHEA90 ATOM 865CE2PHEA90 ATOM 866CZPHEA90 ATOM 867NLYSA91 ATOM 868CALYSA91 ATOM 869CLYSA91 ATOM 870 O LYS A 91 -0.473 -23.263 6.846 1.00 0.66
ATOM 880CBLEUA92 ATOM 881CGLEUA92 ATOM 882CD1LEUA92 ATOM 883CD2LEUA92 ATOM 884 N ARG A 93 -1.142 -19.436 6.853 1.00 0.71 ATOM 885 CA ARG A 93 -2.377 -18.662 6.597 1.00 0.71 ATOM 886 C ARG A 93 -2.560 -17.563 7.659 1.00 0.71 ATOM 887 O ARG A 93 -3.661 -17.312 8.161 1.00 0.71 ATOM 888 CB ARG A 93 -3.592 -19.607 6.622 1.00 0.71 ATOM 889 CG ARG A 93 -3.550 -20.725 5.587 1.00 0.71 ATOM 890 CD ARG A 93 -3.733 -20.089 4.220 1.00 0.71 ATOM 891 NE ARG A 93 -3.737 -21.126 3.183 1.00 0.71 ATOM 892 CZ ARG A 93 -3.664 -20.878 1.878 1.00 0.71 ATOM 893 NH1 ARG A 93 -3.554 -19.633 1.430 1.00 0.71 ATOM 894 NH2 ARG A 93 -3.724 -21.874 1.007 1.00 0.71 ATOM 895 N VAL A 94 -1.452 -16.949 8.061 1.00 0.82 ATOM 896 CA VAL A 94 -1.490 -15.947 9.143 1.00 0.82 ATOM 897 C VAL A 94 -2.050 -14.643 8.571 1.00 0.82 ATOM 898 O VAL A 94 -1.503 -14.094 7.630 1.00 0.82 ATOM 899 CB VAL A 94 -0.107 -15.732 9.782 1.00 0.82 ATOM 900CG1VALA94 -0.159 -14.714 10.929 1.00 0.82 ATOM 901CG2VALA94 0.439 -17.046 10.345 1.00 0.82 ATOM 902 N ASP A 95 -3.016 -14.084 9.293 1.00 0.87 ATOM

903 CA ASP A 95 -3.597 -12.789 8.930 1.00 0.87 ATOM 904 C ASP A 95 -2.537 -
11.698 9.167 1.00 0.87
2.028 -17.417 3.916 1.00 0.79
0.909 -17.320 2.964 1.00 0.79
0.193 -18.664 2.780 1.00 0.79
1.405 -16.791 1.614 1.00 0.79
0.952 -19.662 2.324 1.00 0.64
0.397 -20.968 1.935 1.00 0.64
0.232 -22.019 3.027 1.00 0.64
1.187 -21.554 0.759 1.00 0.64
0.981 -20.750 -0.528 1.00 0.64
-0.205 -20.049 -0.751 1.00 0.64
1.980 -20.738 -1.495 1.00 0.64
-0.385 -19.335 -1.920 1.00 0.64
1.795 -20.026 -2.672 1.00 0.64
0.615 -19.320 -2.884 1.00 0.64
1.123 -21.993 4.013 1.00 0.66
1.105 -23.043 5.042 1.00 0.66
0.387 -22.572 6.310 1.00 0.66
ATOM 871CBLYSA91 ATOM 872CGLYSA91 ATOM 873CDLYSA91 ATOM
874CELYSA91 ATOM 875NZLYSA91 ATOM 876NLEUA92 ATOM
877CALEUA92 ATOM 878 C LEU A 92 -0.938 -20.119 7.977 1.00 0.70 ATOM 879 O
LEU A 92 -1.717 -20.132 8.931 1.00 0.70
2.531 -23.497 5.370 1.00 0.66
2.510 -24.636 6.391 1.00 0.66
3.911 -24.923 6.912 1.00 0.66
3.870 -26.070 7.919 1.00 0.66
5.229 -26.347 8.391 1.00 0.66
0.837 -21.421 6.793 1.00 0.70
0.369 -20.892 8.077 1.00 0.70
1.471 -20.031 8.678 1.00 0.70
2.728 -20.842 8.983 1.00 0.70
3.776 -19.848 9.454 1.00 0.70
2.504 -21.876 10.091 1.00 0.70
ATOM 905 O ASP A 95 -2.145 -11.485 10.323 1.00 0.87 ATOM 906 CB ASP A 95 -
4.862 -12.549 9.754 1.00 0.87 ATOM 907 CG ASP A 95 -5.662 -11.334 9.285 1.00 0.87
ATOM 908 OD1 ASP A 95 -5.042 -10.357 8.805 1.00 0.87 ATOM 909 OD2 ASP A 95 -
6.898 -11.422 9.414 1.00 0.87 ATOM 910 N PRO A 96 -2.159 -10.973 8.111 1.00 0.89
ATOM 911 CA PRO A 96 -1.135 -9.905 8.148 1.00 0.89 ATOM 912 C PRO A 96 -
1.351 -8.876 9.264 1.00 0.89 ATOM 913 O PRO A 96 -0.384 -8.288 9.759 1.00 0.89

ATOM 914 CB PRO A 96 -1.245 -9.217 6.790 1.00 0.89 ATOM 915 CG PRO A 96 -1.686 -10.349 5.867 1.00 0.89 ATOM 916 CD PRO A 96 -2.669 -11.134 6.731 1.00 0.89 ATOM 917 N THR A 97 -2.591 -8.719 9.713 1.00 0.87 ATOM 918 CA THR A 97 -2.939 -7.827 10.844 1.00 0.87 ATOM 919 C THR A 97 -2.125 -8.164 12.108 1.00 0.87 ATOM 920 O THR A 97 -1.624 -7.292 12.813 1.00 0.87 ATOM 921 CB THR A 97 -4.432 -7.917 11.184 1.00 0.87 ATOM 922OG1THRA97 -4.766 -9.256 11.568 1.00 0.87 ATOM 923CG2THRA97 -5.298 -7.435 10.014 1.00 0.87 ATOM 924 N ASN A 98 -1.844 -9.449 12.288 1.00 0.87 ATOM 925 CA ASN A 98 -1.064 -9.951 13.435 1.00 0.87 ATOM 926CASNA98 ATOM 927OASNA98 ATOM 928CBASNA98 ATOM 929CGASNA98 ATOM 930OD1ASNA98 ATOM 931ND2ASNA98 ATOM 932NPHEA99 ATOM 933CAPHEA99 ATOM 934CPHEA99 ATOM 935OPHEA99 ATOM 936CBPHEA99 ATOM 937CGPHEA99 ATOM 938CD1PHEA99 ATOM 939CD2PHEA99 ATOM 940CE1PHEA99 ATOM 941CE2PHEA99 ATOM 942CZPHEA99 ATOM 943NLYSA100 ATOM 944CALYSA100 ATOM 945CLYSA100 ATOM 946OLYSA100 ATOM 947CBLYSA100 ATOM 948CGLYSA100 ATOM 949CDLYSA100 ATOM 950CELYSA100 ATOM 951NZLYSA100 ATOM 952NLEUA101 ATOM 953CALEUA101 ATOM 954CLEUA101 ATOM 955OLEUA101 ATOM 956CBLEUA101 ATOM 957CGLEUA101 ATOM 958 CD1 LEU A 101

0.401 -9.500 13.449 1.00 0.87
0.959 -9.302 14.534 1.00 0.87
-1.147 -11.475 13.507 1.00 0.87
-2.596 -11.923 13.705 1.00 0.87
-3.284 -12.373 12.800 1.00 0.87
-3.079 -11.770 14.918 1.00 0.87
0.974 -9.239 12.277 1.00 0.86
2.362 -8.732 12.177 1.00 0.86
2.546 -7.416 12.923 1.00 0.86
3.498 -7.247 13.689 1.00 0.86
2.776 -8.502 10.721 1.00 0.86
2.750 -9.773 9.875 1.00 0.86
2.957 -11.024 10.456 1.00 0.86
2.499 -9.669 8.514 1.00 0.86
2.893 -12.167 9.679 1.00 0.86
2.440 -10.818 7.737 1.00 0.86
2.632 -12.065 8.319 1.00 0.86
1.521 -6.592 12.839 1.00 0.81
1.506 -5.268 13.483 1.00 0.81
1.421 -5.417 15.006 1.00 0.81
2.152 -4.753 15.740 1.00 0.81

3825	0.306	-4.451	13.025	1.00	0.81
3826	-0.174	-4.748	11.596	1.00	0.81
3827	-1.353	-3.869	11.161	1.00	0.81
3828	-2.414	-3.609	12.245	1.00	0.81
3829	-2.883	-4.845	12.885	1.00	0.81
3830	0.611	-6.390	15.439	1.00	0.86
3831	0.484	-6.749	16.858	1.00	0.86
3832	1.853	-7.114	17.442	1.00	0.86
3833	2.393	-6.368	18.206	1.00	0.86
3834	-0.500	-7.902	17.070	1.00	0.86
3835	-1.931	-7.520	16.686	1.00	0.86
3836	-2.835	-8.744	16.815	1.00	0.86
3837	-2.462	-6.388	17.571	1.00	0.86
3838	2.506	-8.086	16.755	1.00	0.89
3839	3.829	-8.552	17.185	1.00	0.89
3840	4.911	-7.468	17.157	1.00	0.89
3841	5.631	-7.307	18.148	1.00	0.89
3842	4.262	-9.750	16.326	1.00	0.89
3843	5.591	-10.346	16.811	1.00	0.89
3844	5.505	-10.839	18.261	1.00	0.89
3845	6.022	-11.478	15.880	1.00	0.89
3846	4.916	-6.652	16.111	1.00	0.91
3847	5.883	-5.541	15.967	1.00	0.91
3848	5.816	-4.560	17.140	1.00	0.91
3849	6.847	-4.195	17.710	1.00	0.91
3850	5.638	-4.746	14.686	1.00	0.91
3851	5.883	-5.576	13.552	1.00	0.91
3852	4.592	-4.282	17.585	1.00	0.90
3853	4.355	-3.400	18.743	1.00	0.90
3854	4.909	-4.024	20.034	1.00	0.90
3855	5.498	-3.322	20.860	1.00	0.90
3856	2.856	-3.085	18.844	1.00	0.90
3857	2.519	-2.056	19.933	1.00	0.90
3858	1.194	-1.380	19.596	1.00	0.90
3859	2.360	-2.707	21.312	1.00	0.90
3860	4.769	-5.338	20.160	1.00	0.93
3861	5.308	-6.104	21.304	1.00	0.93
3862	6.843	-6.123	21.306	1.00	0.93
3863	7.467	-5.990	22.359	1.00	0.93
3864	4.784	-7.545	21.319	1.00	0.93

3.285 -7.601 21.563 1.00 0.93
2.785 -7.536 22.695 1.00 0.93
2.521 -7.699 20.520 1.00 0.93
7.431 -6.168 20.113 1.00 0.91
8.899 -6.137 19.934 1.00 0.91
9.469 -4.790 20.408 1.00 0.91

ATOM 959 CD2 LEU A 101 ATOM 960NLEUA102 ATOM 961CALEUA102 ATOM 962CLEUA102 ATOM 963OLEUA102 ATOM 964CBLEUA102 ATOM 965CGLEUA102 ATOM 966 CD1 LEU A 102 ATOM 967 CD2 LEU A 102 ATOM 968NSERA103 ATOM 969CASERA103 ATOM 970CSERA103 ATOM 971OSERA103 ATOM 972CBSERA103 ATOM 973OGSERA103 ATOM 974NLEUA104 ATOM 975CALEUA104 ATOM 976CLEUA104 ATOM 977OLEUA104 ATOM 978CBLEUA104 ATOM 979CGLEUA104 ATOM 980 CD1 LEU A 104 ATOM 981 CD2 LEU A 104 ATOM 982NASNA105 ATOM 983CAASNA105 ATOM 984CASNA105 ATOM 985OASNA105 ATOM 986CBASNA105 ATOM 987CGASNA105 ATOM 988 OD1 ASN A 105 ATOM 989 ND2 ASN A 105 ATOM 990NILEA106 ATOM 991CAILEA106 ATOM 992CILEA106 ATOM 993 O ILE A 106 10.489 -4.762 21.104 1.00 0.91 ATOM 994CBILEA106 ATOM 995 CG1 ILE A 106 ATOM 996 CG2 ILE A 106 ATOM 997 CD1 ILE A 106 ATOM 998NLEUA107 ATOM 999CALEUA107 ATOM 1000CLEUA107 ATOM 1001OLEUA107 ATOM 1002 CB LEU A 107 ATOM 1003 CG LEU A 107 ATOM 1004 CD1 LEU A 107 ATOM 1005 CD2 LEU A 107 ATOM 1006NVALA108 ATOM 1007 CA VAL A 108 ATOM 1008CVALA108 ATOM 1009OVALA108 ATOM 1010 CB VAL A 108 ATOM 1011 CG1 VAL A 108 ATOM 1012 CG2 VAL A 108

9.283 -6.453 18.473 1.00 0.91
8.899 -7.899 18.127 1.00 0.91
10.783 -6.237 18.208 1.00 0.91
9.120 -8.267 16.653 1.00 0.91
8.782 -3.710 20.051 1.00 0.92
9.177 -2.346 20.454 1.00 0.92
9.118 -2.151 21.969 1.00 0.92
10.048 -1.606 22.558 1.00 0.92
8.296 -1.298 19.769 1.00 0.92
8.458 -1.283 18.246 1.00 0.92
7.465 -0.300 17.634 1.00 0.92
9.863 -0.853 17.839 1.00 0.92
8.092 -2.747 22.573 1.00 0.92
7.880 -2.712 24.036 1.00 0.92
9.018 -3.441 24.772 1.00 0.92

3905 9.506 -2.966 25.798 1.00 0.92
3906 6.502 -3.309 24.387 1.00 0.92
3907 6.292 -3.493 25.896 1.00 0.92
3908 5.380 -2.397 23.885 1.00 0.92
3909 ATOM 1013 N VAL A 109
3910 ATOM 1014 CA VAL A 109
3911 ATOM 1015 C VAL A 109
3912 ATOM 1016 O VAL A 109
3913 ATOM 1017 CB VAL A 109
3914 ATOM 1018 CG1 VAL A 109
3915 ATOM 1019 CG2 VAL A 109
3916 ATOM 1020 N MET A 110
3917 ATOM 1021 CA MET A 110
3918 ATOM 1022 C MET A 110
3919 ATOM 1023 O MET A 110
3920 ATOM 1024 CB MET A 110
3921 ATOM 1025 CG MET A 110
3922 ATOM 1026 SD MET A 110
3923 ATOM 1027 CE MET A 110
3924 ATOM 1028 N ALA A 111
3925 ATOM 1029 CA ALA A 111
3926 ATOM 1030 C ALA A 111
3927 ATOM 1031 O ALA A 111
3928 ATOM 1032 CB ALA A 111
3929 ATOM 1033 N ILE A 112
3930 ATOM 1034 CA ILE A 112
3931 ATOM 1035 C ILE A 112
3932 ATOM 1036 O ILE A 112
3933 ATOM 1037 CB ILE A 112
3934 ATOM 1038 CG1 ILE A 112
3935 ATOM 1039 CG2 ILE A 112
3936 ATOM 1040 CD1 ILE A 112
3937 ATOM 1041 N MET A 113
3938 ATOM 1042 CA MET A 113
3939 ATOM 1043 C MET A 113
3940 ATOM 1044 O MET A 113
3941 ATOM 1045 CB MET A 113
3942 ATOM 1046 CG MET A 113
3943 ATOM 1047 SD MET A 113
3944 ATOM 1048 CE MET A 113

ATOM 1049 N PHE A 114
ATOM 1050 CA PHE A 114
ATOM 1051 C PHE A 114
ATOM 1052 O PHE A 114
ATOM 1053 CB PHE A 114
ATOM 1054 CG PHE A 114
ATOM 1055 CD1 PHE A 114
ATOM 1056 CD2 PHE A 114
ATOM 1057 CE1 PHE A 114
ATOM 1058 CE2 PHE A 114
ATOM 1059 CZ PHE A 114
ATOM 1060 N PRO A 115
ATOM 1061 CA PRO A 115
ATOM 1062 C PRO A 115
ATOM 1063 O PRO A 115
ATOM 1064 CB PRO A 115
ATOM 1065 CG PRO A 115
ATOM 1066 CD PRO A 115
9.442 -4.568 24.213 1.00 0.92
10.511 -5.398 24.801 1.00 0.92
11.860 -4.672 24.707 1.00 0.92
12.577 -4.498 25.681 1.00 0.92
10.569 -6.776 24.123 1.00 0.92
11.737 -7.622 24.640 1.00 0.92
9.277 -7.548 24.382 1.00 0.92
12.209 -4.259 23.492 1.00 0.88
13.454 -3.495 23.275 1.00 0.88
13.502 -2.240 24.157 1.00 0.88
14.536 -1.945 24.747 1.00 0.88
13.567 -3.065 21.817 1.00 0.88
13.668 -4.277 20.893 1.00 0.88
13.802 -3.820 19.129 1.00 0.88
12.173 -3.181 18.848 1.00 0.88
12.329 -1.644 24.372 1.00 0.89
12.165 -0.460 25.234 1.00 0.89
12.504 -0.762 26.700 1.00 0.89
13.278 -0.023 27.312 1.00 0.89
10.731 0.066 25.140 1.00 0.89
12.028 -1.901 27.192 1.00 0.82
12.295 -2.342 28.578 1.00 0.82

3985	13.754	-2.792	28.791	1.00	0.82
3986	14.283	-2.678	29.892	1.00	0.82
3987	11.314	-3.431	29.057	1.00	0.82
3988	11.504	-4.756	28.308	1.00	0.82
3989	9.868	-2.928	28.962	1.00	0.82
3990	10.710	-5.950	28.825	1.00	0.82
3991	14.367	-3.326	27.733	1.00	0.83
3992	15.718	-3.915	27.821	1.00	0.83
3993	16.848	-2.933	27.495	1.00	0.83
3994	17.905	-2.969	28.126	1.00	0.83
3995	15.854	-5.124	26.897	1.00	0.83
3996	15.045	-6.328	27.362	1.00	0.83
3997	15.384	-7.805	26.342	1.00	0.83
3998	14.325	-8.936	27.212	1.00	0.83
3999	16.604	-2.068	26.521	1.00	0.88
4000	17.612	-1.107	26.023	1.00	0.88
4001	17.035	0.314	26.076	1.00	0.88
4002	17.049	1.029	25.044	1.00	0.88
4003	18.051	-1.504	24.609	1.00	0.88
4004	18.487	-2.963	24.549	1.00	0.88
4005	19.793	-3.317	24.857	1.00	0.88
4006	17.544	-3.935	24.246	1.00	0.88
4007	20.164	-4.654	24.846	1.00	0.88
4008	17.911	-5.270	24.256	1.00	0.88
4009	19.225	-5.631	24.539	1.00	0.88
4010	16.702	0.799	27.264	1.00	0.87
4011	16.097	2.130	27.466	1.00	0.87
4012	16.984	3.284	26.968	1.00	0.87
4013	16.487	4.230	26.377	1.00	0.87
4014	15.809	2.221	28.963	1.00	0.87
4015	16.844	1.295	29.603	1.00	0.87
4016	16.969	0.169	28.587	1.00	0.87
4017	ATOM	1067	N ASP A 116		
4018	ATOM	1068	CA ASP A 116		
4019	ATOM	1069	C ASP A 116		
4020	ATOM	1070	O ASP A 116		
4021	ATOM	1071	CB ASP A 116		
4022	ATOM	1072	CG ASP A 116		
4023	ATOM	1073	OD1 ASP A 116		
4024	ATOM	1074	OD2 ASP A 116		

4025	ATOM	1075	N	ASP	A	117
4026	ATOM	1076	CA	ASP	A	117
4027	ATOM	1077	C	ASP	A	117
4028	ATOM	1078	O	ASP	A	117
4029	ATOM	1079	CB	ASP	A	117
4030	ATOM	1080	CG	ASP	A	117
4031	ATOM	1081	OD1	ASP	A	117
4032	ATOM	1082	OD2	ASP	A	117
4033	ATOM	1083	N	PHE	A	118
4034	ATOM	1084	CA	PHE	A	118
4035	ATOM	1085	C	PHE	A	118
4036	ATOM	1086	O	PHE	A	118
4037	ATOM	1087	CB	PHE	A	118
4038	ATOM	1088	CG	PHE	A	118
4039	ATOM	1089	CD1	PHE	A	118
4040	ATOM	1090	CD2	PHE	A	118
4041	ATOM	1091	CE1	PHE	A	118
4042	ATOM	1092	CE2	PHE	A	118
4043	ATOM	1093	CZ	PHE	A	118
4044	ATOM	1094	N	THR	A	119
4045	ATOM	1095	CA	THR	A	119
4046	ATOM	1096	C	THR	A	119
4047	ATOM	1097	O	THR	A	119
4048	ATOM	1098	CB	THR	A	119
4049	ATOM	1099	OG1	THR	A	119
4050	ATOM	1100	CG2	THR	A	119
4051	ATOM	1101	N	PRO	A	120
4052	ATOM	1102	CA	PRO	A	120
4053	ATOM	1103	C	PRO	A	120
4054	ATOM	1104	O	PRO	A	120
4055	ATOM	1105	CB	PRO	A	120
4056	ATOM	1106	CG	PRO	A	120
4057	ATOM	1107	CD	PRO	A	120
4058	ATOM	1108	N	MET	A	121
4059	ATOM	1109	CA	MET	A	121
4060	ATOM	1110	C	MET	A	121
4061	ATOM	1111	O	MET	A	121
4062	ATOM	1112	CB	MET	A	121
4063	ATOM	1113	CG	MET	A	121
4064	ATOM	1114	SD	MET	A	121

ATOM	1115	CE	MET	A	121
ATOM	1116	N	ALA	A	122
ATOM	1117	CA	ALA	A	122
ATOM	1118	C	ALA	A	122
ATOM	1119	O	ALA	A	122
ATOM	1120	CB	ALA	A	122

18.308	3.114	27.079	1.00	0.83
19.257	4.151	26.632	1.00	0.83
19.487	4.188	25.115	1.00	0.83
19.702	5.247	24.542	1.00	0.83
20.590	4.036	27.378	1.00	0.83
20.448	4.340	28.875	1.00	0.83
19.464	5.014	29.253	1.00	0.83
21.339	3.878	29.616	1.00	0.83
19.464	3.003	24.501	1.00	0.87
19.659	2.873	23.045	1.00	0.87
18.409	3.219	22.231	1.00	0.87
18.526	3.753	21.126	1.00	0.87
20.129	1.463	22.683	1.00	0.87
21.513	1.160	23.257	1.00	0.87
22.454	1.909	22.920	1.00	0.87
21.571	0.231	24.091	1.00	0.87
17.242	2.913	22.792	1.00	0.90
15.954	3.086	22.101	1.00	0.90
15.495	4.547	22.118	1.00	0.90
14.451	4.909	22.669	1.00	0.90
14.912	2.147	22.718	1.00	0.90
13.712	1.972	21.783	1.00	0.90
13.927	1.662	20.445	1.00	0.90
12.414	2.082	22.269	1.00	0.90
12.851	1.454	19.602	1.00	0.90
11.338	1.865	21.417	1.00	0.90
11.553	1.553	20.082	1.00	0.90
16.316	5.397	21.532	1.00	0.88
16.019	6.830	21.400	1.00	0.88
14.831	7.030	20.440	1.00	0.88
14.590	6.164	19.576	1.00	0.88
17.251	7.617	20.924	1.00	0.88
17.569	7.265	19.574	1.00	0.88
18.436	7.455	21.885	1.00	0.88

4105	14.167	8.180	20.495	1.00	0.91
4106	13.058	8.542	19.581	1.00	0.91
4107	13.467	8.408	18.102	1.00	0.91
4108	12.687	7.964	17.263	1.00	0.91
4109	12.762	10.005	19.894	1.00	0.91
4110	13.159	10.151	21.361	1.00	0.91
4111	14.393	9.266	21.479	1.00	0.91
4112	14.750	8.671	17.836	1.00	0.81
4113	15.341	8.570	16.484	1.00	0.81
4114	15.487	7.116	16.016	1.00	0.81
4115	15.282	6.810	14.843	1.00	0.81
4116	16.721	9.228	16.440	1.00	0.81
4117	16.638	10.734	16.667	1.00	0.81
4118	18.242	11.597	16.535	1.00	0.81
4119	17.765	13.286	16.822	1.00	0.81
4120	15.810	6.237	16.965	1.00	0.87
4121	15.912	4.789	16.706	1.00	0.87
4122	14.514	4.183	16.525	1.00	0.87
4123	14.289	3.367	15.635	1.00	0.87
4124	16.622	4.101	17.875	1.00	0.87

4125	ATOM	1121	N	HIS A 123
4126	ATOM	1122	CA	HIS A 123
4127	ATOM	1123	C	HIS A 123
4128	ATOM	1124	O	HIS A 123
4129	ATOM	1125	CB	HIS A 123
4130	ATOM	1126	CG	HIS A 123
4131	ATOM	1127	ND1	HIS A 123
4132	ATOM	1128	CD2	HIS A 123
4133	ATOM	1129	CE1	HIS A 123
4134	ATOM	1130	NE2	HIS A 123
4135	ATOM	1131	N	LEU A 124
4136	ATOM	1132	CA	LEU A 124
4137	ATOM	1133	C	LEU A 124
4138	ATOM	1134	O	LEU A 124
4139	ATOM	1135	CB	LEU A 124
4140	ATOM	1136	CG	LEU A 124
4141	ATOM	1137	CD1	LEU A 124
4142	ATOM	1138	CD2	LEU A 124
4143	ATOM	1139	N	ALA A 125
4144	ATOM	1140	CA	ALA A 125

ATOM 1141 C ALA A 125
ATOM 1142 O ALA A 125
ATOM 1143 CB ALA A 125
ATOM 1144 N VAL A 126
ATOM 1145 CA VAL A 126
ATOM 1146 C VAL A 126
ATOM 1147 O VAL A 126
ATOM 1148 CB VAL A 126
ATOM 1149 CG1 VAL A 126
ATOM 1150 CG2 VAL A 126
ATOM 1151 N ASP A 127
ATOM 1152 CA ASP A 127
ATOM 1153 C ASP A 127
ATOM 1154 O ASP A 127
ATOM 1155 CB ASP A 127
ATOM 1156 CG ASP A 127
ATOM 1157 OD1 ASP A 127
ATOM 1158 OD2 ASP A 127
ATOM 1159 N LYS A 128
ATOM 1160 CA LYS A 128
ATOM 1161 C LYS A 128
ATOM 1162 O LYS A 128
ATOM 1163 CB LYS A 128
ATOM 1164 CG LYS A 128
ATOM 1165 CD LYS A 128
ATOM 1166 CE LYS A 128
ATOM 1167 NZ LYS A 128
ATOM 1168 N PHE A 129
ATOM 1169 CA PHE A 129
ATOM 1170 C PHE A 129
ATOM 1171 O PHE A 129
ATOM 1172 CB PHE A 129
ATOM 1173 CG PHE A 129
ATOM 1174 CD1 PHE A 129
13.562 4.769 17.253 1.00 0.88
12.155 4.357 17.272 1.00 0.88
11.519 4.544 15.887 1.00 0.88
11.044 3.581 15.284 1.00 0.88
11.414 5.191 18.318 1.00 0.88
10.020 4.654 18.656 1.00 0.88

4185	9.398	4.840	19.811	1.00	0.88
4186	9.198	3.936	17.893	1.00	0.88
4187	8.213	4.249	19.775	1.00	0.88
4188	8.092	3.690	18.579	1.00	0.88
4189	11.615	5.757	15.354	1.00	0.87
4190	11.047	6.058	14.027	1.00	0.87
4191	11.613	5.117	12.951	1.00	0.87
4192	10.868	4.594	12.118	1.00	0.87
4193	11.296	7.533	13.682	1.00	0.87
4194	10.721	7.948	12.318	1.00	0.87
4195	10.535	9.458	12.282	1.00	0.87
4196	11.679	7.627	11.168	1.00	0.87
4197	12.907	4.851	13.025	1.00	0.89
4198	13.582	4.033	12.006	1.00	0.89
4199	13.264	2.542	12.124	1.00	0.89
4200	12.994	1.886	11.109	1.00	0.89
4201	15.065	4.264	12.166	1.00	0.89
4202	13.190	2.049	13.351	1.00	0.91
4203	12.826	0.645	13.623	1.00	0.91
4204	11.355	0.399	13.253	1.00	0.91
4205	11.023	-0.634	12.684	1.00	0.91
4206	13.131	0.244	15.078	1.00	0.91
4207	12.299	1.008	16.104	1.00	0.91
4208	12.939	-1.258	15.297	1.00	0.91
4209	10.511	1.384	13.556	1.00	0.90
4210	9.080	1.311	13.239	1.00	0.90
4211	8.876	1.209	11.725	1.00	0.90
4212	8.235	0.270	11.257	1.00	0.90
4213	8.340	2.534	13.783	1.00	0.90
4214	6.828	2.284	13.767	1.00	0.90
4215	6.422	1.150	14.094	1.00	0.90
4216	6.101	3.216	13.386	1.00	0.90
4217	9.587	2.051	10.986	1.00	0.86
4218	9.562	1.997	9.517	1.00	0.86
4219	10.101	0.659	8.988	1.00	0.86
4220	9.502	0.042	8.106	1.00	0.86
4221	10.447	3.114	9.001	1.00	0.86
4222	9.866	4.520	9.170	1.00	0.86
4223	10.751	5.546	8.451	1.00	0.86
4224	10.613	5.535	6.923	1.00	0.86

10.869 4.204 6.353 1.00 0.86
11.161 0.177 9.633 1.00 0.93
11.791 -1.115 9.308 1.00 0.93
10.851 -2.303 9.553 1.00 0.93
10.652 -3.132 8.665 1.00 0.93
13.087 -1.264 10.111 1.00 0.93
13.685 -2.667 9.994 1.00 0.93
14.239 -3.097 8.796 1.00 0.93
ATOM 1175 CD2 PHE A 129
ATOM 1176 CE1 PHE A 129
ATOM 1177 CE2 PHE A 129
ATOM 1178 CZ PHE A 129
ATOM 1179 N LEU A 130
ATOM 1180 CA LEU A 130
ATOM 1181 C LEU A 130
ATOM 1182 O LEU A 130
ATOM 1183 CB LEU A 130
ATOM 1184 CG LEU A 130
ATOM 1185 CD1 LEU A 130
ATOM 1186 CD2 LEU A 130
ATOM 1187 N CYS A 131
ATOM 1188 CA CYS A 131
ATOM 1189 C CYS A 131
ATOM 1190 O CYS A 131
ATOM 1191 CB CYS A 131
ATOM 1192 SG CYS A 131
ATOM 1193 N ALA A 132
ATOM 1194 CA ALA A 132
ATOM 1195 C ALA A 132
ATOM 1196 O ALA A 132
ATOM 1197 CB ALA A 132
ATOM 1198 N LEU A 133
ATOM 1199 CA LEU A 133
ATOM 1200 C LEU A 133
ATOM 1201 O LEU A 133
ATOM 1202 CB LEU A 133
ATOM 1203 CG LEU A 133
ATOM 1204 CD1 LEU A 133
ATOM 1205 CD2 LEU A 133
ATOM 1206 N ALA A 134

ATOM 1207 CA ALA A 134
ATOM 1208 C ALA A 134
ATOM 1209 O ALA A 134
ATOM 1210 CB ALA A 134
ATOM 1211 N LEU A 135
ATOM 1212 CA LEU A 135
ATOM 1213 C LEU A 135
ATOM 1214 O LEU A 135
ATOM 1215 CB LEU A 135
ATOM 1216 CG LEU A 135
ATOM 1217 CD1 LEU A 135
ATOM 1218 CD2 LEU A 135
ATOM 1219 N ALA A 136
ATOM 1220 CA ALA A 136
ATOM 1221 C ALA A 136
ATOM 1222 O ALA A 136
ATOM 1223 CB ALA A 136
ATOM 1224 N LEU A 137
ATOM 1225 CA LEU A 137
ATOM 1226 C LEU A 137
ATOM 1227 O LEU A 137
ATOM 1228 CB LEU A 137
13.661 -3.510 11.097 1.00 0.93
14.776 -4.372 8.704 1.00 0.93
14.214 -4.782 11.006 1.00 0.93
14.771 -5.211 9.809 1.00 0.93
10.213 -2.323 10.713 1.00 0.90
9.283 -3.404 11.090 1.00 0.90
8.026 -3.425 10.223 1.00 0.90
7.568 -4.497 9.818 1.00 0.90
8.904 -3.318 12.569 1.00 0.90
10.059 -3.734 13.488 1.00 0.90
9.654 -3.494 14.942 1.00 0.90
10.434 -5.207 13.291 1.00 0.90
7.584 -2.236 9.827 1.00 0.89
6.462 -2.088 8.878 1.00 0.89
6.856 -2.627 7.495 1.00 0.89
6.088 -3.336 6.847 1.00 0.89
6.057 -0.621 8.748 1.00 0.89
5.419 0.106 10.301 1.00 0.89

4305	8.125	-2.411	7.154	1.00	0.90
4306	8.731	-2.899	5.903	1.00	0.90
4307	8.865	-4.428	5.889	1.00	0.90
4308	8.635	-5.067	4.861	1.00	0.90
4309	10.099	-2.243	5.721	1.00	0.90
4310	9.116	-5.002	7.062	1.00	0.89
4311	9.218	-6.460	7.219	1.00	0.89
4312	7.826	-7.095	7.097	1.00	0.89
4313	7.652	-8.065	6.364	1.00	0.89
4314	9.887	-6.767	8.559	1.00	0.89
4315	10.173	-8.260	8.721	1.00	0.89
4316	11.333	-8.429	9.684	1.00	0.89
4317	8.990	-8.982	9.353	1.00	0.89
4318	6.843	-6.457	7.731	1.00	0.89
4319	5.430	-6.868	7.656	1.00	0.89
4320	4.906	-6.846	6.212	1.00	0.89
4321	4.265	-7.790	5.767	1.00	0.89
4322	4.582	-5.933	8.518	1.00	0.89
4323	5.325	-5.826	5.457	1.00	0.84
4324	4.922	-5.678	4.050	1.00	0.84
4325	5.577	-6.746	3.162	1.00	0.84
4326	4.931	-7.331	2.296	1.00	0.84
4327	5.286	-4.279	3.544	1.00	0.84
4328	4.619	-3.985	2.196	1.00	0.84
4329	3.100	-3.843	2.343	1.00	0.84
4330	5.234	-2.736	1.572	1.00	0.84
4331	6.846	-7.026	3.461	1.00	0.86
4332	7.629	-8.064	2.766	1.00	0.86
4333	7.078	-9.469	3.040	1.00	0.86
4334	6.886	-10.255	2.108	1.00	0.86
4335	9.088	-7.985	3.207	1.00	0.86
4336	6.666	-9.697	4.284	1.00	0.82
4337	6.046	-10.965	4.702	1.00	0.82
4338	4.709	-11.242	4.014	1.00	0.82
4339	4.372	-12.384	3.747	1.00	0.82
4340	5.811	-11.006	6.211	1.00	0.82
4341	ATOM	1229	CG	LEU A 137	
4342	ATOM	1230	CD1	LEU A 137	
4343	ATOM	1231	CD2	LEU A 137	
4344	ATOM	1232	N	SER A 138	

4345	ATOM	1233	CA	SER	A	138
4346	ATOM	1234	C	SER	A	138
4347	ATOM	1235	O	SER	A	138
4348	ATOM	1236	CB	SER	A	138
4349	ATOM	1237	OG	SER	A	138
4350	ATOM	1238	N	GLU	A	139
4351	ATOM	1239	CA	GLU	A	139
4352	ATOM	1240	C	GLU	A	139
4353	ATOM	1241	O	GLU	A	139
4354	ATOM	1242	CB	GLU	A	139
4355	ATOM	1243	CG	GLU	A	139
4356	ATOM	1244	CD	GLU	A	139
4357	ATOM	1245	OE1	GLU	A	139
4358	ATOM	1246	OE2	GLU	A	139
4359	ATOM	1247	N	LYS	A	140
4360	ATOM	1248	CA	LYS	A	140
4361	ATOM	1249	C	LYS	A	140
4362	ATOM	1250	O	LYS	A	140
4363	ATOM	1251	CB	LYS	A	140
4364	ATOM	1252	CG	LYS	A	140
4365	ATOM	1253	CD	LYS	A	140
4366	ATOM	1254	CE	LYS	A	140
4367	ATOM	1255	NZ	LYS	A	140
4368	ATOM	1256	N	TYR	A	141
4369	ATOM	1257	CA	TYR	A	141
4370	ATOM	1258	C	TYR	A	141
4371	ATOM	1259	O	TYR	A	141
4372	ATOM	1260	CB	TYR	A	141
4373	ATOM	1261	CG	TYR	A	141
4374	ATOM	1262	CD1	TYR	A	141
4375	ATOM	1263	CD2	TYR	A	141
4376	ATOM	1264	CE1	TYR	A	141
4377	ATOM	1265	CE2	TYR	A	141
4378	ATOM	1266	CZ	TYR	A	141
4379	ATOM	1267	OH	TYR	A	141
4380	ATOM	1268	N	ARG	A	142
4381	ATOM	1269	CA	ARG	A	142
4382	ATOM	1270	C	ARG	A	142
4383	ATOM	1271	O	ARG	A	142
4384	ATOM	1272	CB	ARG	A	142

```
ATOM   1273  CG  ARG A 142
ATOM   1274  CD  ARG A 142
ATOM   1275  NE  ARG A 142
ATOM   1276  CZ  ARG A 142
ATOM   1277  NH1 ARG A 142
ATOM   1278  NH2 ARG A 142
ATOM   1279  N   GLU B 144
ATOM   1280  CA  GLU B 144
ATOM   1281  C   GLU B 144
ATOM   1282  O   GLU B 144
  7.080 -11.292   7.016  1.00  0.82
  6.695 -11.342   8.492  1.00  0.82
  7.700 -12.633   6.619  1.00  0.82
  4.010 -10.161   3.684  1.00  0.86
  2.680 -10.241   3.056  1.00  0.86
  2.726 -10.291   1.526  1.00  0.86
  1.699 -10.516   0.887  1.00  0.86
  1.830  -9.047   3.490  1.00  0.86
  1.668  -9.069   4.908  1.00  0.86
  3.935 -10.257   0.965  1.00  0.79
  4.139 -10.184  -0.490  1.00  0.79
  3.425 -11.290  -1.286  1.00  0.79
  2.771 -11.013  -2.284  1.00  0.79
  5.639 -10.233  -0.794  1.00  0.79
  5.909  -9.958  -2.276  1.00  0.79
  5.626  -8.513  -2.697  1.00  0.79
  5.019  -7.729  -1.928  1.00  0.79
  6.199  -8.173  -3.747  1.00  0.79
  3.565 -12.522  -0.805  1.00  0.77
  3.115 -13.710  -1.546  1.00  0.77
  1.701 -14.183  -1.195  1.00  0.77
  1.248 -15.189  -1.735  1.00  0.77
  4.115 -14.847  -1.310  1.00  0.77
  5.513 -14.537  -1.868  1.00  0.77
  5.559 -14.443  -3.397  1.00  0.77
  5.221 -15.785  -4.047  1.00  0.77
  5.330 -15.732  -5.507  1.00  0.77
  0.999 -13.431  -0.346  1.00  0.76
 -0.378 -13.786   0.049  1.00  0.76
 -1.367 -13.817  -1.123  1.00  0.76
```

4425 -2.020 -14.832 -1.355 1.00 0.76
4426 -0.890 -12.858 1.154 1.00 0.76
4427 -0.397 -13.304 2.530 1.00 0.76
4428 0.944 -13.242 2.877 1.00 0.76
4429 -1.321 -13.708 3.475 1.00 0.76
4430 1.352 -13.577 4.159 1.00 0.76
4431 -0.924 -14.031 4.759 1.00 0.76
4432 0.420 -13.969 5.108 1.00 0.76
4433 0.832 -14.321 6.351 1.00 0.76
4434 -1.435 -12.723 -1.872 1.00 0.76
4435 -2.434 -12.569 -2.955 1.00 0.76
4436 -1.932 -11.723 -4.138 1.00 0.76
4437 -0.905 -11.037 -3.934 1.00 0.76
4438 -3.704 -11.957 -2.356 1.00 0.76
4439 -3.416 -10.558 -1.813 1.00 0.76
4440 -4.620 -9.972 -1.105 1.00 0.76
4441 -4.283 -8.579 -0.784 1.00 0.76
4442 -5.169 -7.611 -0.578 1.00 0.76
4443 -6.469 -7.870 -0.618 1.00 0.76
4444 -4.756 -6.362 -0.411 1.00 0.76
4445 -14.831 -6.526 32.604 1.00 0.72
4446 -14.576 -5.092 32.810 1.00 0.72
4447 -13.363 -4.893 33.731 1.00 0.72
4448 -12.265 -4.950 33.282 1.00 0.72
4449 ATOM 1283 CB GLU B 144
4450 ATOM 1284 CG GLU B 144
4451 ATOM 1285 CD GLU B 144
4452 ATOM 1286 OE1 GLU B 144
4453 ATOM 1287 OE2 GLU B 144
4454 ATOM 1288 N TRP B 145
4455 ATOM 1289 CA TRP B 145
4456 ATOM 1290 C TRP B 145
4457 ATOM 1291 O TRP B 145
4458 ATOM 1292 CB TRP B 145
4459 ATOM 1293 CG TRP B 145
4460 ATOM 1294 CD1 TRP B 145
4461 ATOM 1295 CD2 TRP B 145
4462 ATOM 1296 NE1 TRP B 145
4463 ATOM 1297 CE2 TRP B 145
4464 ATOM 1298 CE3 TRP B 145

ATOM 1299 CZ2 TRP B 145
ATOM 1300 CZ3 TRP B 145
ATOM 1301 CH2 TRP B 145
ATOM 1302 N THR B 146
ATOM 1303 CA THR B 146
ATOM 1304 C THR B 146
ATOM 1305 O THR B 146
ATOM 1306 CB THR B 146
ATOM 1307 OG1 THR B 146
ATOM 1308 CG2 THR B 146
ATOM 1309 N ALA B 147
ATOM 1310 CA ALA B 147
ATOM 1311 C ALA B 147
ATOM 1312 O ALA B 147
ATOM 1313 CB ALA B 147
ATOM 1314 N ALA B 148
ATOM 1315 CA ALA B 148
ATOM 1316 C ALA B 148
ATOM 1317 O ALA B 148
ATOM 1318 CB ALA B 148
ATOM 1319 N GLU B 149
ATOM 1320 CA GLU B 149
ATOM 1321 C GLU B 149
ATOM 1322 O GLU B 149
ATOM 1323 CB GLU B 149
ATOM 1324 CG GLU B 149
ATOM 1325 CD GLU B 149
ATOM 1326 OE1 GLU B 149
ATOM 1327 OE2 GLU B 149
ATOM 1328 N ARG B 150
ATOM 1329 CA ARG B 150
ATOM 1330 C ARG B 150
ATOM 1331 O ARG B 150
ATOM 1332 CB ARG B 150
ATOM 1333 CG ARG B 150
ATOM 1334 CD ARG B 150
ATOM 1335 NE ARG B 150
ATOM 1336 CZ ARG B 150
-15.837 -4.382 33.302 1.00 0.72
-15.717 -2.870 33.075 1.00 0.72

4505	-15.520	-2.536	31.591	1.00	0.72
4506	-16.371	-2.980	30.787	1.00	0.72
4507	-14.538	-1.821	31.302	1.00	0.72
4508	-13.541	-4.772	35.052	1.00	0.89
4509	-12.351	-4.672	35.912	1.00	0.89
4510	-12.312	-5.792	36.932	1.00	0.89
4511	-13.281	-6.043	37.660	1.00	0.89
4512	-12.247	-3.327	36.637	1.00	0.89
4513	-12.165	-2.138	35.705	1.00	0.89
4514	-13.012	-1.139	35.679	1.00	0.89
4515	-11.139	-1.880	34.703	1.00	0.89
4516	-12.620	-0.250	34.731	1.00	0.89
4517	-11.576	-0.666	34.183	1.00	0.89
4518	-10.058	-2.522	34.330	1.00	0.89
4519	-10.730	-0.109	33.123	1.00	0.89
4520	-9.241	-1.982	33.274	1.00	0.89
4521	-9.644	-0.773	32.716	1.00	0.89
4522	-11.153	-6.406	36.985	1.00	0.91
4523	-10.820	-7.403	38.016	1.00	0.91
4524	-10.067	-6.638	39.111	1.00	0.91
4525	-9.414	-5.622	38.829	1.00	0.91
4526	-9.942	-8.524	37.435	1.00	0.91
4527	-8.653	-8.000	37.103	1.00	0.91
4528	-10.596	-9.204	36.223	1.00	0.91
4529	-10.089	-7.157	40.331	1.00	0.83
4530	-9.368	-6.525	41.457	1.00	0.83
4531	-7.868	-6.357	41.154	1.00	0.83
4532	-7.303	-5.295	41.363	1.00	0.83
4533	-9.537	-7.351	42.729	1.00	0.83
4534	-7.320	-7.357	40.448	1.00	0.86
4535	-5.912	-7.354	40.010	1.00	0.86
4536	-5.617	-6.179	39.069	1.00	0.86
4537	-4.753	-5.362	39.363	1.00	0.86
4538	-5.585	-8.675	39.309	1.00	0.86
4539	-6.523	-5.957	38.113	1.00	0.83
4540	-6.399	-4.838	37.160	1.00	0.83
4541	-6.513	-3.476	37.853	1.00	0.83
4542	-5.676	-2.610	37.620	1.00	0.83
4543	-7.463	-4.930	36.072	1.00	0.83
4544	-7.265	-6.172	35.203	1.00	0.83

4545	-8.407	-6.376	34.205	1.00	0.83
4546	-9.529	-5.886	34.472	1.00	0.83
4547	-8.145	-7.101	33.226	1.00	0.83
4548	-7.406	-3.393	38.841	1.00	0.80
4549	-7.565	-2.180	39.667	1.00	0.80
4550	-6.313	-1.896	40.508	1.00	0.80
4551	-5.891	-0.757	40.685	1.00	0.80
4552	-8.740	-2.300	40.638	1.00	0.80
4553	-10.087	-2.427	39.934	1.00	0.80
4554	-11.186	-2.396	40.993	1.00	0.80
4555	-12.496	-2.569	40.347	1.00	0.80
4556	-13.366	-1.598	40.063	1.00	0.80
4557	ATOM	1337	NH1 ARG B 150		
4558	ATOM	1338	NH2 ARG B 150		
4559	ATOM	1339	N ARG B 151		
4560	ATOM	1340	CA ARG B 151		
4561	ATOM	1341	C ARG B 151		
4562	ATOM	1342	O ARG B 151		
4563	ATOM	1343	CB ARG B 151		
4564	ATOM	1344	CG ARG B 151		
4565	ATOM	1345	CD ARG B 151		
4566	ATOM	1346	NE ARG B 151		
4567	ATOM	1347	CZ ARG B 151		
4568	ATOM	1348	NH1 ARG B 151		
4569	ATOM	1349	NH2 ARG B 151		
4570	ATOM	1350	N HIS B 152		
4571	ATOM	1351	CA HIS B 152		
4572	ATOM	1352	C HIS B 152		
4573	ATOM	1353	O HIS B 152		
4574	ATOM	1354	CB HIS B 152		
4575	ATOM	1355	CG HIS B 152		
4576	ATOM	1356	ND1 HIS B 152		
4577	ATOM	1357	CD2 HIS B 152		
4578	ATOM	1358	CE1 HIS B 152		
4579	ATOM	1359	NE2 HIS B 152		
4580	ATOM	1360	N VAL B 153		
4581	ATOM	1361	CA VAL B 153		
4582	ATOM	1362	C VAL B 153		
4583	ATOM	1363	O VAL B 153		
4584	ATOM	1364	CB VAL B 153		

ATOM 1365 CG1 VAL B 153
ATOM 1366 CG2 VAL B 153
ATOM 1367 N GLU B 154
ATOM 1368 CA GLU B 154
ATOM 1369 C GLU B 154
ATOM 1370 O GLU B 154
ATOM 1371 CB GLU B 154
ATOM 1372 CG GLU B 154
ATOM 1373 CD GLU B 154
ATOM 1374 OE1 GLU B 154
ATOM 1375 OE2 GLU B 154
ATOM 1376 N ALA B 155
ATOM 1377 CA ALA B 155
ATOM 1378 C ALA B 155
ATOM 1379 O ALA B 155
ATOM 1380 CB ALA B 155
ATOM 1381 N VAL B 156
ATOM 1382 CA VAL B 156
ATOM 1383 C VAL B 156
ATOM 1384 O VAL B 156
ATOM 1385 CB VAL B 156
ATOM 1386 CG1 VAL B 156
ATOM 1387 CG2 VAL B 156
ATOM 1388 N TRP B 157
ATOM 1389 CA TRP B 157
ATOM 1390 C TRP B 157
-13.088 -0.323 40.309 1.00 0.80
-14.556 -1.911 39.571 1.00 0.80
-5.688 -2.968 40.976 1.00 0.80
-4.471 -2.876 41.796 1.00 0.80
-3.283 -2.414 40.944 1.00 0.80
-2.602 -1.455 41.298 1.00 0.80
-4.197 -4.243 42.415 1.00 0.80
-2.997 -4.195 43.363 1.00 0.80
-2.745 -5.574 43.969 1.00 0.80
-3.876 -5.950 44.842 1.00 0.80
-4.036 -5.591 46.118 1.00 0.80
-3.139 -4.829 46.733 1.00 0.80
-5.096 -6.006 46.799 1.00 0.80
-3.143 -3.025 39.771 1.00 0.88

4625	-2.073	-2.681	38.816	1.00	0.88
4626	-2.160	-1.230	38.339	1.00	0.88
4627	-1.148	-0.522	38.359	1.00	0.88
4628	-2.100	-3.618	37.607	1.00	0.88
4629	-1.696	-5.039	37.998	1.00	0.88
4630	-0.515	-5.419	38.468	1.00	0.88
4631	-2.445	-6.127	37.876	1.00	0.88
4632	-0.534	-6.736	38.641	1.00	0.88
4633	-1.740	-7.170	38.286	1.00	0.88
4634	-3.377	-0.767	38.087	1.00	0.90
4635	-3.619	0.618	37.630	1.00	0.90
4636	-3.340	1.644	38.745	1.00	0.90
4637	-2.700	2.667	38.549	1.00	0.90
4638	-5.031	0.816	37.051	1.00	0.90
4639	-5.294	-0.085	35.842	1.00	0.90
4640	-6.123	0.604	38.092	1.00	0.90
4641	-3.786	1.296	39.954	1.00	0.83
4642	-3.585	2.161	41.122	1.00	0.83
4643	-2.086	2.247	41.444	1.00	0.83
4644	-1.587	3.334	41.730	1.00	0.83
4645	-4.423	1.610	42.284	1.00	0.83
4646	-4.307	2.435	43.574	1.00	0.83
4647	-2.936	2.317	44.256	1.00	0.83
4648	-2.337	1.219	44.171	1.00	0.83
4649	-2.528	3.323	44.870	1.00	0.83
4650	-1.402	1.111	41.319	1.00	0.91
4651	0.036	0.989	41.620	1.00	0.91
4652	0.854	1.897	40.698	1.00	0.91
4653	1.482	2.862	41.159	1.00	0.91
4654	0.480	-0.470	41.463	1.00	0.91
4655	0.697	1.672	39.401	1.00	0.87
4656	1.373	2.464	38.351	1.00	0.87
4657	1.173	3.976	38.529	1.00	0.87
4658	2.149	4.712	38.635	1.00	0.87
4659	0.946	2.059	36.929	1.00	0.87
4660	1.315	0.620	36.626	1.00	0.87
4661	-0.549	2.178	36.672	1.00	0.87
4662	-0.083	4.381	38.744	1.00	0.83
4663	-0.405	5.807	38.851	1.00	0.83
4664	0.209	6.436	40.104	1.00	0.83

4665	ATOM	1391	O	TRP	B	157
4666	ATOM	1392	CB	TRP	B	157
4667	ATOM	1393	CG	TRP	B	157
4668	ATOM	1394	CD1	TRP	B	157
4669	ATOM	1395	CD2	TRP	B	157
4670	ATOM	1396	NE1	TRP	B	157
4671	ATOM	1397	CE2	TRP	B	157
4672	ATOM	1398	CE3	TRP	B	157
4673	ATOM	1399	CZ2	TRP	B	157
4674	ATOM	1400	CZ3	TRP	B	157
4675	ATOM	1401	CH2	TRP	B	157
4676	ATOM	1402	N	SER	B	158
4677	ATOM	1403	CA	SER	B	158
4678	ATOM	1404	C	SER	B	158
4679	ATOM	1405	O	SER	B	158
4680	ATOM	1406	CB	SER	B	158
4681	ATOM	1407	OG	SER	B	158
4682	ATOM	1408	N	LYS	B	159
4683	ATOM	1409	CA	LYS	B	159
4684	ATOM	1410	C	LYS	B	159
4685	ATOM	1411	O	LYS	B	159
4686	ATOM	1412	CB	LYS	B	159
4687	ATOM	1413	CG	LYS	B	159
4688	ATOM	1414	CD	LYS	B	159
4689	ATOM	1415	CE	LYS	B	159
4690	ATOM	1416	NZ	LYS	B	159
4691	ATOM	1417	N	ILE	B	160
4692	ATOM	1418	CA	ILE	B	160
4693	ATOM	1419	C	ILE	B	160
4694	ATOM	1420	O	ILE	B	160
4695	ATOM	1421	CB	ILE	B	160
4696	ATOM	1422	CG1	ILE	B	160
4697	ATOM	1423	CG2	ILE	B	160
4698	ATOM	1424	CD1	ILE	B	160
4699	ATOM	1425	N	ASP	B	161
4700	ATOM	1426	CA	ASP	B	161
4701	ATOM	1427	C	ASP	B	161
4702	ATOM	1428	O	ASP	B	161
4703	ATOM	1429	CB	ASP	B	161
4704	ATOM	1430	CG	ASP	B	161

ATOM 1431 OD1 ASP B 161
ATOM 1432 OD2 ASP B 161
ATOM 1433 N ILE B 162
ATOM 1434 CA ILE B 162
ATOM 1435 C ILE B 162
ATOM 1436 O ILE B 162
ATOM 1437 CB ILE B 162
ATOM 1438 CG1 ILE B 162
ATOM 1439 CG2 ILE B 162
ATOM 1440 CD1 ILE B 162
ATOM 1441 N ASP B 163
ATOM 1442 CA ASP B 163
ATOM 1443 C ASP B 163
ATOM 1444 O ASP B 163
0.725 7.532 40.056 1.00 0.83
-1.908 6.074 38.851 1.00 0.83
-2.141 7.587 38.941 1.00 0.83
-2.136 8.458 37.924 1.00 0.83
-2.354 8.316 40.082 1.00 0.83
-2.400 9.669 38.364 1.00 0.83
-2.530 9.639 39.709 1.00 0.83
-2.354 7.982 41.446 1.00 0.83
-2.722 10.618 40.634 1.00 0.83
-2.554 8.969 42.386 1.00 0.83
-2.746 10.290 42.000 1.00 0.83
0.159 5.731 41.234 1.00 0.87
0.743 6.292 42.467 1.00 0.87
2.270 6.455 42.409 1.00 0.87
2.846 7.187 43.210 1.00 0.87
0.312 5.508 43.707 1.00 0.87
0.685 4.135 43.588 1.00 0.87
2.909 5.763 41.467 1.00 0.87
4.366 5.888 41.249 1.00 0.87
4.720 6.632 39.954 1.00 0.87
5.901 6.714 39.579 1.00 0.87
4.990 4.497 41.200 1.00 0.87
4.873 3.744 42.527 1.00 0.87
5.592 2.393 42.447 1.00 0.87
7.113 2.530 42.286 1.00 0.87
7.722 3.163 43.465 1.00 0.87

4745	3.726	7.223	39.309	1.00	0.84
4746	3.933	7.987	38.069	1.00	0.84
4747	4.797	9.224	38.347	1.00	0.84
4748	4.522	10.032	39.232	1.00	0.84
4749	2.596	8.355	37.386	1.00	0.84
4750	2.816	8.964	35.999	1.00	0.84
4751	1.755	9.371	38.169	1.00	0.84
4752	3.491	7.986	35.037	1.00	0.84
4753	5.752	9.425	37.454	1.00	0.86
4754	6.539	10.656	37.470	1.00	0.86
4755	6.137	11.457	36.224	1.00	0.86
4756	6.700	11.355	35.160	1.00	0.86
4757	8.027	10.292	37.569	1.00	0.86
4758	8.961	11.509	37.570	1.00	0.86
4759	8.440	12.645	37.677	1.00	0.86
4760	10.174	11.295	37.361	1.00	0.86
4761	5.121	12.303	36.417	1.00	0.81
4762	4.502	13.056	35.306	1.00	0.81
4763	5.495	13.952	34.547	1.00	0.81
4764	5.518	13.981	33.324	1.00	0.81
4765	3.316	13.891	35.825	1.00	0.81
4766	3.783	14.933	36.855	1.00	0.81
4767	2.241	12.957	36.404	1.00	0.81
4768	2.733	15.973	37.248	1.00	0.81
4769	6.409	14.541	35.326	1.00	0.88
4770	7.442	15.452	34.812	1.00	0.88
4771	8.405	14.761	33.842	1.00	0.88
4772	8.942	15.407	32.952	1.00	0.88
4773	ATOM	1445	CB ASP B 163		
4774	ATOM	1446	CG ASP B 163		
4775	ATOM	1447	OD1 ASP B 163		
4776	ATOM	1448	OD2 ASP B 163		
4777	ATOM	1449	N VAL B 164		
4778	ATOM	1450	CA VAL B 164		
4779	ATOM	1451	C VAL B 164		
4780	ATOM	1452	O VAL B 164		
4781	ATOM	1453	CB VAL B 164		
4782	ATOM	1454	CG1 VAL B 164		
4783	ATOM	1455	CG2 VAL B 164		
4784	ATOM	1456	N CYS B 165		

4785	ATOM	1457	CA	CYS	B	165
4786	ATOM	1458	C	CYS	B	165
4787	ATOM	1459	O	CYS	B	165
4788	ATOM	1460	CB	CYS	B	165
4789	ATOM	1461	SG	CYS	B	165
4790	ATOM	1462	N	GLY	B	166
4791	ATOM	1463	CA	GLY	B	166
4792	ATOM	1464	C	GLY	B	166
4793	ATOM	1465	O	GLY	B	166
4794	ATOM	1466	N	PRO	B	167
4795	ATOM	1467	CA	PRO	B	167
4796	ATOM	1468	C	PRO	B	167
4797	ATOM	1469	O	PRO	B	167
4798	ATOM	1470	CB	PRO	B	167
4799	ATOM	1471	CG	PRO	B	167
4800	ATOM	1472	CD	PRO	B	167
4801	ATOM	1473	N	LEU	B	168
4802	ATOM	1474	CA	LEU	B	168
4803	ATOM	1475	C	LEU	B	168
4804	ATOM	1476	O	LEU	B	168
4805	ATOM	1477	CB	LEU	B	168
4806	ATOM	1478	CG	LEU	B	168
4807	ATOM	1479	CD1	LEU	B	168
4808	ATOM	1480	CD2	LEU	B	168
4809	ATOM	1481	N	ALA	B	169
4810	ATOM	1482	CA	ALA	B	169
4811	ATOM	1483	C	ALA	B	169
4812	ATOM	1484	O	ALA	B	169
4813	ATOM	1485	CB	ALA	B	169
4814	ATOM	1486	N	LEU	B	170
4815	ATOM	1487	CA	LEU	B	170
4816	ATOM	1488	C	LEU	B	170
4817	ATOM	1489	O	LEU	B	170
4818	ATOM	1490	CB	LEU	B	170
4819	ATOM	1491	CG	LEU	B	170
4820	ATOM	1492	CD1	LEU	B	170
4821	ATOM	1493	CD2	LEU	B	170
4822	ATOM	1494	N	GLN	B	171
4823	ATOM	1495	CA	GLN	B	171
4824	ATOM	1496	C	GLN	B	171

ATOM 1497 O GLN B 171
ATOM 1498 CB GLN B 171
8.220 16.087 35.973 1.00 0.88
7.421 17.166 36.716 1.00 0.88
6.175 17.051 36.762 1.00 0.88
8.082 18.068 37.268 1.00 0.88
8.576 13.452 34.036 1.00 0.87
9.438 12.633 33.167 1.00 0.87
8.670 12.120 31.935 1.00 0.87
8.760 12.663 30.850 1.00 0.87
10.167 11.528 33.962 1.00 0.87
9.276 10.403 34.469 1.00 0.87
11.249 10.867 33.111 1.00 0.87
7.781 11.153 32.162 1.00 0.90
7.061 10.426 31.116 1.00 0.90
6.193 11.344 30.253 1.00 0.90
6.156 11.172 29.047 1.00 0.90
6.182 9.357 31.759 1.00 0.90
5.240 10.044 33.161 1.00 0.90
5.644 12.397 30.893 1.00 0.94
4.749 13.347 30.204 1.00 0.94
5.398 13.902 28.922 1.00 0.94
4.970 13.510 27.820 1.00 0.94
6.474 14.685 29.050 1.00 0.95
7.222 15.269 27.919 1.00 0.95
7.777 14.208 26.966 1.00 0.95
7.579 14.341 25.772 1.00 0.95
8.390 16.028 28.540 1.00 0.95
7.852 16.426 29.909 1.00 0.95
7.026 15.213 30.320 1.00 0.95
8.231 13.079 27.532 1.00 0.88
8.849 11.982 26.761 1.00 0.88
7.882 11.304 25.797 1.00 0.88
8.208 11.060 24.634 1.00 0.88
9.437 10.935 27.710 1.00 0.88
10.851 11.270 28.202 1.00 0.88
10.955 12.584 28.981 1.00 0.88
11.365 10.115 29.059 1.00 0.88
6.653 11.147 26.246 1.00 0.91
5.621 10.476 25.454 1.00 0.91

4865	5.124	11.336	24.290	1.00	0.91
4866	5.121	10.888	23.147	1.00	0.91
4867	4.482	10.187	26.397	1.00	0.91
4868	4.840	12.603	24.596	1.00	0.88
4869	4.348	13.549	23.583	1.00	0.88
4870	5.429	13.895	22.554	1.00	0.88
4871	5.177	13.848	21.345	1.00	0.88
4872	3.824	14.825	24.249	1.00	0.88
4873	3.194	15.776	23.222	1.00	0.88
4874	1.969	15.153	22.542	1.00	0.88
4875	2.820	17.094	23.892	1.00	0.88
4876	6.642	14.119	23.041	1.00	0.84
4877	7.794	14.422	22.169	1.00	0.84
4878	8.133	13.235	21.253	1.00	0.84
4879	8.309	13.413	20.051	1.00	0.84
4880	9.017	14.850	22.985	1.00	0.84

4881	ATOM	1499	CG	GLN	B	171
4882	ATOM	1500	CD	GLN	B	171
4883	ATOM	1501	OE1	GLN	B	171
4884	ATOM	1502	NE2	GLN	B	171
4885	ATOM	1503	N	ARG	B	172
4886	ATOM	1504	CA	ARG	B	172
4887	ATOM	1505	C	ARG	B	172
4888	ATOM	1506	O	ARG	B	172
4889	ATOM	1507	CB	ARG	B	172
4890	ATOM	1508	CG	ARG	B	172
4891	ATOM	1509	CD	ARG	B	172
4892	ATOM	1510	NE	ARG	B	172
4893	ATOM	1511	CZ	ARG	B	172
4894	ATOM	1512	NH1	ARG	B	172
4895	ATOM	1513	NH2	ARG	B	172
4896	ATOM	1514	N	CYS	B	173
4897	ATOM	1515	CA	CYS	B	173
4898	ATOM	1516	C	CYS	B	173
4899	ATOM	1517	O	CYS	B	173
4900	ATOM	1518	CB	CYS	B	173
4901	ATOM	1519	SG	CYS	B	173
4902	ATOM	1520	N	LEU	B	174
4903	ATOM	1521	CA	LEU	B	174
4904	ATOM	1522	C	LEU	B	174

ATOM	1523	O	LEU	B	174	
ATOM	1524	CB	LEU	B	174	
ATOM	1525	CG	LEU	B	174	
ATOM	1526	CD1	LEU	B	174	
ATOM	1527	CD2	LEU	B	174	
ATOM	1528	N	ILE	B	175	
ATOM	1529	CA	ILE	B	175	
ATOM	1530	C	ILE	B	175	
ATOM	1531	O	ILE	B	175	
ATOM	1532	CB	ILE	B	175	
ATOM	1533	CG1	ILE	B	175	
ATOM	1534	CG2	ILE	B	175	
ATOM	1535	CD1	ILE	B	175	
ATOM	1536	N	VAL	B	176	
ATOM	1537	CA	VAL	B	176	
ATOM	1538	C	VAL	B	176	
ATOM	1539	O	VAL	B	176	
ATOM	1540	CB	VAL	B	176	
ATOM	1541	CG1	VAL	B	176	
ATOM	1542	CG2	VAL	B	176	
ATOM	1543	N	TYR	B	177	
ATOM	1544	CA	TYR	B	177	
ATOM	1545	C	TYR	B	177	
ATOM	1546	O	TYR	B	177	
ATOM	1547	CB	TYR	B	177	
ATOM	1548	CG	TYR	B	177	
ATOM	1549	CD1	TYR	B	177	
ATOM	1550	CD2	TYR	B	177	
ATOM	1551	CE1	TYR	B	177	
ATOM	1552	CE2	TYR	B	177	

9.606	13.707	23.813	1.00	0.84
10.729	14.163	24.723	1.00	0.84
10.613	15.041	25.563	1.00	0.84
11.837	13.486	24.551	1.00	0.84
8.054	12.023	21.811	1.00	0.84
8.308	10.795	21.039	1.00	0.84
7.205	10.589	19.991	1.00	0.84
7.477	10.169	18.868	1.00	0.84
8.406	9.586	21.971	1.00	0.84
8.726	8.310	21.189	1.00	0.84

4945	8.935	7.121	22.119	1.00	0.84
4946	10.219	7.276	22.822	1.00	0.84
4947	11.317	6.549	22.610	1.00	0.84
4948	11.344	5.579	21.712	1.00	0.84
4949	12.438	6.823	23.259	1.00	0.84
4950	5.996	11.020	20.336	1.00	0.90
4951	4.830	10.927	19.445	1.00	0.90
4952	4.952	11.869	18.242	1.00	0.90
4953	4.662	11.460	17.119	1.00	0.90
4954	3.558	11.242	20.228	1.00	0.90
4955	2.036	11.040	19.235	1.00	0.90
4956	5.462	13.073	18.488	1.00	0.86
4957	5.656	14.084	17.432	1.00	0.86
4958	6.743	13.707	16.422	1.00	0.86
4959	6.718	14.138	15.278	1.00	0.86
4960	5.964	15.457	18.038	1.00	0.86
4961	4.803	16.024	18.866	1.00	0.86
4962	5.182	17.405	19.394	1.00	0.86
4963	3.506	16.129	18.059	1.00	0.86
4964	7.680	12.883	16.891	1.00	0.83
4965	8.800	12.407	16.066	1.00	0.83
4966	8.397	11.154	15.275	1.00	0.83
4967	8.685	11.062	14.086	1.00	0.83
4968	10.035	12.180	16.958	1.00	0.83
4969	10.481	13.533	17.531	1.00	0.83
4970	11.183	11.486	16.201	1.00	0.83
4971	11.495	13.411	18.668	1.00	0.83
4972	7.798	10.192	15.968	1.00	0.87
4973	7.436	8.892	15.366	1.00	0.87
4974	6.214	9.025	14.444	1.00	0.87
4975	6.158	8.377	13.400	1.00	0.87
4976	7.221	7.826	16.458	1.00	0.87
4977	6.887	6.452	15.865	1.00	0.87
4978	8.476	7.667	17.320	1.00	0.87
4979	5.230	9.804	14.873	1.00	0.91
4980	4.006	10.027	14.085	1.00	0.91
4981	3.865	11.539	13.850	1.00	0.91
4982	3.005	12.189	14.463	1.00	0.91
4983	2.788	9.413	14.784	1.00	0.91
4984	3.044	7.969	15.219	1.00	0.91

4985	3.197	6.946	14.290	1.00	0.91			
4986	3.086	7.693	16.576	1.00	0.91			
4987	3.373	5.640	14.722	1.00	0.91			
4988	3.268	6.389	17.013	1.00	0.91			
4989	ATOM	1553	CZ	TYR	B	177		
4990	ATOM	1554	OH	TYR	B	177		
4991	ATOM	1555	N	PRO	B	178		
4992	ATOM	1556	CA	PRO	B	178		
4993	ATOM	1557	C	PRO	B	178		
4994	ATOM	1558	O	PRO	B	178		
4995	ATOM	1559	CB	PRO	B	178		
4996	ATOM	1560	CG	PRO	B	178		
4997	ATOM	1561	CD	PRO	B	178		
4998	ATOM	1562	N	TRP	B	179		
4999	ATOM	1563	CA	TRP	B	179		
5000	ATOM	1564	C	TRP	B	179		
5001	ATOM	1565	O	TRP	B	179		
5002	ATOM	1566	CB	TRP	B	179		
5003	ATOM	1567	CG	TRP	B	179		
5004	ATOM	1568	CD1	TRP	B	179		
5005	ATOM	1569	CD2	TRP	B	179		
5006	ATOM	1570	NE1	TRP	B	179		
5007	ATOM	1571	CE2	TRP	B	179		
5008	ATOM	1572	CE3	TRP	B	179		
5009	ATOM	1573	CZ2	TRP	B	179		
5010	ATOM	1574	CZ3	TRP	B	179		
5011	ATOM	1575	CH2	TRP	B	179		
5012	ATOM	1576	N	THR	B	180		
5013	ATOM	1577	CA	THR	B	180		
5014	ATOM	1578	C	THR	B	180		
5015	ATOM	1579	O	THR	B	180		
5016	ATOM	1580	CB	THR	B	180		
5017	ATOM	1581	OG1	THR	B	180		
5018	ATOM	1582	CG2	THR	B	180		
5019	ATOM	1583	N	GLN	B	181		
5020	ATOM	1584	CA	GLN	B	181		
5021	ATOM	1585	C	GLN	B	181		
5022	ATOM	1586	O	GLN	B	181		
5023	ATOM	1587	CB	GLN	B	181		
5024	ATOM	1588	CG	GLN	B	181		

5025	ATOM	1589	CD	GLN B 181		
5026	ATOM	1590	OE1	GLN B 181		
5027	ATOM	1591	NE2	GLN B 181		
5028	ATOM	1592	N	ARG B 182		
5029	ATOM	1593	CA	ARG B 182		
5030	ATOM	1594	C	ARG B 182		
5031	ATOM	1595	O	ARG B 182		
5032	ATOM	1596	CB	ARG B 182		
5033	ATOM	1597	CG	ARG B 182		
5034	ATOM	1598	CD	ARG B 182		
5035	ATOM	1599	NE	ARG B 182		
5036	ATOM	1600	CZ	ARG B 182		
5037	ATOM	1601	NH1	ARG B 182		
5038	ATOM	1602	NH2	ARG B 182		
5039	ATOM	1603	N	TYR B 183		
5040	ATOM	1604	CA	TYR B 183		
5041	ATOM	1605	C	TYR B 183		
5042	ATOM	1606	O	TYR B 183		
5043	3.404	5.365	16.083	1.00	0.91	
5044	3.585	4.088	16.503	1.00	0.91	
5045	4.631	12.064	12.893	1.00	0.89	
5046	4.776	13.510	12.612	1.00	0.89	
5047	3.465	14.235	12.288	1.00	0.89	
5048	3.358	15.440	12.498	1.00	0.89	
5049	5.697	13.592	11.399	1.00	0.89	
5050	6.537	12.327	11.516	1.00	0.89	
5051	5.514	11.299	11.984	1.00	0.89	
5052	2.471	13.508	11.783	1.00	0.80	
5053	1.158	14.098	11.450	1.00	0.80	
5054	0.432	14.687	12.670	1.00	0.80	
5055	-0.371	15.605	12.540	1.00	0.80	
5056	0.270	13.104	10.691	1.00	0.80	
5057	0.190	11.729	11.355	1.00	0.80	
5058	-0.808	11.263	12.096	1.00	0.80	
5059	1.084	10.688	11.149	1.00	0.80	
5060	-0.592	9.971	12.355	1.00	0.80	
5061	0.548	9.590	11.797	1.00	0.80	
5062	2.276	10.590	10.455	1.00	0.80	
5063	1.220	8.370	11.743	1.00	0.80	
5064	2.941	9.371	10.387	1.00	0.80	

2.407 8.264 11.033 1.00 0.80
0.821 14.233 13.862 1.00 0.85
0.274 14.749 15.137 1.00 0.85
0.767 16.165 15.479 1.00 0.85
0.189 16.833 16.337 1.00 0.85
0.557 13.807 16.311 1.00 0.85
1.966 13.706 16.514 1.00 0.85
-0.069 12.428 16.080 1.00 0.85
1.801 16.627 14.780 1.00 0.76
2.256 18.031 14.856 1.00 0.76
1.215 19.019 14.310 1.00 0.76
1.327 20.227 14.542 1.00 0.76
3.529 18.206 14.046 1.00 0.76
4.734 17.471 14.643 1.00 0.76
5.956 17.554 13.720 1.00 0.76
6.823 16.696 13.707 1.00 0.76
6.054 18.625 12.955 1.00 0.76
0.183 18.474 13.664 1.00 0.69
-0.930 19.229 13.058 1.00 0.69
-1.540 20.275 14.009 1.00 0.69
-1.872 21.373 13.582 1.00 0.69
-2.022 18.242 12.623 1.00 0.69
-3.175 18.949 11.906 1.00 0.69
-4.272 17.986 11.462 1.00 0.69
-5.318 18.792 10.809 1.00 0.69
-6.638 18.626 10.910 1.00 0.69
-7.171 17.586 11.537 1.00 0.69
-7.446 19.595 10.500 1.00 0.69
-1.567 19.951 15.308 1.00 0.68
-2.206 20.802 16.322 1.00 0.68
-1.204 21.563 17.226 1.00 0.68
-1.508 22.031 18.302 1.00 0.68
ATOM 1607 CB TYR B 183
ATOM 1608 CG TYR B 183
ATOM 1609 CD1 TYR B 183
ATOM 1610 CD2 TYR B 183
ATOM 1611 CE1 TYR B 183
ATOM 1612 CE2 TYR B 183
ATOM 1613 CZ TYR B 183
ATOM 1614 OH TYR B 183

5105	ATOM	1615	N	PHE	B	184
5106	ATOM	1616	CA	PHE	B	184
5107	ATOM	1617	C	PHE	B	184
5108	ATOM	1618	O	PHE	B	184
5109	ATOM	1619	CB	PHE	B	184
5110	ATOM	1620	CG	PHE	B	184
5111	ATOM	1621	CD1	PHE	B	184
5112	ATOM	1622	CD2	PHE	B	184
5113	ATOM	1623	CE1	PHE	B	184
5114	ATOM	1624	CE2	PHE	B	184
5115	ATOM	1625	CZ	PHE	B	184
5116	ATOM	1626	N	GLY	B	185
5117	ATOM	1627	CA	GLY	B	185
5118	ATOM	1628	C	GLY	B	185
5119	ATOM	1629	O	GLY	B	185
5120	ATOM	1630	N	SER	B	186
5121	ATOM	1631	CA	SER	B	186
5122	ATOM	1632	C	SER	B	186
5123	ATOM	1633	O	SER	B	186
5124	ATOM	1634	CB	SER	B	186
5125	ATOM	1635	OG	SER	B	186
5126	ATOM	1636	N	PHE	B	187
5127	ATOM	1637	CA	PHE	B	187
5128	ATOM	1638	C	PHE	B	187
5129	ATOM	1639	O	PHE	B	187
5130	ATOM	1640	CB	PHE	B	187
5131	ATOM	1641	CG	PHE	B	187
5132	ATOM	1642	CD1	PHE	B	187
5133	ATOM	1643	CD2	PHE	B	187
5134	ATOM	1644	CE1	PHE	B	187
5135	ATOM	1645	CE2	PHE	B	187
5136	ATOM	1646	CZ	PHE	B	187
5137	ATOM	1647	N	GLY	B	188
5138	ATOM	1648	CA	GLY	B	188
5139	ATOM	1649	C	GLY	B	188
5140	ATOM	1650	O	GLY	B	188
5141	ATOM	1651	N	ASP	B	189
5142	ATOM	1652	CA	ASP	B	189
5143	ATOM	1653	C	ASP	B	189
5144	ATOM	1654	O	ASP	B	189

ATOM 1655 CB ASP B 189
ATOM 1656 CG ASP B 189
ATOM 1657 OD1 ASP B 189
ATOM 1658 OD2 ASP B 189
ATOM 1659 N LEU B 190
ATOM 1660 CA LEU B 190
-3.146 19.900 17.145 1.00 0.68
-3.882 20.628 18.266 1.00 0.68
-5.025 21.366 17.996 1.00 0.68
-3.356 20.626 19.573 1.00 0.68
-5.636 22.090 18.998 1.00 0.68
-3.949 21.360 20.563 1.00 0.68
-5.094 22.102 20.288 1.00 0.68
-5.623 22.867 21.264 1.00 0.68
0.008 21.813 16.717 1.00 0.64
1.014 22.449 17.589 1.00 0.64
1.774 23.619 16.956 1.00 0.64
2.896 23.944 17.340 1.00 0.64
1.948 21.383 18.174 1.00 0.64
1.171 20.388 19.038 1.00 0.64
0.723 20.758 20.297 1.00 0.64
0.907 19.114 18.554 1.00 0.64
0.016 19.859 21.082 1.00 0.64
0.206 18.208 19.340 1.00 0.64
-0.240 18.579 20.603 1.00 0.64
1.108 24.280 16.000 1.00 0.59
1.659 25.506 15.392 1.00 0.59
1.441 26.626 16.406 1.00 0.59
0.278 26.767 16.804 1.00 0.59
2.527 27.241 16.877 1.00 0.53
2.591 28.264 17.975 1.00 0.53
3.508 27.779 19.098 1.00 0.53
4.170 28.571 19.765 1.00 0.53
1.228 28.551 18.620 1.00 0.53
1.138 29.543 19.625 1.00 0.53
3.623 26.446 19.170 1.00 0.62
4.550 25.738 20.058 1.00 0.62
6.026 26.087 19.873 1.00 0.62
6.837 25.921 20.759 1.00 0.62
4.298 24.227 20.119 1.00 0.62

3.073 23.874 20.974 1.00 0.62
1.910 24.648 20.980 1.00 0.62
3.174 22.809 21.860 1.00 0.62
0.878 24.368 21.861 1.00 0.62
2.145 22.530 22.750 1.00 0.62
0.992 23.309 22.750 1.00 0.62
6.299 26.700 18.715 1.00 0.69
7.661 27.045 18.304 1.00 0.69
8.034 26.142 17.129 1.00 0.69
7.167 25.649 16.402 1.00 0.69
9.327 25.895 17.005 1.00 0.70
9.821 25.059 15.903 1.00 0.70
9.861 23.588 16.325 1.00 0.70
10.446 23.241 17.355 1.00 0.70
11.196 25.539 15.419 1.00 0.70
11.633 24.848 14.119 1.00 0.70
10.847 24.031 13.596 1.00 0.70
12.745 25.154 13.650 1.00 0.70
9.298 22.769 15.455 1.00 0.73
9.210 21.305 15.618 1.00 0.73
ATOM 1661 C LEU B 190
ATOM 1662 O LEU B 190
ATOM 1663 CB LEU B 190
ATOM 1664 CG LEU B 190
ATOM 1665 CD1 LEU B 190
ATOM 1666 CD2 LEU B 190
ATOM 1667 N SER B 191
ATOM 1668 CA SER B 191
ATOM 1669 C SER B 191
ATOM 1670 O SER B 191
ATOM 1671 CB SER B 191
ATOM 1672 OG SER B 191
ATOM 1673 N THR B 192
ATOM 1674 CA THR B 192
ATOM 1675 C THR B 192
ATOM 1676 O THR B 192
ATOM 1677 CB THR B 192
ATOM 1678 OG1 THR B 192
ATOM 1679 CG2 THR B 192
ATOM 1680 N ASP B 193

ATOM	1681	CA	ASP	B	193
ATOM	1682	C	ASP	B	193
ATOM	1683	O	ASP	B	193
ATOM	1684	CB	ASP	B	193
ATOM	1685	CG	ASP	B	193
ATOM	1686	OD1	ASP	B	193
ATOM	1687	OD2	ASP	B	193
ATOM	1688	N	ALA	B	194
ATOM	1689	CA	ALA	B	194
ATOM	1690	C	ALA	B	194
ATOM	1691	O	ALA	B	194
ATOM	1692	CB	ALA	B	194
ATOM	1693	N	ALA	B	195
ATOM	1694	CA	ALA	B	195
ATOM	1695	C	ALA	B	195
ATOM	1696	O	ALA	B	195
ATOM	1697	CB	ALA	B	195
ATOM	1698	N	ILE	B	196
ATOM	1699	CA	ILE	B	196
ATOM	1700	C	ILE	B	196
ATOM	1701	O	ILE	B	196
ATOM	1702	CB	ILE	B	196
ATOM	1703	CG1	ILE	B	196
ATOM	1704	CG2	ILE	B	196
ATOM	1705	CD1	ILE	B	196
ATOM	1706	N	VAL	B	197
ATOM	1707	CA	VAL	B	197
ATOM	1708	C	VAL	B	197
ATOM	1709	O	VAL	B	197
ATOM	1710	CB	VAL	B	197
ATOM	1711	CG1	VAL	B	197
ATOM	1712	CG2	VAL	B	197
ATOM	1713	N	GLY	B	198
ATOM	1714	CA	GLY	B	198

9.672	20.568	14.352	1.00	0.73
9.315	19.418	14.105	1.00	0.73
7.747	20.955	15.918	1.00	0.73
7.152	21.758	17.081	1.00	0.73
5.679	21.398	17.246	1.00	0.73
7.906	21.516	18.390	1.00	0.73

5265	10.473	21.251	13.540	1.00	0.73
5266	10.935	20.729	12.240	1.00	0.73
5267	11.959	19.593	12.357	1.00	0.73
5268	12.201	18.878	11.388	1.00	0.73
5269	11.476	21.846	11.343	1.00	0.73
5270	12.588	22.499	11.964	1.00	0.73
5271	12.628	19.510	13.504	1.00	0.76
5272	13.605	18.439	13.764	1.00	0.76
5273	13.409	17.824	15.150	1.00	0.76
5274	12.985	18.509	16.093	1.00	0.76
5275	15.049	18.930	13.534	1.00	0.76
5276	15.948	17.822	13.646	1.00	0.76
5277	15.479	20.047	14.492	1.00	0.76
5278	13.941	16.624	15.301	1.00	0.80
5279	13.860	15.859	16.556	1.00	0.80
5280	14.431	16.634	17.759	1.00	0.80
5281	13.773	16.731	18.789	1.00	0.80
5282	14.583	14.527	16.361	1.00	0.80
5283	14.506	13.599	17.581	1.00	0.80
5284	14.548	14.094	18.725	1.00	0.80
5285	14.390	12.384	17.349	1.00	0.80
5286	15.605	17.244	17.574	1.00	0.78
5287	16.264	18.020	18.643	1.00	0.78
5288	15.443	19.245	19.088	1.00	0.78
5289	15.347	19.563	20.265	1.00	0.78
5290	17.650	18.469	18.183	1.00	0.78
5291	14.781	19.881	18.118	1.00	0.79
5292	13.921	21.045	18.397	1.00	0.79
5293	12.679	20.651	19.210	1.00	0.79
5294	12.328	21.321	20.174	1.00	0.79
5295	13.483	21.705	17.088	1.00	0.79
5296	12.130	19.480	18.877	1.00	0.76
5297	10.918	18.939	19.528	1.00	0.76
5298	11.210	18.533	20.975	1.00	0.76
5299	10.456	18.906	21.878	1.00	0.76
5300	10.339	17.758	18.723	1.00	0.76
5301	9.866	18.255	17.350	1.00	0.76
5302	9.205	17.044	19.488	1.00	0.76
5303	9.337	17.168	16.406	1.00	0.76
5304	12.291	17.788	21.165	1.00	0.79

12.693 17.303 22.500 1.00 0.79
12.871 18.485 23.475 1.00 0.79
12.320 18.519 24.555 1.00 0.79
13.968 16.432 22.447 1.00 0.79
13.758 15.191 21.582 1.00 0.79
15.235 17.149 21.967 1.00 0.79
13.562 19.509 22.920 1.00 0.82
13.917 20.742 23.630 1.00 0.82
ATOM 1715 C GLY B 198
ATOM 1716 O GLY B 198
ATOM 1717 N ASN B 199
ATOM 1718 CA ASN B 199
ATOM 1719 C ASN B 199
ATOM 1720 O ASN B 199
ATOM 1721 CB ASN B 199
ATOM 1722 CG ASN B 199
ATOM 1723 OD1 ASN B 199
ATOM 1724 ND2 ASN B 199
ATOM 1725 N PRO B 200
ATOM 1726 CA PRO B 200
ATOM 1727 C PRO B 200
ATOM 1728 O PRO B 200
ATOM 1729 CB PRO B 200
ATOM 1730 CG PRO B 200
ATOM 1731 CD PRO B 200
ATOM 1732 N LYS B 201
ATOM 1733 CA LYS B 201
ATOM 1734 C LYS B 201
ATOM 1735 O LYS B 201
ATOM 1736 CB LYS B 201
ATOM 1737 CG LYS B 201
ATOM 1738 CD LYS B 201
ATOM 1739 CE LYS B 201
ATOM 1740 NZ LYS B 201
ATOM 1741 N VAL B 202
ATOM 1742 CA VAL B 202
ATOM 1743 C VAL B 202
ATOM 1744 O VAL B 202
ATOM 1745 CB VAL B 202
ATOM 1746 CG1 VAL B 202

ATOM 1747 CG2 VAL B 202
ATOM 1748 N ALA B 203
ATOM 1749 CA ALA B 203
ATOM 1750 C ALA B 203
ATOM 1751 O ALA B 203
ATOM 1752 CB ALA B 203
ATOM 1753 N ALA B 204
ATOM 1754 CA ALA B 204
ATOM 1755 C ALA B 204
ATOM 1756 O ALA B 204
ATOM 1757 CB ALA B 204
ATOM 1758 N HIS B 205
ATOM 1759 CA HIS B 205
ATOM 1760 C HIS B 205
ATOM 1761 O HIS B 205
ATOM 1762 CB HIS B 205
ATOM 1763 CG HIS B 205
ATOM 1764 ND1 HIS B 205
ATOM 1765 CD2 HIS B 205
ATOM 1766 CE1 HIS B 205
ATOM 1767 NE2 HIS B 205
ATOM 1768 N GLY B 206
12.729 21.680 23.859 1.00 0.82
12.808 22.557 24.716 1.00 0.82
11.687 21.540 23.041 1.00 0.81
10.531 22.438 23.096 1.00 0.81
9.824 22.370 24.463 1.00 0.81
9.244 21.332 24.823 1.00 0.81
9.578 22.178 21.932 1.00 0.81
8.572 23.320 21.817 1.00 0.81
7.911 23.723 22.759 1.00 0.81
8.462 23.851 20.629 1.00 0.81
9.848 23.489 25.185 1.00 0.85
9.218 23.633 26.516 1.00 0.85
7.685 23.610 26.471 1.00 0.85
7.042 23.126 27.418 1.00 0.85
9.748 24.964 27.044 1.00 0.85
9.943 25.800 25.779 1.00 0.85
10.455 24.778 24.767 1.00 0.85
7.102 24.102 25.395 1.00 0.75

5.639	24.079	25.173	1.00	0.75
5.121	22.643	25.028	1.00	0.75
4.154	22.256	25.688	1.00	0.75
5.272	24.869	23.921	1.00	0.75
5.647	26.345	24.083	1.00	0.75
5.210	27.216	22.907	1.00	0.75
3.700	27.191	22.660	1.00	0.75
3.005	27.929	23.693	1.00	0.75
5.884	21.832	24.300	1.00	0.82
5.598	20.391	24.124	1.00	0.82
5.692	19.662	25.474	1.00	0.82
4.781	18.927	25.853	1.00	0.82
6.562	19.766	23.097	1.00	0.82
6.409	18.243	22.982	1.00	0.82
6.333	20.372	21.711	1.00	0.82
6.758	19.956	26.211	1.00	0.88
7.024	19.328	27.520	1.00	0.88
5.888	19.569	28.524	1.00	0.88
5.342	18.625	29.102	1.00	0.88
8.340	19.874	28.079	1.00	0.88
5.414	20.817	28.561	1.00	0.85
4.315	21.241	29.439	1.00	0.85
2.947	20.657	29.027	1.00	0.85
2.105	20.336	29.823	1.00	0.85
4.231	22.767	29.461	1.00	0.85
2.754	20.494	27.700	1.00	0.80
1.527	19.857	27.216	1.00	0.80
1.546	18.317	27.388	1.00	0.80
0.554	17.629	27.403	1.00	0.80
1.252	20.261	25.765	1.00	0.80
-0.157	19.841	25.342	1.00	0.80
-1.284	20.487	25.635	1.00	0.80
-0.469	18.793	24.586	1.00	0.80
-2.290	19.841	25.052	1.00	0.80
-1.785	18.796	24.404	1.00	0.80
2.785	17.812	27.571	1.00	0.92

ATOM 1769 CA GLY B 206
ATOM 1770 C GLY B 206
ATOM 1771 O GLY B 206
ATOM 1772 N VAL B 207

5425	ATOM	1773	CA	VAL B 207
5426	ATOM	1774	C	VAL B 207
5427	ATOM	1775	O	VAL B 207
5428	ATOM	1776	CB	VAL B 207
5429	ATOM	1777	CG1	VAL B 207
5430	ATOM	1778	CG2	VAL B 207
5431	ATOM	1779	N	VAL B 208
5432	ATOM	1780	CA	VAL B 208
5433	ATOM	1781	C	VAL B 208
5434	ATOM	1782	O	VAL B 208
5435	ATOM	1783	CB	VAL B 208
5436	ATOM	1784	CG1	VAL B 208
5437	ATOM	1785	CG2	VAL B 208
5438	ATOM	1786	N	ALA B 209
5439	ATOM	1787	CA	ALA B 209
5440	ATOM	1788	C	ALA B 209
5441	ATOM	1789	O	ALA B 209
5442	ATOM	1790	CB	ALA B 209
5443	ATOM	1791	N	LEU B 210
5444	ATOM	1792	CA	LEU B 210
5445	ATOM	1793	C	LEU B 210
5446	ATOM	1794	O	LEU B 210
5447	ATOM	1795	CB	LEU B 210
5448	ATOM	1796	CG	LEU B 210
5449	ATOM	1797	CD1	LEU B 210
5450	ATOM	1798	CD2	LEU B 210
5451	ATOM	1799	N	THR B 211
5452	ATOM	1800	CA	THR B 211
5453	ATOM	1801	C	THR B 211
5454	ATOM	1802	O	THR B 211
5455	ATOM	1803	CB	THR B 211
5456	ATOM	1804	OG1	THR B 211
5457	ATOM	1805	CG2	THR B 211
5458	ATOM	1806	N	GLY B 212
5459	ATOM	1807	CA	GLY B 212
5460	ATOM	1808	C	GLY B 212
5461	ATOM	1809	O	GLY B 212
5462	ATOM	1810	N	LEU B 213
5463	ATOM	1811	CA	LEU B 213
5464	ATOM	1812	C	LEU B 213

ATOM 1813 O LEU B 213
ATOM 1814 CB LEU B 213
ATOM 1815 CG LEU B 213
ATOM 1816 CD1 LEU B 213
ATOM 1817 CD2 LEU B 213
ATOM 1818 N ARG B 214
ATOM 1819 CA ARG B 214
ATOM 1820 C ARG B 214
ATOM 1821 O ARG B 214
ATOM 1822 CB ARG B 214
3.005 16.380 27.825 1.00 0.92
2.491 16.020 29.222 1.00 0.92
1.786 15.025 29.414 1.00 0.92
2.775 16.917 30.160 1.00 0.83
2.324 16.795 31.560 1.00 0.83
0.801 16.978 31.688 1.00 0.83
0.146 16.199 32.371 1.00 0.83
3.096 17.727 32.520 1.00 0.83
4.607 17.509 32.423 1.00 0.83
2.795 19.218 32.367 1.00 0.83
0.252 17.914 30.904 1.00 0.79
-1.204 18.179 30.909 1.00 0.79
-1.989 16.938 30.455 1.00 0.79
-2.842 16.421 31.180 1.00 0.79
-1.579 19.447 30.103 1.00 0.79
-1.416 19.335 28.599 1.00 0.79
-3.048 19.817 30.287 1.00 0.79
-1.581 16.389 29.313 1.00 0.83
-2.258 15.235 28.702 1.00 0.83
-2.163 13.994 29.595 1.00 0.83
-3.159 13.319 29.836 1.00 0.83
-1.638 14.942 27.336 1.00 0.83
-1.001 13.844 30.234 1.00 0.81
-0.749 12.701 31.122 1.00 0.81
-1.607 12.749 32.390 1.00 0.81
-2.237 11.755 32.765 1.00 0.81
0.730 12.681 31.490 1.00 0.81
1.096 11.353 32.144 1.00 0.81
1.076 10.188 31.151 1.00 0.81
2.466 11.525 32.763 1.00 0.81

5505 -1.735 13.947 32.950 1.00 0.78
5506 -2.607 14.188 34.117 1.00 0.78
5507 -4.092 13.992 33.765 1.00 0.78
5508 -4.920 13.690 34.606 1.00 0.78
5509 -2.423 15.591 34.702 1.00 0.78
5510 -2.864 16.588 33.778 1.00 0.78
5511 -0.992 15.835 35.192 1.00 0.78
5512 -4.385 14.116 32.456 1.00 0.82
5513 -5.733 13.916 31.894 1.00 0.82
5514 -6.295 12.511 32.159 1.00 0.82
5515 -7.500 12.304 32.077 1.00 0.82
5516 -5.403 11.559 32.439 1.00 0.83
5517 -5.781 10.168 32.753 1.00 0.83
5518 -6.161 9.978 34.222 1.00 0.83
5519 -6.808 8.987 34.571 1.00 0.83
5520 -4.632 9.219 32.402 1.00 0.83
5521 -4.146 9.378 30.955 1.00 0.83
5522 -2.985 8.419 30.699 1.00 0.83
5523 -5.264 9.150 29.934 1.00 0.83
5524 -5.739 10.925 35.056 1.00 0.73
5525 -5.964 10.893 36.513 1.00 0.73
5526 -7.440 10.751 36.865 1.00 0.73
5527 -7.786 9.877 37.659 1.00 0.73
5528 -5.496 12.165 37.206 1.00 0.73
5529 ATOM 1823 CG ARG B 214
5530 ATOM 1824 CD ARG B 214
5531 ATOM 1825 NE ARG B 214
5532 ATOM 1826 CZ ARG B 214
5533 ATOM 1827 NH1 ARG B 214
5534 ATOM 1828 NH2 ARG B 214
5535 ATOM 1829 N THR B 215
5536 ATOM 1830 CA THR B 215
5537 ATOM 1831 C THR B 215
5538 ATOM 1832 O THR B 215
5539 ATOM 1833 CB THR B 215
5540 ATOM 1834 OG1 THR B 215
5541 ATOM 1835 CG2 THR B 215
5542 ATOM 1836 N ALA B 216
5543 ATOM 1837 CA ALA B 216
5544 ATOM 1838 C ALA B 216

ATOM 1839 O ALA B 216
ATOM 1840 CB ALA B 216
ATOM 1841 N LEU B 217
ATOM 1842 CA LEU B 217
ATOM 1843 C LEU B 217
ATOM 1844 O LEU B 217
ATOM 1845 CB LEU B 217
ATOM 1846 CG LEU B 217
ATOM 1847 CD1 LEU B 217
ATOM 1848 CD2 LEU B 217
ATOM 1849 N ASP B 218
ATOM 1850 CA ASP B 218
ATOM 1851 C ASP B 218
ATOM 1852 O ASP B 218
ATOM 1853 CB ASP B 218
ATOM 1854 CG ASP B 218
ATOM 1855 OD1 ASP B 218
ATOM 1856 OD2 ASP B 218
ATOM 1857 N HIS B 219
ATOM 1858 CA HIS B 219
ATOM 1859 C HIS B 219
ATOM 1860 O HIS B 219
ATOM 1861 CB HIS B 219
ATOM 1862 CG HIS B 219
ATOM 1863 ND1 HIS B 219
ATOM 1864 CD2 HIS B 219
ATOM 1865 CE1 HIS B 219
ATOM 1866 NE2 HIS B 219
ATOM 1867 N MET B 220
ATOM 1868 CA MET B 220
ATOM 1869 C MET B 220
ATOM 1870 O MET B 220
ATOM 1871 CB MET B 220
ATOM 1872 CG MET B 220
ATOM 1873 SD MET B 220
ATOM 1874 CE MET B 220
ATOM 1875 N ASP B 221
ATOM 1876 CA ASP B 221
-3.980 12.318 37.148 1.00 0.73
-3.558 13.587 37.881 1.00 0.73

5585	-4.125	14.790	37.240	1.00	0.73
5586	-4.009	16.035	37.699	1.00	0.73
5587	-3.375	16.281	38.838	1.00	0.73
5588	-4.490	17.055	36.999	1.00	0.73
5589	-8.275	11.502	36.146	1.00	0.79
5590	-9.730	11.397	36.347	1.00	0.79
5591	-10.083	9.942	36.115	1.00	0.79
5592	-10.485	9.322	37.178	1.00	0.79
5593	-10.531	12.322	35.413	1.00	0.79
5594	-10.185	12.085	34.050	1.00	0.79
5595	-10.270	13.790	35.763	1.00	0.79
5596	-9.649	9.333	35.089	1.00	0.86
5597	-10.038	7.962	34.668	1.00	0.86
5598	-9.790	6.894	35.727	1.00	0.86
5599	-10.617	6.033	35.978	1.00	0.86
5600	-9.553	7.568	33.288	1.00	0.86
5601	-8.725	7.191	36.472	1.00	0.81
5602	-8.203	6.289	37.496	1.00	0.81
5603	-9.081	6.333	38.733	1.00	0.81
5604	-9.481	5.300	39.251	1.00	0.81
5605	-6.771	6.694	37.835	1.00	0.81
5606	-5.876	6.434	36.629	1.00	0.81
5607	-4.589	7.181	36.788	1.00	0.81
5608	-5.548	4.952	36.444	1.00	0.81
5609	-9.488	7.558	39.058	1.00	0.79
5610	-10.421	7.812	40.169	1.00	0.79
5611	-11.869	7.403	39.860	1.00	0.79
5612	-12.688	7.304	40.773	1.00	0.79
5613	-10.329	9.285	40.572	1.00	0.79
5614	-8.937	9.621	41.117	1.00	0.79
5615	-8.512	8.938	42.076	1.00	0.79
5616	-8.325	10.552	40.553	1.00	0.79
5617	-12.180	7.207	38.578	1.00	0.82
5618	-13.527	6.829	38.123	1.00	0.82
5619	-13.512	5.616	37.170	1.00	0.82
5620	-14.255	5.538	36.208	1.00	0.82
5621	-14.217	8.027	37.448	1.00	0.82
5622	-13.608	8.373	36.092	1.00	0.82
5623	-13.689	7.713	34.938	1.00	0.82
5624	-12.908	9.445	35.843	1.00	0.82

5625	-13.015	8.328	34.000	1.00	0.82	
5626	-12.507	9.407	34.575	1.00	0.82	
5627	-12.857	4.535	37.601	1.00	0.87	
5628	-12.696	3.386	36.687	1.00	0.87	
5629	-14.000	2.759	36.168	1.00	0.87	
5630	-14.034	2.201	35.078	1.00	0.87	
5631	-11.803	2.313	37.297	1.00	0.87	
5632	-10.349	2.773	37.251	1.00	0.87	
5633	-9.221	1.450	37.797	1.00	0.87	
5634	-9.312	0.331	36.417	1.00	0.87	
5635	-15.089	2.938	36.920	1.00	0.86	
5636	-16.405	2.417	36.510	1.00	0.86	
5637	ATOM	1877	C	ASP	B	221
5638	ATOM	1878	O	ASP	B	221
5639	ATOM	1879	CB	ASP	B	221
5640	ATOM	1880	CG	ASP	B	221
5641	ATOM	1881	OD1	ASP	B	221
5642	ATOM	1882	OD2	ASP	B	221
5643	ATOM	1883	N	GLU	B	222
5644	ATOM	1884	CA	GLU	B	222
5645	ATOM	1885	C	GLU	B	222
5646	ATOM	1886	O	GLU	B	222
5647	ATOM	1887	CB	GLU	B	222
5648	ATOM	1888	CG	GLU	B	222
5649	ATOM	1889	CD	GLU	B	222
5650	ATOM	1890	OE1	GLU	B	222
5651	ATOM	1891	OE2	GLU	B	222
5652	ATOM	1892	N	ILE	B	223
5653	ATOM	1893	CA	ILE	B	223
5654	ATOM	1894	C	ILE	B	223
5655	ATOM	1895	O	ILE	B	223
5656	ATOM	1896	CB	ILE	B	223
5657	ATOM	1897	CG1	ILE	B	223
5658	ATOM	1898	CG2	ILE	B	223
5659	ATOM	1899	CD1	ILE	B	223
5660	ATOM	1900	N	LYS	B	224
5661	ATOM	1901	CA	LYS	B	224
5662	ATOM	1902	C	LYS	B	224
5663	ATOM	1903	O	LYS	B	224
5664	ATOM	1904	CB	LYS	B	224

5665 ATOM 1905 CG LYS B 224
5666 ATOM 1906 CD LYS B 224
5667 ATOM 1907 CE LYS B 224
5668 ATOM 1908 NZ LYS B 224
5669 ATOM 1909 N SER B 225
5670 ATOM 1910 CA SER B 225
5671 ATOM 1911 C SER B 225
5672 ATOM 1912 O SER B 225
5673 ATOM 1913 CB SER B 225
5674 ATOM 1914 OG SER B 225
5675 ATOM 1915 N THR B 226
5676 ATOM 1916 CA THR B 226
5677 ATOM 1917 C THR B 226
5678 ATOM 1918 O THR B 226
5679 ATOM 1919 CB THR B 226
5680 ATOM 1920 OG1 THR B 226
5681 ATOM 1921 CG2 THR B 226
5682 ATOM 1922 N TYR B 227
5683 ATOM 1923 CA TYR B 227
5684 ATOM 1924 C TYR B 227
5685 ATOM 1925 O TYR B 227
5686 ATOM 1926 CB TYR B 227
5687 ATOM 1927 CG TYR B 227
5688 ATOM 1928 CD1 TYR B 227
5689 ATOM 1929 CD2 TYR B 227
5690 ATOM 1930 CE1 TYR B 227
5691 -17.228 3.362 35.615 1.00 0.86
5692 -18.244 2.950 35.061 1.00 0.86
5693 -17.211 1.976 37.735 1.00 0.86
5694 -16.471 0.888 38.515 1.00 0.86
5695 -16.474 -0.266 38.036 1.00 0.86
5696 -15.893 1.249 39.563 1.00 0.86
5697 -16.842 4.636 35.552 1.00 0.79
5698 -17.563 5.639 34.743 1.00 0.79
5699 -16.652 6.442 33.800 1.00 0.79
5700 -16.727 7.667 33.711 1.00 0.79
5701 -18.314 6.592 35.672 1.00 0.79
5702 -19.340 5.918 36.585 1.00 0.79
5703 -20.106 6.948 37.420 1.00 0.79
5704 -19.611 8.089 37.568 1.00 0.79

5705	-21.222	6.584	37.842	1.00	0.79
5706	-15.857	5.740	33.002	1.00	0.80
5707	-14.910	6.395	32.073	1.00	0.80
5708	-15.614	7.192	30.958	1.00	0.80
5709	-15.296	8.350	30.710	1.00	0.80
5710	-13.886	5.379	31.531	1.00	0.80
5711	-13.061	4.813	32.701	1.00	0.80
5712	-12.967	6.000	30.463	1.00	0.80
5713	-12.172	3.623	32.321	1.00	0.80
5714	-16.634	6.587	30.358	1.00	0.77
5715	-17.357	7.223	29.237	1.00	0.77
5716	-18.009	8.555	29.623	1.00	0.77
5717	-17.793	9.574	28.971	1.00	0.77
5718	-18.433	6.284	28.688	1.00	0.77
5719	-17.838	5.050	28.013	1.00	0.77
5720	-17.035	5.441	26.779	1.00	0.77
5721	-16.463	4.193	26.127	1.00	0.77
5722	-15.707	4.616	24.954	1.00	0.77
5723	-18.665	8.546	30.783	1.00	0.81
5724	-19.350	9.742	31.308	1.00	0.81
5725	-18.362	10.841	31.710	1.00	0.81
5726	-18.567	12.009	31.382	1.00	0.81
5727	-20.233	9.393	32.508	1.00	0.81
5728	-19.440	8.831	33.558	1.00	0.81
5729	-17.231	10.427	32.273	1.00	0.81
5730	-16.219	11.391	32.740	1.00	0.81
5731	-15.550	12.138	31.577	1.00	0.81
5732	-15.240	13.327	31.693	1.00	0.81
5733	-15.151	10.717	33.590	1.00	0.81
5734	-15.754	10.071	34.721	1.00	0.81
5735	-14.121	11.771	34.029	1.00	0.81
5736	-15.301	11.413	30.500	1.00	0.79
5737	-14.542	11.948	29.361	1.00	0.79
5738	-15.362	12.638	28.279	1.00	0.79
5739	-14.777	13.365	27.471	1.00	0.79
5740	-13.674	10.863	28.737	1.00	0.79
5741	-12.431	10.658	29.594	1.00	0.79
5742	-11.375	11.551	29.499	1.00	0.79
5743	-12.371	9.626	30.508	1.00	0.79
5744	-10.262	11.426	30.318	1.00	0.79

5745	ATOM	1931	CE2	TYR	B	227
5746	ATOM	1932	CZ	TYR	B	227
5747	ATOM	1933	OH	TYR	B	227
5748	ATOM	1934	N	ALA	B	228
5749	ATOM	1935	CA	ALA	B	228
5750	ATOM	1936	C	ALA	B	228
5751	ATOM	1937	O	ALA	B	228
5752	ATOM	1938	CB	ALA	B	228
5753	ATOM	1939	N	ALA	B	229
5754	ATOM	1940	CA	ALA	B	229
5755	ATOM	1941	C	ALA	B	229
5756	ATOM	1942	O	ALA	B	229
5757	ATOM	1943	CB	ALA	B	229
5758	ATOM	1944	N	LEU	B	230
5759	ATOM	1945	CA	LEU	B	230
5760	ATOM	1946	C	LEU	B	230
5761	ATOM	1947	O	LEU	B	230
5762	ATOM	1948	CB	LEU	B	230
5763	ATOM	1949	CG	LEU	B	230
5764	ATOM	1950	CD1	LEU	B	230
5765	ATOM	1951	CD2	LEU	B	230
5766	ATOM	1952	N	SER	B	231
5767	ATOM	1953	CA	SER	B	231
5768	ATOM	1954	C	SER	B	231
5769	ATOM	1955	O	SER	B	231
5770	ATOM	1956	CB	SER	B	231
5771	ATOM	1957	OG	SER	B	231
5772	ATOM	1958	N	VAL	B	232
5773	ATOM	1959	CA	VAL	B	232
5774	ATOM	1960	C	VAL	B	232
5775	ATOM	1961	O	VAL	B	232
5776	ATOM	1962	CB	VAL	B	232
5777	ATOM	1963	CG1	VAL	B	232
5778	ATOM	1964	CG2	VAL	B	232
5779	ATOM	1965	N	LEU	B	233
5780	ATOM	1966	CA	LEU	B	233
5781	ATOM	1967	C	LEU	B	233
5782	ATOM	1968	O	LEU	B	233
5783	ATOM	1969	CB	LEU	B	233
5784	ATOM	1970	CG	LEU	B	233

ATOM 1971 CD1 LEU B 233
ATOM 1972 CD2 LEU B 233
ATOM 1973 N HIS B 234
ATOM 1974 CA HIS B 234
ATOM 1975 C HIS B 234
ATOM 1976 O HIS B 234
ATOM 1977 CB HIS B 234
ATOM 1978 CG HIS B 234
ATOM 1979 ND1 HIS B 234
ATOM 1980 CD2 HIS B 234
ATOM 1981 CE1 HIS B 234
ATOM 1982 NE2 HIS B 234
ATOM 1983 N SER B 235
ATOM 1984 CA SER B 235
-11.268 9.500 31.313 1.00 0.79
-10.201 10.384 31.228 1.00 0.79
-9.071 10.197 31.953 1.00 0.79
-16.686 12.539 28.354 1.00 0.80
-17.593 13.124 27.343 1.00 0.80
-17.276 14.593 27.015 1.00 0.80
-17.094 14.949 25.859 1.00 0.80
-19.040 13.013 27.824 1.00 0.80
-16.974 15.380 28.062 1.00 0.78
-16.611 16.796 27.930 1.00 0.78
-15.250 17.028 27.222 1.00 0.78
-15.059 17.843 26.380 1.00 0.78
-16.567 17.447 29.312 1.00 0.78
-14.277 16.174 27.613 1.00 0.76
-12.959 16.235 26.969 1.00 0.76
-13.026 15.786 25.504 1.00 0.76
-12.362 16.347 24.631 1.00 0.76
-12.024 15.355 27.805 1.00 0.76
-10.556 15.763 27.669 1.00 0.76
-10.327 17.223 28.080 1.00 0.76
-9.714 14.852 28.561 1.00 0.76
-13.975 14.896 25.235 1.00 0.79
-14.275 14.386 23.887 1.00 0.79
-14.931 15.451 23.001 1.00 0.79
-14.533 15.609 21.838 1.00 0.79
-15.180 13.156 23.989 1.00 0.79

5825	-15.498	12.712	22.667	1.00	0.79
5826	-15.810	16.252	23.588	1.00	0.73
5827	-16.485	17.358	22.872	1.00	0.73
5828	-15.463	18.438	22.479	1.00	0.73
5829	-15.451	18.937	21.356	1.00	0.73
5830	-17.670	17.977	23.648	1.00	0.73
5831	-18.723	16.932	24.017	1.00	0.73
5832	-17.310	18.782	24.898	1.00	0.73
5833	-14.516	18.638	23.400	1.00	0.73
5834	-13.448	19.631	23.256	1.00	0.73
5835	-12.543	19.316	22.061	1.00	0.73
5836	-12.378	20.142	21.161	1.00	0.73
5837	-12.625	19.682	24.550	1.00	0.73
5838	-11.503	20.724	24.469	1.00	0.73
5839	-12.066	22.142	24.345	1.00	0.73
5840	-10.583	20.608	25.682	1.00	0.73
5841	-12.070	18.075	22.034	1.00	0.76
5842	-11.158	17.606	20.979	1.00	0.76
5843	-11.826	17.496	19.605	1.00	0.76
5844	-11.181	17.758	18.594	1.00	0.76
5845	-10.532	16.272	21.375	1.00	0.76
5846	-9.520	16.435	22.508	1.00	0.76
5847	-9.793	16.425	23.807	1.00	0.76
5848	-8.206	16.585	22.371	1.00	0.76
5849	-8.658	16.572	24.477	1.00	0.76
5850	-7.676	16.670	23.587	1.00	0.76
5851	-13.115	17.162	19.595	1.00	0.79
5852	-13.883	17.025	18.341	1.00	0.79
5853	ATOM	1985	C	SER B 235	
5854	ATOM	1986	O	SER B 235	
5855	ATOM	1987	CB	SER B 235	
5856	ATOM	1988	OG	SER B 235	
5857	ATOM	1989	N	GLU B 236	
5858	ATOM	1990	CA	GLU B 236	
5859	ATOM	1991	C	GLU B 236	
5860	ATOM	1992	O	GLU B 236	
5861	ATOM	1993	CB	GLU B 236	
5862	ATOM	1994	CG	GLU B 236	
5863	ATOM	1995	CD	GLU B 236	
5864	ATOM	1996	OE1	GLU B 236	

5865	ATOM	1997	OE2	GLU	B	236
5866	ATOM	1998	N	LYS	B	237
5867	ATOM	1999	CA	LYS	B	237
5868	ATOM	2000	C	LYS	B	237
5869	ATOM	2001	O	LYS	B	237
5870	ATOM	2002	CB	LYS	B	237
5871	ATOM	2003	CG	LYS	B	237
5872	ATOM	2004	CD	LYS	B	237
5873	ATOM	2005	CE	LYS	B	237
5874	ATOM	2006	NZ	LYS	B	237
5875	ATOM	2007	N	LEU	B	238
5876	ATOM	2008	CA	LEU	B	238
5877	ATOM	2009	C	LEU	B	238
5878	ATOM	2010	O	LEU	B	238
5879	ATOM	2011	CB	LEU	B	238
5880	ATOM	2012	CG	LEU	B	238
5881	ATOM	2013	CD1	LEU	B	238
5882	ATOM	2014	CD2	LEU	B	238
5883	ATOM	2015	N	HIS	B	239
5884	ATOM	2016	CA	HIS	B	239
5885	ATOM	2017	C	HIS	B	239
5886	ATOM	2018	O	HIS	B	239
5887	ATOM	2019	CB	HIS	B	239
5888	ATOM	2020	CG	HIS	B	239
5889	ATOM	2021	ND1	HIS	B	239
5890	ATOM	2022	CD2	HIS	B	239
5891	ATOM	2023	CE1	HIS	B	239
5892	ATOM	2024	NE2	HIS	B	239
5893	ATOM	2025	N	VAL	B	240
5894	ATOM	2026	CA	VAL	B	240
5895	ATOM	2027	C	VAL	B	240
5896	ATOM	2028	O	VAL	B	240
5897	ATOM	2029	CB	VAL	B	240
5898	ATOM	2030	CG1	VAL	B	240
5899	ATOM	2031	CG2	VAL	B	240
5900	ATOM	2032	N	ASP	B	241
5901	ATOM	2033	CA	ASP	B	241
5902	ATOM	2034	C	ASP	B	241
5903	ATOM	2035	O	ASP	B	241
5904	ATOM	2036	CB	ASP	B	241

ATOM 2037 CG ASP B 241
ATOM 2038 OD1 ASP B 241
-14.248 18.364 17.692 1.00 0.79
-13.972 18.584 16.512 1.00 0.79
-15.165 16.220 18.573 1.00 0.79
-15.886 16.122 17.342 1.00 0.79
-14.769 19.275 18.507 1.00 0.72
-15.407 20.487 17.972 1.00 0.72
-14.544 21.746 17.984 1.00 0.72
-14.654 22.551 17.069 1.00 0.72
-16.727 20.735 18.693 1.00 0.72
-17.774 19.700 18.267 1.00 0.72
-19.070 19.782 19.079 1.00 0.72
-19.318 20.831 19.716 1.00 0.72
-19.790 18.758 19.071 1.00 0.72
-13.696 21.891 18.998 1.00 0.72
-12.829 23.079 19.070 1.00 0.72
-11.418 22.776 18.557 1.00 0.72
-10.850 23.537 17.784 1.00 0.72
-12.769 23.619 20.502 1.00 0.72
-14.172 23.872 21.059 1.00 0.72
-14.099 24.544 22.425 1.00 0.72
-15.458 24.512 23.121 1.00 0.72
-15.366 25.194 24.417 1.00 0.72
-10.910 21.607 18.946 1.00 0.75
-9.541 21.214 18.583 1.00 0.75
-9.415 20.540 17.220 1.00 0.75
-8.370 20.645 16.586 1.00 0.75
-8.936 20.367 19.705 1.00 0.75
-9.023 21.055 21.074 1.00 0.75
-8.280 20.239 22.128 1.00 0.75
-8.543 22.510 21.045 1.00 0.75
-10.469 19.828 16.816 1.00 0.75
-10.528 19.141 15.505 1.00 0.75
-9.442 18.058 15.391 1.00 0.75
-8.917 17.758 14.314 1.00 0.75
-10.378 20.157 14.362 1.00 0.75
-11.465 21.223 14.426 1.00 0.75
-12.744 20.992 14.171 1.00 0.75
-11.307 22.517 14.687 1.00 0.75

-13.393	22.146	14.276	1.00	0.75	
-12.502	23.088	14.572	1.00	0.75	
-9.163	17.419	16.519	1.00	0.84	
-8.110	16.395	16.627	1.00	0.84	
-8.645	15.071	16.080	1.00	0.84	
-9.611	14.512	16.608	1.00	0.84	
-7.644	16.240	18.087	1.00	0.84	
-6.490	15.238	18.213	1.00	0.84	
-7.171	17.574	18.666	1.00	0.84	
-7.949	14.562	15.072	1.00	0.88	
-8.205	13.215	14.541	1.00	0.88	
-8.033	12.205	15.696	1.00	0.88	
-6.899	11.955	16.119	1.00	0.88	
-7.256	12.902	13.376	1.00	0.88	
-7.513	11.530	12.734	1.00	0.88	
-8.000	10.612	13.434	1.00	0.88	
ATOM	2039	OD2	ASP	B	241
ATOM	2040	N	PRO	B	242
ATOM	2041	CA	PRO	B	242
ATOM	2042	C	PRO	B	242
ATOM	2043	O	PRO	B	242
ATOM	2044	CB	PRO	B	242
ATOM	2045	CG	PRO	B	242
ATOM	2046	CD	PRO	B	242
ATOM	2047	N	ASP	B	243
ATOM	2048	CA	ASP	B	243
ATOM	2049	C	ASP	B	243
ATOM	2050	O	ASP	B	243
ATOM	2051	CB	ASP	B	243
ATOM	2052	CG	ASP	B	243
ATOM	2053	OD1	ASP	B	243
ATOM	2054	OD2	ASP	B	243
ATOM	2055	N	ASN	B	244
ATOM	2056	CA	ASN	B	244
ATOM	2057	C	ASN	B	244
ATOM	2058	O	ASN	B	244
ATOM	2059	CB	ASN	B	244
ATOM	2060	CG	ASN	B	244
ATOM	2061	OD1	ASN	B	244
ATOM	2062	ND2	ASN	B	244

ATOM 2063 N PHE B 245
ATOM 2064 CA PHE B 245
ATOM 2065 C PHE B 245
ATOM 2066 O PHE B 245
ATOM 2067 CB PHE B 245
ATOM 2068 CG PHE B 245
ATOM 2069 CD1 PHE B 245
ATOM 2070 CD2 PHE B 245
ATOM 2071 CE1 PHE B 245
ATOM 2072 CE2 PHE B 245
ATOM 2073 CZ PHE B 245
ATOM 2074 N ARG B 246
ATOM 2075 CA ARG B 246
ATOM 2076 C ARG B 246
ATOM 2077 O ARG B 246
ATOM 2078 CB ARG B 246
ATOM 2079 CG ARG B 246
ATOM 2080 CD ARG B 246
ATOM 2081 NE ARG B 246
ATOM 2082 CZ ARG B 246
ATOM 2083 NH1 ARG B 246
ATOM 2084 NH2 ARG B 246
ATOM 2085 N LEU B 247
ATOM 2086 CA LEU B 247
ATOM 2087 C LEU B 247
ATOM 2088 O LEU B 247
ATOM 2089 CB LEU B 247
ATOM 2090 CG LEU B 247
ATOM 2091 CD1 LEU B 247
ATOM 2092 CD2 LEU B 247
-7.150 11.373 11.556 1.00 0.88
-9.118 11.518 16.064 1.00 0.89
-9.132 10.544 17.175 1.00 0.89
-8.153 9.371 17.038 1.00 0.89
-7.761 8.783 18.043 1.00 0.89
-10.578 10.068 17.273 1.00 0.89
-11.091 10.232 15.848 1.00 0.89
-10.423 11.520 15.373 1.00 0.89
-7.680 9.082 15.823 1.00 0.91
-6.649 8.041 15.629 1.00 0.91

6025	-5.332	8.426	16.327	1.00	0.91
6026	-4.561	7.560	16.736	1.00	0.91
6027	-6.399	7.748	14.148	1.00	0.91
6028	-5.457	6.545	14.011	1.00	0.91
6029	-5.947	5.416	14.209	1.00	0.91
6030	-4.285	6.769	13.656	1.00	0.91
6031	-5.107	9.729	16.454	1.00	0.90
6032	-3.957	10.283	17.192	1.00	0.90
6033	-3.976	9.972	18.686	1.00	0.90
6034	-2.913	9.771	19.272	1.00	0.90
6035	-3.852	11.794	16.979	1.00	0.90
6036	-3.583	12.156	15.514	1.00	0.90
6037	-3.704	13.291	15.094	1.00	0.90
6038	-3.219	11.173	14.712	1.00	0.90
6039	-5.177	9.782	19.233	1.00	0.87
6040	-5.331	9.342	20.632	1.00	0.87
6041	-4.681	7.972	20.844	1.00	0.87
6042	-3.889	7.796	21.758	1.00	0.87
6043	-6.803	9.230	21.036	1.00	0.87
6044	-7.585	10.540	20.972	1.00	0.87
6045	-6.992	11.757	21.299	1.00	0.87
6046	-8.927	10.490	20.620	1.00	0.87
6047	-7.743	12.922	21.266	1.00	0.87
6048	-9.679	11.655	20.590	1.00	0.87
6049	-9.084	12.869	20.905	1.00	0.87
6050	-4.854	7.109	19.835	1.00	0.82
6051	-4.278	5.755	19.848	1.00	0.82
6052	-2.746	5.781	19.723	1.00	0.82
6053	-2.040	5.099	20.466	1.00	0.82
6054	-4.901	4.945	18.710	1.00	0.82
6055	-4.420	3.491	18.735	1.00	0.82
6056	-5.092	2.669	17.634	1.00	0.82
6057	-4.707	3.168	16.301	1.00	0.82
6058	-3.604	2.836	15.626	1.00	0.82
6059	-2.720	1.982	16.128	1.00	0.82
6060	-3.382	3.353	14.425	1.00	0.82
6061	-2.256	6.637	18.833	1.00	0.88
6062	-0.807	6.771	18.591	1.00	0.88
6063	-0.032	7.326	19.787	1.00	0.88
6064	1.049	6.827	20.110	1.00	0.88

6065	-0.545	7.609	17.339	1.00	0.88	
6066	-1.119	6.944	16.081	1.00	0.88	
6067	-0.823	7.820	14.867	1.00	0.88	
6068	-0.566	5.530	15.860	1.00	0.88	
6069	ATOM	2093	N	LEU	B	248
6070	ATOM	2094	CA	LEU	B	248
6071	ATOM	2095	C	LEU	B	248
6072	ATOM	2096	O	LEU	B	248
6073	ATOM	2097	CB	LEU	B	248
6074	ATOM	2098	CG	LEU	B	248
6075	ATOM	2099	CD1	LEU	B	248
6076	ATOM	2100	CD2	LEU	B	248
6077	ATOM	2101	N	CYS	B	249
6078	ATOM	2102	CA	CYS	B	249
6079	ATOM	2103	C	CYS	B	249
6080	ATOM	2104	O	CYS	B	249
6081	ATOM	2105	CB	CYS	B	249
6082	ATOM	2106	SG	CYS	B	249
6083	ATOM	2107	N	GLU	B	250
6084	ATOM	2108	CA	GLU	B	250
6085	ATOM	2109	C	GLU	B	250
6086	ATOM	2110	O	GLU	B	250
6087	ATOM	2111	CB	GLU	B	250
6088	ATOM	2112	CG	GLU	B	250
6089	ATOM	2113	CD	GLU	B	250
6090	ATOM	2114	OE1	GLU	B	250
6091	ATOM	2115	OE2	GLU	B	250
6092	ATOM	2116	N	CYS	B	251
6093	ATOM	2117	CA	CYS	B	251
6094	ATOM	2118	C	CYS	B	251
6095	ATOM	2119	O	CYS	B	251
6096	ATOM	2120	CB	CYS	B	251
6097	ATOM	2121	SG	CYS	B	251
6098	ATOM	2122	N	LEU	B	252
6099	ATOM	2123	CA	LEU	B	252
6100	ATOM	2124	C	LEU	B	252
6101	ATOM	2125	O	LEU	B	252
6102	ATOM	2126	CB	LEU	B	252
6103	ATOM	2127	CG	LEU	B	252
6104	ATOM	2128	CD1	LEU	B	252

ATOM	2129	CD2	LEU	B	252
ATOM	2130	N	THR	B	253
ATOM	2131	CA	THR	B	253
ATOM	2132	C	THR	B	253
ATOM	2133	O	THR	B	253
ATOM	2134	CB	THR	B	253
ATOM	2135	OG1	THR	B	253
ATOM	2136	CG2	THR	B	253
ATOM	2137	N	ILE	B	254
ATOM	2138	CA	ILE	B	254
ATOM	2139	C	ILE	B	254
ATOM	2140	O	ILE	B	254
ATOM	2141	CB	ILE	B	254
ATOM	2142	CG1	ILE	B	254
ATOM	2143	CG2	ILE	B	254
ATOM	2144	CD1	ILE	B	254
ATOM	2145	N	VAL	B	255
ATOM	2146	CA	VAL	B	255

-0.677	8.234	20.513	1.00	0.88
-0.090	8.782	21.747	1.00	0.88
-0.059	7.740	22.873	1.00	0.88
0.905	7.667	23.633	1.00	0.88
-0.827	10.044	22.207	1.00	0.88
-0.186	10.644	23.470	1.00	0.88
1.280	11.034	23.251	1.00	0.88
-0.994	11.847	23.950	1.00	0.88
-1.078	6.888	22.895	1.00	0.88
-1.167	5.797	23.881	1.00	0.88
-0.030	4.791	23.743	1.00	0.88
0.584	4.418	24.737	1.00	0.88
-2.468	5.031	23.728	1.00	0.88
-3.879	6.109	24.112	1.00	0.88
0.339	4.499	22.497	1.00	0.85
1.438	3.555	22.229	1.00	0.85
2.768	4.117	22.744	1.00	0.85
3.484	3.434	23.462	1.00	0.85
1.574	3.261	20.740	1.00	0.85
0.318	2.617	20.148	1.00	0.85
0.520	2.208	18.685	1.00	0.85
1.438	2.764	18.038	1.00	0.85

-0.231 1.309 18.254 1.00 0.85
2.961 5.420 22.525 1.00 0.90
4.144 6.148 23.020 1.00 0.90
4.178 6.249 24.550 1.00 0.90
5.232 6.044 25.154 1.00 0.90
4.201 7.544 22.401 1.00 0.90
4.384 7.496 20.583 1.00 0.90
3.002 6.381 25.162 1.00 0.89
2.877 6.373 26.633 1.00 0.89
3.304 5.018 27.201 1.00 0.89
4.142 4.964 28.105 1.00 0.89
1.437 6.660 27.079 1.00 0.89
0.976 8.094 26.799 1.00 0.89
-0.489 8.263 27.185 1.00 0.89
1.735 9.076 27.677 1.00 0.89
2.888 3.969 26.501 1.00 0.88
3.196 2.570 26.850 1.00 0.88
4.682 2.256 26.635 1.00 0.88
5.290 1.597 27.476 1.00 0.88
2.305 1.603 26.055 1.00 0.88
0.939 1.903 26.350 1.00 0.88
2.577 0.128 26.379 1.00 0.88
5.260 2.771 25.558 1.00 0.87
6.686 2.542 25.229 1.00 0.87
7.585 3.213 26.278 1.00 0.87
8.586 2.644 26.717 1.00 0.87
7.018 3.049 23.809 1.00 0.87
6.269 2.261 22.721 1.00 0.87
8.531 3.091 23.521 1.00 0.87
6.654 0.780 22.591 1.00 0.87
7.251 4.451 26.606 1.00 0.87
8.047 5.263 27.545 1.00 0.87
ATOM 2147 C VAL B 255
ATOM 2148 O VAL B 255
ATOM 2149 CB VAL B 255
ATOM 2150 CG1 VAL B 255
ATOM 2151 CG2 VAL B 255
ATOM 2152 N VAL B 256
ATOM 2153 CA VAL B 256
ATOM 2154 C VAL B 256

6185	ATOM	2155	O	VAL	B	256
6186	ATOM	2156	CB	VAL	B	256
6187	ATOM	2157	CG1	VAL	B	256
6188	ATOM	2158	CG2	VAL	B	256
6189	ATOM	2159	N	ALA	B	257
6190	ATOM	2160	CA	ALA	B	257
6191	ATOM	2161	C	ALA	B	257
6192	ATOM	2162	O	ALA	B	257
6193	ATOM	2163	CB	ALA	B	257
6194	ATOM	2164	N	GLY	B	258
6195	ATOM	2165	CA	GLY	B	258
6196	ATOM	2166	C	GLY	B	258
6197	ATOM	2167	O	GLY	B	258
6198	ATOM	2168	N	LYS	B	259
6199	ATOM	2169	CA	LYS	B	259
6200	ATOM	2170	C	LYS	B	259
6201	ATOM	2171	O	LYS	B	259
6202	ATOM	2172	CB	LYS	B	259
6203	ATOM	2173	CG	LYS	B	259
6204	ATOM	2174	CD	LYS	B	259
6205	ATOM	2175	CE	LYS	B	259
6206	ATOM	2176	NZ	LYS	B	259
6207	ATOM	2177	N	MET	B	260
6208	ATOM	2178	CA	MET	B	260
6209	ATOM	2179	C	MET	B	260
6210	ATOM	2180	O	MET	B	260
6211	ATOM	2181	CB	MET	B	260
6212	ATOM	2182	CG	MET	B	260
6213	ATOM	2183	SD	MET	B	260
6214	ATOM	2184	CE	MET	B	260
6215	ATOM	2185	N	GLY	B	261
6216	ATOM	2186	CA	GLY	B	261
6217	ATOM	2187	C	GLY	B	261
6218	ATOM	2188	O	GLY	B	261
6219	ATOM	2189	N	LYS	B	262
6220	ATOM	2190	CA	LYS	B	262
6221	ATOM	2191	C	LYS	B	262
6222	ATOM	2192	O	LYS	B	262
6223	ATOM	2193	CB	LYS	B	262
6224	ATOM	2194	CG	LYS	B	262

ATOM 2195 CD LYS B 262
ATOM 2196 CE LYS B 262
ATOM 2197 NZ LYS B 262
ATOM 2198 N LYS B 263
ATOM 2199 CA LYS B 263
ATOM 2200 C LYS B 263
7.963 4.711 28.974 1.00 0.87
8.998 4.514 29.613 1.00 0.87
7.649 6.745 27.441 1.00 0.87
8.175 7.596 28.604 1.00 0.87
8.212 7.326 26.141 1.00 0.87
6.744 4.476 29.455 1.00 0.86
6.545 3.855 30.785 1.00 0.86
7.288 2.510 30.886 1.00 0.86
7.806 2.134 31.912 1.00 0.86
5.047 3.704 31.122 1.00 0.86
4.304 2.713 30.221 1.00 0.86
4.852 3.300 32.585 1.00 0.86
7.371 1.847 29.703 1.00 0.86
8.074 0.589 29.505 1.00 0.86
9.563 0.646 29.811 1.00 0.86
10.112 -0.146 30.561 1.00 0.86
7.898 0.131 28.047 1.00 0.86
10.117 1.716 29.228 1.00 0.87
11.525 2.082 29.387 1.00 0.87
11.830 2.514 30.830 1.00 0.87
12.957 2.389 31.282 1.00 0.87
10.796 3.011 31.517 1.00 0.78
10.921 3.542 32.888 1.00 0.78
10.869 2.417 33.930 1.00 0.78
11.586 2.433 34.925 1.00 0.78
9.776 4.530 33.142 1.00 0.78
9.885 5.241 34.495 1.00 0.78
10.688 6.537 34.399 1.00 0.78
10.766 7.196 35.777 1.00 0.78
11.212 8.589 35.665 1.00 0.78
9.987 1.456 33.660 1.00 0.77
9.697 0.351 34.562 1.00 0.77
10.625 -0.865 34.365 1.00 0.77
10.946 -1.627 35.258 1.00 0.77

8.204 0.012 34.439 1.00 0.77
7.318 1.087 35.089 1.00 0.77
5.555 0.623 35.259 1.00 0.77
5.622 -0.862 36.239 1.00 0.77
11.058 -1.000 33.101 1.00 0.84
11.928 -2.107 32.674 1.00 0.84
11.277 -3.465 32.960 1.00 0.84
10.132 -3.716 32.586 1.00 0.84
11.940 -4.221 33.831 1.00 0.76
11.518 -5.587 34.200 1.00 0.76
10.141 -5.658 34.881 1.00 0.76
9.384 -6.609 34.669 1.00 0.76
12.582 -6.217 35.108 1.00 0.76
12.300 -7.688 35.437 1.00 0.76
12.293 -8.561 34.177 1.00 0.76
11.928 -10.012 34.495 1.00 0.76
10.545 -10.126 34.981 1.00 0.76
9.854 -4.670 35.725 1.00 0.77
8.571 -4.622 36.460 1.00 0.77
7.367 -4.413 35.534 1.00 0.77
ATOM 2201 O LYS B 263
ATOM 2202 CB LYS B 263
ATOM 2203 CG LYS B 263
ATOM 2204 CD LYS B 263
ATOM 2205 CE LYS B 263
ATOM 2206 NZ LYS B 263
ATOM 2207 N LEU B 264
ATOM 2208 CA LEU B 264
ATOM 2209 C LEU B 264
ATOM 2210 O LEU B 264
ATOM 2211 CB LEU B 264
ATOM 2212 CG LEU B 264
ATOM 2213 CD1 LEU B 264
ATOM 2214 CD2 LEU B 264
ATOM 2215 N SER B 265
ATOM 2216 CA SER B 265
ATOM 2217 C SER B 265
ATOM 2218 O SER B 265
ATOM 2219 CB SER B 265
ATOM 2220 OG SER B 265

ATOM 2221 N PRO B 266
ATOM 2222 CA PRO B 266
ATOM 2223 C PRO B 266
ATOM 2224 O PRO B 266
ATOM 2225 CB PRO B 266
ATOM 2226 CG PRO B 266
ATOM 2227 CD PRO B 266
ATOM 2228 N GLU B 267
ATOM 2229 CA GLU B 267
ATOM 2230 C GLU B 267
ATOM 2231 O GLU B 267
ATOM 2232 CB GLU B 267
ATOM 2233 CG GLU B 267
ATOM 2234 CD GLU B 267
ATOM 2235 OE1 GLU B 267
ATOM 2236 OE2 GLU B 267
ATOM 2237 N MET B 268
ATOM 2238 CA MET B 268
ATOM 2239 C MET B 268
ATOM 2240 O MET B 268
ATOM 2241 CB MET B 268
ATOM 2242 CG MET B 268
ATOM 2243 SD MET B 268
ATOM 2244 CE MET B 268
ATOM 2245 N GLN B 269
ATOM 2246 CA GLN B 269
ATOM 2247 C GLN B 269
ATOM 2248 O GLN B 269
ATOM 2249 CB GLN B 269
ATOM 2250 CG GLN B 269
ATOM 2251 CD GLN B 269
ATOM 2252 OE1 GLN B 269
ATOM 2253 NE2 GLN B 269
ATOM 2254 N ALA B 270
6.222 -4.683 35.905 1.00 0.77
8.591 -3.588 37.591 1.00 0.77
8.841 -2.167 37.101 1.00 0.77
8.888 -1.131 38.222 1.00 0.77
9.343 0.210 37.655 1.00 0.77
9.174 1.379 38.509 1.00 0.77

6345	7.657	-3.935	34.329	1.00	0.87
6346	6.643	-3.793	33.291	1.00	0.87
6347	6.381	-5.146	32.615	1.00	0.87
6348	6.558	-5.362	31.428	1.00	0.87
6349	7.114	-2.749	32.293	1.00	0.87
6350	5.948	-2.564	31.336	1.00	0.87
6351	4.807	-1.715	31.886	1.00	0.87
6352	6.472	-1.991	30.065	1.00	0.87
6353	5.876	-6.050	33.442	1.00	0.94
6354	5.579	-7.424	33.039	1.00	0.94
6355	4.490	-7.484	31.950	1.00	0.94
6356	3.736	-6.485	31.783	1.00	0.94
6357	5.186	-8.172	34.323	1.00	0.94
6358	3.885	-7.781	34.761	1.00	0.94
6359	4.291	-8.619	31.308	1.00	0.99
6360	3.198	-8.835	30.331	1.00	0.99
6361	1.820	-8.596	30.978	1.00	0.99
6362	0.924	-8.023	30.372	1.00	0.99
6363	3.353	-10.286	29.882	1.00	0.99
6364	3.967	-10.976	31.100	1.00	0.99
6365	4.965	-9.927	31.572	1.00	0.99
6366	1.731	-8.934	32.271	1.00	0.87
6367	0.506	-8.748	33.068	1.00	0.87
6368	0.209	-7.256	33.292	1.00	0.87
6369	-0.933	-6.822	33.136	1.00	0.87
6370	0.656	-9.475	34.408	1.00	0.87
6371	-0.708	-9.632	35.087	1.00	0.87
6372	-0.589	-10.379	36.415	1.00	0.87
6373	-0.447	-11.619	36.357	1.00	0.87
6374	-0.544	-9.695	37.454	1.00	0.87
6375	1.262	-6.487	33.563	1.00	0.88
6376	1.162	-5.025	33.740	1.00	0.88
6377	0.792	-4.318	32.434	1.00	0.88
6378	-0.126	-3.498	32.408	1.00	0.88
6379	2.491	-4.451	34.232	1.00	0.88
6380	2.851	-4.925	35.639	1.00	0.88
6381	1.657	-4.397	36.913	1.00	0.88
6382	1.922	-2.646	36.906	1.00	0.88
6383	1.404	-4.777	31.344	1.00	0.88
6384	1.167	-4.214	30.004	1.00	0.88

-0.298	-4.395	29.581	1.00	0.88			
-0.941	-3.441	29.149	1.00	0.88			
2.078	-4.900	28.990	1.00	0.88			
1.949	-4.250	27.608	1.00	0.88			
2.740	-4.989	26.530	1.00	0.88			
3.509	-5.909	26.769	1.00	0.88			
2.513	-4.601	25.298	1.00	0.88			
-0.826	-5.590	29.844	1.00	0.92			
ATOM	2255	CA	ALA	B	270		
ATOM	2256	C	ALA	B	270		
ATOM	2257	O	ALA	B	270		
ATOM	2258	CB	ALA	B	270		
ATOM	2259	N	ALA	B	271		
ATOM	2260	CA	ALA	B	271		
ATOM	2261	C	ALA	B	271		
ATOM	2262	O	ALA	B	271		
ATOM	2263	CB	ALA	B	271		
ATOM	2264	N	TRP	B	272		
ATOM	2265	CA	TRP	B	272		
ATOM	2266	C	TRP	B	272		
ATOM	2267	O	TRP	B	272		
ATOM	2268	CB	TRP	B	272		
ATOM	2269	CG	TRP	B	272		
ATOM	2270	CD1	TRP	B	272		
ATOM	2271	CD2	TRP	B	272		
ATOM	2272	NE1	TRP	B	272		
ATOM	2273	CE2	TRP	B	272		
ATOM	2274	CE3	TRP	B	272		
ATOM	2275	CZ2	TRP	B	272		
ATOM	2276	CZ3	TRP	B	272		
ATOM	2277	CH2	TRP	B	272		
ATOM	2278	N	GLN	B	273		
ATOM	2279	CA	GLN	B	273		
ATOM	2280	C	GLN	B	273		
ATOM	2281	O	GLN	B	273		
ATOM	2282	CB	GLN	B	273		
ATOM	2283	CG	GLN	B	273		
ATOM	2284	CD	GLN	B	273		
ATOM	2285	OE1	GLN	B	273		
ATOM	2286	NE2	GLN	B	273		

ATOM	2287	N	LYS	B	274
ATOM	2288	CA	LYS	B	274
ATOM	2289	C	LYS	B	274
ATOM	2290	O	LYS	B	274
ATOM	2291	CB	LYS	B	274
ATOM	2292	CG	LYS	B	274
ATOM	2293	CD	LYS	B	274
ATOM	2294	CE	LYS	B	274
ATOM	2295	NZ	LYS	B	274
ATOM	2296	N	TYR	B	275
ATOM	2297	CA	TYR	B	275
ATOM	2298	C	TYR	B	275
ATOM	2299	O	TYR	B	275
ATOM	2300	CB	TYR	B	275
ATOM	2301	CG	TYR	B	275
ATOM	2302	CD1	TYR	B	275
ATOM	2303	CD2	TYR	B	275
ATOM	2304	CE1	TYR	B	275
ATOM	2305	CE2	TYR	B	275
ATOM	2306	CZ	TYR	B	275
ATOM	2307	OH	TYR	B	275
ATOM	2308	N	LEU	B	276

-2.213	-5.946	29.496	1.00	0.92
-3.224	-5.028	30.200	1.00	0.92
-4.018	-4.358	29.542	1.00	0.92
-2.477	-7.405	29.872	1.00	0.92
-3.034	-4.870	31.511	1.00	0.92
-3.891	-4.006	32.346	1.00	0.92
-3.756	-2.515	31.998	1.00	0.92
-4.762	-1.801	31.947	1.00	0.92
-3.567	-4.231	33.824	1.00	0.92
-2.543	-2.093	31.649	1.00	0.93
-2.273	-0.687	31.290	1.00	0.93
-2.836	-0.332	29.907	1.00	0.93
-3.501	0.696	29.745	1.00	0.93
-0.774	-0.381	31.368	1.00	0.93
-0.492	1.091	31.053	1.00	0.93
-0.027	1.567	29.901	1.00	0.93
-0.634	2.169	31.919	1.00	0.93
0.141	2.886	30.001	1.00	0.93

6465	-0.220	3.288	31.214	1.00	0.93
6466	-1.105	2.293	33.215	1.00	0.93
6467	-0.270	4.546	31.810	1.00	0.93
6468	-1.171	3.555	33.798	1.00	0.93
6469	-0.750	4.678	33.102	1.00	0.93
6470	-2.691	-1.250	28.961	1.00	0.88
6471	-3.249	-1.069	27.609	1.00	0.88
6472	-4.776	-1.124	27.605	1.00	0.88
6473	-5.415	-0.274	26.984	1.00	0.88
6474	-2.690	-2.097	26.638	1.00	0.88
6475	-1.349	-1.624	26.067	1.00	0.88
6476	-0.721	-2.610	25.076	1.00	0.88
6477	0.472	-2.579	24.810	1.00	0.88
6478	-1.524	-3.457	24.465	1.00	0.88
6479	-5.333	-2.001	28.436	1.00	0.88
6480	-6.791	-2.101	28.623	1.00	0.88
6481	-7.368	-0.771	29.151	1.00	0.88
6482	-8.322	-0.214	28.636	1.00	0.88
6483	-7.098	-3.235	29.600	1.00	0.88
6484	-8.600	-3.378	29.833	1.00	0.88
6485	-8.863	-4.576	30.730	1.00	0.88
6486	-10.360	-4.731	30.935	1.00	0.88
6487	-10.612	-6.003	31.616	1.00	0.88
6488	-6.669	-0.206	30.133	1.00	0.94
6489	-7.086	1.082	30.704	1.00	0.94
6490	-7.006	2.223	29.679	1.00	0.94
6491	-7.961	2.985	29.504	1.00	0.94
6492	-6.224	1.357	31.934	1.00	0.94
6493	-6.640	2.660	32.590	1.00	0.94
6494	-7.731	2.678	33.463	1.00	0.94
6495	-5.931	3.819	32.284	1.00	0.94
6496	-8.105	3.867	34.036	1.00	0.94
6497	-6.301	5.007	32.848	1.00	0.94
6498	-7.376	5.002	33.708	1.00	0.94
6499	-7.511	6.099	34.422	1.00	0.94
6500	-5.933	2.208	28.892	1.00	0.90
6501	ATOM	2309	CA LEU B 276		
6502	ATOM	2310	C LEU B 276		
6503	ATOM	2311	O LEU B 276		
6504	ATOM	2312	CB LEU B 276		

6505	ATOM	2313	CG	LEU	B	276
6506	ATOM	2314	CD1	LEU	B	276
6507	ATOM	2315	CD2	LEU	B	276
6508	ATOM	2316	N	CYS	B	277
6509	ATOM	2317	CA	CYS	B	277
6510	ATOM	2318	C	CYS	B	277
6511	ATOM	2319	O	CYS	B	277
6512	ATOM	2320	CB	CYS	B	277
6513	ATOM	2321	SG	CYS	B	277
6514	ATOM	2322	N	ALA	B	278
6515	ATOM	2323	CA	ALA	B	278
6516	ATOM	2324	C	ALA	B	278
6517	ATOM	2325	O	ALA	B	278
6518	ATOM	2326	CB	ALA	B	278
6519	ATOM	2327	N	VAL	B	279
6520	ATOM	2328	CA	VAL	B	279
6521	ATOM	2329	C	VAL	B	279
6522	ATOM	2330	O	VAL	B	279
6523	ATOM	2331	CB	VAL	B	279
6524	ATOM	2332	CG1	VAL	B	279
6525	ATOM	2333	CG2	VAL	B	279
6526	ATOM	2334	N	VAL	B	280
6527	ATOM	2335	CA	VAL	B	280
6528	ATOM	2336	C	VAL	B	280
6529	ATOM	2337	O	VAL	B	280
6530	ATOM	2338	CB	VAL	B	280
6531	ATOM	2339	CG1	VAL	B	280
6532	ATOM	2340	CG2	VAL	B	280
6533	ATOM	2341	N	SER	B	281
6534	ATOM	2342	CA	SER	B	281
6535	ATOM	2343	C	SER	B	281
6536	ATOM	2344	O	SER	B	281
6537	ATOM	2345	CB	SER	B	281
6538	ATOM	2346	OG	SER	B	281
6539	ATOM	2347	N	ALA	B	282
6540	ATOM	2348	CA	ALA	B	282
6541	ATOM	2349	C	ALA	B	282
6542	ATOM	2350	O	ALA	B	282
6543	ATOM	2351	CB	ALA	B	282
6544	ATOM	2352	N	LEU	B	283

ATOM 2353 CA LEU B 283
ATOM 2354 C LEU B 283
ATOM 2355 O LEU B 283
ATOM 2356 CB LEU B 283
ATOM 2357 CG LEU B 283
ATOM 2358 CD1 LEU B 283
ATOM 2359 CD2 LEU B 283
ATOM 2360 N GLY B 284
ATOM 2361 CA GLY B 284
ATOM 2362 C GLY B 284
-5.707 3.212 27.838 1.00 0.90
-6.703 3.122 26.685 1.00 0.90
-7.179 4.146 26.196 1.00 0.90
-4.293 3.111 27.268 1.00 0.90
-3.238 3.747 28.176 1.00 0.90
-1.865 3.519 27.548 1.00 0.90
-3.474 5.252 28.343 1.00 0.90
-7.100 1.900 26.353 1.00 0.90
-8.032 1.651 25.239 1.00 0.90
-9.454 2.106 25.597 1.00 0.90
-10.168 2.640 24.753 1.00 0.90
-8.010 0.177 24.822 1.00 0.90
-8.770 -0.967 26.031 1.00 0.90
-9.786 1.992 26.885 1.00 0.90
-11.079 2.445 27.433 1.00 0.90
-11.182 3.977 27.446 1.00 0.90
-12.220 4.542 27.094 1.00 0.90
-11.248 1.897 28.851 1.00 0.90
-10.052 4.626 27.719 1.00 0.89
-9.952 6.102 27.738 1.00 0.89
-10.006 6.663 26.309 1.00 0.89
-10.698 7.650 26.046 1.00 0.89
-8.676 6.543 28.478 1.00 0.89
-8.410 8.049 28.356 1.00 0.89
-8.793 6.205 29.966 1.00 0.89
-9.307 5.997 25.402 1.00 0.87
-9.283 6.382 23.972 1.00 0.87
-10.676 6.227 23.370 1.00 0.87
-11.123 7.067 22.586 1.00 0.87
-8.296 5.509 23.192 1.00 0.87

-8.294 5.752 21.678 1.00 0.87
-6.913 5.871 23.676 1.00 0.87
-11.324 5.147 23.779 1.00 0.86
-12.678 4.831 23.326 1.00 0.86
-13.617 5.966 23.761 1.00 0.86
-14.321 6.524 22.939 1.00 0.86
-13.070 3.474 23.910 1.00 0.86
-14.284 3.022 23.315 1.00 0.86
-13.451 6.410 25.021 1.00 0.86
-14.242 7.503 25.616 1.00 0.86
-13.971 8.873 24.981 1.00 0.86
-14.893 9.656 24.767 1.00 0.86
-13.970 7.569 27.120 1.00 0.86
-12.713 9.103 24.604 1.00 0.81
-12.309 10.349 23.933 1.00 0.81
-12.901 10.547 22.537 1.00 0.81
-13.111 11.674 22.109 1.00 0.81
-10.786 10.486 23.852 1.00 0.81
-10.156 10.940 25.171 1.00 0.81
-8.649 11.103 24.977 1.00 0.81
-10.761 12.259 25.669 1.00 0.81
-13.154 9.415 21.862 1.00 0.86
-13.678 9.417 20.484 1.00 0.86
-15.210 9.331 20.413 1.00 0.86
ATOM 2363 O GLY B 284
ATOM 2364 N ARG B 285
ATOM 2365 CA ARG B 285
ATOM 2366 C ARG B 285
ATOM 2367 O ARG B 285
ATOM 2368 CB ARG B 285
ATOM 2369 CG ARG B 285
ATOM 2370 CD ARG B 285
ATOM 2371 NE ARG B 285
ATOM 2372 CZ ARG B 285
ATOM 2373 NH1 ARG B 285
ATOM 2374 NH2 ARG B 285
ATOM 2375 N GLN B 286
ATOM 2376 CA GLN B 286
ATOM 2377 C GLN B 286
ATOM 2378 O GLN B 286

ATOM 2379 CB GLN B 286
ATOM 2380 CG GLN B 286
ATOM 2381 CD GLN B 286
ATOM 2382 OE1 GLN B 286
ATOM 2383 NE2 GLN B 286
ATOM 2384 N TYR B 287
ATOM 2385 CA TYR B 287
ATOM 2386 C TYR B 287
ATOM 2387 O TYR B 287
ATOM 2388 CB TYR B 287
ATOM 2389 CG TYR B 287
ATOM 2390 CD1 TYR B 287
ATOM 2391 CD2 TYR B 287
ATOM 2392 CE1 TYR B 287
ATOM 2393 CE2 TYR B 287
ATOM 2394 CZ TYR B 287
ATOM 2395 OH TYR B 287
ATOM 2396 N HIS B 288
ATOM 2397 CA HIS B 288
ATOM 2398 C HIS B 288
ATOM 2399 O HIS B 288
ATOM 2400 CB HIS B 288
ATOM 2401 CG HIS B 288
ATOM 2402 ND1 HIS B 288
ATOM 2403 CD2 HIS B 288
ATOM 2404 CE1 HIS B 288
ATOM 2405 NE2 HIS B 288
ATOM 2406 N SER C 289
ATOM 2407 CA SER C 289
ATOM 2408 C SER C 289
ATOM 2409 O SER C 289
ATOM 2410 CB SER C 289
ATOM 2411 OG SER C 289
ATOM 2412 N LEU C 290
ATOM 2413 CA LEU C 290
ATOM 2414 C LEU C 290
ATOM 2415 O LEU C 290
ATOM 2416 CB LEU C 290
-15.789 9.522 19.341 1.00 0.86
-15.850 9.155 21.565 1.00 0.72

6665	-17.303	8.918	21.651	1.00	0.72
6666	-18.212	10.084	21.249	1.00	0.72
6667	-19.316	9.845	20.759	1.00	0.72
6668	-17.701	8.416	23.041	1.00	0.72
6669	-17.189	7.002	23.325	1.00	0.72
6670	-17.683	5.965	22.314	1.00	0.72
6671	-17.133	4.625	22.599	1.00	0.72
6672	-17.438	3.504	21.950	1.00	0.72
6673	-18.298	3.511	20.945	1.00	0.72
6674	-16.898	2.350	22.316	1.00	0.72
6675	-17.737	11.315	21.399	1.00	0.71
6676	-18.559	12.503	21.067	1.00	0.71
6677	-18.212	13.106	19.696	1.00	0.71
6678	-18.412	14.307	19.447	1.00	0.71
6679	-18.441	13.567	22.167	1.00	0.71
6680	-18.746	13.056	23.584	1.00	0.71
6681	-20.133	12.436	23.769	1.00	0.71
6682	-20.330	11.540	24.574	1.00	0.71
6683	-21.119	12.947	23.059	1.00	0.71
6684	-17.760	12.262	18.783	1.00	0.68
6685	-17.441	12.663	17.399	1.00	0.68
6686	-18.599	12.333	16.453	1.00	0.68
6687	-18.732	11.210	15.962	1.00	0.68
6688	-16.162	11.977	16.913	1.00	0.68
6689	-14.888	12.655	17.413	1.00	0.68
6690	-14.412	12.403	18.692	1.00	0.68
6691	-14.160	13.464	16.551	1.00	0.68
6692	-13.218	12.964	19.113	1.00	0.68
6693	-12.961	14.024	16.970	1.00	0.68
6694	-12.496	13.778	18.256	1.00	0.68
6695	-11.383	14.399	18.717	1.00	0.68
6696	-19.447	13.328	16.230	1.00	0.64
6697	-20.571	13.192	15.278	1.00	0.64
6698	-20.155	13.604	13.857	1.00	0.64
6699	-21.047	13.547	12.986	1.00	0.64
6700	-21.774	14.042	15.699	1.00	0.64
6701	-22.115	13.929	17.184	1.00	0.64
6702	-21.921	14.899	18.070	1.00	0.64
6703	-22.619	12.874	17.819	1.00	0.64
6704	-22.312	14.449	19.258	1.00	0.64

-22.742 13.200 19.104 1.00 0.64
3.068 -6.025 -3.623 1.00 0.91
3.396 -5.292 -4.863 1.00 0.91
2.515 -5.768 -6.031 1.00 0.91
2.028 -6.908 -6.060 1.00 0.91
4.869 -5.494 -5.254 1.00 0.91
5.073 -6.833 -5.719 1.00 0.91
2.250 -4.849 -6.939 1.00 0.93
1.414 -5.118 -8.119 1.00 0.93
2.146 -6.007 -9.117 1.00 0.93
3.259 -5.701 -9.555 1.00 0.93
1.013 -3.796 -8.776 1.00 0.93
ATOM 2417 CG LEU C 290
ATOM 2418 CD1 LEU C 290
ATOM 2419 CD2 LEU C 290
ATOM 2420 N THR C 291
ATOM 2421 CA THR C 291
ATOM 2422 C THR C 291
ATOM 2423 O THR C 291
ATOM 2424 CB THR C 291
ATOM 2425 OG1 THR C 291
ATOM 2426 CG2 THR C 291
ATOM 2427 N PRO C 292
ATOM 2428 CA PRO C 292
ATOM 2429 C PRO C 292
ATOM 2430 O PRO C 292
ATOM 2431 CB PRO C 292
ATOM 2432 CG PRO C 292
ATOM 2433 CD PRO C 292
ATOM 2434 N LYS C 293
ATOM 2435 CA LYS C 293
ATOM 2436 C LYS C 293
ATOM 2437 O LYS C 293
ATOM 2438 CB LYS C 293
ATOM 2439 CG LYS C 293
ATOM 2440 CD LYS C 293
ATOM 2441 CE LYS C 293
ATOM 2442 NZ LYS C 293
ATOM 2443 N ASP C 294
ATOM 2444 CA ASP C 294

ATOM 2445 C ASP C 294
ATOM 2446 O ASP C 294
ATOM 2447 CB ASP C 294
ATOM 2448 CG ASP C 294
ATOM 2449 OD1 ASP C 294
ATOM 2450 OD2 ASP C 294
ATOM 2451 N LYS C 295
ATOM 2452 CA LYS C 295
ATOM 2453 C LYS C 295
ATOM 2454 O LYS C 295
ATOM 2455 CB LYS C 295
ATOM 2456 CG LYS C 295
ATOM 2457 CD LYS C 295
ATOM 2458 CE LYS C 295
ATOM 2459 NZ LYS C 295
ATOM 2460 N ALA C 296
ATOM 2461 CA ALA C 296
ATOM 2462 C ALA C 296
ATOM 2463 O ALA C 296
ATOM 2464 CB ALA C 296
ATOM 2465 N THR C 297
ATOM 2466 CA THR C 297
ATOM 2467 C THR C 297
ATOM 2468 O THR C 297
ATOM 2469 CB THR C 297
ATOM 2470 OG1 THR C 297
0.325 -2.848 -7.786 1.00 0.93
0.058 -1.503 -8.457 1.00 0.93
-0.973 -3.448 -7.239 1.00 0.93
1.543 -7.149 -9.380 1.00 0.97
2.006 -8.051 -10.442 1.00 0.97
1.605 -7.465 -11.812 1.00 0.97
0.761 -6.540 -11.850 1.00 0.97
1.411 -9.458 -10.252 1.00 0.97
-0.000 -9.446 -10.505 1.00 0.97
1.806 -10.084 -8.910 1.00 0.97
2.059 -8.036 -12.919 1.00 0.92
1.600 -7.649 -14.269 1.00 0.92
0.093 -7.906 -14.443 1.00 0.92
-0.616 -7.087 -15.016 1.00 0.92

6785	2.432	-8.501	-15.225	1.00	0.92
6786	2.718	-9.767	-14.419	1.00	0.92
6787	2.972	-9.208	-13.024	1.00	0.92
6788	-0.396	-8.962	-13.781	1.00	0.80
6789	-1.824	-9.319	-13.804	1.00	0.80
6790	-2.657	-8.260	-13.066	1.00	0.80
6791	-3.640	-7.779	-13.604	1.00	0.80
6792	-2.042	-10.714	-13.208	1.00	0.80
6793	-3.506	-11.143	-13.345	1.00	0.80
6794	-3.693	-12.619	-13.008	1.00	0.80
6795	-5.165	-13.010	-13.145	1.00	0.80
6796	-5.333	-14.465	-13.032	1.00	0.80
6797	-2.126	-7.786	-11.933	1.00	0.86
6798	-2.758	-6.689	-11.175	1.00	0.86
6799	-2.838	-5.398	-11.994	1.00	0.86
6800	-3.905	-4.793	-12.086	1.00	0.86
6801	-1.988	-6.387	-9.887	1.00	0.86
6802	-2.049	-7.527	-8.872	1.00	0.86
6803	-3.089	-8.220	-8.821	1.00	0.86
6804	-1.000	-7.726	-8.227	1.00	0.86
6805	-1.736	-5.093	-12.677	1.00	0.82
6806	-1.622	-3.881	-13.503	1.00	0.82
6807	-2.587	-3.915	-14.687	1.00	0.82
6808	-3.393	-3.014	-14.811	1.00	0.82
6809	-0.199	-3.678	-14.018	1.00	0.82
6810	0.794	-3.452	-12.880	1.00	0.82
6811	2.162	-3.100	-13.455	1.00	0.82
6812	3.197	-2.936	-12.343	1.00	0.82
6813	4.482	-2.508	-12.907	1.00	0.82
6814	-2.676	-5.073	-15.357	1.00	0.86
6815	-3.606	-5.261	-16.486	1.00	0.86
6816	-5.065	-5.140	-16.030	1.00	0.86
6817	-5.832	-4.360	-16.598	1.00	0.86
6818	-3.378	-6.637	-17.117	1.00	0.86
6819	-5.355	-5.756	-14.888	1.00	0.85
6820	-6.687	-5.716	-14.250	1.00	0.85
6821	-7.109	-4.269	-13.963	1.00	0.85
6822	-8.138	-3.812	-14.475	1.00	0.85
6823	-6.688	-6.547	-12.959	1.00	0.85
6824	-6.391	-7.906	-13.280	1.00	0.85

6825	ATOM	2471	CG2	THR	C	297
6826	ATOM	2472	N	VAL	C	298
6827	ATOM	2473	CA	VAL	C	298
6828	ATOM	2474	C	VAL	C	298
6829	ATOM	2475	O	VAL	C	298
6830	ATOM	2476	CB	VAL	C	298
6831	ATOM	2477	CG1	VAL	C	298
6832	ATOM	2478	CG2	VAL	C	298
6833	ATOM	2479	N	LYS	C	299
6834	ATOM	2480	CA	LYS	C	299
6835	ATOM	2481	C	LYS	C	299
6836	ATOM	2482	O	LYS	C	299
6837	ATOM	2483	CB	LYS	C	299
6838	ATOM	2484	CG	LYS	C	299
6839	ATOM	2485	CD	LYS	C	299
6840	ATOM	2486	CE	LYS	C	299
6841	ATOM	2487	NZ	LYS	C	299
6842	ATOM	2488	N	LEU	C	300
6843	ATOM	2489	CA	LEU	C	300
6844	ATOM	2490	C	LEU	C	300
6845	ATOM	2491	O	LEU	C	300
6846	ATOM	2492	CB	LEU	C	300
6847	ATOM	2493	CG	LEU	C	300
6848	ATOM	2494	CD1	LEU	C	300
6849	ATOM	2495	CD2	LEU	C	300
6850	ATOM	2496	N	PHE	C	301
6851	ATOM	2497	CA	PHE	C	301
6852	ATOM	2498	C	PHE	C	301
6853	ATOM	2499	O	PHE	C	301
6854	ATOM	2500	CB	PHE	C	301
6855	ATOM	2501	CG	PHE	C	301
6856	ATOM	2502	CD1	PHE	C	301
6857	ATOM	2503	CD2	PHE	C	301
6858	ATOM	2504	CE1	PHE	C	301
6859	ATOM	2505	CE2	PHE	C	301
6860	ATOM	2506	CZ	PHE	C	301
6861	ATOM	2507	N	TRP	C	302
6862	ATOM	2508	CA	TRP	C	302
6863	ATOM	2509	C	TRP	C	302
6864	ATOM	2510	O	TRP	C	302

```
ATOM   2511  CB  TRP C 302
ATOM   2512  CG  TRP C 302
ATOM   2513  CD1 TRP C 302
ATOM   2514  CD2 TRP C 302
ATOM   2515  NE1 TRP C 302
ATOM   2516  CE2 TRP C 302
ATOM   2517  CE3 TRP C 302
ATOM   2518  CZ2 TRP C 302
ATOM   2519  CZ3 TRP C 302
ATOM   2520  CH2 TRP C 302
ATOM   2521  N   GLY C 303
ATOM   2522  CA  GLY C 303
ATOM   2523  C   GLY C 303
ATOM   2524  O   GLY C 303
  -8.038  -6.495 -12.237  1.00  0.85
  -6.301  -3.569 -13.180  1.00  0.87
  -6.555  -2.154 -12.822  1.00  0.87
  -6.683  -1.274 -14.080  1.00  0.87
  -7.600  -0.443 -14.150  1.00  0.87
  -5.452  -1.618 -11.888  1.00  0.87
  -5.641  -0.133 -11.548  1.00  0.87
  -5.424  -2.402 -10.573  1.00  0.87
  -5.831  -1.513 -15.067  1.00  0.79
  -5.809  -0.722 -16.322  1.00  0.79
  -7.108  -0.910 -17.102  1.00  0.79
  -7.754   0.056 -17.514  1.00  0.79
  -4.686  -1.139 -17.277  1.00  0.79
  -3.318  -0.891 -16.671  1.00  0.79
  -2.095  -1.157 -17.544  1.00  0.79
  -0.792  -0.816 -16.809  1.00  0.79
   0.365  -1.060 -17.668  1.00  0.79
  -7.501  -2.172 -17.204  1.00  0.80
  -8.707  -2.590 -17.925  1.00  0.80
  -9.976  -2.032 -17.268  1.00  0.80
 -10.811  -1.418 -17.927  1.00  0.80
  -8.696  -4.120 -17.968  1.00  0.80
  -9.787  -4.688 -18.872  1.00  0.80
  -9.686  -4.226 -20.330  1.00  0.80
  -9.907  -6.204 -18.726  1.00  0.80
 -10.008  -2.120 -15.940  1.00  0.80
```

-11.121	-1.591	-15.144	1.00	0.80
-11.251	-0.073	-15.294	1.00	0.80
-12.327	0.431	-15.601	1.00	0.80
-10.900	-1.927	-13.672	1.00	0.80
-12.052	-1.383	-12.864	1.00	0.80
-11.933	-0.181	-12.151	1.00	0.80
-13.145	-2.218	-12.652	1.00	0.80
-12.883	0.155	-11.214	1.00	0.80
-14.018	-1.932	-11.689	1.00	0.80
-13.897	-0.708	-10.933	1.00	0.80
-10.137	0.631	-15.078	1.00	0.77
-10.106	2.094	-15.186	1.00	0.77
-10.530	2.559	-16.584	1.00	0.77
-11.327	3.459	-16.701	1.00	0.77
-8.715	2.644	-14.880	1.00	0.77
-8.807	4.154	-14.670	1.00	0.77
-8.412	5.117	-15.495	1.00	0.77
-9.354	4.760	-13.541	1.00	0.77
-8.673	6.304	-14.929	1.00	0.77
-9.256	6.121	-13.765	1.00	0.77
-9.953	4.253	-12.413	1.00	0.77
-9.805	7.002	-12.815	1.00	0.77
-10.502	5.128	-11.473	1.00	0.77
-10.424	6.500	-11.689	1.00	0.77
-10.167	1.749	-17.603	1.00	0.80
-10.627	1.987	-18.984	1.00	0.80
-12.164	2.015	-19.078	1.00	0.80
-12.734	2.842	-19.791	1.00	0.80
ATOM	2525	N	ARG C 304	
ATOM	2526	CA	ARG C 304	
ATOM	2527	C	ARG C 304	
ATOM	2528	O	ARG C 304	
ATOM	2529	CB	ARG C 304	
ATOM	2530	CG	ARG C 304	
ATOM	2531	CD	ARG C 304	
ATOM	2532	NE	ARG C 304	
ATOM	2533	CZ	ARG C 304	
ATOM	2534	NH1	ARG C 304	
ATOM	2535	NH2	ARG C 304	
ATOM	2536	N	MET C 305	

6945	ATOM	2537	CA	MET	C	305
6946	ATOM	2538	C	MET	C	305
6947	ATOM	2539	O	MET	C	305
6948	ATOM	2540	CB	MET	C	305
6949	ATOM	2541	CG	MET	C	305
6950	ATOM	2542	SD	MET	C	305
6951	ATOM	2543	CE	MET	C	305
6952	ATOM	2544	N	SER	C	306
6953	ATOM	2545	CA	SER	C	306
6954	ATOM	2546	C	SER	C	306
6955	ATOM	2547	O	SER	C	306
6956	ATOM	2548	CB	SER	C	306
6957	ATOM	2549	OG	SER	C	306
6958	ATOM	2550	N	GLY	C	307
6959	ATOM	2551	CA	GLY	C	307
6960	ATOM	2552	C	GLY	C	307
6961	ATOM	2553	O	GLY	C	307
6962	ATOM	2554	N	LYS	C	308
6963	ATOM	2555	CA	LYS	C	308
6964	ATOM	2556	C	LYS	C	308
6965	ATOM	2557	O	LYS	C	308
6966	ATOM	2558	CB	LYS	C	308
6967	ATOM	2559	CG	LYS	C	308
6968	ATOM	2560	CD	LYS	C	308
6969	ATOM	2561	CE	LYS	C	308
6970	ATOM	2562	NZ	LYS	C	308
6971	ATOM	2563	N	ALA	C	309
6972	ATOM	2564	CA	ALA	C	309
6973	ATOM	2565	C	ALA	C	309
6974	ATOM	2566	O	ALA	C	309
6975	ATOM	2567	CB	ALA	C	309
6976	ATOM	2568	N	GLU	C	310
6977	ATOM	2569	CA	GLU	C	310
6978	ATOM	2570	C	GLU	C	310
6979	ATOM	2571	O	GLU	C	310
6980	ATOM	2572	CB	GLU	C	310
6981	ATOM	2573	CG	GLU	C	310
6982	ATOM	2574	CD	GLU	C	310
6983	ATOM	2575	OE1	GLU	C	310
6984	ATOM	2576	OE2	GLU	C	310

ATOM 2577 N LEU C 311
ATOM 2578 CA LEU C 311
-12.805 1.223 -18.225 1.00 0.69
-14.272 1.080 -18.167 1.00 0.69
-14.997 2.109 -17.288 1.00 0.69
-16.066 2.590 -17.658 1.00 0.69
-14.657 -0.316 -17.678 1.00 0.69
-14.274 -1.399 -18.684 1.00 0.69
-14.889 -2.725 -18.242 1.00 0.69
-14.571 -3.788 -19.209 1.00 0.69
-13.412 -4.445 -19.289 1.00 0.69
-12.455 -4.165 -18.430 1.00 0.69
-13.226 -5.393 -20.198 1.00 0.69
-14.398 2.471 -16.162 1.00 0.76
-15.087 3.321 -15.168 1.00 0.76
-14.559 4.734 -15.052 1.00 0.76
-15.282 5.608 -14.544 1.00 0.76
-14.990 2.721 -13.788 1.00 0.76
-15.712 1.402 -13.799 1.00 0.76
-15.996 1.009 -12.064 1.00 0.76
-16.433 -0.620 -12.571 1.00 0.76
-13.360 4.955 -15.562 1.00 0.76
-12.665 6.261 -15.540 1.00 0.76
-13.569 7.432 -15.952 1.00 0.76
-13.471 8.512 -15.397 1.00 0.76
-11.501 6.276 -16.525 1.00 0.76
-10.763 7.493 -16.461 1.00 0.76
-14.540 7.115 -16.839 1.00 0.75
-15.504 8.108 -17.348 1.00 0.75
-16.374 8.737 -16.246 1.00 0.75
-16.690 9.925 -16.294 1.00 0.75
-16.634 7.964 -15.199 1.00 0.74
-17.525 8.360 -14.089 1.00 0.74
-16.739 8.688 -12.813 1.00 0.74
-17.309 8.744 -11.714 1.00 0.74
-18.486 7.198 -13.823 1.00 0.74
-19.393 6.890 -15.023 1.00 0.74
-20.367 8.027 -15.348 1.00 0.74
-21.370 8.207 -14.210 1.00 0.74
-22.313 9.298 -14.471 1.00 0.74

7025	-15.494 9.111 -12.995 1.00 0.82
7026	-14.570 9.412 -11.884 1.00 0.82
7027	-15.099 10.501 -10.936 1.00 0.82
7028	-15.067 10.335 -9.723 1.00 0.82
7029	-13.208 9.836 -12.433 1.00 0.82
7030	-15.752 11.516 -11.508 1.00 0.77
7031	-16.297 12.638 -10.716 1.00 0.77
7032	-17.473 12.204 -9.830 1.00 0.77
7033	-17.558 12.569 -8.661 1.00 0.77
7034	-16.751 13.775 -11.628 1.00 0.77
7035	-15.578 14.378 -12.406 1.00 0.77
7036	-16.038 15.538 -13.290 1.00 0.77
7037	-16.732 16.420 -12.768 1.00 0.77
7038	-15.755 15.503 -14.501 1.00 0.77
7039	-18.303 11.327 -10.397 1.00 0.82
7040	-19.451 10.752 -9.681 1.00 0.82
7041	ATOM 2579 C LEU C 311
7042	ATOM 2580 O LEU C 311
7043	ATOM 2581 CB LEU C 311
7044	ATOM 2582 CG LEU C 311
7045	ATOM 2583 CD1 LEU C 311
7046	ATOM 2584 CD2 LEU C 311
7047	ATOM 2585 N ILE C 312
7048	ATOM 2586 CA ILE C 312
7049	ATOM 2587 C ILE C 312
7050	ATOM 2588 O ILE C 312
7051	ATOM 2589 CB ILE C 312
7052	ATOM 2590 CG1 ILE C 312
7053	ATOM 2591 CG2 ILE C 312
7054	ATOM 2592 CD1 ILE C 312
7055	ATOM 2593 N GLY C 313
7056	ATOM 2594 CA GLY C 313
7057	ATOM 2595 C GLY C 313
7058	ATOM 2596 O GLY C 313
7059	ATOM 2597 N ALA C 314
7060	ATOM 2598 CA ALA C 314
7061	ATOM 2599 C ALA C 314
7062	ATOM 2600 O ALA C 314
7063	ATOM 2601 CB ALA C 314
7064	ATOM 2602 N ASP C 315

ATOM	2603	CA	ASP	C	315
ATOM	2604	C	ASP	C	315
ATOM	2605	O	ASP	C	315
ATOM	2606	CB	ASP	C	315
ATOM	2607	CG	ASP	C	315
ATOM	2608	OD1	ASP	C	315
ATOM	2609	OD2	ASP	C	315
ATOM	2610	N	ALA	C	316
ATOM	2611	CA	ALA	C	316
ATOM	2612	C	ALA	C	316
ATOM	2613	O	ALA	C	316
ATOM	2614	CB	ALA	C	316
ATOM	2615	N	LEU	C	317
ATOM	2616	CA	LEU	C	317
ATOM	2617	C	LEU	C	317
ATOM	2618	O	LEU	C	317
ATOM	2619	CB	LEU	C	317
ATOM	2620	CG	LEU	C	317
ATOM	2621	CD1	LEU	C	317
ATOM	2622	CD2	LEU	C	317
ATOM	2623	N	SER	C	318
ATOM	2624	CA	SER	C	318
ATOM	2625	C	SER	C	318
ATOM	2626	O	SER	C	318
ATOM	2627	CB	SER	C	318
ATOM	2628	OG	SER	C	318
ATOM	2629	N	ARG	C	319
ATOM	2630	CA	ARG	C	319
ATOM	2631	C	ARG	C	319
ATOM	2632	O	ARG	C	319

-19.001	9.810	-8.557	1.00	0.82
-19.438	9.961	-7.410	1.00	0.82
-20.356	10.015	-10.675	1.00	0.82
-21.644	9.519	-10.006	1.00	0.82
-22.489	10.668	-9.450	1.00	0.82
-22.475	8.691	-10.981	1.00	0.82
-18.016	8.975	-8.870	1.00	0.84
-17.423	8.030	-7.901	1.00	0.84
-16.815	8.816	-6.726	1.00	0.84
-17.062	8.490	-5.569	1.00	0.84

7105	-16.355	7.130	-8.556	1.00	0.84
7106	-16.979	6.273	-9.672	1.00	0.84
7107	-15.643	6.253	-7.505	1.00	0.84
7108	-15.977	5.416	-10.460	1.00	0.84
7109	-16.107	9.896	-7.085	1.00	0.94
7110	-15.420	10.756	-6.108	1.00	0.94
7111	-16.374	11.416	-5.120	1.00	0.94
7112	-16.177	11.319	-3.907	1.00	0.94
7113	-17.471	11.934	-5.664	1.00	0.90
7114	-18.506	12.601	-4.857	1.00	0.90
7115	-19.203	11.593	-3.936	1.00	0.90
7116	-19.203	11.755	-2.725	1.00	0.90
7117	-19.534	13.274	-5.766	1.00	0.90
7118	-19.566	10.448	-4.520	1.00	0.91
7119	-20.273	9.391	-3.789	1.00	0.91
7120	-19.416	8.801	-2.657	1.00	0.91
7121	-19.838	8.814	-1.502	1.00	0.91
7122	-20.702	8.326	-4.796	1.00	0.91
7123	-21.794	7.416	-4.239	1.00	0.91
7124	-22.523	7.850	-3.322	1.00	0.91
7125	-21.892	6.303	-4.779	1.00	0.91
7126	-18.159	8.503	-2.981	1.00	0.93
7127	-17.180	7.959	-2.021	1.00	0.93
7128	-16.905	8.907	-0.849	1.00	0.93
7129	-17.068	8.519	0.311	1.00	0.93
7130	-15.866	7.635	-2.735	1.00	0.93
7131	-16.646	10.176	-1.166	1.00	0.91
7132	-16.405	11.197	-0.130	1.00	0.91
7133	-17.611	11.426	0.776	1.00	0.91
7134	-17.483	11.491	1.993	1.00	0.91
7135	-16.020	12.548	-0.724	1.00	0.91
7136	-14.529	12.652	-1.027	1.00	0.91
7137	-14.271	14.057	-1.543	1.00	0.91
7138	-13.654	12.432	0.208	1.00	0.91
7139	-18.785	11.364	0.164	1.00	0.94
7140	-20.057	11.561	0.881	1.00	0.94
7141	-20.314	10.428	1.881	1.00	0.94
7142	-20.663	10.685	3.036	1.00	0.94
7143	-21.204	11.668	-0.122	1.00	0.94
7144	-21.023	12.850	-0.910	1.00	0.94

-19.928	9.218	1.489	1.00	0.87		
-20.027	8.019	2.345	1.00	0.87		
-19.006	8.054	3.487	1.00	0.87		
-19.354	7.796	4.638	1.00	0.87		
ATOM	2633	CB	ARG	C	319	
ATOM	2634	CG	ARG	C	319	
ATOM	2635	CD	ARG	C	319	
ATOM	2636	NE	ARG	C	319	
ATOM	2637	CZ	ARG	C	319	
ATOM	2638	NH1	ARG	C	319	
ATOM	2639	NH2	ARG	C	319	
ATOM	2640	N	MET	C	320	
ATOM	2641	CA	MET	C	320	
ATOM	2642	C	MET	C	320	
ATOM	2643	O	MET	C	320	
ATOM	2644	CB	MET	C	320	
ATOM	2645	CG	MET	C	320	
ATOM	2646	SD	MET	C	320	
ATOM	2647	CE	MET	C	320	
ATOM	2648	N	LEU	C	321	
ATOM	2649	CA	LEU	C	321	
ATOM	2650	C	LEU	C	321	
ATOM	2651	O	LEU	C	321	
ATOM	2652	CB	LEU	C	321	
ATOM	2653	CG	LEU	C	321	
ATOM	2654	CD1	LEU	C	321	
ATOM	2655	CD2	LEU	C	321	
ATOM	2656	N	ALA	C	322	
ATOM	2657	CA	ALA	C	322	
ATOM	2658	C	ALA	C	322	
ATOM	2659	O	ALA	C	322	
ATOM	2660	CB	ALA	C	322	
ATOM	2661	N	VAL	C	323	
ATOM	2662	CA	VAL	C	323	
ATOM	2663	C	VAL	C	323	
ATOM	2664	O	VAL	C	323	
ATOM	2665	CB	VAL	C	323	
ATOM	2666	CG1	VAL	C	323	
ATOM	2667	CG2	VAL	C	323	
ATOM	2668	N	TYR	C	324	

ATOM 2669 CA TYR C 324
ATOM 2670 C TYR C 324
ATOM 2671 O TYR C 324
ATOM 2672 CB TYR C 324
ATOM 2673 CG TYR C 324
ATOM 2674 CD1 TYR C 324
ATOM 2675 CD2 TYR C 324
ATOM 2676 CE1 TYR C 324
ATOM 2677 CE2 TYR C 324
ATOM 2678 CZ TYR C 324
ATOM 2679 OH TYR C 324
ATOM 2680 N PRO C 325
ATOM 2681 CA PRO C 325
ATOM 2682 C PRO C 325
ATOM 2683 O PRO C 325
ATOM 2684 CB PRO C 325
ATOM 2685 CG PRO C 325
ATOM 2686 CD PRO C 325
-19.800 6.759 1.514 1.00 0.87
-20.907 6.582 0.479 1.00 0.87
-20.561 5.420 -0.444 1.00 0.87
-21.665 5.210 -1.396 1.00 0.87
-22.770 4.509 -1.155 1.00 0.87
-22.976 3.934 0.024 1.00 0.87
-23.679 4.371 -2.105 1.00 0.87
-17.812 8.563 3.184 1.00 0.88
-16.734 8.703 4.179 1.00 0.88
-17.130 9.662 5.310 1.00 0.88
-16.873 9.375 6.476 1.00 0.88
-15.448 9.194 3.509 1.00 0.88
-14.300 9.269 4.524 1.00 0.88
-12.761 10.025 3.891 1.00 0.88
-13.275 11.710 3.664 1.00 0.88
-17.774 10.766 4.955 1.00 0.87
-18.184 11.787 5.938 1.00 0.87
-19.373 11.342 6.794 1.00 0.87
-19.455 11.680 7.970 1.00 0.87
-18.492 13.117 5.242 1.00 0.87
-17.241 13.962 4.949 1.00 0.87
-16.264 13.298 3.995 1.00 0.87

-17.636	15.238	4.223	1.00	0.87	
-20.229	10.518	6.190	1.00	0.90	
-21.446	10.020	6.851	1.00	0.90	
-21.157	8.803	7.741	1.00	0.90	
-21.447	8.816	8.937	1.00	0.90	
-22.497	9.676	5.793	1.00	0.90	
-20.491	7.814	7.163	1.00	0.87	
-20.191	6.532	7.840	1.00	0.87	
-19.000	6.687	8.800	1.00	0.87	
-18.892	5.966	9.796	1.00	0.87	
-19.942	5.416	6.805	1.00	0.87	
-19.710	4.056	7.473	1.00	0.87	
-21.118	5.267	5.832	1.00	0.87	
-18.090	7.587	8.453	1.00	0.86	
-16.864	7.820	9.235	1.00	0.86	
-16.778	9.316	9.585	1.00	0.86	
-15.839	10.005	9.173	1.00	0.86	
-15.644	7.319	8.453	1.00	0.86	
-15.814	5.864	8.023	1.00	0.86	
-15.466	4.849	8.900	1.00	0.86	
-16.288	5.570	6.750	1.00	0.86	
-15.579	3.526	8.498	1.00	0.86	
-16.396	4.249	6.343	1.00	0.86	
-16.034	3.231	7.218	1.00	0.86	
-16.113	1.939	6.812	1.00	0.86	
-17.640	9.764	10.501	1.00	0.87	
-17.753	11.186	10.896	1.00	0.87	
-16.483	11.812	11.488	1.00	0.87	
-16.278	13.030	11.415	1.00	0.87	
-18.924	11.220	11.874	1.00	0.87	
-18.944	9.825	12.492	1.00	0.87	
-18.556	8.935	11.318	1.00	0.87	
ATOM	2687	N	GLN	C	326
ATOM	2688	CA	GLN	C	326
ATOM	2689	C	GLN	C	326
ATOM	2690	O	GLN	C	326
ATOM	2691	CB	GLN	C	326
ATOM	2692	CG	GLN	C	326
ATOM	2693	CD	GLN	C	326
ATOM	2694	OE1	GLN	C	326

7265	ATOM	2695	NE2	GLN	C	326
7266	ATOM	2696	N	THR	C	327
7267	ATOM	2697	CA	THR	C	327
7268	ATOM	2698	C	THR	C	327
7269	ATOM	2699	O	THR	C	327
7270	ATOM	2700	CB	THR	C	327
7271	ATOM	2701	OG1	THR	C	327
7272	ATOM	2702	CG2	THR	C	327
7273	ATOM	2703	N	LYS	C	328
7274	ATOM	2704	CA	LYS	C	328
7275	ATOM	2705	C	LYS	C	328
7276	ATOM	2706	O	LYS	C	328
7277	ATOM	2707	CB	LYS	C	328
7278	ATOM	2708	CG	LYS	C	328
7279	ATOM	2709	CD	LYS	C	328
7280	ATOM	2710	CE	LYS	C	328
7281	ATOM	2711	NZ	LYS	C	328
7282	ATOM	2712	N	THR	C	329
7283	ATOM	2713	CA	THR	C	329
7284	ATOM	2714	C	THR	C	329
7285	ATOM	2715	O	THR	C	329
7286	ATOM	2716	CB	THR	C	329
7287	ATOM	2717	OG1	THR	C	329
7288	ATOM	2718	CG2	THR	C	329
7289	ATOM	2719	N	TYR	C	330
7290	ATOM	2720	CA	TYR	C	330
7291	ATOM	2721	C	TYR	C	330
7292	ATOM	2722	O	TYR	C	330
7293	ATOM	2723	CB	TYR	C	330
7294	ATOM	2724	CG	TYR	C	330
7295	ATOM	2725	CD1	TYR	C	330
7296	ATOM	2726	CD2	TYR	C	330
7297	ATOM	2727	CE1	TYR	C	330
7298	ATOM	2728	CE2	TYR	C	330
7299	ATOM	2729	CZ	TYR	C	330
7300	ATOM	2730	OH	TYR	C	330
7301	ATOM	2731	N	PHE	C	331
7302	ATOM	2732	CA	PHE	C	331
7303	ATOM	2733	C	PHE	C	331
7304	ATOM	2734	O	PHE	C	331

ATOM 2735 CB PHE C 331
ATOM 2736 CG PHE C 331
ATOM 2737 CD1 PHE C 331
ATOM 2738 CD2 PHE C 331
ATOM 2739 CE1 PHE C 331
ATOM 2740 CE2 PHE C 331
-15.603 11.021 12.055 1.00 0.81
-14.306 11.504 12.591 1.00 0.81
-13.408 12.105 11.501 1.00 0.81
-12.647 13.023 11.791 1.00 0.81
-13.482 10.470 13.365 1.00 0.81
-13.018 9.253 12.543 1.00 0.81
-14.102 8.190 12.340 1.00 0.81
-13.825 7.061 11.963 1.00 0.81
-15.345 8.530 12.613 1.00 0.81
-13.506 11.591 10.276 1.00 0.88
-12.739 12.137 9.129 1.00 0.88
-13.093 13.600 8.813 1.00 0.88
-12.382 14.263 8.057 1.00 0.88
-12.912 11.319 7.843 1.00 0.88
-14.257 11.432 7.378 1.00 0.88
-12.520 9.855 8.047 1.00 0.88
-14.160 14.100 9.431 1.00 0.81
-14.611 15.494 9.271 1.00 0.81
-13.650 16.505 9.893 1.00 0.81
-13.591 17.651 9.444 1.00 0.81
-16.003 15.641 9.875 1.00 0.81
-16.980 14.878 8.978 1.00 0.81
-18.313 14.546 9.647 1.00 0.81
-19.145 15.735 10.128 1.00 0.81
-20.227 15.205 10.953 1.00 0.81
-12.834 16.034 10.832 1.00 0.83
-11.777 16.839 11.482 1.00 0.83
-10.898 17.597 10.470 1.00 0.83
-10.592 18.766 10.674 1.00 0.83
-10.874 15.962 12.356 1.00 0.83
-10.340 14.887 11.576 1.00 0.83
-11.622 15.441 13.588 1.00 0.83
-10.669 16.969 9.315 1.00 0.80
-9.879 17.560 8.216 1.00 0.80

7345	-10.590	18.632	7.379	1.00	0.80
7346	-9.935	19.393	6.659	1.00	0.80
7347	-9.346	16.457	7.301	1.00	0.80
7348	-8.381	15.561	8.077	1.00	0.80
7349	-7.079	15.984	8.308	1.00	0.80
7350	-8.831	14.354	8.594	1.00	0.80
7351	-6.218	15.197	9.059	1.00	0.80
7352	-7.969	13.562	9.341	1.00	0.80
7353	-6.664	13.987	9.572	1.00	0.80
7354	-5.812	13.206	10.284	1.00	0.80
7355	-11.905	18.726	7.496	1.00	0.77
7356	-12.694	19.638	6.641	1.00	0.77
7357	-13.458	20.692	7.442	1.00	0.77
7358	-14.432	21.280	6.957	1.00	0.77
7359	-13.656	18.836	5.768	1.00	0.77
7360	-12.950	17.760	4.959	1.00	0.77
7361	-12.324	18.124	3.780	1.00	0.77
7362	-12.929	16.445	5.406	1.00	0.77
7363	-11.674	17.162	3.026	1.00	0.77
7364	-12.273	15.484	4.653	1.00	0.77

7365	ATOM	2741	CZ	PHE	C	331
7366	ATOM	2742	N	SER	C	332
7367	ATOM	2743	CA	SER	C	332
7368	ATOM	2744	C	SER	C	332
7369	ATOM	2745	O	SER	C	332
7370	ATOM	2746	CB	SER	C	332
7371	ATOM	2747	OG	SER	C	332
7372	ATOM	2748	N	HIS	C	333
7373	ATOM	2749	CA	HIS	C	333
7374	ATOM	2750	C	HIS	C	333
7375	ATOM	2751	O	HIS	C	333
7376	ATOM	2752	CB	HIS	C	333
7377	ATOM	2753	CG	HIS	C	333
7378	ATOM	2754	ND1	HIS	C	333
7379	ATOM	2755	CD2	HIS	C	333
7380	ATOM	2756	CE1	HIS	C	333
7381	ATOM	2757	NE2	HIS	C	333
7382	ATOM	2758	N	TRP	C	334
7383	ATOM	2759	CA	TRP	C	334
7384	ATOM	2760	C	TRP	C	334

ATOM 2761 O TRP C 334
ATOM 2762 CB TRP C 334
ATOM 2763 CG TRP C 334
ATOM 2764 CD1 TRP C 334
ATOM 2765 CD2 TRP C 334
ATOM 2766 NE1 TRP C 334
ATOM 2767 CE2 TRP C 334
ATOM 2768 CE3 TRP C 334
ATOM 2769 CZ2 TRP C 334
ATOM 2770 CZ3 TRP C 334
ATOM 2771 CH2 TRP C 334
ATOM 2772 N LYS C 335
ATOM 2773 CA LYS C 335
ATOM 2774 C LYS C 335
ATOM 2775 O LYS C 335
ATOM 2776 CB LYS C 335
ATOM 2777 CG LYS C 335
ATOM 2778 CD LYS C 335
ATOM 2779 CE LYS C 335
ATOM 2780 NZ LYS C 335
ATOM 2781 N SER C 336
ATOM 2782 CA SER C 336
ATOM 2783 C SER C 336
ATOM 2784 O SER C 336
ATOM 2785 CB SER C 336
ATOM 2786 OG SER C 336
ATOM 2787 N LEU C 337
ATOM 2788 CA LEU C 337
ATOM 2789 C LEU C 337
ATOM 2790 O LEU C 337
ATOM 2791 CB LEU C 337
ATOM 2792 CG LEU C 337
ATOM 2793 CD1 LEU C 337
ATOM 2794 CD2 LEU C 337
-11.645 15.843 3.464 1.00 0.77
-12.898 21.055 8.587 1.00 0.75
-13.465 22.117 9.442 1.00 0.75
-13.341 23.532 8.854 1.00 0.75
-14.022 24.453 9.284 1.00 0.75
-12.871 22.063 10.845 1.00 0.75

7425	-11.443	22.040	10.795	1.00	0.75
7426	-12.471	23.680	7.853	1.00	0.65
7427	-12.335	24.932	7.084	1.00	0.65
7428	-13.544	25.219	6.175	1.00	0.65
7429	-13.756	26.357	5.761	1.00	0.65
7430	-11.013	24.957	6.302	1.00	0.65
7431	-10.837	23.766	5.356	1.00	0.65
7432	-10.675	22.496	5.718	1.00	0.65
7433	-10.694	23.836	4.037	1.00	0.65
7434	-10.439	21.781	4.624	1.00	0.65
7435	-10.451	22.610	3.583	1.00	0.65
7436	-14.301	24.171	5.843	1.00	0.70
7437	-15.543	24.313	5.068	1.00	0.70
7438	-16.740	24.397	6.014	1.00	0.70
7439	-16.845	23.648	6.986	1.00	0.70
7440	-15.759	23.112	4.139	1.00	0.70
7441	-14.650	22.915	3.111	1.00	0.70
7442	-13.827	23.809	2.601	1.00	0.70
7443	-14.356	21.655	2.512	1.00	0.70
7444	-13.015	23.196	1.705	1.00	0.70
7445	-13.309	21.961	1.654	1.00	0.70
7446	-14.934	20.455	2.607	1.00	0.70
7447	-12.823	20.866	0.839	1.00	0.70
7448	-14.474	19.392	1.786	1.00	0.70
7449	-13.411	19.655	0.923	1.00	0.70
7450	-17.680	25.250	5.635	1.00	0.73
7451	-18.962	25.388	6.357	1.00	0.73
7452	-19.978	24.290	5.990	1.00	0.73
7453	-20.901	24.001	6.744	1.00	0.73
7454	-19.564	26.777	6.115	1.00	0.73
7455	-19.919	27.022	4.645	1.00	0.73
7456	-20.544	28.400	4.450	1.00	0.73
7457	-20.929	28.628	2.984	1.00	0.73
7458	-21.986	27.704	2.544	1.00	0.73
7459	-19.803	23.707	4.804	1.00	0.84
7460	-20.798	22.789	4.222	1.00	0.84
7461	-20.243	21.366	4.104	1.00	0.84
7462	-20.190	20.755	3.035	1.00	0.84
7463	-21.270	23.325	2.865	1.00	0.84
7464	-20.190	23.304	1.926	1.00	0.84

7465	-19.790	20.827	5.220	1.00	0.83	
7466	-19.350	19.427	5.247	1.00	0.83	
7467	-20.611	18.556	5.345	1.00	0.83	
7468	-21.135	18.257	6.427	1.00	0.83	
7469	-18.366	19.271	6.402	1.00	0.83	
7470	-17.701	17.901	6.403	1.00	0.83	
7471	-16.489	17.961	7.314	1.00	0.83	
7472	-18.654	16.844	6.947	1.00	0.83	
7473	ATOM	2795	N	SER	C	338
7474	ATOM	2796	CA	SER	C	338
7475	ATOM	2797	C	SER	C	338
7476	ATOM	2798	O	SER	C	338
7477	ATOM	2799	CB	SER	C	338
7478	ATOM	2800	OG	SER	C	338
7479	ATOM	2801	N	PRO	C	339
7480	ATOM	2802	CA	PRO	C	339
7481	ATOM	2803	C	PRO	C	339
7482	ATOM	2804	O	PRO	C	339
7483	ATOM	2805	CB	PRO	C	339
7484	ATOM	2806	CG	PRO	C	339
7485	ATOM	2807	CD	PRO	C	339
7486	ATOM	2808	N	GLY	C	340
7487	ATOM	2809	CA	GLY	C	340
7488	ATOM	2810	C	GLY	C	340
7489	ATOM	2811	O	GLY	C	340
7490	ATOM	2812	N	SER	C	341
7491	ATOM	2813	CA	SER	C	341
7492	ATOM	2814	C	SER	C	341
7493	ATOM	2815	O	SER	C	341
7494	ATOM	2816	CB	SER	C	341
7495	ATOM	2817	OG	SER	C	341
7496	ATOM	2818	N	PRO	C	342
7497	ATOM	2819	CA	PRO	C	342
7498	ATOM	2820	C	PRO	C	342
7499	ATOM	2821	O	PRO	C	342
7500	ATOM	2822	CB	PRO	C	342
7501	ATOM	2823	CG	PRO	C	342
7502	ATOM	2824	CD	PRO	C	342
7503	ATOM	2825	N	ASP	C	343
7504	ATOM	2826	CA	ASP	C	343

ATOM	2827	C	ASP C 343		
ATOM	2828	O	ASP C 343		
ATOM	2829	CB	ASP C 343		
ATOM	2830	CG	ASP C 343		
ATOM	2831	OD1	ASP C 343		
ATOM	2832	OD2	ASP C 343		
ATOM	2833	N	VAL C 344		
ATOM	2834	CA	VAL C 344		
ATOM	2835	C	VAL C 344		
ATOM	2836	O	VAL C 344		
ATOM	2837	CB	VAL C 344		
ATOM	2838	CG1	VAL C 344		
ATOM	2839	CG2	VAL C 344		
ATOM	2840	N	LYS C 345		
ATOM	2841	CA	LYS C 345		
ATOM	2842	C	LYS C 345		
ATOM	2843	O	LYS C 345		
ATOM	2844	CB	LYS C 345		
ATOM	2845	CG	LYS C 345		
ATOM	2846	CD	LYS C 345		
ATOM	2847	CE	LYS C 345		
ATOM	2848	NZ	LYS C 345		
-21.170	18.289	4.188	1.00	0.90	
-22.423	17.533	4.056	1.00	0.90	
-22.428	16.779	2.726	1.00	0.90	
-21.869	17.313	1.739	1.00	0.90	
-23.619	18.491	4.142	1.00	0.90	
-24.839	17.749	4.062	1.00	0.90	
-23.013	15.593	2.679	1.00	0.96	
-23.056	14.717	1.492	1.00	0.96	
-23.508	15.483	0.244	1.00	0.96	
-24.578	16.082	0.209	1.00	0.96	
-24.092	13.652	1.830	1.00	0.96	
-23.947	13.489	3.339	1.00	0.96	
-23.682	14.910	3.821	1.00	0.96	
-22.622	15.496	-0.748	1.00	0.98	
-22.950	16.173	-2.030	1.00	0.98	
-22.983	17.711	-1.942	1.00	0.98	
-23.477	18.354	-2.873	1.00	0.98	
-22.393	18.336	-0.917	1.00	0.94	

7545	-22.183	19.792	-0.899	1.00	0.94
7546	-21.222	20.188	-2.058	1.00	0.94
7547	-20.434	19.401	-2.524	1.00	0.94
7548	-21.639	20.254	0.455	1.00	0.94
7549	-20.253	19.946	0.619	1.00	0.94
7550	-21.293	21.464	-2.510	1.00	0.94
7551	-20.417	21.944	-3.603	1.00	0.94
7552	-18.933	21.674	-3.314	1.00	0.94
7553	-18.166	21.307	-4.197	1.00	0.94
7554	-20.669	23.452	-3.633	1.00	0.94
7555	-22.094	23.592	-3.102	1.00	0.94
7556	-22.178	22.518	-2.021	1.00	0.94
7557	-18.602	21.708	-2.018	1.00	0.86
7558	-17.245	21.422	-1.520	1.00	0.86
7559	-16.876	19.943	-1.614	1.00	0.86
7560	-15.779	19.604	-2.064	1.00	0.86
7561	-17.088	21.905	-0.074	1.00	0.86
7562	-17.211	23.428	0.054	1.00	0.86
7563	-17.119	24.117	-0.988	1.00	0.86
7564	-17.474	23.874	1.188	1.00	0.86
7565	-17.836	19.080	-1.316	1.00	0.87
7566	-17.581	17.631	-1.405	1.00	0.87
7567	-17.500	17.176	-2.876	1.00	0.87
7568	-16.595	16.414	-3.213	1.00	0.87
7569	-18.570	16.774	-0.603	1.00	0.87
7570	-18.764	17.191	0.855	1.00	0.87
7571	-19.905	16.773	-1.281	1.00	0.87
7572	-18.376	17.695	-3.739	1.00	0.83
7573	-18.369	17.361	-5.177	1.00	0.83
7574	-17.059	17.793	-5.840	1.00	0.83
7575	-16.391	16.983	-6.476	1.00	0.83
7576	-19.526	18.005	-5.941	1.00	0.83
7577	-20.866	17.370	-5.579	1.00	0.83
7578	-21.971	17.882	-6.501	1.00	0.83
7579	-23.321	17.357	-6.017	1.00	0.83
7580	-24.417	17.761	-6.908	1.00	0.83
7581	ATOM	2849	N	LYS C 346	
7582	ATOM	2850	CA	LYS C 346	
7583	ATOM	2851	C	LYS C 346	
7584	ATOM	2852	O	LYS C 346	

7585	ATOM	2853	CB	LYS	C	346
7586	ATOM	2854	CG	LYS	C	346
7587	ATOM	2855	CD	LYS	C	346
7588	ATOM	2856	CE	LYS	C	346
7589	ATOM	2857	NZ	LYS	C	346
7590	ATOM	2858	N	HIS	C	347
7591	ATOM	2859	CA	HIS	C	347
7592	ATOM	2860	C	HIS	C	347
7593	ATOM	2861	O	HIS	C	347
7594	ATOM	2862	CB	HIS	C	347
7595	ATOM	2863	CG	HIS	C	347
7596	ATOM	2864	ND1	HIS	C	347
7597	ATOM	2865	CD2	HIS	C	347
7598	ATOM	2866	CE1	HIS	C	347
7599	ATOM	2867	NE2	HIS	C	347
7600	ATOM	2868	N	GLY	C	348
7601	ATOM	2869	CA	GLY	C	348
7602	ATOM	2870	C	GLY	C	348
7603	ATOM	2871	O	GLY	C	348
7604	ATOM	2872	N	LYS	C	349
7605	ATOM	2873	CA	LYS	C	349
7606	ATOM	2874	C	LYS	C	349
7607	ATOM	2875	O	LYS	C	349
7608	ATOM	2876	CB	LYS	C	349
7609	ATOM	2877	CG	LYS	C	349
7610	ATOM	2878	CD	LYS	C	349
7611	ATOM	2879	CE	LYS	C	349
7612	ATOM	2880	NZ	LYS	C	349
7613	ATOM	2881	N	THR	C	350
7614	ATOM	2882	CA	THR	C	350
7615	ATOM	2883	C	THR	C	350
7616	ATOM	2884	O	THR	C	350
7617	ATOM	2885	CB	THR	C	350
7618	ATOM	2886	OG1	THR	C	350
7619	ATOM	2887	CG2	THR	C	350
7620	ATOM	2888	N	ILE	C	351
7621	ATOM	2889	CA	ILE	C	351
7622	ATOM	2890	C	ILE	C	351
7623	ATOM	2891	O	ILE	C	351
7624	ATOM	2892	CB	ILE	C	351

ATOM 2893 CG1 ILE C 351
ATOM 2894 CG2 ILE C 351
ATOM 2895 CD1 ILE C 351
ATOM 2896 N MET C 352
ATOM 2897 CA MET C 352
ATOM 2898 C MET C 352
ATOM 2899 O MET C 352
ATOM 2900 CB MET C 352
ATOM 2901 CG MET C 352
ATOM 2902 SD MET C 352
-16.628 19.012 -5.503 1.00 0.80
-15.383 19.568 -6.057 1.00 0.80
-14.159 18.767 -5.597 1.00 0.80
-13.357 18.308 -6.410 1.00 0.80
-15.241 21.039 -5.655 1.00 0.80
-14.052 21.710 -6.350 1.00 0.80
-14.229 21.729 -7.873 1.00 0.80
-13.021 22.349 -8.576 1.00 0.80
-11.807 21.545 -8.371 1.00 0.80
-14.095 18.518 -4.291 1.00 0.83
-12.995 17.740 -3.696 1.00 0.83
-12.999 16.284 -4.200 1.00 0.83
-11.976 15.673 -4.443 1.00 0.83
-13.132 17.743 -2.176 1.00 0.83
-11.877 17.183 -1.504 1.00 0.83
-10.624 17.523 -1.786 1.00 0.83
-11.839 16.235 -0.576 1.00 0.83
-9.813 16.783 -1.037 1.00 0.83
-10.568 15.983 -0.290 1.00 0.83
-14.219 15.766 -4.423 1.00 0.91
-14.430 14.405 -4.943 1.00 0.91
-13.736 14.172 -6.289 1.00 0.91
-13.151 13.132 -6.556 1.00 0.91
-13.755 15.204 -7.122 1.00 0.79
-13.110 15.081 -8.439 1.00 0.79
-11.611 15.384 -8.411 1.00 0.79
-10.880 14.923 -9.288 1.00 0.79
-13.833 15.888 -9.508 1.00 0.79
-13.765 17.406 -9.324 1.00 0.79
-14.705 18.098 -10.308 1.00 0.79

-16.158 17.736 -9.986 1.00 0.79
-17.083 18.289 -10.979 1.00 0.79
-11.171 16.129 -7.406 1.00 0.81
-9.728 16.383 -7.200 1.00 0.81
-9.033 15.099 -6.723 1.00 0.81
-8.005 14.700 -7.285 1.00 0.81
-9.451 17.525 -6.211 1.00 0.81
-9.890 17.160 -4.905 1.00 0.81
-10.107 18.838 -6.648 1.00 0.81
-9.703 14.361 -5.848 1.00 0.82
-9.192 13.086 -5.291 1.00 0.82
-9.116 11.995 -6.374 1.00 0.82
-8.082 11.361 -6.570 1.00 0.82
-10.011 12.589 -4.080 1.00 0.82
-11.453 12.251 -4.471 1.00 0.82
-9.980 13.625 -2.951 1.00 0.82
-12.342 11.534 -3.461 1.00 0.82
-10.203 11.871 -7.134 1.00 0.82
-10.306 10.912 -8.248 1.00 0.82
-9.366 11.254 -9.401 1.00 0.82
-8.903 10.363 -10.106 1.00 0.82
-11.741 10.815 -8.755 1.00 0.82
-12.600 10.075 -7.738 1.00 0.82
-12.120 8.336 -7.450 1.00 0.82
ATOM 2903 CE MET C 352
ATOM 2904 N MET C 353
ATOM 2905 CA MET C 353
ATOM 2906 C MET C 353
ATOM 2907 O MET C 353
ATOM 2908 CB MET C 353
ATOM 2909 CG MET C 353
ATOM 2910 SD MET C 353
ATOM 2911 CE MET C 353
ATOM 2912 N GLY C 354
ATOM 2913 CA GLY C 354
ATOM 2914 C GLY C 354
ATOM 2915 O GLY C 354
ATOM 2916 N ILE C 355
ATOM 2917 CA ILE C 355
ATOM 2918 C ILE C 355

ATOM 2919 O ILE C 355
ATOM 2920 CB ILE C 355
ATOM 2921 CG1 ILE C 355
ATOM 2922 CG2 ILE C 355
ATOM 2923 CD1 ILE C 355
ATOM 2924 N GLY C 356
ATOM 2925 CA GLY C 356
ATOM 2926 C GLY C 356
ATOM 2927 O GLY C 356
ATOM 2928 N ASP C 357
ATOM 2929 CA ASP C 357
ATOM 2930 C ASP C 357
ATOM 2931 O ASP C 357
ATOM 2932 CB ASP C 357
ATOM 2933 CG ASP C 357
ATOM 2934 OD1 ASP C 357
ATOM 2935 OD2 ASP C 357
ATOM 2936 N ALA C 358
ATOM 2937 CA ALA C 358
ATOM 2938 C ALA C 358
ATOM 2939 O ALA C 358
ATOM 2940 CB ALA C 358
ATOM 2941 N VAL C 359
ATOM 2942 CA VAL C 359
ATOM 2943 C VAL C 359
ATOM 2944 O VAL C 359
ATOM 2945 CB VAL C 359
ATOM 2946 CG1 VAL C 359
ATOM 2947 CG2 VAL C 359
ATOM 2948 N THR C 360
ATOM 2949 CA THR C 360
ATOM 2950 C THR C 360
ATOM 2951 O THR C 360
ATOM 2952 CB THR C 360
ATOM 2953 OG1 THR C 360
ATOM 2954 CG2 THR C 360
ATOM 2955 N LYS C 361
ATOM 2956 CA LYS C 361
-11.954 8.424 -5.685 1.00 0.82
-9.013 12.537 -9.491 1.00 0.79

7745	-7.965	13.001	-10.416	1.00	0.79
7746	-6.597	12.423	-10.010	1.00	0.79
7747	-5.805	12.012	-10.858	1.00	0.79
7748	-7.910	14.531	-10.404	1.00	0.79
7749	-6.937	15.082	-11.452	1.00	0.79
7750	-7.419	14.681	-13.172	1.00	0.79
7751	-8.848	15.730	-13.341	1.00	0.79
7752	-6.412	12.298	-8.688	1.00	0.88
7753	-5.202	11.709	-8.079	1.00	0.88
7754	-5.113	10.197	-8.340	1.00	0.88
7755	-4.035	9.675	-8.634	1.00	0.88
7756	-6.267	9.533	-8.322	1.00	0.84
7757	-6.380	8.095	-8.666	1.00	0.84
7758	-6.093	7.888	-10.163	1.00	0.84
7759	-5.440	6.919	-10.540	1.00	0.84
7760	-7.774	7.536	-8.313	1.00	0.84
7761	-8.103	7.703	-6.819	1.00	0.84
7762	-7.972	6.080	-8.785	1.00	0.84
7763	-7.335	6.799	-5.849	1.00	0.84
7764	-6.565	8.847	-10.975	1.00	0.87
7765	-6.349	8.832	-12.434	1.00	0.87
7766	-4.848	8.906	-12.747	1.00	0.87
7767	-4.310	8.076	-13.475	1.00	0.87
7768	-4.172	9.777	-11.992	1.00	0.81
7769	-2.709	9.940	-12.058	1.00	0.81
7770	-1.985	8.639	-11.671	1.00	0.81
7771	-1.147	8.133	-12.412	1.00	0.81
7772	-2.284	11.076	-11.120	1.00	0.81
7773	-0.775	11.332	-11.179	1.00	0.81
7774	-0.292	11.615	-12.295	1.00	0.81
7775	-0.139	11.163	-10.118	1.00	0.81
7776	-2.393	8.070	-10.541	1.00	0.88
7777	-1.807	6.818	-10.025	1.00	0.88
7778	-1.949	5.648	-10.992	1.00	0.88
7779	-0.996	4.881	-11.155	1.00	0.88
7780	-2.469	6.458	-8.699	1.00	0.88
7781	-3.060	5.596	-11.735	1.00	0.86
7782	-3.298	4.596	-12.772	1.00	0.86
7783	-2.268	4.793	-13.935	1.00	0.86
7784	-1.557	3.972	-14.353	1.00	0.86

-4.744	4.616	-13.295	1.00	0.86		
-4.993	3.619	-14.432	1.00	0.86		
-5.737	4.276	-12.179	1.00	0.86		
-1.984	6.049	-14.242	1.00	0.82		
-1.019	6.264	-15.360	1.00	0.82		
0.440	6.054	-14.942	1.00	0.82		
1.298	5.679	-15.747	1.00	0.82		
-1.258	7.641	-15.960	1.00	0.82		
-0.979	8.622	-14.963	1.00	0.82		
-2.695	7.753	-16.483	1.00	0.82		
0.680	6.198	-13.643	1.00	0.84		
2.009	6.040	-13.034	1.00	0.84		
ATOM	2957	C	LYS	C	361	
ATOM	2958	O	LYS	C	361	
ATOM	2959	CB	LYS	C	361	
ATOM	2960	CG	LYS	C	361	
ATOM	2961	CD	LYS	C	361	
ATOM	2962	CE	LYS	C	361	
ATOM	2963	NZ	LYS	C	361	
ATOM	2964	N	MET	C	362	
ATOM	2965	CA	MET	C	362	
ATOM	2966	C	MET	C	362	
ATOM	2967	O	MET	C	362	
ATOM	2968	CB	MET	C	362	
ATOM	2969	CG	MET	C	362	
ATOM	2970	SD	MET	C	362	
ATOM	2971	CE	MET	C	362	
ATOM	2972	N	ASP	C	363	
ATOM	2973	CA	ASP	C	363	
ATOM	2974	C	ASP	C	363	
ATOM	2975	O	ASP	C	363	
ATOM	2976	CB	ASP	C	363	
ATOM	2977	CG	ASP	C	363	
ATOM	2978	OD1	ASP	C	363	
ATOM	2979	OD2	ASP	C	363	
ATOM	2980	N	ASP	C	364	
ATOM	2981	CA	ASP	C	364	
ATOM	2982	C	ASP	C	364	
ATOM	2983	O	ASP	C	364	
ATOM	2984	CB	ASP	C	364	

ATOM 2985 CG ASP C 364
ATOM 2986 OD1 ASP C 364
ATOM 2987 OD2 ASP C 364
ATOM 2988 N LEU C 365
ATOM 2989 CA LEU C 365
ATOM 2990 C LEU C 365
ATOM 2991 O LEU C 365
ATOM 2992 CB LEU C 365
ATOM 2993 CG LEU C 365
ATOM 2994 CD1 LEU C 365
ATOM 2995 CD2 LEU C 365
ATOM 2996 N GLU C 366
ATOM 2997 CA GLU C 366
ATOM 2998 C GLU C 366
ATOM 2999 O GLU C 366
ATOM 3000 CB GLU C 366
ATOM 3001 CG GLU C 366
ATOM 3002 CD GLU C 366
ATOM 3003 OE1 GLU C 366
ATOM 3004 OE2 GLU C 366
ATOM 3005 N ARG C 367
ATOM 3006 CA ARG C 367
ATOM 3007 C ARG C 367
ATOM 3008 O ARG C 367
ATOM 3009 CB ARG C 367
ATOM 3010 CG ARG C 367
2.072 4.736 -12.231 1.00 0.84
2.996 4.535 -11.428 1.00 0.84
2.276 7.244 -12.122 1.00 0.84
2.273 8.595 -12.850 1.00 0.84
3.316 8.634 -13.972 1.00 0.84
3.661 10.059 -14.414 1.00 0.84
2.580 10.744 -15.135 1.00 0.84
1.239 3.786 -12.641 1.00 0.91
1.082 2.467 -11.993 1.00 0.91
2.382 1.671 -11.853 1.00 0.91
2.493 0.825 -10.977 1.00 0.91
0.083 1.595 -12.750 1.00 0.91
-1.350 1.947 -12.359 1.00 0.91
-2.675 1.146 -13.338 1.00 0.91

7865	-2.231	1.496	-15.020	1.00	0.91
7866	3.346	1.965	-12.728	1.00	0.90
7867	4.645	1.271	-12.697	1.00	0.90
7868	5.586	1.755	-11.585	1.00	0.90
7869	6.527	1.051	-11.222	1.00	0.90
7870	5.328	1.328	-14.065	1.00	0.90
7871	4.503	0.576	-15.113	1.00	0.90
7872	4.481	-0.669	-15.042	1.00	0.90
7873	3.915	1.269	-15.967	1.00	0.90
7874	5.353	2.972	-11.100	1.00	0.86
7875	6.162	3.566	-10.030	1.00	0.86
7876	5.355	4.646	-9.304	1.00	0.86
7877	5.572	5.853	-9.469	1.00	0.86
7878	7.465	4.105	-10.638	1.00	0.86
7879	8.449	4.588	-9.573	1.00	0.86
7880	8.238	4.278	-8.374	1.00	0.86
7881	9.385	5.292	-9.989	1.00	0.86
7882	4.555	4.163	-8.368	1.00	0.83
7883	3.660	5.012	-7.559	1.00	0.83
7884	4.407	5.943	-6.602	1.00	0.83
7885	4.040	7.104	-6.457	1.00	0.83
7886	2.679	4.134	-6.785	1.00	0.83
7887	1.712	3.427	-7.737	1.00	0.83
7888	0.934	2.357	-6.983	1.00	0.83
7889	0.724	4.422	-8.351	1.00	0.83
7890	5.539	5.448	-6.095	1.00	0.76
7891	6.407	6.223	-5.193	1.00	0.76
7892	6.815	7.560	-5.836	1.00	0.76
7893	6.537	8.622	-5.296	1.00	0.76
7894	7.641	5.390	-4.839	1.00	0.76
7895	8.546	6.122	-3.841	1.00	0.76
7896	9.767	5.280	-3.474	1.00	0.76
7897	10.486	4.846	-4.400	1.00	0.76
7898	9.963	5.080	-2.259	1.00	0.76
7899	7.396	7.462	-7.035	1.00	0.68
7900	7.841	8.646	-7.789	1.00	0.68
7901	6.679	9.459	-8.371	1.00	0.68
7902	6.642	10.676	-8.227	1.00	0.68
7903	8.787	8.230	-8.918	1.00	0.68
7904	10.110	7.679	-8.385	1.00	0.68

7905 ATOM 3011 CD ARG C 367
7906 ATOM 3012 NE ARG C 367
7907 ATOM 3013 CZ ARG C 367
7908 ATOM 3014 NH1 ARG C 367
7909 ATOM 3015 NH2 ARG C 367
7910 ATOM 3016 N GLY C 368
7911 ATOM 3017 CA GLY C 368
7912 ATOM 3018 C GLY C 368
7913 ATOM 3019 O GLY C 368
7914 ATOM 3020 N LEU C 369
7915 ATOM 3021 CA LEU C 369
7916 ATOM 3022 C LEU C 369
7917 ATOM 3023 O LEU C 369
7918 ATOM 3024 CB LEU C 369
7919 ATOM 3025 CG LEU C 369
7920 ATOM 3026 CD1 LEU C 369
7921 ATOM 3027 CD2 LEU C 369
7922 ATOM 3028 N LEU C 370
7923 ATOM 3029 CA LEU C 370
7924 ATOM 3030 C LEU C 370
7925 ATOM 3031 O LEU C 370
7926 ATOM 3032 CB LEU C 370
7927 ATOM 3033 CG LEU C 370
7928 ATOM 3034 CD1 LEU C 370
7929 ATOM 3035 CD2 LEU C 370
7930 ATOM 3036 N THR C 371
7931 ATOM 3037 CA THR C 371
7932 ATOM 3038 C THR C 371
7933 ATOM 3039 O THR C 371
7934 ATOM 3040 CB THR C 371
7935 ATOM 3041 OG1 THR C 371
7936 ATOM 3042 CG2 THR C 371
7937 ATOM 3043 N LEU C 372
7938 ATOM 3044 CA LEU C 372
7939 ATOM 3045 C LEU C 372
7940 ATOM 3046 O LEU C 372
7941 ATOM 3047 CB LEU C 372
7942 ATOM 3048 CG LEU C 372
7943 ATOM 3049 CD1 LEU C 372
7944 ATOM 3050 CD2 LEU C 372

ATOM	3051	N	SER	C	373
ATOM	3052	CA	SER	C	373
ATOM	3053	C	SER	C	373
ATOM	3054	O	SER	C	373
ATOM	3055	CB	SER	C	373
ATOM	3056	OG	SER	C	373
ATOM	3057	N	GLU	C	374
ATOM	3058	CA	GLU	C	374
ATOM	3059	C	GLU	C	374
ATOM	3060	O	GLU	C	374
ATOM	3061	CB	GLU	C	374
ATOM	3062	CG	GLU	C	374
ATOM	3063	CD	GLU	C	374
ATOM	3064	OE1	GLU	C	374

10.957	8.759	-7.714	1.00	0.68
12.206	8.152	-7.222	1.00	0.68
12.358	7.477	-6.082	1.00	0.68
11.345	7.324	-5.251	1.00	0.68
13.535	6.962	-5.755	1.00	0.68
5.679	8.721	-8.890	1.00	0.83
4.499	9.317	-9.542	1.00	0.83
3.677	10.238	-8.628	1.00	0.83
3.265	11.319	-9.044	1.00	0.83
3.567	9.852	-7.360	1.00	0.80
2.740	10.589	-6.378	1.00	0.80
3.575	11.384	-5.371	1.00	0.80
3.040	11.841	-4.346	1.00	0.80
1.868	9.603	-5.589	1.00	0.80
1.018	8.650	-6.435	1.00	0.80
0.219	7.745	-5.501	1.00	0.80
0.063	9.397	-7.368	1.00	0.80
4.812	11.699	-5.724	1.00	0.71
5.724	12.394	-4.795	1.00	0.71
5.147	13.718	-4.271	1.00	0.71
4.957	13.897	-3.064	1.00	0.71
7.088	12.651	-5.441	1.00	0.71
8.035	13.353	-4.456	1.00	0.71
8.350	12.448	-3.265	1.00	0.71
9.318	13.792	-5.156	1.00	0.71
4.778	14.579	-5.206	1.00	0.69

7985	4.243	15.927	-4.904	1.00	0.69
7986	2.971	15.866	-4.051	1.00	0.69
7987	2.774	16.671	-3.141	1.00	0.69
7988	3.918	16.686	-6.194	1.00	0.69
7989	2.955	15.945	-6.949	1.00	0.69
7990	5.180	16.939	-7.027	1.00	0.69
7991	2.195	14.810	-4.280	1.00	0.73
7992	0.936	14.604	-3.563	1.00	0.73
7993	1.163	14.141	-2.122	1.00	0.73
7994	0.514	14.628	-1.198	1.00	0.73
7995	0.057	13.652	-4.377	1.00	0.73
7996	-1.444	13.889	-4.155	1.00	0.73
7997	-1.824	15.378	-4.125	1.00	0.73
7998	-2.202	13.239	-5.312	1.00	0.73
7999	2.238	13.380	-1.950	1.00	0.77
8000	2.703	12.914	-0.634	1.00	0.77
8001	3.224	14.062	0.238	1.00	0.77
8002	2.869	14.124	1.412	1.00	0.77
8003	3.793	11.854	-0.796	1.00	0.77
8004	4.228	11.457	0.507	1.00	0.77
8005	3.960	14.999	-0.353	1.00	0.70
8006	4.486	16.163	0.390	1.00	0.70
8007	3.361	17.080	0.879	1.00	0.70
8008	3.354	17.523	2.024	1.00	0.70
8009	5.425	17.003	-0.470	1.00	0.70
8010	6.681	16.235	-0.880	1.00	0.70
8011	7.732	17.168	-1.486	1.00	0.70
8012	7.884	18.295	-0.963	1.00	0.70
8013	ATOM	3065	OE2 GLU C 374		
8014	ATOM	3066	N LEU C 375		
8015	ATOM	3067	CA LEU C 375		
8016	ATOM	3068	C LEU C 375		
8017	ATOM	3069	O LEU C 375		
8018	ATOM	3070	CB LEU C 375		
8019	ATOM	3071	CG LEU C 375		
8020	ATOM	3072	CD1 LEU C 375		
8021	ATOM	3073	CD2 LEU C 375		
8022	ATOM	3074	N HIS C 376		
8023	ATOM	3075	CA HIS C 376		
8024	ATOM	3076	C HIS C 376		

8025 ATOM 3077 O HIS C 376
8026 ATOM 3078 CB HIS C 376
8027 ATOM 3079 CG HIS C 376
8028 ATOM 3080 ND1 HIS C 376
8029 ATOM 3081 CD2 HIS C 376
8030 ATOM 3082 CE1 HIS C 376
8031 ATOM 3083 NE2 HIS C 376
8032 ATOM 3084 N ALA C 377
8033 ATOM 3085 CA ALA C 377
8034 ATOM 3086 C ALA C 377
8035 ATOM 3087 O ALA C 377
8036 ATOM 3088 CB ALA C 377
8037 ATOM 3089 N PHE C 378
8038 ATOM 3090 CA PHE C 378
8039 ATOM 3091 C PHE C 378
8040 ATOM 3092 O PHE C 378
8041 ATOM 3093 CB PHE C 378
8042 ATOM 3094 CG PHE C 378
8043 ATOM 3095 CD1 PHE C 378
8044 ATOM 3096 CD2 PHE C 378
8045 ATOM 3097 CE1 PHE C 378
8046 ATOM 3098 CE2 PHE C 378
8047 ATOM 3099 CZ PHE C 378
8048 ATOM 3100 N LYS C 379
8049 ATOM 3101 CA LYS C 379
8050 ATOM 3102 C LYS C 379
8051 ATOM 3103 O LYS C 379
8052 ATOM 3104 CB LYS C 379
8053 ATOM 3105 CG LYS C 379
8054 ATOM 3106 CD LYS C 379
8055 ATOM 3107 CE LYS C 379
8056 ATOM 3108 NZ LYS C 379
8057 ATOM 3109 N LEU C 380
8058 ATOM 3110 CA LEU C 380
8059 ATOM 3111 C LEU C 380
8060 ATOM 3112 O LEU C 380
8061 ATOM 3113 CB LEU C 380
8062 ATOM 3114 CG LEU C 380
8063 ATOM 3115 CD1 LEU C 380
8064 ATOM 3116 CD2 LEU C 380

8065	ATOM 3117 N ARG C 381				
8066	ATOM 3118 CA ARG C 381				
8067	8.383	16.720	-2.453	1.00	0.70
8068	2.350	17.231	0.025	1.00	0.68
8069	1.182	18.061	0.345	1.00	0.68
8070	0.410	17.473	1.541	1.00	0.68
8071	0.028	18.196	2.459	1.00	0.68
8072	0.345	18.201	-0.936	1.00	0.68
8073	-0.864	19.124	-0.764	1.00	0.68
8074	-1.399	19.634	-2.095	1.00	0.68
8075	-2.008	18.280	-0.250	1.00	0.68
8076	0.255	16.153	1.550	1.00	0.72
8077	-0.447	15.454	2.638	1.00	0.72
8078	0.405	15.346	3.908	1.00	0.72
8079	-0.115	15.519	5.000	1.00	0.72
8080	-0.908	14.070	2.181	1.00	0.72
8081	-2.067	14.169	1.189	1.00	0.72
8082	-1.962	14.309	-0.126	1.00	0.72
8083	-3.362	14.110	1.490	1.00	0.72
8084	-3.185	14.343	-0.643	1.00	0.72
8085	-4.051	14.222	0.358	1.00	0.72
8086	1.713	15.165	3.728	1.00	0.77
8087	2.673	15.044	4.839	1.00	0.77
8088	2.933	16.362	5.577	1.00	0.77
8089	2.769	16.418	6.789	1.00	0.77
8090	3.997	14.477	4.323	1.00	0.77
8091	3.214	17.423	4.820	1.00	0.61
8092	3.617	18.717	5.401	1.00	0.61
8093	2.475	19.703	5.614	1.00	0.61
8094	2.367	20.322	6.667	1.00	0.61
8095	4.701	19.377	4.538	1.00	0.61
8096	6.016	18.596	4.530	1.00	0.61
8097	6.412	17.866	5.649	1.00	0.61
8098	6.837	18.645	3.409	1.00	0.61
8099	7.616	17.187	5.647	1.00	0.61
8100	8.049	17.967	3.409	1.00	0.61
8101	8.440	17.239	4.529	1.00	0.61
8102	1.652	19.830	4.579	1.00	0.65
8103	0.590	20.845	4.558	1.00	0.65
8104	-0.670	20.368	5.288	1.00	0.65

-1.193 21.056 6.159 1.00 0.65
0.289 21.181 3.098 1.00 0.65
-0.757 22.281 2.938 1.00 0.65
-1.137 22.330 1.465 1.00 0.65
-2.066 23.494 1.141 1.00 0.65
-2.351 23.449 -0.297 1.00 0.65
-1.142 19.198 4.877 1.00 0.69
-2.428 18.683 5.361 1.00 0.69
-2.315 17.919 6.672 1.00 0.69
-3.194 18.058 7.528 1.00 0.69
-3.059 17.809 4.288 1.00 0.69
-3.346 18.554 2.986 1.00 0.69
-4.116 17.566 2.121 1.00 0.69
-4.203 19.813 3.164 1.00 0.69
-1.233 17.167 6.818 1.00 0.71
-0.931 16.370 8.028 1.00 0.71
ATOM 3119 C ARG C 381
ATOM 3120 O ARG C 381
ATOM 3121 CB ARG C 381
ATOM 3122 CG ARG C 381
ATOM 3123 CD ARG C 381
ATOM 3124 NE ARG C 381
ATOM 3125 CZ ARG C 381
ATOM 3126 NH1 ARG C 381
ATOM 3127 NH2 ARG C 381
ATOM 3128 N VAL C 382
ATOM 3129 CA VAL C 382
ATOM 3130 C VAL C 382
ATOM 3131 O VAL C 382
ATOM 3132 CB VAL C 382
ATOM 3133 CG1 VAL C 382
ATOM 3134 CG2 VAL C 382
ATOM 3135 N ASP C 383
ATOM 3136 CA ASP C 383
ATOM 3137 C ASP C 383
ATOM 3138 O ASP C 383
ATOM 3139 CB ASP C 383
ATOM 3140 CG ASP C 383
ATOM 3141 OD1 ASP C 383
ATOM 3142 OD2 ASP C 383

ATOM	3143	N	PRO	C	384
ATOM	3144	CA	PRO	C	384
ATOM	3145	C	PRO	C	384
ATOM	3146	O	PRO	C	384
ATOM	3147	CB	PRO	C	384
ATOM	3148	CG	PRO	C	384
ATOM	3149	CD	PRO	C	384
ATOM	3150	N	THR	C	385
ATOM	3151	CA	THR	C	385
ATOM	3152	C	THR	C	385
ATOM	3153	O	THR	C	385
ATOM	3154	CB	THR	C	385
ATOM	3155	OG1	THR	C	385
ATOM	3156	CG2	THR	C	385
ATOM	3157	N	ASN	C	386
ATOM	3158	CA	ASN	C	386
ATOM	3159	C	ASN	C	386
ATOM	3160	O	ASN	C	386
ATOM	3161	CB	ASN	C	386
ATOM	3162	CG	ASN	C	386
ATOM	3163	OD1	ASN	C	386
ATOM	3164	ND2	ASN	C	386
ATOM	3165	N	PHE	C	387
ATOM	3166	CA	PHE	C	387
ATOM	3167	C	PHE	C	387
ATOM	3168	O	PHE	C	387
ATOM	3169	CB	PHE	C	387
ATOM	3170	CG	PHE	C	387
ATOM	3171	CD1	PHE	C	387
ATOM	3172	CD2	PHE	C	387

-1.976	15.262	8.256	1.00	0.71
-2.355	14.933	9.382	1.00	0.71
-0.879	17.301	9.248	1.00	0.71
0.206	18.367	9.164	1.00	0.71
1.541	17.680	9.403	1.00	0.71
2.602	18.689	9.463	1.00	0.71
3.867	18.434	9.788	1.00	0.71
4.269	17.199	10.064	1.00	0.71
4.723	19.433	9.925	1.00	0.71
-2.424	14.660	7.160	1.00	0.83

8185	-3.492	13.642	7.204	1.00	0.83
8186	-2.917	12.330	7.739	1.00	0.83
8187	-2.079	11.708	7.081	1.00	0.83
8188	-4.131	13.442	5.817	1.00	0.83
8189	-5.274	12.419	5.853	1.00	0.83
8190	-4.684	14.760	5.269	1.00	0.83
8191	-3.455	11.883	8.865	1.00	0.88
8192	-3.146	10.547	9.396	1.00	0.88
8193	-3.466	9.501	8.305	1.00	0.88
8194	-4.634	9.364	7.915	1.00	0.88
8195	-3.962	10.281	10.663	1.00	0.88
8196	-3.557	8.992	11.384	1.00	0.88
8197	-3.132	8.022	10.717	1.00	0.88
8198	-3.660	9.024	12.626	1.00	0.88
8199	-2.454	8.742	7.879	1.00	0.90
8200	-2.570	7.749	6.791	1.00	0.90
8201	-3.595	6.636	7.033	1.00	0.90
8202	-4.061	6.012	6.074	1.00	0.90
8203	-1.160	7.190	6.611	1.00	0.90
8204	-0.536	7.345	7.998	1.00	0.90
8205	-1.100	8.681	8.476	1.00	0.90
8206	-3.994	6.419	8.280	1.00	0.89
8207	-5.076	5.466	8.618	1.00	0.89
8208	-6.364	5.795	7.845	1.00	0.89
8209	-7.017	4.906	7.308	1.00	0.89
8210	-5.407	5.473	10.114	1.00	0.89
8211	-5.854	6.777	10.506	1.00	0.89
8212	-4.209	5.021	10.954	1.00	0.89
8213	-6.579	7.091	7.616	1.00	0.89
8214	-7.735	7.607	6.858	1.00	0.89
8215	-7.745	7.201	5.381	1.00	0.89
8216	-8.821	7.128	4.776	1.00	0.89
8217	-7.824	9.128	6.990	1.00	0.89
8218	-8.119	9.525	8.438	1.00	0.89
8219	-7.305	10.106	9.140	1.00	0.89
8220	-9.293	9.155	8.904	1.00	0.89
8221	-6.583	6.833	4.851	1.00	0.87
8222	-6.472	6.339	3.465	1.00	0.87
8223	-7.196	5.008	3.276	1.00	0.87
8224	-7.967	4.850	2.337	1.00	0.87

8225	-5.011	6.190	3.036	1.00	0.87	
8226	-4.251	7.517	3.046	1.00	0.87	
8227	-4.913	8.724	2.822	1.00	0.87	
8228	-2.885	7.515	3.297	1.00	0.87	
8229	ATOM	3173	CE1	PHE	C	387
8230	ATOM	3174	CE2	PHE	C	387
8231	ATOM	3175	CZ	PHE	C	387
8232	ATOM	3176	N	LYS	C	388
8233	ATOM	3177	CA	LYS	C	388
8234	ATOM	3178	C	LYS	C	388
8235	ATOM	3179	O	LYS	C	388
8236	ATOM	3180	CB	LYS	C	388
8237	ATOM	3181	CG	LYS	C	388
8238	ATOM	3182	CD	LYS	C	388
8239	ATOM	3183	CE	LYS	C	388
8240	ATOM	3184	NZ	LYS	C	388
8241	ATOM	3185	N	LEU	C	389
8242	ATOM	3186	CA	LEU	C	389
8243	ATOM	3187	C	LEU	C	389
8244	ATOM	3188	O	LEU	C	389
8245	ATOM	3189	CB	LEU	C	389
8246	ATOM	3190	CG	LEU	C	389
8247	ATOM	3191	CD1	LEU	C	389
8248	ATOM	3192	CD2	LEU	C	389
8249	ATOM	3193	N	LEU	C	390
8250	ATOM	3194	CA	LEU	C	390
8251	ATOM	3195	C	LEU	C	390
8252	ATOM	3196	O	LEU	C	390
8253	ATOM	3197	CB	LEU	C	390
8254	ATOM	3198	CG	LEU	C	390
8255	ATOM	3199	CD1	LEU	C	390
8256	ATOM	3200	CD2	LEU	C	390
8257	ATOM	3201	N	SER	C	391
8258	ATOM	3202	CA	SER	C	391
8259	ATOM	3203	C	SER	C	391
8260	ATOM	3204	O	SER	C	391
8261	ATOM	3205	CB	SER	C	391
8262	ATOM	3206	OG	SER	C	391
8263	ATOM	3207	N	LEU	C	392
8264	ATOM	3208	CA	LEU	C	392

ATOM 3209 C LEU C 392
ATOM 3210 O LEU C 392
ATOM 3211 CB LEU C 392
ATOM 3212 CG LEU C 392
ATOM 3213 CD1 LEU C 392
ATOM 3214 CD2 LEU C 392
ATOM 3215 N ASN C 393
ATOM 3216 CA ASN C 393
ATOM 3217 C ASN C 393
ATOM 3218 O ASN C 393
ATOM 3219 CB ASN C 393
ATOM 3220 CG ASN C 393
ATOM 3221 OD1 ASN C 393
ATOM 3222 ND2 ASN C 393
ATOM 3223 N ILE C 394
ATOM 3224 CA ILE C 394
ATOM 3225 C ILE C 394
ATOM 3226 O ILE C 394
-4.220 9.919 2.869 1.00 0.87
-2.187 8.715 3.333 1.00 0.87
-2.855 9.918 3.127 1.00 0.87
-7.122 4.184 4.319 1.00 0.83
-7.814 2.883 4.345 1.00 0.83
-9.338 3.066 4.338 1.00 0.83
-10.057 2.394 3.595 1.00 0.83
-7.448 2.103 5.602 1.00 0.83
-5.947 1.841 5.755 1.00 0.83
-5.686 0.903 6.940 1.00 0.83
-5.731 1.558 8.330 1.00 0.83
-6.944 2.354 8.574 1.00 0.83
-9.779 4.092 5.060 1.00 0.86
-11.203 4.430 5.219 1.00 0.86
-11.825 4.807 3.875 1.00 0.86
-12.692 4.112 3.391 1.00 0.86
-11.389 5.574 6.219 1.00 0.86
-11.303 5.128 7.685 1.00 0.86
-9.943 4.548 8.080 1.00 0.86
-11.607 6.318 8.589 1.00 0.86
-11.141 5.730 3.181 1.00 0.89
-11.612 6.222 1.881 1.00 0.89

8305	-11.566	5.155	0.781	1.00	0.89
8306	-12.522	5.025	0.016	1.00	0.89
8307	-10.792	7.456	1.489	1.00	0.89
8308	-11.229	8.072	0.151	1.00	0.89
8309	-12.716	8.451	0.136	1.00	0.89
8310	-10.372	9.303	-0.137	1.00	0.89
8311	-10.554	4.298	0.845	1.00	0.90
8312	-10.372	3.192	-0.118	1.00	0.90
8313	-11.528	2.192	-0.047	1.00	0.90
8314	-12.070	1.778	-1.070	1.00	0.90
8315	-9.076	2.432	0.159	1.00	0.90
8316	-7.967	3.309	-0.026	1.00	0.90
8317	-11.986	1.952	1.183	1.00	0.89
8318	-13.132	1.068	1.448	1.00	0.89
8319	-14.432	1.633	0.856	1.00	0.89
8320	-15.170	0.928	0.171	1.00	0.89
8321	-13.276	0.863	2.958	1.00	0.89
8322	-14.368	-0.156	3.308	1.00	0.89
8323	-14.038	-1.550	2.761	1.00	0.89
8324	-14.560	-0.211	4.821	1.00	0.89
8325	-14.604	2.943	1.027	1.00	0.92
8326	-15.749	3.682	0.460	1.00	0.92
8327	-15.722	3.696	-1.074	1.00	0.92
8328	-16.758	3.513	-1.714	1.00	0.92
8329	-15.795	5.124	0.979	1.00	0.92
8330	-16.117	5.180	2.470	1.00	0.92
8331	-17.257	5.081	2.914	1.00	0.92
8332	-15.101	5.279	3.283	1.00	0.92
8333	-14.520	3.764	-1.640	1.00	0.90
8334	-14.321	3.731	-3.105	1.00	0.90
8335	-14.736	2.364	-3.673	1.00	0.90
8336	-15.385	2.314	-4.721	1.00	0.90
8337	ATOM	3227	CB ILE C 394		
8338	ATOM	3228	CG1 ILE C 394		
8339	ATOM	3229	CG2 ILE C 394		
8340	ATOM	3230	CD1 ILE C 394		
8341	ATOM	3231	N LEU C 395		
8342	ATOM	3232	CA LEU C 395		
8343	ATOM	3233	C LEU C 395		
8344	ATOM	3234	O LEU C 395		

8345	ATOM	3235	CB	LEU	C	395
8346	ATOM	3236	CG	LEU	C	395
8347	ATOM	3237	CD1	LEU	C	395
8348	ATOM	3238	CD2	LEU	C	395
8349	ATOM	3239	N	VAL	C	396
8350	ATOM	3240	CA	VAL	C	396
8351	ATOM	3241	C	VAL	C	396
8352	ATOM	3242	O	VAL	C	396
8353	ATOM	3243	CB	VAL	C	396
8354	ATOM	3244	CG1	VAL	C	396
8355	ATOM	3245	CG2	VAL	C	396
8356	ATOM	3246	N	VAL	C	397
8357	ATOM	3247	CA	VAL	C	397
8358	ATOM	3248	C	VAL	C	397
8359	ATOM	3249	O	VAL	C	397
8360	ATOM	3250	CB	VAL	C	397
8361	ATOM	3251	CG1	VAL	C	397
8362	ATOM	3252	CG2	VAL	C	397
8363	ATOM	3253	N	MET	C	398
8364	ATOM	3254	CA	MET	C	398
8365	ATOM	3255	C	MET	C	398
8366	ATOM	3256	O	MET	C	398
8367	ATOM	3257	CB	MET	C	398
8368	ATOM	3258	CG	MET	C	398
8369	ATOM	3259	SD	MET	C	398
8370	ATOM	3260	CE	MET	C	398
8371	ATOM	3261	N	ALA	C	399
8372	ATOM	3262	CA	ALA	C	399
8373	ATOM	3263	C	ALA	C	399
8374	ATOM	3264	O	ALA	C	399
8375	ATOM	3265	CB	ALA	C	399
8376	ATOM	3266	N	ILE	C	400
8377	ATOM	3267	CA	ILE	C	400
8378	ATOM	3268	C	ILE	C	400
8379	ATOM	3269	O	ILE	C	400
8380	ATOM	3270	CB	ILE	C	400
8381	ATOM	3271	CG1	ILE	C	400
8382	ATOM	3272	CG2	ILE	C	400
8383	ATOM	3273	CD1	ILE	C	400
8384	ATOM	3274	N	MET	C	401

8385	ATOM	3275	CA	MET C 401	
8386	ATOM	3276	C	MET C 401	
8387	ATOM	3277	O	MET C 401	
8388	ATOM	3278	CB	MET C 401	
8389	ATOM	3279	CG	MET C 401	
8390	ATOM	3280	SD	MET C 401	
8391	-12.876	4.111	-3.490	1.00	0.90
8392	-12.598	5.571	-3.098	1.00	0.90
8393	-12.613	3.910	-4.995	1.00	0.90
8394	-11.142	6.011	-3.310	1.00	0.90
8395	-14.426	1.297	-2.944	1.00	0.92
8396	-14.795	-0.071	-3.359	1.00	0.92
8397	-16.310	-0.275	-3.344	1.00	0.92
8398	-16.875	-0.805	-4.296	1.00	0.92
8399	-14.133	-1.135	-2.481	1.00	0.92
8400	-12.601	-1.145	-2.553	1.00	0.92
8401	-12.059	-2.240	-1.641	1.00	0.92
8402	-12.083	-1.445	-3.956	1.00	0.92
8403	-16.941	0.307	-2.327	1.00	0.92
8404	-18.407	0.270	-2.154	1.00	0.92
8405	-19.104	1.002	-3.312	1.00	0.92
8406	-20.051	0.482	-3.897	1.00	0.92
8407	-18.793	0.871	-0.787	1.00	0.92
8408	-20.308	1.057	-0.618	1.00	0.92
8409	-18.306	-0.033	0.348	1.00	0.92
8410	-18.588	2.178	-3.652	1.00	0.91
8411	-19.142	3.003	-4.743	1.00	0.91
8412	-18.977	2.299	-6.099	1.00	0.91
8413	-19.895	2.223	-6.904	1.00	0.91
8414	-18.485	4.392	-4.759	1.00	0.91
8415	-18.959	5.245	-5.936	1.00	0.91
8416	-18.828	5.148	-3.480	1.00	0.91
8417	-17.777	1.793	-6.353	1.00	0.87
8418	-17.523	1.046	-7.600	1.00	0.87
8419	-18.418	-0.196	-7.695	1.00	0.87
8420	-18.942	-0.501	-8.761	1.00	0.87
8421	-16.065	0.611	-7.682	1.00	0.87
8422	-15.134	1.817	-7.815	1.00	0.87
8423	-13.374	1.345	-7.942	1.00	0.87
8424	-13.125	0.667	-6.324	1.00	0.87

8425	-18.678	-0.799	-6.535	1.00	0.88	
8426	-19.545	-1.983	-6.404	1.00	0.88	
8427	-21.007	-1.654	-6.722	1.00	0.88	
8428	-21.622	-2.339	-7.542	1.00	0.88	
8429	-19.445	-2.555	-4.988	1.00	0.88	
8430	-21.489	-0.540	-6.181	1.00	0.81	
8431	-22.871	-0.078	-6.428	1.00	0.81	
8432	-23.067	0.312	-7.906	1.00	0.81	
8433	-24.093	0.017	-8.506	1.00	0.81	
8434	-23.293	1.078	-5.494	1.00	0.81	
8435	-22.508	2.361	-5.786	1.00	0.81	
8436	-23.167	0.663	-4.022	1.00	0.81	
8437	-22.966	3.616	-5.063	1.00	0.81	
8438	-22.037	0.956	-8.464	1.00	0.81	
8439	-22.104	1.518	-9.820	1.00	0.81	
8440	-21.836	0.489	-10.913	1.00	0.81	
8441	-22.452	0.546	-11.981	1.00	0.81	
8442	-21.137	2.690	-9.968	1.00	0.81	
8443	-21.590	3.908	-9.177	1.00	0.81	
8444	-20.511	5.354	-9.460	1.00	0.81	
8445	ATOM	3281	CE	MET	C	401
8446	ATOM	3282	N	PHE	C	402
8447	ATOM	3283	CA	PHE	C	402
8448	ATOM	3284	C	PHE	C	402
8449	ATOM	3285	O	PHE	C	402
8450	ATOM	3286	CB	PHE	C	402
8451	ATOM	3287	CG	PHE	C	402
8452	ATOM	3288	CD1	PHE	C	402
8453	ATOM	3289	CD2	PHE	C	402
8454	ATOM	3290	CE1	PHE	C	402
8455	ATOM	3291	CE2	PHE	C	402
8456	ATOM	3292	CZ	PHE	C	402
8457	ATOM	3293	N	PRO	C	403
8458	ATOM	3294	CA	PRO	C	403
8459	ATOM	3295	C	PRO	C	403
8460	ATOM	3296	O	PRO	C	403
8461	ATOM	3297	CB	PRO	C	403
8462	ATOM	3298	CG	PRO	C	403
8463	ATOM	3299	CD	PRO	C	403
8464	ATOM	3300	N	ASP	C	404

ATOM 3301 CA ASP C 404
ATOM 3302 C ASP C 404
ATOM 3303 O ASP C 404
ATOM 3304 CB ASP C 404
ATOM 3305 CG ASP C 404
ATOM 3306 OD1 ASP C 404
ATOM 3307 OD2 ASP C 404
ATOM 3308 N ASP C 405
ATOM 3309 CA ASP C 405
ATOM 3310 C ASP C 405
ATOM 3311 O ASP C 405
ATOM 3312 CB ASP C 405
ATOM 3313 CG ASP C 405
ATOM 3314 OD1 ASP C 405
ATOM 3315 OD2 ASP C 405
ATOM 3316 N PHE C 406
ATOM 3317 CA PHE C 406
ATOM 3318 C PHE C 406
ATOM 3319 O PHE C 406
ATOM 3320 CB PHE C 406
ATOM 3321 CG PHE C 406
ATOM 3322 CD1 PHE C 406
ATOM 3323 CD2 PHE C 406
ATOM 3324 CE1 PHE C 406
ATOM 3325 CE2 PHE C 406
ATOM 3326 CZ PHE C 406
ATOM 3327 N THR C 407
ATOM 3328 CA THR C 407
ATOM 3329 C THR C 407
ATOM 3330 O THR C 407
ATOM 3331 CB THR C 407
ATOM 3332 OG1 THR C 407
ATOM 3333 CG2 THR C 407
ATOM 3334 N PRO C 408
-21.384 6.493 -8.416 1.00 0.81
-20.919 -0.428 -10.653 1.00 0.85
-20.590 -1.401 -11.698 1.00 0.85
-20.373 -2.805 -11.148 1.00 0.85
-19.268 -3.396 -11.228 1.00 0.85
-19.423 -0.932 -12.489 1.00 0.85

8505	-19.249	0.572	-12.751	1.00	0.85
8506	-18.861	1.521	-11.819	1.00	0.85
8507	-19.360	0.876	-14.089	1.00	0.85
8508	-18.650	2.845	-12.241	1.00	0.85
8509	-19.164	2.186	-14.503	1.00	0.85
8510	-18.827	3.159	-13.581	1.00	0.85
8511	-21.512	-3.417	-10.878	1.00	0.84
8512	-21.606	-4.761	-10.295	1.00	0.84
8513	-21.043	-5.860	-11.206	1.00	0.84
8514	-20.282	-6.698	-10.748	1.00	0.84
8515	-23.094	-4.936	-10.002	1.00	0.84
8516	-23.797	-4.072	-11.048	1.00	0.84
8517	-22.873	-2.874	-11.151	1.00	0.84
8518	-21.325	-5.771	-12.513	1.00	0.78
8519	-20.819	-6.774	-13.471	1.00	0.78
8520	-19.293	-6.730	-13.630	1.00	0.78
8521	-18.631	-7.758	-13.629	1.00	0.78
8522	-21.498	-6.641	-14.839	1.00	0.78
8523	-22.997	-6.964	-14.795	1.00	0.78
8524	-23.432	-7.624	-13.825	1.00	0.78
8525	-23.682	-6.525	-15.741	1.00	0.78
8526	-18.771	-5.503	-13.701	1.00	0.84
8527	-17.322	-5.275	-13.856	1.00	0.84
8528	-16.502	-5.592	-12.598	1.00	0.84
8529	-15.390	-6.113	-12.714	1.00	0.84
8530	-17.054	-3.836	-14.302	1.00	0.84
8531	-17.611	-3.557	-15.701	1.00	0.84
8532	-17.209	-4.283	-16.634	1.00	0.84
8533	-18.503	-2.684	-15.777	1.00	0.84
8534	-17.059	-5.289	-11.430	1.00	0.90
8535	-16.352	-5.444	-10.144	1.00	0.90
8536	-16.359	-6.893	-9.642	1.00	0.90
8537	-16.859	-7.220	-8.558	1.00	0.90
8538	-16.939	-4.472	-9.115	1.00	0.90
8539	-15.985	-4.317	-7.929	1.00	0.90
8540	-14.732	-3.763	-8.156	1.00	0.90
8541	-16.364	-4.675	-6.641	1.00	0.90
8542	-13.864	-3.560	-7.098	1.00	0.90
8543	-15.490	-4.467	-5.582	1.00	0.90
8544	-14.237	-3.910	-5.808	1.00	0.90

-15.747 -7.759 -10.428 1.00 0.89
-15.583 -9.181 -10.082 1.00 0.89
-14.684 -9.330 -8.838 1.00 0.89
-13.902 -8.411 -8.537 1.00 0.89
-14.981 -9.972 -11.254 1.00 0.89
-13.617 -9.584 -11.457 1.00 0.89
-15.842 -9.875 -12.520 1.00 0.89
-14.663 -10.510 -8.222 1.00 0.93
ATOM 3335 CA PRO C 408
ATOM 3336 C PRO C 408
ATOM 3337 O PRO C 408
ATOM 3338 CB PRO C 408
ATOM 3339 CG PRO C 408
ATOM 3340 CD PRO C 408
ATOM 3341 N MET C 409
ATOM 3342 CA MET C 409
ATOM 3343 C MET C 409
ATOM 3344 O MET C 409
ATOM 3345 CB MET C 409
ATOM 3346 CG MET C 409
ATOM 3347 SD MET C 409
ATOM 3348 CE MET C 409
ATOM 3349 N ALA C 410
ATOM 3350 CA ALA C 410
ATOM 3351 C ALA C 410
ATOM 3352 O ALA C 410
ATOM 3353 CB ALA C 410
ATOM 3354 N HIS C 411
ATOM 3355 CA HIS C 411
ATOM 3356 C HIS C 411
ATOM 3357 O HIS C 411
ATOM 3358 CB HIS C 411
ATOM 3359 CG HIS C 411
ATOM 3360 ND1 HIS C 411
ATOM 3361 CD2 HIS C 411
ATOM 3362 CE1 HIS C 411
ATOM 3363 NE2 HIS C 411
ATOM 3364 N LEU C 412
ATOM 3365 CA LEU C 412
ATOM 3366 C LEU C 412

ATOM 3367 O LEU C 412
ATOM 3368 CB LEU C 412
ATOM 3369 CG LEU C 412
ATOM 3370 CD1 LEU C 412
ATOM 3371 CD2 LEU C 412
ATOM 3372 N ALA C 413
ATOM 3373 CA ALA C 413
ATOM 3374 C ALA C 413
ATOM 3375 O ALA C 413
ATOM 3376 CB ALA C 413
ATOM 3377 N VAL C 414
ATOM 3378 CA VAL C 414
ATOM 3379 C VAL C 414
ATOM 3380 O VAL C 414
ATOM 3381 CB VAL C 414
ATOM 3382 CG1 VAL C 414
ATOM 3383 CG2 VAL C 414
ATOM 3384 N ASP C 415
ATOM 3385 CA ASP C 415
ATOM 3386 C ASP C 415
ATOM 3387 O ASP C 415
ATOM 3388 CB ASP C 415
-13.729 -10.853 -7.125 1.00 0.93
-12.258 -10.694 -7.553 1.00 0.93
-11.412 -10.240 -6.786 1.00 0.93
-14.010 -12.322 -6.822 1.00 0.93
-15.477 -12.497 -7.209 1.00 0.93
-15.619 -11.619 -8.446 1.00 0.93
-11.988 -10.988 -8.827 1.00 0.82
-10.635 -10.881 -9.420 1.00 0.82
-10.196 -9.425 -9.615 1.00 0.82
-9.047 -9.071 -9.346 1.00 0.82
-10.572 -11.584 -10.776 1.00 0.82
-10.776 -13.089 -10.646 1.00 0.82
-10.659 -13.985 -12.234 1.00 0.82
-10.927 -15.678 -11.766 1.00 0.82
-11.144 -8.599 -10.057 1.00 0.88
-10.934 -7.145 -10.192 1.00 0.88
-10.798 -6.491 -8.812 1.00 0.88
-9.978 -5.596 -8.620 1.00 0.88

-12.101 -6.508 -10.948 1.00 0.88
-11.480 -7.104 -7.846 1.00 0.90
-11.513 -6.667 -6.447 1.00 0.90
-10.131 -6.826 -5.802 1.00 0.90
-9.592 -5.879 -5.231 1.00 0.90
-12.549 -7.501 -5.689 1.00 0.90
-12.866 -6.973 -4.285 1.00 0.90
-13.969 -7.235 -3.602 1.00 0.90
-12.142 -6.153 -3.529 1.00 0.90
-13.942 -6.586 -2.448 1.00 0.90
-12.799 -5.918 -2.405 1.00 0.90
-9.531 -7.996 -5.986 1.00 0.88
-8.206 -8.282 -5.411 1.00 0.88
-7.157 -7.277 -5.919 1.00 0.88
-6.504 -6.600 -5.128 1.00 0.88
-7.830 -9.736 -5.723 1.00 0.88
-6.484 -10.159 -5.121 1.00 0.88
-6.465 -11.674 -4.941 1.00 0.88
-5.323 -9.807 -6.054 1.00 0.88
-7.084 -7.130 -7.241 1.00 0.89
-6.097 -6.233 -7.870 1.00 0.89
-6.313 -4.752 -7.543 1.00 0.89
-5.342 -4.052 -7.233 1.00 0.89
-6.106 -6.428 -9.379 1.00 0.89
-7.567 -4.314 -7.507 1.00 0.91
-7.896 -2.916 -7.149 1.00 0.91
-7.547 -2.632 -5.678 1.00 0.91
-7.064 -1.553 -5.340 1.00 0.91
-9.363 -2.566 -7.465 1.00 0.91
-10.370 -3.323 -6.602 1.00 0.91
-9.620 -1.063 -7.325 1.00 0.91
-7.779 -3.638 -4.837 1.00 0.91
-7.507 -3.536 -3.400 1.00 0.91
-5.999 -3.443 -3.157 1.00 0.91
-5.554 -2.550 -2.443 1.00 0.91
-8.094 -4.732 -2.649 1.00 0.91
ATOM 3389 CG ASP C 415
ATOM 3390 OD1 ASP C 415
ATOM 3391 OD2 ASP C 415
ATOM 3392 N LYS C 416

8665 ATOM 3393 CA LYS C 416
8666 ATOM 3394 C LYS C 416
8667 ATOM 3395 O LYS C 416
8668 ATOM 3396 CB LYS C 416
8669 ATOM 3397 CG LYS C 416
8670 ATOM 3398 CD LYS C 416
8671 ATOM 3399 CE LYS C 416
8672 ATOM 3400 NZ LYS C 416
8673 ATOM 3401 N PHE C 417
8674 ATOM 3402 CA PHE C 417
8675 ATOM 3403 C PHE C 417
8676 ATOM 3404 O PHE C 417
8677 ATOM 3405 CB PHE C 417
8678 ATOM 3406 CG PHE C 417
8679 ATOM 3407 CD1 PHE C 417
8680 ATOM 3408 CD2 PHE C 417
8681 ATOM 3409 CE1 PHE C 417
8682 ATOM 3410 CE2 PHE C 417
8683 ATOM 3411 CZ PHE C 417
8684 ATOM 3412 N LEU C 418
8685 ATOM 3413 CA LEU C 418
8686 ATOM 3414 C LEU C 418
8687 ATOM 3415 O LEU C 418
8688 ATOM 3416 CB LEU C 418
8689 ATOM 3417 CG LEU C 418
8690 ATOM 3418 CD1 LEU C 418
8691 ATOM 3419 CD2 LEU C 418
8692 ATOM 3420 N CYS C 419
8693 ATOM 3421 CA CYS C 419
8694 ATOM 3422 C CYS C 419
8695 ATOM 3423 O CYS C 419
8696 ATOM 3424 CB CYS C 419
8697 ATOM 3425 SG CYS C 419
8698 ATOM 3426 N ALA C 420
8699 ATOM 3427 CA ALA C 420
8700 ATOM 3428 C ALA C 420
8701 ATOM 3429 O ALA C 420
8702 ATOM 3430 CB ALA C 420
8703 ATOM 3431 N LEU C 421
8704 ATOM 3432 CA LEU C 421

ATOM 3433 C LEU C 421
ATOM 3434 O LEU C 421
ATOM 3435 CB LEU C 421
ATOM 3436 CG LEU C 421
ATOM 3437 CD1 LEU C 421
ATOM 3438 CD2 LEU C 421
ATOM 3439 N ALA C 422
ATOM 3440 CA ALA C 422
ATOM 3441 C ALA C 422
ATOM 3442 O ALA C 422
-8.108 -4.470 -1.140 1.00 0.91
-8.391 -3.316 -0.754 1.00 0.91
-7.792 -5.418 -0.399 1.00 0.91
-5.240 -4.256 -3.887 1.00 0.86
-3.767 -4.203 -3.836 1.00 0.86
-3.234 -2.849 -4.327 1.00 0.86
-2.431 -2.209 -3.647 1.00 0.86
-3.174 -5.319 -4.691 1.00 0.86
-3.451 -6.706 -4.105 1.00 0.86
-2.729 -7.793 -4.901 1.00 0.86
-1.208 -7.659 -4.811 1.00 0.86
-0.539 -8.723 -5.562 1.00 0.86
-3.827 -2.358 -5.414 1.00 0.92
-3.486 -1.049 -6.003 1.00 0.92
-3.800 0.139 -5.083 1.00 0.92
-2.970 1.032 -4.930 1.00 0.92
-4.179 -0.906 -7.362 1.00 0.92
-4.177 0.527 -7.906 1.00 0.92
-3.037 1.080 -8.477 1.00 0.92
-5.361 1.251 -7.868 1.00 0.92
-3.087 2.359 -9.022 1.00 0.92
-5.412 2.527 -8.415 1.00 0.92
-4.276 3.080 -8.990 1.00 0.92
-4.959 0.119 -4.435 1.00 0.91
-5.344 1.210 -3.519 1.00 0.91
-4.458 1.265 -2.274 1.00 0.91
-4.030 2.341 -1.857 1.00 0.91
-6.813 1.121 -3.112 1.00 0.91
-7.759 1.512 -4.252 1.00 0.91
-9.204 1.337 -3.790 1.00 0.91

8745	-7.531	2.958	-4.710	1.00	0.91
8746	-4.071	0.081	-1.808	1.00	0.90
8747	-3.093	-0.063	-0.713	1.00	0.90
8748	-1.729	0.500	-1.141	1.00	0.90
8749	-1.079	1.231	-0.395	1.00	0.90
8750	-2.926	-1.539	-0.355	1.00	0.90
8751	-4.467	-2.337	0.221	1.00	0.90
8752	-1.410	0.278	-2.415	1.00	0.91
8753	-0.192	0.802	-3.056	1.00	0.91
8754	-0.216	2.334	-3.158	1.00	0.91
8755	0.785	2.994	-2.872	1.00	0.91
8756	-0.055	0.171	-4.441	1.00	0.91
8757	-1.399	2.883	-3.414	1.00	0.89
8758	-1.591	4.340	-3.475	1.00	0.89
8759	-1.463	4.954	-2.073	1.00	0.89
8760	-0.799	5.981	-1.901	1.00	0.89
8761	-2.946	4.644	-4.117	1.00	0.89
8762	-3.141	6.149	-4.308	1.00	0.89
8763	-4.048	6.373	-5.502	1.00	0.89
8764	-3.808	6.791	-3.092	1.00	0.89
8765	-2.020	4.263	-1.086	1.00	0.89
8766	-1.970	4.684	0.327	1.00	0.89
8767	-0.523	4.740	0.838	1.00	0.89
8768	-0.103	5.736	1.424	1.00	0.89

8769	ATOM	3443	CB	ALA	C	422
8770	ATOM	3444	N	LEU	C	423
8771	ATOM	3445	CA	LEU	C	423
8772	ATOM	3446	C	LEU	C	423
8773	ATOM	3447	O	LEU	C	423
8774	ATOM	3448	CB	LEU	C	423
8775	ATOM	3449	CG	LEU	C	423
8776	ATOM	3450	CD1	LEU	C	423
8777	ATOM	3451	CD2	LEU	C	423
8778	ATOM	3452	N	ALA	C	424
8779	ATOM	3453	CA	ALA	C	424
8780	ATOM	3454	C	ALA	C	424
8781	ATOM	3455	O	ALA	C	424
8782	ATOM	3456	CB	ALA	C	424
8783	ATOM	3457	N	LEU	C	425
8784	ATOM	3458	CA	LEU	C	425

ATOM 3459 C LEU C 425
ATOM 3460 O LEU C 425
ATOM 3461 CB LEU C 425
ATOM 3462 CG LEU C 425
ATOM 3463 CD1 LEU C 425
ATOM 3464 CD2 LEU C 425
ATOM 3465 N SER C 426
ATOM 3466 CA SER C 426
ATOM 3467 C SER C 426
ATOM 3468 O SER C 426
ATOM 3469 CB SER C 426
ATOM 3470 OG SER C 426
ATOM 3471 N GLU C 427
ATOM 3472 CA GLU C 427
ATOM 3473 C GLU C 427
ATOM 3474 O GLU C 427
ATOM 3475 CB GLU C 427
ATOM 3476 CG GLU C 427
ATOM 3477 CD GLU C 427
ATOM 3478 OE1 GLU C 427
ATOM 3479 OE2 GLU C 427
ATOM 3480 N LYS C 428
ATOM 3481 CA LYS C 428
ATOM 3482 C LYS C 428
ATOM 3483 O LYS C 428
ATOM 3484 CB LYS C 428
ATOM 3485 CG LYS C 428
ATOM 3486 CD LYS C 428
ATOM 3487 CE LYS C 428
ATOM 3488 NZ LYS C 428
ATOM 3489 N TYR C 429
ATOM 3490 CA TYR C 429
ATOM 3491 C TYR C 429
ATOM 3492 O TYR C 429
ATOM 3493 CB TYR C 429
ATOM 3494 CG TYR C 429
ATOM 3495 CD1 TYR C 429
ATOM 3496 CD2 TYR C 429
-2.779 3.709 1.183 1.00 0.89
0.259 3.736 0.437 1.00 0.85

8825	1.676	3.649	0.821	1.00	0.85
8826	2.541	4.709	0.124	1.00	0.85
8827	3.449	5.292	0.718	1.00	0.85
8828	2.214	2.247	0.528	1.00	0.85
8829	3.481	1.975	1.347	1.00	0.85
8830	3.173	1.913	2.847	1.00	0.85
8831	4.133	0.678	0.877	1.00	0.85
8832	2.177	5.020	-1.115	1.00	0.86
8833	2.876	6.049	-1.903	1.00	0.86
8834	2.624	7.460	-1.356	1.00	0.86
8835	3.546	8.263	-1.253	1.00	0.86
8836	2.440	5.959	-3.360	1.00	0.86
8837	1.400	7.691	-0.878	1.00	0.82
8838	1.034	8.966	-0.239	1.00	0.82
8839	1.732	9.186	1.101	1.00	0.82
8840	2.076	10.307	1.451	1.00	0.82
8841	-0.477	9.071	-0.028	1.00	0.82
8842	-1.253	9.234	-1.338	1.00	0.82
8843	-2.733	9.387	-0.997	1.00	0.82
8844	-0.783	10.451	-2.144	1.00	0.82
8845	1.953	8.081	1.805	1.00	0.85
8846	2.641	8.085	3.107	1.00	0.85
8847	4.168	8.213	3.008	1.00	0.85
8848	4.840	8.443	4.012	1.00	0.85
8849	2.282	6.826	3.896	1.00	0.85
8850	2.721	5.658	3.202	1.00	0.85
8851	4.684	8.175	1.777	1.00	0.78
8852	6.131	8.147	1.507	1.00	0.78
8853	6.929	9.280	2.173	1.00	0.78
8854	7.993	9.021	2.731	1.00	0.78
8855	6.366	8.177	-0.007	1.00	0.78
8856	7.834	7.936	-0.374	1.00	0.78
8857	8.332	6.575	0.118	1.00	0.78
8858	7.577	5.583	-0.012	1.00	0.78
8859	9.499	6.550	0.547	1.00	0.78
8860	6.385	10.490	2.152	1.00	0.75
8861	7.114	11.679	2.635	1.00	0.75
8862	6.771	12.095	4.071	1.00	0.75
8863	7.213	13.149	4.526	1.00	0.75
8864	6.835	12.858	1.697	1.00	0.75

7.413 12.657 0.292 1.00 0.75
8.945 12.644 0.267 1.00 0.75
9.530 14.005 0.650 1.00 0.75
10.993 13.982 0.568 1.00 0.75
6.081 11.223 4.801 1.00 0.72
5.628 11.550 6.168 1.00 0.72
6.751 11.553 7.210 1.00 0.72
6.820 12.446 8.051 1.00 0.72
4.496 10.617 6.606 1.00 0.72
3.140 11.106 6.094 1.00 0.72
2.788 10.969 4.760 1.00 0.72
2.202 11.579 7.001 1.00 0.72
ATOM 3497 CE1 TYR C 429
ATOM 3498 CE2 TYR C 429
ATOM 3499 CZ TYR C 429
ATOM 3500 OH TYR C 429
ATOM 3501 N ARG C 430
ATOM 3502 CA ARG C 430
ATOM 3503 C ARG C 430
ATOM 3504 O ARG C 430
ATOM 3505 CB ARG C 430
ATOM 3506 CG ARG C 430
ATOM 3507 CD ARG C 430
ATOM 3508 NE ARG C 430
ATOM 3509 CZ ARG C 430
ATOM 3510 NH1 ARG C 430
ATOM 3511 NH2 ARG C 430
ATOM 3512 N VAL D 431
ATOM 3513 CA VAL D 431
ATOM 3514 C VAL D 431
ATOM 3515 O VAL D 431
ATOM 3516 CB VAL D 431
ATOM 3517 CG1 VAL D 431
ATOM 3518 CG2 VAL D 431
ATOM 3519 N GLU D 432
ATOM 3520 CA GLU D 432
ATOM 3521 C GLU D 432
ATOM 3522 O GLU D 432
ATOM 3523 CB GLU D 432
ATOM 3524 CG GLU D 432

ATOM 3525 CD GLU D 432
ATOM 3526 OE1 GLU D 432
ATOM 3527 OE2 GLU D 432
ATOM 3528 N TRP D 433
ATOM 3529 CA TRP D 433
ATOM 3530 C TRP D 433
ATOM 3531 O TRP D 433
ATOM 3532 CB TRP D 433
ATOM 3533 CG TRP D 433
ATOM 3534 CD1 TRP D 433
ATOM 3535 CD2 TRP D 433
ATOM 3536 NE1 TRP D 433
ATOM 3537 CE2 TRP D 433
ATOM 3538 CE3 TRP D 433
ATOM 3539 CZ2 TRP D 433
ATOM 3540 CZ3 TRP D 433
ATOM 3541 CH2 TRP D 433
ATOM 3542 N THR D 434
ATOM 3543 CA THR D 434
ATOM 3544 C THR D 434
ATOM 3545 O THR D 434
ATOM 3546 CB THR D 434
ATOM 3547 OG1 THR D 434
ATOM 3548 CG2 THR D 434
ATOM 3549 N ALA D 435
ATOM 3550 CA ALA D 435
1.502 11.287 4.349 1.00 0.72
0.916 11.896 6.594 1.00 0.72
0.567 11.749 5.260 1.00 0.72
-0.670 12.091 4.824 1.00 0.72
7.636 10.569 7.104 1.00 0.73
8.726 10.343 8.081 1.00 0.73
9.843 9.451 7.509 1.00 0.73
9.552 8.776 6.494 1.00 0.73
8.143 9.703 9.347 1.00 0.73
7.545 8.324 9.066 1.00 0.73
6.957 7.738 10.335 1.00 0.73
6.548 6.350 10.076 1.00 0.73
6.359 5.438 11.025 1.00 0.73
6.511 5.760 12.302 1.00 0.73

8945	6.099	4.178	10.703	1.00	0.73
8946	-24.161	6.342	21.158	1.00	0.59
8947	-25.480	5.959	20.607	1.00	0.59
8948	-25.537	4.440	20.324	1.00	0.59
8949	-24.564	3.841	19.870	1.00	0.59
8950	-25.784	6.748	19.311	1.00	0.59
8951	-27.191	6.470	18.765	1.00	0.59
8952	-25.632	8.261	19.508	1.00	0.59
8953	-26.718	3.883	20.567	1.00	0.70
8954	-27.008	2.466	20.285	1.00	0.70
8955	-27.954	2.390	19.070	1.00	0.70
8956	-27.612	2.851	18.049	1.00	0.70
8957	-27.543	1.813	21.553	1.00	0.70
8958	-27.364	0.292	21.508	1.00	0.70
8959	-25.893	-0.086	21.300	1.00	0.70
8960	-25.101	0.177	22.233	1.00	0.70
8961	-25.595	-0.585	20.196	1.00	0.70
8962	-29.269	2.149	19.262	1.00	0.85
8963	-30.138	2.086	18.075	1.00	0.85
8964	-31.165	3.208	18.046	1.00	0.85
8965	-31.914	3.415	19.009	1.00	0.85
8966	-30.859	0.739	17.999	1.00	0.85
8967	-29.883	-0.427	17.957	1.00	0.85
8968	-29.831	-1.422	18.821	1.00	0.85
8969	-28.878	-0.657	16.989	1.00	0.85
8970	-28.858	-2.277	18.453	1.00	0.85
8971	-28.294	-1.850	17.379	1.00	0.85
8972	-28.470	0.030	15.902	1.00	0.85
8973	-27.235	-2.382	16.577	1.00	0.85
8974	-27.399	-0.481	15.157	1.00	0.85
8975	-26.820	-1.696	15.487	1.00	0.85
8976	-31.185	3.905	16.929	1.00	0.88
8977	-32.255	4.872	16.617	1.00	0.88
8978	-33.312	4.101	15.816	1.00	0.88
8979	-32.983	3.152	15.096	1.00	0.88
8980	-31.741	6.071	15.802	1.00	0.88
8981	-31.452	5.679	14.452	1.00	0.88
8982	-30.519	6.731	16.454	1.00	0.88
8983	-34.549	4.585	15.842	1.00	0.83
8984	-35.656	3.950	15.095	1.00	0.83

8985	ATOM	3551	C	ALA	D	435
8986	ATOM	3552	O	ALA	D	435
8987	ATOM	3553	CB	ALA	D	435
8988	ATOM	3554	N	ALA	D	436
8989	ATOM	3555	CA	ALA	D	436
8990	ATOM	3556	C	ALA	D	436
8991	ATOM	3557	O	ALA	D	436
8992	ATOM	3558	CB	ALA	D	436
8993	ATOM	3559	N	GLU	D	437
8994	ATOM	3560	CA	GLU	D	437
8995	ATOM	3561	C	GLU	D	437
8996	ATOM	3562	O	GLU	D	437
8997	ATOM	3563	CB	GLU	D	437
8998	ATOM	3564	CG	GLU	D	437
8999	ATOM	3565	CD	GLU	D	437
9000	ATOM	3566	OE1	GLU	D	437
9001	ATOM	3567	OE2	GLU	D	437
9002	ATOM	3568	N	ARG	D	438
9003	ATOM	3569	CA	ARG	D	438
9004	ATOM	3570	C	ARG	D	438
9005	ATOM	3571	O	ARG	D	438
9006	ATOM	3572	CB	ARG	D	438
9007	ATOM	3573	CG	ARG	D	438
9008	ATOM	3574	CD	ARG	D	438
9009	ATOM	3575	NE	ARG	D	438
9010	ATOM	3576	CZ	ARG	D	438
9011	ATOM	3577	NH1	ARG	D	438
9012	ATOM	3578	NH2	ARG	D	438
9013	ATOM	3579	N	ARG	D	439
9014	ATOM	3580	CA	ARG	D	439
9015	ATOM	3581	C	ARG	D	439
9016	ATOM	3582	O	ARG	D	439
9017	ATOM	3583	CB	ARG	D	439
9018	ATOM	3584	CG	ARG	D	439
9019	ATOM	3585	CD	ARG	D	439
9020	ATOM	3586	NE	ARG	D	439
9021	ATOM	3587	CZ	ARG	D	439
9022	ATOM	3588	NH1	ARG	D	439
9023	ATOM	3589	NH2	ARG	D	439
9024	ATOM	3590	N	HIS	D	440

ATOM 3591 CA HIS D 440
ATOM 3592 C HIS D 440
ATOM 3593 O HIS D 440
ATOM 3594 CB HIS D 440
ATOM 3595 CG HIS D 440
ATOM 3596 ND1 HIS D 440
ATOM 3597 CD2 HIS D 440
ATOM 3598 CE1 HIS D 440
ATOM 3599 NE2 HIS D 440
ATOM 3600 N VAL D 441
ATOM 3601 CA VAL D 441
ATOM 3602 C VAL D 441
ATOM 3603 O VAL D 441
ATOM 3604 CB VAL D 441
-35.349 3.820 13.594 1.00 0.83
-35.597 2.783 12.999 1.00 0.83
-36.945 4.748 15.276 1.00 0.83
-34.632 4.825 13.065 1.00 0.85
-34.189 4.847 11.663 1.00 0.85
-33.322 3.614 11.361 1.00 0.85
-33.794 2.717 10.707 1.00 0.85
-33.406 6.126 11.366 1.00 0.85
-32.313 3.429 12.230 1.00 0.81
-31.422 2.260 12.098 1.00 0.81
-32.254 0.987 12.181 1.00 0.81
-32.147 0.128 11.222 1.00 0.81
-30.367 2.334 13.214 1.00 0.81
-29.474 3.568 13.071 1.00 0.81
-28.449 3.693 14.198 1.00 0.81
-28.760 3.254 15.326 1.00 0.81
-27.417 4.342 13.930 1.00 0.81
-33.170 0.873 13.069 1.00 0.78
-34.047 -0.313 13.248 1.00 0.78
-34.864 -0.597 11.972 1.00 0.78
-35.013 -1.730 11.535 1.00 0.78
-35.032 -0.146 14.393 1.00 0.78
-34.353 -0.032 15.756 1.00 0.78
-35.431 0.110 16.828 1.00 0.78
-34.808 0.181 18.160 1.00 0.78
-34.502 -0.856 18.944 1.00 0.78

9065	-34.724	-2.107	18.557	1.00	0.78
9066	-33.999	-0.640	20.152	1.00	0.78
9067	-35.341	0.457	11.338	1.00	0.78
9068	-36.112	0.341	10.088	1.00	0.78
9069	-35.248	-0.195	8.938	1.00	0.78
9070	-35.631	-1.162	8.275	1.00	0.78
9071	-36.677	1.713	9.731	1.00	0.78
9072	-37.518	1.650	8.455	1.00	0.78
9073	-38.008	3.043	8.067	1.00	0.78
9074	-38.947	3.537	9.090	1.00	0.78
9075	-40.246	3.240	9.174	1.00	0.78
9076	-40.825	2.435	8.291	1.00	0.78
9077	-40.983	3.759	10.147	1.00	0.78
9078	-34.071	0.394	8.769	1.00	0.87
9079	-33.149	0.060	7.665	1.00	0.87
9080	-32.688	-1.391	7.737	1.00	0.87
9081	-32.828	-2.133	6.755	1.00	0.87
9082	-31.913	0.957	7.687	1.00	0.87
9083	-32.279	2.417	7.432	1.00	0.87
9084	-32.635	3.286	8.364	1.00	0.87
9085	-32.260	3.063	6.272	1.00	0.87
9086	-32.864	4.463	7.803	1.00	0.87
9087	-32.616	4.324	6.508	1.00	0.87
9088	-32.322	-1.830	8.918	1.00	0.89
9089	-31.964	-3.242	9.115	1.00	0.89
9090	-33.101	-4.211	8.764	1.00	0.89
9091	-32.899	-5.340	8.336	1.00	0.89
9092	-31.594	-3.490	10.552	1.00	0.89
9093	ATOM	3605	CG1 VAL D 441		
9094	ATOM	3606	CG2 VAL D 441		
9095	ATOM	3607	N GLU D 442		
9096	ATOM	3608	CA GLU D 442		
9097	ATOM	3609	C GLU D 442		
9098	ATOM	3610	O GLU D 442		
9099	ATOM	3611	CB GLU D 442		
9100	ATOM	3612	CG GLU D 442		
9101	ATOM	3613	CD GLU D 442		
9102	ATOM	3614	OE1 GLU D 442		
9103	ATOM	3615	OE2 GLU D 442		
9104	ATOM	3616	N ALA D 443		

9105	ATOM	3617	CA	ALA	D	443
9106	ATOM	3618	C	ALA	D	443
9107	ATOM	3619	O	ALA	D	443
9108	ATOM	3620	CB	ALA	D	443
9109	ATOM	3621	N	VAL	D	444
9110	ATOM	3622	CA	VAL	D	444
9111	ATOM	3623	C	VAL	D	444
9112	ATOM	3624	O	VAL	D	444
9113	ATOM	3625	CB	VAL	D	444
9114	ATOM	3626	CG1	VAL	D	444
9115	ATOM	3627	CG2	VAL	D	444
9116	ATOM	3628	N	TRP	D	445
9117	ATOM	3629	CA	TRP	D	445
9118	ATOM	3630	C	TRP	D	445
9119	ATOM	3631	O	TRP	D	445
9120	ATOM	3632	CB	TRP	D	445
9121	ATOM	3633	CG	TRP	D	445
9122	ATOM	3634	CD1	TRP	D	445
9123	ATOM	3635	CD2	TRP	D	445
9124	ATOM	3636	NE1	TRP	D	445
9125	ATOM	3637	CE2	TRP	D	445
9126	ATOM	3638	CE3	TRP	D	445
9127	ATOM	3639	CZ2	TRP	D	445
9128	ATOM	3640	CZ3	TRP	D	445
9129	ATOM	3641	CH2	TRP	D	445
9130	ATOM	3642	N	SER	D	446
9131	ATOM	3643	CA	SER	D	446
9132	ATOM	3644	C	SER	D	446
9133	ATOM	3645	O	SER	D	446
9134	ATOM	3646	CB	SER	D	446
9135	ATOM	3647	OG	SER	D	446
9136	ATOM	3648	N	LYS	D	447
9137	ATOM	3649	CA	LYS	D	447
9138	ATOM	3650	C	LYS	D	447
9139	ATOM	3651	O	LYS	D	447
9140	ATOM	3652	CB	LYS	D	447
9141	ATOM	3653	CG	LYS	D	447
9142	ATOM	3654	CD	LYS	D	447
9143	ATOM	3655	CE	LYS	D	447
9144	ATOM	3656	NZ	LYS	D	447

ATOM 3657 N ILE D 448
ATOM 3658 CA ILE D 448
-32.653 -3.246 11.633 1.00 0.89
-31.069 -4.901 10.683 1.00 0.89
-34.251 -3.819 9.284 1.00 0.82
-35.442 -4.646 9.168 1.00 0.82
-35.767 -4.756 7.677 1.00 0.82
-36.002 -5.861 7.199 1.00 0.82
-36.529 -3.981 10.011 1.00 0.82
-37.867 -4.721 9.949 1.00 0.82
-38.569 -4.579 8.590 1.00 0.82
-38.412 -3.509 7.957 1.00 0.82
-39.277 -5.542 8.230 1.00 0.82
-35.628 -3.636 6.972 1.00 0.91
-35.884 -3.562 5.523 1.00 0.91
-34.882 -4.419 4.747 1.00 0.91
-35.274 -5.297 3.963 1.00 0.91
-35.793 -2.106 5.060 1.00 0.91
-33.614 -4.280 5.104 1.00 0.87
-32.520 -5.012 4.443 1.00 0.87
-32.697 -6.527 4.611 1.00 0.87
-32.843 -7.206 3.610 1.00 0.87
-31.116 -4.559 4.877 1.00 0.87
-30.861 -3.089 4.562 1.00 0.87
-30.873 -4.736 6.362 1.00 0.87
-32.978 -6.956 5.854 1.00 0.83
-33.128 -8.384 6.160 1.00 0.83
-34.387 -9.004 5.561 1.00 0.83
-34.331 -10.118 5.036 1.00 0.83
-33.053 -8.702 7.649 1.00 0.83
-33.129 -10.222 7.833 1.00 0.83
-32.116 -11.099 7.743 1.00 0.83
-34.250 -10.940 8.156 1.00 0.83
-32.543 -12.304 8.062 1.00 0.83
-33.869 -12.264 8.314 1.00 0.83
-35.608 -10.598 8.241 1.00 0.83
-34.789 -13.238 8.564 1.00 0.83
-36.542 -11.584 8.475 1.00 0.83
-36.150 -12.907 8.642 1.00 0.83
-35.463 -8.229 5.543 1.00 0.87

9185 -36.738 -8.725 4.998 1.00 0.87
9186 -36.643 -8.979 3.486 1.00 0.87
9187 -37.406 -9.766 2.939 1.00 0.87
9188 -37.911 -7.797 5.320 1.00 0.87
9189 -37.723 -6.513 4.722 1.00 0.87
9190 -35.681 -8.312 2.842 1.00 0.87
9191 -35.419 -8.491 1.402 1.00 0.87
9192 -34.116 -9.248 1.107 1.00 0.87
9193 -33.727 -9.377 -0.062 1.00 0.87
9194 -35.345 -7.124 0.728 1.00 0.87
9195 -36.687 -6.389 0.708 1.00 0.87
9196 -36.572 -5.087 -0.093 1.00 0.87
9197 -36.295 -5.318 -1.586 1.00 0.87
9198 -37.419 -5.999 -2.245 1.00 0.87
9199 -33.461 -9.782 2.132 1.00 0.84
9200 -32.211 -10.542 1.940 1.00 0.84
9201 ATOM 3659 C ILE D 448
9202 ATOM 3660 O ILE D 448
9203 ATOM 3661 CB ILE D 448
9204 ATOM 3662 CG1 ILE D 448
9205 ATOM 3663 CG2 ILE D 448
9206 ATOM 3664 CD1 ILE D 448
9207 ATOM 3665 N ASP D 449
9208 ATOM 3666 CA ASP D 449
9209 ATOM 3667 C ASP D 449
9210 ATOM 3668 O ASP D 449
9211 ATOM 3669 CB ASP D 449
9212 ATOM 3670 CG ASP D 449
9213 ATOM 3671 OD1 ASP D 449
9214 ATOM 3672 OD2 ASP D 449
9215 ATOM 3673 N ILE D 450
9216 ATOM 3674 CA ILE D 450
9217 ATOM 3675 C ILE D 450
9218 ATOM 3676 O ILE D 450
9219 ATOM 3677 CB ILE D 450
9220 ATOM 3678 CG1 ILE D 450
9221 ATOM 3679 CG2 ILE D 450
9222 ATOM 3680 CD1 ILE D 450
9223 ATOM 3681 N ASP D 451
9224 ATOM 3682 CA ASP D 451

ATOM 3683 C ASP D 451
ATOM 3684 O ASP D 451
ATOM 3685 CB ASP D 451
ATOM 3686 CG ASP D 451
ATOM 3687 OD1 ASP D 451
ATOM 3688 OD2 ASP D 451
ATOM 3689 N VAL D 452
ATOM 3690 CA VAL D 452
ATOM 3691 C VAL D 452
ATOM 3692 O VAL D 452
ATOM 3693 CB VAL D 452
ATOM 3694 CG1 VAL D 452
ATOM 3695 CG2 VAL D 452
ATOM 3696 N CYS D 453
ATOM 3697 CA CYS D 453
ATOM 3698 C CYS D 453
ATOM 3699 O CYS D 453
ATOM 3700 CB CYS D 453
ATOM 3701 SG CYS D 453
ATOM 3702 N GLY D 454
ATOM 3703 CA GLY D 454
ATOM 3704 C GLY D 454
ATOM 3705 O GLY D 454
ATOM 3706 N PRO D 455
ATOM 3707 CA PRO D 455
ATOM 3708 C PRO D 455
ATOM 3709 O PRO D 455
ATOM 3710 CB PRO D 455
ATOM 3711 CG PRO D 455
ATOM 3712 CD PRO D 455
-32.514 -11.811 1.140 1.00 0.84
-33.271 -12.686 1.557 1.00 0.84
-31.488 -10.882 3.259 1.00 0.84
-31.089 -9.627 4.041 1.00 0.84
-30.219 -11.726 3.057 1.00 0.84
-30.210 -8.600 3.308 1.00 0.84
-31.749 -11.941 0.066 1.00 0.87
-31.774 -13.162 -0.740 1.00 0.87
-30.528 -13.970 -0.372 1.00 0.87
-29.529 -13.973 -1.082 1.00 0.87

9265	-31.862	-12.796	-2.229	1.00	0.87
9266	-31.782	-14.019	-3.158	1.00	0.87
9267	-31.912	-15.156	-2.645	1.00	0.87
9268	-31.492	-13.807	-4.354	1.00	0.87
9269	-30.672	-14.764	0.678	1.00	0.80
9270	-29.571	-15.562	1.265	1.00	0.80
9271	-28.830	-16.449	0.248	1.00	0.80
9272	-27.606	-16.528	0.255	1.00	0.80
9273	-30.090	-16.419	2.433	1.00	0.80
9274	-31.189	-17.385	1.958	1.00	0.80
9275	-30.596	-15.511	3.563	1.00	0.80
9276	-31.607	-18.457	2.966	1.00	0.80
9277	-29.603	-16.996	-0.694	1.00	0.86
9278	-29.081	-17.926	-1.708	1.00	0.86
9279	-28.157	-17.256	-2.733	1.00	0.86
9280	-27.319	-17.928	-3.323	1.00	0.86
9281	-30.220	-18.708	-2.373	1.00	0.86
9282	-30.821	-19.736	-1.406	1.00	0.86
9283	-31.303	-19.302	-0.338	1.00	0.86
9284	-30.789	-20.935	-1.744	1.00	0.86
9285	-28.322	-15.945	-2.913	1.00	0.85
9286	-27.411	-15.148	-3.756	1.00	0.85
9287	-26.201	-14.646	-2.943	1.00	0.85
9288	-25.074	-15.074	-3.125	1.00	0.85
9289	-28.155	-14.017	-4.505	1.00	0.85
9290	-28.691	-12.892	-3.623	1.00	0.85
9291	-27.248	-13.363	-5.546	1.00	0.85
9292	-26.486	-13.790	-1.967	1.00	0.88
9293	-25.479	-13.053	-1.191	1.00	0.88
9294	-24.516	-13.954	-0.417	1.00	0.88
9295	-23.311	-13.763	-0.519	1.00	0.88
9296	-26.175	-12.084	-0.236	1.00	0.88
9297	-27.636	-12.841	0.550	1.00	0.88
9298	-25.062	-15.016	0.199	1.00	0.93
9299	-24.266	-15.927	1.050	1.00	0.93
9300	-23.002	-16.453	0.340	1.00	0.93
9301	-21.888	-16.050	0.730	1.00	0.93
9302	-23.159	-17.240	-0.728	1.00	0.92
9303	-22.048	-17.836	-1.496	1.00	0.92
9304	-21.101	-16.779	-2.067	1.00	0.92

-19.907 -16.888 -1.849 1.00 0.92
-22.698 -18.588 -2.654 1.00 0.92
-24.071 -18.955 -2.101 1.00 0.92
-24.448 -17.733 -1.273 1.00 0.92
ATOM 3713 N LEU D 456
ATOM 3714 CA LEU D 456
ATOM 3715 C LEU D 456
ATOM 3716 O LEU D 456
ATOM 3717 CB LEU D 456
ATOM 3718 CG LEU D 456
ATOM 3719 CD1 LEU D 456
ATOM 3720 CD2 LEU D 456
ATOM 3721 N ALA D 457
ATOM 3722 CA ALA D 457
ATOM 3723 C ALA D 457
ATOM 3724 O ALA D 457
ATOM 3725 CB ALA D 457
ATOM 3726 N LEU D 458
ATOM 3727 CA LEU D 458
ATOM 3728 C LEU D 458
ATOM 3729 O LEU D 458
ATOM 3730 CB LEU D 458
ATOM 3731 CG LEU D 458
ATOM 3732 CD1 LEU D 458
ATOM 3733 CD2 LEU D 458
ATOM 3734 N GLN D 459
ATOM 3735 CA GLN D 459
ATOM 3736 C GLN D 459
ATOM 3737 O GLN D 459
ATOM 3738 CB GLN D 459
ATOM 3739 CG GLN D 459
ATOM 3740 CD GLN D 459
ATOM 3741 OE1 GLN D 459
ATOM 3742 NE2 GLN D 459
ATOM 3743 N ARG D 460
ATOM 3744 CA ARG D 460
ATOM 3745 C ARG D 460
ATOM 3746 O ARG D 460
ATOM 3747 CB ARG D 460
ATOM 3748 CG ARG D 460

ATOM 3749 CD ARG D 460
ATOM 3750 NE ARG D 460
ATOM 3751 CZ ARG D 460
ATOM 3752 NH1 ARG D 460
ATOM 3753 NH2 ARG D 460
ATOM 3754 N CYS D 461
ATOM 3755 CA CYS D 461
ATOM 3756 C CYS D 461
ATOM 3757 O CYS D 461
ATOM 3758 CB CYS D 461
ATOM 3759 SG CYS D 461
ATOM 3760 N LEU D 462
ATOM 3761 CA LEU D 462
ATOM 3762 C LEU D 462
ATOM 3763 O LEU D 462
ATOM 3764 CB LEU D 462
ATOM 3765 CG LEU D 462
ATOM 3766 CD1 LEU D 462
-21.679 -15.678 -2.565 1.00 0.87
-20.927 -14.566 -3.178 1.00 0.87
-20.027 -13.828 -2.186 1.00 0.87
-18.872 -13.528 -2.494 1.00 0.87
-21.892 -13.575 -3.836 1.00 0.87
-22.365 -13.998 -5.234 1.00 0.87
-23.132 -15.324 -5.271 1.00 0.87
-23.228 -12.883 -5.821 1.00 0.87
-20.532 -13.661 -0.973 1.00 0.91
-19.811 -12.976 0.111 1.00 0.91
-18.628 -13.811 0.615 1.00 0.91
-17.501 -13.322 0.676 1.00 0.91
-20.781 -12.682 1.253 1.00 0.91
-18.885 -15.100 0.840 1.00 0.89
-17.837 -16.018 1.314 1.00 0.89
-16.794 -16.313 0.233 1.00 0.89
-15.588 -16.225 0.494 1.00 0.89
-18.426 -17.328 1.845 1.00 0.89
-17.325 -18.219 2.444 1.00 0.89
-16.647 -17.559 3.650 1.00 0.89
-17.893 -19.584 2.819 1.00 0.89
-17.262 -16.591 -0.973 1.00 0.84

-16.365 -16.842 -2.117 1.00 0.84
-15.497 -15.616 -2.448 1.00 0.84
-14.307 -15.768 -2.714 1.00 0.84
-17.124 -17.337 -3.351 1.00 0.84
-18.029 -16.273 -3.973 1.00 0.84
-18.836 -16.828 -5.135 1.00 0.84
-19.576 -17.796 -5.053 1.00 0.84
-18.681 -16.148 -6.244 1.00 0.84
-16.066 -14.416 -2.296 1.00 0.85
-15.290 -13.176 -2.496 1.00 0.85
-14.233 -13.014 -1.394 1.00 0.85
-13.085 -12.658 -1.675 1.00 0.85
-16.199 -11.947 -2.548 1.00 0.85
-15.377 -10.685 -2.831 1.00 0.85
-16.253 -9.451 -3.018 1.00 0.85
-17.009 -9.582 -4.275 1.00 0.85
-16.804 -8.893 -5.399 1.00 0.85
-15.839 -7.999 -5.513 1.00 0.85
-17.594 -9.074 -6.443 1.00 0.85
-14.610 -13.384 -0.175 1.00 0.92
-13.719 -13.314 0.996 1.00 0.92
-12.521 -14.262 0.865 1.00 0.92
-11.388 -13.855 1.121 1.00 0.92
-14.510 -13.643 2.259 1.00 0.92
-13.528 -13.456 3.792 1.00 0.92
-12.783 -15.478 0.395 1.00 0.88
-11.737 -16.496 0.186 1.00 0.88
-10.674 -16.099 -0.845 1.00 0.88
-9.515 -16.464 -0.716 1.00 0.88
-12.358 -17.837 -0.215 1.00 0.88
-13.155 -18.482 0.924 1.00 0.88
-13.806 -19.769 0.424 1.00 0.88
ATOM 3767 CD2 LEU D 462
ATOM 3768 N ILE D 463
ATOM 3769 CA ILE D 463
ATOM 3770 C ILE D 463
ATOM 3771 O ILE D 463
ATOM 3772 CB ILE D 463
ATOM 3773 CG1 ILE D 463
ATOM 3774 CG2 ILE D 463

9425	ATOM	3775	CD1	ILE	D	463
9426	ATOM	3776	N	VAL	D	464
9427	ATOM	3777	CA	VAL	D	464
9428	ATOM	3778	C	VAL	D	464
9429	ATOM	3779	O	VAL	D	464
9430	ATOM	3780	CB	VAL	D	464
9431	ATOM	3781	CG1	VAL	D	464
9432	ATOM	3782	CG2	VAL	D	464
9433	ATOM	3783	N	TYR	D	465
9434	ATOM	3784	CA	TYR	D	465
9435	ATOM	3785	C	TYR	D	465
9436	ATOM	3786	O	TYR	D	465
9437	ATOM	3787	CB	TYR	D	465
9438	ATOM	3788	CG	TYR	D	465
9439	ATOM	3789	CD1	TYR	D	465
9440	ATOM	3790	CD2	TYR	D	465
9441	ATOM	3791	CE1	TYR	D	465
9442	ATOM	3792	CE2	TYR	D	465
9443	ATOM	3793	CZ	TYR	D	465
9444	ATOM	3794	OH	TYR	D	465
9445	ATOM	3795	N	PRO	D	466
9446	ATOM	3796	CA	PRO	D	466
9447	ATOM	3797	C	PRO	D	466
9448	ATOM	3798	O	PRO	D	466
9449	ATOM	3799	CB	PRO	D	466
9450	ATOM	3800	CG	PRO	D	466
9451	ATOM	3801	CD	PRO	D	466
9452	ATOM	3802	N	TRP	D	467
9453	ATOM	3803	CA	TRP	D	467
9454	ATOM	3804	C	TRP	D	467
9455	ATOM	3805	O	TRP	D	467
9456	ATOM	3806	CB	TRP	D	467
9457	ATOM	3807	CG	TRP	D	467
9458	ATOM	3808	CD1	TRP	D	467
9459	ATOM	3809	CD2	TRP	D	467
9460	ATOM	3810	NE1	TRP	D	467
9461	ATOM	3811	CE2	TRP	D	467
9462	ATOM	3812	CE3	TRP	D	467
9463	ATOM	3813	CZ2	TRP	D	467
9464	ATOM	3814	CZ3	TRP	D	467

ATOM	3815	CH2	TRP	D	467
ATOM	3816	N	THR	D	468
ATOM	3817	CA	THR	D	468
ATOM	3818	C	THR	D	468
ATOM	3819	O	THR	D	468
ATOM	3820	CB	THR	D	468

-12.267	-18.803	2.130	1.00	0.88
-11.100	-15.304	-1.828	1.00	0.84
-10.200	-14.843	-2.901	1.00	0.84
-9.413	-13.599	-2.469	1.00	0.84
-8.195	-13.550	-2.635	1.00	0.84
-10.998	-14.621	-4.199	1.00	0.84
-11.548	-15.975	-4.662	1.00	0.84
-10.147	-13.962	-5.303	1.00	0.84
-12.666	-15.838	-5.692	1.00	0.84
-10.140	-12.583	-2.020	1.00	0.88
-9.535	-11.282	-1.661	1.00	0.88
-8.648	-11.412	-0.412	1.00	0.88
-7.605	-10.758	-0.322	1.00	0.88
-10.620	-10.204	-1.476	1.00	0.88
-10.020	-8.833	-1.144	1.00	0.88
-11.464	-10.051	-2.745	1.00	0.88
-9.100	-12.216	0.539	1.00	0.92
-8.376	-12.452	1.801	1.00	0.92
-8.083	-13.952	1.928	1.00	0.92
-8.548	-14.586	2.884	1.00	0.92
-9.209	-11.926	2.974	1.00	0.92
-9.570	-10.452	2.814	1.00	0.92
-8.604	-9.511	2.477	1.00	0.92
-10.898	-10.079	2.946	1.00	0.92
-8.968	-8.191	2.278	1.00	0.92
-11.265	-8.756	2.756	1.00	0.92
-10.299	-7.814	2.420	1.00	0.92
-10.657	-6.526	2.197	1.00	0.92
-7.175	-14.466	1.096	1.00	0.91
-6.854	-15.904	1.008	1.00	0.91
-6.371	-16.573	2.304	1.00	0.91
-6.360	-17.800	2.387	1.00	0.91
-5.829	-16.019	-0.122	1.00	0.91
-5.146	-14.654	-0.132	1.00	0.91

-6.281 -13.695 0.201 1.00 0.91
-6.024 -15.792 3.326 1.00 0.81
-5.680 -16.354 4.653 1.00 0.81
-6.882 -16.997 5.366 1.00 0.81
-6.734 -17.964 6.117 1.00 0.81
-4.973 -15.331 5.553 1.00 0.81
-5.653 -13.960 5.605 1.00 0.81
-6.367 -13.469 6.620 1.00 0.81
-5.418 -12.923 4.739 1.00 0.81
-6.567 -12.171 6.429 1.00 0.81
-5.994 -11.786 5.274 1.00 0.81
-4.743 -12.840 3.516 1.00 0.81
-5.902 -10.580 4.627 1.00 0.81
-4.647 -11.632 2.858 1.00 0.81
-5.227 -10.492 3.402 1.00 0.81
-8.080 -16.535 5.025 1.00 0.86
-9.358 -17.090 5.529 1.00 0.86
-9.601 -18.554 5.135 1.00 0.86
-10.344 -19.260 5.823 1.00 0.86
-10.559 -16.256 5.073 1.00 0.86
ATOM 3821 OG1 THR D 468
ATOM 3822 CG2 THR D 468
ATOM 3823 N GLN D 469
ATOM 3824 CA GLN D 469
ATOM 3825 C GLN D 469
ATOM 3826 O GLN D 469
ATOM 3827 CB GLN D 469
ATOM 3828 CG GLN D 469
ATOM 3829 CD GLN D 469
ATOM 3830 OE1 GLN D 469
ATOM 3831 NE2 GLN D 469
ATOM 3832 N ARG D 470
ATOM 3833 CA ARG D 470
ATOM 3834 C ARG D 470
ATOM 3835 O ARG D 470
ATOM 3836 CB ARG D 470
ATOM 3837 CG ARG D 470
ATOM 3838 CD ARG D 470
ATOM 3839 NE ARG D 470
ATOM 3840 CZ ARG D 470

ATOM 3841 NH1 ARG D 470
ATOM 3842 NH2 ARG D 470
ATOM 3843 N TYR D 471
ATOM 3844 CA TYR D 471
ATOM 3845 C TYR D 471
ATOM 3846 O TYR D 471
ATOM 3847 CB TYR D 471
ATOM 3848 CG TYR D 471
ATOM 3849 CD1 TYR D 471
ATOM 3850 CD2 TYR D 471
ATOM 3851 CE1 TYR D 471
ATOM 3852 CE2 TYR D 471
ATOM 3853 CZ TYR D 471
ATOM 3854 OH TYR D 471
ATOM 3855 N PHE D 472
ATOM 3856 CA PHE D 472
ATOM 3857 C PHE D 472
ATOM 3858 O PHE D 472
ATOM 3859 CB PHE D 472
ATOM 3860 CG PHE D 472
ATOM 3861 CD1 PHE D 472
ATOM 3862 CD2 PHE D 472
ATOM 3863 CE1 PHE D 472
ATOM 3864 CE2 PHE D 472
ATOM 3865 CZ PHE D 472
ATOM 3866 N GLY D 473
ATOM 3867 CA GLY D 473
ATOM 3868 C GLY D 473
ATOM 3869 O GLY D 473
ATOM 3870 N SER D 474
ATOM 3871 CA SER D 474
ATOM 3872 C SER D 474
ATOM 3873 O SER D 474
ATOM 3874 CB SER D 474
-10.599 -16.215 3.644 1.00 0.86
-10.530 -14.856 5.693 1.00 0.86
-8.912 -19.026 4.101 1.00 0.77
-8.996 -20.431 3.652 1.00 0.77
-8.476 -21.448 4.678 1.00 0.77
-8.694 -22.654 4.524 1.00 0.77

9585	-8.202	-20.603	2.370	1.00	0.77
9586	-8.820	-19.856	1.183	1.00	0.77
9587	-8.000	-20.044	-0.096	1.00	0.77
9588	-8.038	-19.244	-1.017	1.00	0.77
9589	-7.289	-21.152	-0.185	1.00	0.77
9590	-7.877	-20.933	5.748	1.00	0.70
9591	-7.319	-21.724	6.859	1.00	0.70
9592	-8.309	-22.749	7.446	1.00	0.70
9593	-7.896	-23.830	7.850	1.00	0.70
9594	-6.840	-20.757	7.945	1.00	0.70
9595	-6.146	-21.479	9.103	1.00	0.70
9596	-5.557	-20.471	10.084	1.00	0.70
9597	-4.996	-21.195	11.235	1.00	0.70
9598	-5.301	-20.977	12.514	1.00	0.70
9599	-6.126	-20.000	12.868	1.00	0.70
9600	-4.812	-21.776	13.447	1.00	0.70
9601	-9.609	-22.434	7.409	1.00	0.69
9602	-10.635	-23.323	7.983	1.00	0.69
9603	-11.483	-24.088	6.940	1.00	0.69
9604	-12.506	-24.685	7.242	1.00	0.69
9605	-11.521	-22.490	8.926	1.00	0.69
9606	-12.542	-23.342	9.682	1.00	0.69
9607	-12.140	-24.069	10.794	1.00	0.69
9608	-13.852	-23.468	9.206	1.00	0.69
9609	-13.030	-24.920	11.421	1.00	0.69
9610	-14.737	-24.324	9.822	1.00	0.69
9611	-14.329	-25.063	10.925	1.00	0.69
9612	-15.174	-25.999	11.417	1.00	0.69
9613	-10.974	-24.229	5.720	1.00	0.67
9614	-11.768	-24.917	4.682	1.00	0.67
9615	-11.020	-26.087	4.034	1.00	0.67
9616	-11.160	-26.376	2.844	1.00	0.67
9617	-12.292	-23.906	3.657	1.00	0.67
9618	-13.122	-22.813	4.328	1.00	0.67
9619	-14.435	-23.056	4.697	1.00	0.67
9620	-12.533	-21.590	4.610	1.00	0.67
9621	-15.165	-22.075	5.351	1.00	0.67
9622	-13.261	-20.607	5.265	1.00	0.67
9623	-14.580	-20.847	5.632	1.00	0.67
9624	-10.208	-26.756	4.861	1.00	0.62

-9.469 -27.954 4.418 1.00 0.62
-10.479 -29.097 4.369 1.00 0.62
-11.194 -29.249 5.358 1.00 0.62
-10.573 -29.770 3.210 1.00 0.57
-11.551 -30.853 2.907 1.00 0.57
-12.745 -30.380 2.059 1.00 0.57
-13.462 -31.177 1.474 1.00 0.57
-12.072 -31.571 4.166 1.00 0.57
ATOM 3875 OG SER D 474
ATOM 3876 N PHE D 475
ATOM 3877 CA PHE D 475
ATOM 3878 C PHE D 475
ATOM 3879 O PHE D 475
ATOM 3880 CB PHE D 475
ATOM 3881 CG PHE D 475
ATOM 3882 CD1 PHE D 475
ATOM 3883 CD2 PHE D 475
ATOM 3884 CE1 PHE D 475
ATOM 3885 CE2 PHE D 475
ATOM 3886 CZ PHE D 475
ATOM 3887 N GLY D 476
ATOM 3888 CA GLY D 476
ATOM 3889 C GLY D 476
ATOM 3890 O GLY D 476
ATOM 3891 N ASP D 477
ATOM 3892 CA ASP D 477
ATOM 3893 C ASP D 477
ATOM 3894 O ASP D 477
ATOM 3895 CB ASP D 477
ATOM 3896 CG ASP D 477
ATOM 3897 OD1 ASP D 477
ATOM 3898 OD2 ASP D 477
ATOM 3899 N LEU D 478
ATOM 3900 CA LEU D 478
ATOM 3901 C LEU D 478
ATOM 3902 O LEU D 478
ATOM 3903 CB LEU D 478
ATOM 3904 CG LEU D 478
ATOM 3905 CD1 LEU D 478
ATOM 3906 CD2 LEU D 478

ATOM 3907 N SER D 479
ATOM 3908 CA SER D 479
ATOM 3909 C SER D 479
ATOM 3910 O SER D 479
ATOM 3911 CB SER D 479
ATOM 3912 OG SER D 479
ATOM 3913 N THR D 480
ATOM 3914 CA THR D 480
ATOM 3915 C THR D 480
ATOM 3916 O THR D 480
ATOM 3917 CB THR D 480
ATOM 3918 OG1 THR D 480
ATOM 3919 CG2 THR D 480
ATOM 3920 N ASP D 481
ATOM 3921 CA ASP D 481
ATOM 3922 C ASP D 481
ATOM 3923 O ASP D 481
ATOM 3924 CB ASP D 481
ATOM 3925 CG ASP D 481
ATOM 3926 OD1 ASP D 481
ATOM 3927 OD2 ASP D 481
ATOM 3928 N ALA D 482
-13.041 -32.588 3.974 1.00 0.57
-12.884 -29.052 1.922 1.00 0.67
-13.912 -28.467 1.037 1.00 0.67
-13.685 -28.792 -0.444 1.00 0.67
-14.574 -28.645 -1.269 1.00 0.67
-14.011 -26.947 1.232 1.00 0.67
-14.877 -26.557 2.432 1.00 0.67
-14.865 -27.300 3.612 1.00 0.67
-15.787 -25.514 2.293 1.00 0.67
-15.752 -27.010 4.634 1.00 0.67
-16.683 -25.227 3.315 1.00 0.67
-16.664 -25.972 4.490 1.00 0.67
-12.446 -29.220 -0.735 1.00 0.75
-11.974 -29.497 -2.096 1.00 0.75
-10.873 -28.496 -2.449 1.00 0.75
-10.330 -27.798 -1.588 1.00 0.75
-10.574 -28.433 -3.736 1.00 0.74
-9.537 -27.519 -4.242 1.00 0.74

-10.036 -26.066 -4.210 1.00 0.74
-11.018 -25.729 -4.875 1.00 0.74
-9.120 -27.935 -5.658 1.00 0.74
-7.928 -27.128 -6.192 1.00 0.74
-7.446 -26.242 -5.456 1.00 0.74
-7.524 -27.385 -7.340 1.00 0.74
-9.275 -25.233 -3.517 1.00 0.76
-9.538 -23.779 -3.428 1.00 0.76
-8.334 -22.956 -3.911 1.00 0.76
-8.233 -21.752 -3.662 1.00 0.76
-9.840 -23.417 -1.969 1.00 0.76
-10.920 -24.283 -1.313 1.00 0.76
-11.082 -23.852 0.142 1.00 0.76
-12.263 -24.203 -2.048 1.00 0.76
-7.463 -23.593 -4.678 1.00 0.76
-6.199 -22.994 -5.152 1.00 0.76
-6.367 -21.966 -6.278 1.00 0.76
-5.384 -21.403 -6.764 1.00 0.76
-5.202 -24.074 -5.580 1.00 0.76
-4.861 -24.881 -4.450 1.00 0.76
-7.588 -21.795 -6.771 1.00 0.77
-7.858 -20.836 -7.860 1.00 0.77
-9.262 -20.259 -7.698 1.00 0.77
-10.217 -20.981 -7.409 1.00 0.77
-7.645 -21.508 -9.230 1.00 0.77
-6.296 -21.976 -9.301 1.00 0.77
-7.888 -20.573 -10.422 1.00 0.77
-9.381 -19.009 -8.127 1.00 0.80
-10.634 -18.248 -8.013 1.00 0.80
-11.840 -18.982 -8.629 1.00 0.80
-12.854 -19.142 -7.962 1.00 0.80
-10.414 -16.882 -8.659 1.00 0.80
-11.648 -15.971 -8.632 1.00 0.80
-12.784 -16.469 -8.769 1.00 0.80
-11.432 -14.762 -8.443 1.00 0.80
-11.667 -19.483 -9.857 1.00 0.76
ATOM 3929 CA ALA D 482
ATOM 3930 C ALA D 482
ATOM 3931 O ALA D 482
ATOM 3932 CB ALA D 482

9745	ATOM	3933	N	ALA	D	483
9746	ATOM	3934	CA	ALA	D	483
9747	ATOM	3935	C	ALA	D	483
9748	ATOM	3936	O	ALA	D	483
9749	ATOM	3937	CB	ALA	D	483
9750	ATOM	3938	N	ILE	D	484
9751	ATOM	3939	CA	ILE	D	484
9752	ATOM	3940	C	ILE	D	484
9753	ATOM	3941	O	ILE	D	484
9754	ATOM	3942	CB	ILE	D	484
9755	ATOM	3943	CG1	ILE	D	484
9756	ATOM	3944	CG2	ILE	D	484
9757	ATOM	3945	CD1	ILE	D	484
9758	ATOM	3946	N	VAL	D	485
9759	ATOM	3947	CA	VAL	D	485
9760	ATOM	3948	C	VAL	D	485
9761	ATOM	3949	O	VAL	D	485
9762	ATOM	3950	CB	VAL	D	485
9763	ATOM	3951	CG1	VAL	D	485
9764	ATOM	3952	CG2	VAL	D	485
9765	ATOM	3953	N	GLY	D	486
9766	ATOM	3954	CA	GLY	D	486
9767	ATOM	3955	C	GLY	D	486
9768	ATOM	3956	O	GLY	D	486
9769	ATOM	3957	N	ASN	D	487
9770	ATOM	3958	CA	ASN	D	487
9771	ATOM	3959	C	ASN	D	487
9772	ATOM	3960	O	ASN	D	487
9773	ATOM	3961	CB	ASN	D	487
9774	ATOM	3962	CG	ASN	D	487
9775	ATOM	3963	OD1	ASN	D	487
9776	ATOM	3964	ND2	ASN	D	487
9777	ATOM	3965	N	PRO	D	488
9778	ATOM	3966	CA	PRO	D	488
9779	ATOM	3967	C	PRO	D	488
9780	ATOM	3968	O	PRO	D	488
9781	ATOM	3969	CB	PRO	D	488
9782	ATOM	3970	CG	PRO	D	488
9783	ATOM	3971	CD	PRO	D	488
9784	ATOM	3972	N	LYS	D	489

ATOM 3973 CA LYS D 489
ATOM 3974 C LYS D 489
ATOM 3975 O LYS D 489
ATOM 3976 CB LYS D 489
ATOM 3977 CG LYS D 489
ATOM 3978 CD LYS D 489
ATOM 3979 CE LYS D 489
ATOM 3980 NZ LYS D 489
ATOM 3981 N VAL D 490
ATOM 3982 CA VAL D 490
-12.740 -20.201 -10.572 1.00 0.76
-13.226 -21.455 -9.821 1.00 0.76
-14.406 -21.759 -9.764 1.00 0.76
-12.252 -20.598 -11.966 1.00 0.76
-12.282 -22.146 -9.176 1.00 0.78
-12.607 -23.327 -8.358 1.00 0.78
-13.404 -22.950 -7.099 1.00 0.78
-14.359 -23.635 -6.742 1.00 0.78
-11.318 -24.050 -7.965 1.00 0.78
-13.077 -21.789 -6.529 1.00 0.76
-13.740 -21.285 -5.307 1.00 0.76
-15.178 -20.850 -5.613 1.00 0.76
-16.097 -21.214 -4.873 1.00 0.76
-12.947 -20.142 -4.641 1.00 0.76
-11.556 -20.644 -4.218 1.00 0.76
-13.726 -19.559 -3.444 1.00 0.76
-10.656 -19.588 -3.560 1.00 0.76
-15.350 -20.097 -6.693 1.00 0.78
-16.684 -19.630 -7.119 1.00 0.78
-17.658 -20.809 -7.336 1.00 0.78
-18.802 -20.768 -6.932 1.00 0.78
-16.625 -18.728 -8.371 1.00 0.78
-15.754 -17.494 -8.133 1.00 0.78
-16.163 -19.429 -9.650 1.00 0.78
-17.076 -21.898 -7.890 1.00 0.81
-17.826 -23.101 -8.277 1.00 0.81
-18.065 -24.087 -7.128 1.00 0.81
-18.886 -24.993 -7.263 1.00 0.81
-17.312 -23.936 -6.041 1.00 0.84
-17.343 -24.900 -4.936 1.00 0.84

-18.681 -24.859 -4.171 1.00 0.84
-18.965 -23.883 -3.452 1.00 0.84
-16.117 -24.742 -4.037 1.00 0.84
-16.050 -25.857 -2.993 1.00 0.84
-17.006 -26.171 -2.298 1.00 0.84
-14.895 -26.455 -2.863 1.00 0.84
-19.414 -25.968 -4.222 1.00 0.87
-20.743 -26.124 -3.586 1.00 0.87
-20.703 -26.097 -2.052 1.00 0.87
-21.650 -25.614 -1.413 1.00 0.87
-21.255 -27.466 -4.103 1.00 0.87
-19.979 -28.288 -4.289 1.00 0.87
-18.988 -27.258 -4.823 1.00 0.87
-19.621 -26.579 -1.471 1.00 0.79
-19.395 -26.566 -0.010 1.00 0.79
-19.197 -25.137 0.506 1.00 0.79
-19.751 -24.756 1.540 1.00 0.79
-18.168 -27.407 0.341 1.00 0.79
-18.355 -28.890 -0.005 1.00 0.79
-19.450 -29.565 0.828 1.00 0.79
-19.061 -29.638 2.307 1.00 0.79
-20.098 -30.315 3.095 1.00 0.79
-18.545 -24.325 -0.320 1.00 0.84
-18.338 -22.889 -0.043 1.00 0.84
ATOM 3983 C VAL D 490
ATOM 3984 O VAL D 490
ATOM 3985 CB VAL D 490
ATOM 3986 CG1 VAL D 490
ATOM 3987 CG2 VAL D 490
ATOM 3988 N ALA D 491
ATOM 3989 CA ALA D 491
ATOM 3990 C ALA D 491
ATOM 3991 O ALA D 491
ATOM 3992 CB ALA D 491
ATOM 3993 N ALA D 492
ATOM 3994 CA ALA D 492
ATOM 3995 C ALA D 492
ATOM 3996 O ALA D 492
ATOM 3997 CB ALA D 492
ATOM 3998 N HIS D 493

ATOM 3999 CA HIS D 493
ATOM 4000 C HIS D 493
ATOM 4001 O HIS D 493
ATOM 4002 CB HIS D 493
ATOM 4003 CG HIS D 493
ATOM 4004 ND1 HIS D 493
ATOM 4005 CD2 HIS D 493
ATOM 4006 CE1 HIS D 493
ATOM 4007 NE2 HIS D 493
ATOM 4008 N GLY D 494
ATOM 4009 CA GLY D 494
ATOM 4010 C GLY D 494
ATOM 4011 O GLY D 494
ATOM 4012 N VAL D 495
ATOM 4013 CA VAL D 495
ATOM 4014 C VAL D 495
ATOM 4015 O VAL D 495
ATOM 4016 CB VAL D 495
ATOM 4017 CG1 VAL D 495
ATOM 4018 CG2 VAL D 495
ATOM 4019 N VAL D 496
ATOM 4020 CA VAL D 496
ATOM 4021 C VAL D 496
ATOM 4022 O VAL D 496
ATOM 4023 CB VAL D 496
ATOM 4024 CG1 VAL D 496
ATOM 4025 CG2 VAL D 496
ATOM 4026 N ALA D 497
ATOM 4027 CA ALA D 497
ATOM 4028 C ALA D 497
ATOM 4029 O ALA D 497
ATOM 4030 CB ALA D 497
ATOM 4031 N LEU D 498
ATOM 4032 CA LEU D 498
ATOM 4033 C LEU D 498
ATOM 4034 O LEU D 498
ATOM 4035 CB LEU D 498
ATOM 4036 CG LEU D 498
-19.690 -22.163 -0.115 1.00 0.84
-20.076 -21.470 0.825 1.00 0.84

9905	-17.340	-22.275	-1.040	1.00	0.84
9906	-17.159	-20.768	-0.817	1.00	0.84
9907	-15.970	-22.946	-0.926	1.00	0.84
9908	-20.437	-22.469	-1.174	1.00	0.87
9909	-21.766	-21.876	-1.409	1.00	0.87
9910	-22.711	-22.152	-0.233	1.00	0.87
9911	-23.221	-21.222	0.398	1.00	0.87
9912	-22.353	-22.447	-2.703	1.00	0.87
9913	-22.707	-23.415	0.199	1.00	0.85
9914	-23.517	-23.890	1.328	1.00	0.85
9915	-23.081	-23.285	2.674	1.00	0.85
9916	-23.881	-22.849	3.461	1.00	0.85
9917	-23.454	-25.415	1.412	1.00	0.85
9918	-21.762	-23.168	2.897	1.00	0.80
9919	-21.288	-22.565	4.156	1.00	0.80
9920	-21.568	-21.054	4.222	1.00	0.80
9921	-21.813	-20.500	5.295	1.00	0.80
9922	-19.800	-22.857	4.355	1.00	0.80
9923	-19.355	-22.361	5.732	1.00	0.80
9924	-19.606	-22.959	6.891	1.00	0.80
9925	-18.662	-21.254	5.978	1.00	0.80
9926	-19.052	-22.228	7.854	1.00	0.80
9927	-18.467	-21.176	7.289	1.00	0.80
9928	-21.618	-20.442	3.032	1.00	0.90
9929	-21.888	-19.002	2.864	1.00	0.90
9930	-23.314	-18.646	3.297	1.00	0.90
9931	-23.535	-17.625	3.954	1.00	0.90
9932	-24.245	-19.554	3.009	1.00	0.82
9933	-25.657	-19.407	3.420	1.00	0.82
9934	-25.795	-19.601	4.940	1.00	0.82
9935	-26.533	-18.880	5.600	1.00	0.82
9936	-26.593	-20.338	2.617	1.00	0.82
9937	-26.451	-21.830	2.909	1.00	0.82
9938	-28.059	-19.986	2.856	1.00	0.82
9939	-24.973	-20.512	5.465	1.00	0.78
9940	-24.923	-20.833	6.903	1.00	0.78
9941	-24.498	-19.579	7.686	1.00	0.78
9942	-25.210	-19.105	8.571	1.00	0.78
9943	-23.939	-21.995	7.150	1.00	0.78
9944	-23.800	-22.319	8.641	1.00	0.78

-24.379 -23.271 6.431 1.00 0.78
-23.382 -18.998 7.256 1.00 0.83
-22.813 -17.803 7.902 1.00 0.83
-23.778 -16.614 7.849 1.00 0.83
-24.125 -16.042 8.879 1.00 0.83
-21.497 -17.424 7.221 1.00 0.83
-24.353 -16.401 6.668 1.00 0.82
-25.271 -15.277 6.466 1.00 0.82
-26.531 -15.355 7.334 1.00 0.82
-26.862 -14.400 8.040 1.00 0.82
-25.648 -15.222 4.998 1.00 0.82
-26.375 -13.919 4.702 1.00 0.82
ATOM 4037 CD1 LEU D 498
ATOM 4038 CD2 LEU D 498
ATOM 4039 N THR D 499
ATOM 4040 CA THR D 499
ATOM 4041 C THR D 499
ATOM 4042 O THR D 499
ATOM 4043 CB THR D 499
ATOM 4044 OG1 THR D 499
ATOM 4045 CG2 THR D 499
ATOM 4046 N GLY D 500
ATOM 4047 CA GLY D 500
ATOM 4048 C GLY D 500
ATOM 4049 O GLY D 500
ATOM 4050 N LEU D 501
ATOM 4051 CA LEU D 501
ATOM 4052 C LEU D 501
ATOM 4053 O LEU D 501
ATOM 4054 CB LEU D 501
ATOM 4055 CG LEU D 501
ATOM 4056 CD1 LEU D 501
ATOM 4057 CD2 LEU D 501
ATOM 4058 N ARG D 502
ATOM 4059 CA ARG D 502
ATOM 4060 C ARG D 502
ATOM 4061 O ARG D 502
ATOM 4062 CB ARG D 502
ATOM 4063 CG ARG D 502
ATOM 4064 CD ARG D 502

9985	ATOM	4065	NE	ARG D 502		
9986	ATOM	4066	CZ	ARG D 502		
9987	ATOM	4067	NH1	ARG D 502		
9988	ATOM	4068	NH2	ARG D 502		
9989	ATOM	4069	N	THR D 503		
9990	ATOM	4070	CA	THR D 503		
9991	ATOM	4071	C	THR D 503		
9992	ATOM	4072	O	THR D 503		
9993	ATOM	4073	CB	THR D 503		
9994	ATOM	4074	OG1	THR D 503		
9995	ATOM	4075	CG2	THR D 503		
9996	ATOM	4076	N	ALA D 504		
9997	ATOM	4077	CA	ALA D 504		
9998	ATOM	4078	C	ALA D 504		
9999	ATOM	4079	O	ALA D 504		
10000	ATOM	4080	CB	ALA D 504		
10001	ATOM	4081	N	LEU D 505		
10002	ATOM	4082	CA	LEU D 505		
10003	ATOM	4083	C	LEU D 505		
10004	ATOM	4084	O	LEU D 505		
10005	ATOM	4085	CB	LEU D 505		
10006	ATOM	4086	CG	LEU D 505		
10007	ATOM	4087	CD1	LEU D 505		
10008	ATOM	4088	CD2	LEU D 505		
10009	ATOM	4089	N	ASP D 506		
10010	ATOM	4090	CA	ASP D 506		
10011	-25.514	-12.669	4.921	1.00	0.82	
10012	-26.747	-14.030	3.250	1.00	0.82	
10013	-27.111	-16.547	7.395	1.00	0.79	
10014	-28.271	-16.823	8.271	1.00	0.79	
10015	-27.933	-16.642	9.760	1.00	0.79	
10016	-28.795	-16.335	10.573	1.00	0.79	
10017	-28.816	-18.238	8.075	1.00	0.79	
10018	-27.832	-19.205	8.455	1.00	0.79	
10019	-29.344	-18.462	6.655	1.00	0.79	
10020	-26.633	-16.772	10.080	1.00	0.84	
10021	-26.104	-16.547	11.439	1.00	0.84	
10022	-26.404	-15.129	11.957	1.00	0.84	
10023	-26.407	-14.884	13.160	1.00	0.84	
10024	-26.660	-14.208	11.030	1.00	0.86	

10025	-27.015	-12.812	11.350	1.00	0.86
10026	-28.468	-12.619	11.788	1.00	0.86
10027	-28.791	-11.641	12.468	1.00	0.86
10028	-26.704	-11.898	10.162	1.00	0.86
10029	-25.223	-11.937	9.763	1.00	0.86
10030	-24.988	-10.962	8.612	1.00	0.86
10031	-24.289	-11.615	10.935	1.00	0.86
10032	-29.306	-13.601	11.483	1.00	0.74
10033	-30.751	-13.545	11.788	1.00	0.74
10034	-31.031	-13.364	13.285	1.00	0.74
10035	-31.823	-12.503	13.679	1.00	0.74
10036	-31.459	-14.815	11.327	1.00	0.74
10037	-31.417	-14.969	9.807	1.00	0.74
10038	-32.135	-16.245	9.375	1.00	0.74
10039	-31.458	-17.448	9.898	1.00	0.74
10040	-31.904	-18.698	9.775	1.00	0.74
10041	-33.054	-18.954	9.163	1.00	0.74
10042	-31.189	-19.712	10.243	1.00	0.74
10043	-30.231	-14.044	14.101	1.00	0.82
10044	-30.359	-13.989	15.573	1.00	0.82
10045	-30.056	-12.590	16.136	1.00	0.82
10046	-30.714	-12.132	17.064	1.00	0.82
10047	-29.455	-15.027	16.242	1.00	0.82
10048	-28.097	-14.811	15.846	1.00	0.82
10049	-29.882	-16.455	15.886	1.00	0.82
10050	-29.118	-11.892	15.482	1.00	0.87
10051	-28.757	-10.502	15.829	1.00	0.87
10052	-29.904	-9.539	15.531	1.00	0.87
10053	-30.270	-8.723	16.370	1.00	0.87
10054	-27.573	-10.007	14.998	1.00	0.87
10055	-30.522	-9.748	14.373	1.00	0.83
10056	-31.634	-8.906	13.896	1.00	0.83
10057	-32.850	-8.994	14.810	1.00	0.83
10058	-33.384	-7.976	15.235	1.00	0.83
10059	-32.039	-9.351	12.498	1.00	0.83
10060	-30.802	-9.323	11.613	1.00	0.83
10061	-31.125	-9.985	10.314	1.00	0.83
10062	-30.348	-7.921	11.285	1.00	0.83
10063	-33.140	-10.228	15.216	1.00	0.82
10064	-34.262	-10.506	16.129	1.00	0.82

10065	ATOM	4091	C	ASP	D	506
10066	ATOM	4092	O	ASP	D	506
10067	ATOM	4093	CB	ASP	D	506
10068	ATOM	4094	CG	ASP	D	506
10069	ATOM	4095	OD1	ASP	D	506
10070	ATOM	4096	OD2	ASP	D	506
10071	ATOM	4097	N	HIS	D	507
10072	ATOM	4098	CA	HIS	D	507
10073	ATOM	4099	C	HIS	D	507
10074	ATOM	4100	O	HIS	D	507
10075	ATOM	4101	CB	HIS	D	507
10076	ATOM	4102	CG	HIS	D	507
10077	ATOM	4103	ND1	HIS	D	507
10078	ATOM	4104	CD2	HIS	D	507
10079	ATOM	4105	CE1	HIS	D	507
10080	ATOM	4106	NE2	HIS	D	507
10081	ATOM	4107	N	MET	D	508
10082	ATOM	4108	CA	MET	D	508
10083	ATOM	4109	C	MET	D	508
10084	ATOM	4110	O	MET	D	508
10085	ATOM	4111	CB	MET	D	508
10086	ATOM	4112	CG	MET	D	508
10087	ATOM	4113	SD	MET	D	508
10088	ATOM	4114	CE	MET	D	508
10089	ATOM	4115	N	ASP	D	509
10090	ATOM	4116	CA	ASP	D	509
10091	ATOM	4117	C	ASP	D	509
10092	ATOM	4118	O	ASP	D	509
10093	ATOM	4119	CB	ASP	D	509
10094	ATOM	4120	CG	ASP	D	509
10095	ATOM	4121	OD1	ASP	D	509
10096	ATOM	4122	OD2	ASP	D	509
10097	ATOM	4123	N	GLU	D	510
10098	ATOM	4124	CA	GLU	D	510
10099	ATOM	4125	C	GLU	D	510
10100	ATOM	4126	O	GLU	D	510
10101	ATOM	4127	CB	GLU	D	510
10102	ATOM	4128	CG	GLU	D	510
10103	ATOM	4129	CD	GLU	D	510
10104	ATOM	4130	OE1	GLU	D	510

ATOM 4131 OE2 GLU D 510
ATOM 4132 N ILE D 511
ATOM 4133 CA ILE D 511
ATOM 4134 C ILE D 511
ATOM 4135 O ILE D 511
ATOM 4136 CB ILE D 511
ATOM 4137 CG1 ILE D 511
ATOM 4138 CG2 ILE D 511
ATOM 4139 CD1 ILE D 511
ATOM 4140 N LYS D 512
ATOM 4141 CA LYS D 512
ATOM 4142 C LYS D 512
ATOM 4143 O LYS D 512
ATOM 4144 CB LYS D 512
-34.016 -9.989 17.553 1.00 0.82
-34.957 -9.668 18.275 1.00 0.82
-34.582 -12.004 16.139 1.00 0.82
-35.053 -12.521 14.772 1.00 0.82
-35.482 -11.692 13.938 1.00 0.82
-34.994 -13.756 14.599 1.00 0.82
-32.744 -9.921 17.931 1.00 0.84
-32.329 -9.480 19.275 1.00 0.84
-31.313 -8.335 19.189 1.00 0.84
-30.245 -8.383 19.817 1.00 0.84
-31.748 -10.670 20.048 1.00 0.84
-32.753 -11.799 20.248 1.00 0.84
-33.729 -11.814 21.149 1.00 0.84
-32.712 -12.987 19.654 1.00 0.84
-34.292 -13.017 21.118 1.00 0.84
-33.665 -13.741 20.194 1.00 0.84
-31.747 -7.218 18.622 1.00 0.87
-30.865 -6.044 18.448 1.00 0.87
-30.386 -5.398 19.756 1.00 0.87
-29.341 -4.760 19.798 1.00 0.87
-31.504 -4.983 17.557 1.00 0.87
-31.319 -5.331 16.079 1.00 0.87
-31.989 -4.056 14.953 1.00 0.87
-31.007 -2.650 15.416 1.00 0.87
-31.137 -5.622 20.834 1.00 0.85
-30.793 -5.049 22.149 1.00 0.85

10145 -29.949 -5.998 23.018 1.00 0.85
10146 -29.531 -5.630 24.112 1.00 0.85
10147 -32.064 -4.630 22.897 1.00 0.85
10148 -32.925 -3.677 22.064 1.00 0.85
10149 -32.602 -2.470 22.049 1.00 0.85
10150 -33.870 -4.197 21.432 1.00 0.85
10151 -29.788 -7.239 22.558 1.00 0.79
10152 -29.037 -8.267 23.302 1.00 0.79
10153 -28.063 -9.053 22.414 1.00 0.79
10154 -27.772 -10.218 22.661 1.00 0.79
10155 -30.019 -9.242 23.951 1.00 0.79
10156 -30.976 -8.603 24.958 1.00 0.79
10157 -31.849 -9.657 25.643 1.00 0.79
10158 -31.997 -10.769 25.086 1.00 0.79
10159 -32.338 -9.328 26.742 1.00 0.79
10160 -27.455 -8.360 21.452 1.00 0.79
10161 -26.535 -9.002 20.497 1.00 0.79
10162 -25.392 -9.711 21.220 1.00 0.79
10163 -25.082 -10.808 20.806 1.00 0.79
10164 -26.007 -8.003 19.456 1.00 0.79
10165 -27.173 -7.623 18.535 1.00 0.79
10166 -24.809 -8.533 18.642 1.00 0.79
10167 -26.882 -6.395 17.667 1.00 0.79
10168 -24.821 -9.080 22.254 1.00 0.76
10169 -23.758 -9.706 23.055 1.00 0.76
10170 -24.280 -11.061 23.543 1.00 0.76
10171 -24.343 -11.929 22.732 1.00 0.76
10172 -23.235 -8.810 24.176 1.00 0.76
10173 ATOM 4145 CG LYS D 512
10174 ATOM 4146 CD LYS D 512
10175 ATOM 4147 CE LYS D 512
10176 ATOM 4148 NZ LYS D 512
10177 ATOM 4149 N SER D 513
10178 ATOM 4150 CA SER D 513
10179 ATOM 4151 C SER D 513
10180 ATOM 4152 O SER D 513
10181 ATOM 4153 CB SER D 513
10182 ATOM 4154 OG SER D 513
10183 ATOM 4155 N THR D 514
10184 ATOM 4156 CA THR D 514

10185	ATOM	4157	C	THR	D	514
10186	ATOM	4158	O	THR	D	514
10187	ATOM	4159	CB	THR	D	514
10188	ATOM	4160	OG1	THR	D	514
10189	ATOM	4161	CG2	THR	D	514
10190	ATOM	4162	N	TYR	D	515
10191	ATOM	4163	CA	TYR	D	515
10192	ATOM	4164	C	TYR	D	515
10193	ATOM	4165	O	TYR	D	515
10194	ATOM	4166	CB	TYR	D	515
10195	ATOM	4167	CG	TYR	D	515
10196	ATOM	4168	CD1	TYR	D	515
10197	ATOM	4169	CD2	TYR	D	515
10198	ATOM	4170	CE1	TYR	D	515
10199	ATOM	4171	CE2	TYR	D	515
10200	ATOM	4172	CZ	TYR	D	515
10201	ATOM	4173	OH	TYR	D	515
10202	ATOM	4174	N	ALA	D	516
10203	ATOM	4175	CA	ALA	D	516
10204	ATOM	4176	C	ALA	D	516
10205	ATOM	4177	O	ALA	D	516
10206	ATOM	4178	CB	ALA	D	516
10207	ATOM	4179	N	ALA	D	517
10208	ATOM	4180	CA	ALA	D	517
10209	ATOM	4181	C	ALA	D	517
10210	ATOM	4182	O	ALA	D	517
10211	ATOM	4183	CB	ALA	D	517
10212	ATOM	4184	N	LEU	D	518
10213	ATOM	4185	CA	LEU	D	518
10214	ATOM	4186	C	LEU	D	518
10215	ATOM	4187	O	LEU	D	518
10216	ATOM	4188	CB	LEU	D	518
10217	ATOM	4189	CG	LEU	D	518
10218	ATOM	4190	CD1	LEU	D	518
10219	ATOM	4191	CD2	LEU	D	518
10220	ATOM	4192	N	SER	D	519
10221	ATOM	4193	CA	SER	D	519
10222	ATOM	4194	C	SER	D	519
10223	ATOM	4195	O	SER	D	519
10224	ATOM	4196	CB	SER	D	519

ATOM 4197 OG SER D 519
ATOM 4198 N VAL D 520
-22.529 -7.566 23.627 1.00 0.76
-21.268 -7.908 22.833 1.00 0.76
-20.629 -6.625 22.312 1.00 0.76
-19.408 -6.933 21.565 1.00 0.76
-25.162 -11.046 24.564 1.00 0.77
-25.686 -12.313 25.125 1.00 0.77
-26.084 -13.407 24.110 1.00 0.77
-25.579 -14.530 24.220 1.00 0.77
-26.903 -12.025 26.008 1.00 0.77
-27.927 -11.427 25.214 1.00 0.77
-26.738 -13.016 23.015 1.00 0.79
-27.143 -13.921 21.912 1.00 0.79
-25.969 -14.682 21.267 1.00 0.79
-26.082 -15.881 20.996 1.00 0.79
-27.894 -13.140 20.820 1.00 0.79
-28.997 -12.445 21.406 1.00 0.79
-28.437 -14.051 19.713 1.00 0.79
-24.852 -14.005 21.042 1.00 0.77
-23.723 -14.557 20.272 1.00 0.77
-22.627 -15.278 21.061 1.00 0.77
-21.788 -15.945 20.471 1.00 0.77
-23.090 -13.468 19.405 1.00 0.77
-23.925 -13.256 18.144 1.00 0.77
-23.792 -14.130 17.078 1.00 0.77
-24.820 -12.208 18.049 1.00 0.77
-24.556 -13.957 15.931 1.00 0.77
-25.577 -12.023 16.915 1.00 0.77
-25.450 -12.900 15.848 1.00 0.77
-26.204 -12.743 14.730 1.00 0.77
-22.770 -15.302 22.393 1.00 0.76
-21.727 -15.821 23.306 1.00 0.76
-21.324 -17.257 22.993 1.00 0.76
-20.144 -17.578 22.915 1.00 0.76
-22.194 -15.736 24.764 1.00 0.76
-22.343 -18.024 22.605 1.00 0.74
-22.175 -19.439 22.246 1.00 0.74
-21.431 -19.627 20.913 1.00 0.74
-20.559 -20.467 20.759 1.00 0.74

-23.549 -20.101 22.166 1.00 0.74
-21.771 -18.781 19.941 1.00 0.73
-21.105 -18.808 18.632 1.00 0.73
-19.658 -18.310 18.731 1.00 0.73
-18.748 -18.872 18.126 1.00 0.73
-21.932 -17.954 17.672 1.00 0.73
-22.061 -18.604 16.292 1.00 0.73
-22.711 -19.991 16.372 1.00 0.73
-22.902 -17.698 15.393 1.00 0.73
-19.463 -17.381 19.659 1.00 0.75
-18.150 -16.794 19.979 1.00 0.75
-17.185 -17.838 20.556 1.00 0.75
-16.124 -18.096 19.971 1.00 0.75
-18.382 -15.659 20.973 1.00 0.75
-17.158 -15.019 21.333 1.00 0.75
-17.664 -18.591 21.540 1.00 0.68
ATOM 4199 CA VAL D 520
ATOM 4200 C VAL D 520
ATOM 4201 O VAL D 520
ATOM 4202 CB VAL D 520
ATOM 4203 CG1 VAL D 520
ATOM 4204 CG2 VAL D 520
ATOM 4205 N LEU D 521
ATOM 4206 CA LEU D 521
ATOM 4207 C LEU D 521
ATOM 4208 O LEU D 521
ATOM 4209 CB LEU D 521
ATOM 4210 CG LEU D 521
ATOM 4211 CD1 LEU D 521
ATOM 4212 CD2 LEU D 521
ATOM 4213 N HIS D 522
ATOM 4214 CA HIS D 522
ATOM 4215 C HIS D 522
ATOM 4216 O HIS D 522
ATOM 4217 CB HIS D 522
ATOM 4218 CG HIS D 522
ATOM 4219 ND1 HIS D 522
ATOM 4220 CD2 HIS D 522
ATOM 4221 CE1 HIS D 522
ATOM 4222 NE2 HIS D 522

ATOM 4223 N SER D 523
ATOM 4224 CA SER D 523
ATOM 4225 C SER D 523
ATOM 4226 O SER D 523
ATOM 4227 CB SER D 523
ATOM 4228 OG SER D 523
ATOM 4229 N GLU D 524
ATOM 4230 CA GLU D 524
ATOM 4231 C GLU D 524
ATOM 4232 O GLU D 524
ATOM 4233 CB GLU D 524
ATOM 4234 CG GLU D 524
ATOM 4235 CD GLU D 524
ATOM 4236 OE1 GLU D 524
ATOM 4237 OE2 GLU D 524
ATOM 4238 N LYS D 525
ATOM 4239 CA LYS D 525
ATOM 4240 C LYS D 525
ATOM 4241 O LYS D 525
ATOM 4242 CB LYS D 525
ATOM 4243 CG LYS D 525
ATOM 4244 CD LYS D 525
ATOM 4245 CE LYS D 525
ATOM 4246 NZ LYS D 525
ATOM 4247 N LEU D 526
ATOM 4248 CA LEU D 526
ATOM 4249 C LEU D 526
ATOM 4250 O LEU D 526
ATOM 4251 CB LEU D 526
ATOM 4252 CG LEU D 526
-16.890 -19.677 22.185 1.00 0.68
-16.510 -20.793 21.193 1.00 0.68
-15.415 -21.344 21.249 1.00 0.68
-17.611 -20.285 23.407 1.00 0.68
-17.891 -19.250 24.493 1.00 0.68
-18.923 -21.002 23.089 1.00 0.68
-17.439 -21.057 20.269 1.00 0.69
-17.284 -22.103 19.247 1.00 0.69
-16.122 -21.800 18.297 1.00 0.69
-15.199 -22.604 18.150 1.00 0.69

10345	-18.583	-22.248	18.443	1.00	0.69
10346	-18.457	-23.335	17.369	1.00	0.69
10347	-18.371	-24.726	17.997	1.00	0.69
10348	-19.615	-23.249	16.379	1.00	0.69
10349	-16.169	-20.603	17.720	1.00	0.72
10350	-15.140	-20.150	16.772	1.00	0.72
10351	-13.768	-20.036	17.442	1.00	0.72
10352	-12.757	-20.337	16.830	1.00	0.72
10353	-15.536	-18.805	16.172	1.00	0.72
10354	-16.715	-18.940	15.210	1.00	0.72
10355	-17.996	-19.008	15.550	1.00	0.72
10356	-16.649	-18.943	13.883	1.00	0.72
10357	-18.725	-19.055	14.443	1.00	0.72
10358	-17.890	-19.013	13.411	1.00	0.72
10359	-13.792	-19.680	18.727	1.00	0.76
10360	-12.585	-19.561	19.560	1.00	0.76
10361	-11.923	-20.916	19.843	1.00	0.76
10362	-10.853	-21.224	19.310	1.00	0.76
10363	-12.951	-18.844	20.866	1.00	0.76
10364	-11.784	-18.663	21.669	1.00	0.76
10365	-12.629	-21.745	20.600	1.00	0.69
10366	-12.073	-22.994	21.142	1.00	0.69
10367	-12.108	-24.206	20.210	1.00	0.69
10368	-11.257	-25.075	20.341	1.00	0.69
10369	-12.774	-23.335	22.451	1.00	0.69
10370	-12.484	-22.276	23.519	1.00	0.69
10371	-13.293	-22.478	24.804	1.00	0.69
10372	-14.077	-23.452	24.880	1.00	0.69
10373	-13.141	-21.606	25.686	1.00	0.69
10374	-13.116	-24.296	19.350	1.00	0.69
10375	-13.223	-25.470	18.464	1.00	0.69
10376	-12.689	-25.192	17.058	1.00	0.69
10377	-11.933	-25.980	16.503	1.00	0.69
10378	-14.666	-25.976	18.390	1.00	0.69
10379	-15.211	-26.227	19.796	1.00	0.69
10380	-16.376	-27.206	19.769	1.00	0.69
10381	-16.987	-27.323	21.163	1.00	0.69
10382	-18.067	-28.313	21.140	1.00	0.69
10383	-13.036	-24.013	16.550	1.00	0.71
10384	-12.648	-23.630	15.184	1.00	0.71

-11.284 -22.960 15.105 1.00 0.71
-10.608 -23.071 14.083 1.00 0.71
-13.738 -22.767 14.538 1.00 0.71
-15.120 -23.434 14.585 1.00 0.71
ATOM 4253 CD1 LEU D 526
ATOM 4254 CD2 LEU D 526
ATOM 4255 N HIS D 527
ATOM 4256 CA HIS D 527
ATOM 4257 C HIS D 527
ATOM 4258 O HIS D 527
ATOM 4259 CB HIS D 527
ATOM 4260 CG HIS D 527
ATOM 4261 ND1 HIS D 527
ATOM 4262 CD2 HIS D 527
ATOM 4263 CE1 HIS D 527
ATOM 4264 NE2 HIS D 527
ATOM 4265 N VAL D 528
ATOM 4266 CA VAL D 528
ATOM 4267 C VAL D 528
ATOM 4268 O VAL D 528
ATOM 4269 CB VAL D 528
ATOM 4270 CG1 VAL D 528
ATOM 4271 CG2 VAL D 528
ATOM 4272 N ASP D 529
ATOM 4273 CA ASP D 529
ATOM 4274 C ASP D 529
ATOM 4275 O ASP D 529
ATOM 4276 CB ASP D 529
ATOM 4277 CG ASP D 529
ATOM 4278 OD1 ASP D 529
ATOM 4279 OD2 ASP D 529
ATOM 4280 N PRO D 530
ATOM 4281 CA PRO D 530
ATOM 4282 C PRO D 530
ATOM 4283 O PRO D 530
ATOM 4284 CB PRO D 530
ATOM 4285 CG PRO D 530
ATOM 4286 CD PRO D 530
ATOM 4287 N ASP D 531
ATOM 4288 CA ASP D 531

ATOM	4289	C	ASP	D	531
ATOM	4290	O	ASP	D	531
ATOM	4291	CB	ASP	D	531
ATOM	4292	CG	ASP	D	531
ATOM	4293	OD1	ASP	D	531
ATOM	4294	OD2	ASP	D	531
ATOM	4295	N	ASN	D	532
ATOM	4296	CA	ASN	D	532
ATOM	4297	C	ASN	D	532
ATOM	4298	O	ASN	D	532
ATOM	4299	CB	ASN	D	532
ATOM	4300	CG	ASN	D	532
ATOM	4301	OD1	ASN	D	532
ATOM	4302	ND2	ASN	D	532
ATOM	4303	N	PHE	D	533
ATOM	4304	CA	PHE	D	533
ATOM	4305	C	PHE	D	533
ATOM	4306	O	PHE	D	533

-16.134	-22.642	13.765	1.00	0.71
-15.088	-24.900	14.154	1.00	0.71
-10.920	-22.262	16.177	1.00	0.73
-9.633	-21.543	16.286	1.00	0.73
-9.534	-20.438	15.223	1.00	0.73
-8.467	-20.141	14.685	1.00	0.73
-8.468	-22.533	16.136	1.00	0.73
-8.550	-23.625	17.195	1.00	0.73
-8.586	-23.397	18.499	1.00	0.73
-8.631	-24.935	16.984	1.00	0.73
-8.670	-24.572	19.109	1.00	0.73
-8.691	-25.519	18.176	1.00	0.73
-10.655	-19.773	14.971	1.00	0.81
-10.723	-18.736	13.928	1.00	0.81
-10.208	-17.415	14.503	1.00	0.81
-10.665	-16.942	15.540	1.00	0.81
-12.145	-18.603	13.352	1.00	0.81
-12.239	-17.510	12.278	1.00	0.81
-12.588	-19.922	12.713	1.00	0.81
-9.275	-16.839	13.751	1.00	0.86
-8.777	-15.479	13.988	1.00	0.86
-9.960	-14.504	13.817	1.00	0.86

-10.399 -14.288 12.678 1.00 0.86
-7.645 -15.170 12.997 1.00 0.86
-7.000 -13.791 13.209 1.00 0.86
-7.588 -12.959 13.938 1.00 0.86
-5.979 -13.530 12.547 1.00 0.86
-10.376 -13.843 14.897 1.00 0.87
-11.512 -12.900 14.898 1.00 0.87
-11.367 -11.707 13.939 1.00 0.87
-12.358 -11.064 13.610 1.00 0.87
-11.663 -12.448 16.348 1.00 0.87
-10.244 -12.572 16.887 1.00 0.87
-9.711 -13.838 16.217 1.00 0.87
-10.155 -11.450 13.438 1.00 0.90
-9.956 -10.418 12.400 1.00 0.90
-10.623 -10.809 11.070 1.00 0.90
-11.047 -9.943 10.306 1.00 0.90
-8.473 -10.116 12.177 1.00 0.90
-8.331 -8.929 11.217 1.00 0.90
-8.557 -7.797 11.685 1.00 0.90
-7.937 -9.173 10.062 1.00 0.90
-10.736 -12.114 10.842 1.00 0.90
-11.450 -12.664 9.676 1.00 0.90
-12.950 -12.353 9.703 1.00 0.90
-13.556 -12.164 8.650 1.00 0.90
-11.227 -14.172 9.566 1.00 0.90
-9.773 -14.501 9.216 1.00 0.90
-9.019 -13.705 8.674 1.00 0.90
-9.352 -15.688 9.589 1.00 0.90
-13.484 -12.148 10.910 1.00 0.86
-14.880 -11.703 11.087 1.00 0.86
-15.133 -10.347 10.432 1.00 0.86
-16.051 -10.202 9.632 1.00 0.86
ATOM 4307 CB PHE D 533
ATOM 4308 CG PHE D 533
ATOM 4309 CD1 PHE D 533
ATOM 4310 CD2 PHE D 533
ATOM 4311 CE1 PHE D 533
ATOM 4312 CE2 PHE D 533
ATOM 4313 CZ PHE D 533
ATOM 4314 N ARG D 534

ATOM 4315 CA ARG D 534
ATOM 4316 C ARG D 534
ATOM 4317 O ARG D 534
ATOM 4318 CB ARG D 534
ATOM 4319 CG ARG D 534
ATOM 4320 CD ARG D 534
ATOM 4321 NE ARG D 534
ATOM 4322 CZ ARG D 534
ATOM 4323 NH1 ARG D 534
ATOM 4324 NH2 ARG D 534
ATOM 4325 N LEU D 535
ATOM 4326 CA LEU D 535
ATOM 4327 C LEU D 535
ATOM 4328 O LEU D 535
ATOM 4329 CB LEU D 535
ATOM 4330 CG LEU D 535
ATOM 4331 CD1 LEU D 535
ATOM 4332 CD2 LEU D 535
ATOM 4333 N LEU D 536
ATOM 4334 CA LEU D 536
ATOM 4335 C LEU D 536
ATOM 4336 O LEU D 536
ATOM 4337 CB LEU D 536
ATOM 4338 CG LEU D 536
ATOM 4339 CD1 LEU D 536
ATOM 4340 CD2 LEU D 536
ATOM 4341 N CYS D 537
ATOM 4342 CA CYS D 537
ATOM 4343 C CYS D 537
ATOM 4344 O CYS D 537
ATOM 4345 CB CYS D 537
ATOM 4346 SG CYS D 537
ATOM 4347 N GLU D 538
ATOM 4348 CA GLU D 538
ATOM 4349 C GLU D 538
ATOM 4350 O GLU D 538
ATOM 4351 CB GLU D 538
ATOM 4352 CG GLU D 538
ATOM 4353 CD GLU D 538
ATOM 4354 OE1 GLU D 538

10545	ATOM	4355	OE2 GLU D 538		
10546	ATOM	4356	N CYS D 539		
10547	ATOM	4357	CA CYS D 539		
10548	ATOM	4358	C CYS D 539		
10549	ATOM	4359	O CYS D 539		
10550	ATOM	4360	CB CYS D 539		
10551	-15.249	-11.616	12.572	1.00	0.86
10552	-15.170	-12.958	13.298	1.00	0.86
10553	-15.334	-14.153	12.599	1.00	0.86
10554	-15.045	-12.978	14.682	1.00	0.86
10555	-15.378	-15.356	13.273	1.00	0.86
10556	-15.099	-14.187	15.361	1.00	0.86
10557	-15.264	-15.372	14.656	1.00	0.86
10558	-14.143	-9.471	10.598	1.00	0.81
10559	-14.172	-8.117	10.025	1.00	0.81
10560	-14.040	-8.145	8.493	1.00	0.81
10561	-14.774	-7.454	7.785	1.00	0.81
10562	-13.041	-7.305	10.657	1.00	0.81
10563	-13.054	-5.858	10.166	1.00	0.81
10564	-11.953	-5.057	10.853	1.00	0.81
10565	-11.948	-3.683	10.324	1.00	0.81
10566	-11.361	-3.272	9.196	1.00	0.81
10567	-10.698	-4.120	8.417	1.00	0.81
10568	-11.407	-1.991	8.853	1.00	0.81
10569	-13.158	-9.010	8.008	1.00	0.88
10570	-12.888	-9.131	6.562	1.00	0.88
10571	-14.079	-9.673	5.781	1.00	0.88
10572	-14.430	-9.148	4.719	1.00	0.88
10573	-11.664	-10.019	6.330	1.00	0.88
10574	-10.399	-9.438	6.971	1.00	0.88
10575	-9.222	-10.360	6.674	1.00	0.88
10576	-10.093	-8.014	6.490	1.00	0.88
10577	-14.774	-10.614	6.415	1.00	0.89
10578	-15.983	-11.200	5.859	1.00	0.89
10579	-17.141	-10.184	5.790	1.00	0.89
10580	-17.657	-9.937	4.784	1.00	0.89
10581	-16.378	-12.473	6.608	1.00	0.89
10582	-17.612	-13.157	6.002	1.00	0.89
10583	-17.430	-13.490	4.515	1.00	0.89
10584	-17.932	-14.433	6.773	1.00	0.89

-17.261 -9.393 6.923 1.00 0.89
-18.294 -8.351 6.960 1.00 0.89
-18.135 -7.293 5.875 1.00 0.89
-19.115 -6.870 5.264 1.00 0.89
-18.236 -7.640 8.312 1.00 0.89
-18.634 -8.799 9.654 1.00 0.89
-16.879 -6.993 5.543 1.00 0.87
-16.568 -6.059 4.451 1.00 0.87
-17.007 -6.626 3.094 1.00 0.87
-17.597 -5.912 2.289 1.00 0.87
-15.076 -5.758 4.383 1.00 0.87
-14.549 -5.074 5.646 1.00 0.87
-13.078 -4.676 5.500 1.00 0.87
-12.381 -5.271 4.644 1.00 0.87
-12.698 -3.714 6.195 1.00 0.87
-16.821 -7.933 2.926 1.00 0.92
-17.261 -8.655 1.713 1.00 0.92
-18.788 -8.765 1.630 1.00 0.92
-19.365 -8.561 0.548 1.00 0.92
-16.619 -10.042 1.655 1.00 0.92
ATOM 4361 SG CYS D 539
ATOM 4362 N LEU D 540
ATOM 4363 CA LEU D 540
ATOM 4364 C LEU D 540
ATOM 4365 O LEU D 540
ATOM 4366 CB LEU D 540
ATOM 4367 CG LEU D 540
ATOM 4368 CD1 LEU D 540
ATOM 4369 CD2 LEU D 540
ATOM 4370 N THR D 541
ATOM 4371 CA THR D 541
ATOM 4372 C THR D 541
ATOM 4373 O THR D 541
ATOM 4374 CB THR D 541
ATOM 4375 OG1 THR D 541
ATOM 4376 CG2 THR D 541
ATOM 4377 N ILE D 542
ATOM 4378 CA ILE D 542
ATOM 4379 C ILE D 542
ATOM 4380 O ILE D 542

ATOM 4381 CB ILE D 542
ATOM 4382 CG1 ILE D 542
ATOM 4383 CG2 ILE D 542
ATOM 4384 CD1 ILE D 542
ATOM 4385 N VAL D 543
ATOM 4386 CA VAL D 543
ATOM 4387 C VAL D 543
ATOM 4388 O VAL D 543
ATOM 4389 CB VAL D 543
ATOM 4390 CG1 VAL D 543
ATOM 4391 CG2 VAL D 543
ATOM 4392 N VAL D 544
ATOM 4393 CA VAL D 544
ATOM 4394 C VAL D 544
ATOM 4395 O VAL D 544
ATOM 4396 CB VAL D 544
ATOM 4397 CG1 VAL D 544
ATOM 4398 CG2 VAL D 544
ATOM 4399 N ALA D 545
ATOM 4400 CA ALA D 545
ATOM 4401 C ALA D 545
ATOM 4402 O ALA D 545
ATOM 4403 CB ALA D 545
ATOM 4404 N GLY D 546
ATOM 4405 CA GLY D 546
ATOM 4406 C GLY D 546
ATOM 4407 O GLY D 546
ATOM 4408 N LYS D 547
ATOM 4409 CA LYS D 547
ATOM 4410 C LYS D 547
ATOM 4411 O LYS D 547
ATOM 4412 CB LYS D 547
ATOM 4413 CG LYS D 547
ATOM 4414 CD LYS D 547
-14.793 -9.955 1.590 1.00 0.92
-19.434 -8.898 2.774 1.00 0.90
-20.908 -8.877 2.889 1.00 0.90
-21.471 -7.524 2.446 1.00 0.90
-22.409 -7.477 1.649 1.00 0.90
-21.362 -9.159 4.325 1.00 0.90

10665	-21.483	-10.649	4.672	1.00	0.90
10666	-20.170	-11.397	4.542	1.00	0.90
10667	-21.948	-10.820	6.114	1.00	0.90
10668	-20.761	-6.466	2.824	1.00	0.90
10669	-21.142	-5.078	2.494	1.00	0.90
10670	-20.940	-4.780	1.004	1.00	0.90
10671	-21.793	-4.152	0.379	1.00	0.90
10672	-20.391	-4.066	3.370	1.00	0.90
10673	-20.720	-4.324	4.736	1.00	0.90
10674	-20.737	-2.608	3.038	1.00	0.90
10675	-19.848	-5.271	0.436	1.00	0.89
10676	-19.544	-5.056	-0.996	1.00	0.89
10677	-20.599	-5.745	-1.873	1.00	0.89
10678	-21.083	-5.180	-2.855	1.00	0.89
10679	-18.135	-5.577	-1.345	1.00	0.89
10680	-17.034	-4.873	-0.534	1.00	0.89
10681	-17.838	-5.509	-2.853	1.00	0.89
10682	-16.898	-3.361	-0.764	1.00	0.89
10683	-20.916	-6.979	-1.510	1.00	0.88
10684	-21.877	-7.806	-2.268	1.00	0.88
10685	-23.305	-7.265	-2.122	1.00	0.88
10686	-24.042	-7.202	-3.112	1.00	0.88
10687	-21.757	-9.283	-1.851	1.00	0.88
10688	-22.881	-10.159	-2.422	1.00	0.88
10689	-20.424	-9.854	-2.343	1.00	0.88
10690	-23.677	-6.870	-0.910	1.00	0.87
10691	-25.007	-6.268	-0.668	1.00	0.87
10692	-25.153	-4.957	-1.465	1.00	0.87
10693	-26.169	-4.673	-2.057	1.00	0.87
10694	-25.292	-6.084	0.838	1.00	0.87
10695	-24.408	-5.047	1.532	1.00	0.87
10696	-26.751	-5.694	1.073	1.00	0.87
10697	-23.993	-4.252	-1.539	1.00	0.87
10698	-23.806	-3.017	-2.293	1.00	0.87
10699	-24.113	-3.145	-3.775	1.00	0.87
10700	-24.826	-2.345	-4.365	1.00	0.87
10701	-22.337	-2.574	-2.176	1.00	0.87
10702	-23.552	-4.253	-4.282	1.00	0.87
10703	-23.666	-4.639	-5.688	1.00	0.87
10704	-25.109	-5.018	-6.042	1.00	0.87

10705 -25.523 -4.880 -7.182 1.00 0.87
10706 -25.847 -5.479 -5.025 1.00 0.78
10707 -27.222 -5.978 -5.186 1.00 0.78
10708 -28.235 -4.827 -5.165 1.00 0.78
10709 -29.111 -4.737 -6.018 1.00 0.78
10710 -27.511 -6.961 -4.044 1.00 0.78
10711 -28.872 -7.653 -4.158 1.00 0.78
10712 -28.843 -8.800 -5.167 1.00 0.78
10713 ATOM 4415 CE LYS D 547
10714 ATOM 4416 NZ LYS D 547
10715 ATOM 4417 N MET D 548
10716 ATOM 4418 CA MET D 548
10717 ATOM 4419 C MET D 548
10718 ATOM 4420 O MET D 548
10719 ATOM 4421 CB MET D 548
10720 ATOM 4422 CG MET D 548
10721 ATOM 4423 SD MET D 548
10722 ATOM 4424 CE MET D 548
10723 ATOM 4425 N GLY D 549
10724 ATOM 4426 CA GLY D 549
10725 ATOM 4427 C GLY D 549
10726 ATOM 4428 O GLY D 549
10727 ATOM 4429 N LYS D 550
10728 ATOM 4430 CA LYS D 550
10729 ATOM 4431 C LYS D 550
10730 ATOM 4432 O LYS D 550
10731 ATOM 4433 CB LYS D 550
10732 ATOM 4434 CG LYS D 550
10733 ATOM 4435 CD LYS D 550
10734 ATOM 4436 CE LYS D 550
10735 ATOM 4437 NZ LYS D 550
10736 ATOM 4438 N LYS D 551
10737 ATOM 4439 CA LYS D 551
10738 ATOM 4440 C LYS D 551
10739 ATOM 4441 O LYS D 551
10740 ATOM 4442 CB LYS D 551
10741 ATOM 4443 CG LYS D 551
10742 ATOM 4444 CD LYS D 551
10743 ATOM 4445 CE LYS D 551
10744 ATOM 4446 NZ LYS D 551

ATOM	4447	N	LEU	D	552	
ATOM	4448	CA	LEU	D	552	
ATOM	4449	C	LEU	D	552	
ATOM	4450	O	LEU	D	552	
ATOM	4451	CB	LEU	D	552	
ATOM	4452	CG	LEU	D	552	
ATOM	4453	CD1	LEU	D	552	
ATOM	4454	CD2	LEU	D	552	
ATOM	4455	N	SER	D	553	
ATOM	4456	CA	SER	D	553	
ATOM	4457	C	SER	D	553	
ATOM	4458	O	SER	D	553	
ATOM	4459	CB	SER	D	553	
ATOM	4460	OG	SER	D	553	
ATOM	4461	N	PRO	D	554	
ATOM	4462	CA	PRO	D	554	
ATOM	4463	C	PRO	D	554	
ATOM	4464	O	PRO	D	554	
ATOM	4465	CB	PRO	D	554	
ATOM	4466	CG	PRO	D	554	
ATOM	4467	CD	PRO	D	554	
ATOM	4468	N	GLU	D	555	

-30.115	-9.634	-5.017	1.00	0.78
-29.923	-10.970	-5.594	1.00	0.78
-28.126	-4.001	-4.130	1.00	0.78
-29.043	-2.870	-3.918	1.00	0.78
-28.798	-1.683	-4.859	1.00	0.78
-29.681	-0.839	-5.035	1.00	0.78
-28.954	-2.435	-2.461	1.00	0.78
-29.496	-3.536	-1.544	1.00	0.78
-29.613	-2.993	0.191	1.00	0.78
-30.911	-1.825	-0.139	1.00	0.78
-27.554	-1.566	-5.349	1.00	0.85
-27.153	-0.466	-6.246	1.00	0.85
-27.345	0.885	-5.547	1.00	0.85
-27.081	1.015	-4.351	1.00	0.85
-28.006	1.798	-6.246	1.00	0.76
-28.302	3.157	-5.742	1.00	0.76
-29.063	3.178	-4.405	1.00	0.76
-28.916	4.108	-3.616	1.00	0.76

-29.140	3.924	-6.759	1.00	0.76	
-28.396	4.159	-8.076	1.00	0.76	
-29.264	4.936	-9.071	1.00	0.76	
-29.602	6.355	-8.597	1.00	0.76	
-28.394	7.184	-8.470	1.00	0.76	
-29.853	2.127	-4.172	1.00	0.78	
-30.610	1.968	-2.914	1.00	0.78	
-29.673	1.861	-1.706	1.00	0.78	
-30.066	2.212	-0.576	1.00	0.78	
-31.505	0.732	-3.014	1.00	0.78	
-32.480	0.607	-1.841	1.00	0.78	
-33.441	1.790	-1.715	1.00	0.78	
-34.358	1.624	-0.503	1.00	0.78	
-33.616	1.683	0.767	1.00	0.78	
-28.471	1.379	-1.960	1.00	0.88	
-27.403	1.304	-0.965	1.00	0.88	
-26.818	2.680	-0.663	1.00	0.88	
-25.633	2.945	-0.854	1.00	0.88	
-26.368	0.237	-1.306	1.00	0.88	
-25.432	0.126	-0.094	1.00	0.88	
-25.867	-0.776	1.048	1.00	0.88	
-24.127	-0.452	-0.514	1.00	0.88	
-27.653	3.516	-0.080	1.00	0.94	
-27.304	4.905	0.212	1.00	0.94	
-26.250	4.995	1.332	1.00	0.94	
-26.121	4.022	2.125	1.00	0.94	
-28.603	5.655	0.540	1.00	0.94	
-29.095	5.293	1.831	1.00	0.94	
-25.599	6.130	1.498	1.00	0.98	
-24.631	6.380	2.590	1.00	0.98	
-25.263	6.195	3.982	1.00	0.98	
-24.606	5.757	4.916	1.00	0.98	
-24.163	7.818	2.383	1.00	0.98	
-25.360	8.493	1.716	1.00	0.98	
-25.830	7.406	0.757	1.00	0.98	
-26.577	6.430	4.062	1.00	0.87	
ATOM	4469	CA	GLU	D	555
ATOM	4470	C	GLU	D	555
ATOM	4471	O	GLU	D	555
ATOM	4472	CB	GLU	D	555

10825	ATOM	4473	CG	GLU	D	555
10826	ATOM	4474	CD	GLU	D	555
10827	ATOM	4475	OE1	GLU	D	555
10828	ATOM	4476	OE2	GLU	D	555
10829	ATOM	4477	N	MET	D	556
10830	ATOM	4478	CA	MET	D	556
10831	ATOM	4479	C	MET	D	556
10832	ATOM	4480	O	MET	D	556
10833	ATOM	4481	CB	MET	D	556
10834	ATOM	4482	CG	MET	D	556
10835	ATOM	4483	SD	MET	D	556
10836	ATOM	4484	CE	MET	D	556
10837	ATOM	4485	N	GLN	D	557
10838	ATOM	4486	CA	GLN	D	557
10839	ATOM	4487	C	GLN	D	557
10840	ATOM	4488	O	GLN	D	557
10841	ATOM	4489	CB	GLN	D	557
10842	ATOM	4490	CG	GLN	D	557
10843	ATOM	4491	CD	GLN	D	557
10844	ATOM	4492	OE1	GLN	D	557
10845	ATOM	4493	NE2	GLN	D	557
10846	ATOM	4494	N	ALA	D	558
10847	ATOM	4495	CA	ALA	D	558
10848	ATOM	4496	C	ALA	D	558
10849	ATOM	4497	O	ALA	D	558
10850	ATOM	4498	CB	ALA	D	558
10851	ATOM	4499	N	ALA	D	559
10852	ATOM	4500	CA	ALA	D	559
10853	ATOM	4501	C	ALA	D	559
10854	ATOM	4502	O	ALA	D	559
10855	ATOM	4503	CB	ALA	D	559
10856	ATOM	4504	N	TRP	D	560
10857	ATOM	4505	CA	TRP	D	560
10858	ATOM	4506	C	TRP	D	560
10859	ATOM	4507	O	TRP	D	560
10860	ATOM	4508	CB	TRP	D	560
10861	ATOM	4509	CG	TRP	D	560
10862	ATOM	4510	CD1	TRP	D	560
10863	ATOM	4511	CD2	TRP	D	560
10864	ATOM	4512	NE1	TRP	D	560

10865 ATOM 4513 CE2 TRP D 560
10866 ATOM 4514 CE3 TRP D 560
10867 ATOM 4515 CZ2 TRP D 560
10868 ATOM 4516 CZ3 TRP D 560
10869 ATOM 4517 CH2 TRP D 560
10870 ATOM 4518 N GLN D 561
10871 ATOM 4519 CA GLN D 561
10872 ATOM 4520 C GLN D 561
10873 ATOM 4521 O GLN D 561
10874 ATOM 4522 CB GLN D 561
10875 -27.327 6.284 5.321 1.00 0.87
10876 -27.542 4.806 5.670 1.00 0.87
10877 -27.458 4.410 6.832 1.00 0.87
10878 -28.665 7.014 5.203 1.00 0.87
10879 -29.442 6.984 6.525 1.00 0.87
10880 -30.708 7.839 6.476 1.00 0.87
10881 -30.647 8.931 5.871 1.00 0.87
10882 -31.717 7.369 7.045 1.00 0.87
10883 -27.800 4.005 4.645 1.00 0.88
10884 -28.010 2.572 4.861 1.00 0.88
10885 -26.697 1.806 5.039 1.00 0.88
10886 -26.619 0.952 5.925 1.00 0.88
10887 -28.826 2.020 3.716 1.00 0.88
10888 -29.163 0.554 4.010 1.00 0.88
10889 -30.116 -0.232 2.679 1.00 0.88
10890 -28.915 0.176 1.461 1.00 0.88
10891 -25.665 2.200 4.299 1.00 0.88
10892 -24.309 1.654 4.507 1.00 0.88
10893 -23.875 1.870 5.968 1.00 0.88
10894 -23.374 0.956 6.615 1.00 0.88
10895 -23.312 2.361 3.591 1.00 0.88
10896 -21.913 1.751 3.741 1.00 0.88
10897 -20.818 2.579 3.074 1.00 0.88
10898 -21.045 3.551 2.366 1.00 0.88
10899 -19.588 2.206 3.340 1.00 0.88
10900 -24.242 3.042 6.492 1.00 0.91
10901 -23.920 3.448 7.871 1.00 0.91
10902 -24.553 2.514 8.909 1.00 0.91
10903 -23.850 1.925 9.728 1.00 0.91
10904 -24.398 4.884 8.104 1.00 0.91

10905 -25.852 2.255 8.730 1.00 0.91
10906 -26.626 1.399 9.639 1.00 0.91
10907 -26.242 -0.079 9.477 1.00 0.91
10908 -25.912 -0.728 10.431 1.00 0.91
10909 -28.125 1.581 9.402 1.00 0.91
10910 -25.990 -0.512 8.236 1.00 0.92
10911 -25.614 -1.916 7.987 1.00 0.92
10912 -24.235 -2.268 8.561 1.00 0.92
10913 -24.054 -3.302 9.213 1.00 0.92
10914 -25.656 -2.175 6.480 1.00 0.92
10915 -25.303 -3.629 6.173 1.00 0.92
10916 -24.144 -4.073 5.699 1.00 0.92
10917 -26.151 -4.721 6.297 1.00 0.92
10918 -24.224 -5.391 5.504 1.00 0.92
10919 -25.427 -5.817 5.858 1.00 0.92
10920 -27.450 -4.864 6.745 1.00 0.92
10921 -26.019 -7.081 5.849 1.00 0.92
10922 -28.030 -6.132 6.731 1.00 0.92
10923 -27.325 -7.231 6.276 1.00 0.92
10924 -23.299 -1.340 8.414 1.00 0.87
10925 -21.940 -1.523 8.946 1.00 0.87
10926 -21.895 -1.420 10.471 1.00 0.87
10927 -21.186 -2.193 11.114 1.00 0.87
10928 -20.989 -0.513 8.332 1.00 0.87
10929 ATOM 4523 CG GLN D 561
10930 ATOM 4524 CD GLN D 561
10931 ATOM 4525 OE1 GLN D 561
10932 ATOM 4526 NE2 GLN D 561
10933 ATOM 4527 N LYS D 562
10934 ATOM 4528 CA LYS D 562
10935 ATOM 4529 C LYS D 562
10936 ATOM 4530 O LYS D 562
10937 ATOM 4531 CB LYS D 562
10938 ATOM 4532 CG LYS D 562
10939 ATOM 4533 CD LYS D 562
10940 ATOM 4534 CE LYS D 562
10941 ATOM 4535 NZ LYS D 562
10942 ATOM 4536 N TYR D 563
10943 ATOM 4537 CA TYR D 563
10944 ATOM 4538 C TYR D 563

ATOM	4539	O	TYR	D	563	
ATOM	4540	CB	TYR	D	563	
ATOM	4541	CG	TYR	D	563	
ATOM	4542	CD1	TYR	D	563	
ATOM	4543	CD2	TYR	D	563	
ATOM	4544	CE1	TYR	D	563	
ATOM	4545	CE2	TYR	D	563	
ATOM	4546	CZ	TYR	D	563	
ATOM	4547	OH	TYR	D	563	
ATOM	4548	N	LEU	D	564	
ATOM	4549	CA	LEU	D	564	
ATOM	4550	C	LEU	D	564	
ATOM	4551	O	LEU	D	564	
ATOM	4552	CB	LEU	D	564	
ATOM	4553	CG	LEU	D	564	
ATOM	4554	CD1	LEU	D	564	
ATOM	4555	CD2	LEU	D	564	
ATOM	4556	N	CYS	D	565	
ATOM	4557	CA	CYS	D	565	
ATOM	4558	C	CYS	D	565	
ATOM	4559	O	CYS	D	565	
ATOM	4560	CB	CYS	D	565	
ATOM	4561	SG	CYS	D	565	
ATOM	4562	N	ALA	D	566	
ATOM	4563	CA	ALA	D	566	
ATOM	4564	C	ALA	D	566	
ATOM	4565	O	ALA	D	566	
ATOM	4566	CB	ALA	D	566	
ATOM	4567	N	VAL	D	567	
ATOM	4568	CA	VAL	D	567	
ATOM	4569	C	VAL	D	567	
ATOM	4570	O	VAL	D	567	
ATOM	4571	CB	VAL	D	567	
ATOM	4572	CG1	VAL	D	567	
ATOM	4573	CG2	VAL	D	567	
ATOM	4574	N	VAL	D	568	
ATOM	4575	CA	VAL	D	568	
ATOM	4576	C	VAL	D	568	
-20.658	-0.876	6.878	1.00	0.87		
-19.586	0.037	6.270	1.00	0.87		

10985	-19.343	0.035	5.072	1.00	0.87
10986	-18.856	0.746	7.105	1.00	0.87
10987	-22.760	-0.573	11.021	1.00	0.86
10988	-22.935	-0.467	12.483	1.00	0.86
10989	-23.463	-1.791	13.059	1.00	0.86
10990	-23.052	-2.267	14.129	1.00	0.86
10991	-23.878	0.696	12.794	1.00	0.86
10992	-24.095	0.844	14.298	1.00	0.86
10993	-24.924	2.087	14.568	1.00	0.86
10994	-25.237	2.173	16.052	1.00	0.86
10995	-25.886	3.457	16.297	1.00	0.86
10996	-24.305	-2.445	12.299	1.00	0.92
10997	-24.904	-3.723	12.717	1.00	0.92
10998	-23.893	-4.854	12.654	1.00	0.92
10999	-23.729	-5.593	13.624	1.00	0.92
11000	-26.094	-4.046	11.833	1.00	0.92
11001	-27.233	-3.082	12.115	1.00	0.92
11002	-27.108	-1.727	12.412	1.00	0.92
11003	-28.496	-3.574	12.009	1.00	0.92
11004	-28.153	-0.846	12.478	1.00	0.92
11005	-29.516	-2.700	12.245	1.00	0.92
11006	-29.403	-1.354	12.414	1.00	0.92
11007	-30.412	-0.724	12.997	1.00	0.92
11008	-23.104	-4.828	11.588	1.00	0.89
11009	-22.037	-5.821	11.404	1.00	0.89
11010	-20.904	-5.676	12.416	1.00	0.89
11011	-20.467	-6.673	12.993	1.00	0.89
11012	-21.475	-5.798	9.985	1.00	0.89
11013	-22.407	-6.483	8.980	1.00	0.89
11014	-21.772	-6.424	7.592	1.00	0.89
11015	-22.677	-7.944	9.359	1.00	0.89
11016	-20.557	-4.433	12.728	1.00	0.88
11017	-19.502	-4.142	13.716	1.00	0.88
11018	-19.917	-4.577	15.131	1.00	0.88
11019	-19.106	-5.134	15.867	1.00	0.88
11020	-19.101	-2.665	13.679	1.00	0.88
11021	-20.394	-1.518	14.272	1.00	0.88
11022	-21.215	-4.452	15.427	1.00	0.89
11023	-21.793	-4.911	16.706	1.00	0.89
11024	-21.749	-6.440	16.852	1.00	0.89

11025	-21.363	-6.962	17.903	1.00	0.89	
11026	-23.239	-4.423	16.823	1.00	0.89	
11027	-21.985	-7.135	15.743	1.00	0.87	
11028	-22.026	-8.614	15.714	1.00	0.87	
11029	-20.606	-9.192	15.757	1.00	0.87	
11030	-20.341	-10.157	16.477	1.00	0.87	
11031	-22.795	-9.119	14.480	1.00	0.87	
11032	-22.825	-10.651	14.407	1.00	0.87	
11033	-24.238	-8.612	14.502	1.00	0.87	
11034	-19.704	-8.563	15.017	1.00	0.85	
11035	-18.270	-8.930	15.009	1.00	0.85	
11036	-17.688	-8.788	16.416	1.00	0.85	
11037	ATOM	4577	O	VAL	D	568
11038	ATOM	4578	CB	VAL	D	568
11039	ATOM	4579	CG1	VAL	D	568
11040	ATOM	4580	CG2	VAL	D	568
11041	ATOM	4581	N	SER	D	569
11042	ATOM	4582	CA	SER	D	569
11043	ATOM	4583	C	SER	D	569
11044	ATOM	4584	O	SER	D	569
11045	ATOM	4585	CB	SER	D	569
11046	ATOM	4586	OG	SER	D	569
11047	ATOM	4587	N	ALA	D	570
11048	ATOM	4588	CA	ALA	D	570
11049	ATOM	4589	C	ALA	D	570
11050	ATOM	4590	O	ALA	D	570
11051	ATOM	4591	CB	ALA	D	570
11052	ATOM	4592	N	LEU	D	571
11053	ATOM	4593	CA	LEU	D	571
11054	ATOM	4594	C	LEU	D	571
11055	ATOM	4595	O	LEU	D	571
11056	ATOM	4596	CB	LEU	D	571
11057	ATOM	4597	CG	LEU	D	571
11058	ATOM	4598	CD1	LEU	D	571
11059	ATOM	4599	CD2	LEU	D	571
11060	ATOM	4600	N	GLY	D	572
11061	ATOM	4601	CA	GLY	D	572
11062	ATOM	4602	C	GLY	D	572
11063	ATOM	4603	O	GLY	D	572
11064	ATOM	4604	N	ARG	D	573

11065 ATOM 4605 CA ARG D 573
11066 ATOM 4606 C ARG D 573
11067 ATOM 4607 O ARG D 573
11068 ATOM 4608 CB ARG D 573
11069 ATOM 4609 CG ARG D 573
11070 ATOM 4610 CD ARG D 573
11071 ATOM 4611 NE ARG D 573
11072 ATOM 4612 CZ ARG D 573
11073 ATOM 4613 NH1 ARG D 573
11074 ATOM 4614 NH2 ARG D 573
11075 ATOM 4615 N GLN D 574
11076 ATOM 4616 CA GLN D 574
11077 ATOM 4617 C GLN D 574
11078 ATOM 4618 O GLN D 574
11079 ATOM 4619 CB GLN D 574
11080 ATOM 4620 CG GLN D 574
11081 ATOM 4621 CD GLN D 574
11082 ATOM 4622 OE1 GLN D 574
11083 ATOM 4623 NE2 GLN D 574
11084 ATOM 4624 N TYR D 575
11085 ATOM 4625 CA TYR D 575
11086 ATOM 4626 C TYR D 575
11087 ATOM 4627 O TYR D 575
11088 ATOM 4628 CB TYR D 575
11089 ATOM 4629 CG TYR D 575
11090 ATOM 4630 CD1 TYR D 575
11091 -16.964 -9.665 16.884 1.00 0.85
11092 -17.494 -8.029 14.041 1.00 0.85
11093 -15.971 -8.196 14.096 1.00 0.85
11094 -17.902 -8.414 12.638 1.00 0.85
11095 -18.060 -7.686 17.056 1.00 0.84
11096 -17.597 -7.377 18.418 1.00 0.84
11097 -18.038 -8.484 19.391 1.00 0.84
11098 -17.234 -9.050 20.123 1.00 0.84
11099 -18.164 -6.015 18.821 1.00 0.84
11100 -17.520 -5.561 20.011 1.00 0.84
11101 -19.285 -8.927 19.197 1.00 0.83
11102 -19.895 -10.033 19.945 1.00 0.83
11103 -19.237 -11.404 19.686 1.00 0.83
11104 -18.923 -12.167 20.601 1.00 0.83

11105	-21.374	-10.053	19.550	1.00	0.83
11106	-18.891	-11.634	18.425	1.00	0.79
11107	-18.260	-12.890	17.990	1.00	0.79
11108	-16.876	-13.139	18.594	1.00	0.79
11109	-16.459	-14.279	18.709	1.00	0.79
11110	-18.155	-12.951	16.466	1.00	0.79
11111	-19.473	-13.344	15.796	1.00	0.79
11112	-19.295	-13.285	14.279	1.00	0.79
11113	-19.915	-14.752	16.213	1.00	0.79
11114	-16.213	-12.034	18.979	1.00	0.81
11115	-14.836	-12.095	19.485	1.00	0.81
11116	-14.692	-11.705	20.960	1.00	0.81
11117	-13.574	-11.457	21.403	1.00	0.81
11118	-15.771	-11.819	21.734	1.00	0.68
11119	-15.703	-11.465	23.165	1.00	0.68
11120	-15.294	-12.641	24.066	1.00	0.68
11121	-14.676	-12.450	25.103	1.00	0.68
11122	-16.992	-10.813	23.669	1.00	0.68
11123	-18.196	-11.746	23.647	1.00	0.68
11124	-19.338	-11.070	24.396	1.00	0.68
11125	-20.496	-11.959	24.332	1.00	0.68
11126	-21.291	-12.084	23.292	1.00	0.68
11127	-21.189	-11.383	22.198	1.00	0.68
11128	-22.193	-12.997	23.348	1.00	0.68
11129	-15.563	-13.857	23.588	1.00	0.67
11130	-15.262	-15.082	24.350	1.00	0.67
11131	-13.901	-15.688	23.973	1.00	0.67
11132	-13.625	-16.866	24.243	1.00	0.67
11133	-16.384	-16.102	24.136	1.00	0.67
11134	-17.789	-15.558	24.452	1.00	0.67
11135	-17.937	-14.941	25.844	1.00	0.67
11136	-18.672	-13.990	26.052	1.00	0.67
11137	-17.238	-15.499	26.809	1.00	0.67
11138	-13.049	-14.868	23.380	1.00	0.66
11139	-11.654	-15.230	23.080	1.00	0.66
11140	-10.771	-14.880	24.280	1.00	0.66
11141	-10.682	-13.723	24.690	1.00	0.66
11142	-11.163	-14.500	21.829	1.00	0.66
11143	-11.676	-15.150	20.542	1.00	0.66
11144	-12.951	-14.871	20.073	1.00	0.66

```
ATOM   4631  CD2 TYR D 575
ATOM   4632  CE1 TYR D 575
ATOM   4633  CE2 TYR D 575
ATOM   4634  CZ  TYR D 575
ATOM   4635  OH  TYR D 575
ATOM   4636  N   HIS D 576
ATOM   4637  CA  HIS D 576
ATOM   4638  C   HIS D 576
ATOM   4639  O   HIS D 576
ATOM   4640  CB  HIS D 576
ATOM   4641  CG  HIS D 576
ATOM   4642  ND1 HIS D 576
ATOM   4643  CD2 HIS D 576
ATOM   4644  CE1 HIS D 576
ATOM   4645  NE2 HIS D 576
CONECT    1    5   32
CONECT    2    8   15
CONECT    3   18   22
CONECT    4   25   29
CONECT    5    6   39
CONECT    6    7   10
CONECT    7    8    9
CONECT    8   39
CONECT   10   11
CONECT   11   12
CONECT   12   13   14
CONECT   15   16   40
CONECT   16   17   19
CONECT   17   18   20
CONECT   18   40
CONECT   20   21
CONECT   22   23   41
CONECT   23   24   26
CONECT   24   25   27
CONECT   25   41
CONECT   27   28
CONECT   29   30   42
CONECT   30   31   33
CONECT   31   32   34
CONECT   32   42
```

11185 CONECT 34 35
11186 CONECT 35 36
11187 CONECT 36 37 38
11188 CONECT 39 43
11189 CONECT 40 43
11190 CONECT 41 43
11191 CONECT 42 43
11192 CONECT 44 48 75
11193 CONECT 45 51 58
11194 CONECT 46 61 65
11195 CONECT 47 68 72
11196 CONECT 48 49 82
11197 CONECT 49 50 53
11198 CONECT 50 51 52
11199 -10.835 -15.972 19.804 1.00 0.66
11200 -13.385 -15.413 18.871 1.00 0.66
11201 -11.269 -16.517 18.604 1.00 0.66
11202 -12.546 -16.235 18.136 1.00 0.66
11203 -12.992 -16.791 16.981 1.00 0.66
11204 -10.202 -15.920 24.870 1.00 0.63
11205 -9.337 -15.784 26.062 1.00 0.63
11206 -7.956 -16.415 25.849 1.00 0.63
11207 -7.015 -15.956 26.528 1.00 0.63
11208 -10.019 -16.424 27.276 1.00 0.63
11209 -11.389 -15.802 27.569 1.00 0.63
11210 -12.532 -16.475 27.652 1.00 0.63
11211 -11.651 -14.517 27.804 1.00 0.63
11212 -13.498 -15.610 27.940 1.00 0.63
11213 -12.954 -14.402 28.039 1.00 0.63
11214 CONECT 51 82
11215 CONECT 53 54
11216 CONECT 54 55
11217 CONECT 55 56 57
11218 CONECT 58 59 83
11219 CONECT 59 60 62
11220 CONECT 60 61 63
11221 CONECT 61 83
11222 CONECT 63 64
11223 CONECT 65 66 84
11224 CONECT 66 67 69

```
CONECT   67   68   70
CONECT   68   84
CONECT   70   71
CONECT   72   73   85
CONECT   73   74   76
CONECT   74   75   77
CONECT   75   85
CONECT   77   78
CONECT   78   79
CONECT   79   80   81
CONECT   82   86
CONECT   83   86
CONECT   84   86
CONECT   85   86
CONECT   87   91  118
CONECT   88   94  101
CONECT   89  104  108
CONECT   90  111  115
CONECT   91   92  125
CONECT   92   93   96
CONECT   93   94   95
CONECT   94  125
CONECT   96   97
CONECT   97   98
CONECT   98   99  100
CONECT  101  102  126
CONECT  102  103  105
CONECT  103  104  106
CONECT  104  126
CONECT  106  107
CONECT  108  109  127
CONECT  109  110  112
CONECT  110  111  113
CONECT  111  127
CONECT  113  114
CONECT  115  116  128
CONECT  116  117  119
CONECT  117  118  120
CONECT  118  128
CONECT  120  121
```

```
CONECT  121  122
CONECT  122  123  124
CONECT  125  129
CONECT  126  129
CONECT  127  129
CONECT  128  129
CONECT  130  134  161
CONECT  131  137  144
CONECT  132  147  151
CONECT  133  154  158
CONECT  134  135  168
CONECT  135  136  139
CONECT  136  137  138
CONECT  137  168
CONECT  139  140
CONECT  140  141
CONECT  141  142  143
CONECT  144  145  169
CONECT  145  146  148
CONECT  146  147  149
CONECT  147  169
CONECT  149  150
CONECT  151  152  170
CONECT  152  153  155
CONECT  153  154  156
CONECT  154  170
CONECT  156  157
CONECT  158  159  171
CONECT  159  160  162
CONECT  160  161  163
CONECT  161  171
CONECT  163  164
CONECT  164  165
CONECT  165  166  167
CONECT  168  172
CONECT  169  172
CONECT  170  172
CONECT  171  172
SPDBVT
SPDBVT
```

```
SPDBVT
SPDBVT
SPDBVT
SPDBVV default;
SPDBVV     11.008075561409   18902.382304785824      20.000000000000
 1.0000000000
-0.0000160655
-0.0002409692
-5.6660819054
-5.6667232513
0.0000160676
1.0000000000
0.0000085569
 0.0002409690
-0.0000085608
 1.0000000000
11.3785476685
11.3778905869
SPDBVV
SPDBVV
SPDBVV
SPDBVV
SPDBVV
SPDBVf
SPDBVf 32 32 32 32 32 32 32 32 32 32 32 32 32 32 32 32 32 32 32 32
SPDBVf 32 32 48 32 32 32 32 32 32 32 32 32 32 32 32 32 32 32 32 32
SPDBVf 32 32 32 32 32 32 32 32 32 32 32 32 32 32 32 32 32 32 32 32
SPDBVf 32 32 32 32 32 32 32 32 32 32 32 32 32 32 32 32 32 32 32 32
SPDBVf 32 32 32 32 32 32 32 32 32 32 32 32 32 32 32 32 32 32 32 32
SPDBVf 32 32 32 32 32 32 32 32 32 32 32 32 32 32 32 32 32 32 32 32
   0.1942839053
  -0.9387565073
   0.2845873930
  -5.9695000648
-386.9097737234
-0.6499777759
-0.3404844656
-0.6794109357
-1.3029999733
 0.7346990234
```

```
-0.0529768708
-0.6763215183
11.7095003128
-1.1893286705
-1.1911008358
-260.1804046826 1 1 1 1323232323232323232323232323232
0.0000000000
SPDBVf 32 32 32 32 32 32 32 32 32 32 32 32 32 32 32 32 32 32 32 32
SPDBVf 32 32 32 32 32 32 32 32 32 32 32 32 32 32 32 32 32 32 32 32
SPDBVf 32 32 32 32 32 32 32 32 32 32 32 32 32 32 32 32 32 32 32 32
SPDBVf 32 32 32 32 32 32 32 32 32 32 32 32 32 32 32 32 32 32 32 32
SPDBVf 32 32 32 32 32 32 32 32 32 32 32 32 32 32 32 32 32 32 32 32
SPDBVf 32 32 32 32 32 32 32 32 32 32 32 32 32 32 32 32 32 32 32 32
SPDBVf 32 32 32 32 32 32 32 32 32 32 32 32 32 32 32 32 32 32 32 32
SPDBVf 32 32 32 32 32 32 32 32 32 32 32 32 32 32 32 32 32 32 32 32
SPDBVf 32 32 32 32 32 32 32 32 32 32 32 32 32 32 32 32 32 32 32 32
SPDBVf 32 32 32 32 32 32 32 32 32 32 32 32 32 32 32 32 32 32 32 32
SPDBVf 32 32 32 32 32 32 32 32 32 32 32 32 32 32 32 32 32 32 32 32
SPDBVf 32 32 32 32 32 32 32 32 32 32 32 32 32 32 32 32 32 32 32 32
SPDBVf 32 32 32 32 32 32 32 32 32 32 32 32 32 32 32 32 32 32 32 32
SPDBVf 32 32 32 32 32 32 32 32 32 32 32 32 32 32 32 32 32 32 32 32
SPDBVf 32 32 32 32 32 32 32 32 32 32 32 32 32 32 32 32 32 32 32 32
SPDBVf 32 32 32 32 32 32 32 32 32 32 32 32 32 32 32 32 32 32 32 32
SPDBVf 32 32 32 32 32 32 32 32 32 32 32 32 32 32 32 32 32 32 32 32
SPDBVf 32 32 32 32 32 32 32 32 32 32 32 32 32 32 32 32 32 32 32 32
SPDBVf 32 32 32 32 32 32 32 32 32 32 32 32 32 32 32 32 32 32 32 32
SPDBVf 32 32 32 32 32 32 32 32 32 32 32 32 32 32 32 32 32 32 32 32
SPDBVf 32 32 32 32 32 32 32 32 32 32 32 32 32 32 32 32 32 32 32 32
SPDBVf 32 32 32 32 32 32 32 32 32 32 32 32 32 32 32 32 32 32 32
SPDBVo 1d d
SPDBVo 44d HEM
SPDBVo 87d HEM
SPDBVo 130d HEM
SPDBVc 0.03 0.52 0.77 0.03 0.52 0.77 0.03 0.52 0.77 0.03 0.52 0.77
SPDBVc 1.00 1.00 0.00 1.00 1.00 0.00 1.00 1.00 0.00 1.00 1.00 0.00
SPDBVc 1.00 1.00 0.00 1.00 1.00 0.00 1.00 1.00 0.00 1.00 1.00 0.00
SPDBVc 1.00 1.00 0.00 1.00 1.00 0.00 1.00 1.00 0.00 1.00 1.00 0.00
SPDBVc 1.00 1.00 0.00 1.00 1.00 0.00 1.00 1.00 0.00 1.00 1.00 0.00
SPDBVc 1.00 1.00 0.00 1.00 1.00 0.00 1.00 1.00 0.00 1.00 1.00 0.00
SPDBVc 1.00 1.00 0.00 1.00 1.00 0.00 1.00 1.00 0.00 1.00 1.00 0.00
```

11385	SPDBVc	1.00	1.00	0.00	1.00	1.00	0.00	1.00	1.00	0.00	1.00	1.00	0.00
11386	SPDBVc	1.00	1.00	0.00	1.00	1.00	0.00	1.00	1.00	0.00	1.00	1.00	0.00
11387	SPDBVc	1.00	1.00	0.00	1.00	1.00	0.00	1.00	1.00	0.00	1.00	1.00	0.00
11388	SPDBVc	1.00	1.00	0.00	1.00	1.00	0.00	1.00	1.00	0.00	1.00	1.00	0.00
11389	SPDBVc	1.00	1.00	0.00	1.00	1.00	0.00	1.00	1.00	0.00	1.00	1.00	0.00
11390	SPDBVc	1.00	1.00	0.00	1.00	1.00	0.00	1.00	1.00	0.00	1.00	1.00	0.00
11391	SPDBVc	1.00	1.00	0.00	1.00	1.00	0.00	1.00	1.00	0.00	1.00	1.00	0.00
11392	SPDBVc	1.00	1.00	0.00	1.00	1.00	0.00	1.00	1.00	0.00	1.00	1.00	0.00
11393	SPDBVc	1.00	1.00	0.00	1.00	1.00	0.00	1.00	1.00	0.00	1.00	1.00	0.00
11394	SPDBVc	1.00	1.00	0.00	1.00	1.00	0.00	1.00	1.00	0.00	1.00	1.00	0.00
11395	SPDBVc	1.00	1.00	0.00	1.00	1.00	0.00	1.00	1.00	0.00	1.00	1.00	0.00
11396	SPDBVc	1.00	1.00	0.00	1.00	1.00	0.00	1.00	1.00	0.00	1.00	1.00	0.00
11397	SPDBVc	1.00	1.00	0.00	1.00	1.00	0.00	1.00	1.00	0.00	1.00	1.00	0.00
11398	SPDBVc	1.00	1.00	0.00	1.00	1.00	0.00	1.00	1.00	0.00	1.00	1.00	0.00
11399	SPDBVc	1.00	1.00	0.00	1.00	1.00	0.00	1.00	1.00	0.00	1.00	1.00	0.00
11400	SPDBVc	1.00	1.00	0.00	1.00	1.00	0.00	1.00	1.00	0.00	1.00	1.00	0.00
11401	SPDBVc	1.00	1.00	0.00	1.00	1.00	0.00	1.00	1.00	0.00	1.00	1.00	0.00
11402	SPDBVc	1.00	1.00	0.00	1.00	1.00	0.00	1.00	1.00	0.00	1.00	1.00	0.00
11403	SPDBVc	1.00	1.00	0.00	1.00	1.00	0.00	1.00	1.00	0.00	1.00	1.00	0.00
11404	SPDBVc	1.00	1.00	0.00	1.00	1.00	0.00	1.00	1.00	0.00	1.00	1.00	0.00
11405	SPDBVc	1.00	1.00	0.00	1.00	1.00	0.00	1.00	1.00	0.00	1.00	1.00	0.00
11406	SPDBVc	1.00	1.00	0.00	1.00	1.00	0.00	1.00	1.00	0.00	1.00	1.00	0.00
11407	SPDBVc	1.00	1.00	0.00	1.00	1.00	0.00	1.00	1.00	0.00	1.00	1.00	0.00
11408	SPDBVc	1.00	1.00	0.00	1.00	1.00	0.00	1.00	1.00	0.00	1.00	1.00	0.00
11409	SPDBVc	1.00	1.00	0.00	1.00	1.00	0.00	1.00	1.00	0.00	1.00	1.00	0.00
11410	SPDBVc	1.00	1.00	0.00	1.00	1.00	0.00	1.00	1.00	0.00	1.00	1.00	0.00
11411	SPDBVc	1.00	1.00	0.00	1.00	1.00	0.00	1.00	1.00	0.00	1.00	1.00	0.00
11412	SPDBVc	1.00	1.00	0.00	1.00	1.00	0.00	1.00	1.00	0.00	1.00	1.00	0.00
11413	SPDBVc	1.00	1.00	0.00	1.00	1.00	0.00	1.00	1.00	0.00	1.00	1.00	0.00
11414	SPDBVc	1.00	1.00	0.00	1.00	1.00	0.00	0.30	0.30	1.00	0.30	0.30	1.00
11415	SPDBVc	0.30	0.30	1.00	0.30	0.30	1.00	0.30	0.30	1.00	0.30	0.30	1.00
11416	SPDBVc	0.30	0.30	1.00	0.30	0.30	1.00	0.30	0.30	1.00	0.30	0.30	1.00
11417	SPDBVc	0.30	0.30	1.00	0.30	0.30	1.00	0.30	0.30	1.00	0.30	0.30	1.00
11418	SPDBVc	0.30	0.30	1.00	0.30	0.30	1.00	0.30	0.30	1.00	0.30	0.30	1.00
11419	SPDBVc	0.30	0.30	1.00	0.30	0.30	1.00	0.30	0.30	1.00	0.30	0.30	1.00
11420	SPDBVc	0.30	0.30	1.00	0.30	0.30	1.00	0.30	0.30	1.00	0.30	0.30	1.00
11421	SPDBVc	0.30	0.30	1.00	0.30	0.30	1.00	0.30	0.30	1.00	0.30	0.30	1.00
11422	SPDBVc	0.30	0.30	1.00	0.30	0.30	1.00	0.30	0.30	1.00	0.30	0.30	1.00
11423	SPDBVc	0.30	0.30	1.00	0.30	0.30	1.00	0.30	0.30	1.00	0.30	0.30	1.00
11424	SPDBVc	0.30	0.30	1.00	0.30	0.30	1.00	0.30	0.30	1.00	0.30	0.30	1.00

11425	SPDBVc	0.30	0.30	1.00	0.30	0.30	1.00	0.30	0.30	1.00	0.30	0.30	1.00
11426	SPDBVc	0.30	0.30	1.00	0.30	0.30	1.00	0.30	0.30	1.00	0.30	0.30	1.00
11427	SPDBVc	0.30	0.30	1.00	0.30	0.30	1.00	0.30	0.30	1.00	0.30	0.30	1.00
11428	SPDBVc	0.30	0.30	1.00	0.30	0.30	1.00	0.30	0.30	1.00	0.30	0.30	1.00
11429	SPDBVc	0.30	0.30	1.00	0.30	0.30	1.00	0.30	0.30	1.00	0.30	0.30	1.00
11430	SPDBVc	0.30	0.30	1.00	0.30	0.30	1.00	0.30	0.30	1.00	0.30	0.30	1.00
11431	SPDBVc	0.30	0.30	1.00	0.30	0.30	1.00	0.30	0.30	1.00	0.30	0.30	1.00
11432	SPDBVc	0.30	0.30	1.00	0.30	0.30	1.00	0.30	0.30	1.00	0.30	0.30	1.00
11433	SPDBVc	0.30	0.30	1.00	0.30	0.30	1.00	0.30	0.30	1.00	0.30	0.30	1.00
11434	SPDBVc	0.30	0.30	1.00	0.30	0.30	1.00	0.30	0.30	1.00	0.30	0.30	1.00
11435	SPDBVc	0.30	0.30	1.00	0.30	0.30	1.00	0.30	0.30	1.00	0.30	0.30	1.00
11436	SPDBVc	0.30	0.30	1.00	0.30	0.30	1.00	0.30	0.30	1.00	0.30	0.30	1.00
11437	SPDBVc	0.30	0.30	1.00	0.30	0.30	1.00	0.30	0.30	1.00	0.30	0.30	1.00
11438	SPDBVc	0.30	0.30	1.00	0.30	0.30	1.00	0.30	0.30	1.00	0.30	0.30	1.00
11439	SPDBVc	0.30	0.30	1.00	0.30	0.30	1.00	0.30	0.30	1.00	0.30	0.30	1.00
11440	SPDBVc	0.30	0.30	1.00	0.30	0.30	1.00	0.30	0.30	1.00	0.30	0.30	1.00
11441	SPDBVc	0.30	0.30	1.00	0.30	0.30	1.00	0.30	0.30	1.00	0.30	0.30	1.00
11442	SPDBVc	0.30	0.30	1.00	0.30	0.30	1.00	0.30	0.30	1.00	0.30	0.30	1.00
11443	SPDBVc	0.30	0.30	1.00	0.30	0.30	1.00	0.30	0.30	1.00	0.30	0.30	1.00
11444	SPDBVc	0.30	0.30	1.00	0.30	0.30	1.00	0.30	0.30	1.00	0.30	0.30	1.00
11445	SPDBVc	0.30	0.30	1.00	0.30	0.30	1.00	0.30	0.30	1.00	0.30	0.30	1.00
11446	SPDBVc	0.30	0.30	1.00	0.30	0.30	1.00	0.30	0.30	1.00	0.30	0.30	1.00
11447	SPDBVc	0.30	0.30	1.00	0.30	0.30	1.00	0.30	0.30	1.00	0.30	0.30	1.00
11448	SPDBVc	0.30	0.30	1.00	0.30	0.30	1.00	0.30	0.30	1.00	0.30	0.30	1.00
11449	SPDBVc	0.30	0.30	1.00	0.30	0.30	1.00	0.30	0.30	1.00	0.30	0.30	1.00
11450	SPDBVc	0.30	0.30	1.00	0.30	0.30	1.00	0.30	0.30	1.00	0.00	1.00	0.00
11451	SPDBVc	0.00	1.00	0.00	0.00	1.00	0.00	0.00	1.00	0.00	0.00	1.00	0.00
11452	SPDBVc	0.00	1.00	0.00	0.00	1.00	0.00	0.00	1.00	0.00	0.00	1.00	0.00
11453	SPDBVc	0.00	1.00	0.00	0.00	1.00	0.00	0.00	1.00	0.00	0.00	1.00	0.00
11454	SPDBVc	0.00	1.00	0.00	0.00	1.00	0.00	0.00	1.00	0.00	0.00	1.00	0.00
11455	SPDBVc	0.00	1.00	0.00	0.00	1.00	0.00	0.00	1.00	0.00	0.00	1.00	0.00
11456	SPDBVc	0.00	1.00	0.00	0.00	1.00	0.00	0.00	1.00	0.00	0.00	1.00	0.00
11457	SPDBVc	0.00	1.00	0.00	0.00	1.00	0.00	0.00	1.00	0.00	0.00	1.00	0.00
11458	SPDBVc	0.00	1.00	0.00	0.00	1.00	0.00	0.00	1.00	0.00	0.00	1.00	0.00
11459	SPDBVc	0.00	1.00	0.00	0.00	1.00	0.00	0.00	1.00	0.00	0.00	1.00	0.00
11460	SPDBVc	0.00	1.00	0.00	0.00	1.00	0.00	0.00	1.00	0.00	0.00	1.00	0.00
11461	SPDBVc	0.00	1.00	0.00	0.00	1.00	0.00	0.00	1.00	0.00	0.00	1.00	0.00
11462	SPDBVc	0.00	1.00	0.00	0.00	1.00	0.00	0.00	1.00	0.00	0.00	1.00	0.00
11463	SPDBVc	0.00	1.00	0.00	0.00	1.00	0.00	0.00	1.00	0.00	0.00	1.00	0.00
11464	SPDBVc	0.00	1.00	0.00	0.00	1.00	0.00	0.00	1.00	0.00	0.00	1.00	0.00

11465	SPDBVc	0.00	1.00	0.00	0.00	1.00	0.00	0.00	1.00	0.00	0.00	1.00	0.00
11466	SPDBVc	0.00	1.00	0.00	0.00	1.00	0.00	0.00	1.00	0.00	0.00	1.00	0.00
11467	SPDBVc	0.00	1.00	0.00	0.00	1.00	0.00	0.00	1.00	0.00	0.00	1.00	0.00
11468	SPDBVc	0.00	1.00	0.00	0.00	1.00	0.00	0.00	1.00	0.00	0.00	1.00	0.00
11469	SPDBVc	0.00	1.00	0.00	0.00	1.00	0.00	0.00	1.00	0.00	0.00	1.00	0.00
11470	SPDBVc	0.00	1.00	0.00	0.00	1.00	0.00	0.00	1.00	0.00	0.00	1.00	0.00
11471	SPDBVc	0.00	1.00	0.00	0.00	1.00	0.00	0.00	1.00	0.00	0.00	1.00	0.00
11472	SPDBVc	0.00	1.00	0.00	0.00	1.00	0.00	0.00	1.00	0.00	0.00	1.00	0.00
11473	SPDBVc	0.00	1.00	0.00	0.00	1.00	0.00	0.00	1.00	0.00	0.00	1.00	0.00
11474	SPDBVc	0.00	1.00	0.00	0.00	1.00	0.00	0.00	1.00	0.00	0.00	1.00	0.00
11475	SPDBVc	0.00	1.00	0.00	0.00	1.00	0.00	0.00	1.00	0.00	0.00	1.00	0.00
11476	SPDBVc	0.00	1.00	0.00	0.00	1.00	0.00	0.00	1.00	0.00	0.00	1.00	0.00
11477	SPDBVc	0.00	1.00	0.00	0.00	1.00	0.00	0.00	1.00	0.00	0.00	1.00	0.00
11478	SPDBVc	0.00	1.00	0.00	0.00	1.00	0.00	0.00	1.00	0.00	0.00	1.00	0.00
11479	SPDBVc	0.00	1.00	0.00	0.00	1.00	0.00	0.00	1.00	0.00	0.00	1.00	0.00
11480	SPDBVc	0.00	1.00	0.00	0.00	1.00	0.00	0.00	1.00	0.00	0.00	1.00	0.00
11481	SPDBVc	0.00	1.00	0.00	0.00	1.00	0.00	0.00	1.00	0.00	0.00	1.00	0.00
11482	SPDBVc	0.00	1.00	0.00	0.00	1.00	0.00	0.00	1.00	0.00	0.00	1.00	0.00
11483	SPDBVc	0.00	1.00	0.00	0.00	1.00	0.00	0.00	1.00	0.00	0.00	1.00	0.00
11484	SPDBVc	0.00	1.00	0.00	0.00	1.00	0.00	0.00	1.00	0.00	0.00	1.00	0.00
11485	SPDBVc	0.00	1.00	0.00	0.00	1.00	0.00	0.00	1.00	0.00	0.00	1.00	0.00
11486	SPDBVc	0.00	1.00	0.00	1.00	0.00	0.00	1.00	0.00	0.00	1.00	0.00	0.00
11487	SPDBVc	1.00	0.00	0.00	1.00	0.00	0.00	1.00	0.00	0.00	1.00	0.00	0.00
11488	SPDBVc	1.00	0.00	0.00	1.00	0.00	0.00	1.00	0.00	0.00	1.00	0.00	0.00
11489	SPDBVc	1.00	0.00	0.00	1.00	0.00	0.00	1.00	0.00	0.00	1.00	0.00	0.00
11490	SPDBVc	1.00	0.00	0.00	1.00	0.00	0.00	1.00	0.00	0.00	1.00	0.00	0.00
11491	SPDBVc	1.00	0.00	0.00	1.00	0.00	0.00	1.00	0.00	0.00	1.00	0.00	0.00
11492	SPDBVc	1.00	0.00	0.00	1.00	0.00	0.00	1.00	0.00	0.00	1.00	0.00	0.00
11493	SPDBVc	1.00	0.00	0.00	1.00	0.00	0.00	1.00	0.00	0.00	1.00	0.00	0.00
11494	SPDBVc	1.00	0.00	0.00	1.00	0.00	0.00	1.00	0.00	0.00	1.00	0.00	0.00
11495	SPDBVc	1.00	0.00	0.00	1.00	0.00	0.00	1.00	0.00	0.00	1.00	0.00	0.00
11496	SPDBVc	1.00	0.00	0.00	1.00	0.00	0.00	1.00	0.00	0.00	1.00	0.00	0.00
11497	SPDBVc	1.00	0.00	0.00	1.00	0.00	0.00	1.00	0.00	0.00	1.00	0.00	0.00
11498	SPDBVc	1.00	0.00	0.00	1.00	0.00	0.00	1.00	0.00	0.00	1.00	0.00	0.00
11499	SPDBVc	1.00	0.00	0.00	1.00	0.00	0.00	1.00	0.00	0.00	1.00	0.00	0.00
11500	SPDBVc	1.00	0.00	0.00	1.00	0.00	0.00	1.00	0.00	0.00	1.00	0.00	0.00
11501	SPDBVc	1.00	0.00	0.00	1.00	0.00	0.00	1.00	0.00	0.00	1.00	0.00	0.00
11502	SPDBVc	1.00	0.00	0.00	1.00	0.00	0.00	1.00	0.00	0.00	1.00	0.00	0.00
11503	SPDBVc	1.00	0.00	0.00	1.00	0.00	0.00	1.00	0.00	0.00	1.00	0.00	0.00
11504	SPDBVc	1.00	0.00	0.00	1.00	0.00	0.00	1.00	0.00	0.00	1.00	0.00	0.00

11505 SPDBVc 1.00 0.00 0.00 1.00 0.00 0.00 1.00 0.00 0.00 1.00 0.00 0.00
11506 SPDBVc 1.00 0.00 0.00 1.00 0.00 0.00 1.00 0.00 0.00 1.00 0.00 0.00
11507 SPDBVc 1.00 0.00 0.00 1.00 0.00 0.00 1.00 0.00 0.00 1.00 0.00 0.00
11508 SPDBVc 1.00 0.00 0.00 1.00 0.00 0.00 1.00 0.00 0.00 1.00 0.00 0.00
11509 SPDBVc 1.00 0.00 0.00 1.00 0.00 0.00 1.00 0.00 0.00 1.00 0.00 0.00
11510 SPDBVc 1.00 0.00 0.00 1.00 0.00 0.00 1.00 0.00 0.00 1.00 0.00 0.00
11511 SPDBVc 1.00 0.00 0.00 1.00 0.00 0.00 1.00 0.00 0.00 1.00 0.00 0.00
11512 SPDBVc 1.00 0.00 0.00 1.00 0.00 0.00 1.00 0.00 0.00 1.00 0.00 0.00
11513 SPDBVc 1.00 0.00 0.00 1.00 0.00 0.00 1.00 0.00 0.00 1.00 0.00 0.00
11514 SPDBVc 1.00 0.00 0.00 1.00 0.00 0.00 1.00 0.00 0.00 1.00 0.00 0.00
11515 SPDBVc 1.00 0.00 0.00 1.00 0.00 0.00 1.00 0.00 0.00 1.00 0.00 0.00
11516 SPDBVc 1.00 0.00 0.00 1.00 0.00 0.00 1.00 0.00 0.00 1.00 0.00 0.00
11517 SPDBVc 1.00 0.00 0.00 1.00 0.00 0.00 1.00 0.00 0.00 1.00 0.00 0.00
11518 SPDBVc 1.00 0.00 0.00 1.00 0.00 0.00 1.00 0.00 0.00 1.00 0.00 0.00
11519 SPDBVc 1.00 0.00 0.00 1.00 0.00 0.00 1.00 0.00 0.00 1.00 0.00 0.00
11520 SPDBVc 1.00 0.00 0.00 1.00 0.00 0.00 1.00 0.00 0.00 1.00 0.00 0.00
11521 SPDBVc 1.00 0.00 0.00 1.00 0.00 0.00 1.00 0.00 0.00 1.00 0.00 0.00
11522 SPDBVc 1.00 0.00 0.00 1.00 0.00 0.00 1.00 0.00 0.00
11523 SPDBVC 0.03 0.52 0.77 0.03 0.52 0.77 0.03 0.52 0.77 0.03 0.52 0.77
11524 SPDBVC 1.00 1.00 0.00 1.00 1.00 0.00 1.00 1.00 0.00 1.00 1.00 0.00
11525 SPDBVC 1.00 1.00 0.00 1.00 1.00 0.00 1.00 1.00 0.00 1.00 1.00 0.00
11526 SPDBVC 1.00 1.00 0.00 1.00 1.00 0.00 1.00 1.00 0.00 1.00 1.00 0.00
11527 SPDBVC 1.00 1.00 0.00 1.00 1.00 0.00 1.00 1.00 0.00 1.00 1.00 0.00
11528 SPDBVC 1.00 1.00 0.00 1.00 1.00 0.00 1.00 1.00 0.00 1.00 1.00 0.00
11529 SPDBVC 1.00 1.00 0.00 1.00 1.00 0.00 1.00 1.00 0.00 1.00 1.00 0.00
11530 SPDBVC 1.00 1.00 0.00 1.00 1.00 0.00 1.00 1.00 0.00 1.00 1.00 0.00
11531 SPDBVC 1.00 1.00 0.00 1.00 1.00 0.00 1.00 1.00 0.00 1.00 1.00 0.00
11532 SPDBVC 1.00 1.00 0.00 1.00 1.00 0.00 1.00 1.00 0.00 1.00 1.00 0.00
11533 SPDBVC 1.00 1.00 0.00 1.00 1.00 0.00 1.00 1.00 0.00 1.00 1.00 0.00
11534 SPDBVC 1.00 1.00 0.00 1.00 1.00 0.00 1.00 1.00 0.00 1.00 1.00 0.00
11535 SPDBVC 1.00 1.00 0.00 1.00 1.00 0.00 1.00 1.00 0.00 1.00 1.00 0.00
11536 SPDBVC 1.00 1.00 0.00 1.00 1.00 0.00 1.00 1.00 0.00 1.00 1.00 0.00
11537 SPDBVC 1.00 1.00 0.00 1.00 1.00 0.00 1.00 1.00 0.00 1.00 1.00 0.00
11538 SPDBVC 1.00 1.00 0.00 1.00 1.00 0.00 1.00 1.00 0.00 1.00 1.00 0.00
11539 SPDBVC 1.00 1.00 0.00 1.00 1.00 0.00 1.00 1.00 0.00 1.00 1.00 0.00
11540 SPDBVC 1.00 1.00 0.00 1.00 1.00 0.00 1.00 1.00 0.00 1.00 1.00 0.00
11541 SPDBVC 1.00 1.00 0.00 1.00 1.00 0.00 1.00 1.00 0.00 1.00 1.00 0.00
11542 SPDBVC 1.00 1.00 0.00 1.00 1.00 0.00 1.00 1.00 0.00 1.00 1.00 0.00
11543 SPDBVC 1.00 1.00 0.00 1.00 1.00 0.00 1.00 1.00 0.00 1.00 1.00 0.00
11544 SPDBVC 1.00 1.00 0.00 1.00 1.00 0.00 1.00 1.00 0.00 1.00 1.00 0.00

11545	SPDBVC	1.00	1.00	0.00	1.00	1.00	0.00	1.00	1.00	0.00	1.00	1.00	0.00
11546	SPDBVC	1.00	1.00	0.00	1.00	1.00	0.00	1.00	1.00	0.00	1.00	1.00	0.00
11547	SPDBVC	1.00	1.00	0.00	1.00	1.00	0.00	1.00	1.00	0.00	1.00	1.00	0.00
11548	SPDBVC	1.00	1.00	0.00	1.00	1.00	0.00	1.00	1.00	0.00	1.00	1.00	0.00
11549	SPDBVC	1.00	1.00	0.00	1.00	1.00	0.00	1.00	1.00	0.00	1.00	1.00	0.00
11550	SPDBVC	1.00	1.00	0.00	1.00	1.00	0.00	1.00	1.00	0.00	1.00	1.00	0.00
11551	SPDBVC	1.00	1.00	0.00	1.00	1.00	0.00	1.00	1.00	0.00	1.00	1.00	0.00
11552	SPDBVC	1.00	1.00	0.00	1.00	1.00	0.00	1.00	1.00	0.00	1.00	1.00	0.00
11553	SPDBVC	1.00	1.00	0.00	1.00	1.00	0.00	1.00	1.00	0.00	1.00	1.00	0.00
11554	SPDBVC	1.00	1.00	0.00	1.00	1.00	0.00	1.00	1.00	0.00	1.00	1.00	0.00
11555	SPDBVC	1.00	1.00	0.00	1.00	1.00	0.00	1.00	1.00	0.00	1.00	1.00	0.00
11556	SPDBVC	1.00	1.00	0.00	1.00	1.00	0.00	1.00	1.00	0.00	1.00	1.00	0.00
11557	SPDBVC	1.00	1.00	0.00	1.00	1.00	0.00	1.00	1.00	0.00	1.00	1.00	0.00
11558	SPDBVC	1.00	1.00	0.00	1.00	1.00	0.00	1.00	1.00	0.00	1.00	1.00	0.00
11559	SPDBVC	1.00	1.00	0.00	1.00	1.00	0.00	0.30	0.30	1.00	0.30	0.30	1.00
11560	SPDBVC	0.30	0.30	1.00	0.30	0.30	1.00	0.30	0.30	1.00	0.30	0.30	1.00
11561	SPDBVC	0.30	0.30	1.00	0.30	0.30	1.00	0.30	0.30	1.00	0.30	0.30	1.00
11562	SPDBVC	0.30	0.30	1.00	0.30	0.30	1.00	0.30	0.30	1.00	0.30	0.30	1.00
11563	SPDBVC	0.30	0.30	1.00	0.30	0.30	1.00	0.30	0.30	1.00	0.30	0.30	1.00
11564	SPDBVC	0.30	0.30	1.00	0.30	0.30	1.00	0.30	0.30	1.00	0.30	0.30	1.00
11565	SPDBVC	0.30	0.30	1.00	0.30	0.30	1.00	0.30	0.30	1.00	0.30	0.30	1.00
11566	SPDBVC	0.30	0.30	1.00	0.30	0.30	1.00	0.30	0.30	1.00	0.30	0.30	1.00
11567	SPDBVC	0.30	0.30	1.00	0.30	0.30	1.00	0.30	0.30	1.00	0.30	0.30	1.00
11568	SPDBVC	0.30	0.30	1.00	0.30	0.30	1.00	0.30	0.30	1.00	0.30	0.30	1.00
11569	SPDBVC	0.30	0.30	1.00	0.30	0.30	1.00	0.30	0.30	1.00	0.30	0.30	1.00
11570	SPDBVC	0.30	0.30	1.00	0.30	0.30	1.00	0.30	0.30	1.00	0.30	0.30	1.00
11571	SPDBVC	0.30	0.30	1.00	0.30	0.30	1.00	0.30	0.30	1.00	0.30	0.30	1.00
11572	SPDBVC	0.30	0.30	1.00	0.30	0.30	1.00	0.30	0.30	1.00	0.30	0.30	1.00
11573	SPDBVC	0.30	0.30	1.00	0.30	0.30	1.00	0.30	0.30	1.00	0.30	0.30	1.00
11574	SPDBVC	0.30	0.30	1.00	0.30	0.30	1.00	0.30	0.30	1.00	0.30	0.30	1.00
11575	SPDBVC	0.30	0.30	1.00	0.30	0.30	1.00	0.30	0.30	1.00	0.30	0.30	1.00
11576	SPDBVC	0.30	0.30	1.00	0.30	0.30	1.00	0.30	0.30	1.00	0.30	0.30	1.00
11577	SPDBVC	0.30	0.30	1.00	0.30	0.30	1.00	0.30	0.30	1.00	0.30	0.30	1.00
11578	SPDBVC	0.30	0.30	1.00	0.30	0.30	1.00	0.30	0.30	1.00	0.30	0.30	1.00
11579	SPDBVC	0.30	0.30	1.00	0.30	0.30	1.00	0.30	0.30	1.00	0.30	0.30	1.00
11580	SPDBVC	0.30	0.30	1.00	0.30	0.30	1.00	0.30	0.30	1.00	0.30	0.30	1.00
11581	SPDBVC	0.30	0.30	1.00	0.30	0.30	1.00	0.30	0.30	1.00	0.30	0.30	1.00
11582	SPDBVC	0.30	0.30	1.00	0.30	0.30	1.00	0.30	0.30	1.00	0.30	0.30	1.00
11583	SPDBVC	0.30	0.30	1.00	0.30	0.30	1.00	0.30	0.30	1.00	0.30	0.30	1.00
11584	SPDBVC	0.30	0.30	1.00	0.30	0.30	1.00	0.30	0.30	1.00	0.30	0.30	1.00

11585	SPDBVC	0.30	0.30	1.00	0.30	0.30	1.00	0.30	0.30	1.00	0.30	0.30	1.00
11586	SPDBVC	0.30	0.30	1.00	0.30	0.30	1.00	0.30	0.30	1.00	0.30	0.30	1.00
11587	SPDBVC	0.30	0.30	1.00	0.30	0.30	1.00	0.30	0.30	1.00	0.30	0.30	1.00
11588	SPDBVC	0.30	0.30	1.00	0.30	0.30	1.00	0.30	0.30	1.00	0.30	0.30	1.00
11589	SPDBVC	0.30	0.30	1.00	0.30	0.30	1.00	0.30	0.30	1.00	0.30	0.30	1.00
11590	SPDBVC	0.30	0.30	1.00	0.30	0.30	1.00	0.30	0.30	1.00	0.30	0.30	1.00
11591	SPDBVC	0.30	0.30	1.00	0.30	0.30	1.00	0.30	0.30	1.00	0.30	0.30	1.00
11592	SPDBVC	0.30	0.30	1.00	0.30	0.30	1.00	0.30	0.30	1.00	0.30	0.30	1.00
11593	SPDBVC	0.30	0.30	1.00	0.30	0.30	1.00	0.30	0.30	1.00	0.30	0.30	1.00
11594	SPDBVC	0.30	0.30	1.00	0.30	0.30	1.00	0.30	0.30	1.00	0.30	0.30	1.00
11595	SPDBVC	0.30	0.30	1.00	0.30	0.30	1.00	0.30	0.30	1.00	0.00	1.00	0.00
11596	SPDBVC	0.00	1.00	0.00	0.00	1.00	0.00	0.00	1.00	0.00	0.00	1.00	0.00
11597	SPDBVC	0.00	1.00	0.00	0.00	1.00	0.00	0.00	1.00	0.00	0.00	1.00	0.00
11598	SPDBVC	0.00	1.00	0.00	0.00	1.00	0.00	0.00	1.00	0.00	0.00	1.00	0.00
11599	SPDBVC	0.00	1.00	0.00	0.00	1.00	0.00	0.00	1.00	0.00	0.00	1.00	0.00
11600	SPDBVC	0.00	1.00	0.00	0.00	1.00	0.00	0.00	1.00	0.00	0.00	1.00	0.00
11601	SPDBVC	0.00	1.00	0.00	0.00	1.00	0.00	0.00	1.00	0.00	0.00	1.00	0.00
11602	SPDBVC	0.00	1.00	0.00	0.00	1.00	0.00	0.00	1.00	0.00	0.00	1.00	0.00
11603	SPDBVC	0.00	1.00	0.00	0.00	1.00	0.00	0.00	1.00	0.00	0.00	1.00	0.00
11604	SPDBVC	0.00	1.00	0.00	0.00	1.00	0.00	0.00	1.00	0.00	0.00	1.00	0.00
11605	SPDBVC	0.00	1.00	0.00	0.00	1.00	0.00	0.00	1.00	0.00	0.00	1.00	0.00
11606	SPDBVC	0.00	1.00	0.00	0.00	1.00	0.00	0.00	1.00	0.00	0.00	1.00	0.00
11607	SPDBVC	0.00	1.00	0.00	0.00	1.00	0.00	0.00	1.00	0.00	0.00	1.00	0.00
11608	SPDBVC	0.00	1.00	0.00	0.00	1.00	0.00	0.00	1.00	0.00	0.00	1.00	0.00
11609	SPDBVC	0.00	1.00	0.00	0.00	1.00	0.00	0.00	1.00	0.00	0.00	1.00	0.00
11610	SPDBVC	0.00	1.00	0.00	0.00	1.00	0.00	0.00	1.00	0.00	0.00	1.00	0.00
11611	SPDBVC	0.00	1.00	0.00	0.00	1.00	0.00	0.00	1.00	0.00	0.00	1.00	0.00
11612	SPDBVC	0.00	1.00	0.00	0.00	1.00	0.00	0.00	1.00	0.00	0.00	1.00	0.00
11613	SPDBVC	0.00	1.00	0.00	0.00	1.00	0.00	0.00	1.00	0.00	0.00	1.00	0.00
11614	SPDBVC	0.00	1.00	0.00	0.00	1.00	0.00	0.00	1.00	0.00	0.00	1.00	0.00
11615	SPDBVC	0.00	1.00	0.00	0.00	1.00	0.00	0.00	1.00	0.00	0.00	1.00	0.00
11616	SPDBVC	0.00	1.00	0.00	0.00	1.00	0.00	0.00	1.00	0.00	0.00	1.00	0.00
11617	SPDBVC	0.00	1.00	0.00	0.00	1.00	0.00	0.00	1.00	0.00	0.00	1.00	0.00
11618	SPDBVC	0.00	1.00	0.00	0.00	1.00	0.00	0.00	1.00	0.00	0.00	1.00	0.00
11619	SPDBVC	0.00	1.00	0.00	0.00	1.00	0.00	0.00	1.00	0.00	0.00	1.00	0.00
11620	SPDBVC	0.00	1.00	0.00	0.00	1.00	0.00	0.00	1.00	0.00	0.00	1.00	0.00
11621	SPDBVC	0.00	1.00	0.00	0.00	1.00	0.00	0.00	1.00	0.00	0.00	1.00	0.00
11622	SPDBVC	0.00	1.00	0.00	0.00	1.00	0.00	0.00	1.00	0.00	0.00	1.00	0.00
11623	SPDBVC	0.00	1.00	0.00	0.00	1.00	0.00	0.00	1.00	0.00	0.00	1.00	0.00
11624	SPDBVC	0.00	1.00	0.00	0.00	1.00	0.00	0.00	1.00	0.00	0.00	1.00	0.00

11625 SPDBVC 0.00 1.00 0.00 0.00 1.00 0.00 0.00 1.00 0.00 0.00 1.00 0.00
11626 SPDBVC 0.00 1.00 0.00 0.00 1.00 0.00 0.00 1.00 0.00 0.00 1.00 0.00
11627 SPDBVC 0.00 1.00 0.00 0.00 1.00 0.00 0.00 1.00 0.00 0.00 1.00 0.00
11628 SPDBVC 0.00 1.00 0.00 0.00 1.00 0.00 0.00 1.00 0.00 0.00 1.00 0.00
11629 SPDBVC 0.00 1.00 0.00 0.00 1.00 0.00 0.00 1.00 0.00 0.00 1.00 0.00
11630 SPDBVC 0.00 1.00 0.00 0.00 1.00 0.00 0.00 1.00 0.00 0.00 1.00 0.00
11631 SPDBVC 0.00 1.00 0.00 1.00 0.00 0.00 1.00 0.00 0.00 1.00 0.00 0.00
11632 SPDBVC 1.00 0.00 0.00 1.00 0.00 0.00 1.00 0.00 0.00 1.00 0.00 0.00
11633 SPDBVC 1.00 0.00 0.00 1.00 0.00 0.00 1.00 0.00 0.00 1.00 0.00 0.00
11634 SPDBVC 1.00 0.00 0.00 1.00 0.00 0.00 1.00 0.00 0.00 1.00 0.00 0.00
11635 SPDBVC 1.00 0.00 0.00 1.00 0.00 0.00 1.00 0.00 0.00 1.00 0.00 0.00
11636 SPDBVC 1.00 0.00 0.00 1.00 0.00 0.00 1.00 0.00 0.00 1.00 0.00 0.00
11637 SPDBVC 1.00 0.00 0.00 1.00 0.00 0.00 1.00 0.00 0.00 1.00 0.00 0.00
11638 SPDBVC 1.00 0.00 0.00 1.00 0.00 0.00 1.00 0.00 0.00 1.00 0.00 0.00
11639 SPDBVC 1.00 0.00 0.00 1.00 0.00 0.00 1.00 0.00 0.00 1.00 0.00 0.00
11640 SPDBVC 1.00 0.00 0.00 1.00 0.00 0.00 1.00 0.00 0.00 1.00 0.00 0.00
11641 SPDBVC 1.00 0.00 0.00 1.00 0.00 0.00 1.00 0.00 0.00 1.00 0.00 0.00
11642 SPDBVC 1.00 0.00 0.00 1.00 0.00 0.00 1.00 0.00 0.00 1.00 0.00 0.00
11643 SPDBVC 1.00 0.00 0.00 1.00 0.00 0.00 1.00 0.00 0.00 1.00 0.00 0.00
11644 SPDBVC 1.00 0.00 0.00 1.00 0.00 0.00 1.00 0.00 0.00 1.00 0.00 0.00
11645 SPDBVC 1.00 0.00 0.00 1.00 0.00 0.00 1.00 0.00 0.00 1.00 0.00 0.00
11646 SPDBVC 1.00 0.00 0.00 1.00 0.00 0.00 1.00 0.00 0.00 1.00 0.00 0.00
11647 SPDBVC 1.00 0.00 0.00 1.00 0.00 0.00 1.00 0.00 0.00 1.00 0.00 0.00
11648 SPDBVC 1.00 0.00 0.00 1.00 0.00 0.00 1.00 0.00 0.00 1.00 0.00 0.00
11649 SPDBVC 1.00 0.00 0.00 1.00 0.00 0.00 1.00 0.00 0.00 1.00 0.00 0.00
11650 SPDBVC 1.00 0.00 0.00 1.00 0.00 0.00 1.00 0.00 0.00 1.00 0.00 0.00
11651 SPDBVC 1.00 0.00 0.00 1.00 0.00 0.00 1.00 0.00 0.00 1.00 0.00 0.00
11652 SPDBVC 1.00 0.00 0.00 1.00 0.00 0.00 1.00 0.00 0.00 1.00 0.00 0.00
11653 SPDBVC 1.00 0.00 0.00 1.00 0.00 0.00 1.00 0.00 0.00 1.00 0.00 0.00
11654 SPDBVC 1.00 0.00 0.00 1.00 0.00 0.00 1.00 0.00 0.00 1.00 0.00 0.00
11655 SPDBVC 1.00 0.00 0.00 1.00 0.00 0.00 1.00 0.00 0.00 1.00 0.00 0.00
11656 SPDBVC 1.00 0.00 0.00 1.00 0.00 0.00 1.00 0.00 0.00 1.00 0.00 0.00
11657 SPDBVC 1.00 0.00 0.00 1.00 0.00 0.00 1.00 0.00 0.00 1.00 0.00 0.00
11658 SPDBVC 1.00 0.00 0.00 1.00 0.00 0.00 1.00 0.00 0.00 1.00 0.00 0.00
11659 SPDBVC 1.00 0.00 0.00 1.00 0.00 0.00 1.00 0.00 0.00 1.00 0.00 0.00
11660 SPDBVC 1.00 0.00 0.00 1.00 0.00 0.00 1.00 0.00 0.00 1.00 0.00 0.00
11661 SPDBVC 1.00 0.00 0.00 1.00 0.00 0.00 1.00 0.00 0.00 1.00 0.00 0.00
11662 SPDBVC 1.00 0.00 0.00 1.00 0.00 0.00 1.00 0.00 0.00 1.00 0.00 0.00
11663 SPDBVC 1.00 0.00 0.00 1.00 0.00 0.00 1.00 0.00 0.00 1.00 0.00 0.00
11664 SPDBVC 1.00 0.00 0.00 1.00 0.00 0.00 1.00 0.00 0.00 1.00 0.00 0.00

11665 SPDBVC 1.00 0.00 0.00 1.00 0.00 0.00 1.00 0.00 0.00 1.00 0.00 0.00
11666 SPDBVC 1.00 0.00 0.00 1.00 0.00 0.00 1.00 0.00 0.00 1.00 0.00 0.00
11667 SPDBVC 1.00 0.00 0.00 1.00 0.00 0.00 1.00 0.00 0.00
11668 SPDBVR 0.00 0.00 0.80 0.00 0.00 0.80 0.00 0.00 0.80 0.00 0.00 0.80
11669 SPDBVR 0.74 0.26 0.32 0.74 0.26 0.32 0.74 0.26 0.32 0.74 0.26 0.32
11670 SPDBVR 0.74 0.26 0.32 0.74 0.26 0.32 0.74 0.26 0.32 0.74 0.26 0.32
11671 SPDBVR 0.74 0.26 0.32 0.74 0.26 0.32 0.74 0.26 0.32 0.74 0.26 0.32
11672 SPDBVR 0.74 0.26 0.32 0.74 0.26 0.32 0.74 0.26 0.32 0.74 0.26 0.32
11673 SPDBVR 0.74 0.26 0.32 0.74 0.26 0.32 0.16 1.00 0.04 0.74 0.26 0.32
11674 SPDBVR 0.74 0.26 0.32 0.74 0.26 0.32 0.74 0.26 0.32 0.74 0.26 0.32
11675 SPDBVR 0.74 0.26 0.32 0.12 1.00 0.00 0.74 0.26 0.32 0.74 0.26 0.32
11676 SPDBVR 0.74 0.26 0.32 0.74 0.26 0.32 0.74 0.26 0.32 0.74 0.26 0.32
11677 SPDBVR 0.74 0.26 0.32 0.74 0.26 0.32 0.74 0.26 0.32 0.74 0.26 0.32
11678 SPDBVR 0.74 0.26 0.32 0.74 0.26 0.32 0.74 0.26 0.32 0.74 0.26 0.32
11679 SPDBVR 0.74 0.26 0.32 0.74 0.26 0.32 0.74 0.26 0.32 0.74 0.26 0.32
11680 SPDBVR 0.74 0.26 0.32 0.74 0.26 0.32 0.74 0.26 0.32 0.74 0.26 0.32
11681 SPDBVR 0.74 0.26 0.32 0.74 0.26 0.32 0.74 0.26 0.32 0.74 0.26 0.32
11682 SPDBVR 0.74 0.26 0.32 0.07 1.00 0.02 0.74 0.26 0.32 0.74 0.26 0.32
11683 SPDBVR 0.74 0.26 0.32 0.74 0.26 0.32 0.74 0.26 0.32 0.74 0.26 0.32
11684 SPDBVR 0.74 0.26 0.32 0.22 1.00 0.07 0.74 0.26 0.32 0.74 0.26 0.32
11685 SPDBVR 0.74 0.26 0.32 0.74 0.26 0.32 0.74 0.26 0.32 0.74 0.26 0.32
11686 SPDBVR 0.74 0.26 0.32 0.74 0.26 0.32 0.74 0.26 0.32 0.16 1.00 0.09
11687 SPDBVR 0.74 0.26 0.32 0.74 0.26 0.32 0.74 0.26 0.32 0.74 0.26 0.32
11688 SPDBVR 0.74 0.26 0.32 0.74 0.26 0.32 0.74 0.26 0.32 0.74 0.26 0.32
11689 SPDBVR 0.74 0.26 0.32 0.74 0.26 0.32 0.21 1.00 0.00 0.74 0.26 0.32
11690 SPDBVR 0.74 0.26 0.32 0.74 0.26 0.32 0.74 0.26 0.32 0.74 0.26 0.32
11691 SPDBVR 0.74 0.26 0.32 0.74 0.26 0.32 0.74 0.26 0.32 0.74 0.26 0.32
11692 SPDBVR 0.74 0.26 0.32 0.74 0.26 0.32 0.74 0.26 0.32 0.74 0.26 0.32
11693 SPDBVR 0.74 0.26 0.32 0.74 0.26 0.32 0.74 0.26 0.32 0.74 0.26 0.32
11694 SPDBVR 0.74 0.26 0.32 0.74 0.26 0.32 0.74 0.26 0.32 0.74 0.26 0.32
11695 SPDBVR 0.74 0.26 0.32 0.74 0.26 0.32 0.74 0.26 0.32 0.74 0.26 0.32
11696 SPDBVR 0.74 0.26 0.32 0.74 0.26 0.32 0.74 0.26 0.32 0.74 0.26 0.32
11697 SPDBVR 0.74 0.26 0.32 0.74 0.26 0.32 0.74 0.26 0.32 0.74 0.26 0.32
11698 SPDBVR 0.74 0.26 0.32 0.74 0.26 0.32 0.74 0.26 0.32 0.74 0.26 0.32
11699 SPDBVR 0.02 1.00 0.00 0.74 0.26 0.32 0.74 0.26 0.32 0.74 0.26 0.32
11700 SPDBVR 0.74 0.26 0.32 0.74 0.26 0.32 0.74 0.26 0.32 0.74 0.26 0.32
11701 SPDBVR 0.74 0.26 0.32 0.74 0.26 0.32 0.74 0.26 0.32 0.74 0.26 0.32
11702 SPDBVR 0.74 0.26 0.32 0.74 0.26 0.32 0.74 0.26 0.32 0.74 0.26 0.32
11703 SPDBVR 0.74 0.26 0.32 0.74 0.26 0.32 0.74 0.26 0.32 0.74 0.26 0.32
11704 SPDBVR 0.74 0.26 0.32 0.74 0.26 0.32 0.62 0.58 0.58 0.62 0.58 0.58

11705 SPDBVR 0.62 0.58 0.58 0.62 0.58 0.58 0.62 0.58 0.58 0.62 0.58 0.58
11706 SPDBVR 0.62 0.58 0.58 0.62 0.58 0.58 0.62 0.58 0.58 0.62 0.58 0.58
11707 SPDBVR 0.62 0.58 0.58 0.62 0.58 0.58 0.62 0.58 0.58 0.62 0.58 0.58
11708 SPDBVR 0.62 0.58 0.58 0.62 0.58 0.58 0.62 0.58 0.58 0.62 0.58 0.58
11709 SPDBVR 0.62 0.58 0.58 0.62 0.58 0.58 0.62 0.58 0.58 0.62 0.58 0.58
11710 SPDBVR 0.62 0.58 0.58 0.62 0.58 0.58 0.62 0.58 0.58 0.62 0.58 0.58
11711 SPDBVR 0.62 0.58 0.58 0.62 0.58 0.58 0.62 0.58 0.58 0.62 0.58 0.58
11712 SPDBVR 0.62 0.58 0.58 0.62 0.58 0.58 0.62 0.58 0.58 0.62 0.58 0.58
11713 SPDBVR 0.62 0.58 0.58 0.62 0.58 0.58 0.62 0.58 0.58 0.62 0.58 0.58
11714 SPDBVR 0.62 0.58 0.58 0.62 0.58 0.58 0.62 0.58 0.58 0.62 0.58 0.58
11715 SPDBVR 0.62 0.58 0.58 0.62 0.58 0.58 0.62 0.58 0.58 0.62 0.58 0.58
11716 SPDBVR 0.62 0.58 0.58 0.62 0.58 0.58 0.62 0.58 0.58 0.62 0.58 0.58
11717 SPDBVR 0.62 0.58 0.58 0.62 0.58 0.58 0.62 0.58 0.58 0.62 0.58 0.58
11718 SPDBVR 0.62 0.58 0.58 0.62 0.58 0.58 0.62 0.58 0.58 0.62 0.58 0.58
11719 SPDBVR 0.62 0.58 0.58 0.62 0.58 0.58 0.62 0.58 0.58 0.62 0.58 0.58
11720 SPDBVR 0.62 0.58 0.58 0.62 0.58 0.58 0.62 0.58 0.58 0.62 0.58 0.58
11721 SPDBVR 0.62 0.58 0.58 0.62 0.58 0.58 0.62 0.58 0.58 0.62 0.58 0.58
11722 SPDBVR 0.62 0.58 0.58 0.62 0.58 0.58 0.62 0.58 0.58 0.62 0.58 0.58
11723 SPDBVR 0.62 0.58 0.58 0.62 0.58 0.58 0.62 0.58 0.58 0.62 0.58 0.58
11724 SPDBVR 0.62 0.58 0.58 0.62 0.58 0.58 0.62 0.58 0.58 0.62 0.58 0.58
11725 SPDBVR 0.62 0.58 0.58 0.62 0.58 0.58 0.62 0.58 0.58 0.62 0.58 0.58
11726 SPDBVR 0.62 0.58 0.58 0.62 0.58 0.58 0.62 0.58 0.58 0.62 0.58 0.58
11727 SPDBVR 0.62 0.58 0.58 0.62 0.58 0.58 0.62 0.58 0.58 0.62 0.58 0.58
11728 SPDBVR 0.62 0.58 0.58 0.62 0.58 0.58 0.62 0.58 0.58 0.62 0.58 0.58
11729 SPDBVR 0.62 0.58 0.58 0.62 0.58 0.58 0.62 0.58 0.58 0.62 0.58 0.58
11730 SPDBVR 0.62 0.58 0.58 0.62 0.58 0.58 0.62 0.58 0.58 0.62 0.58 0.58
11731 SPDBVR 0.62 0.58 0.58 0.62 0.58 0.58 0.62 0.58 0.58 0.62 0.58 0.58
11732 SPDBVR 0.62 0.58 0.58 0.62 0.58 0.58 0.62 0.58 0.58 0.62 0.58 0.58
11733 SPDBVR 0.62 0.58 0.58 0.62 0.58 0.58 0.62 0.58 0.58 0.62 0.58 0.58
11734 SPDBVR 0.62 0.58 0.58 0.62 0.58 0.58 0.62 0.58 0.58 0.62 0.58 0.58
11735 SPDBVR 0.62 0.58 0.58 0.62 0.58 0.58 0.62 0.58 0.58 0.62 0.58 0.58
11736 SPDBVR 0.62 0.58 0.58 0.62 0.58 0.58 0.62 0.58 0.58 0.62 0.58 0.58
11737 SPDBVR 0.62 0.58 0.58 0.62 0.58 0.58 0.62 0.58 0.58 0.62 0.58 0.58
11738 SPDBVR 0.62 0.58 0.58 0.62 0.58 0.58 0.62 0.58 0.58 0.62 0.58 0.58
11739 SPDBVR 0.62 0.58 0.58 0.62 0.58 0.58 0.62 0.58 0.58 0.62 0.58 0.58
11740 SPDBVR 0.62 0.58 0.58 0.62 0.58 0.58 0.62 0.58 0.58 0.74 0.26 0.32
11741 SPDBVR 0.74 0.26 0.32 0.74 0.26 0.32 0.74 0.26 0.32 0.74 0.26 0.32
11742 SPDBVR 0.74 0.26 0.32 0.74 0.26 0.32 0.74 0.26 0.32 0.74 0.26 0.32
11743 SPDBVR 0.74 0.26 0.32 0.74 0.26 0.32 0.74 0.26 0.32 0.74 0.26 0.32
11744 SPDBVR 0.74 0.26 0.32 0.74 0.26 0.32 0.74 0.26 0.32 0.74 0.26 0.32

11745 SPDBVR 0.74 0.26 0.32 0.13 1.00 0.02 0.90 0.35 0.34 0.74 0.26 0.32
11746 SPDBVR 0.74 0.26 0.32 0.74 0.26 0.32 0.74 0.26 0.32 0.74 0.26 0.32
11747 SPDBVR 0.14 1.00 0.08 0.89 0.40 0.37 0.74 0.26 0.32 0.74 0.26 0.32
11748 SPDBVR 0.74 0.26 0.32 0.74 0.26 0.32 0.74 0.26 0.32 0.74 0.26 0.32
11749 SPDBVR 0.74 0.26 0.32 0.74 0.26 0.32 0.74 0.26 0.32 0.74 0.26 0.32
11750 SPDBVR 0.74 0.26 0.32 0.74 0.26 0.32 0.74 0.26 0.32 0.74 0.26 0.32
11751 SPDBVR 0.74 0.26 0.32 0.74 0.26 0.32 0.74 0.26 0.32 0.74 0.26 0.32
11752 SPDBVR 0.74 0.26 0.32 0.74 0.26 0.32 0.74 0.26 0.32 0.74 0.26 0.32
11753 SPDBVR 0.74 0.26 0.32 0.74 0.26 0.32 0.74 0.26 0.32 0.74 0.26 0.32
11754 SPDBVR 0.13 1.00 0.02 0.74 0.26 0.32 0.74 0.26 0.32 0.74 0.26 0.32
11755 SPDBVR 0.74 0.26 0.32 0.74 0.26 0.32 0.74 0.26 0.32 0.74 0.26 0.32
11756 SPDBVR 0.09 1.00 0.00 0.95 0.36 0.39 0.74 0.26 0.32 0.74 0.26 0.32
11757 SPDBVR 0.74 0.26 0.32 0.74 0.26 0.32 0.74 0.26 0.32 0.74 0.26 0.32
11758 SPDBVR 0.74 0.26 0.32 0.74 0.26 0.32 0.18 1.00 0.05 0.91 0.41 0.35
11759 SPDBVR 0.74 0.26 0.32 0.74 0.26 0.32 0.74 0.26 0.32 0.74 0.26 0.32
11760 SPDBVR 0.74 0.26 0.32 0.74 0.26 0.32 0.74 0.26 0.32 0.74 0.26 0.32
11761 SPDBVR 0.74 0.26 0.32 0.13 1.00 0.05 0.74 0.26 0.32 0.74 0.26 0.32
11762 SPDBVR 0.74 0.26 0.32 0.74 0.26 0.32 0.74 0.26 0.32 0.74 0.26 0.32
11763 SPDBVR 0.74 0.26 0.32 0.74 0.26 0.32 0.74 0.26 0.32 0.74 0.26 0.32
11764 SPDBVR 0.74 0.26 0.32 0.74 0.26 0.32 0.74 0.26 0.32 0.74 0.26 0.32
11765 SPDBVR 0.74 0.26 0.32 0.74 0.26 0.32 0.74 0.26 0.32 0.74 0.26 0.32
11766 SPDBVR 0.74 0.26 0.32 0.74 0.26 0.32 0.74 0.26 0.32 0.74 0.26 0.32
11767 SPDBVR 0.74 0.26 0.32 0.74 0.26 0.32 0.74 0.26 0.32 0.74 0.26 0.32
11768 SPDBVR 0.74 0.26 0.32 0.74 0.26 0.32 0.74 0.26 0.32 0.74 0.26 0.32
11769 SPDBVR 0.74 0.26 0.32 0.74 0.26 0.32 0.74 0.26 0.32 0.74 0.26 0.32
11770 SPDBVR 0.74 0.26 0.32 0.74 0.26 0.32 0.74 0.26 0.32 0.13 1.00 0.00
11771 SPDBVR 0.74 0.26 0.32 0.74 0.26 0.32 0.74 0.26 0.32 0.74 0.26 0.32
11772 SPDBVR 0.74 0.26 0.32 0.74 0.26 0.32 0.74 0.26 0.32 0.74 0.26 0.32
11773 SPDBVR 0.74 0.26 0.32 0.74 0.26 0.32 0.74 0.26 0.32 0.74 0.26 0.32
11774 SPDBVR 0.74 0.26 0.32 0.74 0.26 0.32 0.74 0.26 0.32 0.74 0.26 0.32
11775 SPDBVR 0.74 0.26 0.32 0.74 0.26 0.32 0.74 0.26 0.32 0.74 0.26 0.32
11776 SPDBVR 0.74 0.26 0.32 0.62 0.58 0.58 0.62 0.58 0.58 0.62 0.58 0.58
11777 SPDBVR 0.62 0.58 0.58 0.62 0.58 0.58 0.62 0.58 0.58 0.62 0.58 0.58
11778 SPDBVR 0.62 0.58 0.58 0.62 0.58 0.58 0.62 0.58 0.58 0.62 0.58 0.58
11779 SPDBVR 0.62 0.58 0.58 0.62 0.58 0.58 0.62 0.58 0.58 0.62 0.58 0.58
11780 SPDBVR 0.62 0.58 0.58 0.62 0.58 0.58 0.62 0.58 0.58 0.62 0.58 0.58
11781 SPDBVR 0.62 0.58 0.58 0.62 0.58 0.58 0.62 0.58 0.58 0.62 0.58 0.58
11782 SPDBVR 0.62 0.58 0.58 0.62 0.58 0.58 0.62 0.58 0.58 0.62 0.58 0.58
11783 SPDBVR 0.62 0.58 0.58 0.62 0.58 0.58 0.62 0.58 0.58 0.62 0.58 0.58
11784 SPDBVR 0.62 0.58 0.58 0.62 0.58 0.58 0.62 0.58 0.58 0.62 0.58 0.58

11785 SPDBVR 0.62 0.58 0.58 0.62 0.58 0.58 0.62 0.58 0.58 0.62 0.58 0.58
11786 SPDBVR 0.62 0.58 0.58 0.62 0.58 0.58 0.62 0.58 0.58 0.62 0.58 0.58
11787 SPDBVR 0.62 0.58 0.58 0.62 0.58 0.58 0.62 0.58 0.58 0.62 0.58 0.58
11788 SPDBVR 0.62 0.58 0.58 0.62 0.58 0.58 0.62 0.58 0.58 0.62 0.58 0.58
11789 SPDBVR 0.62 0.58 0.58 0.62 0.58 0.58 0.62 0.58 0.58 0.62 0.58 0.58
11790 SPDBVR 0.62 0.58 0.58 0.62 0.58 0.58 0.62 0.58 0.58 0.62 0.58 0.58
11791 SPDBVR 0.62 0.58 0.58 0.62 0.58 0.58 0.62 0.58 0.58 0.62 0.58 0.58
11792 SPDBVR 0.62 0.58 0.58 0.62 0.58 0.58 0.62 0.58 0.58 0.62 0.58 0.58
11793 SPDBVR 0.62 0.58 0.58 0.62 0.58 0.58 0.62 0.58 0.58 0.62 0.58 0.58
11794 SPDBVR 0.62 0.58 0.58 0.62 0.58 0.58 0.62 0.58 0.58 0.62 0.58 0.58
11795 SPDBVR 0.62 0.58 0.58 0.62 0.58 0.58 0.62 0.58 0.58 0.62 0.58 0.58
11796 SPDBVR 0.62 0.58 0.58 0.62 0.58 0.58 0.62 0.58 0.58 0.62 0.58 0.58
11797 SPDBVR 0.62 0.58 0.58 0.62 0.58 0.58 0.62 0.58 0.58 0.62 0.58 0.58
11798 SPDBVR 0.62 0.58 0.58 0.62 0.58 0.58 0.62 0.58 0.58 0.62 0.58 0.58
11799 SPDBVR 0.62 0.58 0.58 0.62 0.58 0.58 0.62 0.58 0.58 0.62 0.58 0.58
11800 SPDBVR 0.62 0.58 0.58 0.62 0.58 0.58 0.62 0.58 0.58 0.62 0.58 0.58
11801 SPDBVR 0.62 0.58 0.58 0.62 0.58 0.58 0.62 0.58 0.58 0.62 0.58 0.58
11802 SPDBVR 0.62 0.58 0.58 0.62 0.58 0.58 0.62 0.58 0.58 0.62 0.58 0.58
11803 SPDBVR 0.62 0.58 0.58 0.62 0.58 0.58 0.62 0.58 0.58 0.62 0.58 0.58
11804 SPDBVR 0.62 0.58 0.58 0.62 0.58 0.58 0.62 0.58 0.58 0.62 0.58 0.58
11805 SPDBVR 0.62 0.58 0.58 0.62 0.58 0.58 0.62 0.58 0.58 0.62 0.58 0.58
11806 SPDBVR 0.62 0.58 0.58 0.62 0.58 0.58 0.62 0.58 0.58 0.62 0.58 0.58
11807 SPDBVR 0.62 0.58 0.58 0.62 0.58 0.58 0.62 0.58 0.58 0.62 0.58 0.58
11808 SPDBVR 0.62 0.58 0.58 0.62 0.58 0.58 0.62 0.58 0.58 0.62 0.58 0.58
11809 SPDBVR 0.62 0.58 0.58 0.62 0.58 0.58 0.62 0.58 0.58 0.62 0.58 0.58
11810 SPDBVR 0.62 0.58 0.58 0.62 0.58 0.58 0.62 0.58 0.58 0.62 0.58 0.58
11811 SPDBVR 0.62 0.58 0.58 0.62 0.58 0.58 0.62 0.58 0.58 0.62 0.58 0.58
11812 SPDBVR 0.62 0.58 0.58 0.62 0.58 0.58 0.62 0.58 0.58
11813 SPDBVb 1.00 1.00 1.00
11814 SPDBVg 64 64 64 64 64 64 64 64 64 64
11815 SPDBVg 64 64 64 64 64 64 64 64 64 64
11816 SPDBVg 64 64 64 64 64 64 64 64 64 64
11817 SPDBVg 64 64 64 64 64 64 64 64 64 64
11818 SPDBVg 64 64 64 64 64 64 64 64 64 64
11819 SPDBVg 64 64 64 64 64 64 64 64 64 64
11820 SPDBVg 64 64 64 64 64 64 64 64 64 64
11821 SPDBVg 64 64 64 64 64 64 64 64 64 64
11822 SPDBVg 64 64 64 64 64 64 64 64 64 64
11823 SPDBVg 64 64 64 64 64 64 64 64 64 64
11824 SPDBVg 64 64 64 64 64 64 64 64 64 64

```
SPDBVg    64    64    64    64    64    64    64    64    64    64
SPDBVg    64    64    64    64    64    64    64    64    64    64
SPDBVg    64    64    64    64    64    64    64    64    64    64
SPDBVg    64    64    64    64    64    64    64    64    64    64
SPDBVg    64    64    64    64    64    64    64    64    64    64
SPDBVg    64    64    64    64    64    64    64    64    64    64
SPDBVg    64    64    64    64    64    64    64    64    64    64
SPDBVg    64    64    64    64    64    64    64    64    64    64
SPDBVg    64    64    64    64    64    64    64    64    64    64
SPDBVg    64    64    64    64    64    64    64    64    64    64
SPDBVg    64    64    64    64    64    64    64    64    64    64
SPDBVg    64    64    64    64    64    64    64    64    64    64
SPDBVg    64    64    64    64    64    64    64    64    64    64
SPDBVg 64 64 64 64 64 64 64 64 64 64 SPDBVg 64 64 64 64 64 64 64 64 64 64
SPDBVg 64 64 64 64 64 64 64 64 64 64 SPDBVg 64 64 64 64 64 64 64 64 64 64
SPDBVg 64 64 64 64 64 64 64 64 64 64 SPDBVg 64 64 64 64 64 64 64 64 64 64
SPDBVg 64 64 64 64 64 64 64 64 64 64 SPDBVg 64 64 64 64 64 64 64 64 64 64
SPDBVg 64 64 64 64 64 64 64 64 64 64 SPDBVg 64 64 64 64 64 64 64 64 64 64
SPDBVg 64 64 64 64 64 64 64 64 64 64 SPDBVg 64 64 64 64 64 64 64 64 64 64
SPDBVg 64 64 64 64 64 64 64 64 64 64 SPDBVg 64 64 64 64 64 64 64 64 64 64
SPDBVg 64 64 64 64 64 64 64 64 64 64 SPDBVg 64 64 64 64 64 64 64 64 64 64
SPDBVg 64 64 64 64 64 64 64 64 64 64 SPDBVg 64 64 64 64 64 64 64 64 64 64
SPDBVg 64 64 64 64 64 64 64 64 64 64 SPDBVg 64 64 64 64 64 64 64 64 64 64
SPDBVg 64 64 64 64 64 64 64 64 64 64 SPDBVg 64 64 64 64 64 64 64 64 64 64
SPDBVg 64 64 64 64 64 64 64 64 64 64 SPDBVg 64 64 64 64 64 64 64 64 64 64
SPDBVg 64 64 64 64 64 64 64 64 64 64 SPDBVg 64 64 64 64 64 64 64 64 64 64
SPDBVg 64 64 64 64 64 64 64 64 64 64 SPDBVg 64 64 64 64 64 64 64 64 64 64
SPDBVg 64 64 64 64 64 64 64 64 64 64 SPDBVg 64 64 64 64 64 64 64 64 64 64
SPDBVg 64 64 64 64 64 64 64 64 64 64 SPDBVg 64 64 64 64 64 64 64 64 64 64
SPDBVg 64 64 64 64 64 64 64 64 64 64 SPDBVg 64 64 64 64 64 64 64 64 64 64 SPDBVi
11101011010110 0
SPDBVp 0 END
```

References

1. Hansen, T. F., Pienaar, J. & Orzack, S. H. A comparative method for studying adaptation to a randomly evolving environment. *Evolution* **62,** 1965–1977 (2008).
2. Hansen, T. F. Stabilizing selection and the comparative analysis of adaptation. *Evolution* **51,** 1341–1351 (1997).
3. Labra, A., Pienaar, J. & Hansen, T. F. Evolution of Thermal Physiology in Liolaemus Lizards: Adaptation, Phylogenetic Inertia, and Niche Tracking. *The American Naturalist* **174,** 204–220 (2009).
4. Bartoszek, K., Pienaar, J., Mostad, P., Andersson, S. & Hansen, T. F. A phylogenetic comparative method for studying multivariate adaptation. *Journal of Theoretical Biology* **314,** 204–215 (2012).
5. Butler, M. A. & King, A. A. Phylogenetic comparative analysis: A modeling approach for adaptive evolution. *The American Naturalist* **164,** 683–695 (2004).
6. Froese, R. & Pauly, D. FishBase.
7. Team, R. C. R: A language and environment for statistical computing. R Foundation for Statistical Computing, Vienna, Austria. 2013. (2014).
8. Paradis, E., Claude, J. & Strimmer, K. APE: Analyses of phylogenetics and evolution in R language. *Bioinformatics* **20,** 289–290 (2004).
